# Phenotype-genotype comorbidity analysis of patients with rare disorders provides insight into their pathological and molecular bases

**DOI:** 10.1371/journal.pgen.1009054

**Published:** 2020-10-01

**Authors:** Elena Díaz-Santiago, Fernando M. Jabato, Elena Rojano, Pedro Seoane, Florencio Pazos, James R. Perkins, Juan A. G. Ranea

**Affiliations:** 1 Department of Molecular Biology and Biochemistry, University of Malaga, Malaga, Spain; 2 CIBER de Enfermedades Raras (CIBERER), ISCIII, Madrid, Spain; 3 National Centre for Biotechnology (CNB-CSIC), Madrid, Spain; 4 The Biomedical Research Institute of Malaga (IBIMA), Malaga, Spain; Icahn School of Medicine at Mount Sinai, UNITED STATES

## Abstract

Genetic and molecular analysis of rare disease is made difficult by the small numbers of affected patients. Phenotypic comorbidity analysis can help rectify this by combining information from individuals with similar phenotypes and looking for overlap in terms of shared genes and underlying functional systems. However, few studies have combined comorbidity analysis with genomic data. We present a computational approach that connects patient phenotypes based on phenotypic co-occurence and uses genomic information related to the patient mutations to assign genes to the phenotypes, which are used to detect enriched functional systems. These phenotypes are clustered using network analysis to obtain functionally coherent phenotype clusters. We applied the approach to the DECIPHER database, containing phenotypic and genomic information for thousands of patients with heterogeneous rare disorders and copy number variants. Validity was demonstrated through overlap with known diseases, co-mention within the biomedical literature, semantic similarity measures, and patient cluster membership. These connected pairs formed multiple phenotype clusters, showing functional coherence, and mapped to genes and systems involved in similar pathological processes. Examples include claudin genes from the 22q11 genomic region associated with a cluster of phenotypes related to DiGeorge syndrome and genes related to the GO term anterior/posterior pattern specification associated with abnormal development. The clusters generated can help with the diagnosis of rare diseases, by suggesting additional phenotypes for a given patient and potential underlying functional systems. Other tools to find causal genes based on phenotype were also investigated. The approach has been implemented as a workflow, named PhenCo, which can be adapted to any set of patients for which phenomic and genomic data is available. Full details of the analysis, including the clusters formed, their constituent functional systems and underlying genes are given. Code to implement the workflow is available from GitHub.

## Author summary

Although rare diseases each affect a small number of people, taken together they affect millions. Better diagnosis and understanding of the underlying mechanisms are needed. By combining phenotypic data for many rare disease patients, we can build clusters of comorbid phenotypes that tend to co-occur together. By using genomic information, we can supplement these clusters and look for related genes and functional systems, such as pathways and molecular mechanisms. We applied such an approach to thousands of rare disease patients from the DECIPHER resources. We were able to detect hundreds of pairs of comorbid phenotypes, and use them to build tens of phenotype clusters. By mapping genes to these phenotypes, based on data from the same patients, we were able to detect related genes and functional systems, such as genes mapping to the 22q11 genomic region underlying a cluster of phenotypes related to DiGeorge syndrome. To ensure that these clusters made sensible predictions, results were validated using literature co-mention, overlap with known disease and semantic similarity measures. These comorbidity patterns, along with their underlying molecular systems, can give important insights into disease mechanisms, moreover they can be used to direct differential-diagnosis of rare disease patients.

## Introduction

Rare diseases are defined as those that affect fewer than 5 people per 10,000 [1]. Correct diagnosis based on observed clinical features is notoriously difficult for rare diseases, as they often show complex or unusual phenotype profiles and there are, by definition, few patients to compare. As a result, their diagnosis can be delayed for many years, with patients being repeatedly passed between specialists [2, 3]. As such, it is important to develop methodology to help with their medical and molecular diagnosis.

To this end multiple personal medicine initiatives have been implemented that obtain and combine patient-related genomic data with environmental and lifestyle information for RDs [4]. Exome sequencing and other forms of genomic diagnostics have greatly accelerated the discovery of novel disease-associated genes and are starting to be used routinely [5, 6]. In addition, there are databases that contain phenotypic information associated with diseases, such as OMIM [7] and Orphanet [8], with the latter focussing on RDs. Both OMIM and Orphanet provide catalogues of human genes and genetic disorders and contain a variety of phenotypic information. To represent phenotypes in a controlled manner, the Human Phenotype Ontology [9] (HPO) was created to provide a comprehensive vocabulary and knowledge base to describe the manifestations of human diseases. It has been used to annotate diseases for both OMIM and Orphanet.

There has been much interest in measuring similarity between diseases based on phenotypes, with applications for understanding disease mechanisms and improving diagnosis and prognosis. Initial work calculated comorbidity indices for prognostic purposes, predicting mortality and other outcomes, such as the Charlson comorbidity index and Elixhauser comorbidity score [10, 11]. More recently, approaches have been developed to construct networks of similar diseases. Goh et al. used a bipartite graph consisting of diseases and genes, using it to connect diseases that share common genetic components [12]. Further studies have built on this work, and it is clear that similar diseases in terms of pathophysiology tend to group together in such networks [13, 14]. As well as diseases, there has been increasing interest in their underlying phenotypes and the connections between them. Akin to the human disease network, pioneering work by Hidalgo et al. built a network of phenotypes based on ICD-9 disease classification codes from hospital records, dubbed the Phenotypic Disease Network (PDN), and showed its potential utility for prognosis [15]. Amongst other findings, they showed that patients with diseases represented by highly connected nodes had higher mortality. More recent work by Bagley et al. used a similar approach, combining it with a set of disease-associated genetic variant data [16]. Verma et al. employed genetic associations between 625,325 SNPs and 541 ICD-9 codes to build a disease-disease network to identify relevant disease connections characterized by underlying genetic associations [17]. Rzhetsky et al. [18] estimated genetic overlap between comorbid phenotypes for 161 disorders, finding significant overlap between several, such as autism, schizophrenia, and bipolar disorder. More recently, studies have used the structure of the HPO to quantify phenotypic similarity between patient symptoms; such is the approach of the highly-cited tool Phenomizer [19]. Tools such as Phenomizer, Orphamizer and Phenolyzer can also predict affected genes given a patient phenotypic profile [19–21]. Other studies have combined phenotypic similarity with data relating to molecular function and interactions at the gene and protein level. XueZhong Zhou et al. [22] constructed a symptoms-based human disease network, finding that more phenotypically similar diseases tend to be more strongly correlated at the genetic level. Moreover their associated proteins interact more extensively. Peng et al. [23] developed the tool PhenoNet, which calculates phenotype similarity and compares it with phenotype-associated modules in the protein-protein interactome. However, few studies have combined phenotypic and genotypic data from the same patients in order to investigate whether patients with similar phenotypes tend also to have mutations in similar genomic regions, or share mutations for different genes that correspond to the same functional systems.

A key repository of phenotypic and genomic data for RD patients is the DatabasE of genomiC varIation and Phenotype in Humans using Ensembl Resources (DECIPHER) database [24]. Its patients have been phenotyped using the HPO vocabulary, allowing them to be compared directly. In terms of genetic data, it consists principally of structural variants such as copy number variations (CNVs), broadly defined as DNA segments at least 1 kb in length that vary in copy number compared to the reference genome. Many of these patients have unusual combinations of symptoms, making precise disease classification difficult; in fact most patients tend not to be diagnosed at the time of submission. However, they often show symptoms that overlap with genetic diseases when considered alone or in smaller groups. We have previously analysed DECIPHER using a network based method that links phenotype and genotype data, predicting multiple novel phenotypes associated with 9p13.3 microdeletion/microduplication syndrome that were then validated [25].

The work presented here is based on the hypothesis that many undiagnosed patients with rare genomic disorders present a mix of symptoms that do not match exactly with the phenotypes described for a known disease, rather they overlap partially with multiple genetic disorders. Therefore, we propose to split the complex phenotype profiles of such patients into their constituent phenotypes in order to look for patterns of co-occurence across many patients; we refer to phenotypes that co-occur in patients as comorbid, using a statistical test to measure the significance of the overlap and deeming phenotype pairs with a test value above a given threshold as comorbid. By comparing such phenotypic overlap with genotypic overlap between these patients, we can look for shared genes and molecular systems underlying the phenotypes.

We present a novel method to identify such groups of comorbid phenotypes within a patient cohort, implemented as a computational workflow named PhenCo. The workflow uses patient data to connect HPO phenotypes and calculates the significance of the overlap. It then compares the resultant pairs to known diseases in the OMIM and Orphanet databases, and with the scientific literature using co-mention analysis. By incorporating genomic data, it also assigns genes to these phenotypes and performs enrichment analysis for functional systems. Finally, it identifies phenotypically coherent clusters of comorbid phenotypes showing enrichment for shared systems.

The tool is intended to help clinicians with diagnosis by suggesting potential phenotypes. For example, when faced with a patient presenting three HPO phenotypes, all of which are found within a cluster of five, the two additional phenotypes may represent missed phenotypes to be tested for. Moreover, any shared functional systems amongst these phenotypes provide additional information on the potential molecular basis of the phenotypes. The workflow can be downloaded directly from the online GitHub repository, https://github.com/Elenadisa/PhenCo, and can be implemented to analyse data from any patient cohort that includes phenotypic data from the HPO alongside genomic annotation. Moreover, by taking advantage of workflow management software, it can be scaled to analyse large datsets including thousands of patients.

## Results

**Fig 1:**
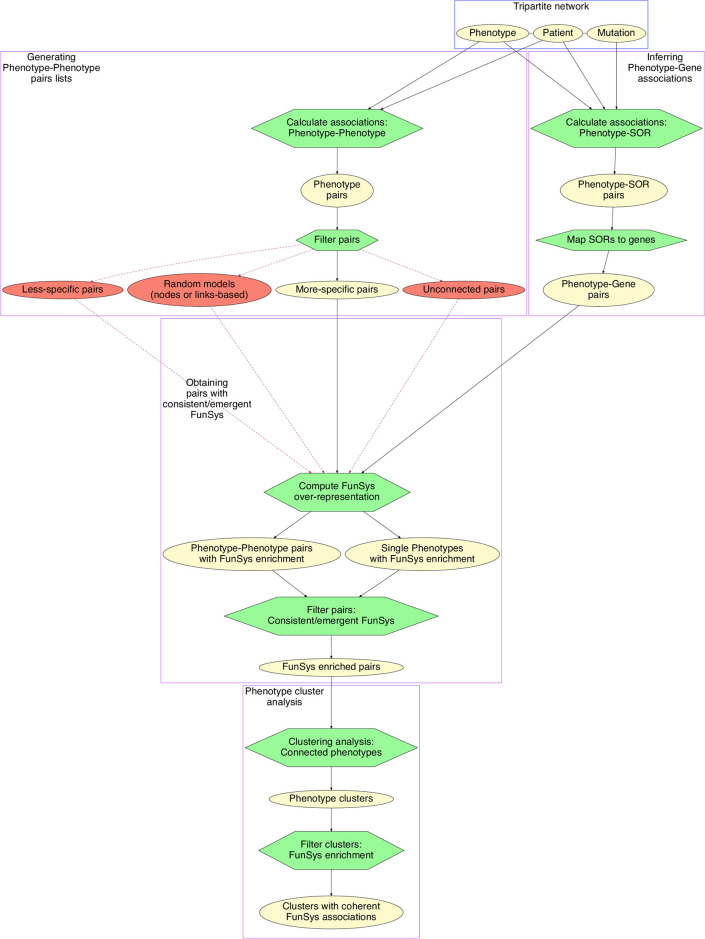
Analysis workflow. Ovals represent data originating from the DECIPHER database: yellow ovals and solid black lines represent the standard pipeline, the red ovals and dashed red lines show the generation of data for comparison with the real data. Distinct processes are contained in purple boxes; the original DECIPHER data is contained in a blue box. FunSys: Functional System, SOR: small overlapping region.

**Fig 2:**
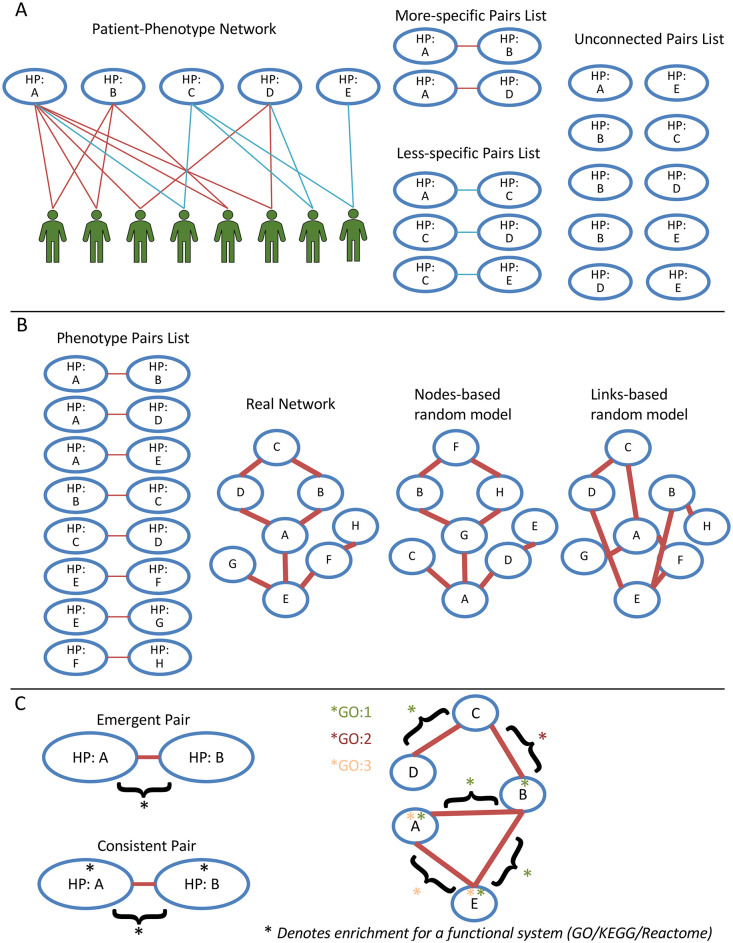
Key workflow components. A) Generation of the phenotype pairs based on patient data. Phenotypes that co-occur in patients were assigned to more-specific or less-specific pairs lists, depending on the connection strength. Note that this is an illustrative example—in real datasets there tend to be far more low-confidence and unconnected pairs. B) Random dataset generation. Performed by shuffling either nodes or links, as indicated. C) Functionally coherent clusters. Left: Emergent Pair: Neither phenotype shows enrichment individually, but the pair does. Consistent Pair: Both phenotypes show enrichment individually, so does the pair. Right: Over 70% of the nodes form part of an emergent/consistent pair for GO:1 or are enriched for it individually, so it is functionally coherent for this FunSys. GO:2 and GO:3 do not meet this threshold.

An overview of the analysis protocol presented in this study is given in Fig 1 and can be divided into four parts. It was developed using the workflow manager AutoFlow [26]. Further details of key components of the workflow are given in Fig 2.

### Properties of the Phenotype-Phenotype datasets and random models

**Fig 3:**
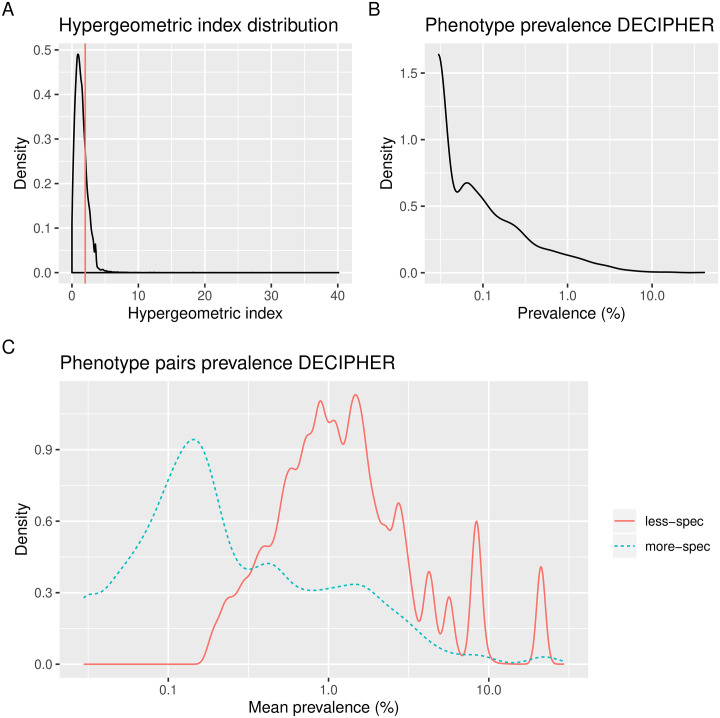
Properties of the Phenotype-Phenotype pairs. **A** Distribution of hypergeometric index values for all Phenotype-Phenotype pairs. The vertical line represents a cut-off of 2 used to separate more and less-specific pairs. **B** Prevalence distribution for all phenotypes in the DECIPHER dataset, defined as the percentage of patients in which a given phenotype occurs. **C** Distribution of average prevalence for pairs of phenotypes in the more and less-specific pairs lists. Figures generated by the PhenCo workflow.

For the 1,758 phenotypes presented by DECIPHER patients, we obtained a total of 36,709 Phenotype-Phenotype pairs. Of these, 8,099 (22%) had a hypergeometric index value greater than or equal to 2; these were termed more-specific phenotype pairs, leaving 28,610 (78%) pairs with a value lower than 2, termed less-specific. The distribution of scores for these sets are shown in Fig 3A. Numbers of patients, HPOs and pairs for the different categories are shown in Table A in S1 Report.

In terms of the prevalence of the phenotypes within DECIPHER, defined as the percentage of patients to which a given phenotype has been ascribed, we see the majority of phenotypes show low prevalence, whilst for a small number it is very high (Fig 3B). This reveals a general tendency of the DECIPHER database, which contains a large number of patients with general phenotypes such as *Intellectual disability* (HP:0001249), as well as a large number of more precise phenotypes that are ascribed to very few patients. When we look at the average prevalence for each pair, we see a marked difference between the more-specific and the less-specific pairs lists, with the more-specific pairs tending to have a lower average prevalence (Fig 3C). This suggests that the pairs in the less-specific list tend to include more general phenotypes that interact with many other general phenotypes. The average prevalence for the unconnected pairs was much lower than for either the more-specific or less-specific pairs lists (Fig A in S1 Report).

### Overlap of the phenotype pairs with known diseases and co-mention of phenotypes and genes in scientific articles

**Fig 4:**
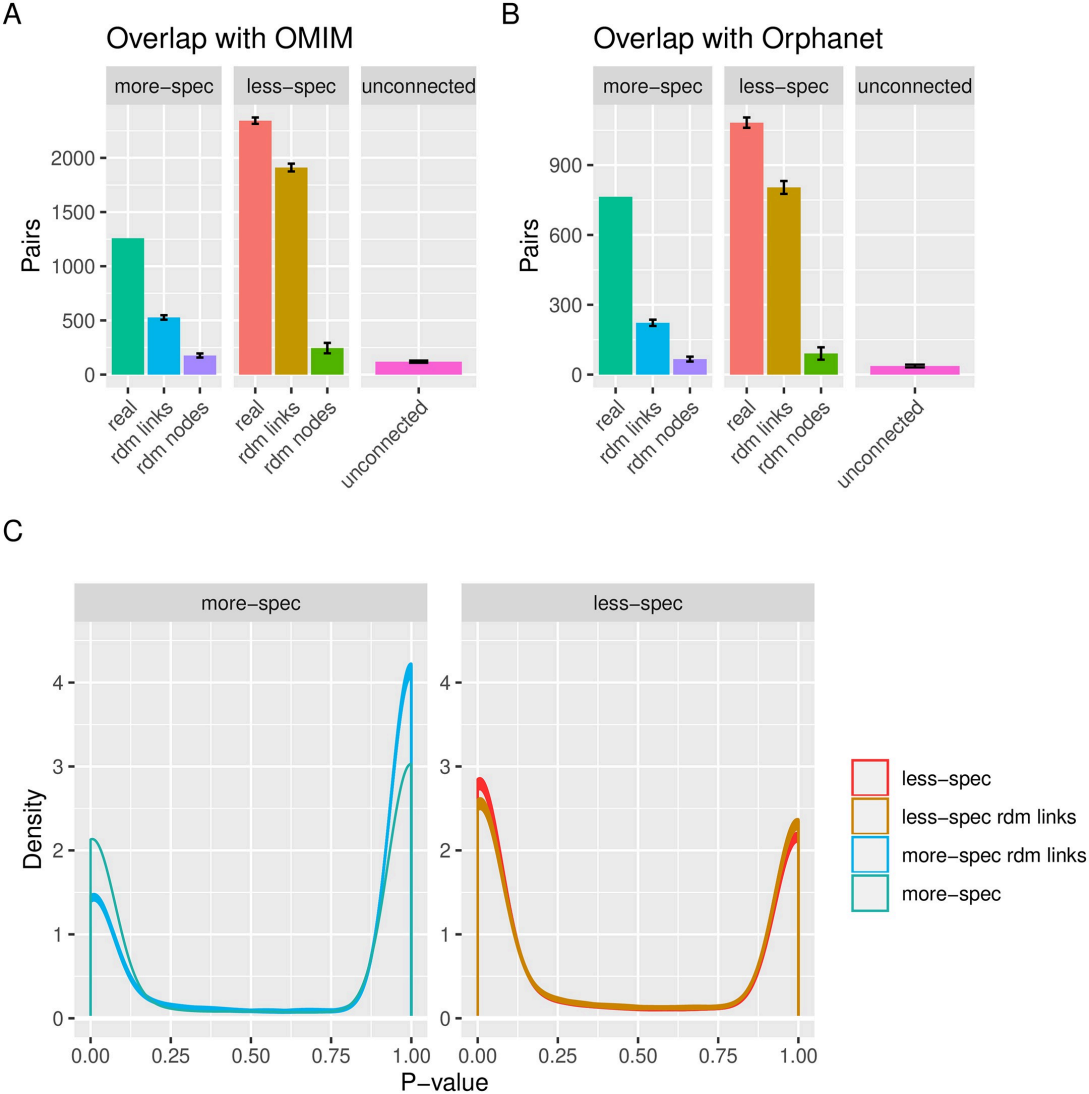
Comparison between the comorbid pairs and external data sources. **A** Overlap of the pairs lists with at least three known diseases from OMIM. **B** Overlap of the pairs lists with at least three known diseases from Orphanet. **C** Distributions of *P*-values obtained for the co-mention analysis, for the more-specific pairs list (left) compared to the less-specific pairs list. In both cases, the distributions are compared to those of the links random datasets. Spec: specific, uncon: unconnected, rdm: random. Figures generated by the PhenCo workflow. More-specific data do not show error bars as a single dataset was used, the full unsampled pairs list.

We compared the Phenotype-Phenotype pairs lists with known diseases taken from OMIM and Orphanet, by counting how many diseases were annotated with both phenotypes for each given pair. We then plotted how many pairs overlapped with disease for the different pairs lists, using thresholds of one, two and three diseases (S1 Report, Section: Overlap of the pairs lists with known diseases). In Fig 4A and 4B we show the results in terms of absolute values found using a threshold of three diseases, for OMIM and Orphanet respectively. In terms of fold change, we see a much greater increase for the more-specific pairs list compared to the links-based random model pairs list for these pairs: 2.4 time as many overlap with OMIM diseases, (1259/527), compared to 1.2 (2344/1911) between real and links-based random models for the less specific pairs. This trend is even more marked for the Orphanet diseases: 3.4 (764/222) for the more-specific vs. 1.3 (1082/804) for the less-specific. However, it should also be remarked the absolute value of overlapping pairs is higher for the less-specific pairs for both disease databases. Comparison with the nodes-based random model gave much higher fold change values, 7.15 (1259/176) for more-specific and 9.56 (2344/245) for less-specific. However, it should be pointed out that the nodes-based randomization swaps the labels of the phenotypes in the network, meaning that each phenotype also changes its degree. On the other hand, the links-based random model keeps the degree for each phenotype the same, and therefore better recreates the properties and structure of the original data. As such, we will focus on the comparison with the links-based random model from now on.

We performed co-mention analysis for each pair within PubMed abstracts. The number of PubMed articles for the phenotypes in each pair were compared using Fisher’s exact test and the distribution of the *P*-values is shown in Fig 4C. Although many pairs of phenotypes are not significantly co-mentioned, pairs in the links-based random models tend to be less co-mentioned than their real counterparts; this difference is more evident for the more specific pairs. Furthermore, the median number of PubMed abstracts for the pairs in the less-specific list is over three times larger than the more-specific pairs, supporting the idea that the phenotypes for the less-specific pairs are far more general (S1 Report, Section: PMID per Pairs List). Co-mention analysis was also performed for the Phenotype-Gene associations used to investigate Functional system (FunSys) enrichment. Co-mention was found for 2810 of the pairs, representing a *Z*-score of 281.5 compared to the random distribution (*p* < 0.0001), which had a mean of 2237.2, and standard deviation 45.5.

### Emergent and consistent functional systems

The pairs lists were analysed in terms of over-representation of functional systems (FunSys), defined as GO-terms, KEGG categories, and Reactome categories. We show here the results for GO-terms, as this resulted in the largest number of consistent/emergent pairs; results for KEGG and Reactome showed similar trends, as shown in S1 Report, Section: Emergent and Consistent functional systems. Over-representation analysis was performed using the genes for each phenotype separately and comparing the results to the union of genes for each pair, to search for emergent and consistent FunSys as described in Fig 2C.

In terms of the total numbers of Phenotype-FunSys associations obtained, for the real data 5592 associations could be found, for 297 distinct phenotypes. This compares to an average of only 942 (s.d. 405) phenotype-FunSys associations in the SOR-gene content simulated data, for 84 (s.d. 17) distinct phenotypes. Although variance was high, in all cases the values found with the real data were higher than the highest values obtained in the distributions of 100 values obtained from the simulations (S1 Table).

**Fig 5:**
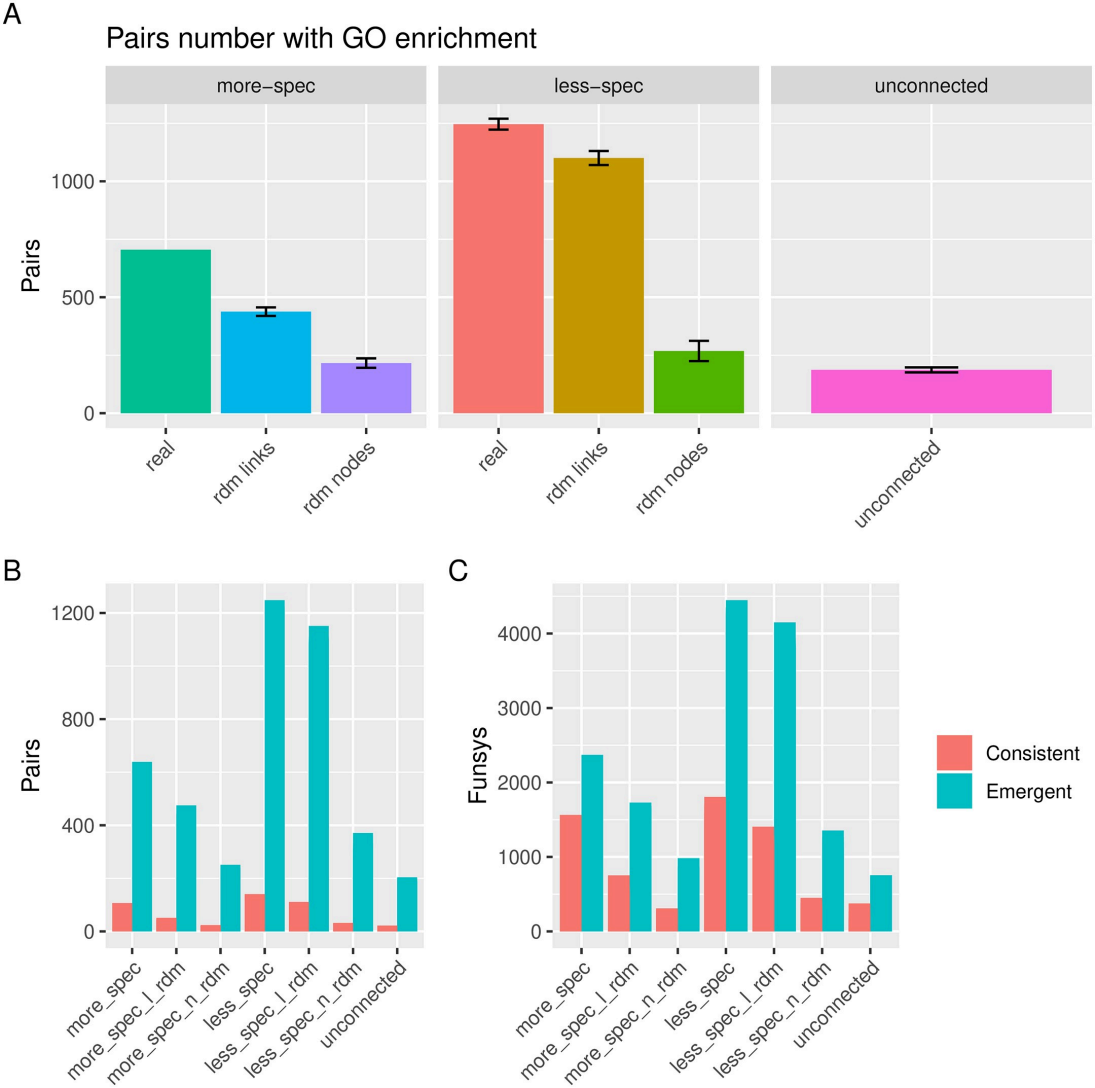
Consistent and emergent functional systems for pairs from the different datasets. **A** Numbers of pairs that show consistent or emergent functional systems. **B** Total numbers of pairs with emergent and/or consistent functional systems. **C** Total numbers of distinct emergent and consistent functional systems among the pairs lists. Figures generated by the PhenCo workflow. More-specific data do not show error bars as a single dataset was used as input to generate the clusters: the full unsampled pairs list.

The total numbers of pairs showing enrichment for at least one emergent or consistent GO-term are shown in Fig 5A. In terms of fold change, the more specific pairs showed a larger increase compared to the links-based random pairs dataset than the less-specific, 1.6 (705/437) vs. 1.1 (1246/1101). However, it must also be remarked that the total number of pairs with at least one emergent/consistent GO-term was higher for less-specific pairs list. In Fig 5B these numbers are broken down to compare the number of pairs with at least one emergent vs. the number of pairs with at least one consistent term, showing that there are many more emergent than consistent terms across all datasets. A similar trend can be seen for total number of GO-terms for each dataset (Fig 5C). For the SOR-gene content simulated datasets only 132 (s.d. 35) pairs could be detected that show emergent/consistent GO terms (S1 Table).

### Functionally coherent clusters

The phenotype pairs that showed consistent/emergent FunSys were used to build networks that were then clustered, creating groups of phenotypes that show overlap in terms of comorbidity and underlying FunSys. As above, we focus here on the results for the GO-term analysis; results for KEGG and Reactome showed similar trends and are shown in S2 Report, Sections: KEGG Enrichment and Reactome Enrichment.

**Fig 6:**
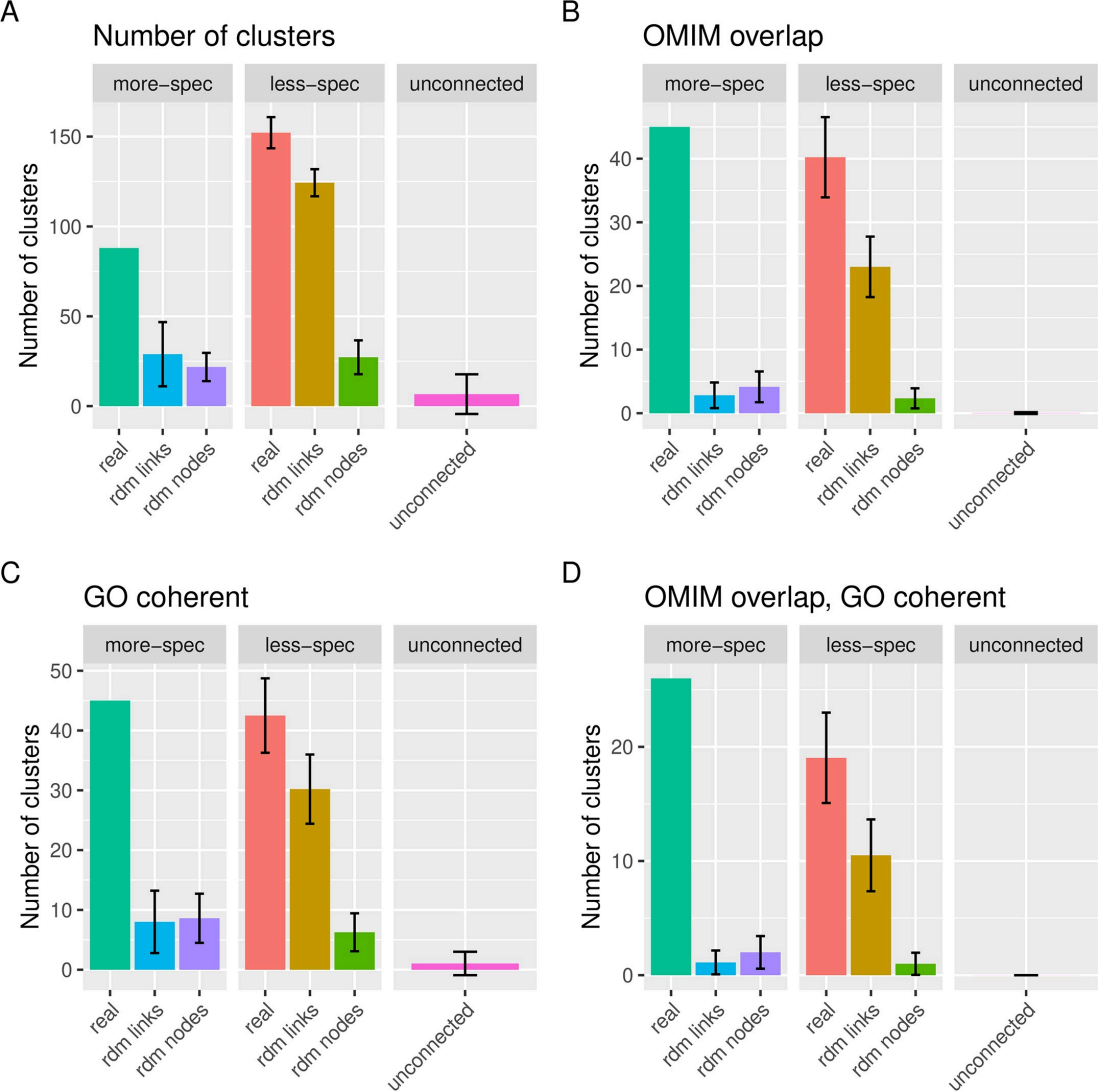
Properties of the clusters found by PhenCo for GO analysis. **A** Total clusters **B** Number of clusters where all phenotypes are found within the same OMIM disease, allowing for one non-overlapping phenotype. **C** Number of clusters considered coherent for at least one GO-term. **D** Number of clusters where all phenotypes are found within the same OMIM disease, allowing for one non-overlapping phenotype, and the cluster is coherent for at least one GO term. Figures generated by the PhenCo workflow.

As shown in Fig 6A, the more-specific phenotype pairs list leads to a lower number of clusters than the less-specific pairs list (88 clusters for the more-specific pairs; 152 clusters for the less-specific). However, compared to their respective links-based random models, the more-specific list finds 3 times as many (88/29), compared to only 1.2 times as many for the less specific (152/124). This shows that the phenotype pairs in the less-specific list are likely to lead to clusters even when the connections are randomized. As such, we should be less confident about how well the phenotypes in these clusters represent realistic and useful groupings of comorbid phenotypes.

This is further demonstrated when we look at the overlap of the clusters from the different phenotype pairs lists with OMIM diseases: despite finding less clusters for the more-specific list, more of these actually overlap with known diseases, 51% for more-specific (45/88), compared to 26% for the less-specific (40/152), as shown in Fig 6B. Moreover, the cluster found using the links-based random model pairs lists found almost no overlap with OMIM diseases: only three clusters showed overlap, compared to 23 for the less-specific random pairs-list.

We then filtered the clusters for each dataset, only retaining the functionally coherent clusters as defined above and in Fig 2C. This filtering reduced the number of clusters greatly, resulting in clusters for which we could be sure that the constituent phenotypes were linked to genes that contributed to common functions. This process resulted in 45 clusters for the more specific pairs lists, 5.6 times as many as found using the links-based random model (45/8), compared to 43 for the less-specific pairs list, only 1.48 times more than the respective links-based random model (43/30), (Fig 6C).

In total, 26 of the 45 functionally coherent clusters found using the more-specific pairs list also overlapped with disease. This compared to only one such cluster for the links-based randomized pairs list. For the less-specific pairs lists, 19 functionally coherent clusters overlapped with diseases, however 11 also overlapped with disease for the links-based randomized pairs (Fig 6D). Full details of these numbers, including overlap with OMIM and Orphanet, as well as overlap with other FunSys (KEGG and Reactome), are shown in S2 Report, finding similar trends.

As well as comparing cluster overlap to those produced using randomized pairs lists, we calculated semantic similarity between the HPO terms within the more-specific pairs list clusters, as a complementary but orthogonal way of quantifying associations between HPOs. We found an average semantic similarity value of 0.38 between all possible pairs of phenotypes within each cluster, which compared to an average of 0.24 (standard deviation: 0.014) in the randomized clusters, representing a z-score of 223.3 and a *P*-value <0.0001. These values are shown in suppoting information (S1 Fig). The average score between phenotypes in the less specific clusters was 0.176 (standard deviation 0.013).

### Examples of clusters obtained by PhenCo

**Fig 7:**
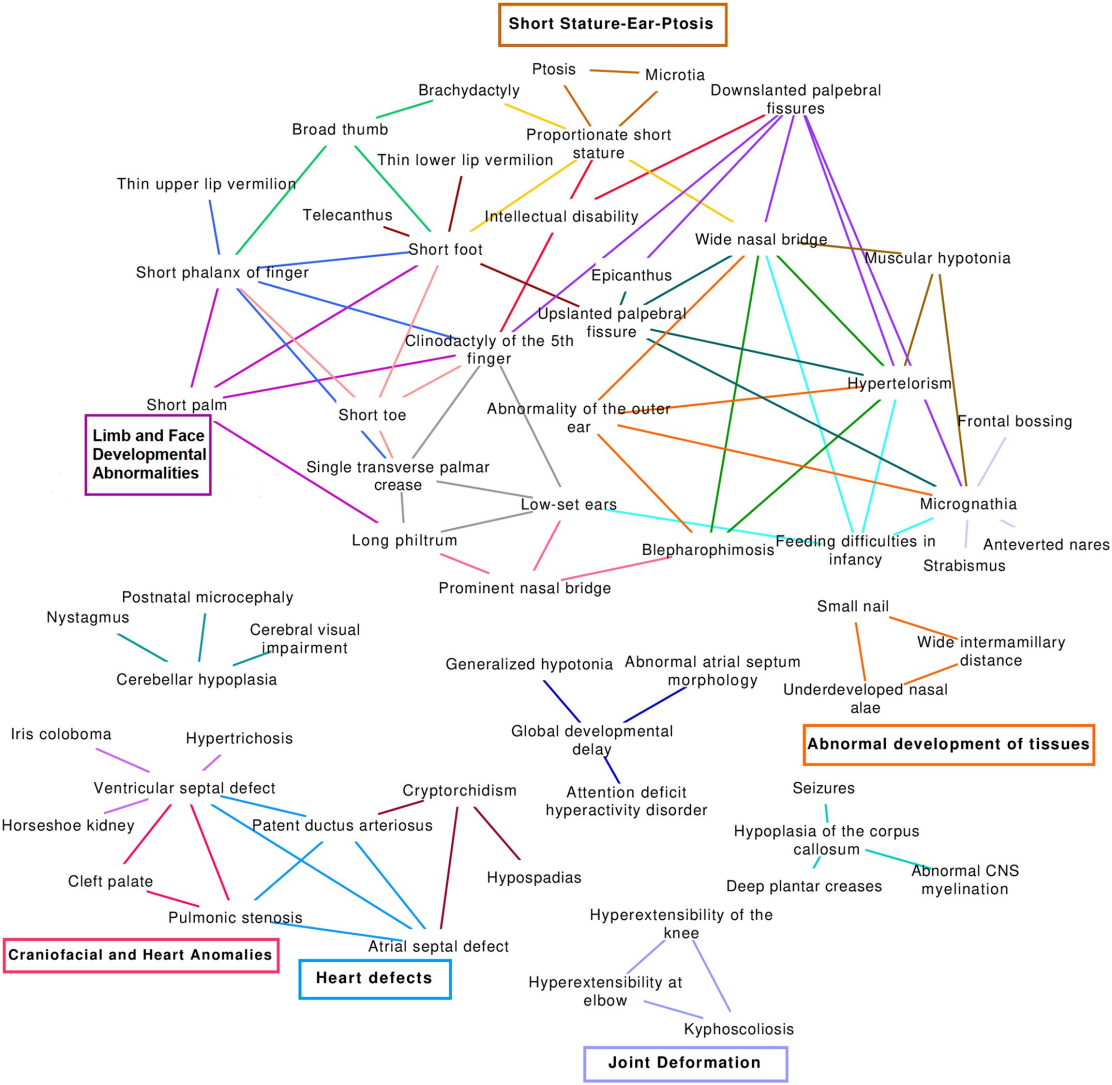
Functionally coherent GO clusters found by PhenCo that overlap with known diseases. All clusters here contain phenotypes that overlap with OMIM diseases, allowing up to one non-matching phenotype. Cluster membership is indicated by different colour links between phenotypes. The clusters that are described in more detail in the text have been labelled using coloured boxes.

In Fig 7 we show all clusters of HPO phenotypes found by our approach that overlap with OMIM diseases, allowing for one missing phenotype, and are functionally coherent for at least one GO term. We can see that there is separation into groups of related clusters, for example the largest cluster shows many phenotypes related to limb and facial feature development, although it also overlaps with different but potentially related phenotypes, such as feeding difficulties in infancy. There is also a separate group of clusters containing multiple phenotypes related to heart, lung and other organ formation. There are also several smaller groups, some of which are more mixed in terms of phenotype. By cross-referencing with the Cluster report (S3 Report) we can obtain the full information related to these clusters. We will now describe a selection of these clusters in more detail, to demonstrate the potential of our approach to better understand the comorbidity relationships within the DECIPHER dataset and the genes and functional systems underlying them.

**Cluster 1**, which we show in Fig 7 with the name Joint Deformation, contains the phenotypes *Hyperextensibility at elbow* (HP:0010485), *Hyperextensibility of the knee* (HP:0010500) and *Kyphoscoliosis* (HP:0002751). Interestingly, these phenotypes overlap with *EHLERS-DANLOS SYNDROME, CLASSIC TYPE, 1; EDSCL1* (OMIM:130000), moreover two patients have been diagnosed as having all phenotypes within this cluster and one with both hyperextensibility phenotypes. These phenotypes all show enrichment for the GO term *opioid receptor signaling pathway* (GO:0038003), which is intriguing given that patients with Ehlers–Danlos syndrome have been found to be refractory to opioid use [27]. In terms of the patients with HPOs in this cluster, two patients who present all three phenotypes posses mutations in the genes *NPBWR2* and *OPRL1*, two G-protein-coupled receptors that interact with opioid ligands (data not shown due to patient confidentiality). Interestingly, patients that present only one or two phenotypes from this cluster tend not to have any affected genes related to opioid receptor signalling, suggesting that these genes may underlie the co-occurrence of all three phenotypes. It should also be noted that these genes are found in a similar location on chromosome 20, as such further analysis would be required to determine whether the phenotype is due to all of these genes, or just a subset. This would be possible if more patients were included, as the SORs linking these patients to the patients would become better stratified.

**Cluster 7**, which we have named Craniofacial and Heart Anomalies, shows a clearer relationship between the phenotypes and enriched functional systems. This cluster contains the phenotypes *Ventricular septal defect* (HP:0001629), *Pulmonic stenosis* (HP:0001642) and *Cleft palate* (HP:0000175). Two of these are found in *DIGEORGE SYNDROME* (OMIM:188400). This syndrome is associated with the gene Claudin 5 *CLDN5* [28]; as might be expected, we find many patients with phenotypes corresponding to this cluster show mutations in this gene, however, we also notice patients with other genes from this family, particularly *CLDN15* and *CLDN23*. In fact, previous work examining CNVs in patients with congenital heart malformations found *CLDN23* amongst other genes affected in three separate patients with distinct types of malformations: hypoplastic left heart syndrome, ventricular septal defect and complete atrioventricular canal. Moreover, the same study also found alterations in the claudin genes *CLDN5*, *CLDN11* and *CLDN18*. However, they did not further comment on these results, rather focussing on known risk genes for congenital heart disease [29]. It should also be made clear that many of the different claudin genes affected in patients in this cluster are found on different chromosomes, suggesting this enrichment is not due to the claudin genes being inherited together in a single mutated region. This is an important point, as *CLDN5* is found on the chromosome segment 22q11, associated with multiple diseases related to the phenotypes in this cluster [30]. The co-mention analysis found 22 abstracts in Pubmed that included all three of these phenotypes, including case-studies [31], syndromes [32, 33], and more general studies of congenital anomalies [34].

**Cluster 12**, which we have named Abnormal Development of Tissues, provides another example of a cluster with disease overlap and for which all phenotypes contain enrichment for multiple GO terms. The phenotypes are *Small nail* (HP:0001792), *Wide intermamillary distance* (HP:0006610) and *Underdeveloped nasal alae* (HP:0000430), which overlap with a range of diseases related to the abnormal development of tissues and organs, such as *RAPP-HODGKIN SYNDROME; RHS* (OMIM:129400). The GO terms associated with these phenotypes are related to keratinization and cornification, as well as *anterior/posterior pattern specification* (GO:0009952), pointing to the involvement of genes relating to these processes in the appearence of these phenotypes and, by extension, various developmental disorders. Going into more detail in terms of patients, two DECIPHER patients suffer from all three phenotypes and have mutations in many of the genes related to the functional systems, in particular for the keratin genes and homeobox transcription factors, although different genes are also mutated in other patients with phenotypes belonging to these clusters, such as T-box genes. A further two patients show mutations in two genes with roles in keratinization and potentially development: Kazrin, Periplakin Interacting Protein. *KAZN*, a component of the cornified envelope of keratinocytes [35], and the elastase 2A gene, *CELA2A*, which is involved in skin barrier function and localized in keratohyalin [36]. Returning to the co-mention analysis, interestingly we see that there is one PubMed article that co-mentions all of these phenotypes, for a disorder named Fryns syndrome [37].

**Cluster 29**, which we have named Heart Defects, contains multiple phenotypes related to pulmonary and cardiovascular disease: *Ventricular septal defect* (HP:0001629), *Pulmonic stenosis* (HP:0001642), *Patent ductus arteriosus* (HP:0001643) and *Atrial septal defect* (HP:0001631), moreover it overlaps with a wide variety of diseases, including *HEART DEFECTS, CONGENITAL, AND OTHER CONGENITAL ANOMALIES; HDCA* (OMIM:600001). There are also less clear results in terms of disease overlap, such as overlap with Noonan syndrome-related conditions, however on closer inspection most of these do indeed often present cardiovascular related symptoms. Less clear at first glance is the association of these phenotypes with the FunSys results: 75% of them are associated with multiple GO terms related to antimicrobial and antifungal immune responses. These phenotypes are also associated with Defensin and Antimicrobial peptide pathways in Reactome (S4 Report), although this data is not shown here as we are focussing on GO categories. Though the relationship between these phenotypes and FunSys are not obvious, it should be noted that increased alpha-defensin production has been shown to be associated with heart disease [38], moreover a genomic deletion in 8p23.1, an area of the genome with a large representation of olfactory receptor/defensin repeats, has been shown to cause congenital heart malformations; conversely, a duplication in 8p23.1 has been associated with atrioventricular septal defects [39]. There are eight patients in this cluster that have affected genes located in chromosome 8p23.1, coding for 14 different defensin genes, providing clear evidence of the link between this process and the phenotypes within this cluster. The co-mention analysis found almost 200 PubMed entries containing these phenotypes, unsurprising given that these phenotypes all have a strong cardiovascular/pulmonary aspect to them.

**Cluster 31**, which we have named Mixed-Short Stature-Ear-Ptosis, also overlaps with diseases and GO terms. It contains three phenotypes: *Proportionate short stature* (HP:0003508), *Microtia* (HP:0008551), and *Ptosis* (HP:0000508). These phenotypes overlap with a range of disorders related to abnormal development and ear malformations. The GO terms associated with these phenotypes involve skeletal development and pattern specification. There are two patients that present all the phenotypes in the cluster; these patients also have mutations in different genes from the HOX family, which have been reported to cause facial dysmorphisms and limb anomalies [40]. These include *HOXA9*, *HOXA10*, and *HOXA11*, important in vertebrate limb development [41]. Other patients with phentoypes belonging to this cluster show mutations in the gene Teashirt Zinc Finger Homeobox 1, *TSHZ1*, which is associated with External Auditory Canal Aplasia/Hypoplasia. There are also three patients that have mutations in the gene Distal-Less Homeobox 5 *DLX5*, which is associated with diseases such as Split-Hand/Foot Malformation 1 With Sensorineural Hearing Loss. Dlx homeobox family genes are also expressed in craniofacial primordia, developing brain, and limbs in vertebrates [42]. Another patient has a mutation in the gene Gastrulation Brain Homeobox 2, *GBX2*, which is related to *CHARGE SYNDROME* (OMIM:214800), another disease that overlaps with the cluster. Finally, there is one patient with mutations in the gene Ras-Related Protein Rab-33B, *RAB33B*, which is associated with *SPONDYLOEPIMETAPHYSEAL DYSPLASIA, FADEN-ALKURAYA TYPE; SEMDFA* (OMIM:616723).

**Cluster 47**, which we have named Limb and Face Developmental Abnormalities, is a particularly large cluster, containing the phenotypes *Long philtrum* (HP:0000343), *Short foot* (HP:0001773), *Clinodactyly of the 5th finger* (HP:0004209), *Short phalanx of finger* (HP:0009803), and *Short palm* (HP:0004279). A handful of GO terms related to the regulation of muscle are associated with this cluster, such as *positive regulation of muscle hypertrophy* (GO:0014742). There are also various OMIM diseases associated with this cluster, including *OSTEOGLOPHONIC DYSPLASIA; OGD* (OMIM:166250) and *FIBROCHONDROGENESIS 1; FBCG1* (OMIM:228520), characterized by abnormal bone growth, distinctive facial features, dwarfism and other skeletal abnormalities. Interestingly, many of the DECIPHER patients have mutations affecting miRNAs. For example, two have miR-214 affected: accumulation of this miRNA has been observed at initial stages of cell differentiation, and it participates in muscle cell differentiation [43]. Other patients have mutations in miRNAs related to muscle growth and mTOR signalling, important in multiple stages of development. For example, miR-199A may regulate targets within the Akt-mTOR signaling pathway [44]. Other patients have mutations in mTOR itself, suggesting that mutations in different parts of the genome can lead to the same effects at the molecular level, resulting in similar phenotypes.

There were also clusters that showed enrichment for FunSys categories that did not overlap with any known diseases. For example, **Cluster 20**, which we have named Anomalies of the Kidney and Lower Thorax, comprises the phenotypes *Abnormality of the kidney* (HP:0000077), *Abnormality of the uterus* (HP:0000130), *Anal atresia* (HP:0002023), *Renal cyst* (HP:000010). Three quarters of these are enriched for *steroid biosynthetic process* (GO:0006694) and *cellular hormone metabolic process* (GO:0034754) terms. Two patients with phenotypes in this cluster have mutations in genes related to the sonic hedgehog signaling molecule, *SHH*, involved in the development of the nervous system and multiple organs. Interestingly, many other patients with phenotypes within this cluster show mutations in genes involved in cholesterol metabolism, making it tempting to speculate a role of cholesterol in signalling related to these phenotypes, although more work would be needed to investigate this further.

### Use of PhenCo comorbid clusters for patient diagnosis

The use of PhenCo to find comorbid phenotype clusters showing FunSys enrichment allows us to better understand patterns of phenotype co-occurrence accross rare diseases and relate this information to the underlying genes and mechanisms involved. On a more practical level however, it is also important to understand and evaluate how these clusters can be of use for the diagnosis of patients with undiagnosed rare diseases. We have done this in two ways: by showing practical examples involving real patients, and through systematic evaluation.

Unfortunately, the full patient report generated by the workflow cannot be published as supplementary material, due to confidentially agreements necessary to use the DECIPHER patient data. However, we have shown two examples of patients for whom data has already been made publicly available.

The first example, patient 254518, has a 2.53 Mb *de novo* heterozygous deletion on chromosome 14q32.2, affecting 25 genes. The patient has been diagnosed with ten phenotypes that overlap with clusters, full details of which can be seen in the **Patient Details** (S6 Report). Of these, the patient holds four phenotypes that match cluster 32. This cluster has a total of 5 phenotypes, all related to craniofacial misformation, including Micrognathia, Blepharophimosis and Abnormality of the outer ear. Patient 254518 holds all these phenotypes except for *Wide nasal bridge* (HP:0000431). As such it seems likely that this may be a missed phenotype for this patient, given its co-cluster membership representing potential comorbidity. In terms of associated FunSys, cluster 32 is associated with the GO term GO:0006898, *receptor-mediated endocytosis*. Endocytosis is important for maintaining the physio-metabolic equilibrium of cells and is associated with a wide range of diseases, having a key role in phenotypes like muscle weakness, hypertrophic cardiomyopathy and mental retardation [45].

Other phenotypes that can be proposed for this patient based on cluster overlap analysis include *Clinodactyly of the 5th finger* (HP:0004209), *Brachydactyly* (HP:0001156), *Prominent nasal bridge* (HP:0000426), *Ptosis* (HP:0000508), *Short foot* (HP:0001773), *Long philtrum*, (HP:0000343), and *Downslanted palpebral fissures* (HP:0000494). Interestingly, the phenotypes of this patient alongside the potential missed (or unrecorded) phenotypes found through PhenCo are associated with other known chromosomic disorders [46].

Another example, patient 256892, has a very large, 9.91 Mb *de novo* heterozygous deletion on chromosome 7, overlapping multiple cytogenic bands and a total of 123 genes, including an important cluster of HOX genes, as well as important immune system genes such as *IL6* and genes involved in epigenetic processes such as *HDAC9*. Perhaps unsurprisingly, this patient displays a large number of pathological phenotypes, as shown in the the **Patient Details** (S6 Report). Furthermore, this patient has nine phenotypes that overlap with PhenCo clusters, including complete overlap with cluster 31, the short stature-ear-ptosis cluster described above, an unsurprising finding given the overlap of the deletion CNV with the HOX genes. However, the patient also holds phenotypes that show partial overlap with other clusters, as we can see in the **Patient Details** (S6 Report). For example, cluster 52 contains two phenotypes, *Brachydactyly* (HP:0001156) and *Short foot* (HP:0001773), phenotypes that can be caused by phalanx deformation [47], that overlap with the patient profile. This cluster contains two further phenotypes, *Broad thumb* (HP:0011304) and *Short phalanx of finger* (HP:0009803), which are not present in the patient’s profile, and as such could represent missed (or unrecorded) phenotypes for that patient. Interestingly, although this cluster is made up of phenotypes related to the malformation of hands and feet, it is associated with several GO terms related to cardiac and skeletal muscle (S3 Report). Brachydactyly is usually is present as part of complex syndromes with phenotypes like hypertension, mental retardation and skeletal involvement. Among the syndromes described, some are caused by mutations in HOX genes [48].

Whilst these examples show potential use cases for PhenCo, it is also important to perform more systematic analysis to ensure that we can be confident that the PhenCo cluster results do indeed contain groups of phenotypes that tend to co-occur, so that we can have confidence in the additional phenotypes it can predict.

To perform such validation we took all 2940 patients who have at least one phenotype mapping to at least one cluster. We then calculate, for each patient phenotype-cluster combination, how many of these clusters contain other phenotypes held by the same patient. We then compare these results to a dataset produced by randomizing the cluster-phenotype assignation, but using the same phenotypes. We find that, if a patient has a phenotype assigned to a given cluster, he is much more likely to have other phenotypes within the same cluster, compared to the random cluster-phenotype assignments (S2 Table). This suggests that using the clusters to propose novel phenotypes that might have been missed or unrecorded during a patient evaluation is a valid approach, as we are able to recapitulate their original phenotypes in addition to suggesting new ones. The putative phenotypes must be evaluated by a clinician, however they represent an important avenue to explore to improve patient diagnosis.

### The use of prior knowledge-based methods to predict Phenotype-Gene associations

We looked at the ability of established methods, namely Phenolyzer [20], Phenomizer and Orphamizer [19, 21], to predict genes for the patients in the studied DECIPHER cohort. These methods use data from OMIM/Orphanet directly, as such it is not possible to make a direct comparison with PhenCo, due to the lack of a suitable reference dataset. Therefore, we examined their ability to predict putative affected genes for each patient, based on their respective phenotypic profiles, and compared these predicted genes to the *de novo* CNVs for the same patients. The results are shown in S3 Table. We see that the methods varied greatly in their ability to predict genes within patient CNVs, with the top performing method predicting them for around one quarter of patients.

### Cluster membership of individual patients

We further investigated cluster membership for the DECIPHER patients. In total, 3,021 of the 3,374 patients had at least one phenotype that was found in a cluster (2,939 for GO, 2,339 for KEGG and 2,609 for Reactome). Of these, 1,144 overlapped with a cluster with coherent functional annotation (1,024 for GO, 367 for KEGG and 298 for Reactome). Full details are given in Fig A in S6 Report.

**Fig 8:**
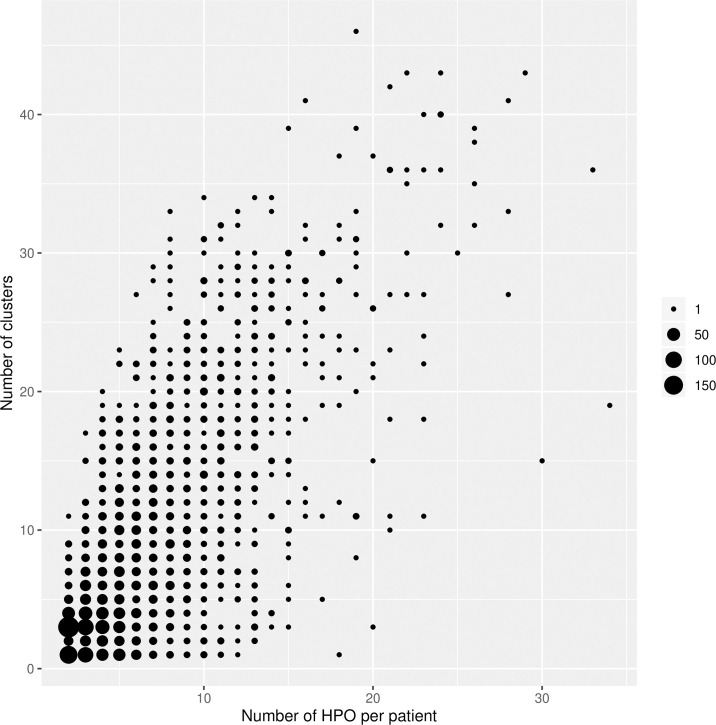
Cluster membership per patient. Number of distinct phenotypes diagnosed for patients against the number of clusters to which these phenotypes belong. Size of point represents number of patients.

We also investigated whether patients tended to belong to a small number of clusters or to many, as this could give an idea of whether their mutations tended to affect the same systems, or multiple systems. As shown in Fig 8, which plots the number of distinct phenotypes held by patients against the number of clusters they belong to, there is a clear tendency for patients with a greater number of phenotypes to map to a greater number of clusters. However, there is a large amount of variation here, and it appears that there is a spectrum: whilst many patients hold phenotypes that correspond to only one or two clusters, others hold phenotypes that belong to many different ones.

## Discussion

The co-occurrence of diseases and phenotypes has been studied from many angles, using comorbidity data from hospital records [15], by linking phenotypes based on the ontological structure and information content of the HPO [19, 23], text-mining based techniques applied to the medical literature [49] and taking MeSH metadata from PubMed [22]. However, few studies have combined phenotypic and genomic data obtained from the same patients [17], and fewer still have provided workflows or software for this purpose. Our workflow, PhenCo, does this, using phenotypic co-occurence within a cohort to find comorbid phenotypes, incorporating random models and comparing the results with external data sources to provide validation. It also uses genomic data to ascertain potential affected genes for these patients and uses them to search for FunSys associated with the phenotypes. Finally, it looks for pairs of phenotypes that are enriched for the same FunSys and/or show enrichment when their underlying genes are combined, and uses these pairs to obtain clusters. This allows clinicians and researchers to look for connections between the phenotypes within their cohort and potentially find out the underlying processes causing them. By examining the patients that present phenotypes belonging to these clusters, the user can obtain hints as to what further studies to perform, both in terms of additional phenotypes for that patient, as well as the underlying biological mechanisms.

The workflow was applied to the DECIPHER database, to test its ability to find associated phenotypes in a highly heterogeneous cohort of undiagnosed patients with complex disorders and a range of CNVs and chromosomal aberrations. These patients have been phenotyped by clinicans from different centres in multiple countries and the level of phenotyping is highly variable, with some patients profiles containing a few, very general phenotypes and others having far more precise ones. This high variability motivated the use of the hypergeometric index to divide the phenotype pairs between “more-specific” and “less-specific”, as the less specific pairs tend to be formed between very general and highly prevalent terms that tend to co-occur with many other terms. We can therefore expect these pairs to be rather uninformative for differential diagnosis. The hypergeometric index is analogous to a contingency table based approach, such as Fisher’s exact test, meaning that only pairs that show specific co-occurence with each other should obtain a value of at least two, equivalent to a *P*-value of 0.01, which we used as the cut-off threshold. Other methods are available and have been compared for related purposes [50], however we used the hypergeometric index for the reasons mentioned above. We also used this index for associating phenotypes with genes; this method has been shown to outperform others in previous work [25, 51].

Nevertheless, the choice of threshold requires justification, and the bulk of the initial results presented here are concerned with demonstrating that the more-specific pairs give stronger signal, in terms of overlap with diseases and the biological literature. Although the less-specific pairs lists actually contained more pairs of phenotypes found in known diseases, this is likely a result of these pairs often containing very general HPO phenotype terms. These terms have relatively high prevalence, and can co-occur with many other phenotypes; as such the association scores for these pairs tends to be rather low. These general terms also tend to occur in a large number of diseases. So, the less-specific pairs list is likely to contain multiple pairs of unspecific, common phenotypes which in turn leads to a large number of pairs that co-occur within known diseases. As an example, if we take the term *Intellectual disability*, this occurs in 791 of the less-specific pairs, but only 57 of the more-specific pairs. Moreover, this term is associated with 703 OMIM diseases. Therefore, the results should be interpreted in terms of the comparison with the random models, in particular those randomized by swapping links between nodes, as these keep the number of pairs per phenotype the same and as such, retain the phenotype prevalence in the dataset the same as for the original data. Indeed, we see a large overlap with the random model for the less specific pairs, for both OMIM and Orphanet (Fig 4A and 4B) despite these connections being made at random—the unspecific phenotypes remain connected to many other unspecific phenotypes due to their high prevalence in the set, and these pairs, though generated by random, still overlap with many diseases. It should also be made clear that we should not expect perfect overlap with disease databases, given the nature of the cohort data (undiagnosed, complex phenotype profiles and CNVs), as these databases are by definition for known diseases and often encompass loss of function mutations that may affect both copies of a specific gene.

These findings are mirrored by the co-mention literature analysis, which finds similar results for the less specific pairs and links-based random-model, indicating that the phenotypes in these pairs tend to be co-mentioned, regardless of whether they are connected based on co-occurence within patients, or just connected at random (Fig 4C). This is in contrast to the results for the more-specific pairs, where there is a clearer separation, indicating that when phenotypes are connected based on more specific co-ocurrence, as calculated by the hypergeometric index, they are more likely to co-occur in the literature than when the same phenotypes are connected at random. However, it should be noted that co-occurence is also found for the randomized connections, albeit to a lower extent.

The genes corresponding to each phenotype and each phenotype pair were used for over-representation analysis, finding that the less-specific pairs show a higher number of consistent and emergent FunSys compared to the more-specific pairs (Fig 5). This is a somewhat curious finding, although it may be due to the total number of genes mapping to the less-specific pairs being higher (mean of 673 vs. 387 for the more-specific pairs list, S1 Report, Section: Number of genes mapping to each phenotype pair). Interestingly, the randomization by node shuffling leads to very low amounts of enrichment in both more and less-specific pairs, showing that it is not intrinsic to the number of genes in each phenotype, rather the pairs of phenotypes. In fact, the average number of genes per phenotype is similar for both datasets (mean of 244 per phenotype for more specific pairs and 266 per phenotype for less-specific pairs), again suggesting that the increase is due to the way the pairs form in the different data-sets.

In any case, the purpose of obtaining pairs with emergent and consistent FunSys was to create input for the clustering process, to identify groups of comorbid phenotypes with similar etiology, in terms of underlying FunSys. This led to more clusters for the less-specific pairs, as well as the random datasets, in line with previous results. However, a much larger proportion of the clusters for the more-specific pairs show overlap with OMIM diseases (51% vs. 26%), moreover when compared to their respective randoms, the results for the more-specific pairs are relatively much larger than the less-specific. In fact, whilst OMIM overlap was found for 45 of the more-specific clusters, it was only found for on average three of the clusters obtained using the links-randomized more-specific pairs. Taken together, this suggests that the process of obtaining the more specific pairs based on more significant associations and further filtering them using the FunSys enrichment analysis leads to more biomedically relevant clusters.

A similar finding was found in terms of functional enrichment for the phenotypes and phenotype pairs within the clusters. The more-specific pairs led to a much larger proportion of functionally coherent clusters compared to the random models than the less-specific pairs. Many of these clusters also contained phenotypes that were co-mentioned within PubMed article abstracts, which often included known syndromes containing these phenotypes. Looking in more detail at these coherent clusters, we find multiple clusters where the phenotypes show relevant underlying FunSys. Moreover, by looking at the patients that contribute to these clusters and their affected genes, we see that in many cases we can form novel hypotheses and suggest further phenotypes and FunSys to study. Such approaches might be combined with exciting new methods that connect laboratory test result data from patient records with HPO phenotypes [52].

More generally, the discovery of functionally coherent phenotype clusters has many potential uses for further understanding comorbidity relationships. For example, by examining overlap with known diseases and underlying FunSys, we can form hypotheses regarding how affected functional systems lead to distinct phenotypes and ultimately disease. Thus by combining diagnoses for multiple patients we are able to identify patterns that cannot be detected when considering them individually.

These clusters also have potential applications for aiding individual patient diagnosis, as patients assigned phenotypes belonging to a given cluster may present other phenotypes within the cluster that have not yet been identified, suggesting additional phenotypes for the practitioner to test for. We have shown the applicability of this approach in terms of patient case studies and through a systematic approach (S2 Table). Furthermore, we have shown that the phenotypes within the clusters show much higher semantic similarity based on the HPO ontology than would be found by assigning the same HPO terms randomly. The less specific clusters showed lower semantic similarity values, again indicating more general, less informative phenotypes.

In terms of cluster membership, many patients have been assigned phenotypes belonging to several different clusters, whilst others have various phenotypes within the same cluster (Fig 8). This shows the complexity of the patients within DECIPHER and underlines the heterogeneous phenotypic patterns of rare disease patients with underlying CNVs. However, it should be considered that DECIPHER patients come from multiple centres all over the world, and despite the use of the unified vocabulary of phenotypic terms provided by the HPO, it is feasible that teams in different centres tend to phenotype to different levels of specificity, which might also contribute to the differences in the phenotypic profiles of patients.

This work contributes to the growing field of co-occurence based phenotype network study. Whilst many previous studies have investigated comorbidity alone, often using electronic health records and billing codes [15, 53–56], fewer approaches have looked at genes shared between phenotypes [22, 23, 57, 58]. Moreover, most of these studies have used disease/phenotype-gene mapping taken from databases, rather than using patient data directly. Such is the case of Chen et al., where they used relations mined from the United Medical Language System, containing clinical manifestation data [57], and Peng et al., where they took HPO-gene data from the HPO resource [23]. Here we have used data from the DECIPHER resource exclusively: these patients tend to present rare and complex phenotypes and are not diagnosed at the time that their data is deposited in the resource, making it vital to make the most of all available data, not relying on information from disease databases, which might not match their phenotypes comprehensively.

In addition to approaches based on comorbidity calculated using patient-cohort data directly, it is important to present our work within the context of other methods that can be used to predict affected genes based on patient profiles. These are often based on disease databases that contain manually curated annotation relating genes to phenotypes, such as OMIM and Orphanet, rather than using phenotypic and genotypic information for the same patients. We will refer to these methods as knowledge-based. They use techniques such as word matching and exploiting the structure of the HPO [19–21]. We have not performed a direct comparison between PhenCo and these tools, as there is no external reference to use to assess performance: there would likely be a bias in favour of the knowledge-based methods if we were to use OMIM/Orphanet disease-gene annotations.

To demonstrate the differences in focus between our approach and the knowledge-based approaches, we investigated the ability of three established tools in this area to predict genes for the patients in our cohort based on their phenotypic profiles. Given that these patients have been characterized as having *de novo* deletions or duplications, we make the assumption that the genes leading to their phenotypes should be located within these regions. The tools are able to predict genes within the CNVs for a number of DECIPHER patients, although the different methods vary greatly. Unsurprisingly, when the confidence thresholds for these methods are relaxed, these values increase. Our method is complementary to these tools, and future work might investigate combining patient-data driven approaches such as ours with knowledge-based approaches.

It should also be made clear that, just because the knowledge-based approaches were unable to detect genes corresponding to the mutated regions of the DECIPHER patients, it does not necessarily mean that our approach is finding the responsible genes or functional systems, just that it is able to find genes in regions corresponding to the patient’s mutation. Thus, it is also important to investigate the specificity of the predictions. However, as mentioned above, this task is hampered by a lack of gold-standard dataset, particularly for rare diseases. To overcome this, we searched for our predicted phenotype-gene associations within the scientific literature, looking for co-mention between the two in PubMed abstracts. We found a significantly larger number of co-mentions for real associations compared to random. Although such an approach is not perfect, it indicates that we are finding real phenotype-gene connections within our cohort.

Another important factor to take into account relating to the DECIPHER data is that we only have data on whether the genomic region has been deleted or duplicated. However, we do not have any higher resolution data, such as for SNPs and other small variants. These might contribute to the manifestation of the phenotype, especially in the case of deletions, as any recessive mutation that would not be expressed in the presence of a functional allele on the other copy of DNA would be unmasked, to function as if it were a dominant/hemizygous allele. Such information would be useful, as SNPs could be mapped directly to genes, moreover further information about disease, including inheritance, could be incorporated into the validation procedures. Ongoing projects such as the Deciphering Developmental Disorders study have already begun to look at the impact of recessive mutations [59]; it would be possible to adapt PhenCo to work with such data, however it should be made clear that the results of the analysis, in terms of associated genes and functional systems may be quite different to those found here looking at *de novo* CNVs.

To conclude, by combining genomic and phenotypic data from thousands of patients we have been able to build clusters of phenotypes that co-occur in many patients that have mutations in genes related to similar functional systems. This approach has myriad potential applications for other diseases; by making the workflow available, it can be applied to any other data set containing the requisite information: all necessary scripts can be downloded from the GitHub repository described above, to be applied to any dataset containing HPO annotation of patients alongside their genomic data.

As genome sequencing becomes routine and incorporated into national health systems, as is predicted to occur in the next decade [60], it will be combined with advances in electronic health records mining to obtain phenotype data [52, 61]. This workflow will be able to take advantage of this data, and by applying it to larger cohorts of patients, its ability to find reliable phenotypic relationships will increase. Such efforts are already underway, and have led to the association of variants and genes with disease [5, 62]. Future work will be needed to adapt the workflow to the new challenges, for example mapping variants to genes, as well as incorporating non-coding mutation data: these processes will be aided greatly as non-coding genome annotation projects mature, and initiatives like GTEx that try to map expression quantitative trait loci become more comprehensive [63, 64]. This will enable the workflow to be used not only for CNVs and structural variants, but also for SNPs and other mutation types.

## Materials and methods

### Description of datasets

This study was performed using data from an anonymized patient cohort downloaded from the DECIPHER database Version 9.12 [24] through a Data Access Agreement with the database consortium. Information was initially obtained for 20,924 patients with rare diseases. Of these, we selected those that have both their clinical profile described in terms of a list of phenotypes from the Human Phenotype Ontology (HPO), and their genetic mutations described in terms of genomic location. We then further filtered these patients according to inheritance type, selecting those with *de novo* CNV mutations, as these can be considered more likely to represent pathogenic mutations. This resulted in a final cohort of 3,374 patients, presenting 1,758 distinct HPO terms.

Phenotype-disease annotation data were downloaded from the OMIM and Orphanet databases. These databases provide, for each disease, a list of HPO annotations that correspond to it.

### Overview of workflow

This workflow can be subdivided into distinct processes:

In ***Generating Phenotype-Phenotype pairs lists***, phenotype-patient data is used to produce a list of co-occurring phenotype-phenotype pairs. Randomized datasets are also generated for comparison. In ***Inferring Phenotype-Gene associations***, the genes corresponding to these phenotypes are obtained using a patient-phenotype-genotype network for the same patients. In ***Obtaining pairs with consistent/emergent FunSys***, over-representation analysis is performed to identify functional systems (FunSys) associated with the phenotype-phenotype pairs. These pairs are then used for ***Phenotype cluster analysis***, to build a network and identify clusters containing multiple phenotypes that show over-representation for the same FunSys, referred to here as functionally coherent clusters.

Validation was performed by comparing the results using the more-specific Phenotype-Phenotype pairs to the less-specific pairs and random datasets, examining overlap with known diseases using data from OMIM and Orphanet, and looking for co-mentioning of the phenotype pairs within PubMed abstracts.

We now describe the sections in greater detail.

### Generating Phenotype-Phenotype pairs lists

Patient data was combined to build a Phenotype-Patient bipartite network. This was used to generate association values between phenotypes based on patient overlap using the hypergeometric index [65], implemented in NetAnalyzer [51], a tool for network analysis that can calculate associations within different layers of a network. This index was used to decide which pairs of phenotypes showed significant co-occurence amongst the DECIPHER patients, which we refer to as the more-specific pairs list.

#### Phenotype-Phenotype comorbidity pair datasets

In total, three datasets were constructed, as shown in Fig 2A.

1. More-specific: Pairs of phenotypes with a hypergeometric index value greater than or equal to 2, representing an *α* ≤ 0.01. These pairs tend to show low prevalence, as shown in Results. The use of this threshold is commented upon in the discussion section. These pairs can be considered comorbid.
2. Less-specific: Pairs with a hypergeometric index value lower than 2. There were many more less-specific pairs in the dataset than more-specific, we therefore sampled the less-specific pairs to obtain lists of the same length as the more-specific pairs list for further comparisons, producing 50 replicates.
3. Unconnected: We also used lists of pairs of phenotypes that shared no connections at all within the original bipartite Phenotype-Patient network, again sampling the unconnected pairs to obtain lists of the same length as the more-specific pairs list for further comparisons, producing 50 replicates.

#### Random pairs datasets

In addition, we built random-pair datasets as shown in Fig 2B, by taking the real more-specific and less-specific pairs lists and randomising them in the following ways:

1. Nodes-based random model: This involved randomising the phenotype-labels in the network, which keeps the pairs lists the same in terms of network properties, but means that each phenotype has a different number of connections, changing their degrees.
2. Links-based random model: The connections between the phenotypes were randomized, keeping the number of connections (degree) the same for each phenotype in the network. Other properties, such as the cluster coefficient and diameter, changed slightly (S4 Table).

These sampling/randomisation procedures were repeated 50 times for each pairs list, producing 50 replicates per dataset.

### Validating pairs using disease databases and PubMed co-mention

HPO annotation for OMIM and Orphanet diseases was then used to investigate overlap between Phenotype-Phenotype pairs and known diseases, by counting the number of diseases annotated with both phenotypes. It should be made clear that in this case we were looking for partial overlap with known disease, based on the rationale that although the patients in DECIPHER have undiagnosed pathologies, when we combine the HPO information across multiple patients, we should still be able to find pairs of phenotypes that tend to co-occur, even if the full phenotypic profiles are different.

Further validation was performed by searching in PubMed for abstracts in which both phenotypes were mentioned. This was conducted using the NCBI Entrez Programming Utilities API in the following manner: firstly, the different textual descriptions for a given HPO term (synonyms) were retrieved. Then, all the PubMed IDs for articles whose abstracts mentioned any of these synonyms were obtained. This resulted in a list of HPOs and the corresponding PubMed IDs where the term appears. These PubMed ID lists were then compared for each Phenotype-Phenotype pair in the network using Fisher’s exact test to detect pairs showing significant overlap.

### Inferring Phenotype-Gene associations

Pathological phenotypes were associated with genes using the phenotype and mutation data for the same DECIPHER patients used to identify the phenotype pairs: Firstly, the mutation data associated with the patients, in the form of genomic regions representing the CNVs in the patient genomes, was decomposed into small overlapping regions (SOR). Full methodology behind the construction of SORs can be found in [51]; in brief, all CNVs were aligned with the genome, and subdivided into smaller regions where they overlapped with other patients. Any region shared by at least two patients was considered a SOR.

These SORs were then used to build a tripartite network with three entities: SORs, patients and phenotypes, similar to the method employed in [25, 51]. This was then converted into a bipartite network, with one layer containing SORs and phenotype terms and the other containing patients. We then calculated association values between the phenotypes and SORs using the hypergeometric index, retaining relations with a value ≥ 2.

These Phenotype-SOR term relations were then converted to Phenotype-Gene relations by mapping the position of each SOR to the genome and, should the SOR overlap a known gene or genes, replacing the Phenotype-SOR pair with the corresponding Phenotype-Gene pair(s). Annotation was obtained from the NCBI for the human genome (Version GRCh37.p13).

### Validating Phenotype-Gene associations using PubMed co-mention

Validation of the Phenotype-Gene associations was performed by obtaining the PubMed IDs corresponding to papers for each gene for which an Phenotype-gene association was made, using Biomart [66]. These were compared to the PubMed entries mentioning the HPO phenotypes, obtained using the NCBI Entrez Programming Utilities API as described above.

### Phenotype-Gene associations using other tools

Phenolyzer, Phenomizer and Orphamizer were used to predict genes for the patients in the studied DECIPHER cohort based on their phenotypic profiles. As Phenomizer and Orphamizer could only be run via their websites, by entering patient phenotypes manually, it was used for the 50 patients with the most and least extensive phenotypic profiles (20 patient with the most extensive profiles. 10 patients with one phenotype, 10 patients with two phenotypes and 10 patients with three phenotypes). Results were presented in terms of the numbers of patients for which each tool could predict genes within the patient CNV. We used thresholds of 0.8 and 0.9 for Orphamizer and Phenolyzer, and an adjusted *P*-value threshold of 0.01 for Phenomizer.

### Obtaining Phenotype-Phenotype pairs that show consistent and emergent functional systems

These Phenotype-Gene pairs were combined to obtain gene lists for each phenotype and for each pair of phenotypes. These lists were in turn used to infer putative Phenotype-FunSys and Phenotype pair-FunSys enrichment using over-representation analysis. FunSys were defined as metabolic/signalling pathways, regulatory processes or molecular complexes; FunSys were obtained for the Biological Process sub-ontology of Gene Ontology (GO) [67, 68], Kyoto Encyclopedia of Genes and Genomes (KEGG) [69], and Reactome [70].

Enrichment was inferred using the R packages clusterProfiler (Version 3.6.0) [71] for GO and KEGG, and ReactomePA (Version 1.22.0) [72] for Reactome. In brief, these packages look for the over-representation of genes from different FunSys within a list of query genes. This was performed for the genes assigned to each phenotype in a given pair separately, and also for all genes assigned to both phenotypes in that pair. Both packages were executed with default parameters and the relations with adjusted *P*-values less than 0.05 were considered significant. If the genes for a given phenotype or phenotype-phenotype pair show over-representation for a given FunSys, it is said to be enriched.

This resulted in lists of Phenotype-FunSys and Phenotype pair-FunSys relations, which were further analysed to identify FunSys that were either consistent or emergent for a given pair. More specifically, if two phenotypes showed enrichment for the same functional system (FunSys) when considered separately, but also when considered together, taking all genes for the pair, this was considered to be showing consistent FunSys enrichment. If neither phenotype showed enrichment for a given FunSys when considered separately, but enrichment was found when considering genes for both, this was considered an emergent FunSys for the pair. This concept is shown graphically in Fig 2C.

We compared the numbers of Phenotype-FunSys and Phenotype pair-FunSys relations obtained using the real dataset to simulated datasets obtained by randomizing the gene-content of the SORs, such that each region remains connected to the same Phenotypes and contains the same total number of genes. This was repeated 100 times so that the numbers obtained with the real data could be compared to the distributions of values obtained with the randomized data.

### Phenotype cluster analysis

The Phenotype-Phenotype pairs with consistent and emergent FunSys as described above were used to build a phenotype network. This was then analysed using the R package linkcomm [73] to obtain link communities, representing clusters of phenotypes within the network that show high connectivity to each other. The package was executed with default parameters for undirected networks. These clusters were then further investigated in order to look for functionally coherent clusters, defined as those for which at least 70% of the constituent phenotypes are associated with the same FunSys, either by showing over-representation for it individually, or by forming part of a consistent or emergent pair that shows over-representation for it, as shown in Fig 2C.

HPO annotation for OMIM and Orphanet diseases was used to quantify how many of these clusters also contained phenotypes belonging the same disease, so that the clusters formed by the more-specific, less-specific and random pairs lists could be compared in terms of diseases overlap.

### Semantic similarity between cluster phenotypes

We calculated semantic similarity between the HPO terms that represent the phenotypes found within the phenotype clusters, using the HPOsim R package [74]. The Resnik method was used to calculate similarity, based on data in the HPO.db package. Results were compared to those found for random clusters, produced by randomizing cluster-phenotype assignment, but using clusters of the same size and keeping the distribution of phenotypes the same. Results were also compared to those found using the networks resulting from the less-specific pairs.

### Reports produced by workflow

The workflow produces multiple reports for the different stages of the analysis. These include the **Pairs Report** (S1 Report), which presents statistics related to the set of patients analysed, including the number of HPOs, numbers of phenotype pairs involving at least one patient, how many of these were more-specific, etc. It also gives details of the numbers of pairs overlapping with known diseases, as described above, the PubMed analysis, and details of how many of the pairs were considered consistent or emergent for the different FunSys categories.

It also produces a report describing the clustering analysis, **Clustering Report** (S2 Report), which shows output related to the clustering procedure, as well as bar-charts showing the numbers of functionally coherent clusters for each pairs list and overlap with disease.

In terms of the actual clusters, the PhenCo workflow produces three reports, **GO Cluster Details, Reactome Cluster Details and KEGG Cluster Details**, (S3, S4 and S5 Reports). Each report provides detailed information for each cluster in terms of its constituent phenotype terms, associated FunSys, and any OMIM diseases that map to the phenotypes. A cluster is said to overlap with a disease if all of its phenotypes, or all of its phenotypes except one are also found within the disease. OMIM diseases are used rather than Orphanet as these show overlap with a greater number of phenotypes. Finally, the report provides details of all patients that have at least one phenotype that overlaps with the cluster, with details of their phenotypic profiles. It also gives details of genes mapping to these patients that overlap with the GOs over-represented in the clusters. However, these details are not included here, as patient confidentiality restrictions mean we cannot present full details of the phenotypic profiles and affected genes of DECIPHER patients.

Each cluster report is ordered in terms of the clusters that show both coherent FunSys and disease overlap, followed by those showing coherent FunSys only, those showing disease overlap only and finally the clusters that show neither. These three reports are accompanied by the **Patient Report** (S6 Report), which presents, for each patient in the initial dataset, details of the clusters to which they belong, including phenotypic overlap, enrichment for each cluster in terms of FunSys (for GO, KEGG and Reactome) and details of overlap with known diseases from OMIM and Orphanet. It also gives details of any genes that can be mapped to the patient through the Inferring Phenotype-Gene associations process described above and shows which of these genes overlap with the FunSys ascribed to the patient phentoypes. Although we have included an example Patient Report as a report file, please note we have only shown details for the two patients described in the Results section, as patient confidentiality restrictions prohibit us from releasing details of all DECIPHER patient profiles and affected genes. Finally, the report **Article Figures** (S7 Report) produces the Figs 3, 4, 5 and 6 as they appear in this article.

## Supporting information

S1 Report pairs_report.html. Various statistics about the dataset (distribution of scores, distribution of HPOs, etc.), for more and less-specific pairs lists.
(HTML)

S2 Report clustering_report.html. General statistics related to the cluster analysis, in terms of the FunSys, overlap with known disease and more.
(HTML)

S3 Report cluster_details_go.html. Cluster Details for GO-term coherent clusters. General details for each of the clusters, as well as details of OMIM diseases and GO-terms. Although not shown here due to patient-confidentiality, this report can also include tables of patients assigned to each cluster, including details of their phenotypes and genes that overlap with the phenotypes in the clusters, allowing the interested user to generate such information for their own patient cohort.
(HTML)

S4 Report cluster_details_reactome.html. Cluster Details for Reactome coherent clusters. General details for each of the clusters, as well as details of OMIM diseases and Reactome pathways. Although not shown here due to patient-confidentiality, this report can also include tables of patients assigned to each cluster, including details of their phenotypes and genes that overlap with the phenotypes in the clusters, allowing the interested user to generate such information for their own patient cohort.
(HTML)

S5 Report Cluster details for KEGG coherent clusters. General details for each of the clusters, as well as details of OMIM diseases and KEGG pathways. Although not shown here due to patient-confidentiality, this report can also include tables of patients assigned to each cluster, including details of their phenotypes and genes that overlap with the phenotypes in the clusters, allowing the interested user to generate such information for their own patient cohort.
(HTML)

S6 Report General details of the patients, their phenotypes, and affected genes. Also contains details, for each patient, to which clusters their phenotypes map, and the genes that overlap functional systems enriched in these clusters. Due to patient confidentiality, we have not shown exact details of phenotypes and genes for each DECIPHER patient analysed here, however the interested reader who has signed the license agreement with DECIPHER could do so easily. We have included output generated for two patients from the DECIPHER database.
(HTML)

S7 Report A report producing the composite figures that appear in this article. (HTML)

S1 Table Comparison of real data with simulations produced by randomizing SOR gene content. Simulations were repeated 100 times and the average was obtained. S.d: standard deviation.
(PDF)

S2 Table Overlap between DECIPHER patient phenotypes and PhenCo clusters. Columns show total numbers of patients with at least 2 and 3 phenotypes in the same PhenCo cluster, for the real clusters and for clusters formed by randomizing Cluster-HPO membership, keeping the same number of HPOs per cluster and the same distribution of HPO terms.
(PDF)

S3 Table Predictions made by the different knowledge-based methods. The patient column refers to the number of patients for which the given method predicted at least one gene within the patient CNV. The gene column refers to the total numbers of genes predicted that overlap with the patient CNV. Phenolyzer and Orphamizer were used with thresholds of 0.8 and 0.9, as indicated. Phenomizer was run with adjusted p-value thresholds of 0.01 and 0.05.
(PDF)

S4 Table Properties of the networks formed by the different pairs lists. Values represent mean ± one standard deviation. Clust Coeff: Clustering Coefficient, Avg min path: Average Minimum Path, Spec: specific, l rdm and n rdm: link randomized and node randomized models respectively.
(PDF)

S1 Fig Distribution of the semantic similarity values for the randomized more-specific clusters (histogram and density curve), compared to semantic similarity obtained with the real data (vertical red broken line). (PDF)
