# Supplementary material for: Phenotype-genotype comorbidity analysis of patients with rare disorders provides insight into their pathological and molecular bases
Source: PLoS Genet. 2020 Oct 1;16(10):e1009054. doi: 10.1371/journal.pgen.1009054 (PMC7553355; doi:10.1371/journal.pgen.1009054)
Supplement: S1 Report — Various statistics about the dataset (distribution of scores, distribution of HPOs, etc.), for more and less-specific pairs lists. (HTML) [file pgen.1009054.s001.html]

pairs\_report\_template.utf8


# Report 1: Pairs Report

We want to study comorbidity relationships between phenotypes pairs from DECIPHER database.

We have built different networks:

**1. more\_spec (Comorbidity Network)**: HPO pairs with a HyI >= 2.  
**2. less\_spec**: HPO pairs with a HyI < 2.  
**4. random\_links**: randmly shufflerd the associations between HPO pairs, while keeping the number of edges and nodes unchanged.  
**5. random\_nodes**: Have de same properties and topological structure than the Hypergeometic\_phen\_reliable\_terms. But the number of edges per node change.  
**6. unconnected**: Random selection of HPO pairs that are not connected by any patient in DECIPHER database (negative control).

### Properties of the phenotype-phenotype pairs lists and random models

| Metric | Value |
| --- | --- |
| Total\_of\_patients | 20924 |
| Number\_of\_patients\_final\_cohort | 3374 |
| Number\_of\_HPO | 1758 |
| Number\_of\_phenotype\_phenotype\_pairs | 36709 |
| Number\_of\_more\_specific\_pairs | 8099 |
| Number\_of\_less\_specific\_pairs | 28610 |
| Number\_of\_unconnected\_pairs | 1507694 |

**Table A**: Properties of the phenotype-phenotype pairs lists and random models.

**Figure A**: Properties of the phenotype-phenotype pairs

### Number of genes mapping to each phenotype pair

| Net\_type | Genes mean per pair |
| --- | --- |
| less\_spec | 673.1729 |
| less\_spec\_l\_rdm | 674.6051 |
| less\_spec\_n\_rdm | 265.6002 |
| more\_spec\_l\_rdm | 393.8026 |
| more\_spec | 386.9433 |
| more\_spec\_n\_rdm | 242.9505 |
| unconnected | 229.3953 |

**Table B**: Properties of the phenotype-phenotype pairs lists and random models.

### Topological analysis

#### MEAN

| Type | Clustering.coef | Diameter | Average.minimum.path | Number.of.nodes |
| --- | --- | --- | --- | --- |
| less\_spec | 0.0545773 | 5.00 | 2.920132 | 1378.00 |
| less\_spec\_l\_rdm | 0.0318852 | 4.74 | 2.963441 | 1378.00 |
| less\_spec\_n\_rdm | 0.0545773 | 5.00 | 2.920132 | 1378.00 |
| more\_spec | 0.2774341 | 11.00 | 4.018239 | 1679.00 |
| more\_spec\_l\_rdm | 0.0088730 | 6.00 | 3.312238 | 1679.00 |
| more\_spec\_n\_rdm | 0.2774341 | 11.00 | 4.018239 | 1679.00 |
| unconnected | 0.0052201 | 6.14 | 3.612377 | 1757.82 |

#### STANDARD DEVIATION

| Type | Clustering.coef | Diameter | Average.minimum.path | Number.of.nodes |
| --- | --- | --- | --- | --- |
| less\_spec | 0.0011708 | 0.0000000 | 0.0090939 | 10.8834268 |
| less\_spec\_l\_rdm | 0.0009284 | 0.4430875 | 0.0158192 | 10.8834268 |
| less\_spec\_n\_rdm | 0.0011708 | 0.0000000 | 0.0090939 | 10.8834268 |
| more\_spec | NA | NA | NA | NA |
| more\_spec\_l\_rdm | 0.0005171 | 0.0000000 | 0.0039248 | 0.0000000 |
| more\_spec\_n\_rdm | 0.0000000 | 0.0000000 | 0.0000000 | 0.0000000 |
| unconnected | 0.0003815 | 0.3505098 | 0.0017224 | 0.4375255 |

**Table C**: Topological Analysis results from the different Pairs Lists.

### Overlap of the pairs lists with known diseases

**Figure B**: Number of phenotype pairs that appear together in OMIM (left) and Orphanet (right) diseases.

| Type | Confirmed pairs OMIM | Confirmed pairs Orphanet | Union diseases | Intersection diseases |
| --- | --- | --- | --- | --- |
| less\_spec | 2343.90 | 1082.44 | 2500.60 | 925.74 |
| less\_spec\_l\_rdm | 1911.00 | 804.08 | 1962.70 | 680.82 |
| less\_spec\_n\_rdm | 244.82 | 90.78 | 271.48 | 64.12 |
| more\_spec | 1259.00 | 764.00 | 1332.00 | 691.00 |
| more\_spec\_l\_rdm | 527.20 | 222.32 | 575.90 | 169.18 |
| more\_spec\_n\_rdm | 176.26 | 66.50 | 196.86 | 45.90 |
| unconnected | 119.30 | 37.00 | 135.54 | 20.76 |

**Table D**: Overlap with diseases summary.

## Comention analysis.

**Figure C**: Comention pvalue density plot.

## PMID per pairs list.

| Net\_type | Union PMID mean | Union PMID sd | Union PMID median | Union PMID IQR |
| --- | --- | --- | --- | --- |
| less\_spec | 28825.93 | 42356.68 | 11364 | 31618.00 |
| less\_spec\_l\_rdm | 28839.30 | 41847.91 | 11818 | 31222.75 |
| more\_spec\_l\_rdm | 16716.29 | 32696.83 | 3446 | 12881.00 |
| more\_spec | 16665.94 | 33562.09 | 3089 | 12408.00 |

**Table E**: Union PMID metrics per network type.

### Emergent and consistent functional systems

#### KEGG

**Figure D**: KEGG enrichment analysis. **Top**: Number of pairs with at least one consistent or emergent pathways. **Bottom left**: Number of consistent/emergent pathways. **Bottom right**: Number of pairs with consistent/emergent pathways.

#### Mean

| Type | HPO pairs with at least 1 term KEGG | Consistent enriched terms KEGG | Emergent enriched terms KEGG | Pairs with consistent terms KEGG | Pairs with emergent terms KEGG |
| --- | --- | --- | --- | --- | --- |
| less\_spec | 383.94 | 107.86 | 541.16 | 54.06 | 340.26 |
| less\_spec\_l\_rdm | 345.02 | 81.20 | 500.92 | 42.68 | 308.98 |
| less\_spec\_n\_rdm | 87.72 | 20.64 | 117.16 | 11.18 | 78.18 |
| more\_spec | 273.00 | 119.00 | 343.00 | 61.00 | 218.00 |
| more\_spec\_l\_rdm | 140.12 | 40.12 | 192.18 | 19.20 | 124.08 |
| more\_spec\_n\_rdm | 73.62 | 18.70 | 97.78 | 9.98 | 65.08 |
| unconnected | 61.04 | 12.90 | 79.34 | 7.44 | 54.72 |

#### SD

| Type | HPO pairs with at least 1 term KEGG | Consistent enriched terms KEGG | Emergent enriched terms KEGG | Pairs with consistent terms KEGG | Pairs with emergent terms KEGG |
| --- | --- | --- | --- | --- | --- |
| less\_spec | 15.534570 | 22.393521 | 43.93518 | 5.433870 | 15.295471 |
| less\_spec\_l\_rdm | 13.796613 | 20.430070 | 41.64174 | 6.935387 | 13.436912 |
| less\_spec\_n\_rdm | 19.871054 | 11.960411 | 39.39562 | 4.378472 | 18.928102 |
| more\_spec | NA | NA | NA | NA | NA |
| more\_spec\_l\_rdm | 11.731068 | 14.046047 | 21.53658 | 3.886134 | 10.520805 |
| more\_spec\_n\_rdm | 8.810685 | 8.172253 | 17.55608 | 3.178307 | 8.052988 |
| unconnected | 7.157014 | 8.352563 | 13.44469 | 2.186788 | 6.872127 |

**Table F**: Summary of KEGG functional analysis

#### Reactome

**Figure E**: Reactome enrichment analysis. **Top**: Number of pairs with at least one consistent or emergent pathways. **Bottom left**: Number of consistent/emergent pathways. **Bottom right**: Number of pairs with consistent/emergent pathways.

#### Mean

| Type | HPO pairs with at least 1 term Reactome | Consistent enriched terms Reactome | Emergent enriched terms Reactome | Pairs with consistent terms Reactome | Pairs with emergent terms Reactome |
| --- | --- | --- | --- | --- | --- |
| less\_spec | 630.04 | 189.14 | 1189.70 | 102.84 | 547.92 |
| less\_spec\_l\_rdm | 551.18 | 133.92 | 1045.06 | 76.76 | 489.92 |
| less\_spec\_n\_rdm | 137.24 | 37.58 | 292.28 | 17.32 | 123.06 |
| more\_spec | 367.00 | 255.00 | 669.00 | 71.00 | 306.00 |
| more\_spec\_l\_rdm | 222.76 | 53.80 | 431.06 | 28.48 | 200.56 |
| more\_spec\_n\_rdm | 108.36 | 34.08 | 243.24 | 15.88 | 95.44 |
| unconnected | 93.14 | 24.88 | 182.00 | 12.52 | 83.02 |

#### SD

| Type | HPO pairs with at least 1 term Reactome | Consistent enriched terms Reactome | Emergent enriched terms Reactome | Pairs with consistent terms Reactome | Pairs with emergent terms Reactome |
| --- | --- | --- | --- | --- | --- |
| less\_spec | 24.346110 | 17.00301 | 89.92951 | 7.717883 | 22.123808 |
| less\_spec\_l\_rdm | 19.289090 | 16.66334 | 72.06500 | 7.471196 | 20.374894 |
| less\_spec\_n\_rdm | 24.212967 | 18.72910 | 106.03754 | 5.452728 | 22.610644 |
| more\_spec | NA | NA | NA | NA | NA |
| more\_spec\_l\_rdm | 13.930674 | 14.76897 | 62.82577 | 4.887468 | 14.078729 |
| more\_spec\_n\_rdm | 13.290721 | 20.10669 | 72.81107 | 4.749393 | 12.597635 |
| unconnected | 9.220673 | 15.92539 | 43.72129 | 3.575655 | 8.732978 |

**Table G**: Summary of Reactome functional analysis

#### GO

**Figure F**: GO enrichment analysis. **Top**: Number of pairs with at least one consistent or emergent pathways. **Bottom left**: Number of consistent/emergent pathways. **Bottom right**: Number of pairs with consistent/emergent pathways.

#### Mean

| Type | HPO pairs with at least 1 term GO | Consistent enriched terms GO | Emergent enriched terms GO | Pairs with consistent terms GO | Pairs with emergent terms GO |
| --- | --- | --- | --- | --- | --- |
| less\_spec | 1246.36 | 1357.78 | 4110.08 | 123.94 | 1184.52 |
| less\_spec\_l\_rdm | 1100.52 | 1013.92 | 3670.72 | 95.08 | 1053.12 |
| less\_spec\_n\_rdm | 268.32 | 182.24 | 857.32 | 19.24 | 257.84 |
| more\_spec | 705.00 | 1564.00 | 2370.00 | 107.00 | 639.00 |
| more\_spec\_l\_rdm | 437.82 | 404.22 | 1476.02 | 38.14 | 416.84 |
| more\_spec\_n\_rdm | 216.20 | 136.74 | 698.44 | 17.22 | 206.12 |
| unconnected | 186.86 | 99.38 | 608.14 | 13.62 | 179.04 |

#### SD

| Type | HPO pairs with at least 1 term GO | Consistent enriched terms GO | Emergent enriched terms GO | Pairs with consistent terms GO | Pairs with emergent terms GO |
| --- | --- | --- | --- | --- | --- |
| less\_spec | 23.47535 | 166.24647 | 148.08035 | 8.997528 | 23.980638 |
| less\_spec\_l\_rdm | 30.28453 | 175.85045 | 152.96325 | 9.360883 | 30.646530 |
| less\_spec\_n\_rdm | 43.64628 | 101.02850 | 168.81187 | 5.860835 | 42.233733 |
| more\_spec | NA | NA | NA | NA | NA |
| more\_spec\_l\_rdm | 18.51937 | 111.06301 | 116.48763 | 5.150827 | 18.639830 |
| more\_spec\_n\_rdm | 20.47996 | 63.14861 | 106.19930 | 3.604362 | 19.755278 |
| unconnected | 10.76087 | 62.49535 | 77.50023 | 3.719173 | 9.889099 |

**Table H**: Summary of GO functional analysis
