# Supplementary material for: Phenotype-genotype comorbidity analysis of patients with rare disorders provides insight into their pathological and molecular bases
Source: PLoS Genet. 2020 Oct 1;16(10):e1009054. doi: 10.1371/journal.pgen.1009054 (PMC7553355; doi:10.1371/journal.pgen.1009054)
Supplement: S2 Report — General statistics related to the cluster analysis, in terms of the FunSys, overlap with known disease and more. (HTML) [file pgen.1009054.s002.html]

clustering\_report\_template.utf8


# Report 2: Clustering Report

To do this clustering analysis, we select those HPO pairs which have consistent or emergent pathways in KEGG, Reactome or GO enrichment. Once we have done this selection, we performed a cluster analysis with these HPO pairs, thanks to the R package Linkcomm.

# KEGG enrichment

### KEGG cluster analysis summary

**Image 1: Summary dendograme of clustering**

**Image 2: Top connected nodes**

**Image 3: Relationships between communities**

### KEGG: Number of clusters

**Figure A**: (**Top**) ***Clusters size average for KEGG analysis***. (**Bottom**) ***Number of clusters for KEGG analysis***. The average size of clusters is very similar in all networks. However, the number of clusters varys. However, the number of clusters varies between real and random networks, being this greater in real networks.

### KEGG: Number of clusters which fits with an OMIM diseases

**Figure B**: (**Top-Left**) ***Number of different OMIM diseases that have a perfect match with clusters***. (**Top-Right**) ***Number of different OMIM diseases that match with clusters except from one phenotype***. (**Botton-Left**) ***Number of clusters that have a perfect match with OMIM diseases***. (**Botton-Left**) ***Number of clusters that match with OMIM diseases except from one phenotype***. High-confidence clusters have more clusters that match with OMIM diseases and those clusters coincide with more than one disease comparing with Low-confidece and with the it random models. The number of clusters and diseases increase when we allow a cluster match failure with respect to the disease.

### KEGG: Number of clusters which fits with an Orphanet diseases

**Figure C**: (**Top-Left**) ***Number of different Orphanet diseases that have a perfect match with clusters***. (**Top-Right**) ***Number of different Orphanet diseases that match with clusters except from one phenotype***. (**Botton-Left**) ***Number of clusters that have a perfect match with Orphanet diseases***. (**Botton-Left**) ***Number of clusters that match with Orphanet diseases except from one phenotype***.

### KEGG: Number of clusters which at least 70% of HPOs share one or more KEGG pathway

**Figure D**: ***Number of clusters in which all their HPOs shared the same KEGG pathway***. The number of clusters in which all its HPO are enriched in the same funtional system is higher in real networks comparing to random models.

### KEGG: Enriched clusters 70% of HPO coincidence with OMIM diseases

**Figure E**: For those clusters which have common KEGG enrichment: (**Top-Left**) ***Number of different OMIM diseases that have a perfect match with clusters***. (**Top-Right**) ***Number of different OMIM diseases that match with clusters except from one phenotype***. (**Botton-Left**) ***Number of clusters that have a perfect match with OMIM diseases***. (**Botton-Left**) ***Number of clusters that match with OMIM diseases except from one phenotype***. High-confidence clusters have more clusters that match with OMIM diseases and those clusters coincide with more than one disease comparing with Low-confidece and with the it random models. The number of clusters and diseases increase when we allow a cluster match failure with respect to the disease.

### KEGG: Enriched clusters 70% of HPO coincidence with Orphanet diseases

**Figure F**: For those clusters which have common KEGG enrichment: (**Top-Left**) ***Number of different Orphanet diseases that have a perfect match with clusters***. (**Top-Right**) ***Number of different Orphanet diseases that match with clusters except from one phenotype***. (**Botton-Left**) ***Number of clusters that have a perfect match with Orphanet diseases***. (**Botton-Left**) ***Number of clusters that match with Orphanet diseases except from one phenotype***.

### Summary table of KEGG cluster analysis 70% HPO

| Type | Clusters number | Average cluster size | Cluster OMIM overlap | Cluster Orphanet overlap | Clusters functional coherent KEGG | Clusters functional coherent KEGG-OMIM overlap | Clusters functional coherent KEGG-Orphanet overlap |
| --- | --- | --- | --- | --- | --- | --- | --- |
| less\_spec | 34.62 | 6.052973 | 8.04 | 5.04 | 17.34 | 5.88 | 3.80 |
| less\_spec\_l\_rdm | 28.40 | 8.458233 | 4.78 | 3.38 | 13.36 | 2.98 | 2.26 |
| less\_spec\_n\_rdm | 5.00 | 4.999375 | 0.42 | 0.34 | 2.24 | 0.36 | 0.28 |
| more\_spec | 33.00 | 4.909091 | 14.00 | 13.00 | 23.00 | 10.00 | 10.00 |
| more\_spec\_l\_rdm | 6.22 | 4.493918 | 0.42 | 0.20 | 2.04 | 0.22 | 0.12 |
| more\_spec\_n\_rdm | 2.12 | 2.519333 | 0.26 | 0.20 | 1.38 | 0.20 | 0.16 |
| unconnected | 0.02 | 0.060000 | 0.00 | 0.00 | 0.02 | 0.00 | 0.00 |

**Table A**: Summary table of KEGG cluster analysis 70% HPO

## Reactome enrichment

## Reactome cluster analysis summary

**Image 4: Summary dendograme of clustering**

**Image 5: Top connected nodes**

**Image 6: Relationships between communities**

### Reactome: Number of clusters and size

**Figure G**: (**Top**) ***Clusters size average for Reactome analysis***. (**Bottom**) ***Number of clusters for Reactome analysis***. The average size of clusters is very similar in all networks. However, the number of clusters varys. However, the number of clusters varies between real and random networks, being this greater in real networks.

### Reactome: Number of clusters which fits with an OMIM diseases

**Figure H**: (**Top-Left**) ***Number of different OMIM diseases that have a perfect match with clusters***. (**Top-Right**) ***Number of different OMIM diseases that match with clusters except from one phenotype***. (**Botton-Left**) ***Number of clusters that have a perfect match with OMIM diseases***. (**Botton-Left**) ***Number of clusters that match with OMIM diseases except from one phenotype***. High-confidence clusters have more clusters that match with OMIM diseases and those clusters coincide with more than one disease comparing with Low-confidece and with the it random models. The number of clusters and diseases increase when we allow a cluster match failure with respect to the disease.

### Reactome: Number of clusters which fits with an Orphanet diseases

**Figure I**: (**Top-Left**) ***Number of different Orphanet diseases that have a perfect match with clusters***. (**Top-Right**) ***Number of different Orphanet diseases that match with clusters except from one phenotype***. (**Botton-Left**) ***Number of clusters that have a perfect match with Orphanet diseases***. (**Botton-Left**) ***Number of clusters that match with Orphanet diseases except from one phenotype***.

### Reactome: Number of clusters which at least 70% of HPOs share one or more Reactome pathway

**Figure J**: ***Number of clusters in which all their HPOs shared the same Reactome pathway***. The number of clusters in which all its HPO are enriched in the same funtional system is higher in real networks comparing to random models.

### Reactome: Enriched clusters 70% of HPO coincidence with OMIM diseases

**Figure K**: For those clusters which have common Reactome enrichment: (**Top-Left**) ***Number of different OMIM diseases that have a perfect match with clusters***. (**Top-Right**) ***Number of different OMIM diseases that match with clusters except from one phenotype***. (**Botton-Left**) ***Number of clusters that have a perfect match with OMIM diseases***. (**Botton-Left**) ***Number of clusters that match with OMIM diseases except from one phenotype***. High-confidence clusters have more clusters that match with OMIM diseases and those clusters coincide with more than one disease comparing with Low-confidece and with the it random models. The number of clusters and diseases increase when we allow a cluster match failure with respect to the disease.

### Reactome: Enriched clusters 70% of HPO coincidence with Orphanet diseases

**Figure L**: For those clusters which have common Reactome enrichment: (**Top-Left**) ***Number of different Orphanet diseases that have a perfect match with clusters***. (**Top-Right**) ***Number of different Orphanet diseases that match with clusters except from one phenotype***. (**Botton-Left**) ***Number of clusters that have a perfect match with Orphanet diseases***. (**Botton-Left**) ***Number of clusters that match with Orphanet diseases except from one phenotype***.

### Summary table of Reactome cluster analysis 70% HPO

| Type | Clusters number | Average cluster size | Cluster OMIM overlap | Cluster Orphanet overlap | Clusters functional coherent Reactome | Clusters functional coherent Reactome-OMIM overlap | Clusters functional coherent Reactome-Orphanet overlap |
| --- | --- | --- | --- | --- | --- | --- | --- |
| less\_spec | 77.20 | 6.420569 | 18.44 | 12.22 | 29.42 | 12.26 | 8.26 |
| less\_spec\_l\_rdm | 68.84 | 5.921636 | 11.96 | 8.52 | 23.68 | 6.98 | 5.06 |
| less\_spec\_n\_rdm | 12.00 | 8.495958 | 0.88 | 0.54 | 3.44 | 0.56 | 0.36 |
| more\_spec | 31.00 | 4.064516 | 20.00 | 16.00 | 19.00 | 14.00 | 10.00 |
| more\_spec\_l\_rdm | 15.70 | 7.962835 | 1.26 | 0.64 | 4.04 | 0.72 | 0.36 |
| more\_spec\_n\_rdm | 4.84 | 3.610621 | 1.32 | 0.66 | 2.34 | 0.74 | 0.34 |
| unconnected | 0.04 | 0.120000 | 0.00 | 0.04 | 0.02 | 0.00 | 0.02 |

**Table B**: Summary table of Reactome cluster analysis 70% HPO

## GO enrichment

## GO cluster analysis summary

**Image 7: Summary dendograme of clustering**

**Image 8: Top connected nodes**

**Image 9: Relationships between communities**

### GO: Number of clusters and size

**Figure M**: (**Top**) ***Clusters size average for GO analysis***. (**Bottom**) ***Number of clusters for GO analysis***. The average size of clusters is very similar in all networks. However, the number of clusters varys. However, the number of clusters varies between real and random networks, being this greater in real networks.

### GO: Number of clusters which fits with an OMIM diseases

**Figure N**: (**Top-Left**) ***Number of different OMIM diseases that have a perfect match with clusters***. (**Top-Right**) ***Number of different OMIM diseases that match with clusters except from one phenotype***. (**Botton-Left**) ***Number of clusters that have a perfect match with OMIM diseases***. (**Botton-Left**) ***Number of clusters that match with OMIM diseases except from one phenotype***. High-confidence clusters have more clusters that match with OMIM diseases and those clusters coincide with more than one disease comparing with Low-confidece and with the it random models. The number of clusters and diseases increase when we allow a cluster match failure with respect to the disease.

### GO: Number of Clusters which fits with an Orphanet diseases

**Figure O**: (**Top-Left**) ***Number of different Orphanet diseases that have a perfect match with clusters***. (**Top-Right**) ***Number of different Orphanet diseases that match with clusters except from one phenotype***. (**Botton-Left**) ***Number of clusters that have a perfect match with Orphanet diseases***. (**Botton-Left**) ***Number of clusters that match with Orphanet diseases except from one phenotype***.

### GO: Number of clusters which at least 70% of HPOs share one or more GO pathway

**Figure P**: ***Number of clusters in which all their HPOs shared the same KEGG pathway***. The number of clusters in which all its HPO are enriched in the same funtional system is higher in real networks comparing to random models.

### GO: Enriched clusters 70% of HPO coincidence with OMIM diseases

**Figure Q**: For those clusters which have common GO enrichment: (**Top-Left**) ***Number of different OMIM diseases that have a perfect match with clusters***. (**Top-Right**) ***Number of different OMIM diseases that match with clusters except from one phenotype***. (**Botton-Left**) ***Number of clusters that have a perfect match with OMIM diseases***. (**Botton-Left**) ***Number of clusters that match with OMIM diseases except from one phenotype***. High-confidence clusters have more clusters that match with OMIM diseases and those clusters coincide with more than one disease comparing with Low-confidece and with the it random models. The number of clusters and diseases increase when we allow a cluster match failure with respect to the disease.

### GO: Enriched clusters 70% of HPO coincidence with Orphanet diseases

**Figure R**: For those clusters which have common GO enrichment: (**Top-Left**) ***Number of different Orphanet diseases that have a perfect match with clusters***. (**Top-Right**) ***Number of different Orphanet diseases that match with clusters except from one phenotype***. (**Botton-Left**) ***Number of clusters that have a perfect match with Orphanet diseases***. (**Botton-Left**) ***Number of clusters that match with Orphanet diseases except from one phenotype***.

### Summary table of GO cluster analysis 70% HPO

| Type | Clusters number | Average cluster size | Cluster OMIM overlap | Cluster Orphanet overlap | Clusters functional coherent GO | Clusters functional coherent GO-OMIM overlap | Clusters functional coherent GO-Orphanet overlap |
| --- | --- | --- | --- | --- | --- | --- | --- |
| less\_spec | 152.16 | 7.056145 | 40.22 | 23.26 | 42.50 | 19.04 | 11.68 |
| less\_spec\_l\_rdm | 124.32 | 7.682959 | 23.00 | 14.80 | 30.20 | 10.50 | 6.92 |
| less\_spec\_n\_rdm | 27.18 | 7.628625 | 2.34 | 1.30 | 6.26 | 1.00 | 0.58 |
| more\_spec | 88.00 | 4.738636 | 45.00 | 32.00 | 45.00 | 26.00 | 17.00 |
| more\_spec\_l\_rdm | 28.90 | 11.970762 | 2.82 | 1.90 | 8.00 | 1.12 | 0.78 |
| more\_spec\_n\_rdm | 21.78 | 4.025864 | 4.14 | 2.52 | 8.60 | 2.00 | 1.08 |
| unconnected | 6.68 | 2.007494 | 0.02 | 0.08 | 1.04 | 0.00 | 0.02 |

**Table C**: Summary table of GO cluster analysis 70% HPO
