# Supplementary material for: Phenotype-genotype comorbidity analysis of patients with rare disorders provides insight into their pathological and molecular bases
Source: PLoS Genet. 2020 Oct 1;16(10):e1009054. doi: 10.1371/journal.pgen.1009054 (PMC7553355; doi:10.1371/journal.pgen.1009054)
Supplement: S3 Report — Cluster Details for GO-term coherent clusters. General details for each of the clusters, as well as details of OMIM diseases and GO-terms. Although not shown here due to patient-confidentiality, this report can also include tables of patients assigned to each cluster, including details of their phenotypes and genes that overlap with the phenotypes in the clusters, allowing the interested user to generate such information for their own patient cohort. (HTML) [file pgen.1009054.s003.html]

cluster\_details\_go\_template.utf8.md


# Report 3: Cluster Details for GO-term coherent clusters

## GO clusters

---


---


---

# Cluster 1

| Cluster | Term | Name |
| --- | --- | --- |
| 1 | HP:0010485 | Hyperextensibility at elbow |
| 1 | HP:0010500 | Hyperextensibility of the knee |
| 1 | HP:0002751 | Kyphoscoliosis |

| Cluster | Term | Name | Genes | Percentage\_of\_nodes\_with\_funsys |
| --- | --- | --- | --- | --- |
| 1 | GO:0038003 | opioid receptor signaling pathway | OPRK1, OPRD1, OPRL1, OGFR, OPRM1, NPBWR2, SYP | 100 |

| Cluster | Term | Name | HPOs\_in\_clusters |
| --- | --- | --- | --- |
| 1 | OMIM:130000 | EHLERS-DANLOS SYNDROME, CLASSIC TYPE, 1; EDSCL1 | HP:0010485, HP:0010500 |

---


---


---

# Cluster 7

| Cluster | Term | Name |
| --- | --- | --- |
| 7 | HP:0001629 | Ventricular septal defect |
| 7 | HP:0001642 | Pulmonic stenosis |
| 7 | HP:0000175 | Cleft palate |

| Cluster | Term | Name | Genes | Percentage\_of\_nodes\_with\_funsys |
| --- | --- | --- | --- | --- |
| 7 | GO:0016338 | calcium-independent cell-cell adhesion via plasma membrane cell-adhesion molecules | CLDN5, CLDN15, CLDN12, CLDN9, CLDN6, CLDN18, CLDN7, CLDN2, CLDN23, CLDN3, CLDN4, CLDN1, CLDN16, CLDN11 | 100 |

| Cluster | Term | Name | HPOs\_in\_clusters |
| --- | --- | --- | --- |
| 7 | OMIM:100300 | ADAMS-OLIVER SYNDROME 1; AOS1 | HP:0001629, HP:0000175, HP:0001642 |
| 7 | OMIM:214800 | CHARGE SYNDROME | HP:0001629, HP:0000175, HP:0001642 |
| 7 | OMIM:220210 | RITSCHER-SCHINZEL SYNDROME 1; RTSC1 | HP:0001629, HP:0000175, HP:0001642 |
| 7 | OMIM:261540 | PETERS-PLUS SYNDROME; PTRPLS | HP:0001629, HP:0000175, HP:0001642 |
| 7 | OMIM:312870 | SIMPSON-GOLABI-BEHMEL SYNDROME, TYPE 1; SGBS1 | HP:0001629, HP:0000175, HP:0001642 |
| 7 | OMIM:101200 | APERT SYNDROME | HP:0001629, HP:0000175 |
| 7 | OMIM:105650 | DIAMOND-BLACKFAN ANEMIA 1; DBA1 | HP:0001629, HP:0000175 |
| 7 | OMIM:106260 | ANKYLOBLEPHARON-ECTODERMAL DEFECTS-CLEFT LIP/PALATE; AEC | HP:0001629, HP:0000175 |
| 7 | OMIM:122470 | CORNELIA DE LANGE SYNDROME 1; CDLS1 | HP:0001629, HP:0000175 |
| 7 | OMIM:135900 | COFFIN-SIRIS SYNDROME 1; CSS1 | HP:0001629, HP:0000175 |
| 7 | OMIM:143095 | SPONDYLOEPIPHYSEAL DYSPLASIA WITH CONGENITAL JOINT DISLOCATIONS; SEDCJD | HP:0001629, HP:0001642 |
| 7 | OMIM:145410 | OPITZ GBBB SYNDROME, TYPE II; GBBB2 | HP:0001629, HP:0000175 |
| 7 | OMIM:147920 | KABUKI SYNDROME 1; KABUK1 | HP:0001629, HP:0000175 |
| 7 | OMIM:150250 | LARSEN SYNDROME; LRS | HP:0001629, HP:0000175 |
| 7 | OMIM:163950 | NOONAN SYNDROME 1; NS1 | HP:0001629, HP:0001642 |
| 7 | OMIM:188400 | DIGEORGE SYNDROME; DGS | HP:0001629, HP:0000175 |
| 7 | OMIM:194190 | WOLF-HIRSCHHORN SYNDROME; WHS | HP:0001629, HP:0000175 |
| 7 | OMIM:201000 | CARPENTER SYNDROME 1; CRPT1 | HP:0001629, HP:0001642 |
| 7 | OMIM:208530 | RIGHT ATRIAL ISOMERISM; RAI | HP:0001629, HP:0001642 |
| 7 | OMIM:214300 | KLIPPEL-FEIL SYNDROME 2, AUTOSOMAL RECESSIVE; KFS2 | HP:0001629, HP:0000175 |
| 7 | OMIM:218040 | COSTELLO SYNDROME; CSTLO | HP:0001629, HP:0001642 |
| 7 | OMIM:235730 | MOWAT-WILSON SYNDROME; MOWS | HP:0001629, HP:0001642 |
| 7 | OMIM:236680 | HYDROLETHALUS SYNDROME 1; HLS1 | HP:0001629, HP:0000175 |
| 7 | OMIM:245150 | KEUTEL SYNDROME; KTLS | HP:0001629, HP:0001642 |
| 7 | OMIM:256520 | NEU-LAXOVA SYNDROME 1; NLS1 | HP:0001629, HP:0000175 |
| 7 | OMIM:257920 | 3MC SYNDROME 1; 3MC1 | HP:0001629, HP:0000175 |
| 7 | OMIM:268300 | ROBERTS SYNDROME; RBS | HP:0001629, HP:0000175 |
| 7 | OMIM:270400 | SMITH-LEMLI-OPITZ SYNDROME; SLOS | HP:0001629, HP:0000175 |
| 7 | OMIM:280000 | COLOBOMA, CONGENITAL HEART DISEASE, ICHTHYOSIFORM DERMATOSIS, MENTAL RETARDATION, AND EAR ANOMALIES SYNDROME; CHIME | HP:0001629, HP:0000175 |
| 7 | OMIM:300166 | MICROPHTHALMIA, SYNDROMIC 2; MCOPS2 | HP:0001629, HP:0001642 |
| 7 | OMIM:300373 | OSTEOPATHIA STRIATA WITH CRANIAL SCLEROSIS; OSCS | HP:0001629, HP:0000175 |
| 7 | OMIM:306955 | HETEROTAXY, VISCERAL, 1, X-LINKED; HTX1 | HP:0001629, HP:0001642 |
| 7 | OMIM:309500 | RENPENNING SYNDROME 1; RENS1 | HP:0001629, HP:0000175 |
| 7 | OMIM:600001 | HEART DEFECTS, CONGENITAL, AND OTHER CONGENITAL ANOMALIES; HDCA | HP:0001629, HP:0001642 |
| 7 | OMIM:600987 | CLEFT PALATE, CARDIAC DEFECTS, AND MENTAL RETARDATION; CPCMR | HP:0001629, HP:0000175 |
| 7 | OMIM:601186 | MICROPHTHALMIA, SYNDROMIC 9; MCOPS9 | HP:0001629, HP:0001642 |
| 7 | OMIM:605039 | BOHRING-OPITZ SYNDROME; BOPS | HP:0001629, HP:0000175 |
| 7 | OMIM:607721 | NOONAN SYNDROME-LIKE DISORDER WITH LOOSE ANAGEN HAIR 1; NSLH1 | HP:0001629, HP:0001642 |
| 7 | OMIM:608328 | WEILL-MARCHESANI SYNDROME 2; WMS2 | HP:0001629, HP:0001642 |
| 7 | OMIM:608572 | BURN-MCKEOWN SYNDROME; BMKS | HP:0001629, HP:0000175 |
| 7 | OMIM:609942 | NOONAN SYNDROME 3; NS3 | HP:0001629, HP:0001642 |
| 7 | OMIM:610443 | KOOLEN-DE VRIES SYNDROME; KDVS | HP:0001629, HP:0001642 |
| 7 | OMIM:610733 | NOONAN SYNDROME 4; NS4 | HP:0001629, HP:0001642 |
| 7 | OMIM:611812 | 46,XX SEX REVERSAL WITH DYSGENESIS OF KIDNEYS, ADRENALS, AND LUNGS; SERKAL | HP:0001629, HP:0000175 |
| 7 | OMIM:612541 | NEUTROPENIA, SEVERE CONGENITAL, 4, AUTOSOMAL RECESSIVE; SCN4 | HP:0000175, HP:0001642 |
| 7 | OMIM:612561 | DIAMOND-BLACKFAN ANEMIA 6; DBA6 | HP:0001629, HP:0000175 |
| 7 | OMIM:612562 | DIAMOND-BLACKFAN ANEMIA 7; DBA7 | HP:0001629, HP:0000175 |
| 7 | OMIM:612938 | GROWTH RETARDATION, DEVELOPMENTAL DELAY, AND FACIAL DYSMORPHISM; GDFD | HP:0001629, HP:0000175 |
| 7 | OMIM:613854 | CONGENITAL HEART DEFECTS, MULTIPLE TYPES, 6; CHTD6 | HP:0001629, HP:0001642 |
| 7 | OMIM:614261 | MICROCEPHALY-CAPILLARY MALFORMATION SYNDROME; MICCAP | HP:0001629, HP:0000175 |
| 7 | OMIM:614262 | ARTHROGRYPOSIS, PERTHES DISEASE, AND UPWARD GAZE PALSY; APUG | HP:0001629, HP:0001642 |
| 7 | OMIM:615355 | NOONAN SYNDROME 8; NS8 | HP:0001629, HP:0001642 |
| 7 | OMIM:616145 | CATEL-MANZKE SYNDROME; CATMANS | HP:0001629, HP:0000175 |
| 7 | OMIM:616894 | ROBINOW SYNDROME, AUTOSOMAL DOMINANT 3; DRS3 | HP:0001629, HP:0000175 |
| 7 | OMIM:616897 | OSTEOCHONDRODYSPLASIA, COMPLEX LETHAL, SYMOENS-BARNES-GISTELINCK TYPE; OCLSBG | HP:0001629, HP:0000175 |
| 7 | OMIM:617063 | MEIER-GORLIN SYNDROME 7; MGORS7 | HP:0001629, HP:0000175 |
| 7 | OMIM:617164 | SHORT STATURE, RHIZOMELIC, WITH MICROCEPHALY, MICROGNATHIA, AND DEVELOPMENTAL DELAY; SRMMD | HP:0001629, HP:0000175 |
| 7 | OMIM:617506 | NOONAN SYNDROME-LIKE DISORDER WITH LOOSE ANAGEN HAIR 2; NSLH2 | HP:0001629, HP:0001642 |
| 7 | OMIM:618223 | VERTEBRAL ANOMALIES AND VARIABLE ENDOCRINE AND T-CELL DYSFUNCTION; VETD | HP:0000175, HP:0001642 |

---


---


---

# Cluster 12

| Cluster | Term | Name |
| --- | --- | --- |
| 12 | HP:0001792 | Small nail |
| 12 | HP:0006610 | Wide intermamillary distance |
| 12 | HP:0000430 | Underdeveloped nasal alae |

| Cluster | Term | Name | Genes | Percentage\_of\_nodes\_with\_funsys |
| --- | --- | --- | --- | --- |
| 12 | GO:0031424 | keratinization | KRTAP19-6, KRT5, KRT84, KRTAP10-5, KRT6C, KRT6A, CASP14, KRTAP8-1, CYP26B1, KRTAP10-2, KRTAP21-1, KRT78, PRSS8, KRT76, KRTAP20-2, KRTAP10-4, KRTAP22-1, KRTAP19-4, KRTAP10-8, KRTAP10-7, KRT1, PKP1, KRT4, KRTAP20-1, KRTAP10-1, KRTAP10-3, KRTAP10-10, KRT85, KRT86, KRTAP12-4, KRTAP6-2, KRTAP10-12, KRTAP19-5, KRT74, KRTAP10-11, KRTAP19-3, KRT7, KRTAP21-3, KRT81, KRTAP12-3, PKP4, KRT80, CSTA, KRTAP10-6, KRT79, KRTAP19-2, KRT2, KRT77, PPL, KRT82, KRTAP6-1, KAZN, KRTAP10-9, KRTAP19-8, KRT72, KRT71, KRTAP12-1, KRT73, KRTAP21-2, KRT3, KRT18, KRT6B, KRT83, SHARPIN, KRTAP11-1, KRT8, KRTAP19-7, KRTAP12-2, KRTAP6-3, KRT75, KRTAP19-1 | 100 |
| 12 | GO:0070268 | cornification | KRT5, KRT84, KRT6C, SPRR2D, SPRR2E, SPRR2B, CASP14, CYP26B1, CELA2A, KLK13, KRT78, KRT76, SPRR1B, KLK14, KRT1, PKP1, KRT4, KRT85, KRT86, KRT74, SPRR2F, KRT7, KLK12, TMEM79, KRT81, KRT80, CSTA, KRT79, KRT77, KRT2, PPL, KRT82, SPRR2G, KAZN, TCHH, SPRR2A, KLK5, KRT72, KRT71, KRT73, KRT3, KRT18, KRT6B, KRT83, KRT8, KRT6A, KRT75, LOR | 100 |
| 12 | GO:0045103 | intermediate filament-based process | ATP8A2, KRT6C, NEFL, KRT71, FAM83H, VIM, DNAJB6, NEFM, KRT18, KRT3, PLEC, KRT74, RAF1, KRT2, PPL, KRT6A, PKP1, EPPK1 | 100 |
| 12 | GO:0009952 | anteriorposterior pattern specification | HOXC5, HOXA2, SHH, ETS2, HIPK2, NEUROG1, HES7, TDRD5, TBXT, HOXC11, PBX3, PRKDC, PLXNA2, HNF1B, DCANP1, SKI, PGAP1, MSX1, HOXA4, HOXA9, CFC1, HES5, TIFAB, RARG, GRSF1, SIX2, TBX3, HOXC9, HOXC8, FOXF1, HOXA11, GLI3, WNT2, DMRT2, GDF11, HOXA5, HOXA3, RIPPLY2, KDM6A, BTG2, EP300, GATA4, HOXA6, RNF2, YY1, PCSK6, HOXA7, HOXC4, CELSR1, OTX1, HOXC10, TSHZ1, HOXC13, FEZF1, SFRP2, VANGL2, NRARP, CELSR2, CTNNBIP1, HES4, HES1, HOXA10, NOTCH1, DLL1, LHX1, PCSK5, SMO, HOXC6, TBX1, MSX2, SIX3, LFNG, PLD6, MEOX1, TBX6, ZEB2, NKX3-1, WNT3A, TGFBR1, POFUT1, CFC1B, NOG, CRKL, CDX2, RIPPLY1, FZD5, SOX17, CRB2, HES6, LRP5L, ACVR2A, GBX2, BPTF, CER1 | 100 |
| 12 | GO:0045104 | intermediate filament cytoskeleton organization | ATP8A2, KRT6C, NEFL, KRT71, FAM83H, VIM, DNAJB6, NEFM, KRT18, KRT3, PLEC, KRT74, RAF1, KRT2, PPL, KRT6A, PKP1, EPPK1 | 100 |

| Cluster | Term | Name | HPOs\_in\_clusters |
| --- | --- | --- | --- |
| 12 | OMIM:129400 | RAPP-HODGKIN SYNDROME; RHS | HP:0000430, HP:0001792 |
| 12 | OMIM:219000 | FRASER SYNDROME 1; FRASRS1 | HP:0000430, HP:0006610 |
| 12 | OMIM:300209 | SIMPSON-GOLABI-BEHMEL SYNDROME, TYPE 2; SGBS2 | HP:0001792, HP:0006610 |
| 12 | OMIM:616580 | AU-KLINE SYNDROME; AUKS | HP:0000430, HP:0006610 |

---


---


---

# Cluster 16

| Cluster | Term | Name |
| --- | --- | --- |
| 16 | HP:0000316 | Hypertelorism |
| 16 | HP:0000431 | Wide nasal bridge |
| 16 | HP:0000581 | Blepharophimosis |

| Cluster | Term | Name | Genes | Percentage\_of\_nodes\_with\_funsys |
| --- | --- | --- | --- | --- |
| 16 | GO:0072376 | protein activation cascade | SERPINC1, IGLL1, IGHV3-33, IGHG4, IGLC6, IGLV2-23, TFPI, CR1, IGHV4-28, IGHV4-39, C8G, IGHV5-51, IGHV3-7, KRT1, F9, IGLV3-25, CFHR4, IGHV1-45, IGHE, TRBC2, CFHR5, IGHV3-48, IGLV2-8, IGHG2, IGHD, IGLV2-11, IGHV3-43, IGHV1-58, APCS, C1QBP, IGLV3-1, CFHR2, IGHV2-70, IGHM, GP5, C6, C4BPB, IGLV1-51, CPB2, IGHV3-49, SCARA3, CRP, IGHV4-61, IGHV4-59, CFHR1, C4BPA, FCN1, KLKB1, C1QC, IGHV3-30, IGLC1, IGHV1-3, F7, IGHV4-4, F12, IGLV7-43, IGLV3-27, C1QB, IGHV2-26, IGLV3-19, CR2, IGHV3-15, IGLV1-40, C9, CFH, IGHV3-21, FBLN1, RGCC, IGHV3-53, CFD, F8, CPN2, FCN2, IGHG3, FGA, IGLC2, CD46, IGHA2, MASP2, F13B, IGHV3-11, SUSD4, IGLC7, CD55, IGHV3-66, IGHV3-73, IGHV3-13, F10, FGG, IGHV1-18, COL20A1, MASP1, IGLV2-14, KNG1, IGLL5, IGHV1OR15-1, IGLV1-47, FCN3, IGHV3-72, A2M, IGLV3-21, IGHV1-69, IGHV3-74, IGHV6-1, GP1BB, IGHV3-64, FGB, COLEC10, GP1BA, VSIG4, IGLV1-44, IGLC3, IGHA1, C1QA, IGHG1, IGLV6-57, C5, IGHV4-34, F11, C7, IGHV3-20, IGHV1-24, IGHV2-5, CD19, CLU, IGHV3-23 | 100 |
| 16 | GO:0002455 | humoral immune response mediated by circulating immunoglobulin | IGLL1, IGHV3-33, IGHG4, IGLC6, IGLV2-23, CR1, IGHV4-28, IGHV4-39, C8G, IGHV5-51, IGHV3-7, IGLV3-25, EXO1, IGHV1-45, IGHE, TRBC2, IGHV3-48, IGLV2-8, IGHG2, IGHD, IGLV2-11, IGHV3-43, IGHV1-58, APCS, C1QBP, IGLV3-1, IGHV2-70, IGHM, FCGR2B, C6, C4BPB, IGLV1-51, IGHV3-49, CRP, IGHV4-61, IGHV4-59, C4BPA, C1QC, IGHV3-30, IGLC1, IGHV1-3, IGHV4-4, IGLV7-43, IGLV3-27, C1QB, IGHV2-26, IGLV3-19, CR2, IGHV3-15, IGLV1-40, C9, IGHV3-21, IGHV3-53, IGHG3, CD46, IGLC2, IGHA2, MASP2, IGHV3-11, SUSD4, IGLC7, CD55, IGHV3-66, IGHV3-73, IGHV3-13, IGHV1-18, PTPRC, IGLV2-14, IGLL5, IGHV1OR15-1, IGLV1-47, IGHV3-72, IGLV3-21, IGHV1-69, IGHV3-74, IGHV6-1, IGHV3-64, IGLV1-44, IGLC3, IGHA1, C1QA, IGHG1, IGLV6-57, C5, IGHV4-34, C7, IGHV3-20, IGHV1-24, IGHV2-5, CLU, IGHV3-23 | 100 |
| 16 | GO:0006910 | phagocytosis, recognition | IGHV3-53, IGLL1, IGHV3-33, IGHV1-69, IGHV4-61, IGHG4, IGLC6, IGHV4-59, FCN2, PEAR1, IGHV4-28, IGHV4-39, ADGRB1, IGHG3, IGHV5-51, IGHV3-7, MEGF10, CD36, IGLC2, FCN1, IGHA2, IGHV3-74, IGHV6-1, IGHV3-11, IGHV3-30, IGHE, IGHV1-45, IGHV3-64, TRBC2, IGLC7, IGLC1, IGHV3-48, IGHV3-66, IGHV3-73, MFGE8, IGHG2, IGHD, IGLC3, IGHA1, IGHV1-3, IGHV3-43, IGHG1, IGHV4-4, COLEC12, CLEC7A, IGHV1-58, IGHV3-13, IGHV4-34, IGHV1-18, IGHV2-26, IGHV2-70, IGHM, IGHV3-20, IGHV1-24, IGLL5, IGHV2-5, IGHV1OR15-1, IGHV3-15, FCN3, IGHV3-21, IGHV3-72, IGHV3-23, IGHV3-49 | 100 |
| 16 | GO:0030449 | regulation of complement activation | IGHV3-53, IGLV3-21, IGHV3-33, IGHV1-69, IGHG4, IGLC6, IGLV2-23, CPN2, CR1, IGHV4-59, IGHV4-39, IGHG3, CFHR1, C8G, IGHV3-7, CD46, C4BPA, CFHR4, IGLV3-25, SUSD4, IGHV3-11, C1QC, IGHV3-30, CFHR5, IGLC7, CD55, IGHV3-48, IGLC1, IGLV2-8, IGLV1-44, IGHG2, IGLV2-11, C1QA, IGHG1, IGLV6-57, IGLV7-43, C5, IGHV3-13, IGLV3-27, IGHV4-34, IGLV3-1, C1QB, CFHR2, IGLV3-19, IGHV2-70, C7, C1QBP, IGLV2-14, CR2, C6, C4BPB, IGHV2-5, IGHV1OR15-1, IGLV1-47, IGLV1-40, CD19, C9, CFH, CLU, IGLV1-51, IGHV3-23, CPB2, A2M | 100 |
| 16 | GO:0019724 | B cell mediated immunity | IGLL1, IGHV3-33, CD40LG, IGHG4, IGLC6, IGLV2-23, FOXP3, CR1, IGHV4-28, IGHV4-39, C8G, IGHV5-51, IGHV3-7, IGLV3-25, EXO1, MLH1, IGHV1-45, IGHE, TNFSF4, TRBC2, IGHV3-48, IGLV2-8, IGHG2, IGHD, IGLV2-11, IGHV3-43, BCL6, IL13RA2, IGHV1-58, APCS, C1QBP, IGLV3-1, IGHV2-70, IGHM, TNFSF13, FCGR2B, C6, C4BPB, XCL1, MAD2L2, IGLV1-51, IGHV3-49, CRP, SHLD2, FCER1G, IGHV4-61, THOC1, IL4, INPP5D, IGHV4-59, C4BPA, C1QC, IGHV3-30, RIF1, IGLC1, APLF, IGHV3-23, IGHV1-3, LIG4, IGHV4-4, IGLV7-43, IGLV3-27, C1QB, IGHV2-26, IGLV3-19, PAXIP1, CR2, HSPD1, IGHV3-15, IGLV1-40, CD28, C9, IGHV3-21, IGHV3-53, IGHG3, IGLC2, CD46, IGHA2, IL2, MASP2, IGHV3-11, SUSD4, IL10, IGLC7, CD55, IGHV3-66, IGHV3-73, BTK, IGHV3-13, GAPT, IGHV1-18, PTPRC, IGLV2-14, IGLL5, IGHV1OR15-1, IGLV1-47, IGHV3-72, ATAD5, NSD2, IGLV3-21, MSH6, IGHV1-69, CCR6, NBN, BCL10, RNF168, IGHV3-74, IGHV6-1, IGHV3-64, MSH2, NDFIP1, IL4R, IGLV1-44, IGLC3, IGHA1, C1QA, IGHG1, IGLV6-57, CD226, C5, IGHV4-34, C7, CLCF1, IGHV3-20, IGHV1-24, IGHV2-5, CLU, SLA2, TFRC | 100 |
| 16 | GO:0038096 | Fc-gamma receptor signaling pathway involved in phagocytosis | IGHV3-33, IGHG4, IGLC6, IGLV2-23, DOCK1, IGHV4-39, VAV2, IGHV3-7, PLCG2, IGLV3-25, WASL, IGHV3-48, IGLV2-8, IGHG2, IGLV2-11, ACTB, CD247, PLA2G6, PRKCE, IGLV3-1, IGHV2-70, FCGR2B, MAPK1, CRK, WASF2, GRB2, ARPC5, IGLV1-51, ARPC4, CD47, RAPGEF1, RAP1A, IGHV4-59, CYFIP1, IGHV3-30, HSP90AA1, MYO1C, ARPC1B, IGLC1, IGLV7-43, IGLV3-27, IGLV3-19, MYO1G, RAC1, IGLV1-40, FCGR1A, YES1, IGHV3-53, WAS, FGR, HCK, ACTR2, IGHG3, FCGR2A, PTPRJ, ABL1, IGHV3-11, IGLC7, ELMO1, PTK2, IGHV3-13, PTPRC, WIPF1, IGLV2-14, IGHV1OR15-1, IGLV1-47, IGLV3-21, BRK1, ABI1, IGHV1-69, NCKAP1L, PIK3CB, MAPK3, MYO10, NCK1, ARPC1A, IGLV1-44, IGHG1, IGLV6-57, SRC, IGHV4-34, WIPF3, FCGR3A, IGHV2-5, NCKAP1, FYN, LIMK1, IGHV3-23 | 100 |
| 16 | GO:0050867 | positive regulation of cell activation | CD83, TESPA1, IGLL1, SHH, IGHV3-33, EGR3, CD40LG, SIRPB1, GAS6, IGHG4, IGLC6, FOXP3, CSK, TNFSF11, IGHV4-28, IGHV4-39, TMIGD2, IGHV5-51, IGHV3-7, PAK2, EFNB2, AP3D1, MLH1, MIR92A2, IGHV1-45, IGHE, CD1D, TRBC2, TNFSF4, PCID2, IGHV3-48, CTLA4, IGHG2, IGHD, CAV1, DOCK8, IGHV3-43, TNFRSF14, CLECL1, DNAJA3, BCL6, IGHV1-58, CARD11, ANXA1, HMGB1, IGHV2-70, IGHM, TNFSF13, GRB2, IL7, TAC1, XCL1, VCAM1, PTAFR, ICOS, MAD2L2, SLC39A10, IGHV3-49, SHLD2, PYCARD, CD274, ITPKB, FCER1G, CD276, CD47, PAK3, IGHV4-61, DPP4, IL4, INPP5D, IGHV4-59, KLRK1, MIR30B, LEP, MIR21, SART1, IL6, IGHV3-30, GLI3, GPR183, RIF1, PPP2R3C, IL6ST, FADD, IGLC1, IGHV3-23, BAD, ITGB2, CRLF2, PDPK1, IGHV1-3, FLT3LG, TNFRSF13C, LBP, FGF10, IGHV4-4, ADAM8, RIPK2, IGHV2-26, NLRP3, IRS2, PAXIP1, IL1RL1, HSPD1, CBFB, SIRPA, HHLA2, RAC1, STXBP1, IGHV3-15, CD28, IGHV3-21, LAMP1, JAK2, YES1, CORO1A, MAP3K8, IGHV3-53, PDCD1LG2, IL5, FGR, IL23R, MIR92A1, ADORA2B, LILRB1, MIF, GPAM, IGHG3, IGLC2, CD46, NFATC2, IGHA2, IL2, IL10, IGHV3-11, EBI3, SOCS5, ZP4, HES1, NKAP, IGLC7, CD55, STAP1, MALT1, IGHV3-66, IGHV3-73, TNIP2, LGALS1, PRKCZ, SELP, BCL2, BTK, IL21, MEF2C, PDPN, ATP11C, AP1G1, IL2RA, IGHV3-13, IGHV1-18, PTPRC, GATA3, HSPH1, SPTA1, IL1RL2, IGLL5, FCRL3, IGHV1OR15-1, CD86, IGHV3-72, SOX15, HLX, ATAD5, NSD2, MSH6, NPPA, SASH3, IGHV1-69, EPO, CD38, BCL10, TNFRSF4, PLEK, NCKAP1L, WNT3A, IL12B, IL12A, GRAP2, DMTN, EFNB3, IGHV3-74, IGHV6-1, SOCS1, BST1, IGHV3-64, MSH2, NCK1, RASAL3, ITGAM, IL15, IL4R, KLRC4-KLRK1, AKT1, CD80, IGLC3, IGHA1, CCDC88B, PDCD1, XBP1, LILRB4, IGHG1, SRC, DUSP10, CD226, IGHV4-34, IL33, CLCF1, STX4, TNFSF13B, IGHV3-20, IGHV1-24, LILRB2, F2RL1, IGHV2-5, SIRPG, PRKCQ, NR4A3, FYN, BTLA, PELI1, TICAM1, IL13, TFRC, TIRAP | 100 |
| 16 | GO:0006958 | complement activation, classical pathway | IGLL1, IGHV3-33, IGHG4, IGLC6, IGLV2-23, CR1, IGHV4-28, IGHV4-39, C8G, IGHV5-51, IGHV3-7, IGLV3-25, IGHV1-45, IGHE, TRBC2, IGHV3-48, IGLV2-8, IGHG2, IGHD, IGLV2-11, IGHV3-43, IGHV1-58, APCS, C1QBP, IGLV3-1, IGHV2-70, IGHM, C6, C4BPB, IGLV1-51, IGHV3-49, CRP, IGHV4-61, IGHV4-59, C4BPA, C1QC, IGHV3-30, IGLC1, IGHV1-3, IGHV4-4, IGLV7-43, IGLV3-27, C1QB, IGHV2-26, IGLV3-19, CR2, IGHV3-15, IGLV1-40, C9, IGHV3-21, IGHV3-53, IGHG3, IGLC2, CD46, IGHA2, MASP2, IGHV3-11, SUSD4, IGLC7, CD55, IGHV3-66, IGHV3-73, IGHV3-13, IGHV1-18, IGLV2-14, IGLL5, IGHV1OR15-1, IGLV1-47, IGHV3-72, IGLV3-21, IGHV1-69, IGHV3-74, IGHV6-1, IGHV3-64, IGLV1-44, IGLC3, IGHA1, C1QA, IGHG1, IGLV6-57, C5, IGHV4-34, C7, IGHV3-20, IGHV1-24, IGHV2-5, CLU, IGHV3-23 | 100 |
| 16 | GO:0050851 | antigen receptor-mediated signaling pathway | TESPA1, IGLL1, IGHV3-33, RAB29, IGHG4, IGLC6, PVRIG, PAG1, FOXP3, CSK, KLHL6, DENND1B, IGHV4-28, IGHV4-39, BLK, DUSP22, IGHV5-51, IGHV3-7, PLCG2, PAK2, IKBKG, IGHV1-45, IGHE, TRBC2, IGHV3-48, CTLA4, HRAS, PTPN2, LCP2, IGHG2, IGHD, NFAM1, RBCK1, IGHV3-43, RELA, CD247, IGHV1-58, PAWR, CARD11, IGHV2-70, IGHM, GCSAM, MAPK1, ELF2, PHPT1, SLC39A10, LAT2, PDE4D, IGHV3-49, CD276, PAK3, IGHV4-61, INPP5D, TXK, IGHV4-59, CMTM3, MNDA, LIME1, TRAT1, BTNL9, PIK3CD, IKBKB, IGHV3-30, IGLC1, PAX5, IGHV3-23, PDPK1, IGHV1-3, IGHV4-4, RIPK2, ERMAP, IGHV2-26, SH2B2, DUSP3, CBFB, HHLA2, IGHV3-15, CD28, PLEKHA1, IGHV3-21, EZR, IGHV3-53, WAS, CACNA1F, BTNL3, LAT, GCSAML, RC3H2, IGHG3, IGLC2, PTPRJ, NFATC2, IGHA2, ABL1, IGHV3-11, FYB1, PLCL2, RC3H1, IGLC7, STAP1, MALT1, IGHV3-66, IGHV3-73, BCL2, STOML2, BTK, MEF2C, PLCG1, IGHV3-13, LGALS3, PDE4B, IGHV1-18, PTPRC, BTNL8, GATA3, IGLL5, FCRL3, IGHV1OR15-1, IGHV3-72, GPS2, IGHV1-69, BTNL10, CD38, BCL10, NCKAP1L, PIK3CB, GRAP2, IGHV3-74, IGHV6-1, IGHV3-64, NCK1, THEMIS2, IGLC3, IGHA1, LILRB4, CD300A, IGHG1, CD226, IGHV4-34, STK11, BAX, TEC, IGHV3-20, IGHV1-24, IGHV2-5, ELF1, CD19, PRKCQ, FYN, RFTN1, BRAF, SLA2, EIF2B5 | 100 |
| 16 | GO:0042113 | B cell activation | POLM, IGLL1, IGHV3-33, CD40LG, IGHG4, IGLC6, FOXP3, HDAC4, IGHV4-28, IGHV4-39, IFNA5, IGHV5-51, IGHV3-7, PLCG2, CHRNA4, EXO1, MLH1, IGHV1-45, IGHE, IFNB1, HHEX, TRBC2, TNFSF4, PCID2, IGHV3-48, CTLA4, PTPN2, IGHG2, IGHD, NFAM1, IGHV3-43, INHBA, BCL6, IGHV1-58, PAWR, CARD11, IFNA14, IRF2BP2, IGHV2-70, IGHM, TNFSF13, FCGR2B, AHR, TNFRSF13B, IL7, VCAM1, MAD2L2, LAT2, SLC39A10, IGHV3-49, SHLD2, IFNA6, IFNA4, IFNA10, IGHV4-61, THOC1, HDAC9, IFNA13, IL4, AKAP17A, INPP5D, IGHV4-59, MNDA, PIK3CD, IFNE, IL6, IGHV3-30, GPR183, RIF1, PPP2R3C, IGLC1, APLF, FZD9, IGHV3-23, BAD, IFNA16, IGHV1-3, GON4L, LIG4, TNFRSF13C, IGHV4-4, FCRL1, IFNA2, EP300, IGHV2-26, IRS2, PAXIP1, CR2, HSPD1, IGHV3-15, ITM2A, CD28, IFNA1, IGHV3-21, ONECUT1, LYL1, IGHV3-53, IL5, FNIP1, CDH17, MIF, IGHG3, CASP8, IGLC2, PTPRJ, NFATC2, IGHA2, IL2, TPD52, IGHV3-11, IL10, ABL1, PLCL2, RC3H1, IGLC7, MALT1, IGHV3-66, IGHV3-73, TNIP2, LGALS1, BCL2, LRRC8A, ZFP36L2, CASP3, DLL1, BTK, ITGA4, IL21, IFNA17, MEF2C, ATP11C, IGHV3-13, GAPT, IGHV1-18, PTPRC, IFNA7, LFNG, IGLL5, FCRL3, IGHV1OR15-1, IGHV3-72, IFNA21, ATAD5, NSD2, GPS2, MSH6, SASH3, PTK2B, IGHV1-69, SLA2, CCR6, NTRK1, CD38, NBN, TNFRSF4, NCKAP1L, WNT3A, SKAP2, RNF168, IGHV3-74, IGHV6-1, BST1, IGHV3-64, MSH2, MFNG, IFNK, NDFIP1, HDAC5, DNAJB9, TCF3, BAK1, KLF6, IFNA8, IGLC3, IGHA1, MIR17HG, SP3, XBP1, ZBTB7A, CD300A, IGHG1, IGHV4-34, TBC1D10C, PKN1, CLCF1, TNFSF13B, BAX, IGHV3-20, IGHV1-24, SFRP1, IGHV2-5, DOCK11, LAX1, PELI1, TICAM1, DCLRE1C, ITGB1, IFNW1, IL13, ZFP36L1, TFRC, TIRAP | 100 |
| 16 | GO:0006909 | phagocytosis | SYT11, IGLL1, IGHV3-33, SIRPB1, GAS6, IGHG4, IGLC6, IGLV2-23, CSK, MSR1, SH3BP1, DOCK1, IGHV4-28, IGHV4-39, IRF8, VAV2, IGHV5-51, IGHV3-7, PLCG2, PEAR1, ELMO3, IGLV3-25, SPON2, MESD, RUBCN, IGHV1-45, IGHE, TRBC2, PRTN3, WASL, IGHV3-48, IGLV2-8, IGHG2, IGHD, IGLV2-11, ACTB, ATG5, AZU1, IGHV3-43, EIF2AK1, CD247, IGHV1-58, PLA2G6, ANXA1, PRKCG, IGLV3-1, HMGB1, TLR2, IGHV2-70, IGHM, FCGR2B, MAPK1, CRK, WASF2, LMAN2, GRB2, ARPC5, C4BPB, ATG3, PPARG, PTX3, IGLV1-51, IGHV3-49, ARPC4, CRP, PYCARD, CD47, FCER1G, NCF4, RAPGEF1, IGHV4-61, RAP1A, ITGAL, IGHV4-59, LEP, CD36, RAB27A, CYFIP1, C4BPA, SFTPD, FCN1, SLAMF1, LYAR, IGHV3-30, HSP90AA1, MYO1C, ARPC1B, RAB14, IGLC1, MERTK, ITGB2, CAMK1D, IGHV1-3, LBP, RACK1, IGHV4-4, COLEC12, ANXA11, CLEC7A, TMEM175, IGLV7-43, ABR, IGLV3-27, PTEN, IGHV2-26, ARHGAP12, IGLV3-19, ADORA1, TM9SF4, MYO1G, SIRPA, RAC1, IGHV3-15, IGLV1-40, LEPR, IGHV3-21, FCGR1A, ANXA3, RHOBTB2, CORO1A, YES1, IGHV3-53, WAS, GSN, FGR, DOCK2, HCK, FCN2, ACTR2, IGHG3, MEGF10, FCGR2A, IGLC2, PTPRJ, IGHA2, ABL1, IGHV3-11, ITGAV, BCR, CALR, IGLC7, STAP1, CDC42SE2, IGHV3-66, IGHV3-73, MIR17, CD14, CYBA, DYSF, ELMO1, PTK2, RAB20, IGHV3-13, IGHV1-18, PTPRC, WIPF1, ADIPOQ, IGLV2-14, IGLL5, IGHV1OR15-1, AHSG, IGLV1-47, CD302, FCN3, IGHV3-72, MIR20A, IGLV3-21, BRK1, ABI1, IGHV1-69, PLD4, NCKAP1L, PIK3CB, ADGRB1, ARHGAP25, MAPK3, MYO10, MYH9, PIP5K1C, IGHV3-74, IGHV6-1, IGHV3-64, NCK1, ARPC1A, ITGAM, ABCA7, MFGE8, IGLV1-44, NR1H3, IGLC3, IGHA1, MET, PRKCE, TGM2, CD300A, IGHG1, IGLV6-57, SRC, ELANE, IGHV4-34, GULP1, WIPF3, IGHV3-20, IGHV1-24, NCF2, XKR8, FCGR3A, F2RL1, IGHV2-5, NCKAP1, SIRPG, FYN, LIMK1, ITGB1, IGHV3-23 | 100 |
| 16 | GO:0038094 | Fc-gamma receptor signaling pathway | IGHV3-33, IGHG4, IGLC6, IGLV2-23, DOCK1, IGHV4-39, VAV2, IGHV3-7, PLCG2, IGLV3-25, WASL, IGHV3-48, IGLV2-8, IGHG2, IGLV2-11, ACTB, CD247, PLA2G6, PRKCE, IGLV3-1, IGHV2-70, FCGR2B, MAPK1, CRK, WASF2, GRB2, ARPC5, IGLV1-51, ARPC4, CD47, FCER1G, RAPGEF1, RAP1A, IGHV4-59, CYFIP1, IGHV3-30, HSP90AA1, MYO1C, ARPC1B, IGLC1, IGLV7-43, IGLV3-27, IGLV3-19, MYO1G, RAC1, IGLV1-40, FCGR1A, YES1, IGHV3-53, WAS, FGR, HCK, ACTR2, IGHG3, FCGR2A, PTPRJ, ABL1, IGHV3-11, IGLC7, ELMO1, PTK2, IGHV3-13, PTPRC, WIPF1, IGLV2-14, IGHV1OR15-1, IGLV1-47, IGLV3-21, BRK1, ABI1, IGHV1-69, NCKAP1L, PIK3CB, MAPK3, MYO10, NCK1, ARPC1A, IGLV1-44, IGHG1, IGLV6-57, SRC, IGHV4-34, WIPF3, FCGR3A, IGHV2-5, NCKAP1, FYN, LIMK1, IGHV3-23 | 100 |
| 16 | GO:0016485 | protein processing | SEC11C, CTSH, LGMN, SHH, IGHV3-33, IGHG4, IGLC6, IGLV2-23, KLK13, CR1, IGHV4-39, C8G, F9, IGHV3-7, MELTF, ASPH, IGLV3-25, CFHR4, IMMP1L, ASPRV1, PRSS37, CFHR5, IGHV3-48, IGLV2-8, IGHG2, KLK2, IGLV2-11, YME1L1, USP17L2, MME, HM13, IGLV3-1, KLK3, CFHR2, IGHV2-70, C6, C4BPB, PISD, PCSK4, CPXM1, ACP4, IGLV1-51, CPB2, PYCARD, CLN5, IMMP2L, CGB3, CTSE, CPXM2, CNTN2, IGHV4-59, CFHR1, C4BPA, DDI2, ADAMTS2, KLKB1, C1QC, IGHV3-30, GLI3, KLK1, ERO1B, FADD, IGLC1, XPNPEP3, CPE, BAD, PMPCA, F7, SERPINF2, ANXA2P2, F12, ADAM8, IGLV7-43, IGLV3-27, RIPK2, C1QB, IGLV3-19, SCG5, CR2, PLAU, C9orf3, XIAP, PCSK6, IGLV1-40, REN, C9, PLGRKT, SPCS3, CFH, TMEM59, IGHV3-53, GSN, MMP16, CPN2, PSEN2, PRSS3, SEC11B, IGHG3, FGA, CD46, IGHV3-11, SUSD4, ENO1, PGK1, IGLC7, CD55, PCSK1N, TIMM17A, CASP3, PTCH1, STOML2, CAPN2, PCSK5, NCSTN, CARD8, PCSK9, PARP1, IGHV3-13, FGG, FXN, IGLV2-14, CTSZ, CLN3, CUZD1, PMPCB, IGHV1OR15-1, IFI16, SERPINE2, IGLV1-47, CASP2, LHB, AEBP1, ANGPTL8, A2M, IGLV3-21, IGHV1-69, KLK6, TBC1D10A, ADAMTS13, MYH9, CPZ, FGB, SERPINE1, NLRP7, GRIN2A, AFG3L2, ECE2, CPA3, BIRC7, BACE2, BAK1, IGLV1-44, BCL2L12, C1QA, IGHG1, IGLV6-57, SRC, C5, IGHV4-34, F11, C7, IL1R2, PRSS12, IGHV2-5, CD19, CLU, TNP2, IGHV3-23, CCBE1 | 100 |
| 16 | GO:1903317 | regulation of protein maturation | IGHV3-33, IGHG4, IGLC6, IGLV2-23, CR1, IGHV4-39, C8G, MELTF, IGHV3-7, IGLV3-25, CFHR4, PRSS37, CFHR5, IGHV3-48, IGLV2-8, IGHG2, IGLV2-11, USP17L2, NKD2, NOL3, C1QBP, IGLV3-1, CFHR2, IGHV2-70, C6, C4BPB, ACP4, IGLV1-51, CPB2, PRKACB, SOX4, CNTN2, IGHV4-59, CFHR1, C4BPA, KLKB1, C1QC, IGHV3-30, IGLC1, SERPINF2, ANXA2P2, F12, ADAM8, IGLV7-43, IGLV3-27, C1QB, IGLV3-19, CR2, XIAP, IGLV1-40, C9, PLGRKT, CFH, TMEM59, IGHV3-53, GSN, CPN2, IGHG3, TFR2, CD46, IGHV3-11, SUSD4, ENO1, IGLC7, CD55, TIMM17A, CARD8, IGHV3-13, IGLV2-14, CTSZ, IGHV1OR15-1, SERPINE2, IGLV1-47, ANGPTL8, A2M, IGLV3-21, IGHV1-69, MYH9, SERPINE1, NLRP7, BIRC7, IGLV1-44, BCL2L12, C1QA, IGHG1, IGLV6-57, SRC, C5, IGHV4-34, C7, IL1R2, IGHV2-5, CD19, CLU, TNP2, IGHV3-23, CCBE1, GLG1 | 100 |
| 16 | GO:0050853 | B cell receptor signaling pathway | IGLL1, IGHV3-33, IGHG4, IGLC6, KLHL6, BLK, IGHV4-28, IGHV4-39, IGHV5-51, IGHV3-7, PLCG2, IGHV1-45, IGHE, TRBC2, IGHV3-48, CTLA4, IGHG2, IGHD, NFAM1, IGHV3-43, IGHV1-58, IGHV2-70, IGHM, GCSAM, MAPK1, ELF2, SLC39A10, LAT2, IGHV3-49, IGHV4-61, IGHV4-59, CMTM3, MNDA, LIME1, PIK3CD, IGHV3-30, IGLC1, PAX5, IGHV1-3, IGHV4-4, IGHV2-26, CBFB, IGHV3-15, PLEKHA1, IGHV3-21, IGHV3-53, GCSAML, IGHG3, IGLC2, NFATC2, IGHA2, ABL1, IGHV3-11, PLCL2, IGLC7, STAP1, IGHV3-66, IGHV3-73, BCL2, BTK, MEF2C, IGHV3-13, IGHV1-18, PTPRC, IGLL5, FCRL3, IGHV1OR15-1, IGHV3-72, GPS2, IGHV1-69, CD38, NCKAP1L, IGHV3-74, IGHV6-1, IGHV3-64, IGLC3, IGHA1, CD300A, IGHG1, IGHV4-34, BAX, TEC, IGHV3-20, IGHV1-24, IGHV2-5, ELF1, CD19, RFTN1, IGHV3-23 | 100 |
| 16 | GO:0016064 | immunoglobulin mediated immune response | IGLL1, IGHV3-33, CD40LG, IGHG4, IGLC6, IGLV2-23, FOXP3, CR1, IGHV4-28, IGHV4-39, C8G, IGHV5-51, IGHV3-7, IGLV3-25, EXO1, MLH1, IGHV1-45, IGHE, TNFSF4, TRBC2, IGHV3-48, IGLV2-8, IGHG2, IGHD, IGLV2-11, IGHV3-43, BCL6, IL13RA2, IGHV1-58, APCS, C1QBP, IGLV3-1, IGHV2-70, IGHM, TNFSF13, FCGR2B, C6, C4BPB, XCL1, MAD2L2, IGLV1-51, IGHV3-49, CRP, SHLD2, FCER1G, IGHV4-61, THOC1, IL4, INPP5D, IGHV4-59, C4BPA, C1QC, IGHV3-30, RIF1, IGLC1, APLF, IGHV1-3, LIG4, IGHV4-4, IGLV7-43, IGLV3-27, C1QB, IGHV2-26, IGLV3-19, PAXIP1, CR2, HSPD1, IGHV3-15, IGLV1-40, CD28, C9, IGHV3-21, IGHV3-53, IGHG3, IGLC2, CD46, IGHA2, IL2, MASP2, IGHV3-11, SUSD4, IL10, IGLC7, CD55, IGHV3-66, IGHV3-73, BTK, IGHV3-13, GAPT, IGHV1-18, PTPRC, IGLV2-14, IGLL5, IGHV1OR15-1, IGLV1-47, IGHV3-72, ATAD5, NSD2, IGLV3-21, MSH6, IGHV1-69, CCR6, NBN, BCL10, RNF168, IGHV3-74, IGHV6-1, IGHV3-64, MSH2, NDFIP1, IL4R, IGLV1-44, IGLC3, IGHA1, C1QA, IGHG1, IGLV6-57, CD226, C5, IGHV4-34, C7, CLCF1, IGHV3-20, IGHV1-24, IGHV2-5, CLU, IGHV3-23, TFRC | 100 |
| 16 | GO:0099024 | plasma membrane invagination | IGLL1, IGHV3-33, IGHG4, IGLC6, MSR1, SH3BP1, DOCK1, IGHV4-28, IGHV4-39, IGHV5-51, IGHV3-7, IGHV1-45, IGHE, TRBC2, IGHV3-48, IGHG2, IGHD, IGHV3-43, IGHV1-58, IGHV2-70, IGHM, FCGR2B, PPARG, ALKBH4, IGHV3-49, FCER1G, IGHV4-61, IGHV4-59, CD36, IGHV3-30, IGLC1, ITGB2, IGHV1-3, IGHV4-4, IGHV2-26, ARHGAP12, IGHV3-15, IGHV3-21, FCGR1A, RHOBTB2, IGHV3-53, GSN, IGHG3, MEGF10, IGLC2, IGHA2, SPIRE2, IGHV3-11, IGLC7, STAP1, IGHV3-66, IGHV3-73, ELMO1, IGHV3-13, IGHV1-18, IGLL5, IGHV1OR15-1, IGHV3-72, ARF1, IGHV1-69, NCKAP1L, ADGRB1, ARHGAP25, MYH9, IGHV3-74, IGHV6-1, IGHV3-64, ITGAM, ABCA7, MFGE8, SNX9, IGLC3, IGHA1, CD300A, IGHG1, SPIRE1, IGHV4-34, SNX33, GULP1, IGHV3-20, IGHV1-24, XKR8, F2RL1, IGHV2-5, IGHV3-23 | 100 |
| 16 | GO:0038093 | Fc receptor signaling pathway | IGHV3-33, IGHG4, IGLC6, IGLV2-23, DOCK1, IGHV4-39, VAV2, IGHV3-7, PLCG2, IGLV3-25, PAK2, IKBKG, IGHE, WASL, IGHV3-48, IGLV2-8, LCP2, IGHG2, IGLV2-11, ACTB, RELA, CD247, CARD11, PLA2G6, PRKCE, IGLV3-1, IGHV2-70, FCGR2B, MAPK1, CRK, WASF2, GRB2, ARPC5, PPP3CB, LAT2, IGLV1-51, ARPC4, MAPK10, CD47, FCER1G, RAPGEF1, RAP1A, IGHV4-59, LILRA4, CYFIP1, IKBKB, IGHV3-30, HSP90AA1, MYO1C, ARPC1B, IGLC1, PPP3R1, PDPK1, IGLV7-43, IGLV3-27, IGLV3-19, MYO1G, RAC1, IGLV1-40, FCGR1A, MAP3K1, YES1, IGHV3-53, WAS, FGR, HCK, LAT, ACTR2, IGHG3, FCGR2A, PTPRJ, NFATC2, ABL1, IGHV3-11, IGLC7, MALT1, BTK, ELMO1, PTK2, IGHV3-13, PTPRC, WIPF1, CD200R1, IGLV2-14, IGHV1OR15-1, IGLV1-47, TAB1, IGLV3-21, BRK1, ABI1, IGHV1-69, BCL10, FCER1A, NCKAP1L, PIK3CB, MAPK3, MYO10, GRAP2, NCK1, ARPC1A, IGLV1-44, NFATC1, IGHG1, IGLV6-57, SRC, PIGR, IGHV4-34, WIPF3, TEC, FCGR3A, IGHV2-5, NCKAP1, PRKCQ, MAPK9, FYN, LIMK1, NR4A3, IGHV3-23 | 100 |
| 16 | GO:0002429 | immune response-activating cell surface receptor signaling pathway | TESPA1, IGLL1, IGHV3-33, CARD9, FPR1, RAB29, IGHG4, IGLC6, IGLV2-23, PVRIG, PAG1, CR1, CSK, KLHL6, FOXP3, DOCK1, IGHV4-28, IGHV4-39, DENND1B, DUSP22, IGHV5-51, IGHV3-7, VAV2, PLCG2, BLK, IGLV3-25, PAK2, IKBKG, IGHV1-45, IGHE, TRBC2, WASL, IGHV3-48, CTLA4, HRAS, IGLV2-8, PTPN2, CLEC10A, IGHG2, IGHD, NFAM1, IGLV2-11, LILRA2, ACTB, LCP2, RBCK1, MUC7, IGHV3-43, RELA, CD247, IGHV1-58, MUC17, FPR2, CARD11, PLA2G6, PRKCE, IGLV3-1, PAWR, IGHV2-70, IGHM, FCGR2B, MAPK1, CRK, WASF2, GCSAM, GRB2, ELF2, ARPC5, PHPT1, SLC39A10, LAT2, IGLV1-51, IGHV3-49, PDE4D, ARPC4, CD276, CD47, FCER1G, PRKACB, PAK3, RAPGEF1, IGHV4-61, RAP1A, INPP5D, TXK, IGHV4-59, CREBBP, MNDA, LIME1, CMTM3, KLRK1, TRAT1, BTNL9, PIK3CD, CYFIP1, IKBKB, FCN1, FPR3, IGHV3-30, HSP90AA1, MYO1C, ARPC1B, IGLC1, PAX5, PDPK1, IGHV1-3, IGHV4-4, IGLV7-43, EP300, IGLV3-27, RIPK2, IGHV2-26, ERMAP, IGLV3-19, SH2B2, DUSP3, CR2, MYO1G, CBFB, HHLA2, RAC1, IGHV3-15, IGLV1-40, MUCL1, MUC4, PLEKHA1, CD28, IGHV3-21, FCGR1A, MUC3A, EZR, YES1, IGHV3-53, WAS, CACNA1F, FGR, BTNL3, PLSCR1, LAT, HCK, GCSAML, RC3H2, ACTR2, IGHG3, FCGR2A, IGLC2, PTPRJ, NFATC2, IGHA2, GPR32, ABL1, IGHV3-11, FYB1, PLCL2, RC3H1, IGLC7, STAP1, MALT1, IGHV3-66, IGHV3-73, PRKACG, MUC12, BCL2, STOML2, BTK, ELMO1, MEF2C, RAF1, PTK2, IGHV3-13, LGALS3, PDE4B, IGHV1-18, PTPRC, WIPF1, BTNL8, GATA3, IGLV2-14, GPR32P1, IGLL5, FCRL3, IGHV1OR15-1, IGLV1-47, TAB1, IGHV3-72, IGLV3-21, GPS2, BRK1, ABI1, IGHV1-69, SLA2, BTNL10, CD38, BCL10, NCKAP1L, PIK3CB, MAPK3, MYO10, GRAP2, IGHV3-74, IGHV6-1, IGHV3-64, NCK1, ARPC1A, THEMIS2, KLRC4-KLRK1, IGLV1-44, IGLC3, IGHA1, LILRB4, CD300A, IGHG1, IGLV6-57, SRC, CD226, IGHV4-34, STK11, WIPF3, BAX, MUC20, IGHV3-20, IGHV1-24, MUC1, TEC, FCGR3A, IGHV2-5, ELF1, NCKAP1, CD19, PRKCQ, FYN, LIMK1, RFTN1, NR4A3, BRAF, IGHV3-23, EIF2B5 | 100 |
| 16 | GO:0050864 | regulation of B cell activation | IGLL1, IGHV3-33, IGHG4, IGLC6, FOXP3, IGHV4-28, IGHV4-39, IGHV5-51, IGHV3-7, MLH1, IGHV1-45, IGHE, TNFSF4, TRBC2, PCID2, IGHV3-48, CTLA4, IGHG2, IGHD, NFAM1, IGHV3-43, INHBA, BCL6, IGHV1-58, PAWR, CARD11, IGHV2-70, IGHM, TNFSF13, FCGR2B, AHR, TNFRSF13B, IL7, MAD2L2, SLC39A10, IGHV3-49, SHLD2, IGHV4-61, THOC1, IL4, INPP5D, IGHV4-59, MNDA, IL6, IGHV3-30, GPR183, RIF1, PPP2R3C, IGLC1, APLF, IGHV3-23, BAD, IGHV1-3, TNFRSF13C, IGHV4-4, IGHV2-26, IRS2, PAXIP1, IGHV3-15, CD28, IGHV3-21, IGHV3-53, IL5, MIF, IGHG3, IGLC2, NFATC2, IGHA2, IL2, IL10, IGHV3-11, RC3H1, IGLC7, IGHV3-66, IGHV3-73, TNIP2, BCL2, ZFP36L2, CASP3, BTK, IL21, MEF2C, ATP11C, IGHV3-13, IGHV1-18, PTPRC, IGLL5, FCRL3, IGHV1OR15-1, IGHV3-72, ATAD5, NSD2, MSH6, SASH3, IGHV1-69, CD38, TNFRSF4, NCKAP1L, WNT3A, IGHV3-74, IGHV6-1, BST1, IGHV3-64, MSH2, NDFIP1, IGLC3, IGHA1, MIR17HG, XBP1, CD300A, IGHG1, IGHV4-34, IL13, PKN1, CLCF1, TBC1D10C, TNFSF13B, IGHV3-20, IGHV1-24, SFRP1, IGHV2-5, PELI1, TICAM1, SLA2, ZFP36L1, TFRC, TIRAP | 100 |
| 16 | GO:0051249 | regulation of lymphocyte activation | CD83, TESPA1, IGLL1, SHH, IGHV3-33, EGR3, CD40LG, IRF1, GAS6, IGHG4, IGLC6, FOXP3, PAG1, SIRPB1, CR1, CSK, IRF4, TNFSF11, IGHV4-28, IGHV4-39, TMIGD2, DUSP22, IGHV5-51, IGHV3-7, TARM1, SOCS5, PAK2, EFNB2, AP3D1, MLH1, IGHV1-45, IGHE, IFNB1, TRBC2, CD1D, TNFSF4, PCID2, IGHV3-48, CTLA4, PTPN2, IGHG2, IGHD, NFAM1, FBXO7, CAV1, DOCK8, IGHV3-43, TNFRSF14, CLECL1, DNAJA3, INHBA, BCL6, IGHV1-58, PAWR, CARD11, IDO1, ANXA1, BMP4, HMGB1, IGHV2-70, IGHM, TNFSF13, FCGR2B, AHR, TNFRSF13B, GRB2, IL7, TAC1, XCL1, VCAM1, SOCS6, PGLYRP2, MARCH7, ICOS, MAD2L2, SPN, SLC39A10, IGHV3-49, MAPK8IP1, SHLD2, PYCARD, CD274, ITPKB, MAD1L1, CD276, CD47, PAK3, IGHV4-61, DPP4, THOC1, PIBF1, IL4, INPP5D, IGHV4-59, KLRK1, MNDA, LEP, MIR30B, PDE5A, SFTPD, MIR21, PLA2G2F, IL6, IGHV3-30, GLI3, GPR183, RIF1, SART1, PPP2R3C, IL6ST, FADD, IGLC1, APLF, MERTK, IGHV3-23, BAD, FANCA, PDPK1, IGHV1-3, FLT3LG, TNFRSF13C, FGF10, IGHV4-4, FANCD2, ADAM8, IFNA2, RIPK2, RHBDD3, IGHV2-26, NLRP3, IRS2, PAXIP1, DUSP3, HSPD1, CBFB, SIRPA, SOX13, HHLA2, RAC1, GNRH1, IGHV3-15, CD28, IGHV3-21, LAMP1, YES1, IL20RB, CORO1A, MAP3K8, IGHV3-53, PDCD1LG2, IL5, GSN, LAT, IL23R, NRARP, LILRB1, MIF, RC3H2, GPAM, IGHG3, IGLC2, CD46, NFATC2, IGHA2, IL2, IL10, IGHV3-11, EBI3, ABL1, ZP4, HES1, NKAP, RC3H1, IL27, IGLC7, CD55, MALT1, IGHV3-66, IGHV3-73, TNIP2, LGALS1, PRKCZ, BCL2, ZFP36L2, CASP3, BTK, ZC3H8, IL21, MEF2C, ATP11C, AP1G1, IL2RA, PLA2G2D, IGHV3-13, LGALS3, IGHV1-18, PTPRC, GATA3, HSPH1, SPTA1, IL1RL2, SIT1, IGLL5, FCRL3, IGHV1OR15-1, CD86, IGHV3-72, HLX, ZEB1, ATAD5, NSD2, MSH6, SASH3, IGHV1-69, SLA2, EPO, CD38, BCL10, TNFRSF4, RAC2, TNFSF18, NCKAP1L, WNT3A, IL12B, IL12A, GRAP2, EFNB3, IGHV3-74, IGHV6-1, TNFRSF18, SOCS1, BST1, IGHV3-64, MSH2, NCK1, TWSG1, RASAL3, IL15, NDFIP1, IL4R, KLRC4-KLRK1, AKT1, CD80, ITCH, IGLC3, IGHA1, MIR17HG, CCDC88B, PDCD1, XBP1, LILRB4, GPNMB, CD300A, IGHG1, SRC, DUSP10, DLG1, SOD1, IGHV4-34, PRDM1, PKN1, CLCF1, TBC1D10C, TNFSF13B, TIGIT, TMEM131L, IGHV3-20, IGHV1-24, CEBPB, LILRB2, SFRP1, IGHV2-5, SIRPG, PRKCQ, LAX1, FYN, BTLA, PELI1, TICAM1, BRAF, IL13, ZFP36L1, PRELID1, TFRC, TIRAP | 100 |
| 16 | GO:0002449 | lymphocyte mediated immunity | CTSH, IGLL1, IGHV3-33, CD40LG, IGHG4, TUBB4B, IGLV2-23, IGLC6, FOXP3, CR1, NCR1, DENND1B, IGHV4-28, IGHV4-39, C8G, IGHV5-51, IGHV3-7, DUSP22, IGLV3-25, EXO1, MLH1, IGHV1-45, IGHE, IFNB1, CD1D, TRBC2, TNFSF4, TSTA3, IGHV3-48, IGLV2-8, SCART1, IGHG2, IGHD, IGLV2-11, IGHV3-43, BCL6, IL13RA2, IGHV1-58, TRAF2, APCS, SH2D1A, C1QBP, IGLV3-1, HMGB1, IGHV2-70, IGHM, FCGR2B, TRPM4, CRK, TNFSF13, C6, C4BPB, PPP3CB, XCL1, MAD2L2, IGLV1-51, IGHV3-49, CRP, SHLD2, FCER1G, IGHV4-61, THOC1, IL4, INPP5D, IGHV4-59, KLRK1, LEP, RAB27A, C4BPA, IL6, IGHV3-30, C1QC, RIF1, KLRF2, IGLC1, FADD, APLF, CLEC12B, IGHV1-3, LIG4, IGHV4-4, GZMM, IGLV7-43, IFNA2, IGLV3-27, C1QB, IGHV2-26, NLRP3, IGLV3-19, PAXIP1, SERPINB4, CR2, MYO1G, HSPD1, IGHV3-15, IGLV1-40, CD28, C9, IGHV3-21, LAMP1, CORO1A, IL20RB, IGHV3-53, WAS, IL23R, LILRB1, IGHG3, CD1A, IGLC2, CD46, IGHA2, IL2, MASP2, IGHV3-11, CD1C, SUSD4, IL10, IGLC7, CD55, MALT1, IGHV3-66, IGHV3-73, PRKCZ, PIK3R6, LYST, BTK, CD1B, IL1R1, IL21, AP1G1, IGHV3-13, GAPT, IGHV1-18, PTPRC, SLAMF6, KIR3DL1, GATA3, IGLV2-14, CD1E, EMP2, IGLL5, KLRD1, IGHV1OR15-1, CD96, IGLV1-47, IGHV3-72, ATAD5, NSD2, IGLV3-21, MSH6, SASH3, IGHV1-69, SLA2, CCR6, RSAD2, NBN, BCL10, SERPINB9, IL12B, IL12A, RNF168, KDELR1, IGHV3-74, IGHV6-1, IGHV3-64, MSH2, KDM5D, SLAMF7, IL18RAP, NDFIP1, IL4R, KLRC4-KLRK1, IGLV1-44, IGLC3, IGHA1, FZD5, IL18R1, C1QA, IGHG1, HPRT1, IGLV6-57, DLG1, CD226, C5, IL31RA, KLRC2, IGHV4-34, C7, CLCF1, CLEC2A, IGHV3-20, IGHV1-24, IGHV2-5, RFTN1, CLU, IGHV3-23, TFRC | 100 |
| 16 | GO:0002433 | immune response-regulating cell surface receptor signaling pathway involved in phagocytosis | IGHV3-33, IGHG4, IGLC6, IGLV2-23, DOCK1, IGHV4-39, VAV2, IGHV3-7, PLCG2, IGLV3-25, WASL, IGHV3-48, IGLV2-8, IGHG2, IGLV2-11, ACTB, CD247, PLA2G6, PRKCE, IGLV3-1, IGHV2-70, FCGR2B, MAPK1, CRK, WASF2, GRB2, ARPC5, IGLV1-51, ARPC4, CD47, RAPGEF1, RAP1A, IGHV4-59, CYFIP1, IGHV3-30, HSP90AA1, MYO1C, ARPC1B, IGLC1, IGLV7-43, IGLV3-27, IGLV3-19, MYO1G, RAC1, IGLV1-40, FCGR1A, YES1, IGHV3-53, WAS, FGR, HCK, ACTR2, IGHG3, FCGR2A, PTPRJ, ABL1, IGHV3-11, IGLC7, ELMO1, PTK2, IGHV3-13, PTPRC, WIPF1, IGLV2-14, IGHV1OR15-1, IGLV1-47, IGLV3-21, BRK1, ABI1, IGHV1-69, NCKAP1L, PIK3CB, MAPK3, MYO10, NCK1, ARPC1A, IGLV1-44, IGHG1, IGLV6-57, SRC, IGHV4-34, WIPF3, FCGR3A, IGHV2-5, NCKAP1, FYN, LIMK1, IGHV3-23 | 100 |
| 16 | GO:0002696 | positive regulation of leukocyte activation | CD83, TESPA1, IGLL1, SHH, IGHV3-33, EGR3, CD40LG, SIRPB1, GAS6, IGHG4, IGLC6, FOXP3, CSK, TNFSF11, IGHV4-28, IGHV4-39, TMIGD2, IGHV5-51, IGHV3-7, PAK2, EFNB2, AP3D1, MLH1, IGHV1-45, IGHE, CD1D, TRBC2, TNFSF4, PCID2, IGHV3-48, CTLA4, IGHG2, IGHD, CAV1, DOCK8, IGHV3-43, TNFRSF14, CLECL1, DNAJA3, BCL6, IGHV1-58, CARD11, ANXA1, HMGB1, IGHV2-70, IGHM, TNFSF13, GRB2, IL7, TAC1, XCL1, VCAM1, PTAFR, ICOS, MAD2L2, SLC39A10, IGHV3-49, SHLD2, PYCARD, CD274, ITPKB, FCER1G, CD276, CD47, PAK3, IGHV4-61, DPP4, IL4, INPP5D, IGHV4-59, KLRK1, MIR30B, LEP, MIR21, SART1, IL6, IGHV3-30, GLI3, GPR183, RIF1, PPP2R3C, IL6ST, FADD, IGLC1, IGHV3-23, BAD, ITGB2, CRLF2, PDPK1, IGHV1-3, FLT3LG, TNFRSF13C, LBP, FGF10, IGHV4-4, ADAM8, RIPK2, IGHV2-26, NLRP3, IRS2, PAXIP1, IL1RL1, HSPD1, CBFB, SIRPA, HHLA2, RAC1, STXBP1, IGHV3-15, CD28, IGHV3-21, LAMP1, YES1, CORO1A, MAP3K8, IGHV3-53, PDCD1LG2, IL5, FGR, IL23R, ADORA2B, LILRB1, MIF, GPAM, IGHG3, IGLC2, CD46, NFATC2, IGHA2, IL2, IL10, IGHV3-11, EBI3, SOCS5, ZP4, HES1, NKAP, IGLC7, CD55, STAP1, MALT1, IGHV3-66, IGHV3-73, TNIP2, LGALS1, PRKCZ, BCL2, BTK, IL21, MEF2C, ATP11C, AP1G1, IL2RA, IGHV3-13, IGHV1-18, PTPRC, GATA3, HSPH1, SPTA1, IL1RL2, IGLL5, FCRL3, IGHV1OR15-1, CD86, IGHV3-72, HLX, ATAD5, NSD2, MSH6, NPPA, SASH3, IGHV1-69, EPO, CD38, BCL10, TNFRSF4, NCKAP1L, WNT3A, IL12B, IL12A, GRAP2, EFNB3, IGHV3-74, IGHV6-1, SOCS1, BST1, IGHV3-64, MSH2, NCK1, RASAL3, ITGAM, IL15, IL4R, KLRC4-KLRK1, AKT1, CD80, IGLC3, IGHA1, CCDC88B, PDCD1, XBP1, LILRB4, IGHG1, SRC, DUSP10, CD226, IGHV4-34, IL33, CLCF1, STX4, TNFSF13B, IGHV3-20, IGHV1-24, LILRB2, F2RL1, IGHV2-5, SIRPG, PRKCQ, NR4A3, FYN, BTLA, PELI1, TICAM1, IL13, TFRC, TIRAP | 100 |
| 16 | GO:0006956 | complement activation | IGLL1, IGHV3-33, IGHG4, IGLC6, IGLV2-23, CR1, IGHV4-28, IGHV4-39, C8G, IGHV5-51, IGHV3-7, KRT1, IGLV3-25, CFHR4, IGHV1-45, IGHE, TRBC2, CFHR5, IGHV3-48, IGLV2-8, IGHG2, IGHD, IGLV2-11, IGHV3-43, IGHV1-58, APCS, C1QBP, IGLV3-1, CFHR2, IGHV2-70, IGHM, C6, C4BPB, IGLV1-51, CPB2, IGHV3-49, SCARA3, CRP, IGHV4-61, IGHV4-59, CFHR1, C4BPA, FCN1, C1QC, IGHV3-30, IGLC1, IGHV1-3, IGHV4-4, IGLV7-43, IGLV3-27, C1QB, IGHV2-26, IGLV3-19, CR2, IGHV3-15, IGLV1-40, C9, CFH, IGHV3-21, RGCC, IGHV3-53, CFD, CPN2, FCN2, IGHG3, IGLC2, CD46, IGHA2, MASP2, IGHV3-11, SUSD4, IGLC7, CD55, IGHV3-66, IGHV3-73, IGHV3-13, IGHV1-18, COL20A1, MASP1, IGLV2-14, IGLL5, IGHV1OR15-1, IGLV1-47, FCN3, IGHV3-72, A2M, IGLV3-21, IGHV1-69, IGHV3-74, IGHV6-1, IGHV3-64, COLEC10, VSIG4, IGLV1-44, IGLC3, IGHA1, C1QA, IGHG1, IGLV6-57, C5, IGHV4-34, C7, IGHV3-20, IGHV1-24, IGHV2-5, CD19, CLU, IGHV3-23 | 100 |
| 16 | GO:0051251 | positive regulation of lymphocyte activation | CD83, TESPA1, IGLL1, SHH, IGHV3-33, EGR3, CD40LG, SIRPB1, GAS6, IGHG4, IGLC6, FOXP3, CSK, TNFSF11, IGHV4-28, IGHV4-39, TMIGD2, IGHV5-51, IGHV3-7, PAK2, EFNB2, AP3D1, MLH1, IGHV1-45, IGHE, CD1D, TRBC2, TNFSF4, PCID2, IGHV3-48, CTLA4, IGHG2, IGHD, CAV1, DOCK8, IGHV3-43, TNFRSF14, CLECL1, DNAJA3, BCL6, IGHV1-58, CARD11, ANXA1, HMGB1, IGHV2-70, IGHM, TNFSF13, GRB2, IL7, TAC1, XCL1, VCAM1, ICOS, MAD2L2, SLC39A10, IGHV3-49, SHLD2, PYCARD, CD274, ITPKB, CD276, CD47, PAK3, IGHV4-61, DPP4, IL4, INPP5D, IGHV4-59, KLRK1, MIR30B, LEP, MIR21, SART1, IL6, IGHV3-30, GLI3, GPR183, RIF1, PPP2R3C, IL6ST, FADD, IGLC1, IGHV3-23, BAD, PDPK1, IGHV1-3, FLT3LG, TNFRSF13C, FGF10, IGHV4-4, ADAM8, RIPK2, IGHV2-26, NLRP3, IRS2, PAXIP1, HSPD1, CBFB, SIRPA, HHLA2, RAC1, IGHV3-15, CD28, IGHV3-21, LAMP1, YES1, CORO1A, MAP3K8, IGHV3-53, PDCD1LG2, IL5, IL23R, LILRB1, MIF, GPAM, IGHG3, IGLC2, NFATC2, CD46, IGHA2, IL2, EBI3, IGHV3-11, SOCS5, ZP4, HES1, NKAP, IGLC7, CD55, MALT1, IGHV3-66, IGHV3-73, TNIP2, LGALS1, PRKCZ, BCL2, BTK, IL21, MEF2C, ATP11C, AP1G1, IL2RA, IGHV3-13, IGHV1-18, PTPRC, GATA3, HSPH1, SPTA1, IL1RL2, IGLL5, FCRL3, IGHV1OR15-1, CD86, IGHV3-72, HLX, ATAD5, NSD2, MSH6, SASH3, IGHV1-69, EPO, CD38, BCL10, TNFRSF4, NCKAP1L, WNT3A, IL12B, IL12A, GRAP2, EFNB3, IGHV3-74, IGHV6-1, SOCS1, BST1, IGHV3-64, MSH2, NCK1, RASAL3, IL15, IL4R, KLRC4-KLRK1, AKT1, CD80, IGLC3, IGHA1, CCDC88B, PDCD1, XBP1, LILRB4, IGHG1, SRC, DUSP10, IGHV4-34, CLCF1, TNFSF13B, IGHV3-20, IGHV1-24, LILRB2, IGHV2-5, SIRPG, PRKCQ, FYN, BTLA, PELI1, TICAM1, IL13, TFRC, TIRAP | 100 |
| 16 | GO:0002768 | immune response-regulating cell surface receptor signaling pathway | TESPA1, IGLL1, IGHV3-33, CARD9, FPR1, RAB29, IGHG4, IGLC6, IGLV2-23, PVRIG, PAG1, CR1, CSK, KLHL6, FOXP3, DOCK1, IGHV4-28, IGHV4-39, DENND1B, DUSP22, IGHV5-51, IGHV3-7, VAV2, PLCG2, BLK, IGLV3-25, PAK2, IKBKG, IGHV1-45, IGHE, TRBC2, WASL, IGHV3-48, CTLA4, HRAS, IGLV2-8, PTPN2, CLEC10A, IGHG2, IGHD, NFAM1, IGLV2-11, LILRA2, ACTB, LCP2, RBCK1, MUC7, IGHV3-43, RELA, CD247, IGHV1-58, MUC17, FPR2, CARD11, PLA2G6, PRKCE, IGLV3-1, PAWR, IGHV2-70, IGHM, FCGR2B, MAPK1, CRK, WASF2, GCSAM, GRB2, ELF2, ARPC5, PPP3CB, PHPT1, SLC39A10, LAT2, IGLV1-51, IGHV3-49, PDE4D, ARPC4, MAPK10, CD276, CD47, FCER1G, PRKACB, PAK3, RAPGEF1, IGHV4-61, RAP1A, INPP5D, TXK, IGHV4-59, CREBBP, MNDA, LIME1, CMTM3, KLRK1, TRAT1, BTNL9, PIK3CD, LILRA4, CYFIP1, IKBKB, FCN1, FPR3, IGHV3-30, HSP90AA1, MYO1C, ARPC1B, IGLC1, PAX5, CLEC12B, PPP3R1, PDPK1, IGHV1-3, IGHV4-4, IGLV7-43, EP300, IGLV3-27, RIPK2, IGHV2-26, ERMAP, IGLV3-19, SH2B2, DUSP3, CR2, MYO1G, CBFB, HHLA2, RAC1, IGHV3-15, IGLV1-40, MUCL1, MUC4, PLEKHA1, CD28, IGHV3-21, FCGR1A, MUC3A, MAP3K1, EZR, YES1, IGHV3-53, WAS, CACNA1F, FGR, BTNL3, PLSCR1, LAT, HCK, GCSAML, RC3H2, LILRB1, ACTR2, IGHG3, FCGR2A, IGLC2, PTPRJ, NFATC2, IGHA2, GPR32, ABL1, IGHV3-11, FYB1, PLCL2, RC3H1, IGLC7, STAP1, MALT1, IGHV3-66, IGHV3-73, PRKACG, MUC12, BCL2, STOML2, BTK, ELMO1, MEF2C, RAF1, IGHV3-13, LGALS3, PDE4B, IGHV1-18, PTPRC, WIPF1, CD200R1, BTNL8, GATA3, IGLV2-14, GPR32P1, IGLL5, FCRL3, IGHV1OR15-1, IGLV1-47, TAB1, IGHV3-72, IGLV3-21, GPS2, BRK1, ABI1, IGHV1-69, SLA2, BTNL10, CD38, BCL10, FCER1A, NCKAP1L, PIK3CB, MAPK3, MYO10, GRAP2, IGHV3-74, IGHV6-1, IGHV3-64, NCK1, ARPC1A, THEMIS2, KLRC4-KLRK1, IGLV1-44, IGLC3, IGHA1, LILRB4, NFATC1, CD300A, IGHG1, IGLV6-57, KIR2DL1, CD226, SRC, PIGR, IGHV4-34, STK11, WIPF3, BAX, MUC20, IGHV3-20, IGHV1-24, MUC1, TEC, LILRB2, FCGR3A, IGHV2-5, ELF1, NCKAP1, CD19, PRKCQ, FYN, LIMK1, RFTN1, BTLA, BRAF, MAPK9, NR4A3, IGHV3-23, EIF2B5 | 100 |
| 16 | GO:0010324 | membrane invagination | SYT11, IGLL1, IGHV3-33, IGHG4, IGLC6, MSR1, SH3BP1, DOCK1, IGHV4-28, IGHV4-39, IGHV5-51, IGHV3-7, IGHV1-45, IGHE, TRBC2, IGHV3-48, IGHG2, IGHD, IGHV3-43, IGHV1-58, IGHV2-70, IGHM, FCGR2B, PPARG, ALKBH4, IGHV3-49, FCER1G, IGHV4-61, IGHV4-59, CD36, IGHV3-30, IGLC1, ITGB2, IGHV1-3, IGHV4-4, IGHV2-26, ARHGAP12, IGHV3-15, IGHV3-21, FCGR1A, RHOBTB2, IGHV3-53, GSN, IGHG3, MEGF10, IGLC2, IGHA2, SPIRE2, IGHV3-11, IGLC7, STAP1, IGHV3-66, IGHV3-73, ELMO1, IGHV3-13, IGHV1-18, IGLL5, IGHV1OR15-1, IGHV3-72, ARF1, IGHV1-69, NCKAP1L, ADGRB1, ARHGAP25, MYH9, IGHV3-74, IGHV6-1, IGHV3-64, ITGAM, ABCA7, MFGE8, SNX9, IGLC3, IGHA1, CD300A, IGHG1, SPIRE1, IGHV4-34, SNX33, GULP1, IGHV3-20, IGHV1-24, XKR8, F2RL1, SMURF1, IGHV2-5, IGHV3-23 | 100 |
| 16 | GO:0002673 | regulation of acute inflammatory response | IGHV3-33, PARK7, IGHG4, IGLC6, IGLV2-23, CR1, TNFSF11, IGHV4-39, C8G, IGHV3-7, CFHR4, IGLV3-25, MIR92A2, GSTP1, CFHR5, IGHV3-48, IGLV2-8, IGHG2, IGLV2-11, PIK3CG, APCS, C1QBP, IGLV3-1, CFHR2, OSMR, IGHV2-70, FCGR2B, C6, TAC1, C4BPB, PPARG, IGLV1-51, CPB2, FCER1G, IGHV4-59, CFHR1, C4BPA, KLKB1, TNFRSF11A, IGHV3-30, IL6, C1QC, IL6ST, IGLC1, F12, ADAM8, IGLV7-43, C1QB, IGLV3-27, RHBDD3, ADORA1, NLRP3, IGLV3-19, CR2, IGLV1-40, C9, CFH, IL20RB, IGHV3-53, SELENOS, CPN2, MIR92A1, IGHG3, CD46, SUSD4, IGHV3-11, IGLC7, CD55, OSM, CREB3L3, DNASE1, BTK, ADCYAP1, IGHV3-13, IGLV2-14, IGHV1OR15-1, IGLV1-47, DNASE1L3, A2M, IGLV3-21, IGHV1-69, EDNRB, ASH1L, PTGS2, PTGER3, IGLV1-44, C1QA, IGHG1, IGLV6-57, C5, IGHV4-34, C7, IGHV2-5, CD19, CLU, IGHV3-23 | 100 |
| 16 | GO:0002920 | regulation of humoral immune response | IGHV3-53, IGLV3-21, IGHV3-33, CXCL13, IGHV1-69, IGHG4, IGLC6, IGLV2-23, CPN2, CR1, ACOD1, IGHV4-59, IGHV4-39, CFHR1, C8G, IGHG3, IGHV3-7, CD46, C4BPA, CFHR4, IGLV3-25, SUSD4, IGHV3-11, ZP4, IGHV3-30, C1QC, PPP2R3C, CFHR5, IGLC7, CD55, IGHV3-48, IGLC1, IGLV2-8, IGLV1-44, IGHG2, IGLV2-11, C1QA, IGHG1, IGLV6-57, IGLV7-43, C5, IGHV3-13, IGLV3-27, IGHV4-34, PTPRC, IGLV3-1, CFHR2, IGLV3-19, IGHV2-70, C1QB, FCGR2B, C7, C1QBP, IGLV2-14, CR2, C6, KLK5, C4BPB, IGHV2-5, IGHV1OR15-1, KLK7, IGLV1-47, IGLV1-40, CD19, C9, CFH, CLU, IGLV1-51, IGHV3-23, CPB2, A2M | 100 |
| 16 | GO:2000257 | regulation of protein activation cascade | IGHV3-53, IGLV3-21, SERPINC1, IGHV3-33, IGHV1-69, IGHG4, IGLC6, IGLV2-23, CPN2, CR1, IGHV4-59, IGHV4-39, IGHG3, CFHR1, C8G, IGHV3-7, CD46, C4BPA, CFHR4, IGLV3-25, SUSD4, IGHV3-11, C1QC, IGHV3-30, CFHR5, IGLC7, CD55, IGHV3-48, IGLC1, IGLV2-8, IGLV1-44, IGHG2, IGLV2-11, C1QA, IGHG1, IGLV6-57, IGLV7-43, C5, IGHV3-13, IGLV3-27, IGHV4-34, IGLV3-1, C1QB, CFHR2, IGLV3-19, IGHV2-70, C7, C1QBP, IGLV2-14, CR2, C6, C4BPB, IGHV2-5, IGHV1OR15-1, IGLV1-47, IGLV1-40, CD19, C9, CFH, CLU, IGLV1-51, IGHV3-23, CPB2, A2M | 100 |
| 16 | GO:0006898 | receptor-mediated endocytosis | SYT11, AMN, AP1S1, CLTCL1, IGHV3-33, CACNG3, CLTC, IGLC6, IGLV2-23, ACHE, AP2A1, MSR1, IGHV4-39, OPHN1, GPR107, IGHV3-7, PLCG2, IGLV3-25, EFNB2, CANX, CACNG5, WASL, EPS15, IGHV3-48, CALCRL, IGLV2-8, SCART1, IGLV2-11, GSG1L, SCYL2, CAV1, LRP1B, CAV3, ILDR1, CD63, VLDLR, SFRP4, RAMP1, LRP2, IGLV3-1, CLEC9A, IGHV2-70, FCGR2B, NEDD4, CACNG8, GRB2, IGF2R, MIR199A2, HTR1B, SCRIB, CLTB, IGLV1-51, SCARA3, FCER1G, SYNJ1, DAB2, CNTN2, IGHV4-59, CD36, SFTPD, SGIP1, IGHV3-30, HSP90AA1, SCARF1, IGLC1, MIR185, JCHAIN, GRK4, ITGB2, DBNL, ASGR2, FMR1, COLEC12, ANXA2P2, NECAB2, IGLV7-43, SCGB3A2, IGLV3-27, PLA2R1, IGLV3-19, DNM1P34, LRRTM2, CD5L, RAC1, SUSD2, IGLV1-40, HHIPL1, FCGR1A, EZR, IGHV3-53, CLTA, SMAP1, LILRB1, MKLN1, CACNG2, SH3GL2, MEGF10, TFR2, SCARA5, RABEPK, APOL1, IGHA2, TBC1D5, IGHV3-11, HIP1, PICK1, CD207, CALR, IGLC7, MICALL1, CD14, DNM1, HTR2B, DLL1, SELE, ITGA4, AAK1, CACNG4, PCSK9, IGHV3-13, MASP1, IGLV2-14, LOXL2, HSPH1, TINAGL1, CLN3, RAB31, CAV2, APOBR, IGHV1OR15-1, LDLR, IGLV1-47, PDLIM7, DMBT1, DNM3, PRG4, IGLV3-21, ASGR1, IGHV1-69, TNK2, ENPP2, GAK, LRPAP1, PIK3CB, ATAD1, CALY, PIP5K1C, CUBN, SERPINE1, SNCA, CACNG7, MRC1, IGLV1-44, ACKR3, SNX9, IGHA1, IGLV6-57, RAMP3, IGHV4-34, RAB21, MAGI2, PRSS12, DRD3, LRP6, IGHV2-5, CLU, DLG4, ITGB1, IGHV3-23, AP2M1, TFRC | 100 |
| 16 | GO:0050871 | positive regulation of B cell activation | IGLL1, IGHV3-33, IGHG4, IGLC6, IGHV4-28, IGHV4-39, IGHV5-51, IGHV3-7, MLH1, IGHV1-45, IGHE, TNFSF4, TRBC2, PCID2, IGHV3-48, IGHG2, IGHD, IGHV3-43, BCL6, IGHV1-58, CARD11, IGHV2-70, IGHM, TNFSF13, IL7, MAD2L2, SLC39A10, IGHV3-49, SHLD2, IGHV4-61, IL4, INPP5D, IGHV4-59, IL6, IGHV3-30, GPR183, RIF1, PPP2R3C, IGLC1, IGHV3-23, BAD, IGHV1-3, TNFRSF13C, IGHV4-4, IGHV2-26, IRS2, PAXIP1, IGHV3-15, CD28, IGHV3-21, IGHV3-53, IL5, MIF, IGHG3, IGLC2, NFATC2, IGHA2, IL2, IGHV3-11, IGLC7, IGHV3-66, IGHV3-73, TNIP2, BCL2, BTK, IL21, MEF2C, ATP11C, IGHV3-13, IGHV1-18, PTPRC, IGLL5, FCRL3, IGHV1OR15-1, IGHV3-72, ATAD5, NSD2, MSH6, SASH3, IGHV1-69, CD38, TNFRSF4, NCKAP1L, WNT3A, IGHV3-74, IGHV6-1, BST1, IGHV3-64, MSH2, IGLC3, IGHA1, XBP1, IGHG1, IGHV4-34, CLCF1, TNFSF13B, IGHV3-20, IGHV1-24, IGHV2-5, PELI1, TICAM1, IL13, TFRC, TIRAP | 100 |
| 16 | GO:0002431 | Fc receptor mediated stimulatory signaling pathway | IGHV3-33, IGHG4, IGLC6, IGLV2-23, CSK, DOCK1, IGHV4-39, VAV2, IGHV3-7, PLCG2, IGLV3-25, WASL, IGHV3-48, IGLV2-8, IGHG2, IGLV2-11, ACTB, CD247, PLA2G6, PRKCE, IGLV3-1, IGHV2-70, FCGR2B, MAPK1, CRK, WASF2, GRB2, ARPC5, IGLV1-51, ARPC4, CD47, FCER1G, RAPGEF1, RAP1A, IGHV4-59, CYFIP1, IGHV3-30, HSP90AA1, MYO1C, ARPC1B, IGLC1, IGLV7-43, IGLV3-27, IGLV3-19, MYO1G, RAC1, IGLV1-40, FCGR1A, YES1, IGHV3-53, WAS, FGR, PLSCR1, HCK, ACTR2, IGHG3, FCGR2A, PTPRJ, ABL1, IGHV3-11, IGLC7, ELMO1, PTK2, IGHV3-13, PTPRC, WIPF1, IGLV2-14, IGHV1OR15-1, IGLV1-47, IGLV3-21, BRK1, ABI1, IGHV1-69, NCKAP1L, PIK3CB, MAPK3, MYO10, NCK1, ARPC1A, IGLV1-44, IGHG1, IGLV6-57, SRC, CD226, IGHV4-34, WIPF3, FCGR3A, IGHV2-5, NCKAP1, NR4A3, FYN, LIMK1, IGHV3-23 | 100 |
| 16 | GO:0006911 | phagocytosis, engulfment | IGHV3-53, FCER1G, IGLL1, GSN, IGHV3-33, IGHV1-69, IGHV4-61, IGHG4, IGLC6, NCKAP1L, IGHV4-59, SH3BP1, DOCK1, IGHV4-28, IGHV4-39, MSR1, IGHG3, IGHV5-51, IGHV3-7, MEGF10, CD36, ARHGAP25, IGLC2, MYH9, IGHA2, IGHV3-74, IGHV6-1, IGHV3-11, IGHV3-30, IGHE, IGHV1-45, IGHV3-64, TRBC2, ITGAM, IGLC7, IGLC1, IGHV3-48, STAP1, ABCA7, IGHV3-66, IGHV3-73, MFGE8, ADGRB1, IGHG2, IGHD, ITGB2, IGLC3, IGHA1, IGHV1-3, IGHV3-43, CD300A, IGHG1, IGHV4-4, ELMO1, IGHV1-58, IGHV3-13, IGHV4-34, IGHV1-18, IGHV2-26, ARHGAP12, GULP1, IGHV2-70, IGHM, FCGR2B, IGHV3-20, IGHV1-24, XKR8, F2RL1, IGLL5, IGHV2-5, PPARG, IGHV1OR15-1, IGHV3-15, IGHV3-21, IGHV3-72, FCGR1A, IGHV3-23, RHOBTB2, IGHV3-49 | 100 |
| 16 | GO:0002460 | adaptive immune response based on somatic recombination of immune receptors built from immunoglobulin superfamily domains | CTSH, IGLL1, IGHV3-33, CXCL13, CD40LG, IGHG4, IGLC6, IGLV2-23, FOXP3, CR1, KLHL6, IRF4, DENND1B, IGHV4-28, IGHV4-39, C8G, IGHV5-51, IGHV3-7, DUSP22, IGLV3-25, EXO1, MLH1, IGHV1-45, IGHE, IFNB1, CD1D, TRBC2, TNFSF4, TSTA3, IGHV3-48, HRAS, IGLV2-8, SCART1, IGHG2, IGHD, IGLV2-11, IGHV3-43, BCL6, IL13RA2, IGHV1-58, TRAF2, APCS, ANXA1, IGLV3-1, HMGB1, C1QBP, IGHV2-70, IGHM, FCGR2B, TRPM4, TNFSF13, C6, C4BPB, PPP3CB, XCL1, MAD2L2, SPN, IGLV1-51, IGHV3-49, CRP, SHLD2, CD274, FCER1G, IGHV4-61, THOC1, IL4, INPP5D, IGHV4-59, RAB27A, C4BPA, MIR21, LY9, IL6, IGHV3-30, C1QC, RIF1, FADD, IGLC1, APLF, IGHV1-3, TNFRSF13C, LIG4, IGHV4-4, GZMM, IGLV7-43, IFNA2, IGLV3-27, RIPK2, IGHV2-26, NLRP3, IGLV3-19, C1QB, PAXIP1, IL1RL1, MTOR, CR2, MYO1G, HSPD1, IGHV3-15, IGLV1-40, SEMA4A, CD28, C9, IGHV3-21, IL20RB, IGHV3-53, WAS, IL23R, RC3H2, LILRB1, IGHG3, CD1A, IGLC2, CD46, SOCS5, IGHA2, IL2, MASP2, IGHV3-11, CD1C, SUSD4, IL10, EBI3, RC3H1, IL27, IGLC7, CD55, MALT1, IGHV3-66, IGHV3-73, PRKCZ, BTK, CD1B, IL1R1, MEF2C, IGHV3-13, GAPT, IGHV1-18, PTPRC, SLAMF6, GATA3, IGLV2-14, CD1E, EMP2, IGLL5, IGHV1OR15-1, IGLV1-47, IGHV3-72, HLX, ATAD5, NSD2, IGLV3-21, MSH6, SASH3, IGHV1-69, SLA2, CCR6, RSAD2, NBN, BCL10, IL12B, IL12A, RNF168, KDELR1, IGHV3-74, IGHV6-1, IGHV3-64, MSH2, KDM5D, IL18RAP, NDFIP1, IL4R, CD80, IGLV1-44, IGLC3, IGHA1, FZD5, IL18R1, C1QA, IGHG1, HPRT1, IGLV6-57, DLG1, CD226, C5, IL31RA, IGHV4-34, IL33, PKN1, C7, CLCF1, TNFSF13B, IGHV3-20, IGHV1-24, IGHV2-5, PRKCQ, RFTN1, CLU, IGHV3-23, TFRC | 100 |
| 16 | GO:0006959 | humoral immune response | CD83, IGLL1, IGHV3-33, CXCL13, ROMO1, IGHG4, IGLC6, IGLV2-23, CR1, IGHV4-28, IGHV4-39, IFNA5, C8G, IGHV5-51, IGHV3-7, DEFB127, DEFA3, KRT1, IGLV3-25, EXO1, SPON2, CHGA, CFHR4, IGHV1-45, IGHE, IFNB1, TRBC2, DEFB1, CFHR5, DEFA4, PRTN3, IGHV3-48, DEFB126, IGLV2-8, IGHG2, IGHD, IGLV2-11, MUC7, AZU1, IGHV3-43, IGHV1-58, DCD, APCS, SH2D1A, PLA2G6, C1QBP, IGLV3-1, IFNA14, KLK3, CFHR2, IGHV2-70, IGHM, FCGR2B, ITLN1, DEFB4A, C6, BPI, IL7, C4BPB, PGLYRP2, BPIFA1, IGLV1-51, HTN1, ST6GAL1, CPB2, IGHV3-49, SCARA3, CRP, IFNA6, MNX1, IFNA4, IFNA10, DEFA5, IGHV4-61, IFNA13, BPIFA2, IGHV4-59, CFHR1, C4BPA, SFTPD, FCN1, IFNE, HTN3, DEFA6, IGHV3-30, GPR183, IL6, C1QC, PPP2R3C, IGLC1, PAX5, YTHDF2, JCHAIN, HRG, IFNA16, IGHV1-3, IGHV4-4, IGLV7-43, IFNA2, IGLV3-27, C1QB, IGHV2-26, RPL39, IGLV3-19, FAM3A, KLK5, DEFA1, CR2, BPIFB2, DEFB118, IGHV3-15, IGLV1-40, CD28, C9, IFNA1, IGHV3-21, CFH, ACOD1, RGCC, IGHV3-53, CFD, CPN2, FCN2, PRSS3, IGHG3, FGA, LCN2, IGLC2, CD46, IGHA2, MASP2, TFE3, IGHV3-11, SUSD4, EBI3, ZP4, IGLC7, CD55, IGHV3-66, IGHV3-73, NOTCH1, DEFB103B, BCL2, DEFA1B, IFNA17, MEF2C, IGHV3-13, LEAP2, IGHV1-18, PTPRC, COL20A1, IFNA7, GATA3, MASP1, IGLV2-14, DEFB103A, IGLL5, TRAF3IP2, IGHV1OR15-1, KLK7, IGLV1-47, FCN3, DMBT1, IGHV3-72, IFNA21, A2M, IGLV3-21, IGHV1-69, CCR6, RNASE7, RARRES2, IGHV3-74, IGHV6-1, BST1, IGHV3-64, FGB, IFNK, COLEC10, VSIG4, IGLV1-44, RNASE6, IFNA8, IGLC3, IGHA1, PLA2G2A, PDCD1, C1QA, IGHG1, IGLV6-57, ELANE, C5, IGHV4-34, C7, RNASE3, IGHV3-20, IGHV1-24, IGHV2-5, BPIFB1, CD19, CLU, IFNW1, IGHV3-23 | 100 |
| 16 | GO:0070613 | regulation of protein processing | IGHV3-33, IGHG4, IGLC6, IGLV2-23, CR1, IGHV4-39, C8G, MELTF, IGHV3-7, IGLV3-25, CFHR4, PRSS37, CFHR5, IGHV3-48, IGLV2-8, IGHG2, IGLV2-11, USP17L2, NKD2, NOL3, C1QBP, IGLV3-1, CFHR2, IGHV2-70, C6, C4BPB, ACP4, IGLV1-51, CPB2, PRKACB, CNTN2, IGHV4-59, CFHR1, C4BPA, KLKB1, C1QC, IGHV3-30, IGLC1, SERPINF2, ANXA2P2, F12, ADAM8, IGLV7-43, IGLV3-27, C1QB, IGLV3-19, CR2, XIAP, IGLV1-40, C9, PLGRKT, CFH, TMEM59, IGHV3-53, GSN, CPN2, IGHG3, CD46, IGHV3-11, SUSD4, ENO1, IGLC7, CD55, TIMM17A, CARD8, IGHV3-13, IGLV2-14, CTSZ, IGHV1OR15-1, SERPINE2, IGLV1-47, ANGPTL8, A2M, IGLV3-21, IGHV1-69, MYH9, SERPINE1, NLRP7, BIRC7, IGLV1-44, BCL2L12, C1QA, IGHG1, IGLV6-57, SRC, C5, IGHV4-34, C7, IL1R2, IGHV2-5, CD19, CLU, TNP2, IGHV3-23, CCBE1, GLG1 | 100 |
| 16 | GO:0042742 | defense response to bacterium | SYT11, IGLL1, IGHV3-33, CXCL13, CARD9, ROMO1, IGHG4, IGLC6, IGHV4-28, IGHV4-39, IRF8, DEFA3, IGHV5-51, IGHV3-7, DEFB127, UNC13B, DEFB115, SPON2, CHGA, DEFB135, IGHV1-45, IGHE, TRBC2, DEFB1, DEFA4, IGHV3-48, DEFB126, IGHG2, IGHD, GSDMD, DEFB121, SIGLEC11, IGHV3-43, AZU1, TNFRSF14, HMGB2, IGHV1-58, DCD, DEFB116, PLA2G6, KLK3, TLR2, IGHV2-70, IGHM, DEFB136, DEFB4A, BPI, ISG15, TIRAP, PGLYRP2, DEFB132, C10orf99, BPIFA1, DEFB125, SPN, DEFB106B, HTN1, LYZL2, DEFB131A, IGHV3-49, CRP, PYCARD, FCER1G, DEFA5, IGHV4-61, BPIFA2, IGHV4-59, KLRK1, CD36, EPX, SFTPD, IFNE, DEFB104A, DEFB124, HTN3, DEFA6, IGHV3-30, IL6, NLRP1, RAB14, MR1, MAVS, IGLC1, JCHAIN, DEFB104B, DEFB108A, DEFB119, IGHV1-3, FOXP1, LBP, IGHV4-4, MPO, NOD1, RIPK2, DEFB128, IGHV2-26, NLRP3, RPL39, DEFB106A, DEFA1, KLK5, DEFB105A, DEFB134, DEFB118, IGHV3-15, LYZL1, IGHV3-21, ANXA3, IGHV3-53, FGR, IL23R, FCN2, RNASE8, IGHG3, FGA, IGLC2, IGHA2, SPAG11A, IL10, IGHV3-11, DEFB129, NR1H4, IGLC7, LYPD8, IGHV3-66, IGHV3-73, TLR3, SELP, DEFB103B, DEFA1B, CYBA, LYST, DEFB130A, DEFB109B, IGHV3-13, LEAP2, IGHV1-18, DEFB123, PRB3, F2, DEFB103A, HP, OPTN, IGLL5, IGHV1OR15-1, DEFB130B, IL22RA1, KLK7, DMBT1, IGHV3-72, LPO, SIGLEC16, PLAC8, IGHV1-69, RNASE7, ADGRB1, IL12B, IL12A, RARRES2, DEFB105B, STATH, IGHV3-74, IGHV6-1, IGHV3-64, FGB, SERPINE1, VGF, KLRC4-KLRK1, RNASE6, TLR5, IGLC3, IGHA1, PLA2G2A, ACP5, SPAG11B, IGHG1, ELANE, IGHV4-34, TMF1, RNASE3, IGHV3-20, IGHV1-24, CEBPB, F2RL1, TBK1, IGHV2-5, LACRT, IGHV3-23, EPHA2, RAB1A | 100 |
| 16 | GO:0038095 | Fc-epsilon receptor signaling pathway | IGHV3-53, IGLV3-21, FCER1G, IGHV3-33, IGHV1-69, IGLC6, IGLV2-23, LAT, BCL10, IGHV4-59, FCER1A, PIK3CB, IGHV4-39, VAV2, IGHV3-7, PLCG2, LILRA4, MAPK3, IKBKB, IGLV3-25, GRAP2, NFATC2, IKBKG, PAK2, IGHV3-11, IGHV3-30, IGHE, IGLC7, IGLC1, IGHV3-48, MALT1, IGLV2-8, IGLV1-44, LCP2, IGLV2-11, PPP3R1, PDPK1, NFATC1, BTK, RELA, IGLV6-57, IGLV7-43, IGHV3-13, IGLV3-27, IGHV4-34, IGLV3-1, CARD11, IGLV3-19, IGHV2-70, MAPK1, TEC, GRB2, IGLV2-14, PPP3CB, IGHV2-5, RAC1, IGHV1OR15-1, NR4A3, IGLV1-47, IGLV1-40, PRKCQ, TAB1, MAPK9, LAT2, IGLV1-51, IGHV3-23, MAP3K1, MAPK10 | 100 |
| 16 | GO:0008037 | cell recognition | TCP1, ROBO4, IGLL1, IGHV3-33, IGHG4, IGLC6, PEAR1, IGHV4-28, IGHV4-39, IGHV5-51, IGHV3-7, EPHB3, OVGP1, EPHB2, CCT2, IGHV1-45, IGHE, PRSS37, TRBC2, IGHV3-48, IGHG2, IGHD, DOCK8, IGHV3-43, CNTNAP3, ZAN, IGHV1-58, IGHV2-70, IGHM, VSTM2L, PCDH12, TNN, NDN, PCSK4, IGHV3-49, IGHV4-61, CDK5R1, CNTN2, IGHV4-59, CD36, IGSF9, CSGALNACT1, CCT3, FCN1, CATSPER3, IGHV3-30, IGLC1, ADAM21, ATP8B3, IGHV1-3, FETUB, BSG, IGHV4-4, COLEC12, CLEC7A, IGHV2-26, MSN, ROBO3, IGHV3-15, CNTN4, IGHV3-21, VDAC2, IGHV3-53, SPA17, B4GALT1, ACR, DOCK2, FCN2, IGHG3, MEGF10, IGLC2, IGHA2, SEMA3A, IGHV3-11, ZP4, DSCAM, ALDOA, IGLC7, CLGN, IGHV3-66, IGHV3-73, ADAM2, SEMA5A, CASP3, SPAM1, ZPBP, IGHV3-13, LGALS3, IGHV1-18, EPHA4, GAP43, IGLL5, IGHV1OR15-1, FCN3, IGHV3-72, CCT5, CNTNAP2, NEXN, PCDHA7, AMIGO1, IGHV1-69, PAEP, KCNU1, ADGRB1, CATSPER1, EFNB3, IGHV3-74, IGHV6-1, IGHV3-64, PCDHB6, NRP1, CCT4, MFGE8, IGLC3, IGHA1, NRCAM, IGHG1, DLG1, CD226, ST6GALNAC6, IGHV4-34, LAMA5, EMB, IGHV3-20, IGHV1-24, CNTN6, IGHV2-5, SPESP1, NPTN, IGHV3-23, IZUMO1, UBAP2L | 100 |

| Cluster | Term | Name | HPOs\_in\_clusters |
| --- | --- | --- | --- |
| 16 | OMIM:193500 | WAARDENBURG SYNDROME, TYPE 1; WS1 | HP:0000581, HP:0000316, HP:0000431 |
| 16 | OMIM:265050 | 3MC SYNDROME 2; 3MC2 | HP:0000581, HP:0000316, HP:0000431 |
| 16 | OMIM:601390 | VAN MALDERGEM SYNDROME 1; VMLDS1 | HP:0000581, HP:0000316, HP:0000431 |
| 16 | OMIM:605130 | WIEDEMANN-STEINER SYNDROME; WDSTS | HP:0000581, HP:0000316, HP:0000431 |
| 16 | OMIM:613610 | CRANIOECTODERMAL DYSPLASIA 2; CED2 | HP:0000581, HP:0000316, HP:0000431 |
| 16 | OMIM:615074 | MENTAL RETARDATION, AUTOSOMAL DOMINANT 18; MRD18 | HP:0000581, HP:0000316, HP:0000431 |
| 16 | OMIM:615546 | VAN MALDERGEM SYNDROME 2; VMLDS2 | HP:0000581, HP:0000316, HP:0000431 |
| 16 | OMIM:617333 | INTELLECTUAL DEVELOPMENTAL DISORDER WITH DYSMORPHIC FACIES AND PTOSIS; IDDDFP | HP:0000581, HP:0000316, HP:0000431 |
| 16 | OMIM:617403 | CUTIS LAXA, AUTOSOMAL RECESSIVE, TYPE IID; ARCL2D | HP:0000581, HP:0000316, HP:0000431 |
| 16 | OMIM:109400 | BASAL CELL NEVUS SYNDROME; BCNS | HP:0000316, HP:0000431 |
| 16 | OMIM:110100 | BLEPHAROPHIMOSIS, PTOSIS, AND EPICANTHUS INVERSUS; BPES | HP:0000581, HP:0000431 |
| 16 | OMIM:114290 | CAMPOMELIC DYSPLASIA | HP:0000581, HP:0000316 |
| 16 | OMIM:122860 | CRANIODIAPHYSEAL DYSPLASIA, AUTOSOMAL DOMINANT; CDD | HP:0000316, HP:0000431 |
| 16 | OMIM:136760 | FRONTONASAL DYSPLASIA 1; FND1 | HP:0000316, HP:0000431 |
| 16 | OMIM:139210 | MYHRE SYNDROME; MYHRS | HP:0000581, HP:0000316 |
| 16 | OMIM:145410 | OPITZ GBBB SYNDROME, TYPE II; GBBB2 | HP:0000316, HP:0000431 |
| 16 | OMIM:145420 | HYPERTELORISM, TEEBI TYPE; TBHS | HP:0000316, HP:0000431 |
| 16 | OMIM:148820 | WAARDENBURG SYNDROME, TYPE 3; WS3 | HP:0000581, HP:0000431 |
| 16 | OMIM:156610 | SKIN CREASES, CONGENITAL SYMMETRIC CIRCUMFERENTIAL, 1; CSCSC1 | HP:0000581, HP:0000316 |
| 16 | OMIM:157800 | CARDIOSPONDYLOCARPOFACIAL SYNDROME; CSCF | HP:0000316, HP:0000431 |
| 16 | OMIM:164280 | FEINGOLD SYNDROME 1; FGLDS1 | HP:0000581, HP:0000431 |
| 16 | OMIM:170390 | ANDERSEN CARDIODYSRHYTHMIC PERIODIC PARALYSIS | HP:0000581, HP:0000316 |
| 16 | OMIM:180700 | ROBINOW SYNDROME, AUTOSOMAL DOMINANT 1; DRS1 | HP:0000316, HP:0000431 |
| 16 | OMIM:188400 | DIGEORGE SYNDROME; DGS | HP:0000581, HP:0000316 |
| 16 | OMIM:193700 | ARTHROGRYPOSIS, DISTAL, TYPE 2A; DA2A | HP:0000581, HP:0000431 |
| 16 | OMIM:194190 | WOLF-HIRSCHHORN SYNDROME; WHS | HP:0000316, HP:0000431 |
| 16 | OMIM:208050 | ARTERIAL TORTUOSITY SYNDROME; ATORS | HP:0000581, HP:0000316 |
| 16 | OMIM:208150 | FETAL AKINESIA DEFORMATION SEQUENCE 1; FADS1 | HP:0000581, HP:0000316 |
| 16 | OMIM:211380 | ELSAHY-WATERS SYNDROME; ESWS | HP:0000316, HP:0000431 |
| 16 | OMIM:218000 | AGENESIS OF THE CORPUS CALLOSUM WITH PERIPHERAL NEUROPATHY; ACCPN | HP:0000316, HP:0000431 |
| 16 | OMIM:218400 | CRANIOMETAPHYSEAL DYSPLASIA, AUTOSOMAL RECESSIVE; CMDR | HP:0000316, HP:0000431 |
| 16 | OMIM:219000 | FRASER SYNDROME 1; FRASRS1 | HP:0000316, HP:0000431 |
| 16 | OMIM:220111 | LEIGH SYNDROME, FRENCH CANADIAN TYPE; LSFC | HP:0000316, HP:0000431 |
| 16 | OMIM:235510 | HENNEKAM LYMPHANGIECTASIA-LYMPHEDEMA SYNDROME 1; HKLLS1 | HP:0000316, HP:0000431 |
| 16 | OMIM:235730 | MOWAT-WILSON SYNDROME; MOWS | HP:0000316, HP:0000431 |
| 16 | OMIM:243310 | BARAITSER-WINTER SYNDROME 1; BRWS1 | HP:0000316, HP:0000431 |
| 16 | OMIM:243605 | STROMME SYNDROME; STROMS | HP:0000316, HP:0000431 |
| 16 | OMIM:248340 | 3MC SYNDROME 3; 3MC3 | HP:0000581, HP:0000316 |
| 16 | OMIM:248700 | MARDEN-WALKER SYNDROME; MWKS | HP:0000581, HP:0000316 |
| 16 | OMIM:253250 | MULIBREY NANISM | HP:0000316, HP:0000431 |
| 16 | OMIM:257920 | 3MC SYNDROME 1; 3MC1 | HP:0000581, HP:0000316 |
| 16 | OMIM:258315 | OMODYSPLASIA 1; OMOD1 | HP:0000581, HP:0000431 |
| 16 | OMIM:264470 | PEROXISOMAL ACYL-CoA OXIDASE DEFICIENCY | HP:0000316, HP:0000431 |
| 16 | OMIM:268300 | ROBERTS SYNDROME; RBS | HP:0000316, HP:0000431 |
| 16 | OMIM:268310 | ROBINOW SYNDROME, AUTOSOMAL RECESSIVE 1; RRS1 | HP:0000316, HP:0000431 |
| 16 | OMIM:269500 | SCLEROSTEOSIS 1; SOST1 | HP:0000316, HP:0000431 |
| 16 | OMIM:269921 | SIALURIA | HP:0000316, HP:0000431 |
| 16 | OMIM:270400 | SMITH-LEMLI-OPITZ SYNDROME; SLOS | HP:0000316, HP:0000431 |
| 16 | OMIM:275210 | RESTRICTIVE DERMOPATHY, LETHAL | HP:0000581, HP:0000316 |
| 16 | OMIM:278250 | WRINKLY SKIN SYNDROME; WSS | HP:0000316, HP:0000431 |
| 16 | OMIM:280000 | COLOBOMA, CONGENITAL HEART DISEASE, ICHTHYOSIFORM DERMATOSIS, MENTAL RETARDATION, AND EAR ANOMALIES SYNDROME; CHIME | HP:0000316, HP:0000431 |
| 16 | OMIM:300000 | OPITZ GBBB SYNDROME, TYPE I; GBBB1 | HP:0000316, HP:0000431 |
| 16 | OMIM:300373 | OSTEOPATHIA STRIATA WITH CRANIAL SCLEROSIS; OSCS | HP:0000316, HP:0000431 |
| 16 | OMIM:300422 | FG SYNDROME 4; FGS4 | HP:0000316, HP:0000431 |
| 16 | OMIM:300749 | MENTAL RETARDATION AND MICROCEPHALY WITH PONTINE AND CEREBELLAR HYPOPLASIA; MICPCH | HP:0000316, HP:0000431 |
| 16 | OMIM:300895 | OHDO SYNDROME, X-LINKED; OHDOX | HP:0000581, HP:0000431 |
| 16 | OMIM:300978 | TONNE-KALSCHEUER SYNDROME; TOKAS | HP:0000316, HP:0000431 |
| 16 | OMIM:304110 | CRANIOFRONTONASAL SYNDROME; CFNS | HP:0000316, HP:0000431 |
| 16 | OMIM:305620 | FRONTOMETAPHYSEAL DYSPLASIA 1; FMD1 | HP:0000316, HP:0000431 |
| 16 | OMIM:309580 | MENTAL RETARDATION-HYPOTONIC FACIES SYNDROME, X-LINKED, 1; MRXHF1 | HP:0000316, HP:0000431 |
| 16 | OMIM:311200 | OROFACIODIGITAL SYNDROME I; OFD1 | HP:0000316, HP:0000431 |
| 16 | OMIM:311300 | OTOPALATODIGITAL SYNDROME, TYPE I; OPD1 | HP:0000316, HP:0000431 |
| 16 | OMIM:311900 | TARP SYNDROME; TARPS | HP:0000316, HP:0000431 |
| 16 | OMIM:312870 | SIMPSON-GOLABI-BEHMEL SYNDROME, TYPE 1; SGBS1 | HP:0000316, HP:0000431 |
| 16 | OMIM:601186 | MICROPHTHALMIA, SYNDROMIC 9; MCOPS9 | HP:0000581, HP:0000431 |
| 16 | OMIM:603671 | ACROMELIC FRONTONASAL DYSOSTOSIS; AFND | HP:0000316, HP:0000431 |
| 16 | OMIM:605039 | BOHRING-OPITZ SYNDROME; BOPS | HP:0000316, HP:0000431 |
| 16 | OMIM:607812 | CRANIOLENTICULOSUTURAL DYSPLASIA; CLSD | HP:0000316, HP:0000431 |
| 16 | OMIM:608572 | BURN-MCKEOWN SYNDROME; BMKS | HP:0000581, HP:0000316 |
| 16 | OMIM:609528 | CEREBRAL DYSGENESIS, NEUROPATHY, ICHTHYOSIS, AND PALMOPLANTAR KERATODERMA SYNDROME | HP:0000316, HP:0000431 |
| 16 | OMIM:609924 | AMINOACYLASE 1 DEFICIENCY; ACY1D | HP:0000316, HP:0000431 |
| 16 | OMIM:611174 | HAMAMY SYNDROME; HMMS | HP:0000316, HP:0000431 |
| 16 | OMIM:611209 | CONGENITAL DISORDER OF GLYCOSYLATION, TYPE IIg; CDG2G | HP:0000316, HP:0000431 |
| 16 | OMIM:612563 | DIAMOND-BLACKFAN ANEMIA 8; DBA8 | HP:0000316, HP:0000431 |
| 16 | OMIM:613177 | CUTIS LAXA, AUTOSOMAL RECESSIVE, TYPE IC; ARCL1C | HP:0000316, HP:0000431 |
| 16 | OMIM:613224 | NOONAN SYNDROME 6; NS6 | HP:0000316, HP:0000431 |
| 16 | OMIM:613406 | WITTEVEEN-KOLK SYNDROME; WITKOS | HP:0000316, HP:0000431 |
| 16 | OMIM:613451 | FRONTONASAL DYSPLASIA 2; FND2 | HP:0000316, HP:0000431 |
| 16 | OMIM:613456 | FRONTONASAL DYSPLASIA 3; FND3 | HP:0000316, HP:0000431 |
| 16 | OMIM:614067 | SPASTIC PARAPLEGIA 52, AUTOSOMAL RECESSIVE; SPG52 | HP:0000316, HP:0000431 |
| 16 | OMIM:614202 | MENTAL RETARDATION, AUTOSOMAL RECESSIVE 15; MRT15 | HP:0000316, HP:0000431 |
| 16 | OMIM:614438 | CUTIS LAXA, AUTOSOMAL RECESSIVE, TYPE IIIB; ARCL3B | HP:0000581, HP:0000316 |
| 16 | OMIM:614749 | HYPERPHOSPHATASIA WITH MENTAL RETARDATION SYNDROME 2; HPMRS2 | HP:0000316, HP:0000431 |
| 16 | OMIM:614976 | CARPENTER SYNDROME 2; CRPT2 | HP:0000316, HP:0000431 |
| 16 | OMIM:615155 | STEEL SYNDROME; STLS | HP:0000316, HP:0000431 |
| 16 | OMIM:615716 | HYPERPHOSPHATASIA WITH MENTAL RETARDATION SYNDROME 4; HPMRS4 | HP:0000316, HP:0000431 |
| 16 | OMIM:616737 | TAKENOUCHI-KOSAKI SYNDROME; TKS | HP:0000316, HP:0000431 |
| 16 | OMIM:616897 | OSTEOCHONDRODYSPLASIA, COMPLEX LETHAL, SYMOENS-BARNES-GISTELINCK TYPE; OCLSBG | HP:0000316, HP:0000431 |
| 16 | OMIM:616920 | HEART AND BRAIN MALFORMATION SYNDROME; HBMS | HP:0000316, HP:0000431 |
| 16 | OMIM:617062 | OKUR-CHUNG NEURODEVELOPMENTAL SYNDROME; OCNDS | HP:0000316, HP:0000431 |
| 16 | OMIM:617127 | OROFACIODIGITAL SYNDROME XV; OFD15 | HP:0000316, HP:0000431 |
| 16 | OMIM:617137 | FRONTOMETAPHYSEAL DYSPLASIA 2; FMD2 | HP:0000316, HP:0000431 |
| 16 | OMIM:617360 | CONGENITAL HEART DEFECTS, DYSMORPHIC FACIAL FEATURES, AND INTELLECTUAL DEVELOPMENTAL DISORDER; CHDFIDD | HP:0000316, HP:0000431 |
| 16 | OMIM:617561 | COHEN-GIBSON SYNDROME; COGIS | HP:0000316, HP:0000431 |
| 16 | OMIM:617746 | SWEENEY-COX SYNDROME; SWCOS | HP:0000316, HP:0000431 |
| 16 | OMIM:617883 | FANCONI ANEMIA, COMPLEMENTATION GROUP S; FANCS | HP:0000581, HP:0000316 |
| 16 | OMIM:618067 | EPILEPTIC ENCEPHALOPATHY, EARLY INFANTILE, 66; EIEE66 | HP:0000316, HP:0000431 |
| 16 | OMIM:618106 | MENTAL RETARDATION, AUTOSOMAL DOMINANT 58; MRD58 | HP:0000316, HP:0000431 |
| 16 | OMIM:618147 | INTELLECTUAL DEVELOPMENTAL DISORDER WITH HYPERTELORISM AND DISTINCTIVE FACIES; IDDHDF | HP:0000316, HP:0000431 |
| 16 | OMIM:618205 | SNIJDERS BLOK-CAMPEAU SYNDROME; SNIBCPS | HP:0000316, HP:0000431 |
| 16 | OMIM:618316 | INTELLECTUAL DEVELOPMENTAL DISORDER WITH CARDIAC DEFECTS AND DYSMORPHIC FACIES; IDDCDF | HP:0000316, HP:0000431 |
| 16 | OMIM:618356 | NEURODEVELOPMENTAL DISORDER WITH CENTRAL AND PERIPHERAL MOTOR DYSFUNCTION; NEDCPMD | HP:0000316, HP:0000431 |

---


---


---

# Cluster 19

| Cluster | Term | Name |
| --- | --- | --- |
| 19 | HP:0001252 | Muscular hypotonia |
| 19 | HP:0000316 | Hypertelorism |
| 19 | HP:0000347 | Micrognathia |
| 19 | HP:0000431 | Wide nasal bridge |

| Cluster | Term | Name | Genes | Percentage\_of\_nodes\_with\_funsys |
| --- | --- | --- | --- | --- |
| 19 | GO:0002920 | regulation of humoral immune response | IGHV3-53, IGLV3-21, IGHV3-33, CXCL13, IGHV1-69, IGHG4, IGLC6, IGLV2-23, CPN2, CR1, ACOD1, IGHV4-59, IGHV4-39, CFHR1, C8G, IGHG3, IGHV3-7, CD46, C4BPA, CFHR4, IGLV3-25, SUSD4, IGHV3-11, ZP4, IGHV3-30, C1QC, PPP2R3C, CFHR5, IGLC7, CD55, IGHV3-48, IGLC1, IGLV2-8, IGLV1-44, IGHG2, IGLV2-11, C1QA, IGHG1, IGLV6-57, IGLV7-43, C5, IGHV3-13, IGLV3-27, IGHV4-34, PTPRC, IGLV3-1, CFHR2, IGLV3-19, IGHV2-70, C1QB, FCGR2B, C7, C1QBP, IGLV2-14, CR2, C6, KLK5, C4BPB, IGHV2-5, IGHV1OR15-1, KLK7, IGLV1-47, IGLV1-40, CD19, C9, CFH, CLU, IGLV1-51, IGHV3-23, CPB2, A2M | 100 |
| 19 | GO:0072376 | protein activation cascade | SERPINC1, IGLL1, IGHV3-33, IGHG4, IGLC6, IGLV2-23, TFPI, CR1, IGHV4-28, IGHV4-39, C8G, IGHV5-51, IGHV3-7, KRT1, F9, IGLV3-25, CFHR4, IGHV1-45, IGHE, TRBC2, CFHR5, IGHV3-48, IGLV2-8, IGHG2, IGHD, IGLV2-11, IGHV3-43, IGHV1-58, APCS, C1QBP, IGLV3-1, CFHR2, IGHV2-70, IGHM, GP5, C6, C4BPB, IGLV1-51, CPB2, IGHV3-49, SCARA3, CRP, IGHV4-61, IGHV4-59, CFHR1, C4BPA, FCN1, KLKB1, C1QC, IGHV3-30, IGLC1, IGHV1-3, F7, IGHV4-4, F12, IGLV7-43, IGLV3-27, C1QB, IGHV2-26, IGLV3-19, CR2, IGHV3-15, IGLV1-40, C9, CFH, IGHV3-21, FBLN1, RGCC, IGHV3-53, CFD, F8, CPN2, FCN2, IGHG3, FGA, IGLC2, CD46, IGHA2, MASP2, F13B, IGHV3-11, SUSD4, IGLC7, CD55, IGHV3-66, IGHV3-73, IGHV3-13, F10, FGG, IGHV1-18, COL20A1, MASP1, IGLV2-14, KNG1, IGLL5, IGHV1OR15-1, IGLV1-47, FCN3, IGHV3-72, A2M, IGLV3-21, IGHV1-69, IGHV3-74, IGHV6-1, GP1BB, IGHV3-64, FGB, COLEC10, GP1BA, VSIG4, IGLV1-44, IGLC3, IGHA1, C1QA, IGHG1, IGLV6-57, C5, IGHV4-34, F11, C7, IGHV3-20, IGHV1-24, IGHV2-5, CD19, CLU, IGHV3-23 | 100 |
| 19 | GO:0010324 | membrane invagination | SYT11, IGLL1, IGHV3-33, IGHG4, IGLC6, MSR1, SH3BP1, DOCK1, IGHV4-28, IGHV4-39, IGHV5-51, IGHV3-7, IGHV1-45, IGHE, TRBC2, IGHV3-48, IGHG2, IGHD, IGHV3-43, IGHV1-58, IGHV2-70, IGHM, FCGR2B, PPARG, ALKBH4, IGHV3-49, FCER1G, IGHV4-61, IGHV4-59, CD36, IGHV3-30, IGLC1, ITGB2, IGHV1-3, IGHV4-4, IGHV2-26, ARHGAP12, IGHV3-15, IGHV3-21, FCGR1A, RHOBTB2, IGHV3-53, GSN, IGHG3, MEGF10, IGLC2, IGHA2, SPIRE2, IGHV3-11, IGLC7, STAP1, IGHV3-66, IGHV3-73, ELMO1, IGHV3-13, IGHV1-18, IGLL5, IGHV1OR15-1, IGHV3-72, ARF1, IGHV1-69, NCKAP1L, ADGRB1, ARHGAP25, MYH9, IGHV3-74, IGHV6-1, IGHV3-64, ITGAM, ABCA7, MFGE8, SNX9, IGLC3, IGHA1, CD300A, IGHG1, SPIRE1, IGHV4-34, SNX33, GULP1, IGHV3-20, IGHV1-24, XKR8, F2RL1, SMURF1, IGHV2-5, IGHV3-23 | 100 |
| 19 | GO:0002455 | humoral immune response mediated by circulating immunoglobulin | IGLL1, IGHV3-33, IGHG4, IGLC6, IGLV2-23, CR1, IGHV4-28, IGHV4-39, C8G, IGHV5-51, IGHV3-7, IGLV3-25, EXO1, IGHV1-45, IGHE, TRBC2, IGHV3-48, IGLV2-8, IGHG2, IGHD, IGLV2-11, IGHV3-43, IGHV1-58, APCS, C1QBP, IGLV3-1, IGHV2-70, IGHM, FCGR2B, C6, C4BPB, IGLV1-51, IGHV3-49, CRP, IGHV4-61, IGHV4-59, C4BPA, C1QC, IGHV3-30, IGLC1, IGHV1-3, IGHV4-4, IGLV7-43, IGLV3-27, C1QB, IGHV2-26, IGLV3-19, CR2, IGHV3-15, IGLV1-40, C9, IGHV3-21, IGHV3-53, IGHG3, CD46, IGLC2, IGHA2, MASP2, IGHV3-11, SUSD4, IGLC7, CD55, IGHV3-66, IGHV3-73, IGHV3-13, IGHV1-18, PTPRC, IGLV2-14, IGLL5, IGHV1OR15-1, IGLV1-47, IGHV3-72, IGLV3-21, IGHV1-69, IGHV3-74, IGHV6-1, IGHV3-64, IGLV1-44, IGLC3, IGHA1, C1QA, IGHG1, IGLV6-57, C5, IGHV4-34, C7, IGHV3-20, IGHV1-24, IGHV2-5, CLU, IGHV3-23 | 100 |
| 19 | GO:1903317 | regulation of protein maturation | IGHV3-33, IGHG4, IGLC6, IGLV2-23, CR1, IGHV4-39, C8G, MELTF, IGHV3-7, IGLV3-25, CFHR4, PRSS37, CFHR5, IGHV3-48, IGLV2-8, IGHG2, IGLV2-11, USP17L2, NKD2, NOL3, C1QBP, IGLV3-1, CFHR2, IGHV2-70, C6, C4BPB, ACP4, IGLV1-51, CPB2, PRKACB, SOX4, CNTN2, IGHV4-59, CFHR1, C4BPA, KLKB1, C1QC, IGHV3-30, IGLC1, SERPINF2, ANXA2P2, F12, ADAM8, IGLV7-43, IGLV3-27, C1QB, IGLV3-19, CR2, XIAP, IGLV1-40, C9, PLGRKT, CFH, TMEM59, IGHV3-53, GSN, CPN2, IGHG3, TFR2, CD46, IGHV3-11, SUSD4, ENO1, IGLC7, CD55, TIMM17A, CARD8, IGHV3-13, IGLV2-14, CTSZ, IGHV1OR15-1, SERPINE2, IGLV1-47, ANGPTL8, A2M, IGLV3-21, IGHV1-69, MYH9, SERPINE1, NLRP7, BIRC7, IGLV1-44, BCL2L12, C1QA, IGHG1, IGLV6-57, SRC, C5, IGHV4-34, C7, IL1R2, IGHV2-5, CD19, CLU, TNP2, IGHV3-23, CCBE1, GLG1 | 100 |
| 19 | GO:0006910 | phagocytosis, recognition | IGHV3-53, IGLL1, IGHV3-33, IGHV1-69, IGHV4-61, IGHG4, IGLC6, IGHV4-59, FCN2, PEAR1, IGHV4-28, IGHV4-39, ADGRB1, IGHG3, IGHV5-51, IGHV3-7, MEGF10, CD36, IGLC2, FCN1, IGHA2, IGHV3-74, IGHV6-1, IGHV3-11, IGHV3-30, IGHE, IGHV1-45, IGHV3-64, TRBC2, IGLC7, IGLC1, IGHV3-48, IGHV3-66, IGHV3-73, MFGE8, IGHG2, IGHD, IGLC3, IGHA1, IGHV1-3, IGHV3-43, IGHG1, IGHV4-4, COLEC12, CLEC7A, IGHV1-58, IGHV3-13, IGHV4-34, IGHV1-18, IGHV2-26, IGHV2-70, IGHM, IGHV3-20, IGHV1-24, IGLL5, IGHV2-5, IGHV1OR15-1, IGHV3-15, FCN3, IGHV3-21, IGHV3-72, IGHV3-23, IGHV3-49 | 100 |
| 19 | GO:0050853 | B cell receptor signaling pathway | IGLL1, IGHV3-33, IGHG4, IGLC6, KLHL6, BLK, IGHV4-28, IGHV4-39, IGHV5-51, IGHV3-7, PLCG2, IGHV1-45, IGHE, TRBC2, IGHV3-48, CTLA4, IGHG2, IGHD, NFAM1, IGHV3-43, IGHV1-58, IGHV2-70, IGHM, GCSAM, MAPK1, ELF2, SLC39A10, LAT2, IGHV3-49, IGHV4-61, IGHV4-59, CMTM3, MNDA, LIME1, PIK3CD, IGHV3-30, IGLC1, PAX5, IGHV1-3, IGHV4-4, IGHV2-26, CBFB, IGHV3-15, PLEKHA1, IGHV3-21, IGHV3-53, GCSAML, IGHG3, IGLC2, NFATC2, IGHA2, ABL1, IGHV3-11, PLCL2, IGLC7, STAP1, IGHV3-66, IGHV3-73, BCL2, BTK, MEF2C, IGHV3-13, IGHV1-18, PTPRC, IGLL5, FCRL3, IGHV1OR15-1, IGHV3-72, GPS2, IGHV1-69, CD38, NCKAP1L, IGHV3-74, IGHV6-1, IGHV3-64, IGLC3, IGHA1, CD300A, IGHG1, IGHV4-34, BAX, TEC, IGHV3-20, IGHV1-24, IGHV2-5, ELF1, CD19, RFTN1, IGHV3-23 | 100 |
| 19 | GO:2000257 | regulation of protein activation cascade | IGHV3-53, IGLV3-21, SERPINC1, IGHV3-33, IGHV1-69, IGHG4, IGLC6, IGLV2-23, CPN2, CR1, IGHV4-59, IGHV4-39, IGHG3, CFHR1, C8G, IGHV3-7, CD46, C4BPA, CFHR4, IGLV3-25, SUSD4, IGHV3-11, C1QC, IGHV3-30, CFHR5, IGLC7, CD55, IGHV3-48, IGLC1, IGLV2-8, IGLV1-44, IGHG2, IGLV2-11, C1QA, IGHG1, IGLV6-57, IGLV7-43, C5, IGHV3-13, IGLV3-27, IGHV4-34, IGLV3-1, C1QB, CFHR2, IGLV3-19, IGHV2-70, C7, C1QBP, IGLV2-14, CR2, C6, C4BPB, IGHV2-5, IGHV1OR15-1, IGLV1-47, IGLV1-40, CD19, C9, CFH, CLU, IGLV1-51, IGHV3-23, CPB2, A2M | 100 |
| 19 | GO:0006898 | receptor-mediated endocytosis | SYT11, AMN, AP1S1, CLTCL1, IGHV3-33, CACNG3, CLTC, IGLC6, IGLV2-23, ACHE, AP2A1, MSR1, IGHV4-39, OPHN1, GPR107, IGHV3-7, PLCG2, IGLV3-25, EFNB2, CANX, CACNG5, WASL, EPS15, IGHV3-48, CALCRL, IGLV2-8, SCART1, IGLV2-11, GSG1L, SCYL2, CAV1, LRP1B, CAV3, ILDR1, CD63, VLDLR, SFRP4, RAMP1, LRP2, IGLV3-1, CLEC9A, IGHV2-70, FCGR2B, NEDD4, CACNG8, GRB2, IGF2R, MIR199A2, HTR1B, SCRIB, CLTB, IGLV1-51, SCARA3, FCER1G, SYNJ1, DAB2, CNTN2, IGHV4-59, CD36, SFTPD, SGIP1, IGHV3-30, HSP90AA1, SCARF1, IGLC1, MIR185, JCHAIN, GRK4, ITGB2, DBNL, ASGR2, FMR1, COLEC12, ANXA2P2, NECAB2, IGLV7-43, SCGB3A2, IGLV3-27, PLA2R1, IGLV3-19, DNM1P34, LRRTM2, CD5L, RAC1, SUSD2, IGLV1-40, HHIPL1, FCGR1A, EZR, IGHV3-53, CLTA, SMAP1, LILRB1, MKLN1, CACNG2, SH3GL2, MEGF10, TFR2, SCARA5, RABEPK, APOL1, IGHA2, TBC1D5, IGHV3-11, HIP1, PICK1, CD207, CALR, IGLC7, MICALL1, CD14, DNM1, HTR2B, DLL1, SELE, ITGA4, AAK1, CACNG4, PCSK9, IGHV3-13, MASP1, IGLV2-14, LOXL2, HSPH1, TINAGL1, CLN3, RAB31, CAV2, APOBR, IGHV1OR15-1, LDLR, IGLV1-47, PDLIM7, DMBT1, DNM3, PRG4, IGLV3-21, ASGR1, IGHV1-69, TNK2, ENPP2, GAK, LRPAP1, PIK3CB, ATAD1, CALY, PIP5K1C, CUBN, SERPINE1, SNCA, CACNG7, MRC1, IGLV1-44, ACKR3, SNX9, IGHA1, IGLV6-57, RAMP3, IGHV4-34, RAB21, MAGI2, PRSS12, DRD3, LRP6, IGHV2-5, CLU, DLG4, ITGB1, IGHV3-23, AP2M1, TFRC | 100 |
| 19 | GO:0030449 | regulation of complement activation | IGHV3-53, IGLV3-21, IGHV3-33, IGHV1-69, IGHG4, IGLC6, IGLV2-23, CPN2, CR1, IGHV4-59, IGHV4-39, IGHG3, CFHR1, C8G, IGHV3-7, CD46, C4BPA, CFHR4, IGLV3-25, SUSD4, IGHV3-11, C1QC, IGHV3-30, CFHR5, IGLC7, CD55, IGHV3-48, IGLC1, IGLV2-8, IGLV1-44, IGHG2, IGLV2-11, C1QA, IGHG1, IGLV6-57, IGLV7-43, C5, IGHV3-13, IGLV3-27, IGHV4-34, IGLV3-1, C1QB, CFHR2, IGLV3-19, IGHV2-70, C7, C1QBP, IGLV2-14, CR2, C6, C4BPB, IGHV2-5, IGHV1OR15-1, IGLV1-47, IGLV1-40, CD19, C9, CFH, CLU, IGLV1-51, IGHV3-23, CPB2, A2M | 100 |
| 19 | GO:0016064 | immunoglobulin mediated immune response | IGLL1, IGHV3-33, CD40LG, IGHG4, IGLC6, IGLV2-23, FOXP3, CR1, IGHV4-28, IGHV4-39, C8G, IGHV5-51, IGHV3-7, IGLV3-25, EXO1, MLH1, IGHV1-45, IGHE, TNFSF4, TRBC2, IGHV3-48, IGLV2-8, IGHG2, IGHD, IGLV2-11, IGHV3-43, BCL6, IL13RA2, IGHV1-58, APCS, C1QBP, IGLV3-1, IGHV2-70, IGHM, TNFSF13, FCGR2B, C6, C4BPB, XCL1, MAD2L2, IGLV1-51, IGHV3-49, CRP, SHLD2, FCER1G, IGHV4-61, THOC1, IL4, INPP5D, IGHV4-59, C4BPA, C1QC, IGHV3-30, RIF1, IGLC1, APLF, IGHV1-3, LIG4, IGHV4-4, IGLV7-43, IGLV3-27, C1QB, IGHV2-26, IGLV3-19, PAXIP1, CR2, HSPD1, IGHV3-15, IGLV1-40, CD28, C9, IGHV3-21, IGHV3-53, IGHG3, IGLC2, CD46, IGHA2, IL2, MASP2, IGHV3-11, SUSD4, IL10, IGLC7, CD55, IGHV3-66, IGHV3-73, BTK, IGHV3-13, GAPT, IGHV1-18, PTPRC, IGLV2-14, IGLL5, IGHV1OR15-1, IGLV1-47, IGHV3-72, ATAD5, NSD2, IGLV3-21, MSH6, IGHV1-69, CCR6, NBN, BCL10, RNF168, IGHV3-74, IGHV6-1, IGHV3-64, MSH2, NDFIP1, IL4R, IGLV1-44, IGLC3, IGHA1, C1QA, IGHG1, IGLV6-57, CD226, C5, IGHV4-34, C7, CLCF1, IGHV3-20, IGHV1-24, IGHV2-5, CLU, IGHV3-23, TFRC | 100 |
| 19 | GO:0002673 | regulation of acute inflammatory response | IGHV3-33, PARK7, IGHG4, IGLC6, IGLV2-23, CR1, TNFSF11, IGHV4-39, C8G, IGHV3-7, CFHR4, IGLV3-25, MIR92A2, GSTP1, CFHR5, IGHV3-48, IGLV2-8, IGHG2, IGLV2-11, PIK3CG, APCS, C1QBP, IGLV3-1, CFHR2, OSMR, IGHV2-70, FCGR2B, C6, TAC1, C4BPB, PPARG, IGLV1-51, CPB2, FCER1G, IGHV4-59, CFHR1, C4BPA, KLKB1, TNFRSF11A, IGHV3-30, IL6, C1QC, IL6ST, IGLC1, F12, ADAM8, IGLV7-43, C1QB, IGLV3-27, RHBDD3, ADORA1, NLRP3, IGLV3-19, CR2, IGLV1-40, C9, CFH, IL20RB, IGHV3-53, SELENOS, CPN2, MIR92A1, IGHG3, CD46, SUSD4, IGHV3-11, IGLC7, CD55, OSM, CREB3L3, DNASE1, BTK, ADCYAP1, IGHV3-13, IGLV2-14, IGHV1OR15-1, IGLV1-47, DNASE1L3, A2M, IGLV3-21, IGHV1-69, EDNRB, ASH1L, PTGS2, PTGER3, IGLV1-44, C1QA, IGHG1, IGLV6-57, C5, IGHV4-34, C7, IGHV2-5, CD19, CLU, IGHV3-23 | 100 |
| 19 | GO:0050871 | positive regulation of B cell activation | IGLL1, IGHV3-33, IGHG4, IGLC6, IGHV4-28, IGHV4-39, IGHV5-51, IGHV3-7, MLH1, IGHV1-45, IGHE, TNFSF4, TRBC2, PCID2, IGHV3-48, IGHG2, IGHD, IGHV3-43, BCL6, IGHV1-58, CARD11, IGHV2-70, IGHM, TNFSF13, IL7, MAD2L2, SLC39A10, IGHV3-49, SHLD2, IGHV4-61, IL4, INPP5D, IGHV4-59, IL6, IGHV3-30, GPR183, RIF1, PPP2R3C, IGLC1, IGHV3-23, BAD, IGHV1-3, TNFRSF13C, IGHV4-4, IGHV2-26, IRS2, PAXIP1, IGHV3-15, CD28, IGHV3-21, IGHV3-53, IL5, MIF, IGHG3, IGLC2, NFATC2, IGHA2, IL2, IGHV3-11, IGLC7, IGHV3-66, IGHV3-73, TNIP2, BCL2, BTK, IL21, MEF2C, ATP11C, IGHV3-13, IGHV1-18, PTPRC, IGLL5, FCRL3, IGHV1OR15-1, IGHV3-72, ATAD5, NSD2, MSH6, SASH3, IGHV1-69, CD38, TNFRSF4, NCKAP1L, WNT3A, IGHV3-74, IGHV6-1, BST1, IGHV3-64, MSH2, IGLC3, IGHA1, XBP1, IGHG1, IGHV4-34, CLCF1, TNFSF13B, IGHV3-20, IGHV1-24, IGHV2-5, PELI1, TICAM1, IL13, TFRC, TIRAP | 100 |
| 19 | GO:0099024 | plasma membrane invagination | IGLL1, IGHV3-33, IGHG4, IGLC6, MSR1, SH3BP1, DOCK1, IGHV4-28, IGHV4-39, IGHV5-51, IGHV3-7, IGHV1-45, IGHE, TRBC2, IGHV3-48, IGHG2, IGHD, IGHV3-43, IGHV1-58, IGHV2-70, IGHM, FCGR2B, PPARG, ALKBH4, IGHV3-49, FCER1G, IGHV4-61, IGHV4-59, CD36, IGHV3-30, IGLC1, ITGB2, IGHV1-3, IGHV4-4, IGHV2-26, ARHGAP12, IGHV3-15, IGHV3-21, FCGR1A, RHOBTB2, IGHV3-53, GSN, IGHG3, MEGF10, IGLC2, IGHA2, SPIRE2, IGHV3-11, IGLC7, STAP1, IGHV3-66, IGHV3-73, ELMO1, IGHV3-13, IGHV1-18, IGLL5, IGHV1OR15-1, IGHV3-72, ARF1, IGHV1-69, NCKAP1L, ADGRB1, ARHGAP25, MYH9, IGHV3-74, IGHV6-1, IGHV3-64, ITGAM, ABCA7, MFGE8, SNX9, IGLC3, IGHA1, CD300A, IGHG1, SPIRE1, IGHV4-34, SNX33, GULP1, IGHV3-20, IGHV1-24, XKR8, F2RL1, IGHV2-5, IGHV3-23 | 100 |
| 19 | GO:0019724 | B cell mediated immunity | IGLL1, IGHV3-33, CD40LG, IGHG4, IGLC6, IGLV2-23, FOXP3, CR1, IGHV4-28, IGHV4-39, C8G, IGHV5-51, IGHV3-7, IGLV3-25, EXO1, MLH1, IGHV1-45, IGHE, TNFSF4, TRBC2, IGHV3-48, IGLV2-8, IGHG2, IGHD, IGLV2-11, IGHV3-43, BCL6, IL13RA2, IGHV1-58, APCS, C1QBP, IGLV3-1, IGHV2-70, IGHM, TNFSF13, FCGR2B, C6, C4BPB, XCL1, MAD2L2, IGLV1-51, IGHV3-49, CRP, SHLD2, FCER1G, IGHV4-61, THOC1, IL4, INPP5D, IGHV4-59, C4BPA, C1QC, IGHV3-30, RIF1, IGLC1, APLF, IGHV3-23, IGHV1-3, LIG4, IGHV4-4, IGLV7-43, IGLV3-27, C1QB, IGHV2-26, IGLV3-19, PAXIP1, CR2, HSPD1, IGHV3-15, IGLV1-40, CD28, C9, IGHV3-21, IGHV3-53, IGHG3, IGLC2, CD46, IGHA2, IL2, MASP2, IGHV3-11, SUSD4, IL10, IGLC7, CD55, IGHV3-66, IGHV3-73, BTK, IGHV3-13, GAPT, IGHV1-18, PTPRC, IGLV2-14, IGLL5, IGHV1OR15-1, IGLV1-47, IGHV3-72, ATAD5, NSD2, IGLV3-21, MSH6, IGHV1-69, CCR6, NBN, BCL10, RNF168, IGHV3-74, IGHV6-1, IGHV3-64, MSH2, NDFIP1, IL4R, IGLV1-44, IGLC3, IGHA1, C1QA, IGHG1, IGLV6-57, CD226, C5, IGHV4-34, C7, CLCF1, IGHV3-20, IGHV1-24, IGHV2-5, CLU, SLA2, TFRC | 100 |
| 19 | GO:0038093 | Fc receptor signaling pathway | IGHV3-33, IGHG4, IGLC6, IGLV2-23, DOCK1, IGHV4-39, VAV2, IGHV3-7, PLCG2, IGLV3-25, PAK2, IKBKG, IGHE, WASL, IGHV3-48, IGLV2-8, LCP2, IGHG2, IGLV2-11, ACTB, RELA, CD247, CARD11, PLA2G6, PRKCE, IGLV3-1, IGHV2-70, FCGR2B, MAPK1, CRK, WASF2, GRB2, ARPC5, PPP3CB, LAT2, IGLV1-51, ARPC4, MAPK10, CD47, FCER1G, RAPGEF1, RAP1A, IGHV4-59, LILRA4, CYFIP1, IKBKB, IGHV3-30, HSP90AA1, MYO1C, ARPC1B, IGLC1, PPP3R1, PDPK1, IGLV7-43, IGLV3-27, IGLV3-19, MYO1G, RAC1, IGLV1-40, FCGR1A, MAP3K1, YES1, IGHV3-53, WAS, FGR, HCK, LAT, ACTR2, IGHG3, FCGR2A, PTPRJ, NFATC2, ABL1, IGHV3-11, IGLC7, MALT1, BTK, ELMO1, PTK2, IGHV3-13, PTPRC, WIPF1, CD200R1, IGLV2-14, IGHV1OR15-1, IGLV1-47, TAB1, IGLV3-21, BRK1, ABI1, IGHV1-69, BCL10, FCER1A, NCKAP1L, PIK3CB, MAPK3, MYO10, GRAP2, NCK1, ARPC1A, IGLV1-44, NFATC1, IGHG1, IGLV6-57, SRC, PIGR, IGHV4-34, WIPF3, TEC, FCGR3A, IGHV2-5, NCKAP1, PRKCQ, MAPK9, FYN, LIMK1, NR4A3, IGHV3-23 | 100 |
| 19 | GO:0002429 | immune response-activating cell surface receptor signaling pathway | TESPA1, IGLL1, IGHV3-33, CARD9, FPR1, RAB29, IGHG4, IGLC6, IGLV2-23, PVRIG, PAG1, CR1, CSK, KLHL6, FOXP3, DOCK1, IGHV4-28, IGHV4-39, DENND1B, DUSP22, IGHV5-51, IGHV3-7, VAV2, PLCG2, BLK, IGLV3-25, PAK2, IKBKG, IGHV1-45, IGHE, TRBC2, WASL, IGHV3-48, CTLA4, HRAS, IGLV2-8, PTPN2, CLEC10A, IGHG2, IGHD, NFAM1, IGLV2-11, LILRA2, ACTB, LCP2, RBCK1, MUC7, IGHV3-43, RELA, CD247, IGHV1-58, MUC17, FPR2, CARD11, PLA2G6, PRKCE, IGLV3-1, PAWR, IGHV2-70, IGHM, FCGR2B, MAPK1, CRK, WASF2, GCSAM, GRB2, ELF2, ARPC5, PHPT1, SLC39A10, LAT2, IGLV1-51, IGHV3-49, PDE4D, ARPC4, CD276, CD47, FCER1G, PRKACB, PAK3, RAPGEF1, IGHV4-61, RAP1A, INPP5D, TXK, IGHV4-59, CREBBP, MNDA, LIME1, CMTM3, KLRK1, TRAT1, BTNL9, PIK3CD, CYFIP1, IKBKB, FCN1, FPR3, IGHV3-30, HSP90AA1, MYO1C, ARPC1B, IGLC1, PAX5, PDPK1, IGHV1-3, IGHV4-4, IGLV7-43, EP300, IGLV3-27, RIPK2, IGHV2-26, ERMAP, IGLV3-19, SH2B2, DUSP3, CR2, MYO1G, CBFB, HHLA2, RAC1, IGHV3-15, IGLV1-40, MUCL1, MUC4, PLEKHA1, CD28, IGHV3-21, FCGR1A, MUC3A, EZR, YES1, IGHV3-53, WAS, CACNA1F, FGR, BTNL3, PLSCR1, LAT, HCK, GCSAML, RC3H2, ACTR2, IGHG3, FCGR2A, IGLC2, PTPRJ, NFATC2, IGHA2, GPR32, ABL1, IGHV3-11, FYB1, PLCL2, RC3H1, IGLC7, STAP1, MALT1, IGHV3-66, IGHV3-73, PRKACG, MUC12, BCL2, STOML2, BTK, ELMO1, MEF2C, RAF1, PTK2, IGHV3-13, LGALS3, PDE4B, IGHV1-18, PTPRC, WIPF1, BTNL8, GATA3, IGLV2-14, GPR32P1, IGLL5, FCRL3, IGHV1OR15-1, IGLV1-47, TAB1, IGHV3-72, IGLV3-21, GPS2, BRK1, ABI1, IGHV1-69, SLA2, BTNL10, CD38, BCL10, NCKAP1L, PIK3CB, MAPK3, MYO10, GRAP2, IGHV3-74, IGHV6-1, IGHV3-64, NCK1, ARPC1A, THEMIS2, KLRC4-KLRK1, IGLV1-44, IGLC3, IGHA1, LILRB4, CD300A, IGHG1, IGLV6-57, SRC, CD226, IGHV4-34, STK11, WIPF3, BAX, MUC20, IGHV3-20, IGHV1-24, MUC1, TEC, FCGR3A, IGHV2-5, ELF1, NCKAP1, CD19, PRKCQ, FYN, LIMK1, RFTN1, NR4A3, BRAF, IGHV3-23, EIF2B5 | 100 |
| 19 | GO:0038096 | Fc-gamma receptor signaling pathway involved in phagocytosis | IGHV3-33, IGHG4, IGLC6, IGLV2-23, DOCK1, IGHV4-39, VAV2, IGHV3-7, PLCG2, IGLV3-25, WASL, IGHV3-48, IGLV2-8, IGHG2, IGLV2-11, ACTB, CD247, PLA2G6, PRKCE, IGLV3-1, IGHV2-70, FCGR2B, MAPK1, CRK, WASF2, GRB2, ARPC5, IGLV1-51, ARPC4, CD47, RAPGEF1, RAP1A, IGHV4-59, CYFIP1, IGHV3-30, HSP90AA1, MYO1C, ARPC1B, IGLC1, IGLV7-43, IGLV3-27, IGLV3-19, MYO1G, RAC1, IGLV1-40, FCGR1A, YES1, IGHV3-53, WAS, FGR, HCK, ACTR2, IGHG3, FCGR2A, PTPRJ, ABL1, IGHV3-11, IGLC7, ELMO1, PTK2, IGHV3-13, PTPRC, WIPF1, IGLV2-14, IGHV1OR15-1, IGLV1-47, IGLV3-21, BRK1, ABI1, IGHV1-69, NCKAP1L, PIK3CB, MAPK3, MYO10, NCK1, ARPC1A, IGLV1-44, IGHG1, IGLV6-57, SRC, IGHV4-34, WIPF3, FCGR3A, IGHV2-5, NCKAP1, FYN, LIMK1, IGHV3-23 | 100 |
| 19 | GO:0050864 | regulation of B cell activation | IGLL1, IGHV3-33, IGHG4, IGLC6, FOXP3, IGHV4-28, IGHV4-39, IGHV5-51, IGHV3-7, MLH1, IGHV1-45, IGHE, TNFSF4, TRBC2, PCID2, IGHV3-48, CTLA4, IGHG2, IGHD, NFAM1, IGHV3-43, INHBA, BCL6, IGHV1-58, PAWR, CARD11, IGHV2-70, IGHM, TNFSF13, FCGR2B, AHR, TNFRSF13B, IL7, MAD2L2, SLC39A10, IGHV3-49, SHLD2, IGHV4-61, THOC1, IL4, INPP5D, IGHV4-59, MNDA, IL6, IGHV3-30, GPR183, RIF1, PPP2R3C, IGLC1, APLF, IGHV3-23, BAD, IGHV1-3, TNFRSF13C, IGHV4-4, IGHV2-26, IRS2, PAXIP1, IGHV3-15, CD28, IGHV3-21, IGHV3-53, IL5, MIF, IGHG3, IGLC2, NFATC2, IGHA2, IL2, IL10, IGHV3-11, RC3H1, IGLC7, IGHV3-66, IGHV3-73, TNIP2, BCL2, ZFP36L2, CASP3, BTK, IL21, MEF2C, ATP11C, IGHV3-13, IGHV1-18, PTPRC, IGLL5, FCRL3, IGHV1OR15-1, IGHV3-72, ATAD5, NSD2, MSH6, SASH3, IGHV1-69, CD38, TNFRSF4, NCKAP1L, WNT3A, IGHV3-74, IGHV6-1, BST1, IGHV3-64, MSH2, NDFIP1, IGLC3, IGHA1, MIR17HG, XBP1, CD300A, IGHG1, IGHV4-34, IL13, PKN1, CLCF1, TBC1D10C, TNFSF13B, IGHV3-20, IGHV1-24, SFRP1, IGHV2-5, PELI1, TICAM1, SLA2, ZFP36L1, TFRC, TIRAP | 100 |
| 19 | GO:0050867 | positive regulation of cell activation | CD83, TESPA1, IGLL1, SHH, IGHV3-33, EGR3, CD40LG, SIRPB1, GAS6, IGHG4, IGLC6, FOXP3, CSK, TNFSF11, IGHV4-28, IGHV4-39, TMIGD2, IGHV5-51, IGHV3-7, PAK2, EFNB2, AP3D1, MLH1, MIR92A2, IGHV1-45, IGHE, CD1D, TRBC2, TNFSF4, PCID2, IGHV3-48, CTLA4, IGHG2, IGHD, CAV1, DOCK8, IGHV3-43, TNFRSF14, CLECL1, DNAJA3, BCL6, IGHV1-58, CARD11, ANXA1, HMGB1, IGHV2-70, IGHM, TNFSF13, GRB2, IL7, TAC1, XCL1, VCAM1, PTAFR, ICOS, MAD2L2, SLC39A10, IGHV3-49, SHLD2, PYCARD, CD274, ITPKB, FCER1G, CD276, CD47, PAK3, IGHV4-61, DPP4, IL4, INPP5D, IGHV4-59, KLRK1, MIR30B, LEP, MIR21, SART1, IL6, IGHV3-30, GLI3, GPR183, RIF1, PPP2R3C, IL6ST, FADD, IGLC1, IGHV3-23, BAD, ITGB2, CRLF2, PDPK1, IGHV1-3, FLT3LG, TNFRSF13C, LBP, FGF10, IGHV4-4, ADAM8, RIPK2, IGHV2-26, NLRP3, IRS2, PAXIP1, IL1RL1, HSPD1, CBFB, SIRPA, HHLA2, RAC1, STXBP1, IGHV3-15, CD28, IGHV3-21, LAMP1, JAK2, YES1, CORO1A, MAP3K8, IGHV3-53, PDCD1LG2, IL5, FGR, IL23R, MIR92A1, ADORA2B, LILRB1, MIF, GPAM, IGHG3, IGLC2, CD46, NFATC2, IGHA2, IL2, IL10, IGHV3-11, EBI3, SOCS5, ZP4, HES1, NKAP, IGLC7, CD55, STAP1, MALT1, IGHV3-66, IGHV3-73, TNIP2, LGALS1, PRKCZ, SELP, BCL2, BTK, IL21, MEF2C, PDPN, ATP11C, AP1G1, IL2RA, IGHV3-13, IGHV1-18, PTPRC, GATA3, HSPH1, SPTA1, IL1RL2, IGLL5, FCRL3, IGHV1OR15-1, CD86, IGHV3-72, SOX15, HLX, ATAD5, NSD2, MSH6, NPPA, SASH3, IGHV1-69, EPO, CD38, BCL10, TNFRSF4, PLEK, NCKAP1L, WNT3A, IL12B, IL12A, GRAP2, DMTN, EFNB3, IGHV3-74, IGHV6-1, SOCS1, BST1, IGHV3-64, MSH2, NCK1, RASAL3, ITGAM, IL15, IL4R, KLRC4-KLRK1, AKT1, CD80, IGLC3, IGHA1, CCDC88B, PDCD1, XBP1, LILRB4, IGHG1, SRC, DUSP10, CD226, IGHV4-34, IL33, CLCF1, STX4, TNFSF13B, IGHV3-20, IGHV1-24, LILRB2, F2RL1, IGHV2-5, SIRPG, PRKCQ, NR4A3, FYN, BTLA, PELI1, TICAM1, IL13, TFRC, TIRAP | 100 |
| 19 | GO:0051249 | regulation of lymphocyte activation | CD83, TESPA1, IGLL1, SHH, IGHV3-33, EGR3, CD40LG, IRF1, GAS6, IGHG4, IGLC6, FOXP3, PAG1, SIRPB1, CR1, CSK, IRF4, TNFSF11, IGHV4-28, IGHV4-39, TMIGD2, DUSP22, IGHV5-51, IGHV3-7, TARM1, SOCS5, PAK2, EFNB2, AP3D1, MLH1, IGHV1-45, IGHE, IFNB1, TRBC2, CD1D, TNFSF4, PCID2, IGHV3-48, CTLA4, PTPN2, IGHG2, IGHD, NFAM1, FBXO7, CAV1, DOCK8, IGHV3-43, TNFRSF14, CLECL1, DNAJA3, INHBA, BCL6, IGHV1-58, PAWR, CARD11, IDO1, ANXA1, BMP4, HMGB1, IGHV2-70, IGHM, TNFSF13, FCGR2B, AHR, TNFRSF13B, GRB2, IL7, TAC1, XCL1, VCAM1, SOCS6, PGLYRP2, MARCH7, ICOS, MAD2L2, SPN, SLC39A10, IGHV3-49, MAPK8IP1, SHLD2, PYCARD, CD274, ITPKB, MAD1L1, CD276, CD47, PAK3, IGHV4-61, DPP4, THOC1, PIBF1, IL4, INPP5D, IGHV4-59, KLRK1, MNDA, LEP, MIR30B, PDE5A, SFTPD, MIR21, PLA2G2F, IL6, IGHV3-30, GLI3, GPR183, RIF1, SART1, PPP2R3C, IL6ST, FADD, IGLC1, APLF, MERTK, IGHV3-23, BAD, FANCA, PDPK1, IGHV1-3, FLT3LG, TNFRSF13C, FGF10, IGHV4-4, FANCD2, ADAM8, IFNA2, RIPK2, RHBDD3, IGHV2-26, NLRP3, IRS2, PAXIP1, DUSP3, HSPD1, CBFB, SIRPA, SOX13, HHLA2, RAC1, GNRH1, IGHV3-15, CD28, IGHV3-21, LAMP1, YES1, IL20RB, CORO1A, MAP3K8, IGHV3-53, PDCD1LG2, IL5, GSN, LAT, IL23R, NRARP, LILRB1, MIF, RC3H2, GPAM, IGHG3, IGLC2, CD46, NFATC2, IGHA2, IL2, IL10, IGHV3-11, EBI3, ABL1, ZP4, HES1, NKAP, RC3H1, IL27, IGLC7, CD55, MALT1, IGHV3-66, IGHV3-73, TNIP2, LGALS1, PRKCZ, BCL2, ZFP36L2, CASP3, BTK, ZC3H8, IL21, MEF2C, ATP11C, AP1G1, IL2RA, PLA2G2D, IGHV3-13, LGALS3, IGHV1-18, PTPRC, GATA3, HSPH1, SPTA1, IL1RL2, SIT1, IGLL5, FCRL3, IGHV1OR15-1, CD86, IGHV3-72, HLX, ZEB1, ATAD5, NSD2, MSH6, SASH3, IGHV1-69, SLA2, EPO, CD38, BCL10, TNFRSF4, RAC2, TNFSF18, NCKAP1L, WNT3A, IL12B, IL12A, GRAP2, EFNB3, IGHV3-74, IGHV6-1, TNFRSF18, SOCS1, BST1, IGHV3-64, MSH2, NCK1, TWSG1, RASAL3, IL15, NDFIP1, IL4R, KLRC4-KLRK1, AKT1, CD80, ITCH, IGLC3, IGHA1, MIR17HG, CCDC88B, PDCD1, XBP1, LILRB4, GPNMB, CD300A, IGHG1, SRC, DUSP10, DLG1, SOD1, IGHV4-34, PRDM1, PKN1, CLCF1, TBC1D10C, TNFSF13B, TIGIT, TMEM131L, IGHV3-20, IGHV1-24, CEBPB, LILRB2, SFRP1, IGHV2-5, SIRPG, PRKCQ, LAX1, FYN, BTLA, PELI1, TICAM1, BRAF, IL13, ZFP36L1, PRELID1, TFRC, TIRAP | 100 |
| 19 | GO:0006958 | complement activation, classical pathway | IGLL1, IGHV3-33, IGHG4, IGLC6, IGLV2-23, CR1, IGHV4-28, IGHV4-39, C8G, IGHV5-51, IGHV3-7, IGLV3-25, IGHV1-45, IGHE, TRBC2, IGHV3-48, IGLV2-8, IGHG2, IGHD, IGLV2-11, IGHV3-43, IGHV1-58, APCS, C1QBP, IGLV3-1, IGHV2-70, IGHM, C6, C4BPB, IGLV1-51, IGHV3-49, CRP, IGHV4-61, IGHV4-59, C4BPA, C1QC, IGHV3-30, IGLC1, IGHV1-3, IGHV4-4, IGLV7-43, IGLV3-27, C1QB, IGHV2-26, IGLV3-19, CR2, IGHV3-15, IGLV1-40, C9, IGHV3-21, IGHV3-53, IGHG3, IGLC2, CD46, IGHA2, MASP2, IGHV3-11, SUSD4, IGLC7, CD55, IGHV3-66, IGHV3-73, IGHV3-13, IGHV1-18, IGLV2-14, IGLL5, IGHV1OR15-1, IGLV1-47, IGHV3-72, IGLV3-21, IGHV1-69, IGHV3-74, IGHV6-1, IGHV3-64, IGLV1-44, IGLC3, IGHA1, C1QA, IGHG1, IGLV6-57, C5, IGHV4-34, C7, IGHV3-20, IGHV1-24, IGHV2-5, CLU, IGHV3-23 | 100 |
| 19 | GO:0002449 | lymphocyte mediated immunity | CTSH, IGLL1, IGHV3-33, CD40LG, IGHG4, TUBB4B, IGLV2-23, IGLC6, FOXP3, CR1, NCR1, DENND1B, IGHV4-28, IGHV4-39, C8G, IGHV5-51, IGHV3-7, DUSP22, IGLV3-25, EXO1, MLH1, IGHV1-45, IGHE, IFNB1, CD1D, TRBC2, TNFSF4, TSTA3, IGHV3-48, IGLV2-8, SCART1, IGHG2, IGHD, IGLV2-11, IGHV3-43, BCL6, IL13RA2, IGHV1-58, TRAF2, APCS, SH2D1A, C1QBP, IGLV3-1, HMGB1, IGHV2-70, IGHM, FCGR2B, TRPM4, CRK, TNFSF13, C6, C4BPB, PPP3CB, XCL1, MAD2L2, IGLV1-51, IGHV3-49, CRP, SHLD2, FCER1G, IGHV4-61, THOC1, IL4, INPP5D, IGHV4-59, KLRK1, LEP, RAB27A, C4BPA, IL6, IGHV3-30, C1QC, RIF1, KLRF2, IGLC1, FADD, APLF, CLEC12B, IGHV1-3, LIG4, IGHV4-4, GZMM, IGLV7-43, IFNA2, IGLV3-27, C1QB, IGHV2-26, NLRP3, IGLV3-19, PAXIP1, SERPINB4, CR2, MYO1G, HSPD1, IGHV3-15, IGLV1-40, CD28, C9, IGHV3-21, LAMP1, CORO1A, IL20RB, IGHV3-53, WAS, IL23R, LILRB1, IGHG3, CD1A, IGLC2, CD46, IGHA2, IL2, MASP2, IGHV3-11, CD1C, SUSD4, IL10, IGLC7, CD55, MALT1, IGHV3-66, IGHV3-73, PRKCZ, PIK3R6, LYST, BTK, CD1B, IL1R1, IL21, AP1G1, IGHV3-13, GAPT, IGHV1-18, PTPRC, SLAMF6, KIR3DL1, GATA3, IGLV2-14, CD1E, EMP2, IGLL5, KLRD1, IGHV1OR15-1, CD96, IGLV1-47, IGHV3-72, ATAD5, NSD2, IGLV3-21, MSH6, SASH3, IGHV1-69, SLA2, CCR6, RSAD2, NBN, BCL10, SERPINB9, IL12B, IL12A, RNF168, KDELR1, IGHV3-74, IGHV6-1, IGHV3-64, MSH2, KDM5D, SLAMF7, IL18RAP, NDFIP1, IL4R, KLRC4-KLRK1, IGLV1-44, IGLC3, IGHA1, FZD5, IL18R1, C1QA, IGHG1, HPRT1, IGLV6-57, DLG1, CD226, C5, IL31RA, KLRC2, IGHV4-34, C7, CLCF1, CLEC2A, IGHV3-20, IGHV1-24, IGHV2-5, RFTN1, CLU, IGHV3-23, TFRC | 100 |
| 19 | GO:0050851 | antigen receptor-mediated signaling pathway | TESPA1, IGLL1, IGHV3-33, RAB29, IGHG4, IGLC6, PVRIG, PAG1, FOXP3, CSK, KLHL6, DENND1B, IGHV4-28, IGHV4-39, BLK, DUSP22, IGHV5-51, IGHV3-7, PLCG2, PAK2, IKBKG, IGHV1-45, IGHE, TRBC2, IGHV3-48, CTLA4, HRAS, PTPN2, LCP2, IGHG2, IGHD, NFAM1, RBCK1, IGHV3-43, RELA, CD247, IGHV1-58, PAWR, CARD11, IGHV2-70, IGHM, GCSAM, MAPK1, ELF2, PHPT1, SLC39A10, LAT2, PDE4D, IGHV3-49, CD276, PAK3, IGHV4-61, INPP5D, TXK, IGHV4-59, CMTM3, MNDA, LIME1, TRAT1, BTNL9, PIK3CD, IKBKB, IGHV3-30, IGLC1, PAX5, IGHV3-23, PDPK1, IGHV1-3, IGHV4-4, RIPK2, ERMAP, IGHV2-26, SH2B2, DUSP3, CBFB, HHLA2, IGHV3-15, CD28, PLEKHA1, IGHV3-21, EZR, IGHV3-53, WAS, CACNA1F, BTNL3, LAT, GCSAML, RC3H2, IGHG3, IGLC2, PTPRJ, NFATC2, IGHA2, ABL1, IGHV3-11, FYB1, PLCL2, RC3H1, IGLC7, STAP1, MALT1, IGHV3-66, IGHV3-73, BCL2, STOML2, BTK, MEF2C, PLCG1, IGHV3-13, LGALS3, PDE4B, IGHV1-18, PTPRC, BTNL8, GATA3, IGLL5, FCRL3, IGHV1OR15-1, IGHV3-72, GPS2, IGHV1-69, BTNL10, CD38, BCL10, NCKAP1L, PIK3CB, GRAP2, IGHV3-74, IGHV6-1, IGHV3-64, NCK1, THEMIS2, IGLC3, IGHA1, LILRB4, CD300A, IGHG1, CD226, IGHV4-34, STK11, BAX, TEC, IGHV3-20, IGHV1-24, IGHV2-5, ELF1, CD19, PRKCQ, FYN, RFTN1, BRAF, SLA2, EIF2B5 | 100 |
| 19 | GO:0002431 | Fc receptor mediated stimulatory signaling pathway | IGHV3-33, IGHG4, IGLC6, IGLV2-23, CSK, DOCK1, IGHV4-39, VAV2, IGHV3-7, PLCG2, IGLV3-25, WASL, IGHV3-48, IGLV2-8, IGHG2, IGLV2-11, ACTB, CD247, PLA2G6, PRKCE, IGLV3-1, IGHV2-70, FCGR2B, MAPK1, CRK, WASF2, GRB2, ARPC5, IGLV1-51, ARPC4, CD47, FCER1G, RAPGEF1, RAP1A, IGHV4-59, CYFIP1, IGHV3-30, HSP90AA1, MYO1C, ARPC1B, IGLC1, IGLV7-43, IGLV3-27, IGLV3-19, MYO1G, RAC1, IGLV1-40, FCGR1A, YES1, IGHV3-53, WAS, FGR, PLSCR1, HCK, ACTR2, IGHG3, FCGR2A, PTPRJ, ABL1, IGHV3-11, IGLC7, ELMO1, PTK2, IGHV3-13, PTPRC, WIPF1, IGLV2-14, IGHV1OR15-1, IGLV1-47, IGLV3-21, BRK1, ABI1, IGHV1-69, NCKAP1L, PIK3CB, MAPK3, MYO10, NCK1, ARPC1A, IGLV1-44, IGHG1, IGLV6-57, SRC, CD226, IGHV4-34, WIPF3, FCGR3A, IGHV2-5, NCKAP1, NR4A3, FYN, LIMK1, IGHV3-23 | 100 |
| 19 | GO:0002433 | immune response-regulating cell surface receptor signaling pathway involved in phagocytosis | IGHV3-33, IGHG4, IGLC6, IGLV2-23, DOCK1, IGHV4-39, VAV2, IGHV3-7, PLCG2, IGLV3-25, WASL, IGHV3-48, IGLV2-8, IGHG2, IGLV2-11, ACTB, CD247, PLA2G6, PRKCE, IGLV3-1, IGHV2-70, FCGR2B, MAPK1, CRK, WASF2, GRB2, ARPC5, IGLV1-51, ARPC4, CD47, RAPGEF1, RAP1A, IGHV4-59, CYFIP1, IGHV3-30, HSP90AA1, MYO1C, ARPC1B, IGLC1, IGLV7-43, IGLV3-27, IGLV3-19, MYO1G, RAC1, IGLV1-40, FCGR1A, YES1, IGHV3-53, WAS, FGR, HCK, ACTR2, IGHG3, FCGR2A, PTPRJ, ABL1, IGHV3-11, IGLC7, ELMO1, PTK2, IGHV3-13, PTPRC, WIPF1, IGLV2-14, IGHV1OR15-1, IGLV1-47, IGLV3-21, BRK1, ABI1, IGHV1-69, NCKAP1L, PIK3CB, MAPK3, MYO10, NCK1, ARPC1A, IGLV1-44, IGHG1, IGLV6-57, SRC, IGHV4-34, WIPF3, FCGR3A, IGHV2-5, NCKAP1, FYN, LIMK1, IGHV3-23 | 100 |
| 19 | GO:0042113 | B cell activation | POLM, IGLL1, IGHV3-33, CD40LG, IGHG4, IGLC6, FOXP3, HDAC4, IGHV4-28, IGHV4-39, IFNA5, IGHV5-51, IGHV3-7, PLCG2, CHRNA4, EXO1, MLH1, IGHV1-45, IGHE, IFNB1, HHEX, TRBC2, TNFSF4, PCID2, IGHV3-48, CTLA4, PTPN2, IGHG2, IGHD, NFAM1, IGHV3-43, INHBA, BCL6, IGHV1-58, PAWR, CARD11, IFNA14, IRF2BP2, IGHV2-70, IGHM, TNFSF13, FCGR2B, AHR, TNFRSF13B, IL7, VCAM1, MAD2L2, LAT2, SLC39A10, IGHV3-49, SHLD2, IFNA6, IFNA4, IFNA10, IGHV4-61, THOC1, HDAC9, IFNA13, IL4, AKAP17A, INPP5D, IGHV4-59, MNDA, PIK3CD, IFNE, IL6, IGHV3-30, GPR183, RIF1, PPP2R3C, IGLC1, APLF, FZD9, IGHV3-23, BAD, IFNA16, IGHV1-3, GON4L, LIG4, TNFRSF13C, IGHV4-4, FCRL1, IFNA2, EP300, IGHV2-26, IRS2, PAXIP1, CR2, HSPD1, IGHV3-15, ITM2A, CD28, IFNA1, IGHV3-21, ONECUT1, LYL1, IGHV3-53, IL5, FNIP1, CDH17, MIF, IGHG3, CASP8, IGLC2, PTPRJ, NFATC2, IGHA2, IL2, TPD52, IGHV3-11, IL10, ABL1, PLCL2, RC3H1, IGLC7, MALT1, IGHV3-66, IGHV3-73, TNIP2, LGALS1, BCL2, LRRC8A, ZFP36L2, CASP3, DLL1, BTK, ITGA4, IL21, IFNA17, MEF2C, ATP11C, IGHV3-13, GAPT, IGHV1-18, PTPRC, IFNA7, LFNG, IGLL5, FCRL3, IGHV1OR15-1, IGHV3-72, IFNA21, ATAD5, NSD2, GPS2, MSH6, SASH3, PTK2B, IGHV1-69, SLA2, CCR6, NTRK1, CD38, NBN, TNFRSF4, NCKAP1L, WNT3A, SKAP2, RNF168, IGHV3-74, IGHV6-1, BST1, IGHV3-64, MSH2, MFNG, IFNK, NDFIP1, HDAC5, DNAJB9, TCF3, BAK1, KLF6, IFNA8, IGLC3, IGHA1, MIR17HG, SP3, XBP1, ZBTB7A, CD300A, IGHG1, IGHV4-34, TBC1D10C, PKN1, CLCF1, TNFSF13B, BAX, IGHV3-20, IGHV1-24, SFRP1, IGHV2-5, DOCK11, LAX1, PELI1, TICAM1, DCLRE1C, ITGB1, IFNW1, IL13, ZFP36L1, TFRC, TIRAP | 100 |
| 19 | GO:0006911 | phagocytosis, engulfment | IGHV3-53, FCER1G, IGLL1, GSN, IGHV3-33, IGHV1-69, IGHV4-61, IGHG4, IGLC6, NCKAP1L, IGHV4-59, SH3BP1, DOCK1, IGHV4-28, IGHV4-39, MSR1, IGHG3, IGHV5-51, IGHV3-7, MEGF10, CD36, ARHGAP25, IGLC2, MYH9, IGHA2, IGHV3-74, IGHV6-1, IGHV3-11, IGHV3-30, IGHE, IGHV1-45, IGHV3-64, TRBC2, ITGAM, IGLC7, IGLC1, IGHV3-48, STAP1, ABCA7, IGHV3-66, IGHV3-73, MFGE8, ADGRB1, IGHG2, IGHD, ITGB2, IGLC3, IGHA1, IGHV1-3, IGHV3-43, CD300A, IGHG1, IGHV4-4, ELMO1, IGHV1-58, IGHV3-13, IGHV4-34, IGHV1-18, IGHV2-26, ARHGAP12, GULP1, IGHV2-70, IGHM, FCGR2B, IGHV3-20, IGHV1-24, XKR8, F2RL1, IGLL5, IGHV2-5, PPARG, IGHV1OR15-1, IGHV3-15, IGHV3-21, IGHV3-72, FCGR1A, IGHV3-23, RHOBTB2, IGHV3-49 | 100 |
| 19 | GO:0006909 | phagocytosis | SYT11, IGLL1, IGHV3-33, SIRPB1, GAS6, IGHG4, IGLC6, IGLV2-23, CSK, MSR1, SH3BP1, DOCK1, IGHV4-28, IGHV4-39, IRF8, VAV2, IGHV5-51, IGHV3-7, PLCG2, PEAR1, ELMO3, IGLV3-25, SPON2, MESD, RUBCN, IGHV1-45, IGHE, TRBC2, PRTN3, WASL, IGHV3-48, IGLV2-8, IGHG2, IGHD, IGLV2-11, ACTB, ATG5, AZU1, IGHV3-43, EIF2AK1, CD247, IGHV1-58, PLA2G6, ANXA1, PRKCG, IGLV3-1, HMGB1, TLR2, IGHV2-70, IGHM, FCGR2B, MAPK1, CRK, WASF2, LMAN2, GRB2, ARPC5, C4BPB, ATG3, PPARG, PTX3, IGLV1-51, IGHV3-49, ARPC4, CRP, PYCARD, CD47, FCER1G, NCF4, RAPGEF1, IGHV4-61, RAP1A, ITGAL, IGHV4-59, LEP, CD36, RAB27A, CYFIP1, C4BPA, SFTPD, FCN1, SLAMF1, LYAR, IGHV3-30, HSP90AA1, MYO1C, ARPC1B, RAB14, IGLC1, MERTK, ITGB2, CAMK1D, IGHV1-3, LBP, RACK1, IGHV4-4, COLEC12, ANXA11, CLEC7A, TMEM175, IGLV7-43, ABR, IGLV3-27, PTEN, IGHV2-26, ARHGAP12, IGLV3-19, ADORA1, TM9SF4, MYO1G, SIRPA, RAC1, IGHV3-15, IGLV1-40, LEPR, IGHV3-21, FCGR1A, ANXA3, RHOBTB2, CORO1A, YES1, IGHV3-53, WAS, GSN, FGR, DOCK2, HCK, FCN2, ACTR2, IGHG3, MEGF10, FCGR2A, IGLC2, PTPRJ, IGHA2, ABL1, IGHV3-11, ITGAV, BCR, CALR, IGLC7, STAP1, CDC42SE2, IGHV3-66, IGHV3-73, MIR17, CD14, CYBA, DYSF, ELMO1, PTK2, RAB20, IGHV3-13, IGHV1-18, PTPRC, WIPF1, ADIPOQ, IGLV2-14, IGLL5, IGHV1OR15-1, AHSG, IGLV1-47, CD302, FCN3, IGHV3-72, MIR20A, IGLV3-21, BRK1, ABI1, IGHV1-69, PLD4, NCKAP1L, PIK3CB, ADGRB1, ARHGAP25, MAPK3, MYO10, MYH9, PIP5K1C, IGHV3-74, IGHV6-1, IGHV3-64, NCK1, ARPC1A, ITGAM, ABCA7, MFGE8, IGLV1-44, NR1H3, IGLC3, IGHA1, MET, PRKCE, TGM2, CD300A, IGHG1, IGLV6-57, SRC, ELANE, IGHV4-34, GULP1, WIPF3, IGHV3-20, IGHV1-24, NCF2, XKR8, FCGR3A, F2RL1, IGHV2-5, NCKAP1, SIRPG, FYN, LIMK1, ITGB1, IGHV3-23 | 100 |
| 19 | GO:0002696 | positive regulation of leukocyte activation | CD83, TESPA1, IGLL1, SHH, IGHV3-33, EGR3, CD40LG, SIRPB1, GAS6, IGHG4, IGLC6, FOXP3, CSK, TNFSF11, IGHV4-28, IGHV4-39, TMIGD2, IGHV5-51, IGHV3-7, PAK2, EFNB2, AP3D1, MLH1, IGHV1-45, IGHE, CD1D, TRBC2, TNFSF4, PCID2, IGHV3-48, CTLA4, IGHG2, IGHD, CAV1, DOCK8, IGHV3-43, TNFRSF14, CLECL1, DNAJA3, BCL6, IGHV1-58, CARD11, ANXA1, HMGB1, IGHV2-70, IGHM, TNFSF13, GRB2, IL7, TAC1, XCL1, VCAM1, PTAFR, ICOS, MAD2L2, SLC39A10, IGHV3-49, SHLD2, PYCARD, CD274, ITPKB, FCER1G, CD276, CD47, PAK3, IGHV4-61, DPP4, IL4, INPP5D, IGHV4-59, KLRK1, MIR30B, LEP, MIR21, SART1, IL6, IGHV3-30, GLI3, GPR183, RIF1, PPP2R3C, IL6ST, FADD, IGLC1, IGHV3-23, BAD, ITGB2, CRLF2, PDPK1, IGHV1-3, FLT3LG, TNFRSF13C, LBP, FGF10, IGHV4-4, ADAM8, RIPK2, IGHV2-26, NLRP3, IRS2, PAXIP1, IL1RL1, HSPD1, CBFB, SIRPA, HHLA2, RAC1, STXBP1, IGHV3-15, CD28, IGHV3-21, LAMP1, YES1, CORO1A, MAP3K8, IGHV3-53, PDCD1LG2, IL5, FGR, IL23R, ADORA2B, LILRB1, MIF, GPAM, IGHG3, IGLC2, CD46, NFATC2, IGHA2, IL2, IL10, IGHV3-11, EBI3, SOCS5, ZP4, HES1, NKAP, IGLC7, CD55, STAP1, MALT1, IGHV3-66, IGHV3-73, TNIP2, LGALS1, PRKCZ, BCL2, BTK, IL21, MEF2C, ATP11C, AP1G1, IL2RA, IGHV3-13, IGHV1-18, PTPRC, GATA3, HSPH1, SPTA1, IL1RL2, IGLL5, FCRL3, IGHV1OR15-1, CD86, IGHV3-72, HLX, ATAD5, NSD2, MSH6, NPPA, SASH3, IGHV1-69, EPO, CD38, BCL10, TNFRSF4, NCKAP1L, WNT3A, IL12B, IL12A, GRAP2, EFNB3, IGHV3-74, IGHV6-1, SOCS1, BST1, IGHV3-64, MSH2, NCK1, RASAL3, ITGAM, IL15, IL4R, KLRC4-KLRK1, AKT1, CD80, IGLC3, IGHA1, CCDC88B, PDCD1, XBP1, LILRB4, IGHG1, SRC, DUSP10, CD226, IGHV4-34, IL33, CLCF1, STX4, TNFSF13B, IGHV3-20, IGHV1-24, LILRB2, F2RL1, IGHV2-5, SIRPG, PRKCQ, NR4A3, FYN, BTLA, PELI1, TICAM1, IL13, TFRC, TIRAP | 100 |
| 19 | GO:0002460 | adaptive immune response based on somatic recombination of immune receptors built from immunoglobulin superfamily domains | CTSH, IGLL1, IGHV3-33, CXCL13, CD40LG, IGHG4, IGLC6, IGLV2-23, FOXP3, CR1, KLHL6, IRF4, DENND1B, IGHV4-28, IGHV4-39, C8G, IGHV5-51, IGHV3-7, DUSP22, IGLV3-25, EXO1, MLH1, IGHV1-45, IGHE, IFNB1, CD1D, TRBC2, TNFSF4, TSTA3, IGHV3-48, HRAS, IGLV2-8, SCART1, IGHG2, IGHD, IGLV2-11, IGHV3-43, BCL6, IL13RA2, IGHV1-58, TRAF2, APCS, ANXA1, IGLV3-1, HMGB1, C1QBP, IGHV2-70, IGHM, FCGR2B, TRPM4, TNFSF13, C6, C4BPB, PPP3CB, XCL1, MAD2L2, SPN, IGLV1-51, IGHV3-49, CRP, SHLD2, CD274, FCER1G, IGHV4-61, THOC1, IL4, INPP5D, IGHV4-59, RAB27A, C4BPA, MIR21, LY9, IL6, IGHV3-30, C1QC, RIF1, FADD, IGLC1, APLF, IGHV1-3, TNFRSF13C, LIG4, IGHV4-4, GZMM, IGLV7-43, IFNA2, IGLV3-27, RIPK2, IGHV2-26, NLRP3, IGLV3-19, C1QB, PAXIP1, IL1RL1, MTOR, CR2, MYO1G, HSPD1, IGHV3-15, IGLV1-40, SEMA4A, CD28, C9, IGHV3-21, IL20RB, IGHV3-53, WAS, IL23R, RC3H2, LILRB1, IGHG3, CD1A, IGLC2, CD46, SOCS5, IGHA2, IL2, MASP2, IGHV3-11, CD1C, SUSD4, IL10, EBI3, RC3H1, IL27, IGLC7, CD55, MALT1, IGHV3-66, IGHV3-73, PRKCZ, BTK, CD1B, IL1R1, MEF2C, IGHV3-13, GAPT, IGHV1-18, PTPRC, SLAMF6, GATA3, IGLV2-14, CD1E, EMP2, IGLL5, IGHV1OR15-1, IGLV1-47, IGHV3-72, HLX, ATAD5, NSD2, IGLV3-21, MSH6, SASH3, IGHV1-69, SLA2, CCR6, RSAD2, NBN, BCL10, IL12B, IL12A, RNF168, KDELR1, IGHV3-74, IGHV6-1, IGHV3-64, MSH2, KDM5D, IL18RAP, NDFIP1, IL4R, CD80, IGLV1-44, IGLC3, IGHA1, FZD5, IL18R1, C1QA, IGHG1, HPRT1, IGLV6-57, DLG1, CD226, C5, IL31RA, IGHV4-34, IL33, PKN1, C7, CLCF1, TNFSF13B, IGHV3-20, IGHV1-24, IGHV2-5, PRKCQ, RFTN1, CLU, IGHV3-23, TFRC | 100 |
| 19 | GO:0002526 | acute inflammatory response | SERPINC1, IGHV3-33, PARK7, IGHG4, IGLC6, IGLV2-23, CR1, TNFSF11, IGHV4-39, C8G, IGHV3-7, IGLV3-25, CFHR4, MIR92A2, TNFSF4, GSTP1, CFHR5, IGHV3-48, IGLV2-8, IGHG2, IGLV2-11, PIK3CG, EIF2AK1, APCS, C1QBP, IGLV3-1, CFHR2, OSMR, IGHV2-70, FCGR2B, C6, TAC1, C4BPB, VCAM1, PPARG, IGLV1-51, OPRM1, CPB2, CRP, FCER1G, IGHV4-59, CFHR1, NUPR1, C4BPA, KLKB1, TNFRSF11A, IGHV3-30, C1QC, IL6, IL6ST, IGLC1, LBP, SERPINF2, F12, ADAM8, IGLV7-43, IGLV3-27, RHBDD3, C1QB, ADORA1, IGLV3-19, NLRP3, NPFF, CR2, IGLV1-40, C9, CFH, IL20RB, IGHV3-53, SELENOS, PTGES, F8, B4GALT1, PLSCR1, CPN2, MIR92A1, CTNNBIP1, IGHG3, TFR2, CD46, IGHV3-11, SUSD4, UGT1A1, IGLC7, CD55, OSM, CREB3L3, DNASE1, BTK, ADCYAP1, FN1, IGHV3-13, GATA3, IGLV2-14, HP, IGHV1OR15-1, AHSG, IGLV1-47, TRPV1, DNASE1L3, A2M, IGLV3-21, ASS1, IGHV1-69, EPO, EDNRB, ASH1L, PTGS2, PTGER3, IGLV1-44, C1QA, IGHG1, IGLV6-57, ELANE, C5, IL31RA, APOA2, IGHV4-34, HPR, C7, OGG1, CEBPB, APOL2, IGHV2-5, CD19, CLU, IGHV3-23 | 75 |
| 19 | GO:0038094 | Fc-gamma receptor signaling pathway | IGHV3-33, IGHG4, IGLC6, IGLV2-23, DOCK1, IGHV4-39, VAV2, IGHV3-7, PLCG2, IGLV3-25, WASL, IGHV3-48, IGLV2-8, IGHG2, IGLV2-11, ACTB, CD247, PLA2G6, PRKCE, IGLV3-1, IGHV2-70, FCGR2B, MAPK1, CRK, WASF2, GRB2, ARPC5, IGLV1-51, ARPC4, CD47, FCER1G, RAPGEF1, RAP1A, IGHV4-59, CYFIP1, IGHV3-30, HSP90AA1, MYO1C, ARPC1B, IGLC1, IGLV7-43, IGLV3-27, IGLV3-19, MYO1G, RAC1, IGLV1-40, FCGR1A, YES1, IGHV3-53, WAS, FGR, HCK, ACTR2, IGHG3, FCGR2A, PTPRJ, ABL1, IGHV3-11, IGLC7, ELMO1, PTK2, IGHV3-13, PTPRC, WIPF1, IGLV2-14, IGHV1OR15-1, IGLV1-47, IGLV3-21, BRK1, ABI1, IGHV1-69, NCKAP1L, PIK3CB, MAPK3, MYO10, NCK1, ARPC1A, IGLV1-44, IGHG1, IGLV6-57, SRC, IGHV4-34, WIPF3, FCGR3A, IGHV2-5, NCKAP1, FYN, LIMK1, IGHV3-23 | 100 |
| 19 | GO:0006959 | humoral immune response | CD83, IGLL1, IGHV3-33, CXCL13, ROMO1, IGHG4, IGLC6, IGLV2-23, CR1, IGHV4-28, IGHV4-39, IFNA5, C8G, IGHV5-51, IGHV3-7, DEFB127, DEFA3, KRT1, IGLV3-25, EXO1, SPON2, CHGA, CFHR4, IGHV1-45, IGHE, IFNB1, TRBC2, DEFB1, CFHR5, DEFA4, PRTN3, IGHV3-48, DEFB126, IGLV2-8, IGHG2, IGHD, IGLV2-11, MUC7, AZU1, IGHV3-43, IGHV1-58, DCD, APCS, SH2D1A, PLA2G6, C1QBP, IGLV3-1, IFNA14, KLK3, CFHR2, IGHV2-70, IGHM, FCGR2B, ITLN1, DEFB4A, C6, BPI, IL7, C4BPB, PGLYRP2, BPIFA1, IGLV1-51, HTN1, ST6GAL1, CPB2, IGHV3-49, SCARA3, CRP, IFNA6, MNX1, IFNA4, IFNA10, DEFA5, IGHV4-61, IFNA13, BPIFA2, IGHV4-59, CFHR1, C4BPA, SFTPD, FCN1, IFNE, HTN3, DEFA6, IGHV3-30, GPR183, IL6, C1QC, PPP2R3C, IGLC1, PAX5, YTHDF2, JCHAIN, HRG, IFNA16, IGHV1-3, IGHV4-4, IGLV7-43, IFNA2, IGLV3-27, C1QB, IGHV2-26, RPL39, IGLV3-19, FAM3A, KLK5, DEFA1, CR2, BPIFB2, DEFB118, IGHV3-15, IGLV1-40, CD28, C9, IFNA1, IGHV3-21, CFH, ACOD1, RGCC, IGHV3-53, CFD, CPN2, FCN2, PRSS3, IGHG3, FGA, LCN2, IGLC2, CD46, IGHA2, MASP2, TFE3, IGHV3-11, SUSD4, EBI3, ZP4, IGLC7, CD55, IGHV3-66, IGHV3-73, NOTCH1, DEFB103B, BCL2, DEFA1B, IFNA17, MEF2C, IGHV3-13, LEAP2, IGHV1-18, PTPRC, COL20A1, IFNA7, GATA3, MASP1, IGLV2-14, DEFB103A, IGLL5, TRAF3IP2, IGHV1OR15-1, KLK7, IGLV1-47, FCN3, DMBT1, IGHV3-72, IFNA21, A2M, IGLV3-21, IGHV1-69, CCR6, RNASE7, RARRES2, IGHV3-74, IGHV6-1, BST1, IGHV3-64, FGB, IFNK, COLEC10, VSIG4, IGLV1-44, RNASE6, IFNA8, IGLC3, IGHA1, PLA2G2A, PDCD1, C1QA, IGHG1, IGLV6-57, ELANE, C5, IGHV4-34, C7, RNASE3, IGHV3-20, IGHV1-24, IGHV2-5, BPIFB1, CD19, CLU, IFNW1, IGHV3-23 | 100 |
| 19 | GO:0070613 | regulation of protein processing | IGHV3-33, IGHG4, IGLC6, IGLV2-23, CR1, IGHV4-39, C8G, MELTF, IGHV3-7, IGLV3-25, CFHR4, PRSS37, CFHR5, IGHV3-48, IGLV2-8, IGHG2, IGLV2-11, USP17L2, NKD2, NOL3, C1QBP, IGLV3-1, CFHR2, IGHV2-70, C6, C4BPB, ACP4, IGLV1-51, CPB2, PRKACB, CNTN2, IGHV4-59, CFHR1, C4BPA, KLKB1, C1QC, IGHV3-30, IGLC1, SERPINF2, ANXA2P2, F12, ADAM8, IGLV7-43, IGLV3-27, C1QB, IGLV3-19, CR2, XIAP, IGLV1-40, C9, PLGRKT, CFH, TMEM59, IGHV3-53, GSN, CPN2, IGHG3, CD46, IGHV3-11, SUSD4, ENO1, IGLC7, CD55, TIMM17A, CARD8, IGHV3-13, IGLV2-14, CTSZ, IGHV1OR15-1, SERPINE2, IGLV1-47, ANGPTL8, A2M, IGLV3-21, IGHV1-69, MYH9, SERPINE1, NLRP7, BIRC7, IGLV1-44, BCL2L12, C1QA, IGHG1, IGLV6-57, SRC, C5, IGHV4-34, C7, IL1R2, IGHV2-5, CD19, CLU, TNP2, IGHV3-23, CCBE1, GLG1 | 100 |
| 19 | GO:0042742 | defense response to bacterium | SYT11, IGLL1, IGHV3-33, CXCL13, CARD9, ROMO1, IGHG4, IGLC6, IGHV4-28, IGHV4-39, IRF8, DEFA3, IGHV5-51, IGHV3-7, DEFB127, UNC13B, DEFB115, SPON2, CHGA, DEFB135, IGHV1-45, IGHE, TRBC2, DEFB1, DEFA4, IGHV3-48, DEFB126, IGHG2, IGHD, GSDMD, DEFB121, SIGLEC11, IGHV3-43, AZU1, TNFRSF14, HMGB2, IGHV1-58, DCD, DEFB116, PLA2G6, KLK3, TLR2, IGHV2-70, IGHM, DEFB136, DEFB4A, BPI, ISG15, TIRAP, PGLYRP2, DEFB132, C10orf99, BPIFA1, DEFB125, SPN, DEFB106B, HTN1, LYZL2, DEFB131A, IGHV3-49, CRP, PYCARD, FCER1G, DEFA5, IGHV4-61, BPIFA2, IGHV4-59, KLRK1, CD36, EPX, SFTPD, IFNE, DEFB104A, DEFB124, HTN3, DEFA6, IGHV3-30, IL6, NLRP1, RAB14, MR1, MAVS, IGLC1, JCHAIN, DEFB104B, DEFB108A, DEFB119, IGHV1-3, FOXP1, LBP, IGHV4-4, MPO, NOD1, RIPK2, DEFB128, IGHV2-26, NLRP3, RPL39, DEFB106A, DEFA1, KLK5, DEFB105A, DEFB134, DEFB118, IGHV3-15, LYZL1, IGHV3-21, ANXA3, IGHV3-53, FGR, IL23R, FCN2, RNASE8, IGHG3, FGA, IGLC2, IGHA2, SPAG11A, IL10, IGHV3-11, DEFB129, NR1H4, IGLC7, LYPD8, IGHV3-66, IGHV3-73, TLR3, SELP, DEFB103B, DEFA1B, CYBA, LYST, DEFB130A, DEFB109B, IGHV3-13, LEAP2, IGHV1-18, DEFB123, PRB3, F2, DEFB103A, HP, OPTN, IGLL5, IGHV1OR15-1, DEFB130B, IL22RA1, KLK7, DMBT1, IGHV3-72, LPO, SIGLEC16, PLAC8, IGHV1-69, RNASE7, ADGRB1, IL12B, IL12A, RARRES2, DEFB105B, STATH, IGHV3-74, IGHV6-1, IGHV3-64, FGB, SERPINE1, VGF, KLRC4-KLRK1, RNASE6, TLR5, IGLC3, IGHA1, PLA2G2A, ACP5, SPAG11B, IGHG1, ELANE, IGHV4-34, TMF1, RNASE3, IGHV3-20, IGHV1-24, CEBPB, F2RL1, TBK1, IGHV2-5, LACRT, IGHV3-23, EPHA2, RAB1A | 100 |
| 19 | GO:0006956 | complement activation | IGLL1, IGHV3-33, IGHG4, IGLC6, IGLV2-23, CR1, IGHV4-28, IGHV4-39, C8G, IGHV5-51, IGHV3-7, KRT1, IGLV3-25, CFHR4, IGHV1-45, IGHE, TRBC2, CFHR5, IGHV3-48, IGLV2-8, IGHG2, IGHD, IGLV2-11, IGHV3-43, IGHV1-58, APCS, C1QBP, IGLV3-1, CFHR2, IGHV2-70, IGHM, C6, C4BPB, IGLV1-51, CPB2, IGHV3-49, SCARA3, CRP, IGHV4-61, IGHV4-59, CFHR1, C4BPA, FCN1, C1QC, IGHV3-30, IGLC1, IGHV1-3, IGHV4-4, IGLV7-43, IGLV3-27, C1QB, IGHV2-26, IGLV3-19, CR2, IGHV3-15, IGLV1-40, C9, CFH, IGHV3-21, RGCC, IGHV3-53, CFD, CPN2, FCN2, IGHG3, IGLC2, CD46, IGHA2, MASP2, IGHV3-11, SUSD4, IGLC7, CD55, IGHV3-66, IGHV3-73, IGHV3-13, IGHV1-18, COL20A1, MASP1, IGLV2-14, IGLL5, IGHV1OR15-1, IGLV1-47, FCN3, IGHV3-72, A2M, IGLV3-21, IGHV1-69, IGHV3-74, IGHV6-1, IGHV3-64, COLEC10, VSIG4, IGLV1-44, IGLC3, IGHA1, C1QA, IGHG1, IGLV6-57, C5, IGHV4-34, C7, IGHV3-20, IGHV1-24, IGHV2-5, CD19, CLU, IGHV3-23 | 100 |
| 19 | GO:0038095 | Fc-epsilon receptor signaling pathway | IGHV3-53, IGLV3-21, FCER1G, IGHV3-33, IGHV1-69, IGLC6, IGLV2-23, LAT, BCL10, IGHV4-59, FCER1A, PIK3CB, IGHV4-39, VAV2, IGHV3-7, PLCG2, LILRA4, MAPK3, IKBKB, IGLV3-25, GRAP2, NFATC2, IKBKG, PAK2, IGHV3-11, IGHV3-30, IGHE, IGLC7, IGLC1, IGHV3-48, MALT1, IGLV2-8, IGLV1-44, LCP2, IGLV2-11, PPP3R1, PDPK1, NFATC1, BTK, RELA, IGLV6-57, IGLV7-43, IGHV3-13, IGLV3-27, IGHV4-34, IGLV3-1, CARD11, IGLV3-19, IGHV2-70, MAPK1, TEC, GRB2, IGLV2-14, PPP3CB, IGHV2-5, RAC1, IGHV1OR15-1, NR4A3, IGLV1-47, IGLV1-40, PRKCQ, TAB1, MAPK9, LAT2, IGLV1-51, IGHV3-23, MAP3K1, MAPK10 | 100 |
| 19 | GO:0051251 | positive regulation of lymphocyte activation | CD83, TESPA1, IGLL1, SHH, IGHV3-33, EGR3, CD40LG, SIRPB1, GAS6, IGHG4, IGLC6, FOXP3, CSK, TNFSF11, IGHV4-28, IGHV4-39, TMIGD2, IGHV5-51, IGHV3-7, PAK2, EFNB2, AP3D1, MLH1, IGHV1-45, IGHE, CD1D, TRBC2, TNFSF4, PCID2, IGHV3-48, CTLA4, IGHG2, IGHD, CAV1, DOCK8, IGHV3-43, TNFRSF14, CLECL1, DNAJA3, BCL6, IGHV1-58, CARD11, ANXA1, HMGB1, IGHV2-70, IGHM, TNFSF13, GRB2, IL7, TAC1, XCL1, VCAM1, ICOS, MAD2L2, SLC39A10, IGHV3-49, SHLD2, PYCARD, CD274, ITPKB, CD276, CD47, PAK3, IGHV4-61, DPP4, IL4, INPP5D, IGHV4-59, KLRK1, MIR30B, LEP, MIR21, SART1, IL6, IGHV3-30, GLI3, GPR183, RIF1, PPP2R3C, IL6ST, FADD, IGLC1, IGHV3-23, BAD, PDPK1, IGHV1-3, FLT3LG, TNFRSF13C, FGF10, IGHV4-4, ADAM8, RIPK2, IGHV2-26, NLRP3, IRS2, PAXIP1, HSPD1, CBFB, SIRPA, HHLA2, RAC1, IGHV3-15, CD28, IGHV3-21, LAMP1, YES1, CORO1A, MAP3K8, IGHV3-53, PDCD1LG2, IL5, IL23R, LILRB1, MIF, GPAM, IGHG3, IGLC2, NFATC2, CD46, IGHA2, IL2, EBI3, IGHV3-11, SOCS5, ZP4, HES1, NKAP, IGLC7, CD55, MALT1, IGHV3-66, IGHV3-73, TNIP2, LGALS1, PRKCZ, BCL2, BTK, IL21, MEF2C, ATP11C, AP1G1, IL2RA, IGHV3-13, IGHV1-18, PTPRC, GATA3, HSPH1, SPTA1, IL1RL2, IGLL5, FCRL3, IGHV1OR15-1, CD86, IGHV3-72, HLX, ATAD5, NSD2, MSH6, SASH3, IGHV1-69, EPO, CD38, BCL10, TNFRSF4, NCKAP1L, WNT3A, IL12B, IL12A, GRAP2, EFNB3, IGHV3-74, IGHV6-1, SOCS1, BST1, IGHV3-64, MSH2, NCK1, RASAL3, IL15, IL4R, KLRC4-KLRK1, AKT1, CD80, IGLC3, IGHA1, CCDC88B, PDCD1, XBP1, LILRB4, IGHG1, SRC, DUSP10, IGHV4-34, CLCF1, TNFSF13B, IGHV3-20, IGHV1-24, LILRB2, IGHV2-5, SIRPG, PRKCQ, FYN, BTLA, PELI1, TICAM1, IL13, TFRC, TIRAP | 100 |
| 19 | GO:0002768 | immune response-regulating cell surface receptor signaling pathway | TESPA1, IGLL1, IGHV3-33, CARD9, FPR1, RAB29, IGHG4, IGLC6, IGLV2-23, PVRIG, PAG1, CR1, CSK, KLHL6, FOXP3, DOCK1, IGHV4-28, IGHV4-39, DENND1B, DUSP22, IGHV5-51, IGHV3-7, VAV2, PLCG2, BLK, IGLV3-25, PAK2, IKBKG, IGHV1-45, IGHE, TRBC2, WASL, IGHV3-48, CTLA4, HRAS, IGLV2-8, PTPN2, CLEC10A, IGHG2, IGHD, NFAM1, IGLV2-11, LILRA2, ACTB, LCP2, RBCK1, MUC7, IGHV3-43, RELA, CD247, IGHV1-58, MUC17, FPR2, CARD11, PLA2G6, PRKCE, IGLV3-1, PAWR, IGHV2-70, IGHM, FCGR2B, MAPK1, CRK, WASF2, GCSAM, GRB2, ELF2, ARPC5, PPP3CB, PHPT1, SLC39A10, LAT2, IGLV1-51, IGHV3-49, PDE4D, ARPC4, MAPK10, CD276, CD47, FCER1G, PRKACB, PAK3, RAPGEF1, IGHV4-61, RAP1A, INPP5D, TXK, IGHV4-59, CREBBP, MNDA, LIME1, CMTM3, KLRK1, TRAT1, BTNL9, PIK3CD, LILRA4, CYFIP1, IKBKB, FCN1, FPR3, IGHV3-30, HSP90AA1, MYO1C, ARPC1B, IGLC1, PAX5, CLEC12B, PPP3R1, PDPK1, IGHV1-3, IGHV4-4, IGLV7-43, EP300, IGLV3-27, RIPK2, IGHV2-26, ERMAP, IGLV3-19, SH2B2, DUSP3, CR2, MYO1G, CBFB, HHLA2, RAC1, IGHV3-15, IGLV1-40, MUCL1, MUC4, PLEKHA1, CD28, IGHV3-21, FCGR1A, MUC3A, MAP3K1, EZR, YES1, IGHV3-53, WAS, CACNA1F, FGR, BTNL3, PLSCR1, LAT, HCK, GCSAML, RC3H2, LILRB1, ACTR2, IGHG3, FCGR2A, IGLC2, PTPRJ, NFATC2, IGHA2, GPR32, ABL1, IGHV3-11, FYB1, PLCL2, RC3H1, IGLC7, STAP1, MALT1, IGHV3-66, IGHV3-73, PRKACG, MUC12, BCL2, STOML2, BTK, ELMO1, MEF2C, RAF1, IGHV3-13, LGALS3, PDE4B, IGHV1-18, PTPRC, WIPF1, CD200R1, BTNL8, GATA3, IGLV2-14, GPR32P1, IGLL5, FCRL3, IGHV1OR15-1, IGLV1-47, TAB1, IGHV3-72, IGLV3-21, GPS2, BRK1, ABI1, IGHV1-69, SLA2, BTNL10, CD38, BCL10, FCER1A, NCKAP1L, PIK3CB, MAPK3, MYO10, GRAP2, IGHV3-74, IGHV6-1, IGHV3-64, NCK1, ARPC1A, THEMIS2, KLRC4-KLRK1, IGLV1-44, IGLC3, IGHA1, LILRB4, NFATC1, CD300A, IGHG1, IGLV6-57, KIR2DL1, CD226, SRC, PIGR, IGHV4-34, STK11, WIPF3, BAX, MUC20, IGHV3-20, IGHV1-24, MUC1, TEC, LILRB2, FCGR3A, IGHV2-5, ELF1, NCKAP1, CD19, PRKCQ, FYN, LIMK1, RFTN1, BTLA, BRAF, MAPK9, NR4A3, IGHV3-23, EIF2B5 | 100 |
| 19 | GO:0016485 | protein processing | SEC11C, CTSH, LGMN, SHH, IGHV3-33, IGHG4, IGLC6, IGLV2-23, KLK13, CR1, IGHV4-39, C8G, F9, IGHV3-7, MELTF, ASPH, IGLV3-25, CFHR4, IMMP1L, ASPRV1, PRSS37, CFHR5, IGHV3-48, IGLV2-8, IGHG2, KLK2, IGLV2-11, YME1L1, USP17L2, MME, HM13, IGLV3-1, KLK3, CFHR2, IGHV2-70, C6, C4BPB, PISD, PCSK4, CPXM1, ACP4, IGLV1-51, CPB2, PYCARD, CLN5, IMMP2L, CGB3, CTSE, CPXM2, CNTN2, IGHV4-59, CFHR1, C4BPA, DDI2, ADAMTS2, KLKB1, C1QC, IGHV3-30, GLI3, KLK1, ERO1B, FADD, IGLC1, XPNPEP3, CPE, BAD, PMPCA, F7, SERPINF2, ANXA2P2, F12, ADAM8, IGLV7-43, IGLV3-27, RIPK2, C1QB, IGLV3-19, SCG5, CR2, PLAU, C9orf3, XIAP, PCSK6, IGLV1-40, REN, C9, PLGRKT, SPCS3, CFH, TMEM59, IGHV3-53, GSN, MMP16, CPN2, PSEN2, PRSS3, SEC11B, IGHG3, FGA, CD46, IGHV3-11, SUSD4, ENO1, PGK1, IGLC7, CD55, PCSK1N, TIMM17A, CASP3, PTCH1, STOML2, CAPN2, PCSK5, NCSTN, CARD8, PCSK9, PARP1, IGHV3-13, FGG, FXN, IGLV2-14, CTSZ, CLN3, CUZD1, PMPCB, IGHV1OR15-1, IFI16, SERPINE2, IGLV1-47, CASP2, LHB, AEBP1, ANGPTL8, A2M, IGLV3-21, IGHV1-69, KLK6, TBC1D10A, ADAMTS13, MYH9, CPZ, FGB, SERPINE1, NLRP7, GRIN2A, AFG3L2, ECE2, CPA3, BIRC7, BACE2, BAK1, IGLV1-44, BCL2L12, C1QA, IGHG1, IGLV6-57, SRC, C5, IGHV4-34, F11, C7, IL1R2, PRSS12, IGHV2-5, CD19, CLU, TNP2, IGHV3-23, CCBE1 | 75 |
| 19 | GO:0008037 | cell recognition | TCP1, ROBO4, IGLL1, IGHV3-33, IGHG4, IGLC6, PEAR1, IGHV4-28, IGHV4-39, IGHV5-51, IGHV3-7, EPHB3, OVGP1, EPHB2, CCT2, IGHV1-45, IGHE, PRSS37, TRBC2, IGHV3-48, IGHG2, IGHD, DOCK8, IGHV3-43, CNTNAP3, ZAN, IGHV1-58, IGHV2-70, IGHM, VSTM2L, PCDH12, TNN, NDN, PCSK4, IGHV3-49, IGHV4-61, CDK5R1, CNTN2, IGHV4-59, CD36, IGSF9, CSGALNACT1, CCT3, FCN1, CATSPER3, IGHV3-30, IGLC1, ADAM21, ATP8B3, IGHV1-3, FETUB, BSG, IGHV4-4, COLEC12, CLEC7A, IGHV2-26, MSN, ROBO3, IGHV3-15, CNTN4, IGHV3-21, VDAC2, IGHV3-53, SPA17, B4GALT1, ACR, DOCK2, FCN2, IGHG3, MEGF10, IGLC2, IGHA2, SEMA3A, IGHV3-11, ZP4, DSCAM, ALDOA, IGLC7, CLGN, IGHV3-66, IGHV3-73, ADAM2, SEMA5A, CASP3, SPAM1, ZPBP, IGHV3-13, LGALS3, IGHV1-18, EPHA4, GAP43, IGLL5, IGHV1OR15-1, FCN3, IGHV3-72, CCT5, CNTNAP2, NEXN, PCDHA7, AMIGO1, IGHV1-69, PAEP, KCNU1, ADGRB1, CATSPER1, EFNB3, IGHV3-74, IGHV6-1, IGHV3-64, PCDHB6, NRP1, CCT4, MFGE8, IGLC3, IGHA1, NRCAM, IGHG1, DLG1, CD226, ST6GALNAC6, IGHV4-34, LAMA5, EMB, IGHV3-20, IGHV1-24, CNTN6, IGHV2-5, SPESP1, NPTN, IGHV3-23, IZUMO1, UBAP2L | 100 |

| Cluster | Term | Name | HPOs\_in\_clusters |
| --- | --- | --- | --- |
| 19 | OMIM:145410 | OPITZ GBBB SYNDROME, TYPE II; GBBB2 | HP:0001252, HP:0000347, HP:0000316, HP:0000431 |
| 19 | OMIM:605039 | BOHRING-OPITZ SYNDROME; BOPS | HP:0001252, HP:0000347, HP:0000316, HP:0000431 |
| 19 | OMIM:611209 | CONGENITAL DISORDER OF GLYCOSYLATION, TYPE IIg; CDG2G | HP:0001252, HP:0000347, HP:0000316, HP:0000431 |
| 19 | OMIM:114290 | CAMPOMELIC DYSPLASIA | HP:0000347, HP:0000316, HP:0001252 |
| 19 | OMIM:115150 | CARDIOFACIOCUTANEOUS SYNDROME 1; CFC1 | HP:0000347, HP:0000316, HP:0001252 |
| 19 | OMIM:151050 | LENZ-MAJEWSKI HYPEROSTOTIC DWARFISM; LMHD | HP:0000347, HP:0000316, HP:0001252 |
| 19 | OMIM:180700 | ROBINOW SYNDROME, AUTOSOMAL DOMINANT 1; DRS1 | HP:0000347, HP:0000316, HP:0000431 |
| 19 | OMIM:180849 | RUBINSTEIN-TAYBI SYNDROME 1; RSTS1 | HP:0001252, HP:0000347, HP:0000431 |
| 19 | OMIM:194190 | WOLF-HIRSCHHORN SYNDROME; WHS | HP:0000347, HP:0000316, HP:0000431 |
| 19 | OMIM:211750 | C SYNDROME | HP:0001252, HP:0000347, HP:0000431 |
| 19 | OMIM:213980 | CRANIOFACIAL DYSMORPHISM, SKELETAL ANOMALIES, AND MENTAL RETARDATION SYNDROME; CFSMR | HP:0000347, HP:0000316, HP:0001252 |
| 19 | OMIM:214100 | PEROXISOME BIOGENESIS DISORDER 1A (ZELLWEGER); PBD1A | HP:0000347, HP:0000316, HP:0001252 |
| 19 | OMIM:214110 | PEROXISOME BIOGENESIS DISORDER 2A (ZELLWEGER); PBD2A | HP:0000347, HP:0000316, HP:0001252 |
| 19 | OMIM:220111 | LEIGH SYNDROME, FRENCH CANADIAN TYPE; LSFC | HP:0001252, HP:0000316, HP:0000431 |
| 19 | OMIM:220210 | RITSCHER-SCHINZEL SYNDROME 1; RTSC1 | HP:0000347, HP:0000316, HP:0001252 |
| 19 | OMIM:222765 | RHIZOMELIC CHONDRODYSPLASIA PUNCTATA, TYPE 2; RCDP2 | HP:0001252, HP:0000347, HP:0000431 |
| 19 | OMIM:242840 | VICI SYNDROME; VICIS | HP:0000347, HP:0000316, HP:0001252 |
| 19 | OMIM:243310 | BARAITSER-WINTER SYNDROME 1; BRWS1 | HP:0001252, HP:0000316, HP:0000431 |
| 19 | OMIM:243605 | STROMME SYNDROME; STROMS | HP:0000347, HP:0000316, HP:0000431 |
| 19 | OMIM:248700 | MARDEN-WALKER SYNDROME; MWKS | HP:0000347, HP:0000316, HP:0001252 |
| 19 | OMIM:251300 | GALLOWAY-MOWAT SYNDROME 1; GAMOS1 | HP:0000347, HP:0000316, HP:0001252 |
| 19 | OMIM:253250 | MULIBREY NANISM | HP:0001252, HP:0000316, HP:0000431 |
| 19 | OMIM:268300 | ROBERTS SYNDROME; RBS | HP:0000347, HP:0000316, HP:0000431 |
| 19 | OMIM:268310 | ROBINOW SYNDROME, AUTOSOMAL RECESSIVE 1; RRS1 | HP:0000347, HP:0000316, HP:0000431 |
| 19 | OMIM:270400 | SMITH-LEMLI-OPITZ SYNDROME; SLOS | HP:0000347, HP:0000316, HP:0000431 |
| 19 | OMIM:277170 | OROFACIODIGITAL SYNDROME VI; OFD6 | HP:0000347, HP:0000316, HP:0001252 |
| 19 | OMIM:278250 | WRINKLY SKIN SYNDROME; WSS | HP:0001252, HP:0000316, HP:0000431 |
| 19 | OMIM:280000 | COLOBOMA, CONGENITAL HEART DISEASE, ICHTHYOSIFORM DERMATOSIS, MENTAL RETARDATION, AND EAR ANOMALIES SYNDROME; CHIME | HP:0001252, HP:0000316, HP:0000431 |
| 19 | OMIM:300215 | LISSENCEPHALY, X-LINKED, 2; LISX2 | HP:0001252, HP:0000347, HP:0000431 |
| 19 | OMIM:300373 | OSTEOPATHIA STRIATA WITH CRANIAL SCLEROSIS; OSCS | HP:0000347, HP:0000316, HP:0000431 |
| 19 | OMIM:300749 | MENTAL RETARDATION AND MICROCEPHALY WITH PONTINE AND CEREBELLAR HYPOPLASIA; MICPCH | HP:0000347, HP:0000316, HP:0000431 |
| 19 | OMIM:300978 | TONNE-KALSCHEUER SYNDROME; TOKAS | HP:0000347, HP:0000316, HP:0000431 |
| 19 | OMIM:304110 | CRANIOFRONTONASAL SYNDROME; CFNS | HP:0001252, HP:0000316, HP:0000431 |
| 19 | OMIM:309580 | MENTAL RETARDATION-HYPOTONIC FACIES SYNDROME, X-LINKED, 1; MRXHF1 | HP:0000347, HP:0000316, HP:0000431 |
| 19 | OMIM:311900 | TARP SYNDROME; TARPS | HP:0000347, HP:0000316, HP:0000431 |
| 19 | OMIM:312870 | SIMPSON-GOLABI-BEHMEL SYNDROME, TYPE 1; SGBS1 | HP:0001252, HP:0000316, HP:0000431 |
| 19 | OMIM:601186 | MICROPHTHALMIA, SYNDROMIC 9; MCOPS9 | HP:0001252, HP:0000347, HP:0000431 |
| 19 | OMIM:601390 | VAN MALDERGEM SYNDROME 1; VMLDS1 | HP:0000347, HP:0000316, HP:0000431 |
| 19 | OMIM:608540 | CONGENITAL DISORDER OF GLYCOSYLATION, TYPE Ik; CDG1K | HP:0000347, HP:0000316, HP:0001252 |
| 19 | OMIM:608799 | CONGENITAL DISORDER OF GLYCOSYLATION, TYPE Ie; CDG1E | HP:0000347, HP:0000316, HP:0001252 |
| 19 | OMIM:609528 | CEREBRAL DYSGENESIS, NEUROPATHY, ICHTHYOSIS, AND PALMOPLANTAR KERATODERMA SYNDROME | HP:0001252, HP:0000316, HP:0000431 |
| 19 | OMIM:609924 | AMINOACYLASE 1 DEFICIENCY; ACY1D | HP:0001252, HP:0000316, HP:0000431 |
| 19 | OMIM:613177 | CUTIS LAXA, AUTOSOMAL RECESSIVE, TYPE IC; ARCL1C | HP:0000347, HP:0000316, HP:0000431 |
| 19 | OMIM:613406 | WITTEVEEN-KOLK SYNDROME; WITKOS | HP:0001252, HP:0000316, HP:0000431 |
| 19 | OMIM:613610 | CRANIOECTODERMAL DYSPLASIA 2; CED2 | HP:0000347, HP:0000316, HP:0000431 |
| 19 | OMIM:615546 | VAN MALDERGEM SYNDROME 2; VMLDS2 | HP:0000347, HP:0000316, HP:0000431 |
| 19 | OMIM:616897 | OSTEOCHONDRODYSPLASIA, COMPLEX LETHAL, SYMOENS-BARNES-GISTELINCK TYPE; OCLSBG | HP:0000347, HP:0000316, HP:0000431 |
| 19 | OMIM:617062 | OKUR-CHUNG NEURODEVELOPMENTAL SYNDROME; OCNDS | HP:0000347, HP:0000316, HP:0000431 |
| 19 | OMIM:617746 | SWEENEY-COX SYNDROME; SWCOS | HP:0000347, HP:0000316, HP:0000431 |

---


---


---

# Cluster 31

| Cluster | Term | Name |
| --- | --- | --- |
| 31 | HP:0003508 | Proportionate short stature |
| 31 | HP:0008551 | Microtia |
| 31 | HP:0000508 | Ptosis |

| Cluster | Term | Name | Genes | Percentage\_of\_nodes\_with\_funsys |
| --- | --- | --- | --- | --- |
| 31 | GO:0048706 | embryonic skeletal system development | HOXC5, SATB2, HOXA2, SIX2, MMP16, SCX, BMP7, HOXC9, HOXA11, GLI3, DLX1, SULF1, DMRT2, TBXT, HOXC11, HOXA5, HOXA3, SP3, RDH10, LHX1, SLC35D1, DLG1, HOXC6, TBX1, BMP4, GNAS, HOXA6, TBX15, PRRX1, DLX2, HOXA4, NIPBL, ACVR2A, EYA1, OSR2, HOXA9, HOXA7, HOXC4, PAX7, HOXA1, ZEB1, MTHFD1L | 100 |
| 31 | GO:0003002 | regionalization | HOXC5, HOXA2, CHRD, ETS2, CYP26B1, NEUROG1, RELN, HES7, TDRD5, HOXC11, PBX3, PRKDC, DVL2, HNF1B, TTC21B, HSPB11, DCANP1, LRP2, SKI, PGAP1, MSX1, NKX2-5, HOXA4, HOXA9, TBX20, EGR2, HES5, TIFAB, TRAF3IP1, RARG, SIX2, TBC1D32, TBX3, HOXC9, FKBP8, HOXC8, FOXF1, HOXA11, GLI3, DMRT2, GDF11, HOXA5, SOX1, HOXA3, PROP1, RIPPLY2, KDM6A, DMRT3, GATA4, HOXA6, RNF2, DLX2, BMI1, GREM2, YY1, PCSK6, HOXA7, HOXC4, CELSR1, MAFB, INTU, AIDA, HOXC10, TSHZ1, TMEM107, TP63, HOXC13, FEZF1, NKX6-2, NRARP, CTNNBIP1, HES4, WNT7A, SEMA3A, SPRY1, HES1, HOXA10, DLX1, NOTO, PTCH1, LHX1, NRP2, HOXC6, TBX1, CITED2, MNS1, SIX3, EMX1, LFNG, PLD6, SNAI1, MEOX1, SETDB2, VAX2, CHSY1, TBX6, ZEB2, TRA2B, NKX3-1, GORAB, TGFBR1, TBR1, POFUT1, IRX2, NOG, CRKL, WNT7B, CDX2, PKD1L1, RIPPLY1, PCDH8, FZD5, SOX17, CRB2, HES6, DISP1, MKS1, NBL1, LHX2, SENP2, ACVR2A, GBX2, PAX7, BPTF, EVX1 | 100 |
| 31 | GO:0048705 | skeletal system morphogenesis | SIX2, HOXA2, SATB2, DHRS3, COMP, MMP16, SCX, RARG, NAB1, TBX4, BMP7, COL6A3, HOXC9, RFLNA, WNT7A, CSGALNACT1, HOXC8, WWOX, HOXA11, GLI3, NOG, ZNF664-RFLNA, TBXT, HOXC11, HOXA5, IMPAD1, PAPPA2, HOXA3, DLX5, NPPC, IFT80, RDH10, LHX1, RIPPLY2, FREM1, SFRP4, DLG1, TBX1, GNAS, POR, HOXA6, TBX15, PRRX1, SKI, DLX2, MSX1, HOXA4, VWA1, OSR2, VIT, HOXA7, HOXC4, FGFR3, RAB33B, STC1, MATN2, HOXA1, SP5, CHSY1, MTHFD1L | 100 |
| 31 | GO:0048704 | embryonic skeletal system morphogenesis | SATB2, HOXA2, SIX2, MMP16, BMP7, HOXC9, HOXA11, GLI3, HOXC11, HOXA5, HOXA3, RDH10, LHX1, DLG1, TBX1, GNAS, HOXA6, TBX15, PRRX1, DLX2, HOXA4, NIPBL, OSR2, HOXA7, HOXC4, HOXA1, ZEB1, MTHFD1L | 100 |
| 31 | GO:0009952 | anteriorposterior pattern specification | HOXC5, HOXA2, SHH, ETS2, HIPK2, NEUROG1, HES7, TDRD5, TBXT, HOXC11, PBX3, PRKDC, PLXNA2, HNF1B, DCANP1, SKI, PGAP1, MSX1, HOXA4, HOXA9, CFC1, HES5, TIFAB, RARG, GRSF1, SIX2, TBX3, HOXC9, HOXC8, FOXF1, HOXA11, GLI3, WNT2, DMRT2, GDF11, HOXA5, HOXA3, RIPPLY2, KDM6A, BTG2, EP300, GATA4, HOXA6, RNF2, YY1, PCSK6, HOXA7, HOXC4, CELSR1, OTX1, HOXC10, TSHZ1, HOXC13, FEZF1, SFRP2, VANGL2, NRARP, CELSR2, CTNNBIP1, HES4, HES1, HOXA10, NOTCH1, DLL1, LHX1, PCSK5, SMO, HOXC6, TBX1, MSX2, SIX3, LFNG, PLD6, MEOX1, TBX6, ZEB2, NKX3-1, WNT3A, TGFBR1, POFUT1, CFC1B, NOG, CRKL, CDX2, RIPPLY1, FZD5, SOX17, CRB2, HES6, LRP5L, ACVR2A, GBX2, BPTF, CER1 | 100 |

| Cluster | Term | Name | HPOs\_in\_clusters |
| --- | --- | --- | --- |
| 31 | OMIM:101400 | SAETHRE-CHOTZEN SYNDROME; SCS | HP:0008551, HP:0000508 |
| 31 | OMIM:113620 | BRANCHIOOCULOFACIAL SYNDROME; BOFS | HP:0008551, HP:0000508 |
| 31 | OMIM:214800 | CHARGE SYNDROME | HP:0008551, HP:0000508 |
| 31 | OMIM:309580 | MENTAL RETARDATION-HYPOTONIC FACIES SYNDROME, X-LINKED, 1; MRXHF1 | HP:0008551, HP:0000508 |
| 31 | OMIM:601390 | VAN MALDERGEM SYNDROME 1; VMLDS1 | HP:0008551, HP:0000508 |
| 31 | OMIM:615546 | VAN MALDERGEM SYNDROME 2; VMLDS2 | HP:0008551, HP:0000508 |
| 31 | OMIM:616580 | AU-KLINE SYNDROME; AUKS | HP:0008551, HP:0000508 |
| 31 | OMIM:616723 | SPONDYLOEPIMETAPHYSEAL DYSPLASIA, FADEN-ALKURAYA TYPE; SEMDFA | HP:0008551, HP:0000508 |
| 31 | OMIM:617564 | MEIER-GORLIN SYNDROME 8; MGORS8 | HP:0008551, HP:0000508 |

---


---


---

# Cluster 54

| Cluster | Term | Name |
| --- | --- | --- |
| 54 | HP:0000286 | Epicanthus |
| 54 | HP:0000431 | Wide nasal bridge |
| 54 | HP:0000494 | Downslanted palpebral fissures |
| 54 | HP:0000316 | Hypertelorism |
| 54 | HP:0000347 | Micrognathia |
| 54 | HP:0004209 | Clinodactyly of the 5th finger |

| Cluster | Term | Name | Genes | Percentage\_of\_nodes\_with\_funsys |
| --- | --- | --- | --- | --- |
| 54 | GO:0002526 | acute inflammatory response | SERPINC1, IGHV3-33, PARK7, IGHG4, IGLC6, IGLV2-23, CR1, TNFSF11, IGHV4-39, C8G, IGHV3-7, IGLV3-25, CFHR4, MIR92A2, TNFSF4, GSTP1, CFHR5, IGHV3-48, IGLV2-8, IGHG2, IGLV2-11, PIK3CG, EIF2AK1, APCS, C1QBP, IGLV3-1, CFHR2, OSMR, IGHV2-70, FCGR2B, C6, TAC1, C4BPB, VCAM1, PPARG, IGLV1-51, OPRM1, CPB2, CRP, FCER1G, IGHV4-59, CFHR1, NUPR1, C4BPA, KLKB1, TNFRSF11A, IGHV3-30, C1QC, IL6, IL6ST, IGLC1, LBP, SERPINF2, F12, ADAM8, IGLV7-43, IGLV3-27, RHBDD3, C1QB, ADORA1, IGLV3-19, NLRP3, NPFF, CR2, IGLV1-40, C9, CFH, IL20RB, IGHV3-53, SELENOS, PTGES, F8, B4GALT1, PLSCR1, CPN2, MIR92A1, CTNNBIP1, IGHG3, TFR2, CD46, IGHV3-11, SUSD4, UGT1A1, IGLC7, CD55, OSM, CREB3L3, DNASE1, BTK, ADCYAP1, FN1, IGHV3-13, GATA3, IGLV2-14, HP, IGHV1OR15-1, AHSG, IGLV1-47, TRPV1, DNASE1L3, A2M, IGLV3-21, ASS1, IGHV1-69, EPO, EDNRB, ASH1L, PTGS2, PTGER3, IGLV1-44, C1QA, IGHG1, IGLV6-57, ELANE, C5, IL31RA, APOA2, IGHV4-34, HPR, C7, OGG1, CEBPB, APOL2, IGHV2-5, CD19, CLU, IGHV3-23 | 83.33333 |
| 54 | GO:0016485 | protein processing | SEC11C, CTSH, LGMN, SHH, IGHV3-33, IGHG4, IGLC6, IGLV2-23, KLK13, CR1, IGHV4-39, C8G, F9, IGHV3-7, MELTF, ASPH, IGLV3-25, CFHR4, IMMP1L, ASPRV1, PRSS37, CFHR5, IGHV3-48, IGLV2-8, IGHG2, KLK2, IGLV2-11, YME1L1, USP17L2, MME, HM13, IGLV3-1, KLK3, CFHR2, IGHV2-70, C6, C4BPB, PISD, PCSK4, CPXM1, ACP4, IGLV1-51, CPB2, PYCARD, CLN5, IMMP2L, CGB3, CTSE, CPXM2, CNTN2, IGHV4-59, CFHR1, C4BPA, DDI2, ADAMTS2, KLKB1, C1QC, IGHV3-30, GLI3, KLK1, ERO1B, FADD, IGLC1, XPNPEP3, CPE, BAD, PMPCA, F7, SERPINF2, ANXA2P2, F12, ADAM8, IGLV7-43, IGLV3-27, RIPK2, C1QB, IGLV3-19, SCG5, CR2, PLAU, C9orf3, XIAP, PCSK6, IGLV1-40, REN, C9, PLGRKT, SPCS3, CFH, TMEM59, IGHV3-53, GSN, MMP16, CPN2, PSEN2, PRSS3, SEC11B, IGHG3, FGA, CD46, IGHV3-11, SUSD4, ENO1, PGK1, IGLC7, CD55, PCSK1N, TIMM17A, CASP3, PTCH1, STOML2, CAPN2, PCSK5, NCSTN, CARD8, PCSK9, PARP1, IGHV3-13, FGG, FXN, IGLV2-14, CTSZ, CLN3, CUZD1, PMPCB, IGHV1OR15-1, IFI16, SERPINE2, IGLV1-47, CASP2, LHB, AEBP1, ANGPTL8, A2M, IGLV3-21, IGHV1-69, KLK6, TBC1D10A, ADAMTS13, MYH9, CPZ, FGB, SERPINE1, NLRP7, GRIN2A, AFG3L2, ECE2, CPA3, BIRC7, BACE2, BAK1, IGLV1-44, BCL2L12, C1QA, IGHG1, IGLV6-57, SRC, C5, IGHV4-34, F11, C7, IL1R2, PRSS12, IGHV2-5, CD19, CLU, TNP2, IGHV3-23, CCBE1 | 83.33333 |

| Cluster | Term | Name | HPOs\_in\_clusters |
| --- | --- | --- | --- |
| 54 | OMIM:115150 | CARDIOFACIOCUTANEOUS SYNDROME 1; CFC1 | HP:0000286, HP:0000347, HP:0000494, HP:0000316, HP:0004209 |
| 54 | OMIM:145410 | OPITZ GBBB SYNDROME, TYPE II; GBBB2 | HP:0000286, HP:0000347, HP:0000431, HP:0000494, HP:0000316 |
| 54 | OMIM:180849 | RUBINSTEIN-TAYBI SYNDROME 1; RSTS1 | HP:0000286, HP:0000347, HP:0000431, HP:0000494, HP:0004209 |
| 54 | OMIM:300373 | OSTEOPATHIA STRIATA WITH CRANIAL SCLEROSIS; OSCS | HP:0000286, HP:0000347, HP:0000431, HP:0000316, HP:0004209 |
| 54 | OMIM:605130 | WIEDEMANN-STEINER SYNDROME; WDSTS | HP:0000286, HP:0000431, HP:0000494, HP:0000316, HP:0004209 |

---


---


---

# Cluster 32

| Cluster | Term | Name |
| --- | --- | --- |
| 32 | HP:0000316 | Hypertelorism |
| 32 | HP:0000347 | Micrognathia |
| 32 | HP:0000356 | Abnormality of the outer ear |
| 32 | HP:0000431 | Wide nasal bridge |
| 32 | HP:0000581 | Blepharophimosis |

| Cluster | Term | Name | Genes | Percentage\_of\_nodes\_with\_funsys |
| --- | --- | --- | --- | --- |
| 32 | GO:0072376 | protein activation cascade | SERPINC1, IGLL1, IGHV3-33, IGHG4, IGLC6, IGLV2-23, TFPI, CR1, IGHV4-28, IGHV4-39, C8G, IGHV5-51, IGHV3-7, KRT1, F9, IGLV3-25, CFHR4, IGHV1-45, IGHE, TRBC2, CFHR5, IGHV3-48, IGLV2-8, IGHG2, IGHD, IGLV2-11, IGHV3-43, IGHV1-58, APCS, C1QBP, IGLV3-1, CFHR2, IGHV2-70, IGHM, GP5, C6, C4BPB, IGLV1-51, CPB2, IGHV3-49, SCARA3, CRP, IGHV4-61, IGHV4-59, CFHR1, C4BPA, FCN1, KLKB1, C1QC, IGHV3-30, IGLC1, IGHV1-3, F7, IGHV4-4, F12, IGLV7-43, IGLV3-27, C1QB, IGHV2-26, IGLV3-19, CR2, IGHV3-15, IGLV1-40, C9, CFH, IGHV3-21, FBLN1, RGCC, IGHV3-53, CFD, F8, CPN2, FCN2, IGHG3, FGA, IGLC2, CD46, IGHA2, MASP2, F13B, IGHV3-11, SUSD4, IGLC7, CD55, IGHV3-66, IGHV3-73, IGHV3-13, F10, FGG, IGHV1-18, COL20A1, MASP1, IGLV2-14, KNG1, IGLL5, IGHV1OR15-1, IGLV1-47, FCN3, IGHV3-72, A2M, IGLV3-21, IGHV1-69, IGHV3-74, IGHV6-1, GP1BB, IGHV3-64, FGB, COLEC10, GP1BA, VSIG4, IGLV1-44, IGLC3, IGHA1, C1QA, IGHG1, IGLV6-57, C5, IGHV4-34, F11, C7, IGHV3-20, IGHV1-24, IGHV2-5, CD19, CLU, IGHV3-23 | 80 |
| 32 | GO:0002455 | humoral immune response mediated by circulating immunoglobulin | IGLL1, IGHV3-33, IGHG4, IGLC6, IGLV2-23, CR1, IGHV4-28, IGHV4-39, C8G, IGHV5-51, IGHV3-7, IGLV3-25, EXO1, IGHV1-45, IGHE, TRBC2, IGHV3-48, IGLV2-8, IGHG2, IGHD, IGLV2-11, IGHV3-43, IGHV1-58, APCS, C1QBP, IGLV3-1, IGHV2-70, IGHM, FCGR2B, C6, C4BPB, IGLV1-51, IGHV3-49, CRP, IGHV4-61, IGHV4-59, C4BPA, C1QC, IGHV3-30, IGLC1, IGHV1-3, IGHV4-4, IGLV7-43, IGLV3-27, C1QB, IGHV2-26, IGLV3-19, CR2, IGHV3-15, IGLV1-40, C9, IGHV3-21, IGHV3-53, IGHG3, CD46, IGLC2, IGHA2, MASP2, IGHV3-11, SUSD4, IGLC7, CD55, IGHV3-66, IGHV3-73, IGHV3-13, IGHV1-18, PTPRC, IGLV2-14, IGLL5, IGHV1OR15-1, IGLV1-47, IGHV3-72, IGLV3-21, IGHV1-69, IGHV3-74, IGHV6-1, IGHV3-64, IGLV1-44, IGLC3, IGHA1, C1QA, IGHG1, IGLV6-57, C5, IGHV4-34, C7, IGHV3-20, IGHV1-24, IGHV2-5, CLU, IGHV3-23 | 80 |
| 32 | GO:0006910 | phagocytosis, recognition | IGHV3-53, IGLL1, IGHV3-33, IGHV1-69, IGHV4-61, IGHG4, IGLC6, IGHV4-59, FCN2, PEAR1, IGHV4-28, IGHV4-39, ADGRB1, IGHG3, IGHV5-51, IGHV3-7, MEGF10, CD36, IGLC2, FCN1, IGHA2, IGHV3-74, IGHV6-1, IGHV3-11, IGHV3-30, IGHE, IGHV1-45, IGHV3-64, TRBC2, IGLC7, IGLC1, IGHV3-48, IGHV3-66, IGHV3-73, MFGE8, IGHG2, IGHD, IGLC3, IGHA1, IGHV1-3, IGHV3-43, IGHG1, IGHV4-4, COLEC12, CLEC7A, IGHV1-58, IGHV3-13, IGHV4-34, IGHV1-18, IGHV2-26, IGHV2-70, IGHM, IGHV3-20, IGHV1-24, IGLL5, IGHV2-5, IGHV1OR15-1, IGHV3-15, FCN3, IGHV3-21, IGHV3-72, IGHV3-23, IGHV3-49 | 80 |
| 32 | GO:0030449 | regulation of complement activation | IGHV3-53, IGLV3-21, IGHV3-33, IGHV1-69, IGHG4, IGLC6, IGLV2-23, CPN2, CR1, IGHV4-59, IGHV4-39, IGHG3, CFHR1, C8G, IGHV3-7, CD46, C4BPA, CFHR4, IGLV3-25, SUSD4, IGHV3-11, C1QC, IGHV3-30, CFHR5, IGLC7, CD55, IGHV3-48, IGLC1, IGLV2-8, IGLV1-44, IGHG2, IGLV2-11, C1QA, IGHG1, IGLV6-57, IGLV7-43, C5, IGHV3-13, IGLV3-27, IGHV4-34, IGLV3-1, C1QB, CFHR2, IGLV3-19, IGHV2-70, C7, C1QBP, IGLV2-14, CR2, C6, C4BPB, IGHV2-5, IGHV1OR15-1, IGLV1-47, IGLV1-40, CD19, C9, CFH, CLU, IGLV1-51, IGHV3-23, CPB2, A2M | 80 |
| 32 | GO:0019724 | B cell mediated immunity | IGLL1, IGHV3-33, CD40LG, IGHG4, IGLC6, IGLV2-23, FOXP3, CR1, IGHV4-28, IGHV4-39, C8G, IGHV5-51, IGHV3-7, IGLV3-25, EXO1, MLH1, IGHV1-45, IGHE, TNFSF4, TRBC2, IGHV3-48, IGLV2-8, IGHG2, IGHD, IGLV2-11, IGHV3-43, BCL6, IL13RA2, IGHV1-58, APCS, C1QBP, IGLV3-1, IGHV2-70, IGHM, TNFSF13, FCGR2B, C6, C4BPB, XCL1, MAD2L2, IGLV1-51, IGHV3-49, CRP, SHLD2, FCER1G, IGHV4-61, THOC1, IL4, INPP5D, IGHV4-59, C4BPA, C1QC, IGHV3-30, RIF1, IGLC1, APLF, IGHV3-23, IGHV1-3, LIG4, IGHV4-4, IGLV7-43, IGLV3-27, C1QB, IGHV2-26, IGLV3-19, PAXIP1, CR2, HSPD1, IGHV3-15, IGLV1-40, CD28, C9, IGHV3-21, IGHV3-53, IGHG3, IGLC2, CD46, IGHA2, IL2, MASP2, IGHV3-11, SUSD4, IL10, IGLC7, CD55, IGHV3-66, IGHV3-73, BTK, IGHV3-13, GAPT, IGHV1-18, PTPRC, IGLV2-14, IGLL5, IGHV1OR15-1, IGLV1-47, IGHV3-72, ATAD5, NSD2, IGLV3-21, MSH6, IGHV1-69, CCR6, NBN, BCL10, RNF168, IGHV3-74, IGHV6-1, IGHV3-64, MSH2, NDFIP1, IL4R, IGLV1-44, IGLC3, IGHA1, C1QA, IGHG1, IGLV6-57, CD226, C5, IGHV4-34, C7, CLCF1, IGHV3-20, IGHV1-24, IGHV2-5, CLU, SLA2, TFRC | 80 |
| 32 | GO:0050867 | positive regulation of cell activation | CD83, TESPA1, IGLL1, SHH, IGHV3-33, EGR3, CD40LG, SIRPB1, GAS6, IGHG4, IGLC6, FOXP3, CSK, TNFSF11, IGHV4-28, IGHV4-39, TMIGD2, IGHV5-51, IGHV3-7, PAK2, EFNB2, AP3D1, MLH1, MIR92A2, IGHV1-45, IGHE, CD1D, TRBC2, TNFSF4, PCID2, IGHV3-48, CTLA4, IGHG2, IGHD, CAV1, DOCK8, IGHV3-43, TNFRSF14, CLECL1, DNAJA3, BCL6, IGHV1-58, CARD11, ANXA1, HMGB1, IGHV2-70, IGHM, TNFSF13, GRB2, IL7, TAC1, XCL1, VCAM1, PTAFR, ICOS, MAD2L2, SLC39A10, IGHV3-49, SHLD2, PYCARD, CD274, ITPKB, FCER1G, CD276, CD47, PAK3, IGHV4-61, DPP4, IL4, INPP5D, IGHV4-59, KLRK1, MIR30B, LEP, MIR21, SART1, IL6, IGHV3-30, GLI3, GPR183, RIF1, PPP2R3C, IL6ST, FADD, IGLC1, IGHV3-23, BAD, ITGB2, CRLF2, PDPK1, IGHV1-3, FLT3LG, TNFRSF13C, LBP, FGF10, IGHV4-4, ADAM8, RIPK2, IGHV2-26, NLRP3, IRS2, PAXIP1, IL1RL1, HSPD1, CBFB, SIRPA, HHLA2, RAC1, STXBP1, IGHV3-15, CD28, IGHV3-21, LAMP1, JAK2, YES1, CORO1A, MAP3K8, IGHV3-53, PDCD1LG2, IL5, FGR, IL23R, MIR92A1, ADORA2B, LILRB1, MIF, GPAM, IGHG3, IGLC2, CD46, NFATC2, IGHA2, IL2, IL10, IGHV3-11, EBI3, SOCS5, ZP4, HES1, NKAP, IGLC7, CD55, STAP1, MALT1, IGHV3-66, IGHV3-73, TNIP2, LGALS1, PRKCZ, SELP, BCL2, BTK, IL21, MEF2C, PDPN, ATP11C, AP1G1, IL2RA, IGHV3-13, IGHV1-18, PTPRC, GATA3, HSPH1, SPTA1, IL1RL2, IGLL5, FCRL3, IGHV1OR15-1, CD86, IGHV3-72, SOX15, HLX, ATAD5, NSD2, MSH6, NPPA, SASH3, IGHV1-69, EPO, CD38, BCL10, TNFRSF4, PLEK, NCKAP1L, WNT3A, IL12B, IL12A, GRAP2, DMTN, EFNB3, IGHV3-74, IGHV6-1, SOCS1, BST1, IGHV3-64, MSH2, NCK1, RASAL3, ITGAM, IL15, IL4R, KLRC4-KLRK1, AKT1, CD80, IGLC3, IGHA1, CCDC88B, PDCD1, XBP1, LILRB4, IGHG1, SRC, DUSP10, CD226, IGHV4-34, IL33, CLCF1, STX4, TNFSF13B, IGHV3-20, IGHV1-24, LILRB2, F2RL1, IGHV2-5, SIRPG, PRKCQ, NR4A3, FYN, BTLA, PELI1, TICAM1, IL13, TFRC, TIRAP | 80 |
| 32 | GO:0038096 | Fc-gamma receptor signaling pathway involved in phagocytosis | IGHV3-33, IGHG4, IGLC6, IGLV2-23, DOCK1, IGHV4-39, VAV2, IGHV3-7, PLCG2, IGLV3-25, WASL, IGHV3-48, IGLV2-8, IGHG2, IGLV2-11, ACTB, CD247, PLA2G6, PRKCE, IGLV3-1, IGHV2-70, FCGR2B, MAPK1, CRK, WASF2, GRB2, ARPC5, IGLV1-51, ARPC4, CD47, RAPGEF1, RAP1A, IGHV4-59, CYFIP1, IGHV3-30, HSP90AA1, MYO1C, ARPC1B, IGLC1, IGLV7-43, IGLV3-27, IGLV3-19, MYO1G, RAC1, IGLV1-40, FCGR1A, YES1, IGHV3-53, WAS, FGR, HCK, ACTR2, IGHG3, FCGR2A, PTPRJ, ABL1, IGHV3-11, IGLC7, ELMO1, PTK2, IGHV3-13, PTPRC, WIPF1, IGLV2-14, IGHV1OR15-1, IGLV1-47, IGLV3-21, BRK1, ABI1, IGHV1-69, NCKAP1L, PIK3CB, MAPK3, MYO10, NCK1, ARPC1A, IGLV1-44, IGHG1, IGLV6-57, SRC, IGHV4-34, WIPF3, FCGR3A, IGHV2-5, NCKAP1, FYN, LIMK1, IGHV3-23 | 80 |
| 32 | GO:0006958 | complement activation, classical pathway | IGLL1, IGHV3-33, IGHG4, IGLC6, IGLV2-23, CR1, IGHV4-28, IGHV4-39, C8G, IGHV5-51, IGHV3-7, IGLV3-25, IGHV1-45, IGHE, TRBC2, IGHV3-48, IGLV2-8, IGHG2, IGHD, IGLV2-11, IGHV3-43, IGHV1-58, APCS, C1QBP, IGLV3-1, IGHV2-70, IGHM, C6, C4BPB, IGLV1-51, IGHV3-49, CRP, IGHV4-61, IGHV4-59, C4BPA, C1QC, IGHV3-30, IGLC1, IGHV1-3, IGHV4-4, IGLV7-43, IGLV3-27, C1QB, IGHV2-26, IGLV3-19, CR2, IGHV3-15, IGLV1-40, C9, IGHV3-21, IGHV3-53, IGHG3, IGLC2, CD46, IGHA2, MASP2, IGHV3-11, SUSD4, IGLC7, CD55, IGHV3-66, IGHV3-73, IGHV3-13, IGHV1-18, IGLV2-14, IGLL5, IGHV1OR15-1, IGLV1-47, IGHV3-72, IGLV3-21, IGHV1-69, IGHV3-74, IGHV6-1, IGHV3-64, IGLV1-44, IGLC3, IGHA1, C1QA, IGHG1, IGLV6-57, C5, IGHV4-34, C7, IGHV3-20, IGHV1-24, IGHV2-5, CLU, IGHV3-23 | 80 |
| 32 | GO:0050851 | antigen receptor-mediated signaling pathway | TESPA1, IGLL1, IGHV3-33, RAB29, IGHG4, IGLC6, PVRIG, PAG1, FOXP3, CSK, KLHL6, DENND1B, IGHV4-28, IGHV4-39, BLK, DUSP22, IGHV5-51, IGHV3-7, PLCG2, PAK2, IKBKG, IGHV1-45, IGHE, TRBC2, IGHV3-48, CTLA4, HRAS, PTPN2, LCP2, IGHG2, IGHD, NFAM1, RBCK1, IGHV3-43, RELA, CD247, IGHV1-58, PAWR, CARD11, IGHV2-70, IGHM, GCSAM, MAPK1, ELF2, PHPT1, SLC39A10, LAT2, PDE4D, IGHV3-49, CD276, PAK3, IGHV4-61, INPP5D, TXK, IGHV4-59, CMTM3, MNDA, LIME1, TRAT1, BTNL9, PIK3CD, IKBKB, IGHV3-30, IGLC1, PAX5, IGHV3-23, PDPK1, IGHV1-3, IGHV4-4, RIPK2, ERMAP, IGHV2-26, SH2B2, DUSP3, CBFB, HHLA2, IGHV3-15, CD28, PLEKHA1, IGHV3-21, EZR, IGHV3-53, WAS, CACNA1F, BTNL3, LAT, GCSAML, RC3H2, IGHG3, IGLC2, PTPRJ, NFATC2, IGHA2, ABL1, IGHV3-11, FYB1, PLCL2, RC3H1, IGLC7, STAP1, MALT1, IGHV3-66, IGHV3-73, BCL2, STOML2, BTK, MEF2C, PLCG1, IGHV3-13, LGALS3, PDE4B, IGHV1-18, PTPRC, BTNL8, GATA3, IGLL5, FCRL3, IGHV1OR15-1, IGHV3-72, GPS2, IGHV1-69, BTNL10, CD38, BCL10, NCKAP1L, PIK3CB, GRAP2, IGHV3-74, IGHV6-1, IGHV3-64, NCK1, THEMIS2, IGLC3, IGHA1, LILRB4, CD300A, IGHG1, CD226, IGHV4-34, STK11, BAX, TEC, IGHV3-20, IGHV1-24, IGHV2-5, ELF1, CD19, PRKCQ, FYN, RFTN1, BRAF, SLA2, EIF2B5 | 80 |
| 32 | GO:0042113 | B cell activation | POLM, IGLL1, IGHV3-33, CD40LG, IGHG4, IGLC6, FOXP3, HDAC4, IGHV4-28, IGHV4-39, IFNA5, IGHV5-51, IGHV3-7, PLCG2, CHRNA4, EXO1, MLH1, IGHV1-45, IGHE, IFNB1, HHEX, TRBC2, TNFSF4, PCID2, IGHV3-48, CTLA4, PTPN2, IGHG2, IGHD, NFAM1, IGHV3-43, INHBA, BCL6, IGHV1-58, PAWR, CARD11, IFNA14, IRF2BP2, IGHV2-70, IGHM, TNFSF13, FCGR2B, AHR, TNFRSF13B, IL7, VCAM1, MAD2L2, LAT2, SLC39A10, IGHV3-49, SHLD2, IFNA6, IFNA4, IFNA10, IGHV4-61, THOC1, HDAC9, IFNA13, IL4, AKAP17A, INPP5D, IGHV4-59, MNDA, PIK3CD, IFNE, IL6, IGHV3-30, GPR183, RIF1, PPP2R3C, IGLC1, APLF, FZD9, IGHV3-23, BAD, IFNA16, IGHV1-3, GON4L, LIG4, TNFRSF13C, IGHV4-4, FCRL1, IFNA2, EP300, IGHV2-26, IRS2, PAXIP1, CR2, HSPD1, IGHV3-15, ITM2A, CD28, IFNA1, IGHV3-21, ONECUT1, LYL1, IGHV3-53, IL5, FNIP1, CDH17, MIF, IGHG3, CASP8, IGLC2, PTPRJ, NFATC2, IGHA2, IL2, TPD52, IGHV3-11, IL10, ABL1, PLCL2, RC3H1, IGLC7, MALT1, IGHV3-66, IGHV3-73, TNIP2, LGALS1, BCL2, LRRC8A, ZFP36L2, CASP3, DLL1, BTK, ITGA4, IL21, IFNA17, MEF2C, ATP11C, IGHV3-13, GAPT, IGHV1-18, PTPRC, IFNA7, LFNG, IGLL5, FCRL3, IGHV1OR15-1, IGHV3-72, IFNA21, ATAD5, NSD2, GPS2, MSH6, SASH3, PTK2B, IGHV1-69, SLA2, CCR6, NTRK1, CD38, NBN, TNFRSF4, NCKAP1L, WNT3A, SKAP2, RNF168, IGHV3-74, IGHV6-1, BST1, IGHV3-64, MSH2, MFNG, IFNK, NDFIP1, HDAC5, DNAJB9, TCF3, BAK1, KLF6, IFNA8, IGLC3, IGHA1, MIR17HG, SP3, XBP1, ZBTB7A, CD300A, IGHG1, IGHV4-34, TBC1D10C, PKN1, CLCF1, TNFSF13B, BAX, IGHV3-20, IGHV1-24, SFRP1, IGHV2-5, DOCK11, LAX1, PELI1, TICAM1, DCLRE1C, ITGB1, IFNW1, IL13, ZFP36L1, TFRC, TIRAP | 80 |
| 32 | GO:0006909 | phagocytosis | SYT11, IGLL1, IGHV3-33, SIRPB1, GAS6, IGHG4, IGLC6, IGLV2-23, CSK, MSR1, SH3BP1, DOCK1, IGHV4-28, IGHV4-39, IRF8, VAV2, IGHV5-51, IGHV3-7, PLCG2, PEAR1, ELMO3, IGLV3-25, SPON2, MESD, RUBCN, IGHV1-45, IGHE, TRBC2, PRTN3, WASL, IGHV3-48, IGLV2-8, IGHG2, IGHD, IGLV2-11, ACTB, ATG5, AZU1, IGHV3-43, EIF2AK1, CD247, IGHV1-58, PLA2G6, ANXA1, PRKCG, IGLV3-1, HMGB1, TLR2, IGHV2-70, IGHM, FCGR2B, MAPK1, CRK, WASF2, LMAN2, GRB2, ARPC5, C4BPB, ATG3, PPARG, PTX3, IGLV1-51, IGHV3-49, ARPC4, CRP, PYCARD, CD47, FCER1G, NCF4, RAPGEF1, IGHV4-61, RAP1A, ITGAL, IGHV4-59, LEP, CD36, RAB27A, CYFIP1, C4BPA, SFTPD, FCN1, SLAMF1, LYAR, IGHV3-30, HSP90AA1, MYO1C, ARPC1B, RAB14, IGLC1, MERTK, ITGB2, CAMK1D, IGHV1-3, LBP, RACK1, IGHV4-4, COLEC12, ANXA11, CLEC7A, TMEM175, IGLV7-43, ABR, IGLV3-27, PTEN, IGHV2-26, ARHGAP12, IGLV3-19, ADORA1, TM9SF4, MYO1G, SIRPA, RAC1, IGHV3-15, IGLV1-40, LEPR, IGHV3-21, FCGR1A, ANXA3, RHOBTB2, CORO1A, YES1, IGHV3-53, WAS, GSN, FGR, DOCK2, HCK, FCN2, ACTR2, IGHG3, MEGF10, FCGR2A, IGLC2, PTPRJ, IGHA2, ABL1, IGHV3-11, ITGAV, BCR, CALR, IGLC7, STAP1, CDC42SE2, IGHV3-66, IGHV3-73, MIR17, CD14, CYBA, DYSF, ELMO1, PTK2, RAB20, IGHV3-13, IGHV1-18, PTPRC, WIPF1, ADIPOQ, IGLV2-14, IGLL5, IGHV1OR15-1, AHSG, IGLV1-47, CD302, FCN3, IGHV3-72, MIR20A, IGLV3-21, BRK1, ABI1, IGHV1-69, PLD4, NCKAP1L, PIK3CB, ADGRB1, ARHGAP25, MAPK3, MYO10, MYH9, PIP5K1C, IGHV3-74, IGHV6-1, IGHV3-64, NCK1, ARPC1A, ITGAM, ABCA7, MFGE8, IGLV1-44, NR1H3, IGLC3, IGHA1, MET, PRKCE, TGM2, CD300A, IGHG1, IGLV6-57, SRC, ELANE, IGHV4-34, GULP1, WIPF3, IGHV3-20, IGHV1-24, NCF2, XKR8, FCGR3A, F2RL1, IGHV2-5, NCKAP1, SIRPG, FYN, LIMK1, ITGB1, IGHV3-23 | 80 |
| 32 | GO:0038094 | Fc-gamma receptor signaling pathway | IGHV3-33, IGHG4, IGLC6, IGLV2-23, DOCK1, IGHV4-39, VAV2, IGHV3-7, PLCG2, IGLV3-25, WASL, IGHV3-48, IGLV2-8, IGHG2, IGLV2-11, ACTB, CD247, PLA2G6, PRKCE, IGLV3-1, IGHV2-70, FCGR2B, MAPK1, CRK, WASF2, GRB2, ARPC5, IGLV1-51, ARPC4, CD47, FCER1G, RAPGEF1, RAP1A, IGHV4-59, CYFIP1, IGHV3-30, HSP90AA1, MYO1C, ARPC1B, IGLC1, IGLV7-43, IGLV3-27, IGLV3-19, MYO1G, RAC1, IGLV1-40, FCGR1A, YES1, IGHV3-53, WAS, FGR, HCK, ACTR2, IGHG3, FCGR2A, PTPRJ, ABL1, IGHV3-11, IGLC7, ELMO1, PTK2, IGHV3-13, PTPRC, WIPF1, IGLV2-14, IGHV1OR15-1, IGLV1-47, IGLV3-21, BRK1, ABI1, IGHV1-69, NCKAP1L, PIK3CB, MAPK3, MYO10, NCK1, ARPC1A, IGLV1-44, IGHG1, IGLV6-57, SRC, IGHV4-34, WIPF3, FCGR3A, IGHV2-5, NCKAP1, FYN, LIMK1, IGHV3-23 | 80 |
| 32 | GO:1903317 | regulation of protein maturation | IGHV3-33, IGHG4, IGLC6, IGLV2-23, CR1, IGHV4-39, C8G, MELTF, IGHV3-7, IGLV3-25, CFHR4, PRSS37, CFHR5, IGHV3-48, IGLV2-8, IGHG2, IGLV2-11, USP17L2, NKD2, NOL3, C1QBP, IGLV3-1, CFHR2, IGHV2-70, C6, C4BPB, ACP4, IGLV1-51, CPB2, PRKACB, SOX4, CNTN2, IGHV4-59, CFHR1, C4BPA, KLKB1, C1QC, IGHV3-30, IGLC1, SERPINF2, ANXA2P2, F12, ADAM8, IGLV7-43, IGLV3-27, C1QB, IGLV3-19, CR2, XIAP, IGLV1-40, C9, PLGRKT, CFH, TMEM59, IGHV3-53, GSN, CPN2, IGHG3, TFR2, CD46, IGHV3-11, SUSD4, ENO1, IGLC7, CD55, TIMM17A, CARD8, IGHV3-13, IGLV2-14, CTSZ, IGHV1OR15-1, SERPINE2, IGLV1-47, ANGPTL8, A2M, IGLV3-21, IGHV1-69, MYH9, SERPINE1, NLRP7, BIRC7, IGLV1-44, BCL2L12, C1QA, IGHG1, IGLV6-57, SRC, C5, IGHV4-34, C7, IL1R2, IGHV2-5, CD19, CLU, TNP2, IGHV3-23, CCBE1, GLG1 | 80 |
| 32 | GO:0050853 | B cell receptor signaling pathway | IGLL1, IGHV3-33, IGHG4, IGLC6, KLHL6, BLK, IGHV4-28, IGHV4-39, IGHV5-51, IGHV3-7, PLCG2, IGHV1-45, IGHE, TRBC2, IGHV3-48, CTLA4, IGHG2, IGHD, NFAM1, IGHV3-43, IGHV1-58, IGHV2-70, IGHM, GCSAM, MAPK1, ELF2, SLC39A10, LAT2, IGHV3-49, IGHV4-61, IGHV4-59, CMTM3, MNDA, LIME1, PIK3CD, IGHV3-30, IGLC1, PAX5, IGHV1-3, IGHV4-4, IGHV2-26, CBFB, IGHV3-15, PLEKHA1, IGHV3-21, IGHV3-53, GCSAML, IGHG3, IGLC2, NFATC2, IGHA2, ABL1, IGHV3-11, PLCL2, IGLC7, STAP1, IGHV3-66, IGHV3-73, BCL2, BTK, MEF2C, IGHV3-13, IGHV1-18, PTPRC, IGLL5, FCRL3, IGHV1OR15-1, IGHV3-72, GPS2, IGHV1-69, CD38, NCKAP1L, IGHV3-74, IGHV6-1, IGHV3-64, IGLC3, IGHA1, CD300A, IGHG1, IGHV4-34, BAX, TEC, IGHV3-20, IGHV1-24, IGHV2-5, ELF1, CD19, RFTN1, IGHV3-23 | 80 |
| 32 | GO:0016064 | immunoglobulin mediated immune response | IGLL1, IGHV3-33, CD40LG, IGHG4, IGLC6, IGLV2-23, FOXP3, CR1, IGHV4-28, IGHV4-39, C8G, IGHV5-51, IGHV3-7, IGLV3-25, EXO1, MLH1, IGHV1-45, IGHE, TNFSF4, TRBC2, IGHV3-48, IGLV2-8, IGHG2, IGHD, IGLV2-11, IGHV3-43, BCL6, IL13RA2, IGHV1-58, APCS, C1QBP, IGLV3-1, IGHV2-70, IGHM, TNFSF13, FCGR2B, C6, C4BPB, XCL1, MAD2L2, IGLV1-51, IGHV3-49, CRP, SHLD2, FCER1G, IGHV4-61, THOC1, IL4, INPP5D, IGHV4-59, C4BPA, C1QC, IGHV3-30, RIF1, IGLC1, APLF, IGHV1-3, LIG4, IGHV4-4, IGLV7-43, IGLV3-27, C1QB, IGHV2-26, IGLV3-19, PAXIP1, CR2, HSPD1, IGHV3-15, IGLV1-40, CD28, C9, IGHV3-21, IGHV3-53, IGHG3, IGLC2, CD46, IGHA2, IL2, MASP2, IGHV3-11, SUSD4, IL10, IGLC7, CD55, IGHV3-66, IGHV3-73, BTK, IGHV3-13, GAPT, IGHV1-18, PTPRC, IGLV2-14, IGLL5, IGHV1OR15-1, IGLV1-47, IGHV3-72, ATAD5, NSD2, IGLV3-21, MSH6, IGHV1-69, CCR6, NBN, BCL10, RNF168, IGHV3-74, IGHV6-1, IGHV3-64, MSH2, NDFIP1, IL4R, IGLV1-44, IGLC3, IGHA1, C1QA, IGHG1, IGLV6-57, CD226, C5, IGHV4-34, C7, CLCF1, IGHV3-20, IGHV1-24, IGHV2-5, CLU, IGHV3-23, TFRC | 80 |
| 32 | GO:0099024 | plasma membrane invagination | IGLL1, IGHV3-33, IGHG4, IGLC6, MSR1, SH3BP1, DOCK1, IGHV4-28, IGHV4-39, IGHV5-51, IGHV3-7, IGHV1-45, IGHE, TRBC2, IGHV3-48, IGHG2, IGHD, IGHV3-43, IGHV1-58, IGHV2-70, IGHM, FCGR2B, PPARG, ALKBH4, IGHV3-49, FCER1G, IGHV4-61, IGHV4-59, CD36, IGHV3-30, IGLC1, ITGB2, IGHV1-3, IGHV4-4, IGHV2-26, ARHGAP12, IGHV3-15, IGHV3-21, FCGR1A, RHOBTB2, IGHV3-53, GSN, IGHG3, MEGF10, IGLC2, IGHA2, SPIRE2, IGHV3-11, IGLC7, STAP1, IGHV3-66, IGHV3-73, ELMO1, IGHV3-13, IGHV1-18, IGLL5, IGHV1OR15-1, IGHV3-72, ARF1, IGHV1-69, NCKAP1L, ADGRB1, ARHGAP25, MYH9, IGHV3-74, IGHV6-1, IGHV3-64, ITGAM, ABCA7, MFGE8, SNX9, IGLC3, IGHA1, CD300A, IGHG1, SPIRE1, IGHV4-34, SNX33, GULP1, IGHV3-20, IGHV1-24, XKR8, F2RL1, IGHV2-5, IGHV3-23 | 80 |
| 32 | GO:0038093 | Fc receptor signaling pathway | IGHV3-33, IGHG4, IGLC6, IGLV2-23, DOCK1, IGHV4-39, VAV2, IGHV3-7, PLCG2, IGLV3-25, PAK2, IKBKG, IGHE, WASL, IGHV3-48, IGLV2-8, LCP2, IGHG2, IGLV2-11, ACTB, RELA, CD247, CARD11, PLA2G6, PRKCE, IGLV3-1, IGHV2-70, FCGR2B, MAPK1, CRK, WASF2, GRB2, ARPC5, PPP3CB, LAT2, IGLV1-51, ARPC4, MAPK10, CD47, FCER1G, RAPGEF1, RAP1A, IGHV4-59, LILRA4, CYFIP1, IKBKB, IGHV3-30, HSP90AA1, MYO1C, ARPC1B, IGLC1, PPP3R1, PDPK1, IGLV7-43, IGLV3-27, IGLV3-19, MYO1G, RAC1, IGLV1-40, FCGR1A, MAP3K1, YES1, IGHV3-53, WAS, FGR, HCK, LAT, ACTR2, IGHG3, FCGR2A, PTPRJ, NFATC2, ABL1, IGHV3-11, IGLC7, MALT1, BTK, ELMO1, PTK2, IGHV3-13, PTPRC, WIPF1, CD200R1, IGLV2-14, IGHV1OR15-1, IGLV1-47, TAB1, IGLV3-21, BRK1, ABI1, IGHV1-69, BCL10, FCER1A, NCKAP1L, PIK3CB, MAPK3, MYO10, GRAP2, NCK1, ARPC1A, IGLV1-44, NFATC1, IGHG1, IGLV6-57, SRC, PIGR, IGHV4-34, WIPF3, TEC, FCGR3A, IGHV2-5, NCKAP1, PRKCQ, MAPK9, FYN, LIMK1, NR4A3, IGHV3-23 | 80 |
| 32 | GO:0002429 | immune response-activating cell surface receptor signaling pathway | TESPA1, IGLL1, IGHV3-33, CARD9, FPR1, RAB29, IGHG4, IGLC6, IGLV2-23, PVRIG, PAG1, CR1, CSK, KLHL6, FOXP3, DOCK1, IGHV4-28, IGHV4-39, DENND1B, DUSP22, IGHV5-51, IGHV3-7, VAV2, PLCG2, BLK, IGLV3-25, PAK2, IKBKG, IGHV1-45, IGHE, TRBC2, WASL, IGHV3-48, CTLA4, HRAS, IGLV2-8, PTPN2, CLEC10A, IGHG2, IGHD, NFAM1, IGLV2-11, LILRA2, ACTB, LCP2, RBCK1, MUC7, IGHV3-43, RELA, CD247, IGHV1-58, MUC17, FPR2, CARD11, PLA2G6, PRKCE, IGLV3-1, PAWR, IGHV2-70, IGHM, FCGR2B, MAPK1, CRK, WASF2, GCSAM, GRB2, ELF2, ARPC5, PHPT1, SLC39A10, LAT2, IGLV1-51, IGHV3-49, PDE4D, ARPC4, CD276, CD47, FCER1G, PRKACB, PAK3, RAPGEF1, IGHV4-61, RAP1A, INPP5D, TXK, IGHV4-59, CREBBP, MNDA, LIME1, CMTM3, KLRK1, TRAT1, BTNL9, PIK3CD, CYFIP1, IKBKB, FCN1, FPR3, IGHV3-30, HSP90AA1, MYO1C, ARPC1B, IGLC1, PAX5, PDPK1, IGHV1-3, IGHV4-4, IGLV7-43, EP300, IGLV3-27, RIPK2, IGHV2-26, ERMAP, IGLV3-19, SH2B2, DUSP3, CR2, MYO1G, CBFB, HHLA2, RAC1, IGHV3-15, IGLV1-40, MUCL1, MUC4, PLEKHA1, CD28, IGHV3-21, FCGR1A, MUC3A, EZR, YES1, IGHV3-53, WAS, CACNA1F, FGR, BTNL3, PLSCR1, LAT, HCK, GCSAML, RC3H2, ACTR2, IGHG3, FCGR2A, IGLC2, PTPRJ, NFATC2, IGHA2, GPR32, ABL1, IGHV3-11, FYB1, PLCL2, RC3H1, IGLC7, STAP1, MALT1, IGHV3-66, IGHV3-73, PRKACG, MUC12, BCL2, STOML2, BTK, ELMO1, MEF2C, RAF1, PTK2, IGHV3-13, LGALS3, PDE4B, IGHV1-18, PTPRC, WIPF1, BTNL8, GATA3, IGLV2-14, GPR32P1, IGLL5, FCRL3, IGHV1OR15-1, IGLV1-47, TAB1, IGHV3-72, IGLV3-21, GPS2, BRK1, ABI1, IGHV1-69, SLA2, BTNL10, CD38, BCL10, NCKAP1L, PIK3CB, MAPK3, MYO10, GRAP2, IGHV3-74, IGHV6-1, IGHV3-64, NCK1, ARPC1A, THEMIS2, KLRC4-KLRK1, IGLV1-44, IGLC3, IGHA1, LILRB4, CD300A, IGHG1, IGLV6-57, SRC, CD226, IGHV4-34, STK11, WIPF3, BAX, MUC20, IGHV3-20, IGHV1-24, MUC1, TEC, FCGR3A, IGHV2-5, ELF1, NCKAP1, CD19, PRKCQ, FYN, LIMK1, RFTN1, NR4A3, BRAF, IGHV3-23, EIF2B5 | 80 |
| 32 | GO:0050864 | regulation of B cell activation | IGLL1, IGHV3-33, IGHG4, IGLC6, FOXP3, IGHV4-28, IGHV4-39, IGHV5-51, IGHV3-7, MLH1, IGHV1-45, IGHE, TNFSF4, TRBC2, PCID2, IGHV3-48, CTLA4, IGHG2, IGHD, NFAM1, IGHV3-43, INHBA, BCL6, IGHV1-58, PAWR, CARD11, IGHV2-70, IGHM, TNFSF13, FCGR2B, AHR, TNFRSF13B, IL7, MAD2L2, SLC39A10, IGHV3-49, SHLD2, IGHV4-61, THOC1, IL4, INPP5D, IGHV4-59, MNDA, IL6, IGHV3-30, GPR183, RIF1, PPP2R3C, IGLC1, APLF, IGHV3-23, BAD, IGHV1-3, TNFRSF13C, IGHV4-4, IGHV2-26, IRS2, PAXIP1, IGHV3-15, CD28, IGHV3-21, IGHV3-53, IL5, MIF, IGHG3, IGLC2, NFATC2, IGHA2, IL2, IL10, IGHV3-11, RC3H1, IGLC7, IGHV3-66, IGHV3-73, TNIP2, BCL2, ZFP36L2, CASP3, BTK, IL21, MEF2C, ATP11C, IGHV3-13, IGHV1-18, PTPRC, IGLL5, FCRL3, IGHV1OR15-1, IGHV3-72, ATAD5, NSD2, MSH6, SASH3, IGHV1-69, CD38, TNFRSF4, NCKAP1L, WNT3A, IGHV3-74, IGHV6-1, BST1, IGHV3-64, MSH2, NDFIP1, IGLC3, IGHA1, MIR17HG, XBP1, CD300A, IGHG1, IGHV4-34, IL13, PKN1, CLCF1, TBC1D10C, TNFSF13B, IGHV3-20, IGHV1-24, SFRP1, IGHV2-5, PELI1, TICAM1, SLA2, ZFP36L1, TFRC, TIRAP | 80 |
| 32 | GO:0051249 | regulation of lymphocyte activation | CD83, TESPA1, IGLL1, SHH, IGHV3-33, EGR3, CD40LG, IRF1, GAS6, IGHG4, IGLC6, FOXP3, PAG1, SIRPB1, CR1, CSK, IRF4, TNFSF11, IGHV4-28, IGHV4-39, TMIGD2, DUSP22, IGHV5-51, IGHV3-7, TARM1, SOCS5, PAK2, EFNB2, AP3D1, MLH1, IGHV1-45, IGHE, IFNB1, TRBC2, CD1D, TNFSF4, PCID2, IGHV3-48, CTLA4, PTPN2, IGHG2, IGHD, NFAM1, FBXO7, CAV1, DOCK8, IGHV3-43, TNFRSF14, CLECL1, DNAJA3, INHBA, BCL6, IGHV1-58, PAWR, CARD11, IDO1, ANXA1, BMP4, HMGB1, IGHV2-70, IGHM, TNFSF13, FCGR2B, AHR, TNFRSF13B, GRB2, IL7, TAC1, XCL1, VCAM1, SOCS6, PGLYRP2, MARCH7, ICOS, MAD2L2, SPN, SLC39A10, IGHV3-49, MAPK8IP1, SHLD2, PYCARD, CD274, ITPKB, MAD1L1, CD276, CD47, PAK3, IGHV4-61, DPP4, THOC1, PIBF1, IL4, INPP5D, IGHV4-59, KLRK1, MNDA, LEP, MIR30B, PDE5A, SFTPD, MIR21, PLA2G2F, IL6, IGHV3-30, GLI3, GPR183, RIF1, SART1, PPP2R3C, IL6ST, FADD, IGLC1, APLF, MERTK, IGHV3-23, BAD, FANCA, PDPK1, IGHV1-3, FLT3LG, TNFRSF13C, FGF10, IGHV4-4, FANCD2, ADAM8, IFNA2, RIPK2, RHBDD3, IGHV2-26, NLRP3, IRS2, PAXIP1, DUSP3, HSPD1, CBFB, SIRPA, SOX13, HHLA2, RAC1, GNRH1, IGHV3-15, CD28, IGHV3-21, LAMP1, YES1, IL20RB, CORO1A, MAP3K8, IGHV3-53, PDCD1LG2, IL5, GSN, LAT, IL23R, NRARP, LILRB1, MIF, RC3H2, GPAM, IGHG3, IGLC2, CD46, NFATC2, IGHA2, IL2, IL10, IGHV3-11, EBI3, ABL1, ZP4, HES1, NKAP, RC3H1, IL27, IGLC7, CD55, MALT1, IGHV3-66, IGHV3-73, TNIP2, LGALS1, PRKCZ, BCL2, ZFP36L2, CASP3, BTK, ZC3H8, IL21, MEF2C, ATP11C, AP1G1, IL2RA, PLA2G2D, IGHV3-13, LGALS3, IGHV1-18, PTPRC, GATA3, HSPH1, SPTA1, IL1RL2, SIT1, IGLL5, FCRL3, IGHV1OR15-1, CD86, IGHV3-72, HLX, ZEB1, ATAD5, NSD2, MSH6, SASH3, IGHV1-69, SLA2, EPO, CD38, BCL10, TNFRSF4, RAC2, TNFSF18, NCKAP1L, WNT3A, IL12B, IL12A, GRAP2, EFNB3, IGHV3-74, IGHV6-1, TNFRSF18, SOCS1, BST1, IGHV3-64, MSH2, NCK1, TWSG1, RASAL3, IL15, NDFIP1, IL4R, KLRC4-KLRK1, AKT1, CD80, ITCH, IGLC3, IGHA1, MIR17HG, CCDC88B, PDCD1, XBP1, LILRB4, GPNMB, CD300A, IGHG1, SRC, DUSP10, DLG1, SOD1, IGHV4-34, PRDM1, PKN1, CLCF1, TBC1D10C, TNFSF13B, TIGIT, TMEM131L, IGHV3-20, IGHV1-24, CEBPB, LILRB2, SFRP1, IGHV2-5, SIRPG, PRKCQ, LAX1, FYN, BTLA, PELI1, TICAM1, BRAF, IL13, ZFP36L1, PRELID1, TFRC, TIRAP | 80 |
| 32 | GO:0002449 | lymphocyte mediated immunity | CTSH, IGLL1, IGHV3-33, CD40LG, IGHG4, TUBB4B, IGLV2-23, IGLC6, FOXP3, CR1, NCR1, DENND1B, IGHV4-28, IGHV4-39, C8G, IGHV5-51, IGHV3-7, DUSP22, IGLV3-25, EXO1, MLH1, IGHV1-45, IGHE, IFNB1, CD1D, TRBC2, TNFSF4, TSTA3, IGHV3-48, IGLV2-8, SCART1, IGHG2, IGHD, IGLV2-11, IGHV3-43, BCL6, IL13RA2, IGHV1-58, TRAF2, APCS, SH2D1A, C1QBP, IGLV3-1, HMGB1, IGHV2-70, IGHM, FCGR2B, TRPM4, CRK, TNFSF13, C6, C4BPB, PPP3CB, XCL1, MAD2L2, IGLV1-51, IGHV3-49, CRP, SHLD2, FCER1G, IGHV4-61, THOC1, IL4, INPP5D, IGHV4-59, KLRK1, LEP, RAB27A, C4BPA, IL6, IGHV3-30, C1QC, RIF1, KLRF2, IGLC1, FADD, APLF, CLEC12B, IGHV1-3, LIG4, IGHV4-4, GZMM, IGLV7-43, IFNA2, IGLV3-27, C1QB, IGHV2-26, NLRP3, IGLV3-19, PAXIP1, SERPINB4, CR2, MYO1G, HSPD1, IGHV3-15, IGLV1-40, CD28, C9, IGHV3-21, LAMP1, CORO1A, IL20RB, IGHV3-53, WAS, IL23R, LILRB1, IGHG3, CD1A, IGLC2, CD46, IGHA2, IL2, MASP2, IGHV3-11, CD1C, SUSD4, IL10, IGLC7, CD55, MALT1, IGHV3-66, IGHV3-73, PRKCZ, PIK3R6, LYST, BTK, CD1B, IL1R1, IL21, AP1G1, IGHV3-13, GAPT, IGHV1-18, PTPRC, SLAMF6, KIR3DL1, GATA3, IGLV2-14, CD1E, EMP2, IGLL5, KLRD1, IGHV1OR15-1, CD96, IGLV1-47, IGHV3-72, ATAD5, NSD2, IGLV3-21, MSH6, SASH3, IGHV1-69, SLA2, CCR6, RSAD2, NBN, BCL10, SERPINB9, IL12B, IL12A, RNF168, KDELR1, IGHV3-74, IGHV6-1, IGHV3-64, MSH2, KDM5D, SLAMF7, IL18RAP, NDFIP1, IL4R, KLRC4-KLRK1, IGLV1-44, IGLC3, IGHA1, FZD5, IL18R1, C1QA, IGHG1, HPRT1, IGLV6-57, DLG1, CD226, C5, IL31RA, KLRC2, IGHV4-34, C7, CLCF1, CLEC2A, IGHV3-20, IGHV1-24, IGHV2-5, RFTN1, CLU, IGHV3-23, TFRC | 80 |
| 32 | GO:0002433 | immune response-regulating cell surface receptor signaling pathway involved in phagocytosis | IGHV3-33, IGHG4, IGLC6, IGLV2-23, DOCK1, IGHV4-39, VAV2, IGHV3-7, PLCG2, IGLV3-25, WASL, IGHV3-48, IGLV2-8, IGHG2, IGLV2-11, ACTB, CD247, PLA2G6, PRKCE, IGLV3-1, IGHV2-70, FCGR2B, MAPK1, CRK, WASF2, GRB2, ARPC5, IGLV1-51, ARPC4, CD47, RAPGEF1, RAP1A, IGHV4-59, CYFIP1, IGHV3-30, HSP90AA1, MYO1C, ARPC1B, IGLC1, IGLV7-43, IGLV3-27, IGLV3-19, MYO1G, RAC1, IGLV1-40, FCGR1A, YES1, IGHV3-53, WAS, FGR, HCK, ACTR2, IGHG3, FCGR2A, PTPRJ, ABL1, IGHV3-11, IGLC7, ELMO1, PTK2, IGHV3-13, PTPRC, WIPF1, IGLV2-14, IGHV1OR15-1, IGLV1-47, IGLV3-21, BRK1, ABI1, IGHV1-69, NCKAP1L, PIK3CB, MAPK3, MYO10, NCK1, ARPC1A, IGLV1-44, IGHG1, IGLV6-57, SRC, IGHV4-34, WIPF3, FCGR3A, IGHV2-5, NCKAP1, FYN, LIMK1, IGHV3-23 | 80 |
| 32 | GO:0002696 | positive regulation of leukocyte activation | CD83, TESPA1, IGLL1, SHH, IGHV3-33, EGR3, CD40LG, SIRPB1, GAS6, IGHG4, IGLC6, FOXP3, CSK, TNFSF11, IGHV4-28, IGHV4-39, TMIGD2, IGHV5-51, IGHV3-7, PAK2, EFNB2, AP3D1, MLH1, IGHV1-45, IGHE, CD1D, TRBC2, TNFSF4, PCID2, IGHV3-48, CTLA4, IGHG2, IGHD, CAV1, DOCK8, IGHV3-43, TNFRSF14, CLECL1, DNAJA3, BCL6, IGHV1-58, CARD11, ANXA1, HMGB1, IGHV2-70, IGHM, TNFSF13, GRB2, IL7, TAC1, XCL1, VCAM1, PTAFR, ICOS, MAD2L2, SLC39A10, IGHV3-49, SHLD2, PYCARD, CD274, ITPKB, FCER1G, CD276, CD47, PAK3, IGHV4-61, DPP4, IL4, INPP5D, IGHV4-59, KLRK1, MIR30B, LEP, MIR21, SART1, IL6, IGHV3-30, GLI3, GPR183, RIF1, PPP2R3C, IL6ST, FADD, IGLC1, IGHV3-23, BAD, ITGB2, CRLF2, PDPK1, IGHV1-3, FLT3LG, TNFRSF13C, LBP, FGF10, IGHV4-4, ADAM8, RIPK2, IGHV2-26, NLRP3, IRS2, PAXIP1, IL1RL1, HSPD1, CBFB, SIRPA, HHLA2, RAC1, STXBP1, IGHV3-15, CD28, IGHV3-21, LAMP1, YES1, CORO1A, MAP3K8, IGHV3-53, PDCD1LG2, IL5, FGR, IL23R, ADORA2B, LILRB1, MIF, GPAM, IGHG3, IGLC2, CD46, NFATC2, IGHA2, IL2, IL10, IGHV3-11, EBI3, SOCS5, ZP4, HES1, NKAP, IGLC7, CD55, STAP1, MALT1, IGHV3-66, IGHV3-73, TNIP2, LGALS1, PRKCZ, BCL2, BTK, IL21, MEF2C, ATP11C, AP1G1, IL2RA, IGHV3-13, IGHV1-18, PTPRC, GATA3, HSPH1, SPTA1, IL1RL2, IGLL5, FCRL3, IGHV1OR15-1, CD86, IGHV3-72, HLX, ATAD5, NSD2, MSH6, NPPA, SASH3, IGHV1-69, EPO, CD38, BCL10, TNFRSF4, NCKAP1L, WNT3A, IL12B, IL12A, GRAP2, EFNB3, IGHV3-74, IGHV6-1, SOCS1, BST1, IGHV3-64, MSH2, NCK1, RASAL3, ITGAM, IL15, IL4R, KLRC4-KLRK1, AKT1, CD80, IGLC3, IGHA1, CCDC88B, PDCD1, XBP1, LILRB4, IGHG1, SRC, DUSP10, CD226, IGHV4-34, IL33, CLCF1, STX4, TNFSF13B, IGHV3-20, IGHV1-24, LILRB2, F2RL1, IGHV2-5, SIRPG, PRKCQ, NR4A3, FYN, BTLA, PELI1, TICAM1, IL13, TFRC, TIRAP | 80 |
| 32 | GO:0006956 | complement activation | IGLL1, IGHV3-33, IGHG4, IGLC6, IGLV2-23, CR1, IGHV4-28, IGHV4-39, C8G, IGHV5-51, IGHV3-7, KRT1, IGLV3-25, CFHR4, IGHV1-45, IGHE, TRBC2, CFHR5, IGHV3-48, IGLV2-8, IGHG2, IGHD, IGLV2-11, IGHV3-43, IGHV1-58, APCS, C1QBP, IGLV3-1, CFHR2, IGHV2-70, IGHM, C6, C4BPB, IGLV1-51, CPB2, IGHV3-49, SCARA3, CRP, IGHV4-61, IGHV4-59, CFHR1, C4BPA, FCN1, C1QC, IGHV3-30, IGLC1, IGHV1-3, IGHV4-4, IGLV7-43, IGLV3-27, C1QB, IGHV2-26, IGLV3-19, CR2, IGHV3-15, IGLV1-40, C9, CFH, IGHV3-21, RGCC, IGHV3-53, CFD, CPN2, FCN2, IGHG3, IGLC2, CD46, IGHA2, MASP2, IGHV3-11, SUSD4, IGLC7, CD55, IGHV3-66, IGHV3-73, IGHV3-13, IGHV1-18, COL20A1, MASP1, IGLV2-14, IGLL5, IGHV1OR15-1, IGLV1-47, FCN3, IGHV3-72, A2M, IGLV3-21, IGHV1-69, IGHV3-74, IGHV6-1, IGHV3-64, COLEC10, VSIG4, IGLV1-44, IGLC3, IGHA1, C1QA, IGHG1, IGLV6-57, C5, IGHV4-34, C7, IGHV3-20, IGHV1-24, IGHV2-5, CD19, CLU, IGHV3-23 | 80 |
| 32 | GO:0051251 | positive regulation of lymphocyte activation | CD83, TESPA1, IGLL1, SHH, IGHV3-33, EGR3, CD40LG, SIRPB1, GAS6, IGHG4, IGLC6, FOXP3, CSK, TNFSF11, IGHV4-28, IGHV4-39, TMIGD2, IGHV5-51, IGHV3-7, PAK2, EFNB2, AP3D1, MLH1, IGHV1-45, IGHE, CD1D, TRBC2, TNFSF4, PCID2, IGHV3-48, CTLA4, IGHG2, IGHD, CAV1, DOCK8, IGHV3-43, TNFRSF14, CLECL1, DNAJA3, BCL6, IGHV1-58, CARD11, ANXA1, HMGB1, IGHV2-70, IGHM, TNFSF13, GRB2, IL7, TAC1, XCL1, VCAM1, ICOS, MAD2L2, SLC39A10, IGHV3-49, SHLD2, PYCARD, CD274, ITPKB, CD276, CD47, PAK3, IGHV4-61, DPP4, IL4, INPP5D, IGHV4-59, KLRK1, MIR30B, LEP, MIR21, SART1, IL6, IGHV3-30, GLI3, GPR183, RIF1, PPP2R3C, IL6ST, FADD, IGLC1, IGHV3-23, BAD, PDPK1, IGHV1-3, FLT3LG, TNFRSF13C, FGF10, IGHV4-4, ADAM8, RIPK2, IGHV2-26, NLRP3, IRS2, PAXIP1, HSPD1, CBFB, SIRPA, HHLA2, RAC1, IGHV3-15, CD28, IGHV3-21, LAMP1, YES1, CORO1A, MAP3K8, IGHV3-53, PDCD1LG2, IL5, IL23R, LILRB1, MIF, GPAM, IGHG3, IGLC2, NFATC2, CD46, IGHA2, IL2, EBI3, IGHV3-11, SOCS5, ZP4, HES1, NKAP, IGLC7, CD55, MALT1, IGHV3-66, IGHV3-73, TNIP2, LGALS1, PRKCZ, BCL2, BTK, IL21, MEF2C, ATP11C, AP1G1, IL2RA, IGHV3-13, IGHV1-18, PTPRC, GATA3, HSPH1, SPTA1, IL1RL2, IGLL5, FCRL3, IGHV1OR15-1, CD86, IGHV3-72, HLX, ATAD5, NSD2, MSH6, SASH3, IGHV1-69, EPO, CD38, BCL10, TNFRSF4, NCKAP1L, WNT3A, IL12B, IL12A, GRAP2, EFNB3, IGHV3-74, IGHV6-1, SOCS1, BST1, IGHV3-64, MSH2, NCK1, RASAL3, IL15, IL4R, KLRC4-KLRK1, AKT1, CD80, IGLC3, IGHA1, CCDC88B, PDCD1, XBP1, LILRB4, IGHG1, SRC, DUSP10, IGHV4-34, CLCF1, TNFSF13B, IGHV3-20, IGHV1-24, LILRB2, IGHV2-5, SIRPG, PRKCQ, FYN, BTLA, PELI1, TICAM1, IL13, TFRC, TIRAP | 80 |
| 32 | GO:0002768 | immune response-regulating cell surface receptor signaling pathway | TESPA1, IGLL1, IGHV3-33, CARD9, FPR1, RAB29, IGHG4, IGLC6, IGLV2-23, PVRIG, PAG1, CR1, CSK, KLHL6, FOXP3, DOCK1, IGHV4-28, IGHV4-39, DENND1B, DUSP22, IGHV5-51, IGHV3-7, VAV2, PLCG2, BLK, IGLV3-25, PAK2, IKBKG, IGHV1-45, IGHE, TRBC2, WASL, IGHV3-48, CTLA4, HRAS, IGLV2-8, PTPN2, CLEC10A, IGHG2, IGHD, NFAM1, IGLV2-11, LILRA2, ACTB, LCP2, RBCK1, MUC7, IGHV3-43, RELA, CD247, IGHV1-58, MUC17, FPR2, CARD11, PLA2G6, PRKCE, IGLV3-1, PAWR, IGHV2-70, IGHM, FCGR2B, MAPK1, CRK, WASF2, GCSAM, GRB2, ELF2, ARPC5, PPP3CB, PHPT1, SLC39A10, LAT2, IGLV1-51, IGHV3-49, PDE4D, ARPC4, MAPK10, CD276, CD47, FCER1G, PRKACB, PAK3, RAPGEF1, IGHV4-61, RAP1A, INPP5D, TXK, IGHV4-59, CREBBP, MNDA, LIME1, CMTM3, KLRK1, TRAT1, BTNL9, PIK3CD, LILRA4, CYFIP1, IKBKB, FCN1, FPR3, IGHV3-30, HSP90AA1, MYO1C, ARPC1B, IGLC1, PAX5, CLEC12B, PPP3R1, PDPK1, IGHV1-3, IGHV4-4, IGLV7-43, EP300, IGLV3-27, RIPK2, IGHV2-26, ERMAP, IGLV3-19, SH2B2, DUSP3, CR2, MYO1G, CBFB, HHLA2, RAC1, IGHV3-15, IGLV1-40, MUCL1, MUC4, PLEKHA1, CD28, IGHV3-21, FCGR1A, MUC3A, MAP3K1, EZR, YES1, IGHV3-53, WAS, CACNA1F, FGR, BTNL3, PLSCR1, LAT, HCK, GCSAML, RC3H2, LILRB1, ACTR2, IGHG3, FCGR2A, IGLC2, PTPRJ, NFATC2, IGHA2, GPR32, ABL1, IGHV3-11, FYB1, PLCL2, RC3H1, IGLC7, STAP1, MALT1, IGHV3-66, IGHV3-73, PRKACG, MUC12, BCL2, STOML2, BTK, ELMO1, MEF2C, RAF1, IGHV3-13, LGALS3, PDE4B, IGHV1-18, PTPRC, WIPF1, CD200R1, BTNL8, GATA3, IGLV2-14, GPR32P1, IGLL5, FCRL3, IGHV1OR15-1, IGLV1-47, TAB1, IGHV3-72, IGLV3-21, GPS2, BRK1, ABI1, IGHV1-69, SLA2, BTNL10, CD38, BCL10, FCER1A, NCKAP1L, PIK3CB, MAPK3, MYO10, GRAP2, IGHV3-74, IGHV6-1, IGHV3-64, NCK1, ARPC1A, THEMIS2, KLRC4-KLRK1, IGLV1-44, IGLC3, IGHA1, LILRB4, NFATC1, CD300A, IGHG1, IGLV6-57, KIR2DL1, CD226, SRC, PIGR, IGHV4-34, STK11, WIPF3, BAX, MUC20, IGHV3-20, IGHV1-24, MUC1, TEC, LILRB2, FCGR3A, IGHV2-5, ELF1, NCKAP1, CD19, PRKCQ, FYN, LIMK1, RFTN1, BTLA, BRAF, MAPK9, NR4A3, IGHV3-23, EIF2B5 | 80 |
| 32 | GO:0010324 | membrane invagination | SYT11, IGLL1, IGHV3-33, IGHG4, IGLC6, MSR1, SH3BP1, DOCK1, IGHV4-28, IGHV4-39, IGHV5-51, IGHV3-7, IGHV1-45, IGHE, TRBC2, IGHV3-48, IGHG2, IGHD, IGHV3-43, IGHV1-58, IGHV2-70, IGHM, FCGR2B, PPARG, ALKBH4, IGHV3-49, FCER1G, IGHV4-61, IGHV4-59, CD36, IGHV3-30, IGLC1, ITGB2, IGHV1-3, IGHV4-4, IGHV2-26, ARHGAP12, IGHV3-15, IGHV3-21, FCGR1A, RHOBTB2, IGHV3-53, GSN, IGHG3, MEGF10, IGLC2, IGHA2, SPIRE2, IGHV3-11, IGLC7, STAP1, IGHV3-66, IGHV3-73, ELMO1, IGHV3-13, IGHV1-18, IGLL5, IGHV1OR15-1, IGHV3-72, ARF1, IGHV1-69, NCKAP1L, ADGRB1, ARHGAP25, MYH9, IGHV3-74, IGHV6-1, IGHV3-64, ITGAM, ABCA7, MFGE8, SNX9, IGLC3, IGHA1, CD300A, IGHG1, SPIRE1, IGHV4-34, SNX33, GULP1, IGHV3-20, IGHV1-24, XKR8, F2RL1, SMURF1, IGHV2-5, IGHV3-23 | 80 |
| 32 | GO:0002673 | regulation of acute inflammatory response | IGHV3-33, PARK7, IGHG4, IGLC6, IGLV2-23, CR1, TNFSF11, IGHV4-39, C8G, IGHV3-7, CFHR4, IGLV3-25, MIR92A2, GSTP1, CFHR5, IGHV3-48, IGLV2-8, IGHG2, IGLV2-11, PIK3CG, APCS, C1QBP, IGLV3-1, CFHR2, OSMR, IGHV2-70, FCGR2B, C6, TAC1, C4BPB, PPARG, IGLV1-51, CPB2, FCER1G, IGHV4-59, CFHR1, C4BPA, KLKB1, TNFRSF11A, IGHV3-30, IL6, C1QC, IL6ST, IGLC1, F12, ADAM8, IGLV7-43, C1QB, IGLV3-27, RHBDD3, ADORA1, NLRP3, IGLV3-19, CR2, IGLV1-40, C9, CFH, IL20RB, IGHV3-53, SELENOS, CPN2, MIR92A1, IGHG3, CD46, SUSD4, IGHV3-11, IGLC7, CD55, OSM, CREB3L3, DNASE1, BTK, ADCYAP1, IGHV3-13, IGLV2-14, IGHV1OR15-1, IGLV1-47, DNASE1L3, A2M, IGLV3-21, IGHV1-69, EDNRB, ASH1L, PTGS2, PTGER3, IGLV1-44, C1QA, IGHG1, IGLV6-57, C5, IGHV4-34, C7, IGHV2-5, CD19, CLU, IGHV3-23 | 80 |
| 32 | GO:0002920 | regulation of humoral immune response | IGHV3-53, IGLV3-21, IGHV3-33, CXCL13, IGHV1-69, IGHG4, IGLC6, IGLV2-23, CPN2, CR1, ACOD1, IGHV4-59, IGHV4-39, CFHR1, C8G, IGHG3, IGHV3-7, CD46, C4BPA, CFHR4, IGLV3-25, SUSD4, IGHV3-11, ZP4, IGHV3-30, C1QC, PPP2R3C, CFHR5, IGLC7, CD55, IGHV3-48, IGLC1, IGLV2-8, IGLV1-44, IGHG2, IGLV2-11, C1QA, IGHG1, IGLV6-57, IGLV7-43, C5, IGHV3-13, IGLV3-27, IGHV4-34, PTPRC, IGLV3-1, CFHR2, IGLV3-19, IGHV2-70, C1QB, FCGR2B, C7, C1QBP, IGLV2-14, CR2, C6, KLK5, C4BPB, IGHV2-5, IGHV1OR15-1, KLK7, IGLV1-47, IGLV1-40, CD19, C9, CFH, CLU, IGLV1-51, IGHV3-23, CPB2, A2M | 80 |
| 32 | GO:2000257 | regulation of protein activation cascade | IGHV3-53, IGLV3-21, SERPINC1, IGHV3-33, IGHV1-69, IGHG4, IGLC6, IGLV2-23, CPN2, CR1, IGHV4-59, IGHV4-39, IGHG3, CFHR1, C8G, IGHV3-7, CD46, C4BPA, CFHR4, IGLV3-25, SUSD4, IGHV3-11, C1QC, IGHV3-30, CFHR5, IGLC7, CD55, IGHV3-48, IGLC1, IGLV2-8, IGLV1-44, IGHG2, IGLV2-11, C1QA, IGHG1, IGLV6-57, IGLV7-43, C5, IGHV3-13, IGLV3-27, IGHV4-34, IGLV3-1, C1QB, CFHR2, IGLV3-19, IGHV2-70, C7, C1QBP, IGLV2-14, CR2, C6, C4BPB, IGHV2-5, IGHV1OR15-1, IGLV1-47, IGLV1-40, CD19, C9, CFH, CLU, IGLV1-51, IGHV3-23, CPB2, A2M | 80 |
| 32 | GO:0006898 | receptor-mediated endocytosis | SYT11, AMN, AP1S1, CLTCL1, IGHV3-33, CACNG3, CLTC, IGLC6, IGLV2-23, ACHE, AP2A1, MSR1, IGHV4-39, OPHN1, GPR107, IGHV3-7, PLCG2, IGLV3-25, EFNB2, CANX, CACNG5, WASL, EPS15, IGHV3-48, CALCRL, IGLV2-8, SCART1, IGLV2-11, GSG1L, SCYL2, CAV1, LRP1B, CAV3, ILDR1, CD63, VLDLR, SFRP4, RAMP1, LRP2, IGLV3-1, CLEC9A, IGHV2-70, FCGR2B, NEDD4, CACNG8, GRB2, IGF2R, MIR199A2, HTR1B, SCRIB, CLTB, IGLV1-51, SCARA3, FCER1G, SYNJ1, DAB2, CNTN2, IGHV4-59, CD36, SFTPD, SGIP1, IGHV3-30, HSP90AA1, SCARF1, IGLC1, MIR185, JCHAIN, GRK4, ITGB2, DBNL, ASGR2, FMR1, COLEC12, ANXA2P2, NECAB2, IGLV7-43, SCGB3A2, IGLV3-27, PLA2R1, IGLV3-19, DNM1P34, LRRTM2, CD5L, RAC1, SUSD2, IGLV1-40, HHIPL1, FCGR1A, EZR, IGHV3-53, CLTA, SMAP1, LILRB1, MKLN1, CACNG2, SH3GL2, MEGF10, TFR2, SCARA5, RABEPK, APOL1, IGHA2, TBC1D5, IGHV3-11, HIP1, PICK1, CD207, CALR, IGLC7, MICALL1, CD14, DNM1, HTR2B, DLL1, SELE, ITGA4, AAK1, CACNG4, PCSK9, IGHV3-13, MASP1, IGLV2-14, LOXL2, HSPH1, TINAGL1, CLN3, RAB31, CAV2, APOBR, IGHV1OR15-1, LDLR, IGLV1-47, PDLIM7, DMBT1, DNM3, PRG4, IGLV3-21, ASGR1, IGHV1-69, TNK2, ENPP2, GAK, LRPAP1, PIK3CB, ATAD1, CALY, PIP5K1C, CUBN, SERPINE1, SNCA, CACNG7, MRC1, IGLV1-44, ACKR3, SNX9, IGHA1, IGLV6-57, RAMP3, IGHV4-34, RAB21, MAGI2, PRSS12, DRD3, LRP6, IGHV2-5, CLU, DLG4, ITGB1, IGHV3-23, AP2M1, TFRC | 80 |
| 32 | GO:0050871 | positive regulation of B cell activation | IGLL1, IGHV3-33, IGHG4, IGLC6, IGHV4-28, IGHV4-39, IGHV5-51, IGHV3-7, MLH1, IGHV1-45, IGHE, TNFSF4, TRBC2, PCID2, IGHV3-48, IGHG2, IGHD, IGHV3-43, BCL6, IGHV1-58, CARD11, IGHV2-70, IGHM, TNFSF13, IL7, MAD2L2, SLC39A10, IGHV3-49, SHLD2, IGHV4-61, IL4, INPP5D, IGHV4-59, IL6, IGHV3-30, GPR183, RIF1, PPP2R3C, IGLC1, IGHV3-23, BAD, IGHV1-3, TNFRSF13C, IGHV4-4, IGHV2-26, IRS2, PAXIP1, IGHV3-15, CD28, IGHV3-21, IGHV3-53, IL5, MIF, IGHG3, IGLC2, NFATC2, IGHA2, IL2, IGHV3-11, IGLC7, IGHV3-66, IGHV3-73, TNIP2, BCL2, BTK, IL21, MEF2C, ATP11C, IGHV3-13, IGHV1-18, PTPRC, IGLL5, FCRL3, IGHV1OR15-1, IGHV3-72, ATAD5, NSD2, MSH6, SASH3, IGHV1-69, CD38, TNFRSF4, NCKAP1L, WNT3A, IGHV3-74, IGHV6-1, BST1, IGHV3-64, MSH2, IGLC3, IGHA1, XBP1, IGHG1, IGHV4-34, CLCF1, TNFSF13B, IGHV3-20, IGHV1-24, IGHV2-5, PELI1, TICAM1, IL13, TFRC, TIRAP | 80 |
| 32 | GO:0002431 | Fc receptor mediated stimulatory signaling pathway | IGHV3-33, IGHG4, IGLC6, IGLV2-23, CSK, DOCK1, IGHV4-39, VAV2, IGHV3-7, PLCG2, IGLV3-25, WASL, IGHV3-48, IGLV2-8, IGHG2, IGLV2-11, ACTB, CD247, PLA2G6, PRKCE, IGLV3-1, IGHV2-70, FCGR2B, MAPK1, CRK, WASF2, GRB2, ARPC5, IGLV1-51, ARPC4, CD47, FCER1G, RAPGEF1, RAP1A, IGHV4-59, CYFIP1, IGHV3-30, HSP90AA1, MYO1C, ARPC1B, IGLC1, IGLV7-43, IGLV3-27, IGLV3-19, MYO1G, RAC1, IGLV1-40, FCGR1A, YES1, IGHV3-53, WAS, FGR, PLSCR1, HCK, ACTR2, IGHG3, FCGR2A, PTPRJ, ABL1, IGHV3-11, IGLC7, ELMO1, PTK2, IGHV3-13, PTPRC, WIPF1, IGLV2-14, IGHV1OR15-1, IGLV1-47, IGLV3-21, BRK1, ABI1, IGHV1-69, NCKAP1L, PIK3CB, MAPK3, MYO10, NCK1, ARPC1A, IGLV1-44, IGHG1, IGLV6-57, SRC, CD226, IGHV4-34, WIPF3, FCGR3A, IGHV2-5, NCKAP1, NR4A3, FYN, LIMK1, IGHV3-23 | 80 |
| 32 | GO:0006911 | phagocytosis, engulfment | IGHV3-53, FCER1G, IGLL1, GSN, IGHV3-33, IGHV1-69, IGHV4-61, IGHG4, IGLC6, NCKAP1L, IGHV4-59, SH3BP1, DOCK1, IGHV4-28, IGHV4-39, MSR1, IGHG3, IGHV5-51, IGHV3-7, MEGF10, CD36, ARHGAP25, IGLC2, MYH9, IGHA2, IGHV3-74, IGHV6-1, IGHV3-11, IGHV3-30, IGHE, IGHV1-45, IGHV3-64, TRBC2, ITGAM, IGLC7, IGLC1, IGHV3-48, STAP1, ABCA7, IGHV3-66, IGHV3-73, MFGE8, ADGRB1, IGHG2, IGHD, ITGB2, IGLC3, IGHA1, IGHV1-3, IGHV3-43, CD300A, IGHG1, IGHV4-4, ELMO1, IGHV1-58, IGHV3-13, IGHV4-34, IGHV1-18, IGHV2-26, ARHGAP12, GULP1, IGHV2-70, IGHM, FCGR2B, IGHV3-20, IGHV1-24, XKR8, F2RL1, IGLL5, IGHV2-5, PPARG, IGHV1OR15-1, IGHV3-15, IGHV3-21, IGHV3-72, FCGR1A, IGHV3-23, RHOBTB2, IGHV3-49 | 80 |
| 32 | GO:0002460 | adaptive immune response based on somatic recombination of immune receptors built from immunoglobulin superfamily domains | CTSH, IGLL1, IGHV3-33, CXCL13, CD40LG, IGHG4, IGLC6, IGLV2-23, FOXP3, CR1, KLHL6, IRF4, DENND1B, IGHV4-28, IGHV4-39, C8G, IGHV5-51, IGHV3-7, DUSP22, IGLV3-25, EXO1, MLH1, IGHV1-45, IGHE, IFNB1, CD1D, TRBC2, TNFSF4, TSTA3, IGHV3-48, HRAS, IGLV2-8, SCART1, IGHG2, IGHD, IGLV2-11, IGHV3-43, BCL6, IL13RA2, IGHV1-58, TRAF2, APCS, ANXA1, IGLV3-1, HMGB1, C1QBP, IGHV2-70, IGHM, FCGR2B, TRPM4, TNFSF13, C6, C4BPB, PPP3CB, XCL1, MAD2L2, SPN, IGLV1-51, IGHV3-49, CRP, SHLD2, CD274, FCER1G, IGHV4-61, THOC1, IL4, INPP5D, IGHV4-59, RAB27A, C4BPA, MIR21, LY9, IL6, IGHV3-30, C1QC, RIF1, FADD, IGLC1, APLF, IGHV1-3, TNFRSF13C, LIG4, IGHV4-4, GZMM, IGLV7-43, IFNA2, IGLV3-27, RIPK2, IGHV2-26, NLRP3, IGLV3-19, C1QB, PAXIP1, IL1RL1, MTOR, CR2, MYO1G, HSPD1, IGHV3-15, IGLV1-40, SEMA4A, CD28, C9, IGHV3-21, IL20RB, IGHV3-53, WAS, IL23R, RC3H2, LILRB1, IGHG3, CD1A, IGLC2, CD46, SOCS5, IGHA2, IL2, MASP2, IGHV3-11, CD1C, SUSD4, IL10, EBI3, RC3H1, IL27, IGLC7, CD55, MALT1, IGHV3-66, IGHV3-73, PRKCZ, BTK, CD1B, IL1R1, MEF2C, IGHV3-13, GAPT, IGHV1-18, PTPRC, SLAMF6, GATA3, IGLV2-14, CD1E, EMP2, IGLL5, IGHV1OR15-1, IGLV1-47, IGHV3-72, HLX, ATAD5, NSD2, IGLV3-21, MSH6, SASH3, IGHV1-69, SLA2, CCR6, RSAD2, NBN, BCL10, IL12B, IL12A, RNF168, KDELR1, IGHV3-74, IGHV6-1, IGHV3-64, MSH2, KDM5D, IL18RAP, NDFIP1, IL4R, CD80, IGLV1-44, IGLC3, IGHA1, FZD5, IL18R1, C1QA, IGHG1, HPRT1, IGLV6-57, DLG1, CD226, C5, IL31RA, IGHV4-34, IL33, PKN1, C7, CLCF1, TNFSF13B, IGHV3-20, IGHV1-24, IGHV2-5, PRKCQ, RFTN1, CLU, IGHV3-23, TFRC | 80 |
| 32 | GO:0006959 | humoral immune response | CD83, IGLL1, IGHV3-33, CXCL13, ROMO1, IGHG4, IGLC6, IGLV2-23, CR1, IGHV4-28, IGHV4-39, IFNA5, C8G, IGHV5-51, IGHV3-7, DEFB127, DEFA3, KRT1, IGLV3-25, EXO1, SPON2, CHGA, CFHR4, IGHV1-45, IGHE, IFNB1, TRBC2, DEFB1, CFHR5, DEFA4, PRTN3, IGHV3-48, DEFB126, IGLV2-8, IGHG2, IGHD, IGLV2-11, MUC7, AZU1, IGHV3-43, IGHV1-58, DCD, APCS, SH2D1A, PLA2G6, C1QBP, IGLV3-1, IFNA14, KLK3, CFHR2, IGHV2-70, IGHM, FCGR2B, ITLN1, DEFB4A, C6, BPI, IL7, C4BPB, PGLYRP2, BPIFA1, IGLV1-51, HTN1, ST6GAL1, CPB2, IGHV3-49, SCARA3, CRP, IFNA6, MNX1, IFNA4, IFNA10, DEFA5, IGHV4-61, IFNA13, BPIFA2, IGHV4-59, CFHR1, C4BPA, SFTPD, FCN1, IFNE, HTN3, DEFA6, IGHV3-30, GPR183, IL6, C1QC, PPP2R3C, IGLC1, PAX5, YTHDF2, JCHAIN, HRG, IFNA16, IGHV1-3, IGHV4-4, IGLV7-43, IFNA2, IGLV3-27, C1QB, IGHV2-26, RPL39, IGLV3-19, FAM3A, KLK5, DEFA1, CR2, BPIFB2, DEFB118, IGHV3-15, IGLV1-40, CD28, C9, IFNA1, IGHV3-21, CFH, ACOD1, RGCC, IGHV3-53, CFD, CPN2, FCN2, PRSS3, IGHG3, FGA, LCN2, IGLC2, CD46, IGHA2, MASP2, TFE3, IGHV3-11, SUSD4, EBI3, ZP4, IGLC7, CD55, IGHV3-66, IGHV3-73, NOTCH1, DEFB103B, BCL2, DEFA1B, IFNA17, MEF2C, IGHV3-13, LEAP2, IGHV1-18, PTPRC, COL20A1, IFNA7, GATA3, MASP1, IGLV2-14, DEFB103A, IGLL5, TRAF3IP2, IGHV1OR15-1, KLK7, IGLV1-47, FCN3, DMBT1, IGHV3-72, IFNA21, A2M, IGLV3-21, IGHV1-69, CCR6, RNASE7, RARRES2, IGHV3-74, IGHV6-1, BST1, IGHV3-64, FGB, IFNK, COLEC10, VSIG4, IGLV1-44, RNASE6, IFNA8, IGLC3, IGHA1, PLA2G2A, PDCD1, C1QA, IGHG1, IGLV6-57, ELANE, C5, IGHV4-34, C7, RNASE3, IGHV3-20, IGHV1-24, IGHV2-5, BPIFB1, CD19, CLU, IFNW1, IGHV3-23 | 80 |
| 32 | GO:0070613 | regulation of protein processing | IGHV3-33, IGHG4, IGLC6, IGLV2-23, CR1, IGHV4-39, C8G, MELTF, IGHV3-7, IGLV3-25, CFHR4, PRSS37, CFHR5, IGHV3-48, IGLV2-8, IGHG2, IGLV2-11, USP17L2, NKD2, NOL3, C1QBP, IGLV3-1, CFHR2, IGHV2-70, C6, C4BPB, ACP4, IGLV1-51, CPB2, PRKACB, CNTN2, IGHV4-59, CFHR1, C4BPA, KLKB1, C1QC, IGHV3-30, IGLC1, SERPINF2, ANXA2P2, F12, ADAM8, IGLV7-43, IGLV3-27, C1QB, IGLV3-19, CR2, XIAP, IGLV1-40, C9, PLGRKT, CFH, TMEM59, IGHV3-53, GSN, CPN2, IGHG3, CD46, IGHV3-11, SUSD4, ENO1, IGLC7, CD55, TIMM17A, CARD8, IGHV3-13, IGLV2-14, CTSZ, IGHV1OR15-1, SERPINE2, IGLV1-47, ANGPTL8, A2M, IGLV3-21, IGHV1-69, MYH9, SERPINE1, NLRP7, BIRC7, IGLV1-44, BCL2L12, C1QA, IGHG1, IGLV6-57, SRC, C5, IGHV4-34, C7, IL1R2, IGHV2-5, CD19, CLU, TNP2, IGHV3-23, CCBE1, GLG1 | 80 |
| 32 | GO:0042742 | defense response to bacterium | SYT11, IGLL1, IGHV3-33, CXCL13, CARD9, ROMO1, IGHG4, IGLC6, IGHV4-28, IGHV4-39, IRF8, DEFA3, IGHV5-51, IGHV3-7, DEFB127, UNC13B, DEFB115, SPON2, CHGA, DEFB135, IGHV1-45, IGHE, TRBC2, DEFB1, DEFA4, IGHV3-48, DEFB126, IGHG2, IGHD, GSDMD, DEFB121, SIGLEC11, IGHV3-43, AZU1, TNFRSF14, HMGB2, IGHV1-58, DCD, DEFB116, PLA2G6, KLK3, TLR2, IGHV2-70, IGHM, DEFB136, DEFB4A, BPI, ISG15, TIRAP, PGLYRP2, DEFB132, C10orf99, BPIFA1, DEFB125, SPN, DEFB106B, HTN1, LYZL2, DEFB131A, IGHV3-49, CRP, PYCARD, FCER1G, DEFA5, IGHV4-61, BPIFA2, IGHV4-59, KLRK1, CD36, EPX, SFTPD, IFNE, DEFB104A, DEFB124, HTN3, DEFA6, IGHV3-30, IL6, NLRP1, RAB14, MR1, MAVS, IGLC1, JCHAIN, DEFB104B, DEFB108A, DEFB119, IGHV1-3, FOXP1, LBP, IGHV4-4, MPO, NOD1, RIPK2, DEFB128, IGHV2-26, NLRP3, RPL39, DEFB106A, DEFA1, KLK5, DEFB105A, DEFB134, DEFB118, IGHV3-15, LYZL1, IGHV3-21, ANXA3, IGHV3-53, FGR, IL23R, FCN2, RNASE8, IGHG3, FGA, IGLC2, IGHA2, SPAG11A, IL10, IGHV3-11, DEFB129, NR1H4, IGLC7, LYPD8, IGHV3-66, IGHV3-73, TLR3, SELP, DEFB103B, DEFA1B, CYBA, LYST, DEFB130A, DEFB109B, IGHV3-13, LEAP2, IGHV1-18, DEFB123, PRB3, F2, DEFB103A, HP, OPTN, IGLL5, IGHV1OR15-1, DEFB130B, IL22RA1, KLK7, DMBT1, IGHV3-72, LPO, SIGLEC16, PLAC8, IGHV1-69, RNASE7, ADGRB1, IL12B, IL12A, RARRES2, DEFB105B, STATH, IGHV3-74, IGHV6-1, IGHV3-64, FGB, SERPINE1, VGF, KLRC4-KLRK1, RNASE6, TLR5, IGLC3, IGHA1, PLA2G2A, ACP5, SPAG11B, IGHG1, ELANE, IGHV4-34, TMF1, RNASE3, IGHV3-20, IGHV1-24, CEBPB, F2RL1, TBK1, IGHV2-5, LACRT, IGHV3-23, EPHA2, RAB1A | 80 |
| 32 | GO:0038095 | Fc-epsilon receptor signaling pathway | IGHV3-53, IGLV3-21, FCER1G, IGHV3-33, IGHV1-69, IGLC6, IGLV2-23, LAT, BCL10, IGHV4-59, FCER1A, PIK3CB, IGHV4-39, VAV2, IGHV3-7, PLCG2, LILRA4, MAPK3, IKBKB, IGLV3-25, GRAP2, NFATC2, IKBKG, PAK2, IGHV3-11, IGHV3-30, IGHE, IGLC7, IGLC1, IGHV3-48, MALT1, IGLV2-8, IGLV1-44, LCP2, IGLV2-11, PPP3R1, PDPK1, NFATC1, BTK, RELA, IGLV6-57, IGLV7-43, IGHV3-13, IGLV3-27, IGHV4-34, IGLV3-1, CARD11, IGLV3-19, IGHV2-70, MAPK1, TEC, GRB2, IGLV2-14, PPP3CB, IGHV2-5, RAC1, IGHV1OR15-1, NR4A3, IGLV1-47, IGLV1-40, PRKCQ, TAB1, MAPK9, LAT2, IGLV1-51, IGHV3-23, MAP3K1, MAPK10 | 80 |
| 32 | GO:0008037 | cell recognition | TCP1, ROBO4, IGLL1, IGHV3-33, IGHG4, IGLC6, PEAR1, IGHV4-28, IGHV4-39, IGHV5-51, IGHV3-7, EPHB3, OVGP1, EPHB2, CCT2, IGHV1-45, IGHE, PRSS37, TRBC2, IGHV3-48, IGHG2, IGHD, DOCK8, IGHV3-43, CNTNAP3, ZAN, IGHV1-58, IGHV2-70, IGHM, VSTM2L, PCDH12, TNN, NDN, PCSK4, IGHV3-49, IGHV4-61, CDK5R1, CNTN2, IGHV4-59, CD36, IGSF9, CSGALNACT1, CCT3, FCN1, CATSPER3, IGHV3-30, IGLC1, ADAM21, ATP8B3, IGHV1-3, FETUB, BSG, IGHV4-4, COLEC12, CLEC7A, IGHV2-26, MSN, ROBO3, IGHV3-15, CNTN4, IGHV3-21, VDAC2, IGHV3-53, SPA17, B4GALT1, ACR, DOCK2, FCN2, IGHG3, MEGF10, IGLC2, IGHA2, SEMA3A, IGHV3-11, ZP4, DSCAM, ALDOA, IGLC7, CLGN, IGHV3-66, IGHV3-73, ADAM2, SEMA5A, CASP3, SPAM1, ZPBP, IGHV3-13, LGALS3, IGHV1-18, EPHA4, GAP43, IGLL5, IGHV1OR15-1, FCN3, IGHV3-72, CCT5, CNTNAP2, NEXN, PCDHA7, AMIGO1, IGHV1-69, PAEP, KCNU1, ADGRB1, CATSPER1, EFNB3, IGHV3-74, IGHV6-1, IGHV3-64, PCDHB6, NRP1, CCT4, MFGE8, IGLC3, IGHA1, NRCAM, IGHG1, DLG1, CD226, ST6GALNAC6, IGHV4-34, LAMA5, EMB, IGHV3-20, IGHV1-24, CNTN6, IGHV2-5, SPESP1, NPTN, IGHV3-23, IZUMO1, UBAP2L | 80 |

| Cluster | Term | Name | HPOs\_in\_clusters |
| --- | --- | --- | --- |
| 32 | OMIM:601390 | VAN MALDERGEM SYNDROME 1; VMLDS1 | HP:0000581, HP:0000347, HP:0000316, HP:0000431 |
| 32 | OMIM:613610 | CRANIOECTODERMAL DYSPLASIA 2; CED2 | HP:0000581, HP:0000347, HP:0000316, HP:0000431 |
| 32 | OMIM:615546 | VAN MALDERGEM SYNDROME 2; VMLDS2 | HP:0000581, HP:0000347, HP:0000316, HP:0000431 |

---


---


---

# Cluster 45

| Cluster | Term | Name |
| --- | --- | --- |
| 45 | HP:0000954 | Single transverse palmar crease |
| 45 | HP:0001773 | Short foot |
| 45 | HP:0004209 | Clinodactyly of the 5th finger |
| 45 | HP:0009803 | Short phalanx of finger |
| 45 | HP:0001831 | Short toe |

| Cluster | Term | Name | Genes | Percentage\_of\_nodes\_with\_funsys |
| --- | --- | --- | --- | --- |
| 45 | GO:0010615 | positive regulation of cardiac muscle adaptation | TRPC3, MIR214, MIR199A2, MIR199A1, MIR17, MIR20A | 80 |
| 45 | GO:0043500 | muscle adaptation | MIR133A2, MYOC, NPPA, CTDP1, MIR214, GLRX3, MIR499A, CAMTA2, GSN, MSTN, AKAP6, HDAC4, AGT, TNNI1, PDE5A, G6PD, MIR21, FOXO1, IL6ST, IL15, ACTA1, MIR17, MIR1-1, ATP2B4, MIR19B2, MIR17HG, GTF2IRD2, MIR19A, TRPC3, RPS6KB1, MIR19B1, GATA5, HAND2, MYOG, PARP1, ERRFI1, PPARGC1A, CAMK2B, BMP10, NOS3, MIR25, MTOR, SLC9A1, MIR199A2, MIR199A1, TNFRSF1B, JARID2, PRKCA, RGS2, MEF2A, MIR20A | 80 |
| 45 | GO:1903244 | positive regulation of cardiac muscle hypertrophy in response to stress | TRPC3, MIR214, MIR199A2, MIR199A1, MIR17, MIR20A | 80 |
| 45 | GO:0014742 | positive regulation of muscle hypertrophy | MIR19A, MIR214, MIR21, MIR199A2, TRPC3, MIR199A1, MTOR, MEF2A, SLC9A1, AKAP6, MIR19B1, PRKCA, HAND2, PARP1, BMP10, MIR17, PDE5A, MIR20A, MIR19B2 | 80 |
| 45 | GO:0014744 | positive regulation of muscle adaptation | MIR17HG, TRPC3, MIR214, MTOR, MIR199A2, MIR199A1, MYOG, MIR17, MIR20A | 80 |
| 45 | GO:0061051 | positive regulation of cell growth involved in cardiac muscle cell development | MIR19A, MTOR, MIR199A1, MIR199A2, AKAP6, MIR19B1, MIR19B2 | 80 |
| 45 | GO:1903242 | regulation of cardiac muscle hypertrophy in response to stress | TRPC3, MIR214, MIR199A2, MIR199A1, MIR25, ERRFI1, MIR17, ATP2B4, MIR20A, BMP10 | 80 |
| 45 | GO:0010612 | regulation of cardiac muscle adaptation | TRPC3, MIR214, MIR199A2, MIR199A1, MIR25, ERRFI1, MIR17, ATP2B4, MIR20A, BMP10 | 80 |
| 45 | GO:0010613 | positive regulation of cardiac muscle hypertrophy | MIR19A, MIR214, MIR21, MIR199A2, TRPC3, MIR199A1, MTOR, MEF2A, SLC9A1, AKAP6, MIR19B1, PRKCA, HAND2, PARP1, BMP10, MIR17, PDE5A, MIR20A, MIR19B2 | 80 |

| Cluster | Term | Name | HPOs\_in\_clusters |
| --- | --- | --- | --- |
| 45 | OMIM:261540 | PETERS-PLUS SYNDROME; PTRPLS | HP:0001831, HP:0000954, HP:0004209, HP:0001773 |

---


---


---

# Cluster 46

| Cluster | Term | Name |
| --- | --- | --- |
| 46 | HP:0008872 | Feeding difficulties in infancy |
| 46 | HP:0000316 | Hypertelorism |
| 46 | HP:0000347 | Micrognathia |
| 46 | HP:0000369 | Low-set ears |
| 46 | HP:0000431 | Wide nasal bridge |

| Cluster | Term | Name | Genes | Percentage\_of\_nodes\_with\_funsys |
| --- | --- | --- | --- | --- |
| 46 | GO:0015697 | quaternary ammonium group transport | SLC25A29, SLC25A47, SLC22A4, SLC25A48, SLC22A3, SLC22A16, SLC22A5, SLC22A1 | 80 |

| Cluster | Term | Name | HPOs\_in\_clusters |
| --- | --- | --- | --- |
| 46 | OMIM:115150 | CARDIOFACIOCUTANEOUS SYNDROME 1; CFC1 | HP:0000369, HP:0000347, HP:0008872, HP:0000316 |
| 46 | OMIM:180849 | RUBINSTEIN-TAYBI SYNDROME 1; RSTS1 | HP:0000369, HP:0000347, HP:0008872, HP:0000431 |
| 46 | OMIM:243605 | STROMME SYNDROME; STROMS | HP:0000347, HP:0000369, HP:0000316, HP:0000431 |
| 46 | OMIM:257300 | MOSAIC VARIEGATED ANEUPLOIDY SYNDROME 1; MVA1 | HP:0000369, HP:0000347, HP:0008872, HP:0000316 |
| 46 | OMIM:261515 | D-BIFUNCTIONAL PROTEIN DEFICIENCY | HP:0000369, HP:0000347, HP:0008872, HP:0000316 |
| 46 | OMIM:261540 | PETERS-PLUS SYNDROME; PTRPLS | HP:0000369, HP:0000347, HP:0008872, HP:0000316 |
| 46 | OMIM:268300 | ROBERTS SYNDROME; RBS | HP:0000347, HP:0000369, HP:0000316, HP:0000431 |
| 46 | OMIM:270400 | SMITH-LEMLI-OPITZ SYNDROME; SLOS | HP:0000347, HP:0000369, HP:0000316, HP:0000431 |
| 46 | OMIM:300215 | LISSENCEPHALY, X-LINKED, 2; LISX2 | HP:0000369, HP:0000347, HP:0008872, HP:0000431 |
| 46 | OMIM:300373 | OSTEOPATHIA STRIATA WITH CRANIAL SCLEROSIS; OSCS | HP:0000347, HP:0000369, HP:0000316, HP:0000431 |
| 46 | OMIM:309580 | MENTAL RETARDATION-HYPOTONIC FACIES SYNDROME, X-LINKED, 1; MRXHF1 | HP:0000347, HP:0000369, HP:0000316, HP:0000431 |
| 46 | OMIM:311900 | TARP SYNDROME; TARPS | HP:0000347, HP:0000369, HP:0000316, HP:0000431 |
| 46 | OMIM:605039 | BOHRING-OPITZ SYNDROME; BOPS | HP:0000347, HP:0000369, HP:0000316, HP:0000431 |
| 46 | OMIM:613610 | CRANIOECTODERMAL DYSPLASIA 2; CED2 | HP:0000347, HP:0000369, HP:0000316, HP:0000431 |
| 46 | OMIM:616897 | OSTEOCHONDRODYSPLASIA, COMPLEX LETHAL, SYMOENS-BARNES-GISTELINCK TYPE; OCLSBG | HP:0000347, HP:0000369, HP:0000316, HP:0000431 |
| 46 | OMIM:617062 | OKUR-CHUNG NEURODEVELOPMENTAL SYNDROME; OCNDS | HP:0000347, HP:0000369, HP:0000316, HP:0000431 |
| 46 | OMIM:617746 | SWEENEY-COX SYNDROME; SWCOS | HP:0000347, HP:0000369, HP:0000316, HP:0000431 |

---


---


---

# Cluster 47

| Cluster | Term | Name |
| --- | --- | --- |
| 47 | HP:0000343 | Long philtrum |
| 47 | HP:0001773 | Short foot |
| 47 | HP:0004209 | Clinodactyly of the 5th finger |
| 47 | HP:0009803 | Short phalanx of finger |
| 47 | HP:0004279 | Short palm |

| Cluster | Term | Name | Genes | Percentage\_of\_nodes\_with\_funsys |
| --- | --- | --- | --- | --- |
| 47 | GO:0010665 | regulation of cardiac muscle cell apoptotic process | MIR133A2, MIR195, EIF5A, MIR16-2, MIR16-1, MIR34A, GHRH, MIR21, BNIP3, MIR17, MIR19B2, PDPK1, MIR19A, HSF1, MIR19B1, HAND2, MIR199A2, MIR199A1, POU4F2, JAK2, MIR20A | 80 |
| 47 | GO:0010615 | positive regulation of cardiac muscle adaptation | TRPC3, MIR214, MIR199A2, MIR199A1, MIR17, MIR20A | 80 |
| 47 | GO:1903244 | positive regulation of cardiac muscle hypertrophy in response to stress | TRPC3, MIR214, MIR199A2, MIR199A1, MIR17, MIR20A | 80 |
| 47 | GO:0014742 | positive regulation of muscle hypertrophy | MIR19A, MIR214, MIR21, MIR199A2, TRPC3, MIR199A1, MTOR, MEF2A, SLC9A1, AKAP6, MIR19B1, PRKCA, HAND2, PARP1, BMP10, MIR17, PDE5A, MIR20A, MIR19B2 | 80 |
| 47 | GO:1903242 | regulation of cardiac muscle hypertrophy in response to stress | TRPC3, MIR214, MIR199A2, MIR199A1, MIR25, ERRFI1, MIR17, ATP2B4, MIR20A, BMP10 | 80 |
| 47 | GO:0010662 | regulation of striated muscle cell apoptotic process | MIR133A2, MIR195, EIF5A, BMP7, MIR16-2, MIR16-1, MIR34A, GHRH, MIR21, BNIP3, MIR17, MIR19B2, PDPK1, MIR19A, HSF1, MIR19B1, HAND2, MIR199A2, MIR199A1, POU4F2, JAK2, MIR20A | 80 |
| 47 | GO:0010612 | regulation of cardiac muscle adaptation | TRPC3, MIR214, MIR199A2, MIR199A1, MIR25, ERRFI1, MIR17, ATP2B4, MIR20A, BMP10 | 80 |
| 47 | GO:0010613 | positive regulation of cardiac muscle hypertrophy | MIR19A, MIR214, MIR21, MIR199A2, TRPC3, MIR199A1, MTOR, MEF2A, SLC9A1, AKAP6, MIR19B1, PRKCA, HAND2, PARP1, BMP10, MIR17, PDE5A, MIR20A, MIR19B2 | 80 |

| Cluster | Term | Name | HPOs\_in\_clusters |
| --- | --- | --- | --- |
| 47 | OMIM:102370 | ACROMICRIC DYSPLASIA; ACMICD | HP:0004279, HP:0009803, HP:0000343, HP:0001773 |
| 47 | OMIM:166250 | OSTEOGLOPHONIC DYSPLASIA; OGD | HP:0004279, HP:0009803, HP:0000343, HP:0001773 |
| 47 | OMIM:170390 | ANDERSEN CARDIODYSRHYTHMIC PERIODIC PARALYSIS | HP:0004279, HP:0009803, HP:0004209, HP:0001773 |
| 47 | OMIM:190351 | TRICHORHINOPHALANGEAL SYNDROME, TYPE III; TRPS3 | HP:0004279, HP:0009803, HP:0000343, HP:0001773 |
| 47 | OMIM:228520 | FIBROCHONDROGENESIS 1; FBCG1 | HP:0004279, HP:0004209, HP:0000343, HP:0001773 |
| 47 | OMIM:261540 | PETERS-PLUS SYNDROME; PTRPLS | HP:0004279, HP:0004209, HP:0000343, HP:0001773 |

---


---


---

# Cluster 55

| Cluster | Term | Name |
| --- | --- | --- |
| 55 | HP:0000286 | Epicanthus |
| 55 | HP:0000316 | Hypertelorism |
| 55 | HP:0000431 | Wide nasal bridge |
| 55 | HP:0000582 | Upslanted palpebral fissure |
| 55 | HP:0000347 | Micrognathia |

| Cluster | Term | Name | Genes | Percentage\_of\_nodes\_with\_funsys |
| --- | --- | --- | --- | --- |
| 55 | GO:0002526 | acute inflammatory response | SERPINC1, IGHV3-33, PARK7, IGHG4, IGLC6, IGLV2-23, CR1, TNFSF11, IGHV4-39, C8G, IGHV3-7, IGLV3-25, CFHR4, MIR92A2, TNFSF4, GSTP1, CFHR5, IGHV3-48, IGLV2-8, IGHG2, IGLV2-11, PIK3CG, EIF2AK1, APCS, C1QBP, IGLV3-1, CFHR2, OSMR, IGHV2-70, FCGR2B, C6, TAC1, C4BPB, VCAM1, PPARG, IGLV1-51, OPRM1, CPB2, CRP, FCER1G, IGHV4-59, CFHR1, NUPR1, C4BPA, KLKB1, TNFRSF11A, IGHV3-30, C1QC, IL6, IL6ST, IGLC1, LBP, SERPINF2, F12, ADAM8, IGLV7-43, IGLV3-27, RHBDD3, C1QB, ADORA1, IGLV3-19, NLRP3, NPFF, CR2, IGLV1-40, C9, CFH, IL20RB, IGHV3-53, SELENOS, PTGES, F8, B4GALT1, PLSCR1, CPN2, MIR92A1, CTNNBIP1, IGHG3, TFR2, CD46, IGHV3-11, SUSD4, UGT1A1, IGLC7, CD55, OSM, CREB3L3, DNASE1, BTK, ADCYAP1, FN1, IGHV3-13, GATA3, IGLV2-14, HP, IGHV1OR15-1, AHSG, IGLV1-47, TRPV1, DNASE1L3, A2M, IGLV3-21, ASS1, IGHV1-69, EPO, EDNRB, ASH1L, PTGS2, PTGER3, IGLV1-44, C1QA, IGHG1, IGLV6-57, ELANE, C5, IL31RA, APOA2, IGHV4-34, HPR, C7, OGG1, CEBPB, APOL2, IGHV2-5, CD19, CLU, IGHV3-23 | 80 |

| Cluster | Term | Name | HPOs\_in\_clusters |
| --- | --- | --- | --- |
| 55 | OMIM:309580 | MENTAL RETARDATION-HYPOTONIC FACIES SYNDROME, X-LINKED, 1; MRXHF1 | HP:0000431, HP:0000286, HP:0000347, HP:0000582, HP:0000316 |
| 55 | OMIM:613610 | CRANIOECTODERMAL DYSPLASIA 2; CED2 | HP:0000431, HP:0000286, HP:0000347, HP:0000582, HP:0000316 |
| 55 | OMIM:145410 | OPITZ GBBB SYNDROME, TYPE II; GBBB2 | HP:0000316, HP:0000347, HP:0000286, HP:0000431 |
| 55 | OMIM:164280 | FEINGOLD SYNDROME 1; FGLDS1 | HP:0000347, HP:0000582, HP:0000286, HP:0000431 |
| 55 | OMIM:194190 | WOLF-HIRSCHHORN SYNDROME; WHS | HP:0000316, HP:0000347, HP:0000286, HP:0000431 |
| 55 | OMIM:211750 | C SYNDROME | HP:0000347, HP:0000582, HP:0000286, HP:0000431 |
| 55 | OMIM:213980 | CRANIOFACIAL DYSMORPHISM, SKELETAL ANOMALIES, AND MENTAL RETARDATION SYNDROME; CFSMR | HP:0000316, HP:0000347, HP:0000286, HP:0000582 |
| 55 | OMIM:214100 | PEROXISOME BIOGENESIS DISORDER 1A (ZELLWEGER); PBD1A | HP:0000316, HP:0000347, HP:0000286, HP:0000582 |
| 55 | OMIM:214110 | PEROXISOME BIOGENESIS DISORDER 2A (ZELLWEGER); PBD2A | HP:0000316, HP:0000347, HP:0000286, HP:0000582 |
| 55 | OMIM:257300 | MOSAIC VARIEGATED ANEUPLOIDY SYNDROME 1; MVA1 | HP:0000316, HP:0000347, HP:0000286, HP:0000582 |
| 55 | OMIM:261515 | D-BIFUNCTIONAL PROTEIN DEFICIENCY | HP:0000316, HP:0000347, HP:0000286, HP:0000582 |
| 55 | OMIM:270400 | SMITH-LEMLI-OPITZ SYNDROME; SLOS | HP:0000316, HP:0000347, HP:0000286, HP:0000431 |
| 55 | OMIM:300373 | OSTEOPATHIA STRIATA WITH CRANIAL SCLEROSIS; OSCS | HP:0000316, HP:0000347, HP:0000286, HP:0000431 |
| 55 | OMIM:300749 | MENTAL RETARDATION AND MICROCEPHALY WITH PONTINE AND CEREBELLAR HYPOPLASIA; MICPCH | HP:0000316, HP:0000347, HP:0000286, HP:0000431 |
| 55 | OMIM:309500 | RENPENNING SYNDROME 1; RENS1 | HP:0000347, HP:0000582, HP:0000286, HP:0000431 |
| 55 | OMIM:601390 | VAN MALDERGEM SYNDROME 1; VMLDS1 | HP:0000316, HP:0000347, HP:0000286, HP:0000431 |
| 55 | OMIM:605039 | BOHRING-OPITZ SYNDROME; BOPS | HP:0000347, HP:0000582, HP:0000316, HP:0000431 |
| 55 | OMIM:611209 | CONGENITAL DISORDER OF GLYCOSYLATION, TYPE IIg; CDG2G | HP:0000347, HP:0000582, HP:0000316, HP:0000431 |
| 55 | OMIM:613406 | WITTEVEEN-KOLK SYNDROME; WITKOS | HP:0000316, HP:0000582, HP:0000286, HP:0000431 |
| 55 | OMIM:614976 | CARPENTER SYNDROME 2; CRPT2 | HP:0000316, HP:0000582, HP:0000286, HP:0000431 |
| 55 | OMIM:615546 | VAN MALDERGEM SYNDROME 2; VMLDS2 | HP:0000316, HP:0000347, HP:0000286, HP:0000431 |
| 55 | OMIM:617062 | OKUR-CHUNG NEURODEVELOPMENTAL SYNDROME; OCNDS | HP:0000316, HP:0000347, HP:0000286, HP:0000431 |
| 55 | OMIM:617360 | CONGENITAL HEART DEFECTS, DYSMORPHIC FACIAL FEATURES, AND INTELLECTUAL DEVELOPMENTAL DISORDER; CHDFIDD | HP:0000316, HP:0000582, HP:0000286, HP:0000431 |
| 55 | OMIM:617755 | NEURODEVELOPMENTAL DISORDER WITH DYSMORPHIC FACIES AND DISTAL LIMB ANOMALIES; NEDDFL | HP:0000316, HP:0000347, HP:0000286, HP:0000582 |
| 55 | OMIM:617991 | DEVELOPMENTAL DELAY, INTELLECTUAL DISABILITY, OBESITY, AND DYSMORPHISM; DIDOD | HP:0000316, HP:0000347, HP:0000286, HP:0000582 |

---


---


---

# Cluster 65

| Cluster | Term | Name |
| --- | --- | --- |
| 65 | HP:0000219 | Thin upper lip vermilion |
| 65 | HP:0004209 | Clinodactyly of the 5th finger |
| 65 | HP:0009803 | Short phalanx of finger |
| 65 | HP:0000954 | Single transverse palmar crease |
| 65 | HP:0001773 | Short foot |

| Cluster | Term | Name | Genes | Percentage\_of\_nodes\_with\_funsys |
| --- | --- | --- | --- | --- |
| 65 | GO:0010660 | regulation of muscle cell apoptotic process | MIR133A2, RBM10, MIR195, PTK2B, MIR106B, EIF5A, BMP7, MIR92A1, MIR16-1, MIR16-2, IL12A, MIR34A, GHRH, HMOX1, MIR21, MIR92A2, MYOCD, ARRB2, BNIP3, MIR17, MIR1-1, MIR19B2, PDPK1, MIR19A, HSF1, DIPK2A, MIR19B1, HAND2, ALOX12, APOPT1, MIR199A2, MIR199A1, LRP6, POU4F2, ATF4, MFN2, JAK2, MIR20A, CDKN2A | 80 |
| 65 | GO:0010659 | cardiac muscle cell apoptotic process | MIR133A2, GNB1, MIR195, EIF5A, MIR16-2, MIR16-1, MIR34A, GHRH, MIR21, GNGT1, BNIP3, APAF1, MIR17, MIR19B2, PDPK1, MIR19A, HSF1, MIR19B1, HAND2, MIR199A2, MIR199A1, POU4F2, JAK2, MIR20A | 80 |
| 65 | GO:0010658 | striated muscle cell apoptotic process | MIR133A2, GNB1, MIR195, EIF5A, BMP7, MIR16-2, MIR16-1, MIR34A, GHRH, MIR21, GNGT1, BNIP3, APAF1, MIR17, MIR19B2, PDPK1, MIR19A, HSF1, MIR19B1, HAND2, MIR199A2, MIR199A1, POU4F2, JAK2, MIR20A | 80 |
| 65 | GO:0010657 | muscle cell apoptotic process | MIR133A2, GNB1, RBM10, MIR195, PTK2B, EIF5A, BMP7, MIR92A1, MIR16-2, MIR16-1, IL12A, MIR34A, GHRH, HMOX1, MIR21, MIR92A2, GNGT1, MYOCD, ARRB2, BNIP3, APAF1, MIR17, MIR1-1, MIR19B2, PDPK1, MIR19A, HSF1, DIPK2A, MIR19B1, HAND2, ALOX12, APOPT1, MIR199A2, MIR199A1, LRP6, POU4F2, ATF4, MFN2, JAK2, MIR20A, CDKN2A | 80 |

| Cluster | Term | Name | HPOs\_in\_clusters |
| --- | --- | --- | --- |
| 65 | OMIM:170390 | ANDERSEN CARDIODYSRHYTHMIC PERIODIC PARALYSIS | HP:0009803, HP:0001773, HP:0000219, HP:0004209 |
| 65 | OMIM:261540 | PETERS-PLUS SYNDROME; PTRPLS | HP:0001773, HP:0000954, HP:0000219, HP:0004209 |

---


---


---

# Cluster 9

| Cluster | Term | Name |
| --- | --- | --- |
| 9 | HP:0001250 | Seizures |
| 9 | HP:0002079 | Hypoplasia of the corpus callosum |
| 9 | HP:0001869 | Deep plantar creases |
| 9 | HP:0011400 | Abnormal CNS myelination |

| Cluster | Term | Name | Genes | Percentage\_of\_nodes\_with\_funsys |
| --- | --- | --- | --- | --- |
| 9 | GO:0006261 | DNA-dependent DNA replication | POLN, FBXO5, SLBP, NUGGC, WRN, FGFR1, SENP2, RFC4, BCL6, GINS4, POLB, ETAA1, DACH1, WRNIP1 | 75 |

| Cluster | Term | Name | HPOs\_in\_clusters |
| --- | --- | --- | --- |
| 9 | OMIM:605039 | BOHRING-OPITZ SYNDROME; BOPS | HP:0002079, HP:0001250, HP:0001869 |

---


---


---

# Cluster 15

| Cluster | Term | Name |
| --- | --- | --- |
| 15 | HP:0000343 | Long philtrum |
| 15 | HP:0000369 | Low-set ears |
| 15 | HP:0000426 | Prominent nasal bridge |
| 15 | HP:0000581 | Blepharophimosis |

| Cluster | Term | Name | Genes | Percentage\_of\_nodes\_with\_funsys |
| --- | --- | --- | --- | --- |
| 15 | GO:0001937 | negative regulation of endothelial cell proliferation | MIR129-1, MIR342, KRIT1, MIR22, PDCD10, NR2F2, MIR16-2, MIR16-1, MIR30B, GHRL, MIR497, MIR361, MIR494, MIR34A, MIR20B, MIR329-1, MIR503, MIR21, MIR424, TNMD, ATP5IF1, MIR98, MIR15B, MIR487B, STAT1, CAV1, MIR410, MIR329-2, MIR126, FLT1, MIR15A, CAV2, SYNJ2BP, MIR24-1, MIR495, RGCC | 75 |

| Cluster | Term | Name | HPOs\_in\_clusters |
| --- | --- | --- | --- |
| 15 | OMIM:139210 | MYHRE SYNDROME; MYHRS | HP:0000581, HP:0000426, HP:0000369 |
| 15 | OMIM:214150 | CEREBROOCULOFACIOSKELETAL SYNDROME 1; COFS1 | HP:0000581, HP:0000426, HP:0000343 |
| 15 | OMIM:248700 | MARDEN-WALKER SYNDROME; MWKS | HP:0000581, HP:0000369, HP:0000343 |
| 15 | OMIM:300166 | MICROPHTHALMIA, SYNDROMIC 2; MCOPS2 | HP:0000581, HP:0000426, HP:0000343 |
| 15 | OMIM:300215 | LISSENCEPHALY, X-LINKED, 2; LISX2 | HP:0000426, HP:0000369, HP:0000343 |
| 15 | OMIM:605130 | WIEDEMANN-STEINER SYNDROME; WDSTS | HP:0000581, HP:0000369, HP:0000343 |

---


---


---

# Cluster 18

| Cluster | Term | Name |
| --- | --- | --- |
| 18 | HP:0001249 | Intellectual disability |
| 18 | HP:0000494 | Downslanted palpebral fissures |
| 18 | HP:0003508 | Proportionate short stature |
| 18 | HP:0004209 | Clinodactyly of the 5th finger |

| Cluster | Term | Name | Genes | Percentage\_of\_nodes\_with\_funsys |
| --- | --- | --- | --- | --- |
| 18 | GO:0021872 | forebrain generation of neurons | SALL3, DRD1, SATB2, UQCRQ, TBR1, SEMA3A, PROX1, METTL14, GLI3, OGDH, DLX1, ERBB4, POU3F4, SOX1, BCL11B, ATP7A, DLX5, SHANK3, NRP2, LHX6, INHBA, DCT, PLXNA3, UBB, DLX2, SLC4A10, ATF5, UNCX, GBX2, LHX8, HES5, ASPM | 75 |

| Cluster | Term | Name | HPOs\_in\_clusters |
| --- | --- | --- | --- |
| 18 | OMIM:210600 | SECKEL SYNDROME 1; SCKL1 | HP:0001249, HP:0000494, HP:0003508, HP:0004209 |
| 18 | OMIM:115150 | CARDIOFACIOCUTANEOUS SYNDROME 1; CFC1 | HP:0001249, HP:0000494, HP:0004209 |
| 18 | OMIM:180849 | RUBINSTEIN-TAYBI SYNDROME 1; RSTS1 | HP:0001249, HP:0000494, HP:0004209 |
| 18 | OMIM:300504 | MOVED TO 300419 | HP:0001249, HP:0000494, HP:0004209 |
| 18 | OMIM:300990 | MIDFACE HYPOPLASIA, HEARING IMPAIRMENT, ELLIPTOCYTOSIS, AND NEPHROCALCINOSIS; MFHIEN | HP:0001249, HP:0000494, HP:0004209 |
| 18 | OMIM:605130 | WIEDEMANN-STEINER SYNDROME; WDSTS | HP:0001249, HP:0000494, HP:0004209 |
| 18 | OMIM:615637 | MENTAL RETARDATION, AUTOSOMAL RECESSIVE 41; MRT41 | HP:0001249, HP:0000494, HP:0004209 |
| 18 | OMIM:618067 | EPILEPTIC ENCEPHALOPATHY, EARLY INFANTILE, 66; EIEE66 | HP:0001249, HP:0000494, HP:0004209 |

---


---


---

# Cluster 22

| Cluster | Term | Name |
| --- | --- | --- |
| 22 | HP:0000639 | Nystagmus |
| 22 | HP:0001321 | Cerebellar hypoplasia |
| 22 | HP:0005484 | Postnatal microcephaly |
| 22 | HP:0100704 | Cerebral visual impairment |

| Cluster | Term | Name | Genes | Percentage\_of\_nodes\_with\_funsys |
| --- | --- | --- | --- | --- |
| 22 | GO:0019336 | phenol-containing compound catabolic process | MAOA, MAOB | 75 |
| 22 | GO:0042135 | neurotransmitter catabolic process | MAOB, GLDC, MAOA, PRIMA1, ABAT | 75 |

| Cluster | Term | Name | HPOs\_in\_clusters |
| --- | --- | --- | --- |
| 22 | OMIM:300868 | MULTIPLE CONGENITAL ANOMALIES-HYPOTONIA-SEIZURES SYNDROME 2; MCAHS2 | HP:0005484, HP:0100704, HP:0001321 |
| 22 | OMIM:608799 | CONGENITAL DISORDER OF GLYCOSYLATION, TYPE Ie; CDG1E | HP:0005484, HP:0100704, HP:0000639 |

---


---


---

# Cluster 25

| Cluster | Term | Name |
| --- | --- | --- |
| 25 | HP:0001629 | Ventricular septal defect |
| 25 | HP:0000085 | Horseshoe kidney |
| 25 | HP:0000612 | Iris coloboma |
| 25 | HP:0000998 | Hypertrichosis |

| Cluster | Term | Name | Genes | Percentage\_of\_nodes\_with\_funsys |
| --- | --- | --- | --- | --- |
| 25 | GO:0046503 | glycerolipid catabolic process | FABP12, GDPD3, FABP5, PNPLA3, PIK3CG, DAGLB, PNPLA8, FABP9, FABP4, PNPLA5, LPL | 75 |

| Cluster | Term | Name | HPOs\_in\_clusters |
| --- | --- | --- | --- |
| 25 | OMIM:607323 | DUANE-RADIAL RAY SYNDROME; DRRS | HP:0001629, HP:0000085, HP:0000612 |

---


---


---

# Cluster 29

| Cluster | Term | Name |
| --- | --- | --- |
| 29 | HP:0001629 | Ventricular septal defect |
| 29 | HP:0001642 | Pulmonic stenosis |
| 29 | HP:0001643 | Patent ductus arteriosus |
| 29 | HP:0001631 | Atrial septal defect |

| Cluster | Term | Name | Genes | Percentage\_of\_nodes\_with\_funsys |
| --- | --- | --- | --- | --- |
| 29 | GO:0031640 | killing of cells of other organism | DEFA1B, F2, DEFB4A, DEFA1, DEFA5, DEFA6, DEFB103A, AZU1, ROMO1, DEFA4, ELANE, DCD, C9, DEFA3, HRG, APOL1, DEFB103B | 75 |
| 29 | GO:0044364 | disruption of cells of other organism | DEFA1B, F2, DEFB4A, DEFA1, DEFA5, DEFA6, DEFB103A, AZU1, ROMO1, DEFA4, ELANE, DCD, C9, DEFA3, HRG, APOL1, DEFB103B | 75 |
| 29 | GO:0050829 | defense response to Gram-negative bacterium | DEFA5, ROMO1, RNASE7, RNASE8, ADGRB1, DEFA3, DEFB127, DEFB104A, CHGA, DEFA6, IL6, SERPINE1, MR1, DEFB1, DEFA4, CD160, DEFB126, DEFB104B, RNASE6, GSDMD, DEFB103B, SELP, DEFA1B, AZU1, LBP, TNFRSF14, ELANE, DEFB128, IGHM, RNASE3, PRB3, DEFB4A, DEFB106A, DEFA1, DEFB103A, F2, BPI, DEFB118, IL22RA1, DEFB132, LYZL1, DMBT1, DEFB106B, LYZL2 | 75 |
| 29 | GO:0002227 | innate immune response in mucosa | DEFA1B, DEFA1, DEFA5, DEFA6, BPIFB1, DEFB1, DEFA4, RPL39, DEFA3, RNASE3 | 75 |
| 29 | GO:0050830 | defense response to Gram-positive bacterium | DEFA5, FGR, CARD9, ROMO1, RNASE7, RNASE8, KLRK1, DEFA3, CD36, IL12A, DEFB104A, EPHA2, DEFA6, DEFB1, DEFA4, DEFB104B, KLRC4-KLRK1, RNASE6, GSDMD, DEFB103B, DEFA1B, PLA2G2A, ACP5, LBP, TNFRSF14, RIPK2, TLR2, RNASE3, DEFB4A, DEFB106A, DEFA1, DEFB103A, LYZL1, DMBT1, DEFB106B, LYZL2, CRP | 75 |
| 29 | GO:0002251 | organ or tissue specific immune response | DEFA1B, DEFA1, DEFA5, DEFA6, IL6, XCL1, BPIFB1, DEFB1, DEFA4, BPIFA1, PIGR, DEFA3, RAB17, RNASE3 | 75 |
| 29 | GO:0009620 | response to fungus | DEFA5, CARD9, CHIA, COTL1, RNASE7, RNASE8, DEFA3, SPON2, IL17RC, DEFA6, DEFA4, IL17RA, HRG, DEFA1B, MPO, ELANE, DCD, JAGN1, CRK, DEFB106A, DEFA1, PTX3, DEFB106B | 75 |
| 29 | GO:0051673 | membrane disruption in other organism | DEFA1B, DEFA1, DEFA5, DEFA6, DEFB118, RNASE7, DEFA4, DEFA3, GSDMD | 75 |
| 29 | GO:0002385 | mucosal immune response | DEFA1B, DEFA1, DEFA5, DEFA6, XCL1, BPIFB1, DEFB1, DEFA4, BPIFA1, PIGR, DEFA3, RAB17, RNASE3 | 75 |
| 29 | GO:0019731 | antibacterial humoral response | DEFA5, RNASE7, DEFA3, FGA, SFTPD, IGHA2, SPON2, DEFA6, FGB, DEFB1, DEFA4, JCHAIN, RNASE6, IGHA1, DEFA1B, ELANE, PLA2G6, KLK3, IGHM, RNASE3, KLK5, DEFA1, KLK7, BPIFA1, DMBT1 | 75 |
| 29 | GO:0050832 | defense response to fungus | DEFA5, COTL1, RNASE7, RNASE8, DEFA3, RARRES2, SPON2, IL17RC, HTN3, DEFA6, DEFA4, IL17RA, HRG, DEFA1B, MPO, ELANE, DCD, JAGN1, FAM3A, DEFB106A, DEFA1, DEFB106B, HTN1 | 75 |

| Cluster | Term | Name | HPOs\_in\_clusters |
| --- | --- | --- | --- |
| 29 | OMIM:163950 | NOONAN SYNDROME 1; NS1 | HP:0001629, HP:0001631, HP:0001642, HP:0001643 |
| 29 | OMIM:201000 | CARPENTER SYNDROME 1; CRPT1 | HP:0001629, HP:0001631, HP:0001642, HP:0001643 |
| 29 | OMIM:214800 | CHARGE SYNDROME | HP:0001629, HP:0001631, HP:0001642, HP:0001643 |
| 29 | OMIM:235730 | MOWAT-WILSON SYNDROME; MOWS | HP:0001629, HP:0001631, HP:0001642, HP:0001643 |
| 29 | OMIM:261540 | PETERS-PLUS SYNDROME; PTRPLS | HP:0001629, HP:0001631, HP:0001642, HP:0001643 |
| 29 | OMIM:300166 | MICROPHTHALMIA, SYNDROMIC 2; MCOPS2 | HP:0001629, HP:0001631, HP:0001642, HP:0001643 |
| 29 | OMIM:600001 | HEART DEFECTS, CONGENITAL, AND OTHER CONGENITAL ANOMALIES; HDCA | HP:0001629, HP:0001631, HP:0001642, HP:0001643 |
| 29 | OMIM:601186 | MICROPHTHALMIA, SYNDROMIC 9; MCOPS9 | HP:0001629, HP:0001631, HP:0001642, HP:0001643 |
| 29 | OMIM:609942 | NOONAN SYNDROME 3; NS3 | HP:0001629, HP:0001631, HP:0001642, HP:0001643 |
| 29 | OMIM:100300 | ADAMS-OLIVER SYNDROME 1; AOS1 | HP:0001629, HP:0001631, HP:0001642 |
| 29 | OMIM:117550 | SOTOS SYNDROME 1; SOTOS1 | HP:0001629, HP:0001631, HP:0001643 |
| 29 | OMIM:117650 | CEREBROCOSTOMANDIBULAR SYNDROME; CCMS | HP:0001629, HP:0001631, HP:0001643 |
| 29 | OMIM:121050 | CONTRACTURAL ARACHNODACTYLY, CONGENITAL; CCA | HP:0001629, HP:0001631, HP:0001643 |
| 29 | OMIM:135900 | COFFIN-SIRIS SYNDROME 1; CSS1 | HP:0001629, HP:0001631, HP:0001643 |
| 29 | OMIM:145410 | OPITZ GBBB SYNDROME, TYPE II; GBBB2 | HP:0001629, HP:0001631, HP:0001643 |
| 29 | OMIM:180849 | RUBINSTEIN-TAYBI SYNDROME 1; RSTS1 | HP:0001629, HP:0001631, HP:0001643 |
| 29 | OMIM:208530 | RIGHT ATRIAL ISOMERISM; RAI | HP:0001629, HP:0001631, HP:0001642 |
| 29 | OMIM:218040 | COSTELLO SYNDROME; CSTLO | HP:0001629, HP:0001631, HP:0001642 |
| 29 | OMIM:220210 | RITSCHER-SCHINZEL SYNDROME 1; RTSC1 | HP:0001629, HP:0001631, HP:0001642 |
| 29 | OMIM:257920 | 3MC SYNDROME 1; 3MC1 | HP:0001629, HP:0001631, HP:0001643 |
| 29 | OMIM:268300 | ROBERTS SYNDROME; RBS | HP:0001629, HP:0001631, HP:0001643 |
| 29 | OMIM:270400 | SMITH-LEMLI-OPITZ SYNDROME; SLOS | HP:0001629, HP:0001631, HP:0001643 |
| 29 | OMIM:300373 | OSTEOPATHIA STRIATA WITH CRANIAL SCLEROSIS; OSCS | HP:0001629, HP:0001631, HP:0001643 |
| 29 | OMIM:300963 | RITSCHER-SCHINZEL SYNDROME 2; RTSC2 | HP:0001629, HP:0001631, HP:0001643 |
| 29 | OMIM:306955 | HETEROTAXY, VISCERAL, 1, X-LINKED; HTX1 | HP:0001629, HP:0001631, HP:0001642 |
| 29 | OMIM:312870 | SIMPSON-GOLABI-BEHMEL SYNDROME, TYPE 1; SGBS1 | HP:0001629, HP:0001642, HP:0001643 |
| 29 | OMIM:607721 | NOONAN SYNDROME-LIKE DISORDER WITH LOOSE ANAGEN HAIR 1; NSLH1 | HP:0001629, HP:0001631, HP:0001642 |
| 29 | OMIM:608328 | WEILL-MARCHESANI SYNDROME 2; WMS2 | HP:0001629, HP:0001642, HP:0001643 |
| 29 | OMIM:610443 | KOOLEN-DE VRIES SYNDROME; KDVS | HP:0001629, HP:0001631, HP:0001642 |
| 29 | OMIM:612541 | NEUTROPENIA, SEVERE CONGENITAL, 4, AUTOSOMAL RECESSIVE; SCN4 | HP:0001631, HP:0001642, HP:0001643 |
| 29 | OMIM:613870 | HIRSCHSPRUNG DISEASE, CARDIAC DEFECTS, AND AUTONOMIC DYSFUNCTION; HCAD | HP:0001629, HP:0001631, HP:0001643 |
| 29 | OMIM:614262 | ARTHROGRYPOSIS, PERTHES DISEASE, AND UPWARD GAZE PALSY; APUG | HP:0001629, HP:0001631, HP:0001642 |
| 29 | OMIM:615355 | NOONAN SYNDROME 8; NS8 | HP:0001629, HP:0001631, HP:0001642 |
| 29 | OMIM:616268 | MENTAL RETARDATION, AUTOSOMAL DOMINANT 32; MRD32 | HP:0001629, HP:0001631, HP:0001643 |
| 29 | OMIM:617506 | NOONAN SYNDROME-LIKE DISORDER WITH LOOSE ANAGEN HAIR 2; NSLH2 | HP:0001629, HP:0001642, HP:0001643 |
| 29 | OMIM:618142 | MICROCEPHALY, FACIAL DYSMORPHISM, RENAL AGENESIS, AND AMBIGUOUS GENITALIA SYNDROME; MFRG | HP:0001629, HP:0001631, HP:0001643 |
| 29 | OMIM:618223 | VERTEBRAL ANOMALIES AND VARIABLE ENDOCRINE AND T-CELL DYSFUNCTION; VETD | HP:0001631, HP:0001642, HP:0001643 |
| 29 | OMIM:618280 | CARDIAC-UROGENITAL SYNDROME; CUGS | HP:0001629, HP:0001631, HP:0001643 |

---


---


---

# Cluster 35

| Cluster | Term | Name |
| --- | --- | --- |
| 35 | HP:0000028 | Cryptorchidism |
| 35 | HP:0000047 | Hypospadias |
| 35 | HP:0001631 | Atrial septal defect |
| 35 | HP:0001643 | Patent ductus arteriosus |

| Cluster | Term | Name | Genes | Percentage\_of\_nodes\_with\_funsys |
| --- | --- | --- | --- | --- |
| 35 | GO:0000098 | sulfur amino acid catabolic process | TST, MPST, MTRR, AGXT, TXN2, MAT1A | 75 |
| 35 | GO:0000096 | sulfur amino acid metabolic process | TST, NFS1, GGT5, MPST, MTRR, AGXT, GGT3P, MSRA, TXN2, AHCY, GGT7 | 75 |

| Cluster | Term | Name | HPOs\_in\_clusters |
| --- | --- | --- | --- |
| 35 | OMIM:135900 | COFFIN-SIRIS SYNDROME 1; CSS1 | HP:0000028, HP:0000047, HP:0001643, HP:0001631 |
| 35 | OMIM:145410 | OPITZ GBBB SYNDROME, TYPE II; GBBB2 | HP:0000028, HP:0000047, HP:0001643, HP:0001631 |
| 35 | OMIM:180849 | RUBINSTEIN-TAYBI SYNDROME 1; RSTS1 | HP:0000028, HP:0000047, HP:0001643, HP:0001631 |
| 35 | OMIM:261540 | PETERS-PLUS SYNDROME; PTRPLS | HP:0000028, HP:0000047, HP:0001643, HP:0001631 |
| 35 | OMIM:268300 | ROBERTS SYNDROME; RBS | HP:0000028, HP:0000047, HP:0001643, HP:0001631 |
| 35 | OMIM:270400 | SMITH-LEMLI-OPITZ SYNDROME; SLOS | HP:0000028, HP:0000047, HP:0001643, HP:0001631 |
| 35 | OMIM:102500 | HAJDU-CHENEY SYNDROME; HJCYS | HP:0000047, HP:0001643, HP:0000028 |
| 35 | OMIM:163950 | NOONAN SYNDROME 1; NS1 | HP:0001631, HP:0001643, HP:0000028 |
| 35 | OMIM:201000 | CARPENTER SYNDROME 1; CRPT1 | HP:0001631, HP:0001643, HP:0000028 |
| 35 | OMIM:206900 | MICROPHTHALMIA, SYNDROMIC 3; MCOPS3 | HP:0000047, HP:0001643, HP:0000028 |
| 35 | OMIM:214100 | PEROXISOME BIOGENESIS DISORDER 1A (ZELLWEGER); PBD1A | HP:0000047, HP:0001643, HP:0000028 |
| 35 | OMIM:214800 | CHARGE SYNDROME | HP:0001631, HP:0001643, HP:0000028 |
| 35 | OMIM:225500 | ELLIS-VAN CREVELD SYNDROME; EVC | HP:0000047, HP:0001631, HP:0000028 |
| 35 | OMIM:243800 | JOHANSON-BLIZZARD SYNDROME; JBS | HP:0000047, HP:0001631, HP:0000028 |
| 35 | OMIM:275210 | RESTRICTIVE DERMOPATHY, LETHAL | HP:0000047, HP:0001643, HP:0001631 |
| 35 | OMIM:300166 | MICROPHTHALMIA, SYNDROMIC 2; MCOPS2 | HP:0001631, HP:0001643, HP:0000028 |
| 35 | OMIM:300963 | RITSCHER-SCHINZEL SYNDROME 2; RTSC2 | HP:0001631, HP:0001643, HP:0000028 |
| 35 | OMIM:312870 | SIMPSON-GOLABI-BEHMEL SYNDROME, TYPE 1; SGBS1 | HP:0000047, HP:0001643, HP:0000028 |
| 35 | OMIM:601186 | MICROPHTHALMIA, SYNDROMIC 9; MCOPS9 | HP:0001631, HP:0001643, HP:0000028 |
| 35 | OMIM:607143 | CONGENITAL DISORDER OF GLYCOSYLATION, TYPE Ig; CDG1G | HP:0000047, HP:0001643, HP:0000028 |
| 35 | OMIM:612289 | FONTAINE PROGEROID SYNDROME; FPS | HP:0001631, HP:0001643, HP:0000028 |
| 35 | OMIM:612541 | NEUTROPENIA, SEVERE CONGENITAL, 4, AUTOSOMAL RECESSIVE; SCN4 | HP:0001631, HP:0001643, HP:0000028 |
| 35 | OMIM:617053 | MIRAGE SYNDROME; MIRAGE | HP:0000047, HP:0001643, HP:0000028 |
| 35 | OMIM:617063 | MEIER-GORLIN SYNDROME 7; MGORS7 | HP:0000047, HP:0001631, HP:0000028 |
| 35 | OMIM:617516 | STANKIEWICZ-ISIDOR SYNDROME; STISS | HP:0000047, HP:0001643, HP:0000028 |
| 35 | OMIM:618109 | MENTAL RETARDATION, AUTOSOMAL RECESSIVE 65; MRT65 | HP:0000047, HP:0001631, HP:0000028 |
| 35 | OMIM:618280 | CARDIAC-UROGENITAL SYNDROME; CUGS | HP:0001631, HP:0001643, HP:0000028 |
| 35 | OMIM:618316 | INTELLECTUAL DEVELOPMENTAL DISORDER WITH CARDIAC DEFECTS AND DYSMORPHIC FACIES; IDDCDF | HP:0000047, HP:0001643, HP:0000028 |

---


---


---

# Cluster 36

| Cluster | Term | Name |
| --- | --- | --- |
| 36 | HP:0000463 | Anteverted nares |
| 36 | HP:0000486 | Strabismus |
| 36 | HP:0002007 | Frontal bossing |
| 36 | HP:0000347 | Micrognathia |

| Cluster | Term | Name | Genes | Percentage\_of\_nodes\_with\_funsys |
| --- | --- | --- | --- | --- |
| 36 | GO:0000209 | protein polyubiquitination | MARCH5, UBE2L5, SPSB1, UBE4B, FBXO2, FBXO44, FBXO6, FBXL7, UBE3C, ANKIB1, MARCH6, RNF4, UBE2E3, TMEM129, FBXL21, KLHL3, CDC23, UBE2Q2, FBXO22, TRAF2, FBXW5, ANAPC2, SKP1, UBE2B, NHLRC1, RNF144B, FBXL5, FBXL14, MYLIP, KLHL42, GPS2, HERC6, HERC5, UBE2J2, MIB2, RNF114, UBE2V1, KLHL20, RC3H1, C10orf90, UBE2L3, PPIL2, FBXO11, ANAPC1, UBE2T, DTL, HECW1, UBE2D4, RBBP6, MKRN1, FBXL13, PRKN, FBXW10, PARP10, SHARPIN, RBCK1, UBB, DZIP3, FBXO40, DTX3L, FBXL18, RNF216, CDC16, TPP2, FBXW9, DYSF, FBXW2, RC3H2 | 75 |

| Cluster | Term | Name | HPOs\_in\_clusters |
| --- | --- | --- | --- |
| 36 | OMIM:616975 | NEURODEVELOPMENTAL DISORDER WITH OR WITHOUT ANOMALIES OF THE BRAIN, EYE, OR HEART; NEDBEH | HP:0002007, HP:0000463, HP:0000347, HP:0000486 |
| 36 | OMIM:115150 | CARDIOFACIOCUTANEOUS SYNDROME 1; CFC1 | HP:0000463, HP:0000347, HP:0000486 |
| 36 | OMIM:122470 | CORNELIA DE LANGE SYNDROME 1; CDLS1 | HP:0000463, HP:0000347, HP:0000486 |
| 36 | OMIM:145410 | OPITZ GBBB SYNDROME, TYPE II; GBBB2 | HP:0002007, HP:0000347, HP:0000486 |
| 36 | OMIM:180700 | ROBINOW SYNDROME, AUTOSOMAL DOMINANT 1; DRS1 | HP:0002007, HP:0000463, HP:0000347 |
| 36 | OMIM:211750 | C SYNDROME | HP:0000463, HP:0000347, HP:0000486 |
| 36 | OMIM:218040 | COSTELLO SYNDROME; CSTLO | HP:0000463, HP:0000347, HP:0000486 |
| 36 | OMIM:219200 | CUTIS LAXA, AUTOSOMAL RECESSIVE, TYPE IIA; ARCL2A | HP:0002007, HP:0000463, HP:0000486 |
| 36 | OMIM:224690 | MEIER-GORLIN SYNDROME 1; MGORS1 | HP:0002007, HP:0000347, HP:0000486 |
| 36 | OMIM:230740 | GAPO SYNDROME; GAPOS | HP:0002007, HP:0000347, HP:0000486 |
| 36 | OMIM:248700 | MARDEN-WALKER SYNDROME; MWKS | HP:0000463, HP:0000347, HP:0000486 |
| 36 | OMIM:256600 | NEURODEGENERATION WITH BRAIN IRON ACCUMULATION 2A; NBIA2A | HP:0002007, HP:0000347, HP:0000486 |
| 36 | OMIM:261515 | D-BIFUNCTIONAL PROTEIN DEFICIENCY | HP:0002007, HP:0000347, HP:0000486 |
| 36 | OMIM:268310 | ROBINOW SYNDROME, AUTOSOMAL RECESSIVE 1; RRS1 | HP:0002007, HP:0000463, HP:0000347 |
| 36 | OMIM:270400 | SMITH-LEMLI-OPITZ SYNDROME; SLOS | HP:0000463, HP:0000347, HP:0000486 |
| 36 | OMIM:305450 | OPITZ-KAVEGGIA SYNDROME; OKS | HP:0002007, HP:0000347, HP:0000486 |
| 36 | OMIM:608013 | GAUCHER DISEASE, PERINATAL LETHAL | HP:0000463, HP:0000347, HP:0000486 |
| 36 | OMIM:614080 | MULTIPLE CONGENITAL ANOMALIES-HYPOTONIA-SEIZURES SYNDROME 1; MCAHS1 | HP:0002007, HP:0000463, HP:0000347 |
| 36 | OMIM:615761 | MENTAL RETARDATION, AUTOSOMAL DOMINANT 23; MRD23 | HP:0000463, HP:0000347, HP:0000486 |
| 36 | OMIM:617616 | SKRABAN-DEARDORFF SYNDROME; SKDEAS | HP:0000463, HP:0000347, HP:0000486 |
| 36 | OMIM:617991 | DEVELOPMENTAL DELAY, INTELLECTUAL DISABILITY, OBESITY, AND DYSMORPHISM; DIDOD | HP:0000463, HP:0000347, HP:0000486 |

---


---


---

# Cluster 52

| Cluster | Term | Name |
| --- | --- | --- |
| 52 | HP:0001156 | Brachydactyly |
| 52 | HP:0011304 | Broad thumb |
| 52 | HP:0001773 | Short foot |
| 52 | HP:0009803 | Short phalanx of finger |

| Cluster | Term | Name | Genes | Percentage\_of\_nodes\_with\_funsys |
| --- | --- | --- | --- | --- |
| 52 | GO:0010665 | regulation of cardiac muscle cell apoptotic process | MIR133A2, MIR195, EIF5A, MIR16-2, MIR16-1, MIR34A, GHRH, MIR21, BNIP3, MIR17, MIR19B2, PDPK1, MIR19A, HSF1, MIR19B1, HAND2, MIR199A2, MIR199A1, POU4F2, JAK2, MIR20A | 75 |
| 52 | GO:0003300 | cardiac muscle hypertrophy | MIR133A2, HDAC2, GLRX3, NPPA, MIR214, CAMTA2, MIR195, AKAP6, HDAC4, RYR2, PDE5A, G6PD, MIR21, MIR17, MIR1-1, ATP2B4, MIR19B2, HTR2B, MIR19A, TRPC3, MIR19B1, GATA5, PARP1, ERRFI1, BMP10, MIR25, MTOR, SLC9A1, MIR199A2, TNFRSF1B, AGTR2, RGS2, MEF2A, MIR20A | 75 |
| 52 | GO:0010615 | positive regulation of cardiac muscle adaptation | TRPC3, MIR214, MIR199A2, MIR199A1, MIR17, MIR20A | 75 |
| 52 | GO:0043500 | muscle adaptation | MIR133A2, MYOC, NPPA, CTDP1, MIR214, GLRX3, MIR499A, CAMTA2, GSN, MSTN, AKAP6, HDAC4, AGT, TNNI1, PDE5A, G6PD, MIR21, FOXO1, IL6ST, IL15, ACTA1, MIR17, MIR1-1, ATP2B4, MIR19B2, MIR17HG, GTF2IRD2, MIR19A, TRPC3, RPS6KB1, MIR19B1, GATA5, HAND2, MYOG, PARP1, ERRFI1, PPARGC1A, CAMK2B, BMP10, NOS3, MIR25, MTOR, SLC9A1, MIR199A2, MIR199A1, TNFRSF1B, JARID2, PRKCA, RGS2, MEF2A, MIR20A | 75 |
| 52 | GO:1903244 | positive regulation of cardiac muscle hypertrophy in response to stress | TRPC3, MIR214, MIR199A2, MIR199A1, MIR17, MIR20A | 75 |
| 52 | GO:0014897 | striated muscle hypertrophy | MYOC, NPPA, GLRX3, MIR214, HDAC2, CAMTA2, MIR195, AKAP6, SORBS2, HDAC4, RYR2, PDE5A, G6PD, MIR21, MIR15B, MIR17, ATP2B4, MIR19B2, HTR2B, MIR19A, TRPC3, MIR19B1, HAND2, PARP1, ERRFI1, BMP10, MIR25, MTOR, SLC9A1, MIR199A2, MIR199A1, TNFRSF1B, AGTR2, RGS2, MEF2A, MIR20A | 75 |
| 52 | GO:2000181 | negative regulation of blood vessel morphogenesis | MECP2, PDCD10, MIR16-2, MIR23B, TEK, MIR497, AGT, DAB2IP, MIR34A, MIR20B, MIR92A2, MIR424, HHEX, MIR15B, CNMD, MIR222, MIR7-2, MIR410, PML, KLK3, ANGPT4, ANGPT2, STARD13, FOXO4, ATF2, MIR214, THBS2, EPN2, MIR30B, TGFB2, MIR221, E2F2, MIR21, MIR503, TNMD, MIR185, HOXA5, HRG, STAT1, MIR19B1, FOXC1, MIR137, SEMA4A, SARS, GTF2I, MIR377, MIR106B, RGCC, MIR212, MIR342, KRIT1, MMRN2, TBXA2R, MIR92A1, MIR329-1, PGK1, MIR18A, MIR200B, MIR487B, FASLG, NOTCH1, MIR17, MIR19A, MINAR1, MIR2355, WNT4, MIR7-3, MIR329-2, MIR188, MIR505, SPRY2, COL4A2, MIR24-1, MIR495, MIR20A, AMOT, MIR22, CREB3L1, SERPINF1, ADGRB2, MIR16-1, MIR361, MIR494, MIR29C, SERPINE1, PTN, NPPB, HDAC5, MIR19B2, HSPG2, MIR939, MIR15A, EPHA2 | 75 |
| 52 | GO:0014896 | muscle hypertrophy | MYOC, NPPA, GLRX3, MIR214, HDAC2, CAMTA2, MIR195, MSTN, AKAP6, SORBS2, HDAC4, RYR2, PDE5A, G6PD, MIR21, MIR15B, MIR17, ATP2B4, MIR19B2, HTR2B, MIR19A, TRPC3, MIR19B1, HAND2, PARP1, ERRFI1, BMP10, MIR25, MTOR, SLC9A1, MIR199A2, MIR199A1, TNFRSF1B, AGTR2, RGS2, MEF2A, MIR20A | 75 |
| 52 | GO:0014742 | positive regulation of muscle hypertrophy | MIR19A, MIR214, MIR21, MIR199A2, TRPC3, MIR199A1, MTOR, MEF2A, SLC9A1, AKAP6, MIR19B1, PRKCA, HAND2, PARP1, BMP10, MIR17, PDE5A, MIR20A, MIR19B2 | 75 |
| 52 | GO:0014888 | striated muscle adaptation | MYOC, NPPA, MIR499A, MIR214, CAMTA2, GSN, MSTN, HDAC4, TNNI1, IL15, MIR17, ATP2B4, GTF2IRD2, GTF2IRD1, TRPC3, RPS6KB1, MYOG, ERRFI1, PPARGC1A, CAMK2B, MIR25, BMP10, MTOR, MIR199A2, MIR199A1, GTF2IRD2B, MIR20A | 75 |
| 52 | GO:0014743 | regulation of muscle hypertrophy | MIR133A2, GLRX3, MIR214, MSTN, AKAP6, PDE5A, G6PD, MIR21, FOXO1, MIR1-1, MIR17, ATP2B4, MIR19B2, MIR19A, TRPC3, MIR19B1, GATA5, HAND2, PARP1, ERRFI1, BMP10, MIR25, MTOR, SLC9A1, MIR199A2, MIR199A1, TNFRSF1B, RGS2, MEF2A, MIR20A | 75 |
| 52 | GO:0043502 | regulation of muscle adaptation | MIR133A2, GLRX3, CTDP1, MIR214, MIR499A, AKAP6, HDAC4, MTMR4, AGT, TNNI1, PDE5A, G6PD, MIR21, FOXO1, IL6ST, MIR17, MIR1-1, ATP2B4, MIR19B2, MIR17HG, GTF2IRD2, MIR19A, TRPC3, RPS6KB1, MIR19B1, GATA5, HAND2, PARP1, MYOG, ERRFI1, PPARGC1A, CAMK2B, BMP10, NOS3, MIR25, MTOR, SLC9A1, MIR199A2, MIR199A1, TNFRSF1B, JARID2, PRKCA, RGS2, MEF2A, MIR20A | 75 |
| 52 | GO:0014744 | positive regulation of muscle adaptation | MIR17HG, TRPC3, MIR214, MTOR, MIR199A2, MIR199A1, MYOG, MIR17, MIR20A | 75 |
| 52 | GO:1903242 | regulation of cardiac muscle hypertrophy in response to stress | TRPC3, MIR214, MIR199A2, MIR199A1, MIR25, ERRFI1, MIR17, ATP2B4, MIR20A, BMP10 | 75 |
| 52 | GO:0061051 | positive regulation of cell growth involved in cardiac muscle cell development | MIR19A, MTOR, MIR199A1, MIR199A2, AKAP6, MIR19B1, MIR19B2 | 75 |
| 52 | GO:0010662 | regulation of striated muscle cell apoptotic process | MIR133A2, MIR195, EIF5A, BMP7, MIR16-2, MIR16-1, MIR34A, GHRH, MIR21, BNIP3, MIR17, MIR19B2, PDPK1, MIR19A, HSF1, MIR19B1, HAND2, MIR199A2, MIR199A1, POU4F2, JAK2, MIR20A | 75 |
| 52 | GO:0033002 | muscle cell proliferation | APLN, MIR499A, MIR214, SHH, MIR509-2, MSTN, ABCC4, MIR223, HDAC4, IRAK1, CTNNBIP1, TGFB2, MIR301A, IL12A, MIR34A, IL10, MIR221, MIR21, MIR503, MAPK11, MIR424, PTGS2, IGFBP3, MIR200B, MIR185, MIR4632, MIR17, MIR19B2, STAT1, MIR17HG, MIR222, RPS6KB1, TGM2, VIPR2, SERPINF2, MIR19B1, MIR638, MIR509-3, MYOG, XRCC6, MIR362, PPARGC1A, ADIPOQ, NOX1, COMT, CNN1, KCNK2, MIR199A2, MIR199A1, MTOR, NOTCH3, MIR137, TBX20, PTAFR, MIR509-1, MFN2, HES5, CDKN1B, MIR20A, MIR448 | 75 |
| 52 | GO:0010656 | negative regulation of muscle cell apoptotic process | MIR133A2, PTK2B, BMP7, MIR92A1, GHRH, MIR21, MIR92A2, ARRB2, MIR17, MIR19B2, PDPK1, MIR19A, HSF1, DIPK2A, MIR19B1, HAND2, ALOX12, MIR199A2, MIR199A1, LRP6, JAK2, MIR20A | 75 |
| 52 | GO:1903671 | negative regulation of sprouting angiogenesis | MIR196A2, MIR342, MIR22, MMRN2, PDCD10, CREB3L1, EPN2, TBXA2R, MIR92A1, MIR16-2, MIR16-1, MIR23B, MIR497, MIR361, MIR320A, MIR494, MIR34A, MIR221, MIR29C, E2F2, MIR329-1, MIR503, MIR92A2, MIR424, MEOX2, MIR18A, MIR15B, HDAC5, MIR487B, NOTCH1, MIR17, MIR19B2, MIR19A, MIR222, MIR7-2, MIR410, KLF4, MIR19B1, MIR2355, MIR7-3, MIR329-2, MIR188, MAP2K5, MIR15A, MIR495, CARD10, SYNJ2BP, STARD13, MIR24-1, MIR377, MIR20A | 75 |
| 52 | GO:0010612 | regulation of cardiac muscle adaptation | TRPC3, MIR214, MIR199A2, MIR199A1, MIR25, ERRFI1, MIR17, ATP2B4, MIR20A, BMP10 | 75 |
| 52 | GO:0010613 | positive regulation of cardiac muscle hypertrophy | MIR19A, MIR214, MIR21, MIR199A2, TRPC3, MIR199A1, MTOR, MEF2A, SLC9A1, AKAP6, MIR19B1, PRKCA, HAND2, PARP1, BMP10, MIR17, PDE5A, MIR20A, MIR19B2 | 75 |
| 52 | GO:0016525 | negative regulation of angiogenesis | MECP2, PDCD10, MIR16-2, MIR23B, TEK, MIR497, AGT, DAB2IP, MIR34A, MIR20B, MIR92A2, MIR424, HHEX, MIR15B, CNMD, MIR222, MIR7-2, MIR410, PML, KLK3, ANGPT4, ANGPT2, STARD13, FOXO4, ATF2, MIR214, THBS2, EPN2, MIR30B, TGFB2, MIR221, E2F2, MIR21, MIR503, TNMD, MIR185, HOXA5, HRG, STAT1, MIR19B1, FOXC1, MIR137, SEMA4A, SARS, GTF2I, MIR377, MIR106B, RGCC, MIR212, MIR342, KRIT1, MMRN2, TBXA2R, MIR92A1, MIR329-1, PGK1, MIR18A, MIR200B, MIR487B, FASLG, NOTCH1, MIR17, MIR19A, MINAR1, MIR2355, MIR7-3, MIR329-2, MIR188, MIR505, SPRY2, COL4A2, MIR24-1, MIR495, MIR20A, AMOT, MIR22, CREB3L1, SERPINF1, ADGRB2, MIR16-1, MIR361, MIR494, MIR29C, SERPINE1, PTN, NPPB, HDAC5, MIR19B2, HSPG2, MIR939, MIR15A, EPHA2 | 75 |
| 52 | GO:1901343 | negative regulation of vasculature development | MECP2, PDCD10, BMP7, MIR16-2, TEK, MIR497, AGT, DAB2IP, MIR34A, MIR20B, MIR92A2, MIR424, HHEX, MIR15B, CNMD, MIR222, MIR7-2, MIR410, PML, ANGPT4, STARD13, FOXO4, ATF2, MIR214, THBS2, EPN2, TGFB2, MIR221, E2F2, MIR21, MIR503, TNMD, MIR185, HOXA5, HRG, STAT1, MIR19B1, FOXC1, MIR137, SEMA4A, SARS, GTF2I, MIR377, MIR106B, RGCC, MIR212, MIR342, KRIT1, TBXA2R, MIR92A1, NFATC2, MIR329-1, PGK1, MIR18A, MIR200B, MIR487B, FASLG, NOTCH1, MIR17, MIR19A, MINAR1, MIR2355, WNT4, MIR7-3, MIR329-2, MIR188, MIR505, SPRY2, COL4A2, MIR495, MIR20A, AMOT, MIR22, CREB3L1, SERPINF1, ADGRB2, MIR16-1, MIR361, MIR494, MIR29C, SERPINE1, PTN, NPPB, HDAC5, MIR19B2, NFATC1, HSPG2, MIR939, MIR15A, EPHA2 | 75 |
| 52 | GO:0010611 | regulation of cardiac muscle hypertrophy | MIR133A2, GLRX3, MIR214, AKAP6, PDE5A, G6PD, MIR21, FOXO1, MIR1-1, MIR17, ATP2B4, MIR19B2, MIR19A, TRPC3, MIR19B1, GATA5, HAND2, PARP1, ERRFI1, BMP10, MIR25, MTOR, SLC9A1, MIR199A2, MIR199A1, TNFRSF1B, RGS2, MEF2A, MIR20A | 75 |

| Cluster | Term | Name | HPOs\_in\_clusters |
| --- | --- | --- | --- |
| 52 | OMIM:170390 | ANDERSEN CARDIODYSRHYTHMIC PERIODIC PARALYSIS | HP:0009803, HP:0001156, HP:0001773 |
| 52 | OMIM:190351 | TRICHORHINOPHALANGEAL SYNDROME, TYPE III; TRPS3 | HP:0009803, HP:0001156, HP:0001773 |
| 52 | OMIM:271700 | SPONDYLOPERIPHERAL DYSPLASIA | HP:0011304, HP:0001156, HP:0001773 |
| 52 | OMIM:300106 | SPONDYLOEPIMETAPHYSEAL DYSPLASIA, X-LINKED; SEMDX | HP:0009803, HP:0001156, HP:0001773 |

---


---


---

# Cluster 73

| Cluster | Term | Name |
| --- | --- | --- |
| 73 | HP:0003508 | Proportionate short stature |
| 73 | HP:0000431 | Wide nasal bridge |
| 73 | HP:0001156 | Brachydactyly |
| 73 | HP:0001773 | Short foot |

| Cluster | Term | Name | Genes | Percentage\_of\_nodes\_with\_funsys |
| --- | --- | --- | --- | --- |
| 73 | GO:0090370 | negative regulation of cholesterol efflux | MIR302A, MIR19B1, MIR33B, SREBF2, MIR130B, MIR301B, MIR33A, MIR758, MIR148A | 75 |
| 73 | GO:1903671 | negative regulation of sprouting angiogenesis | MIR196A2, MIR342, MIR22, MMRN2, PDCD10, CREB3L1, EPN2, TBXA2R, MIR92A1, MIR16-2, MIR16-1, MIR23B, MIR497, MIR361, MIR320A, MIR494, MIR34A, MIR221, MIR29C, E2F2, MIR329-1, MIR503, MIR92A2, MIR424, MEOX2, MIR18A, MIR15B, HDAC5, MIR487B, NOTCH1, MIR17, MIR19B2, MIR19A, MIR222, MIR7-2, MIR410, KLF4, MIR19B1, MIR2355, MIR7-3, MIR329-2, MIR188, MAP2K5, MIR15A, MIR495, CARD10, SYNJ2BP, STARD13, MIR24-1, MIR377, MIR20A | 75 |

| Cluster | Term | Name | HPOs\_in\_clusters |
| --- | --- | --- | --- |
| 73 | OMIM:157800 | CARDIOSPONDYLOCARPOFACIAL SYNDROME; CSCF | HP:0001156, HP:0000431, HP:0001773 |
| 73 | OMIM:305400 | AARSKOG-SCOTT SYNDROME; AAS | HP:0001156, HP:0000431, HP:0001773 |
| 73 | OMIM:614078 | CHONDRODYSPLASIA WITH JOINT DISLOCATIONS, GPAPP TYPE | HP:0001156, HP:0000431, HP:0001773 |
| 73 | OMIM:617809 | GELEOPHYSIC DYSPLASIA 3; GPHYSD3 | HP:0001156, HP:0000431, HP:0001773 |

---


---


---

# Cluster 74

| Cluster | Term | Name |
| --- | --- | --- |
| 74 | HP:0000582 | Upslanted palpebral fissure |
| 74 | HP:0001773 | Short foot |
| 74 | HP:0000506 | Telecanthus |
| 74 | HP:0010282 | Thin lower lip vermilion |

| Cluster | Term | Name | Genes | Percentage\_of\_nodes\_with\_funsys |
| --- | --- | --- | --- | --- |
| 74 | GO:0090370 | negative regulation of cholesterol efflux | MIR302A, MIR19B1, MIR33B, SREBF2, MIR130B, MIR301B, MIR33A, MIR758, MIR148A | 75 |
| 74 | GO:0010874 | regulation of cholesterol efflux | GPS2, MIR27B, TTC39B, MIR302A, PTCH1, MIR19B1, SREBF2, MIR130B, MIR301B, MIR33A, MIR758 | 75 |

| Cluster | Term | Name | HPOs\_in\_clusters |
| --- | --- | --- | --- |
| 74 | OMIM:157800 | CARDIOSPONDYLOCARPOFACIAL SYNDROME; CSCF | HP:0000506, HP:0000582, HP:0001773 |

---


---


---

# Cluster 82

| Cluster | Term | Name |
| --- | --- | --- |
| 82 | HP:0001263 | Global developmental delay |
| 82 | HP:0001290 | Generalized hypotonia |
| 82 | HP:0007018 | Attention deficit hyperactivity disorder |
| 82 | HP:0011994 | Abnormal atrial septum morphology |

| Cluster | Term | Name | Genes | Percentage\_of\_nodes\_with\_funsys |
| --- | --- | --- | --- | --- |
| 82 | GO:0050829 | defense response to Gram-negative bacterium | DEFA5, ROMO1, RNASE7, RNASE8, ADGRB1, DEFA3, DEFB127, DEFB104A, CHGA, DEFA6, IL6, SERPINE1, MR1, DEFB1, DEFA4, CD160, DEFB126, DEFB104B, RNASE6, GSDMD, DEFB103B, SELP, DEFA1B, AZU1, LBP, TNFRSF14, ELANE, DEFB128, IGHM, RNASE3, PRB3, DEFB4A, DEFB106A, DEFA1, DEFB103A, F2, BPI, DEFB118, IL22RA1, DEFB132, LYZL1, DMBT1, DEFB106B, LYZL2 | 75 |

| Cluster | Term | Name | HPOs\_in\_clusters |
| --- | --- | --- | --- |
| 82 | OMIM:176270 | PRADER-WILLI SYNDROME; PWS | HP:0001290, HP:0001263, HP:0007018 |
| 82 | OMIM:204750 | 2-AMINOADIPIC 2-OXOADIPIC ACIDURIA; AMOXAD | HP:0001290, HP:0001263, HP:0007018 |
| 82 | OMIM:300352 | CEREBRAL CREATINE DEFICIENCY SYNDROME 1; CCDS1 | HP:0001290, HP:0001263, HP:0007018 |
| 82 | OMIM:613406 | WITTEVEEN-KOLK SYNDROME; WITKOS | HP:0001290, HP:0001263, HP:0007018 |
| 82 | OMIM:617062 | OKUR-CHUNG NEURODEVELOPMENTAL SYNDROME; OCNDS | HP:0001290, HP:0001263, HP:0007018 |
| 82 | OMIM:617450 | JANSEN-DE VRIES SYNDROME; JDVS | HP:0001290, HP:0001263, HP:0007018 |
| 82 | OMIM:617600 | MENTAL RETARDATION, AUTOSOMAL DOMINANT 45; MRD45 | HP:0001290, HP:0001263, HP:0007018 |
| 82 | OMIM:617854 | MENTAL RETARDATION, AUTOSOMAL DOMINANT 56; MRD56 | HP:0001290, HP:0001263, HP:0007018 |
| 82 | OMIM:617991 | DEVELOPMENTAL DELAY, INTELLECTUAL DISABILITY, OBESITY, AND DYSMORPHISM; DIDOD | HP:0001290, HP:0001263, HP:0007018 |
| 82 | OMIM:618050 | MENTAL RETARDATION, AUTOSOMAL DOMINANT 57; MRD57 | HP:0001290, HP:0001263, HP:0007018 |

---


---


---

# Cluster 88

| Cluster | Term | Name |
| --- | --- | --- |
| 88 | HP:0000343 | Long philtrum |
| 88 | HP:0000369 | Low-set ears |
| 88 | HP:0004209 | Clinodactyly of the 5th finger |
| 88 | HP:0000954 | Single transverse palmar crease |

| Cluster | Term | Name | Genes | Percentage\_of\_nodes\_with\_funsys |
| --- | --- | --- | --- | --- |
| 88 | GO:0010633 | negative regulation of epithelial cell migration | MIR212, MIR196A2, MIR129-1, KRIT1, CXCL13, MIR22, MECP2, SERPINF1, PDCD10, MMRN2, PFN2, IL4, NR2F2, MIR16-2, MIR92A1, MIR16-1, RGCC, TBXA2R, MIR497, PTPRR, MIR361, MIR320A, DAB2IP, EVL, MIR494, MIR29C, MIR221, MIR329-1, MIR21, MIR503, MIR424, MIR92A2, MIR200B, MIR15B, HDAC5, NOTCH1, HRG, MIR19B2, FGF2, KLF4, MIR410, MIR132, MIR19B1, DUSP10, SVBP, ADIPOR1, MIR329-2, MIR188, PTEN, MIR204, MIR505, HMGB1, BMP10, MIR15A, ANGPT4, MIR137, AGTR2, ANGPT2, MIR495, CARD10, STC1, STARD13, MIR24-1, MIR20A, EPPK1 | 75 |
| 88 | GO:0090051 | negative regulation of cell migration involved in sprouting angiogenesis | MIR196A2, MIR22, MMRN2, PDCD10, TBXA2R, MIR16-2, MIR16-1, MIR497, MIR361, MIR494, MIR221, MIR29C, MIR329-1, MIR503, MIR424, HDAC5, NOTCH1, MIR19B2, KLF4, MIR410, MIR19B1, MIR2355, MIR329-2, MIR188, MIR15A, MIR495, STARD13, MIR20A | 75 |
| 88 | GO:1903670 | regulation of sprouting angiogenesis | FGFBP1, PDCD10, BMPER, MIR16-2, MIR23B, AGTR1, MIR497, MIR34A, MIR92A2, MIR424, MIR15B, MIR31, SMAD1, FGF2, MIR222, MIR410, MIR7-2, MIR132, ANXA1, HMGB1, MAP2K5, MIRLET7F1, STARD13, PPP1R16B, MIR196A2, HDAC9, EPN2, MIR296, MIR30B, MIR221, JCAD, E2F2, MIR503, PDPK1, CEMIP2, MIR19B1, SRPX2, PKM, AKT3, MIR377, MIR27B, MIR342, MMRN2, MIR150, ITGA5, TBXA2R, MIR92A1, IL10, ABL1, MIR329-1, MIR1224, MIR18A, MIR487B, NOTCH1, MIR17, MIR19A, MIR101-2, DLL1, MIRLET7F2, JMJD8, MIR2355, MIR7-3, MIR329-2, MIR188, KLF2, MIR126, MIR193A, CARD10, MIR24-1, MIR20A, MIR495, MIR22, GHSR, MIR16-1, GHRL, MIR361, MIR494, MEOX2, MIR29C, HMOX1, PTGS2, NRP1, HDAC5, MIR1-1, FOXC2, MIR19B2, KLF4, MIR15A, SYNJ2BP | 75 |
| 88 | GO:0002040 | sprouting angiogenesis | FGFBP1, EGR3, PDCD10, BMPER, MIR16-2, MIR23B, AGTR1, TEK, MIR497, MIR320A, MIR34A, EFNB2, MIR92A2, MIR424, MIR15B, FLT4, MIR31, SMAD1, FGF2, MIR7-2, MIR410, MIR222, ANXA1, HMGB1, MAP2K5, EPHB4, MIRLET7F1, STARD13, PPP1R16B, MIR196A2, ADGRA2, HDAC9, EPN2, MIR296, MIR30B, MIR221, JCAD, E2F2, MIR503, PDPK1, CEMIP2, MIR19B1, SRPX2, PKM, AKT3, MIR377, MIR27B, MIR342, MIR150, ITGA5, MMRN2, TBXA2R, MIR92A1, SEMA3E, NRARP, IL10, ABL1, E2F7, MIR329-1, MIR1224, MIR18A, MIR487B, NOTCH1, MIR17, MIR19A, SEMA5A, MIR101-2, DLL1, MIRLET7F2, MIR2355, JMJD8, MIR329-2, MIR188, KLF2, MIR126, MIR193A, LOXL2, CARD10, MIR24-1, MIA3, MIR20A, MIR495, PTK2B, MIR22, GHSR, MIR16-1, GHRL, MIR361, OTULIN, MIR494, MIR29C, HMOX1, PTGS2, NRP1, HDAC5, VEGFB, CDH13, AKT1, MIR1-1, FOXC2, MIR19B2, KLF4, CDC42, MIR15A, SYNJ2BP, ITGB1, EPHA2 | 75 |

| Cluster | Term | Name | HPOs\_in\_clusters |
| --- | --- | --- | --- |
| 88 | OMIM:122470 | CORNELIA DE LANGE SYNDROME 1; CDLS1 | HP:0000954, HP:0000369, HP:0000343, HP:0004209 |
| 88 | OMIM:261540 | PETERS-PLUS SYNDROME; PTRPLS | HP:0000954, HP:0000369, HP:0000343, HP:0004209 |
| 88 | OMIM:113620 | BRANCHIOOCULOFACIAL SYNDROME; BOFS | HP:0000954, HP:0000369, HP:0004209 |
| 88 | OMIM:117650 | CEREBROCOSTOMANDIBULAR SYNDROME; CCMS | HP:0000369, HP:0000343, HP:0004209 |
| 88 | OMIM:180849 | RUBINSTEIN-TAYBI SYNDROME 1; RSTS1 | HP:0000954, HP:0000369, HP:0004209 |
| 88 | OMIM:210600 | SECKEL SYNDROME 1; SCKL1 | HP:0000954, HP:0000369, HP:0004209 |
| 88 | OMIM:228520 | FIBROCHONDROGENESIS 1; FBCG1 | HP:0000369, HP:0000343, HP:0004209 |
| 88 | OMIM:301022 | NEURODEVELOPMENTAL DISORDER, X-LINKED, WITH CRANIOFACIAL ABNORMALITIES; NEDXCF | HP:0000369, HP:0000343, HP:0004209 |
| 88 | OMIM:605130 | WIEDEMANN-STEINER SYNDROME; WDSTS | HP:0000369, HP:0000343, HP:0004209 |
| 88 | OMIM:616145 | CATEL-MANZKE SYNDROME; CATMANS | HP:0000954, HP:0000369, HP:0004209 |
| 88 | OMIM:617527 | NEURODEVELOPMENTAL DISORDER WITH PROGRESSIVE MICROCEPHALY, SPASTICITY, AND BRAIN ANOMALIES; NDMSBA | HP:0000954, HP:0000369, HP:0000343 |
| 88 | OMIM:617877 | SHORT STATURE, FACIAL DYSMORPHISM, AND SKELETAL ANOMALIES WITH OR WITHOUT CARDIAC ANOMALIES; SSFSC | HP:0000369, HP:0000343, HP:0004209 |

---


---


---

# Cluster 2

| Cluster | Term | Name |
| --- | --- | --- |
| 2 | HP:0005107 | Abnormal sacrum morphology |
| 2 | HP:0011927 | Short digit |
| 2 | HP:0100491 | Abnormality of lower limb joint |

| Cluster | Term | Name | Genes | Percentage\_of\_nodes\_with\_funsys |
| --- | --- | --- | --- | --- |
| 2 | GO:1905563 | negative regulation of vascular endothelial cell proliferation | MIR34A, FLT1, MIR29C, MIR20B, MIR129-1, MIR329-1, MIR503, MIR424, MIR132, MEF2C, MIR98, MIR24-1, MIR15B, MIR30B, MIR329-2, MIR487B, MIR126, MIR495 | 100 |
| 2 | GO:0050680 | negative regulation of epithelial cell proliferation | NKX3-1, CDC73, SLURP1, PEX2, MIR129-1, MIR342, KRIT1, MIR22, PDCD10, NLRC3, CASK, SERPINF1, MIR16-2, MIR16-1, RGCC, MIR30B, CDKN2B, TGFB2, GHRL, MIR497, MIR361, KRT4, MIR29B1, DAB2IP, MIR494, MIR34A, MIR29C, EFNB2, RB1, MIR329-1, MIR503, MIR20B, MIR424, MIR21, SULF1, NFIB, GDF5, TNMD, PTN, ATP5IF1, MIR15B, MIR98, MIR487B, IFT74, CDK6, NF1, STAT1, CAV1, IFT80, MIR410, PTCH1, KLF9, MIR132, DUSP10, DLG1, MIR329-2, PTEN, MIR29B2, AR, MIR126, GPC3, MAGED1, MIR193A, FLT1, MIR15A, GATA3, A4GNT, SFRP1, CPB2, CAV2, SYNJ2BP, MIR24-1, WDR13, ATF2, INTU, MIR495, EPPK1 | 100 |
| 2 | GO:0090049 | regulation of cell migration involved in sprouting angiogenesis | MIR27B, MIR196A2, FGFBP1, MIR22, MMRN2, PDCD10, HDAC9, TBXA2R, MIR16-2, MIR23B, MIR296, MIR16-1, MIR497, MIR361, MIR494, JCAD, MEOX2, MIR29C, ABL1, MIR329-1, MIR503, MIR221, MIR424, PTGS2, HDAC5, MIR487B, NOTCH1, MIR31, FOXC2, MIR19B2, FGF2, MIR101-2, MIR410, KLF4, MIR19B1, MIR2355, MIRLET7F2, MIR132, MIR329-2, MIR188, ANXA1, MIR126, MAP2K5, MIR193A, MIR15A, SRPX2, MIR495, MIRLET7F1, STARD13, AKT3, MIR20A | 100 |

---


---


---

# Cluster 10

| Cluster | Term | Name |
| --- | --- | --- |
| 10 | HP:0006919 | Abnormal aggressive, impulsive or violent behavior |
| 10 | HP:0004686 | Short third metatarsal |
| 10 | HP:0004689 | Short fourth metatarsal |
| 10 | HP:0010012 | Abnormality of the 4th metacarpal |
| 10 | HP:0010013 | Abnormality of the 5th metacarpal |
| 10 | HP:0010042 | Aplasia/Hypoplasia of the 4th metacarpal |
| 10 | HP:0010045 | Aplasia/Hypoplasia of the 5th metacarpal |

| Cluster | Term | Name | Genes | Percentage\_of\_nodes\_with\_funsys |
| --- | --- | --- | --- | --- |
| 10 | GO:0005996 | monosaccharide metabolic process | WDTC1, FUOM, SERP1, UGT2B28, NKX1-1, C1QTNF12, PPP1R3F, PASK, TFF3, UGT1A10, IGFBP3, PGAM2, MAN2B1, PKLR, PMM1, HMGB1, UGT1A6, UGT2B15, UGT2B4, ALDH1A1, PRKN, SDHAF3, G6PC3, PGM1, COX11, CYB5R3, SLC25A1, MPI, FUT7, UGT1A5, MAN2C1, FUT9, PER2, CYB5A, OMA1, HK3, FBP1, MAEA, FBP2, GCG, TKTL1, SESN2, FAM3A, IRS2, MTOR, ALDOB, ATF4, PKM, ONECUT1, USF1, PPP4R3B, SELENOS, UGT1A8, SLC25A12, SLC25A13, MLYCD, MDH1, PGM5, PGP, NLN, FPGT, UGT1A7, B3GLCT, G6PD, ARPP19, DGKQ, ENO1, UGT2A1, PRKAA1, UGT1A1, H6PD, ALDOA, ATF3, SLC35A2, RBP4, SLC25A11, GCK, UGT2B11, UGT2A3, ADIPOR1, FGGY, BRAT1, SLC2A1, UGT1A9, G6PC2, ENO3, CHST15, MIR103A1, SLC45A3, PPP1R3B, CRTC2, PFKFB3, NCOA2, UGT2B7, FUCA1, POFUT1, CLK2, PGD, INPP5K, GALE, AKT1, PDK4, SOGA1, PDK1, PFKP, SRC, UGT1A3, FAM3C, PPARA, UGT1A4, GNMT | 100 |

---


---


---

# Cluster 21

| Cluster | Term | Name |
| --- | --- | --- |
| 21 | HP:0000126 | Hydronephrosis |
| 21 | HP:0001829 | Foot polydactyly |
| 21 | HP:0002901 | Hypocalcemia |
| 21 | HP:0005562 | Multiple renal cysts |

| Cluster | Term | Name | Genes | Percentage\_of\_nodes\_with\_funsys |
| --- | --- | --- | --- | --- |
| 21 | GO:0046394 | carboxylic acid biosynthetic process | AKR1C3, DHTKD1, PFKFB3, GGTLC3, MIF, CYP1A1, OGDH, PGAM2, MIR185, ACACA, GGT3P, HACD1, MALRD1, SEPHS1, GCK, PFKP, PRXL2B, GGTLC2, OLAH, GGT5, ADPGK, AKR1C4, PKM, CYP1A2 | 100 |
| 21 | GO:0016053 | organic acid biosynthetic process | AKR1C3, DHTKD1, PFKFB3, GGTLC3, MIF, CYP1A1, OGDH, PGAM2, MIR185, ACACA, GGT3P, HACD1, MALRD1, SEPHS1, GCK, PFKP, PRXL2B, GGTLC2, OLAH, GGT5, ADPGK, AKR1C4, PKM, CYP1A2 | 100 |

---


---


---

# Cluster 23

| Cluster | Term | Name |
| --- | --- | --- |
| 23 | HP:0001171 | Split hand |
| 23 | HP:0001839 | Split foot |
| 23 | HP:0008050 | Abnormality of the palpebral fissures |
| 23 | HP:0000288 | Abnormality of the philtrum |

| Cluster | Term | Name | Genes | Percentage\_of\_nodes\_with\_funsys |
| --- | --- | --- | --- | --- |
| 23 | GO:0009067 | aspartate family amino acid biosynthetic process | PLOD3, PLOD1, MTHFR, PLOD2, AASS, MTR, MRI1, ENOPH1, ASNS | 100 |

---


---


---

# Cluster 28

| Cluster | Term | Name |
| --- | --- | --- |
| 28 | HP:0000369 | Low-set ears |
| 28 | HP:0000431 | Wide nasal bridge |
| 28 | HP:0000954 | Single transverse palmar crease |
| 28 | HP:0000960 | Sacral dimple |
| 28 | HP:0000581 | Blepharophimosis |

| Cluster | Term | Name | Genes | Percentage\_of\_nodes\_with\_funsys |
| --- | --- | --- | --- | --- |
| 28 | GO:0043534 | blood vessel endothelial cell migration | CLEC14A, EGR3, MECP2, PDCD10, MIR16-2, MIR23B, SH3BP1, MIR497, FGFR1, MIR320A, EFNB2, MIR92A2, MIR424, MIR15B, MIR31, FGF2, MIR410, MIR132, ANXA1, HMGB1, EPHB4, ANGPT4, ANGPT2, PRKCA, MIRLET7F1, STARD13, MIR196A2, SOX18, FGF18, JCAD, MIR503, NF1, HRG, PDPK1, MIR19B1, SP1, SRPX2, MIR137, AKT3, RGCC, MIR27B, MIR212, MIR342, NUS1, MMRN2, TBXA2R, MIR92A1, MIR329-1, MIR200B, MIR487B, NOTCH1, MIR101-2, MIRLET7F2, MIR2355, MIR329-2, MIR188, MIR204, MIR505, MIR193A, NOS3, CARD10, MIR24-1, VEGFC, MIA3, MIR20A, MIR495, PRKD1, MIR499A, PTK2B, AMOT, MIR22, MIR16-1, MIR361, MYH9, MIR494, MIR200A, MIR29C, HMOX1, PTGS2, HDAC5, ID1, AKT1, MIR19B2, KLF4, GREM1, PDGFB, MIR939, MIR15A, AGTR2 | 80 |
| 28 | GO:1905651 | regulation of artery morphogenesis | MIR494, EFNB2, MIR329-1, MIR153-2, MIR29B2, MIR329-2, MIR487B, NOTCH1, MIR205, AKT3, MIR495, MIR29B1 | 80 |
| 28 | GO:1902547 | regulation of cellular response to vascular endothelial growth factor stimulus | JCAD, ADGRA2, MIR329-1, MIR342, DLL1, MIR424, MYO1C, CD63, MIR16-2, SMOC2, MIR16-1, MIR329-2, HRG, SPRY2, DAB2IP | 100 |
| 28 | GO:1903588 | negative regulation of blood vessel endothelial cell proliferation involved in sprouting angiogenesis | MIR494, MIR29C, MIR15A, MIR410, MIR342, MIR503, MIR424, PDCD10, MIR16-2, MIR15B, MIR24-1, MIR16-1, MIR497, MIR361, MIR495, MIR193A | 100 |
| 28 | GO:0090130 | tissue migration | LGMN, EGR3, PDCD10, S100P, LGALS8, MIR16-2, MIR23B, SH3BP1, TEK, MIR497, FGFR1, MIR320A, EVL, EFNB2, MIR92A2, MIR424, KANK1, TBXT, MIR15B, MIR31, MIR410, MIR132, ANXA1, HMGB1, EPHB4, ANGPT4, ANGPT2, MIRLET7F1, STARD13, MIR196A2, ADGRA2, SOX18, ACTA2, TGFB2, JCAD, MIR21, MIR503, FGF16, TNFSF12, HDAC6, HRG, FGF4, PDPK1, FOXP1, MIR19B1, PTEN, BMP10, SP1, PAXIP1, MTOR, SRPX2, MIR137, SEMA4A, RAB25, DOCK5, AKT3, CAPN7, RGCC, MIR212, MIR27B, MIR342, KRIT1, NUS1, MMRN2, TBXA2R, MIR92A1, PRSS3, MIR329-1, PROX1, CALR, PRKX, MIR200B, MIR487B, NOTCH1, SEMA5A, MIR101-2, MIRLET7F2, KRT2, ADIPOR1, MIR329-2, MIR188, MIR505, MIR126, NOS3, GATA3, LOXL2, PPM1F, CARD10, STC1, MIR24-1, MIR20A, MIR495, PRKD1, ZEB2, MIR499A, PTK2B, MIR22, CCR6, SERPINF1, AMOT, PFN2, MIR16-1, PTPRR, MIR361, MYH9, MIR494, MIR200A, MIR29C, HMOX1, ADAM9, PTGS2, HDAC5, ID1, AKT1, MIR19B2, KANK2, MAP4K4, KLF4, SASH1, PDGFB, SRC, DUSP10, SVBP, SMOC2, EPB41L4B, MIR939, CORO1B, MIR15A, HBEGF, AGTR2, BCAS3, EPPK1 | 80 |
| 28 | GO:0101023 | vascular endothelial cell proliferation | APLN, MIR499A, MIR129-1, GHSR, SIRT6, MIR30B, GHRL, MIR34A, MIR20B, MIR329-1, MIR503, MIR21, MIR424, MIR98, MIR15B, MIR487B, PDPK1, FGF2, ITGA4, MIR329-2, HMGB1, MIR126, FLT1, MIR29A, MIR24-1, AKT3, MIR495 | 100 |
| 28 | GO:1903587 | regulation of blood vessel endothelial cell proliferation involved in sprouting angiogenesis | MIR27B, MIR342, FGFBP1, PDCD10, MIR16-2, MIR23B, MIR16-1, AGTR1, MIR497, MIR361, MIR494, JCAD, MIR29C, HMOX1, MIR503, MIR424, MIR15B, MIR487B, MIR101-2, MIR410, MIR2355, MIR126, MIR193A, MIR15A, MIRLET7B, MIR24-1, PPP1R16B, MIR495 | 100 |
| 28 | GO:0038084 | vascular endothelial growth factor signaling pathway | FLT1, JCAD, MIR329-1, MIR342, DLL1, MIR424, MYO1C, MIR16-2, SMOC2, MIR329-2 | 80 |
| 28 | GO:1900746 | regulation of vascular endothelial growth factor signaling pathway | JCAD, ADGRA2, MIR329-1, MIR342, DLL1, MIR424, MYO1C, CD63, MIR16-2, SMOC2, MIR16-1, MIR329-2, HRG, SPRY2, DAB2IP | 100 |
| 28 | GO:0010633 | negative regulation of epithelial cell migration | MIR212, MIR196A2, MIR129-1, KRIT1, CXCL13, MIR22, MECP2, SERPINF1, PDCD10, MMRN2, PFN2, IL4, NR2F2, MIR16-2, MIR92A1, MIR16-1, RGCC, TBXA2R, MIR497, PTPRR, MIR361, MIR320A, DAB2IP, EVL, MIR494, MIR29C, MIR221, MIR329-1, MIR21, MIR503, MIR424, MIR92A2, MIR200B, MIR15B, HDAC5, NOTCH1, HRG, MIR19B2, FGF2, KLF4, MIR410, MIR132, MIR19B1, DUSP10, SVBP, ADIPOR1, MIR329-2, MIR188, PTEN, MIR204, MIR505, HMGB1, BMP10, MIR15A, ANGPT4, MIR137, AGTR2, ANGPT2, MIR495, CARD10, STC1, STARD13, MIR24-1, MIR20A, EPPK1 | 100 |
| 28 | GO:1905563 | negative regulation of vascular endothelial cell proliferation | MIR34A, FLT1, MIR29C, MIR20B, MIR129-1, MIR329-1, MIR503, MIR424, MIR132, MEF2C, MIR98, MIR24-1, MIR15B, MIR30B, MIR329-2, MIR487B, MIR126, MIR495 | 100 |
| 28 | GO:1902548 | negative regulation of cellular response to vascular endothelial growth factor stimulus | ADGRA2, MIR329-1, MIR342, MIR424, MIR16-2, MIR16-1, MIR329-2, HRG, SPRY2, DAB2IP | 80 |
| 28 | GO:0010594 | regulation of endothelial cell migration | LGMN, CXCL13, PDCD10, MIR16-2, MIR23B, SH3BP1, TEK, MIR497, FGFR1, MIR320A, MIR92A2, MIR424, MIR15B, MIR31, MIR410, ANXA1, HMGB1, ANGPT4, ANGPT2, PRKCA, MIRLET7F1, STARD13, MIR196A2, ADGRA2, MIR296, JCAD, MIR21, MIR503, FGF16, NF1, HRG, FGF4, PDPK1, FOXP1, MIR19B1, BMP10, SP1, SRPX2, MIR137, SEMA4A, ANXA3, AKT3, RGCC, MIR27B, MIR129-1, MIR342, KRIT1, NUS1, MMRN2, TBXA2R, MIR92A1, MIR329-1, PROX1, CALR, MIR200B, MIR487B, NOTCH1, SEMA5A, MIR101-2, MIRLET7F2, MIR329-2, MIR188, MIR204, MIR505, MIR126, MIR193A, NOS3, GATA3, MIR29A, CARD10, STC1, MIR24-1, MIR20A, MIR495, PRKD1, MIR499A, PTK2B, MIR22, SERPINF1, MIR16-1, MIR361, MIR200A, MIR494, MIR29C, HMOX1, PTGS2, HDAC5, AKT1, MIR19B2, MET, KLF4, SASH1, PDGFB, SVBP, SMOC2, MIR939, MIR15A, AGTR2, BCAS3 | 80 |
| 28 | GO:0002043 | blood vessel endothelial cell proliferation involved in sprouting angiogenesis | MIR27B, MIR342, FGFBP1, PDCD10, BMPER, MIR16-2, MIR23B, NRARP, AGTR1, MIR16-1, MIR497, MIR494, JCAD, MIR29C, EPHA2, HMOX1, MIR503, MIR424, MIR15B, MIR487B, SEMA5A, MIR101-2, MIR410, BMP4, MIR126, MIR193A, MIR15A, MIRLET7B, MIR24-1, PPP1R16B, MIR495 | 100 |
| 28 | GO:0043537 | negative regulation of blood vessel endothelial cell migration | MIR212, MIR22, MECP2, PDCD10, MMRN2, TBXA2R, MIR92A1, MIR16-1, MIR16-2, MIR361, MIR494, MIR221, MIR29C, MIR329-1, MIR503, MIR92A2, MIR424, MIR200B, MIR15B, NOTCH1, MIR19B2, MIR410, MIR19B1, MIR132, MIR329-2, MIR188, MIR505, HMGB1, MIR15A, ANGPT4, MIR137, AGTR2, ANGPT2, MIR495, STARD13, MIR24-1, MIR20A, RGCC | 80 |
| 28 | GO:0050680 | negative regulation of epithelial cell proliferation | NKX3-1, CDC73, SLURP1, PEX2, MIR129-1, MIR342, KRIT1, MIR22, PDCD10, NLRC3, CASK, SERPINF1, MIR16-2, MIR16-1, RGCC, MIR30B, CDKN2B, TGFB2, GHRL, MIR497, MIR361, KRT4, MIR29B1, DAB2IP, MIR494, MIR34A, MIR29C, EFNB2, RB1, MIR329-1, MIR503, MIR20B, MIR424, MIR21, SULF1, NFIB, GDF5, TNMD, PTN, ATP5IF1, MIR15B, MIR98, MIR487B, IFT74, CDK6, NF1, STAT1, CAV1, IFT80, MIR410, PTCH1, KLF9, MIR132, DUSP10, DLG1, MIR329-2, PTEN, MIR29B2, AR, MIR126, GPC3, MAGED1, MIR193A, FLT1, MIR15A, GATA3, A4GNT, SFRP1, CPB2, CAV2, SYNJ2BP, MIR24-1, WDR13, ATF2, INTU, MIR495, EPPK1 | 100 |
| 28 | GO:1900747 | negative regulation of vascular endothelial growth factor signaling pathway | ADGRA2, MIR329-1, MIR342, MIR424, MIR16-2, MIR16-1, MIR329-2, HRG, SPRY2, DAB2IP | 100 |
| 28 | GO:0010632 | regulation of epithelial cell migration | LGMN, CXCL13, PDCD10, MIR16-2, MIR23B, SH3BP1, DOCK1, TEK, MIR497, AGT, FGFR1, MIR320A, EVL, MIR92A2, MIR424, MIR15B, MIR31, MIR410, ANXA1, HMGB1, ANGPT4, ANGPT2, PRKCA, MIRLET7F1, STARD13, MIR196A2, ADGRA2, MIR296, TGFB2, JCAD, MIR21, MIR503, FGF16, HDAC6, HRG, FGF4, PDPK1, FOXP1, MIR19B1, PTEN, BMP10, SP1, MTOR, SRPX2, MIR137, SEMA4A, RAB25, DOCK5, ANXA3, AKT3, CAPN7, RGCC, MIR27B, MIR129-1, MIR342, KRIT1, NUS1, MMRN2, TBXA2R, MIR92A1, MIR329-1, PROX1, CALR, MIR200B, MIR487B, NOTCH1, SEMA5A, MIR101-2, MIRLET7F2, ADIPOR1, MIR329-2, MIR188, MIR204, MIR505, MIR126, NOS3, GATA3, MIR29A, PPM1F, CARD10, STC1, MIR24-1, MIR495, MIR20A, PRKD1, MIR499A, PTK2B, MIR22, CCR6, SERPINF1, PFN2, MIR16-1, PTPRR, MIR361, MIR200A, MIR494, MIR29C, HMOX1, ADAM9, PTGS2, HDAC5, AKT1, MIR19B2, MET, MAP4K4, KLF4, SASH1, PDGFB, SRC, DUSP10, SVBP, SMOC2, EPB41L4B, MIR939, MIR135B, MIR15A, HBEGF, AGTR2, EPPK1 | 80 |
| 28 | GO:0090051 | negative regulation of cell migration involved in sprouting angiogenesis | MIR196A2, MIR22, MMRN2, PDCD10, TBXA2R, MIR16-2, MIR16-1, MIR497, MIR361, MIR494, MIR221, MIR29C, MIR329-1, MIR503, MIR424, HDAC5, NOTCH1, MIR19B2, KLF4, MIR410, MIR19B1, MIR2355, MIR329-2, MIR188, MIR15A, MIR495, STARD13, MIR20A | 100 |
| 28 | GO:0010596 | negative regulation of endothelial cell migration | MIR212, MIR196A2, MIR129-1, KRIT1, CXCL13, MIR22, MECP2, PDCD10, SERPINF1, MMRN2, TBXA2R, MIR16-2, MIR92A1, MIR16-1, MIR497, MIR361, MIR320A, DAB2IP, MIR494, MIR221, MIR29C, MIR329-1, MIR21, MIR503, MIR424, MIR92A2, MIR200B, MIR15B, HDAC5, HRG, MIR19B2, KLF4, MIR410, MIR19B1, MIR132, MIR329-2, MIR188, MIR204, MIR505, HMGB1, BMP10, MIR15A, ANGPT4, MIR137, AGTR2, ANGPT2, MIR495, STC1, STARD13, MIR24-1, MIR20A, RGCC | 80 |
| 28 | GO:1903670 | regulation of sprouting angiogenesis | FGFBP1, PDCD10, BMPER, MIR16-2, MIR23B, AGTR1, MIR497, MIR34A, MIR92A2, MIR424, MIR15B, MIR31, SMAD1, FGF2, MIR222, MIR410, MIR7-2, MIR132, ANXA1, HMGB1, MAP2K5, MIRLET7F1, STARD13, PPP1R16B, MIR196A2, HDAC9, EPN2, MIR296, MIR30B, MIR221, JCAD, E2F2, MIR503, PDPK1, CEMIP2, MIR19B1, SRPX2, PKM, AKT3, MIR377, MIR27B, MIR342, MMRN2, MIR150, ITGA5, TBXA2R, MIR92A1, IL10, ABL1, MIR329-1, MIR1224, MIR18A, MIR487B, NOTCH1, MIR17, MIR19A, MIR101-2, DLL1, MIRLET7F2, JMJD8, MIR2355, MIR7-3, MIR329-2, MIR188, KLF2, MIR126, MIR193A, CARD10, MIR24-1, MIR20A, MIR495, MIR22, GHSR, MIR16-1, GHRL, MIR361, MIR494, MEOX2, MIR29C, HMOX1, PTGS2, NRP1, HDAC5, MIR1-1, FOXC2, MIR19B2, KLF4, MIR15A, SYNJ2BP | 100 |
| 28 | GO:0001936 | regulation of endothelial cell proliferation | PRKD1, MIR27B, APLN, MIR499A, MYDGF, MIR129-1, MIR342, FGFBP1, KRIT1, MIR22, PDCD10, EGR3, GHSR, NR2F2, MIR16-2, MIR16-1, NRARP, AGTR1, SIRT6, TEK, LEP, MIR23B, MIR497, MIR30B, GHRL, MIR361, FGFR1, MIR494, JCAD, MIR34A, IL10, MIR29C, MIR329-1, MIR503, PROX1, MIR424, MIR20B, WNT2, TNFSF12, EGFL7, MIR21, TNMD, ATP5IF1, RICTOR, MIR15B, VEGFB, MIR487B, AKT1, STAT1, PDPK1, CAV1, SEMA5A, FGF2, MIR101-2, MIR410, VASH2, VIP, HTR2B, ITGA4, MIR132, PDGFB, MIR329-2, HMGB1, MIR126, MIR135B, PLXNB3, SP1, FLT1, MIR15A, MTOR, LRG1, CAV2, MIR29A, PRKCA, SYNJ2BP, MIR24-1, PPP1R16B, HMOX1, AKT3, MIR495, RGCC | 100 |
| 28 | GO:0090132 | epithelium migration | LGMN, CXCL13, EGR3, PDCD10, S100P, LGALS8, MIR16-2, MIR23B, SH3BP1, TEK, MIR497, FGFR1, MIR320A, EVL, EFNB2, MIR424, KANK1, MIR15B, MIR31, MIR410, MIR132, ANXA1, HMGB1, EPHB4, ANGPT4, ANGPT2, MIRLET7F1, STARD13, MIR196A2, ADGRA2, SOX18, MIR296, TGFB2, JCAD, MIR21, MIR503, TNFSF12, HDAC6, HRG, FGF4, PDPK1, FOXP1, MIR19B1, PTEN, BMP10, SP1, PAXIP1, MTOR, MIR137, SEMA4A, RAB25, DOCK5, ANXA3, AKT3, CAPN7, RGCC, MIR212, MIR27B, MIR342, KRIT1, NUS1, MMRN2, TBXA2R, MIR92A1, PRSS3, MIR329-1, PROX1, CALR, PRKX, MIR200B, MIR487B, SEMA5A, MIR101-2, KRT2, ADIPOR1, MIR329-2, MIR188, NOS3, GATA3, LOXL2, PPM1F, CARD10, STC1, MIR24-1, MIR20A, MIR495, PRKD1, ZEB2, MIR499A, PTK2B, MIR22, CCR6, SERPINF1, PFN2, MIR16-1, PTPRR, MYH9, MIR494, MIR200A, MIR29C, HMOX1, ADAM9, PTGS2, HDAC5, ID1, AKT1, MAP4K4, KLF4, SRC, DUSP10, SVBP, SMOC2, EPB41L4B, MIR939, CORO1B, MIR15A, HBEGF, BCAS3, EPPK1 | 80 |
| 28 | GO:1903671 | negative regulation of sprouting angiogenesis | MIR196A2, MIR342, MIR22, MMRN2, PDCD10, CREB3L1, EPN2, TBXA2R, MIR92A1, MIR16-2, MIR16-1, MIR23B, MIR497, MIR361, MIR320A, MIR494, MIR34A, MIR221, MIR29C, E2F2, MIR329-1, MIR503, MIR92A2, MIR424, MEOX2, MIR18A, MIR15B, HDAC5, MIR487B, NOTCH1, MIR17, MIR19B2, MIR19A, MIR222, MIR7-2, MIR410, KLF4, MIR19B1, MIR2355, MIR7-3, MIR329-2, MIR188, MAP2K5, MIR15A, MIR495, CARD10, SYNJ2BP, STARD13, MIR24-1, MIR377, MIR20A | 100 |
| 28 | GO:0001935 | endothelial cell proliferation | APLN, FGFBP1, EGR3, PDCD10, MIR16-2, MIR23B, AGTR1, TEK, MIR497, FGFR1, MIR34A, MIR20B, MIR424, MIR15B, CAV1, FGF2, MIR410, MIR132, HMGB1, PLXNB3, LRG1, PRKCA, PPP1R16B, MYDGF, NR2F2, SIRT6, MIR30B, LEP, JCAD, MIR21, MIR503, WNT2, TNFSF12, TNMD, ATP5IF1, RICTOR, STAT1, PDPK1, SP1, FLT1, MTOR, AKT3, RGCC, MIR27B, MIR129-1, MIR342, KRIT1, NRARP, IL10, MIR329-1, PROX1, PRKX, MIR487B, HTR2B, SEMA5A, MIR101-2, ITGA4, MIR329-2, MIR126, LOXL2, CAV2, MIR29A, MIR24-1, CD34, MIR495, PRKD1, MIR499A, MIR22, GHSR, MIR16-1, PIK3CB, GHRL, MIR361, MIR494, MIR29C, HMOX1, EGFL7, VEGFB, AKT1, THAP1, VIP, VASH2, PDGFB, DLG1, MIR135B, MIR15A, SYNJ2BP, EPHA2 | 100 |
| 28 | GO:0001937 | negative regulation of endothelial cell proliferation | MIR129-1, MIR342, KRIT1, MIR22, PDCD10, NR2F2, MIR16-2, MIR16-1, MIR30B, GHRL, MIR497, MIR361, MIR494, MIR34A, MIR20B, MIR329-1, MIR503, MIR21, MIR424, TNMD, ATP5IF1, MIR98, MIR15B, MIR487B, STAT1, CAV1, MIR410, MIR329-2, MIR126, FLT1, MIR15A, CAV2, SYNJ2BP, MIR24-1, MIR495, RGCC | 100 |
| 28 | GO:0002040 | sprouting angiogenesis | FGFBP1, EGR3, PDCD10, BMPER, MIR16-2, MIR23B, AGTR1, TEK, MIR497, MIR320A, MIR34A, EFNB2, MIR92A2, MIR424, MIR15B, FLT4, MIR31, SMAD1, FGF2, MIR7-2, MIR410, MIR222, ANXA1, HMGB1, MAP2K5, EPHB4, MIRLET7F1, STARD13, PPP1R16B, MIR196A2, ADGRA2, HDAC9, EPN2, MIR296, MIR30B, MIR221, JCAD, E2F2, MIR503, PDPK1, CEMIP2, MIR19B1, SRPX2, PKM, AKT3, MIR377, MIR27B, MIR342, MIR150, ITGA5, MMRN2, TBXA2R, MIR92A1, SEMA3E, NRARP, IL10, ABL1, E2F7, MIR329-1, MIR1224, MIR18A, MIR487B, NOTCH1, MIR17, MIR19A, SEMA5A, MIR101-2, DLL1, MIRLET7F2, MIR2355, JMJD8, MIR329-2, MIR188, KLF2, MIR126, MIR193A, LOXL2, CARD10, MIR24-1, MIA3, MIR20A, MIR495, PTK2B, MIR22, GHSR, MIR16-1, GHRL, MIR361, OTULIN, MIR494, MIR29C, HMOX1, PTGS2, NRP1, HDAC5, VEGFB, CDH13, AKT1, MIR1-1, FOXC2, MIR19B2, KLF4, CDC42, MIR15A, SYNJ2BP, ITGB1, EPHA2 | 100 |
| 28 | GO:0043535 | regulation of blood vessel endothelial cell migration | PRKD1, MIR27B, MIR212, MIR499A, MIR196A2, MIR129-1, MIR342, NUS1, MIR22, MECP2, PDCD10, FGFBP1, HDAC9, MMRN2, TBXA2R, MIR16-2, MIR23B, MIR92A1, MIR16-1, SH3BP1, MIR497, MIR361, FGFR1, MIR320A, MIR200A, MIR494, JCAD, MIR29C, HMOX1, MIR329-1, MIR503, MIR92A2, MIR424, PTGS2, MIR200B, MIR15B, MIR487B, AKT1, MIR31, NF1, HRG, NOTCH1, MIR19B2, PDPK1, FGF2, MIR101-2, MIR410, KLF4, MIR19B1, MIRLET7F2, MIR2355, PDGFB, MIR132, MIR329-2, MIR188, ANXA1, MIR939, MIR505, HMGB1, MIR204, MIR126, MIR193A, NOS3, SP1, MIR15A, ANGPT4, SRPX2, MIR137, AGTR2, ANGPT2, MIR495, PRKCA, CARD10, MIRLET7F1, MIR24-1, STARD13, VEGFC, AKT3, MIR20A, RGCC | 100 |
| 28 | GO:0010631 | epithelial cell migration | LGMN, CXCL13, EGR3, PDCD10, S100P, LGALS8, MIR16-2, MIR23B, SH3BP1, TEK, MIR497, FGFR1, MIR320A, EVL, EFNB2, MIR424, KANK1, MIR15B, MIR31, MIR410, MIR132, ANXA1, HMGB1, EPHB4, ANGPT4, ANGPT2, MIRLET7F1, STARD13, MIR196A2, ADGRA2, SOX18, MIR296, TGFB2, JCAD, MIR21, MIR503, TNFSF12, HDAC6, HRG, FGF4, PDPK1, FOXP1, MIR19B1, PTEN, BMP10, SP1, PAXIP1, MTOR, MIR137, SEMA4A, RAB25, DOCK5, ANXA3, AKT3, CAPN7, RGCC, MIR212, MIR27B, MIR342, KRIT1, NUS1, MMRN2, TBXA2R, MIR92A1, PRSS3, MIR329-1, PROX1, CALR, PRKX, MIR200B, MIR487B, SEMA5A, MIR101-2, KRT2, ADIPOR1, MIR329-2, MIR188, NOS3, GATA3, LOXL2, PPM1F, CARD10, STC1, MIR24-1, MIR20A, MIR495, PRKD1, ZEB2, MIR499A, PTK2B, MIR22, CCR6, SERPINF1, PFN2, MIR16-1, PTPRR, MYH9, MIR494, MIR200A, MIR29C, HMOX1, ADAM9, PTGS2, HDAC5, ID1, AKT1, MAP4K4, KLF4, SRC, DUSP10, SVBP, SMOC2, EPB41L4B, MIR939, CORO1B, MIR15A, HBEGF, BCAS3, EPPK1 | 80 |
| 28 | GO:0090049 | regulation of cell migration involved in sprouting angiogenesis | MIR27B, MIR196A2, FGFBP1, MIR22, MMRN2, PDCD10, HDAC9, TBXA2R, MIR16-2, MIR23B, MIR296, MIR16-1, MIR497, MIR361, MIR494, JCAD, MEOX2, MIR29C, ABL1, MIR329-1, MIR503, MIR221, MIR424, PTGS2, HDAC5, MIR487B, NOTCH1, MIR31, FOXC2, MIR19B2, FGF2, MIR101-2, MIR410, KLF4, MIR19B1, MIR2355, MIRLET7F2, MIR132, MIR329-2, MIR188, ANXA1, MIR126, MAP2K5, MIR193A, MIR15A, SRPX2, MIR495, MIRLET7F1, STARD13, AKT3, MIR20A | 80 |
| 28 | GO:0043542 | endothelial cell migration | LGMN, CLEC14A, EGR3, PDCD10, S100P, LGALS8, MIR16-2, MIR23B, SH3BP1, TEK, FGFR1, MIR320A, EFNB2, MIR92A2, MIR424, MIR15B, MIR31, MIR410, ANXA1, HMGB1, EPHB4, ANGPT4, ANGPT2, MIRLET7F1, STARD13, ADGRA2, SOX18, JCAD, MIR21, MIR503, FGF16, TNFSF12, HRG, NF1, FGF4, PDPK1, FOXP1, MIR19B1, PTEN, BMP10, PAXIP1, SRPX2, MIR137, SEMA4A, AKT3, RGCC, MIR27B, MIR129-1, MIR342, KRIT1, MMRN2, NUS1, TBXA2R, MIR92A1, PRSS3, MIR329-1, CALR, PRKX, MIR200B, MIR487B, NOTCH1, SEMA5A, MIR101-2, MIRLET7F2, MIR329-2, MIR188, MIR505, MIR126, NOS3, MIR193A, GATA3, LOXL2, MIR29A, CARD10, STC1, MIR24-1, MIR20A, MIR495, PRKD1, MIR499A, PTK2B, MIR22, AMOT, SERPINF1, MIR16-1, MIR361, MYH9, MIR494, MIR200A, HMOX1, PTGS2, HDAC5, ID1, AKT1, MIR19B2, MET, KLF4, SASH1, PDGFB, SVBP, SMOC2, CORO1B, MIR15A, AGTR2, BCAS3 | 80 |
| 28 | GO:0050678 | regulation of epithelial cell proliferation | SHH, PDCD10, MIR16-2, MIR23B, AGTR1, CDKN2B, TEK, MIR497, MIR29B1, MIR34A, EFNB2, MIR424, MIR15B, BCL11B, DLX5, CAV1, GLUL, MIR410, KLF9, MIR132, HMGB1, LRG1, PRKCA, PPP1R16B, HES5, ATF2, CPB2, CDC73, NLRC3, CASK, SIRT6, MIR30B, LEP, TGFB2, KRT4, JCAD, MIR21, MIR503, WNT2, TNFSF12, BMPR1A, ATP5IF1, MST1L, RICTOR, KDM5B, IFT74, HOXA5, BAD, STAT1, PDPK1, PTEN, FOXP2, STXBP4, SP1, FLT1, MTOR, EYA1, AKT3, INTU, RGCC, MIR27B, MIR129-1, MIR342, KRIT1, B4GALT1, NRARP, IL10, HMGN1, MIR329-1, PROX1, HES1, SULF1, GDF5, MIR487B, NOTCH1, CDK6, HTR2B, SEMA5A, IFT80, MIR101-2, PTCH1, ITGA4, SMO, MIR329-2, TBX1, ERRFI1, MIR126, TWIST1, TGFA, GATA3, DLX6, LAMC1, CAV2, MIR29A, MIR24-1, WDR13, MIR495, NKX3-1, PRKD1, MIR499A, PEX2, MIR22, SERPINF1, GHSR, MIR16-1, WNT3A, GHRL, MIR494, MIR29C, RB1, NOG, EGFL7, NFIB, PTN, VEGFB, ID1, AKT1, VASH2, DUSP10, DLG1, MIR29B2, MIR135B, MAGED1, MIR15A, A4GNT, FMC1, LACRT, CCND1, EPPK1 | 80 |
| 28 | GO:1905562 | regulation of vascular endothelial cell proliferation | APLN, MIR499A, MIR129-1, GHSR, SIRT6, MIR30B, GHRL, MIR34A, MIR20B, MIR329-1, MIR503, MIR21, MIR424, MIR98, MIR15B, MIR487B, PDPK1, FGF2, ITGA4, MIR329-2, HMGB1, MIR126, FLT1, MIR29A, MIR24-1, AKT3, MIR495 | 100 |

---


---


---

# Cluster 63

| Cluster | Term | Name |
| --- | --- | --- |
| 63 | HP:0000356 | Abnormality of the outer ear |
| 63 | HP:0000369 | Low-set ears |
| 63 | HP:0000581 | Blepharophimosis |
| 63 | HP:0004209 | Clinodactyly of the 5th finger |
| 63 | HP:0005280 | Depressed nasal bridge |

| Cluster | Term | Name | Genes | Percentage\_of\_nodes\_with\_funsys |
| --- | --- | --- | --- | --- |
| 63 | GO:0021544 | subpallium development | SHANK3, JAKMIP1, DRD1, GLI3, OGDH, DLX1, HPRT1, SLC7A11, ALDH1A3, BBS4, INHBA, SLITRK5, FOXP2, BCL11B, DLX2, CNTNAP2 | 100 |

---


---


---

# Cluster 70

| Cluster | Term | Name |
| --- | --- | --- |
| 70 | HP:0000085 | Horseshoe kidney |
| 70 | HP:0000368 | Low-set, posteriorly rotated ears |
| 70 | HP:0002020 | Gastroesophageal reflux |
| 70 | HP:0006008 | Unilateral brachydactyly |
| 70 | HP:0007369 | Atrophy/Degeneration affecting the cerebrum |

| Cluster | Term | Name | Genes | Percentage\_of\_nodes\_with\_funsys |
| --- | --- | --- | --- | --- |
| 70 | GO:0001941 | postsynaptic membrane organization | SHANK3, MESD, MUSK, GDNF, NRXN1, LRP4, NLGN2, SLC7A11, RAPSN, PTN, RER1, RELN, FZD9, DLG4, DVL1, LHFPL4, CDH2, MAGI2, CHRNB1 | 100 |

---


---


---

# Cluster 87

| Cluster | Term | Name |
| --- | --- | --- |
| 87 | HP:0000369 | Low-set ears |
| 87 | HP:0000048 | Bifid scrotum |
| 87 | HP:0000277 | Abnormality of the mandible |
| 87 | HP:0000545 | Myopia |
| 87 | HP:0001363 | Craniosynostosis |

| Cluster | Term | Name | Genes | Percentage\_of\_nodes\_with\_funsys |
| --- | --- | --- | --- | --- |
| 87 | GO:0001937 | negative regulation of endothelial cell proliferation | MIR129-1, MIR342, KRIT1, MIR22, PDCD10, NR2F2, MIR16-2, MIR16-1, MIR30B, GHRL, MIR497, MIR361, MIR494, MIR34A, MIR20B, MIR329-1, MIR503, MIR21, MIR424, TNMD, ATP5IF1, MIR98, MIR15B, MIR487B, STAT1, CAV1, MIR410, MIR329-2, MIR126, FLT1, MIR15A, CAV2, SYNJ2BP, MIR24-1, MIR495, RGCC | 80 |
| 87 | GO:1903670 | regulation of sprouting angiogenesis | FGFBP1, PDCD10, BMPER, MIR16-2, MIR23B, AGTR1, MIR497, MIR34A, MIR92A2, MIR424, MIR15B, MIR31, SMAD1, FGF2, MIR222, MIR410, MIR7-2, MIR132, ANXA1, HMGB1, MAP2K5, MIRLET7F1, STARD13, PPP1R16B, MIR196A2, HDAC9, EPN2, MIR296, MIR30B, MIR221, JCAD, E2F2, MIR503, PDPK1, CEMIP2, MIR19B1, SRPX2, PKM, AKT3, MIR377, MIR27B, MIR342, MMRN2, MIR150, ITGA5, TBXA2R, MIR92A1, IL10, ABL1, MIR329-1, MIR1224, MIR18A, MIR487B, NOTCH1, MIR17, MIR19A, MIR101-2, DLL1, MIRLET7F2, JMJD8, MIR2355, MIR7-3, MIR329-2, MIR188, KLF2, MIR126, MIR193A, CARD10, MIR24-1, MIR20A, MIR495, MIR22, GHSR, MIR16-1, GHRL, MIR361, MIR494, MEOX2, MIR29C, HMOX1, PTGS2, NRP1, HDAC5, MIR1-1, FOXC2, MIR19B2, KLF4, MIR15A, SYNJ2BP | 80 |
| 87 | GO:1903671 | negative regulation of sprouting angiogenesis | MIR196A2, MIR342, MIR22, MMRN2, PDCD10, CREB3L1, EPN2, TBXA2R, MIR92A1, MIR16-2, MIR16-1, MIR23B, MIR497, MIR361, MIR320A, MIR494, MIR34A, MIR221, MIR29C, E2F2, MIR329-1, MIR503, MIR92A2, MIR424, MEOX2, MIR18A, MIR15B, HDAC5, MIR487B, NOTCH1, MIR17, MIR19B2, MIR19A, MIR222, MIR7-2, MIR410, KLF4, MIR19B1, MIR2355, MIR7-3, MIR329-2, MIR188, MAP2K5, MIR15A, MIR495, CARD10, SYNJ2BP, STARD13, MIR24-1, MIR377, MIR20A | 100 |

---


---


---

# Cluster 34

| Cluster | Term | Name |
| --- | --- | --- |
| 34 | HP:0000316 | Hypertelorism |
| 34 | HP:0000347 | Micrognathia |
| 34 | HP:0000369 | Low-set ears |
| 34 | HP:0000431 | Wide nasal bridge |
| 34 | HP:0000954 | Single transverse palmar crease |
| 34 | HP:0004209 | Clinodactyly of the 5th finger |
| 34 | HP:0000581 | Blepharophimosis |

| Cluster | Term | Name | Genes | Percentage\_of\_nodes\_with\_funsys |
| --- | --- | --- | --- | --- |
| 34 | GO:0072376 | protein activation cascade | SERPINC1, IGLL1, IGHV3-33, IGHG4, IGLC6, IGLV2-23, TFPI, CR1, IGHV4-28, IGHV4-39, C8G, IGHV5-51, IGHV3-7, KRT1, F9, IGLV3-25, CFHR4, IGHV1-45, IGHE, TRBC2, CFHR5, IGHV3-48, IGLV2-8, IGHG2, IGHD, IGLV2-11, IGHV3-43, IGHV1-58, APCS, C1QBP, IGLV3-1, CFHR2, IGHV2-70, IGHM, GP5, C6, C4BPB, IGLV1-51, CPB2, IGHV3-49, SCARA3, CRP, IGHV4-61, IGHV4-59, CFHR1, C4BPA, FCN1, KLKB1, C1QC, IGHV3-30, IGLC1, IGHV1-3, F7, IGHV4-4, F12, IGLV7-43, IGLV3-27, C1QB, IGHV2-26, IGLV3-19, CR2, IGHV3-15, IGLV1-40, C9, CFH, IGHV3-21, FBLN1, RGCC, IGHV3-53, CFD, F8, CPN2, FCN2, IGHG3, FGA, IGLC2, CD46, IGHA2, MASP2, F13B, IGHV3-11, SUSD4, IGLC7, CD55, IGHV3-66, IGHV3-73, IGHV3-13, F10, FGG, IGHV1-18, COL20A1, MASP1, IGLV2-14, KNG1, IGLL5, IGHV1OR15-1, IGLV1-47, FCN3, IGHV3-72, A2M, IGLV3-21, IGHV1-69, IGHV3-74, IGHV6-1, GP1BB, IGHV3-64, FGB, COLEC10, GP1BA, VSIG4, IGLV1-44, IGLC3, IGHA1, C1QA, IGHG1, IGLV6-57, C5, IGHV4-34, F11, C7, IGHV3-20, IGHV1-24, IGHV2-5, CD19, CLU, IGHV3-23 | 85.71429 |
| 34 | GO:1905651 | regulation of artery morphogenesis | MIR494, EFNB2, MIR329-1, MIR153-2, MIR29B2, MIR329-2, MIR487B, NOTCH1, MIR205, AKT3, MIR495, MIR29B1 | 71.42857 |
| 34 | GO:0002455 | humoral immune response mediated by circulating immunoglobulin | IGLL1, IGHV3-33, IGHG4, IGLC6, IGLV2-23, CR1, IGHV4-28, IGHV4-39, C8G, IGHV5-51, IGHV3-7, IGLV3-25, EXO1, IGHV1-45, IGHE, TRBC2, IGHV3-48, IGLV2-8, IGHG2, IGHD, IGLV2-11, IGHV3-43, IGHV1-58, APCS, C1QBP, IGLV3-1, IGHV2-70, IGHM, FCGR2B, C6, C4BPB, IGLV1-51, IGHV3-49, CRP, IGHV4-61, IGHV4-59, C4BPA, C1QC, IGHV3-30, IGLC1, IGHV1-3, IGHV4-4, IGLV7-43, IGLV3-27, C1QB, IGHV2-26, IGLV3-19, CR2, IGHV3-15, IGLV1-40, C9, IGHV3-21, IGHV3-53, IGHG3, CD46, IGLC2, IGHA2, MASP2, IGHV3-11, SUSD4, IGLC7, CD55, IGHV3-66, IGHV3-73, IGHV3-13, IGHV1-18, PTPRC, IGLV2-14, IGLL5, IGHV1OR15-1, IGLV1-47, IGHV3-72, IGLV3-21, IGHV1-69, IGHV3-74, IGHV6-1, IGHV3-64, IGLV1-44, IGLC3, IGHA1, C1QA, IGHG1, IGLV6-57, C5, IGHV4-34, C7, IGHV3-20, IGHV1-24, IGHV2-5, CLU, IGHV3-23 | 85.71429 |
| 34 | GO:0006910 | phagocytosis, recognition | IGHV3-53, IGLL1, IGHV3-33, IGHV1-69, IGHV4-61, IGHG4, IGLC6, IGHV4-59, FCN2, PEAR1, IGHV4-28, IGHV4-39, ADGRB1, IGHG3, IGHV5-51, IGHV3-7, MEGF10, CD36, IGLC2, FCN1, IGHA2, IGHV3-74, IGHV6-1, IGHV3-11, IGHV3-30, IGHE, IGHV1-45, IGHV3-64, TRBC2, IGLC7, IGLC1, IGHV3-48, IGHV3-66, IGHV3-73, MFGE8, IGHG2, IGHD, IGLC3, IGHA1, IGHV1-3, IGHV3-43, IGHG1, IGHV4-4, COLEC12, CLEC7A, IGHV1-58, IGHV3-13, IGHV4-34, IGHV1-18, IGHV2-26, IGHV2-70, IGHM, IGHV3-20, IGHV1-24, IGLL5, IGHV2-5, IGHV1OR15-1, IGHV3-15, FCN3, IGHV3-21, IGHV3-72, IGHV3-23, IGHV3-49 | 85.71429 |
| 34 | GO:0030449 | regulation of complement activation | IGHV3-53, IGLV3-21, IGHV3-33, IGHV1-69, IGHG4, IGLC6, IGLV2-23, CPN2, CR1, IGHV4-59, IGHV4-39, IGHG3, CFHR1, C8G, IGHV3-7, CD46, C4BPA, CFHR4, IGLV3-25, SUSD4, IGHV3-11, C1QC, IGHV3-30, CFHR5, IGLC7, CD55, IGHV3-48, IGLC1, IGLV2-8, IGLV1-44, IGHG2, IGLV2-11, C1QA, IGHG1, IGLV6-57, IGLV7-43, C5, IGHV3-13, IGLV3-27, IGHV4-34, IGLV3-1, C1QB, CFHR2, IGLV3-19, IGHV2-70, C7, C1QBP, IGLV2-14, CR2, C6, C4BPB, IGHV2-5, IGHV1OR15-1, IGLV1-47, IGLV1-40, CD19, C9, CFH, CLU, IGLV1-51, IGHV3-23, CPB2, A2M | 85.71429 |
| 34 | GO:0019724 | B cell mediated immunity | IGLL1, IGHV3-33, CD40LG, IGHG4, IGLC6, IGLV2-23, FOXP3, CR1, IGHV4-28, IGHV4-39, C8G, IGHV5-51, IGHV3-7, IGLV3-25, EXO1, MLH1, IGHV1-45, IGHE, TNFSF4, TRBC2, IGHV3-48, IGLV2-8, IGHG2, IGHD, IGLV2-11, IGHV3-43, BCL6, IL13RA2, IGHV1-58, APCS, C1QBP, IGLV3-1, IGHV2-70, IGHM, TNFSF13, FCGR2B, C6, C4BPB, XCL1, MAD2L2, IGLV1-51, IGHV3-49, CRP, SHLD2, FCER1G, IGHV4-61, THOC1, IL4, INPP5D, IGHV4-59, C4BPA, C1QC, IGHV3-30, RIF1, IGLC1, APLF, IGHV3-23, IGHV1-3, LIG4, IGHV4-4, IGLV7-43, IGLV3-27, C1QB, IGHV2-26, IGLV3-19, PAXIP1, CR2, HSPD1, IGHV3-15, IGLV1-40, CD28, C9, IGHV3-21, IGHV3-53, IGHG3, IGLC2, CD46, IGHA2, IL2, MASP2, IGHV3-11, SUSD4, IL10, IGLC7, CD55, IGHV3-66, IGHV3-73, BTK, IGHV3-13, GAPT, IGHV1-18, PTPRC, IGLV2-14, IGLL5, IGHV1OR15-1, IGLV1-47, IGHV3-72, ATAD5, NSD2, IGLV3-21, MSH6, IGHV1-69, CCR6, NBN, BCL10, RNF168, IGHV3-74, IGHV6-1, IGHV3-64, MSH2, NDFIP1, IL4R, IGLV1-44, IGLC3, IGHA1, C1QA, IGHG1, IGLV6-57, CD226, C5, IGHV4-34, C7, CLCF1, IGHV3-20, IGHV1-24, IGHV2-5, CLU, SLA2, TFRC | 85.71429 |
| 34 | GO:0038096 | Fc-gamma receptor signaling pathway involved in phagocytosis | IGHV3-33, IGHG4, IGLC6, IGLV2-23, DOCK1, IGHV4-39, VAV2, IGHV3-7, PLCG2, IGLV3-25, WASL, IGHV3-48, IGLV2-8, IGHG2, IGLV2-11, ACTB, CD247, PLA2G6, PRKCE, IGLV3-1, IGHV2-70, FCGR2B, MAPK1, CRK, WASF2, GRB2, ARPC5, IGLV1-51, ARPC4, CD47, RAPGEF1, RAP1A, IGHV4-59, CYFIP1, IGHV3-30, HSP90AA1, MYO1C, ARPC1B, IGLC1, IGLV7-43, IGLV3-27, IGLV3-19, MYO1G, RAC1, IGLV1-40, FCGR1A, YES1, IGHV3-53, WAS, FGR, HCK, ACTR2, IGHG3, FCGR2A, PTPRJ, ABL1, IGHV3-11, IGLC7, ELMO1, PTK2, IGHV3-13, PTPRC, WIPF1, IGLV2-14, IGHV1OR15-1, IGLV1-47, IGLV3-21, BRK1, ABI1, IGHV1-69, NCKAP1L, PIK3CB, MAPK3, MYO10, NCK1, ARPC1A, IGLV1-44, IGHG1, IGLV6-57, SRC, IGHV4-34, WIPF3, FCGR3A, IGHV2-5, NCKAP1, FYN, LIMK1, IGHV3-23 | 85.71429 |
| 34 | GO:0050867 | positive regulation of cell activation | CD83, TESPA1, IGLL1, SHH, IGHV3-33, EGR3, CD40LG, SIRPB1, GAS6, IGHG4, IGLC6, FOXP3, CSK, TNFSF11, IGHV4-28, IGHV4-39, TMIGD2, IGHV5-51, IGHV3-7, PAK2, EFNB2, AP3D1, MLH1, MIR92A2, IGHV1-45, IGHE, CD1D, TRBC2, TNFSF4, PCID2, IGHV3-48, CTLA4, IGHG2, IGHD, CAV1, DOCK8, IGHV3-43, TNFRSF14, CLECL1, DNAJA3, BCL6, IGHV1-58, CARD11, ANXA1, HMGB1, IGHV2-70, IGHM, TNFSF13, GRB2, IL7, TAC1, XCL1, VCAM1, PTAFR, ICOS, MAD2L2, SLC39A10, IGHV3-49, SHLD2, PYCARD, CD274, ITPKB, FCER1G, CD276, CD47, PAK3, IGHV4-61, DPP4, IL4, INPP5D, IGHV4-59, KLRK1, MIR30B, LEP, MIR21, SART1, IL6, IGHV3-30, GLI3, GPR183, RIF1, PPP2R3C, IL6ST, FADD, IGLC1, IGHV3-23, BAD, ITGB2, CRLF2, PDPK1, IGHV1-3, FLT3LG, TNFRSF13C, LBP, FGF10, IGHV4-4, ADAM8, RIPK2, IGHV2-26, NLRP3, IRS2, PAXIP1, IL1RL1, HSPD1, CBFB, SIRPA, HHLA2, RAC1, STXBP1, IGHV3-15, CD28, IGHV3-21, LAMP1, JAK2, YES1, CORO1A, MAP3K8, IGHV3-53, PDCD1LG2, IL5, FGR, IL23R, MIR92A1, ADORA2B, LILRB1, MIF, GPAM, IGHG3, IGLC2, CD46, NFATC2, IGHA2, IL2, IL10, IGHV3-11, EBI3, SOCS5, ZP4, HES1, NKAP, IGLC7, CD55, STAP1, MALT1, IGHV3-66, IGHV3-73, TNIP2, LGALS1, PRKCZ, SELP, BCL2, BTK, IL21, MEF2C, PDPN, ATP11C, AP1G1, IL2RA, IGHV3-13, IGHV1-18, PTPRC, GATA3, HSPH1, SPTA1, IL1RL2, IGLL5, FCRL3, IGHV1OR15-1, CD86, IGHV3-72, SOX15, HLX, ATAD5, NSD2, MSH6, NPPA, SASH3, IGHV1-69, EPO, CD38, BCL10, TNFRSF4, PLEK, NCKAP1L, WNT3A, IL12B, IL12A, GRAP2, DMTN, EFNB3, IGHV3-74, IGHV6-1, SOCS1, BST1, IGHV3-64, MSH2, NCK1, RASAL3, ITGAM, IL15, IL4R, KLRC4-KLRK1, AKT1, CD80, IGLC3, IGHA1, CCDC88B, PDCD1, XBP1, LILRB4, IGHG1, SRC, DUSP10, CD226, IGHV4-34, IL33, CLCF1, STX4, TNFSF13B, IGHV3-20, IGHV1-24, LILRB2, F2RL1, IGHV2-5, SIRPG, PRKCQ, NR4A3, FYN, BTLA, PELI1, TICAM1, IL13, TFRC, TIRAP | 85.71429 |
| 34 | GO:0006958 | complement activation, classical pathway | IGLL1, IGHV3-33, IGHG4, IGLC6, IGLV2-23, CR1, IGHV4-28, IGHV4-39, C8G, IGHV5-51, IGHV3-7, IGLV3-25, IGHV1-45, IGHE, TRBC2, IGHV3-48, IGLV2-8, IGHG2, IGHD, IGLV2-11, IGHV3-43, IGHV1-58, APCS, C1QBP, IGLV3-1, IGHV2-70, IGHM, C6, C4BPB, IGLV1-51, IGHV3-49, CRP, IGHV4-61, IGHV4-59, C4BPA, C1QC, IGHV3-30, IGLC1, IGHV1-3, IGHV4-4, IGLV7-43, IGLV3-27, C1QB, IGHV2-26, IGLV3-19, CR2, IGHV3-15, IGLV1-40, C9, IGHV3-21, IGHV3-53, IGHG3, IGLC2, CD46, IGHA2, MASP2, IGHV3-11, SUSD4, IGLC7, CD55, IGHV3-66, IGHV3-73, IGHV3-13, IGHV1-18, IGLV2-14, IGLL5, IGHV1OR15-1, IGLV1-47, IGHV3-72, IGLV3-21, IGHV1-69, IGHV3-74, IGHV6-1, IGHV3-64, IGLV1-44, IGLC3, IGHA1, C1QA, IGHG1, IGLV6-57, C5, IGHV4-34, C7, IGHV3-20, IGHV1-24, IGHV2-5, CLU, IGHV3-23 | 85.71429 |
| 34 | GO:0050851 | antigen receptor-mediated signaling pathway | TESPA1, IGLL1, IGHV3-33, RAB29, IGHG4, IGLC6, PVRIG, PAG1, FOXP3, CSK, KLHL6, DENND1B, IGHV4-28, IGHV4-39, BLK, DUSP22, IGHV5-51, IGHV3-7, PLCG2, PAK2, IKBKG, IGHV1-45, IGHE, TRBC2, IGHV3-48, CTLA4, HRAS, PTPN2, LCP2, IGHG2, IGHD, NFAM1, RBCK1, IGHV3-43, RELA, CD247, IGHV1-58, PAWR, CARD11, IGHV2-70, IGHM, GCSAM, MAPK1, ELF2, PHPT1, SLC39A10, LAT2, PDE4D, IGHV3-49, CD276, PAK3, IGHV4-61, INPP5D, TXK, IGHV4-59, CMTM3, MNDA, LIME1, TRAT1, BTNL9, PIK3CD, IKBKB, IGHV3-30, IGLC1, PAX5, IGHV3-23, PDPK1, IGHV1-3, IGHV4-4, RIPK2, ERMAP, IGHV2-26, SH2B2, DUSP3, CBFB, HHLA2, IGHV3-15, CD28, PLEKHA1, IGHV3-21, EZR, IGHV3-53, WAS, CACNA1F, BTNL3, LAT, GCSAML, RC3H2, IGHG3, IGLC2, PTPRJ, NFATC2, IGHA2, ABL1, IGHV3-11, FYB1, PLCL2, RC3H1, IGLC7, STAP1, MALT1, IGHV3-66, IGHV3-73, BCL2, STOML2, BTK, MEF2C, PLCG1, IGHV3-13, LGALS3, PDE4B, IGHV1-18, PTPRC, BTNL8, GATA3, IGLL5, FCRL3, IGHV1OR15-1, IGHV3-72, GPS2, IGHV1-69, BTNL10, CD38, BCL10, NCKAP1L, PIK3CB, GRAP2, IGHV3-74, IGHV6-1, IGHV3-64, NCK1, THEMIS2, IGLC3, IGHA1, LILRB4, CD300A, IGHG1, CD226, IGHV4-34, STK11, BAX, TEC, IGHV3-20, IGHV1-24, IGHV2-5, ELF1, CD19, PRKCQ, FYN, RFTN1, BRAF, SLA2, EIF2B5 | 85.71429 |
| 34 | GO:0042113 | B cell activation | POLM, IGLL1, IGHV3-33, CD40LG, IGHG4, IGLC6, FOXP3, HDAC4, IGHV4-28, IGHV4-39, IFNA5, IGHV5-51, IGHV3-7, PLCG2, CHRNA4, EXO1, MLH1, IGHV1-45, IGHE, IFNB1, HHEX, TRBC2, TNFSF4, PCID2, IGHV3-48, CTLA4, PTPN2, IGHG2, IGHD, NFAM1, IGHV3-43, INHBA, BCL6, IGHV1-58, PAWR, CARD11, IFNA14, IRF2BP2, IGHV2-70, IGHM, TNFSF13, FCGR2B, AHR, TNFRSF13B, IL7, VCAM1, MAD2L2, LAT2, SLC39A10, IGHV3-49, SHLD2, IFNA6, IFNA4, IFNA10, IGHV4-61, THOC1, HDAC9, IFNA13, IL4, AKAP17A, INPP5D, IGHV4-59, MNDA, PIK3CD, IFNE, IL6, IGHV3-30, GPR183, RIF1, PPP2R3C, IGLC1, APLF, FZD9, IGHV3-23, BAD, IFNA16, IGHV1-3, GON4L, LIG4, TNFRSF13C, IGHV4-4, FCRL1, IFNA2, EP300, IGHV2-26, IRS2, PAXIP1, CR2, HSPD1, IGHV3-15, ITM2A, CD28, IFNA1, IGHV3-21, ONECUT1, LYL1, IGHV3-53, IL5, FNIP1, CDH17, MIF, IGHG3, CASP8, IGLC2, PTPRJ, NFATC2, IGHA2, IL2, TPD52, IGHV3-11, IL10, ABL1, PLCL2, RC3H1, IGLC7, MALT1, IGHV3-66, IGHV3-73, TNIP2, LGALS1, BCL2, LRRC8A, ZFP36L2, CASP3, DLL1, BTK, ITGA4, IL21, IFNA17, MEF2C, ATP11C, IGHV3-13, GAPT, IGHV1-18, PTPRC, IFNA7, LFNG, IGLL5, FCRL3, IGHV1OR15-1, IGHV3-72, IFNA21, ATAD5, NSD2, GPS2, MSH6, SASH3, PTK2B, IGHV1-69, SLA2, CCR6, NTRK1, CD38, NBN, TNFRSF4, NCKAP1L, WNT3A, SKAP2, RNF168, IGHV3-74, IGHV6-1, BST1, IGHV3-64, MSH2, MFNG, IFNK, NDFIP1, HDAC5, DNAJB9, TCF3, BAK1, KLF6, IFNA8, IGLC3, IGHA1, MIR17HG, SP3, XBP1, ZBTB7A, CD300A, IGHG1, IGHV4-34, TBC1D10C, PKN1, CLCF1, TNFSF13B, BAX, IGHV3-20, IGHV1-24, SFRP1, IGHV2-5, DOCK11, LAX1, PELI1, TICAM1, DCLRE1C, ITGB1, IFNW1, IL13, ZFP36L1, TFRC, TIRAP | 85.71429 |
| 34 | GO:0006909 | phagocytosis | SYT11, IGLL1, IGHV3-33, SIRPB1, GAS6, IGHG4, IGLC6, IGLV2-23, CSK, MSR1, SH3BP1, DOCK1, IGHV4-28, IGHV4-39, IRF8, VAV2, IGHV5-51, IGHV3-7, PLCG2, PEAR1, ELMO3, IGLV3-25, SPON2, MESD, RUBCN, IGHV1-45, IGHE, TRBC2, PRTN3, WASL, IGHV3-48, IGLV2-8, IGHG2, IGHD, IGLV2-11, ACTB, ATG5, AZU1, IGHV3-43, EIF2AK1, CD247, IGHV1-58, PLA2G6, ANXA1, PRKCG, IGLV3-1, HMGB1, TLR2, IGHV2-70, IGHM, FCGR2B, MAPK1, CRK, WASF2, LMAN2, GRB2, ARPC5, C4BPB, ATG3, PPARG, PTX3, IGLV1-51, IGHV3-49, ARPC4, CRP, PYCARD, CD47, FCER1G, NCF4, RAPGEF1, IGHV4-61, RAP1A, ITGAL, IGHV4-59, LEP, CD36, RAB27A, CYFIP1, C4BPA, SFTPD, FCN1, SLAMF1, LYAR, IGHV3-30, HSP90AA1, MYO1C, ARPC1B, RAB14, IGLC1, MERTK, ITGB2, CAMK1D, IGHV1-3, LBP, RACK1, IGHV4-4, COLEC12, ANXA11, CLEC7A, TMEM175, IGLV7-43, ABR, IGLV3-27, PTEN, IGHV2-26, ARHGAP12, IGLV3-19, ADORA1, TM9SF4, MYO1G, SIRPA, RAC1, IGHV3-15, IGLV1-40, LEPR, IGHV3-21, FCGR1A, ANXA3, RHOBTB2, CORO1A, YES1, IGHV3-53, WAS, GSN, FGR, DOCK2, HCK, FCN2, ACTR2, IGHG3, MEGF10, FCGR2A, IGLC2, PTPRJ, IGHA2, ABL1, IGHV3-11, ITGAV, BCR, CALR, IGLC7, STAP1, CDC42SE2, IGHV3-66, IGHV3-73, MIR17, CD14, CYBA, DYSF, ELMO1, PTK2, RAB20, IGHV3-13, IGHV1-18, PTPRC, WIPF1, ADIPOQ, IGLV2-14, IGLL5, IGHV1OR15-1, AHSG, IGLV1-47, CD302, FCN3, IGHV3-72, MIR20A, IGLV3-21, BRK1, ABI1, IGHV1-69, PLD4, NCKAP1L, PIK3CB, ADGRB1, ARHGAP25, MAPK3, MYO10, MYH9, PIP5K1C, IGHV3-74, IGHV6-1, IGHV3-64, NCK1, ARPC1A, ITGAM, ABCA7, MFGE8, IGLV1-44, NR1H3, IGLC3, IGHA1, MET, PRKCE, TGM2, CD300A, IGHG1, IGLV6-57, SRC, ELANE, IGHV4-34, GULP1, WIPF3, IGHV3-20, IGHV1-24, NCF2, XKR8, FCGR3A, F2RL1, IGHV2-5, NCKAP1, SIRPG, FYN, LIMK1, ITGB1, IGHV3-23 | 85.71429 |
| 34 | GO:0002526 | acute inflammatory response | SERPINC1, IGHV3-33, PARK7, IGHG4, IGLC6, IGLV2-23, CR1, TNFSF11, IGHV4-39, C8G, IGHV3-7, IGLV3-25, CFHR4, MIR92A2, TNFSF4, GSTP1, CFHR5, IGHV3-48, IGLV2-8, IGHG2, IGLV2-11, PIK3CG, EIF2AK1, APCS, C1QBP, IGLV3-1, CFHR2, OSMR, IGHV2-70, FCGR2B, C6, TAC1, C4BPB, VCAM1, PPARG, IGLV1-51, OPRM1, CPB2, CRP, FCER1G, IGHV4-59, CFHR1, NUPR1, C4BPA, KLKB1, TNFRSF11A, IGHV3-30, C1QC, IL6, IL6ST, IGLC1, LBP, SERPINF2, F12, ADAM8, IGLV7-43, IGLV3-27, RHBDD3, C1QB, ADORA1, IGLV3-19, NLRP3, NPFF, CR2, IGLV1-40, C9, CFH, IL20RB, IGHV3-53, SELENOS, PTGES, F8, B4GALT1, PLSCR1, CPN2, MIR92A1, CTNNBIP1, IGHG3, TFR2, CD46, IGHV3-11, SUSD4, UGT1A1, IGLC7, CD55, OSM, CREB3L3, DNASE1, BTK, ADCYAP1, FN1, IGHV3-13, GATA3, IGLV2-14, HP, IGHV1OR15-1, AHSG, IGLV1-47, TRPV1, DNASE1L3, A2M, IGLV3-21, ASS1, IGHV1-69, EPO, EDNRB, ASH1L, PTGS2, PTGER3, IGLV1-44, C1QA, IGHG1, IGLV6-57, ELANE, C5, IL31RA, APOA2, IGHV4-34, HPR, C7, OGG1, CEBPB, APOL2, IGHV2-5, CD19, CLU, IGHV3-23 | 71.42857 |
| 34 | GO:0038094 | Fc-gamma receptor signaling pathway | IGHV3-33, IGHG4, IGLC6, IGLV2-23, DOCK1, IGHV4-39, VAV2, IGHV3-7, PLCG2, IGLV3-25, WASL, IGHV3-48, IGLV2-8, IGHG2, IGLV2-11, ACTB, CD247, PLA2G6, PRKCE, IGLV3-1, IGHV2-70, FCGR2B, MAPK1, CRK, WASF2, GRB2, ARPC5, IGLV1-51, ARPC4, CD47, FCER1G, RAPGEF1, RAP1A, IGHV4-59, CYFIP1, IGHV3-30, HSP90AA1, MYO1C, ARPC1B, IGLC1, IGLV7-43, IGLV3-27, IGLV3-19, MYO1G, RAC1, IGLV1-40, FCGR1A, YES1, IGHV3-53, WAS, FGR, HCK, ACTR2, IGHG3, FCGR2A, PTPRJ, ABL1, IGHV3-11, IGLC7, ELMO1, PTK2, IGHV3-13, PTPRC, WIPF1, IGLV2-14, IGHV1OR15-1, IGLV1-47, IGLV3-21, BRK1, ABI1, IGHV1-69, NCKAP1L, PIK3CB, MAPK3, MYO10, NCK1, ARPC1A, IGLV1-44, IGHG1, IGLV6-57, SRC, IGHV4-34, WIPF3, FCGR3A, IGHV2-5, NCKAP1, FYN, LIMK1, IGHV3-23 | 85.71429 |
| 34 | GO:0016485 | protein processing | SEC11C, CTSH, LGMN, SHH, IGHV3-33, IGHG4, IGLC6, IGLV2-23, KLK13, CR1, IGHV4-39, C8G, F9, IGHV3-7, MELTF, ASPH, IGLV3-25, CFHR4, IMMP1L, ASPRV1, PRSS37, CFHR5, IGHV3-48, IGLV2-8, IGHG2, KLK2, IGLV2-11, YME1L1, USP17L2, MME, HM13, IGLV3-1, KLK3, CFHR2, IGHV2-70, C6, C4BPB, PISD, PCSK4, CPXM1, ACP4, IGLV1-51, CPB2, PYCARD, CLN5, IMMP2L, CGB3, CTSE, CPXM2, CNTN2, IGHV4-59, CFHR1, C4BPA, DDI2, ADAMTS2, KLKB1, C1QC, IGHV3-30, GLI3, KLK1, ERO1B, FADD, IGLC1, XPNPEP3, CPE, BAD, PMPCA, F7, SERPINF2, ANXA2P2, F12, ADAM8, IGLV7-43, IGLV3-27, RIPK2, C1QB, IGLV3-19, SCG5, CR2, PLAU, C9orf3, XIAP, PCSK6, IGLV1-40, REN, C9, PLGRKT, SPCS3, CFH, TMEM59, IGHV3-53, GSN, MMP16, CPN2, PSEN2, PRSS3, SEC11B, IGHG3, FGA, CD46, IGHV3-11, SUSD4, ENO1, PGK1, IGLC7, CD55, PCSK1N, TIMM17A, CASP3, PTCH1, STOML2, CAPN2, PCSK5, NCSTN, CARD8, PCSK9, PARP1, IGHV3-13, FGG, FXN, IGLV2-14, CTSZ, CLN3, CUZD1, PMPCB, IGHV1OR15-1, IFI16, SERPINE2, IGLV1-47, CASP2, LHB, AEBP1, ANGPTL8, A2M, IGLV3-21, IGHV1-69, KLK6, TBC1D10A, ADAMTS13, MYH9, CPZ, FGB, SERPINE1, NLRP7, GRIN2A, AFG3L2, ECE2, CPA3, BIRC7, BACE2, BAK1, IGLV1-44, BCL2L12, C1QA, IGHG1, IGLV6-57, SRC, C5, IGHV4-34, F11, C7, IL1R2, PRSS12, IGHV2-5, CD19, CLU, TNP2, IGHV3-23, CCBE1 | 71.42857 |
| 34 | GO:1903317 | regulation of protein maturation | IGHV3-33, IGHG4, IGLC6, IGLV2-23, CR1, IGHV4-39, C8G, MELTF, IGHV3-7, IGLV3-25, CFHR4, PRSS37, CFHR5, IGHV3-48, IGLV2-8, IGHG2, IGLV2-11, USP17L2, NKD2, NOL3, C1QBP, IGLV3-1, CFHR2, IGHV2-70, C6, C4BPB, ACP4, IGLV1-51, CPB2, PRKACB, SOX4, CNTN2, IGHV4-59, CFHR1, C4BPA, KLKB1, C1QC, IGHV3-30, IGLC1, SERPINF2, ANXA2P2, F12, ADAM8, IGLV7-43, IGLV3-27, C1QB, IGLV3-19, CR2, XIAP, IGLV1-40, C9, PLGRKT, CFH, TMEM59, IGHV3-53, GSN, CPN2, IGHG3, TFR2, CD46, IGHV3-11, SUSD4, ENO1, IGLC7, CD55, TIMM17A, CARD8, IGHV3-13, IGLV2-14, CTSZ, IGHV1OR15-1, SERPINE2, IGLV1-47, ANGPTL8, A2M, IGLV3-21, IGHV1-69, MYH9, SERPINE1, NLRP7, BIRC7, IGLV1-44, BCL2L12, C1QA, IGHG1, IGLV6-57, SRC, C5, IGHV4-34, C7, IL1R2, IGHV2-5, CD19, CLU, TNP2, IGHV3-23, CCBE1, GLG1 | 85.71429 |
| 34 | GO:0050853 | B cell receptor signaling pathway | IGLL1, IGHV3-33, IGHG4, IGLC6, KLHL6, BLK, IGHV4-28, IGHV4-39, IGHV5-51, IGHV3-7, PLCG2, IGHV1-45, IGHE, TRBC2, IGHV3-48, CTLA4, IGHG2, IGHD, NFAM1, IGHV3-43, IGHV1-58, IGHV2-70, IGHM, GCSAM, MAPK1, ELF2, SLC39A10, LAT2, IGHV3-49, IGHV4-61, IGHV4-59, CMTM3, MNDA, LIME1, PIK3CD, IGHV3-30, IGLC1, PAX5, IGHV1-3, IGHV4-4, IGHV2-26, CBFB, IGHV3-15, PLEKHA1, IGHV3-21, IGHV3-53, GCSAML, IGHG3, IGLC2, NFATC2, IGHA2, ABL1, IGHV3-11, PLCL2, IGLC7, STAP1, IGHV3-66, IGHV3-73, BCL2, BTK, MEF2C, IGHV3-13, IGHV1-18, PTPRC, IGLL5, FCRL3, IGHV1OR15-1, IGHV3-72, GPS2, IGHV1-69, CD38, NCKAP1L, IGHV3-74, IGHV6-1, IGHV3-64, IGLC3, IGHA1, CD300A, IGHG1, IGHV4-34, BAX, TEC, IGHV3-20, IGHV1-24, IGHV2-5, ELF1, CD19, RFTN1, IGHV3-23 | 85.71429 |
| 34 | GO:0016064 | immunoglobulin mediated immune response | IGLL1, IGHV3-33, CD40LG, IGHG4, IGLC6, IGLV2-23, FOXP3, CR1, IGHV4-28, IGHV4-39, C8G, IGHV5-51, IGHV3-7, IGLV3-25, EXO1, MLH1, IGHV1-45, IGHE, TNFSF4, TRBC2, IGHV3-48, IGLV2-8, IGHG2, IGHD, IGLV2-11, IGHV3-43, BCL6, IL13RA2, IGHV1-58, APCS, C1QBP, IGLV3-1, IGHV2-70, IGHM, TNFSF13, FCGR2B, C6, C4BPB, XCL1, MAD2L2, IGLV1-51, IGHV3-49, CRP, SHLD2, FCER1G, IGHV4-61, THOC1, IL4, INPP5D, IGHV4-59, C4BPA, C1QC, IGHV3-30, RIF1, IGLC1, APLF, IGHV1-3, LIG4, IGHV4-4, IGLV7-43, IGLV3-27, C1QB, IGHV2-26, IGLV3-19, PAXIP1, CR2, HSPD1, IGHV3-15, IGLV1-40, CD28, C9, IGHV3-21, IGHV3-53, IGHG3, IGLC2, CD46, IGHA2, IL2, MASP2, IGHV3-11, SUSD4, IL10, IGLC7, CD55, IGHV3-66, IGHV3-73, BTK, IGHV3-13, GAPT, IGHV1-18, PTPRC, IGLV2-14, IGLL5, IGHV1OR15-1, IGLV1-47, IGHV3-72, ATAD5, NSD2, IGLV3-21, MSH6, IGHV1-69, CCR6, NBN, BCL10, RNF168, IGHV3-74, IGHV6-1, IGHV3-64, MSH2, NDFIP1, IL4R, IGLV1-44, IGLC3, IGHA1, C1QA, IGHG1, IGLV6-57, CD226, C5, IGHV4-34, C7, CLCF1, IGHV3-20, IGHV1-24, IGHV2-5, CLU, IGHV3-23, TFRC | 85.71429 |
| 34 | GO:0099024 | plasma membrane invagination | IGLL1, IGHV3-33, IGHG4, IGLC6, MSR1, SH3BP1, DOCK1, IGHV4-28, IGHV4-39, IGHV5-51, IGHV3-7, IGHV1-45, IGHE, TRBC2, IGHV3-48, IGHG2, IGHD, IGHV3-43, IGHV1-58, IGHV2-70, IGHM, FCGR2B, PPARG, ALKBH4, IGHV3-49, FCER1G, IGHV4-61, IGHV4-59, CD36, IGHV3-30, IGLC1, ITGB2, IGHV1-3, IGHV4-4, IGHV2-26, ARHGAP12, IGHV3-15, IGHV3-21, FCGR1A, RHOBTB2, IGHV3-53, GSN, IGHG3, MEGF10, IGLC2, IGHA2, SPIRE2, IGHV3-11, IGLC7, STAP1, IGHV3-66, IGHV3-73, ELMO1, IGHV3-13, IGHV1-18, IGLL5, IGHV1OR15-1, IGHV3-72, ARF1, IGHV1-69, NCKAP1L, ADGRB1, ARHGAP25, MYH9, IGHV3-74, IGHV6-1, IGHV3-64, ITGAM, ABCA7, MFGE8, SNX9, IGLC3, IGHA1, CD300A, IGHG1, SPIRE1, IGHV4-34, SNX33, GULP1, IGHV3-20, IGHV1-24, XKR8, F2RL1, IGHV2-5, IGHV3-23 | 85.71429 |
| 34 | GO:0038093 | Fc receptor signaling pathway | IGHV3-33, IGHG4, IGLC6, IGLV2-23, DOCK1, IGHV4-39, VAV2, IGHV3-7, PLCG2, IGLV3-25, PAK2, IKBKG, IGHE, WASL, IGHV3-48, IGLV2-8, LCP2, IGHG2, IGLV2-11, ACTB, RELA, CD247, CARD11, PLA2G6, PRKCE, IGLV3-1, IGHV2-70, FCGR2B, MAPK1, CRK, WASF2, GRB2, ARPC5, PPP3CB, LAT2, IGLV1-51, ARPC4, MAPK10, CD47, FCER1G, RAPGEF1, RAP1A, IGHV4-59, LILRA4, CYFIP1, IKBKB, IGHV3-30, HSP90AA1, MYO1C, ARPC1B, IGLC1, PPP3R1, PDPK1, IGLV7-43, IGLV3-27, IGLV3-19, MYO1G, RAC1, IGLV1-40, FCGR1A, MAP3K1, YES1, IGHV3-53, WAS, FGR, HCK, LAT, ACTR2, IGHG3, FCGR2A, PTPRJ, NFATC2, ABL1, IGHV3-11, IGLC7, MALT1, BTK, ELMO1, PTK2, IGHV3-13, PTPRC, WIPF1, CD200R1, IGLV2-14, IGHV1OR15-1, IGLV1-47, TAB1, IGLV3-21, BRK1, ABI1, IGHV1-69, BCL10, FCER1A, NCKAP1L, PIK3CB, MAPK3, MYO10, GRAP2, NCK1, ARPC1A, IGLV1-44, NFATC1, IGHG1, IGLV6-57, SRC, PIGR, IGHV4-34, WIPF3, TEC, FCGR3A, IGHV2-5, NCKAP1, PRKCQ, MAPK9, FYN, LIMK1, NR4A3, IGHV3-23 | 85.71429 |
| 34 | GO:0002429 | immune response-activating cell surface receptor signaling pathway | TESPA1, IGLL1, IGHV3-33, CARD9, FPR1, RAB29, IGHG4, IGLC6, IGLV2-23, PVRIG, PAG1, CR1, CSK, KLHL6, FOXP3, DOCK1, IGHV4-28, IGHV4-39, DENND1B, DUSP22, IGHV5-51, IGHV3-7, VAV2, PLCG2, BLK, IGLV3-25, PAK2, IKBKG, IGHV1-45, IGHE, TRBC2, WASL, IGHV3-48, CTLA4, HRAS, IGLV2-8, PTPN2, CLEC10A, IGHG2, IGHD, NFAM1, IGLV2-11, LILRA2, ACTB, LCP2, RBCK1, MUC7, IGHV3-43, RELA, CD247, IGHV1-58, MUC17, FPR2, CARD11, PLA2G6, PRKCE, IGLV3-1, PAWR, IGHV2-70, IGHM, FCGR2B, MAPK1, CRK, WASF2, GCSAM, GRB2, ELF2, ARPC5, PHPT1, SLC39A10, LAT2, IGLV1-51, IGHV3-49, PDE4D, ARPC4, CD276, CD47, FCER1G, PRKACB, PAK3, RAPGEF1, IGHV4-61, RAP1A, INPP5D, TXK, IGHV4-59, CREBBP, MNDA, LIME1, CMTM3, KLRK1, TRAT1, BTNL9, PIK3CD, CYFIP1, IKBKB, FCN1, FPR3, IGHV3-30, HSP90AA1, MYO1C, ARPC1B, IGLC1, PAX5, PDPK1, IGHV1-3, IGHV4-4, IGLV7-43, EP300, IGLV3-27, RIPK2, IGHV2-26, ERMAP, IGLV3-19, SH2B2, DUSP3, CR2, MYO1G, CBFB, HHLA2, RAC1, IGHV3-15, IGLV1-40, MUCL1, MUC4, PLEKHA1, CD28, IGHV3-21, FCGR1A, MUC3A, EZR, YES1, IGHV3-53, WAS, CACNA1F, FGR, BTNL3, PLSCR1, LAT, HCK, GCSAML, RC3H2, ACTR2, IGHG3, FCGR2A, IGLC2, PTPRJ, NFATC2, IGHA2, GPR32, ABL1, IGHV3-11, FYB1, PLCL2, RC3H1, IGLC7, STAP1, MALT1, IGHV3-66, IGHV3-73, PRKACG, MUC12, BCL2, STOML2, BTK, ELMO1, MEF2C, RAF1, PTK2, IGHV3-13, LGALS3, PDE4B, IGHV1-18, PTPRC, WIPF1, BTNL8, GATA3, IGLV2-14, GPR32P1, IGLL5, FCRL3, IGHV1OR15-1, IGLV1-47, TAB1, IGHV3-72, IGLV3-21, GPS2, BRK1, ABI1, IGHV1-69, SLA2, BTNL10, CD38, BCL10, NCKAP1L, PIK3CB, MAPK3, MYO10, GRAP2, IGHV3-74, IGHV6-1, IGHV3-64, NCK1, ARPC1A, THEMIS2, KLRC4-KLRK1, IGLV1-44, IGLC3, IGHA1, LILRB4, CD300A, IGHG1, IGLV6-57, SRC, CD226, IGHV4-34, STK11, WIPF3, BAX, MUC20, IGHV3-20, IGHV1-24, MUC1, TEC, FCGR3A, IGHV2-5, ELF1, NCKAP1, CD19, PRKCQ, FYN, LIMK1, RFTN1, NR4A3, BRAF, IGHV3-23, EIF2B5 | 85.71429 |
| 34 | GO:0050864 | regulation of B cell activation | IGLL1, IGHV3-33, IGHG4, IGLC6, FOXP3, IGHV4-28, IGHV4-39, IGHV5-51, IGHV3-7, MLH1, IGHV1-45, IGHE, TNFSF4, TRBC2, PCID2, IGHV3-48, CTLA4, IGHG2, IGHD, NFAM1, IGHV3-43, INHBA, BCL6, IGHV1-58, PAWR, CARD11, IGHV2-70, IGHM, TNFSF13, FCGR2B, AHR, TNFRSF13B, IL7, MAD2L2, SLC39A10, IGHV3-49, SHLD2, IGHV4-61, THOC1, IL4, INPP5D, IGHV4-59, MNDA, IL6, IGHV3-30, GPR183, RIF1, PPP2R3C, IGLC1, APLF, IGHV3-23, BAD, IGHV1-3, TNFRSF13C, IGHV4-4, IGHV2-26, IRS2, PAXIP1, IGHV3-15, CD28, IGHV3-21, IGHV3-53, IL5, MIF, IGHG3, IGLC2, NFATC2, IGHA2, IL2, IL10, IGHV3-11, RC3H1, IGLC7, IGHV3-66, IGHV3-73, TNIP2, BCL2, ZFP36L2, CASP3, BTK, IL21, MEF2C, ATP11C, IGHV3-13, IGHV1-18, PTPRC, IGLL5, FCRL3, IGHV1OR15-1, IGHV3-72, ATAD5, NSD2, MSH6, SASH3, IGHV1-69, CD38, TNFRSF4, NCKAP1L, WNT3A, IGHV3-74, IGHV6-1, BST1, IGHV3-64, MSH2, NDFIP1, IGLC3, IGHA1, MIR17HG, XBP1, CD300A, IGHG1, IGHV4-34, IL13, PKN1, CLCF1, TBC1D10C, TNFSF13B, IGHV3-20, IGHV1-24, SFRP1, IGHV2-5, PELI1, TICAM1, SLA2, ZFP36L1, TFRC, TIRAP | 85.71429 |
| 34 | GO:0051249 | regulation of lymphocyte activation | CD83, TESPA1, IGLL1, SHH, IGHV3-33, EGR3, CD40LG, IRF1, GAS6, IGHG4, IGLC6, FOXP3, PAG1, SIRPB1, CR1, CSK, IRF4, TNFSF11, IGHV4-28, IGHV4-39, TMIGD2, DUSP22, IGHV5-51, IGHV3-7, TARM1, SOCS5, PAK2, EFNB2, AP3D1, MLH1, IGHV1-45, IGHE, IFNB1, TRBC2, CD1D, TNFSF4, PCID2, IGHV3-48, CTLA4, PTPN2, IGHG2, IGHD, NFAM1, FBXO7, CAV1, DOCK8, IGHV3-43, TNFRSF14, CLECL1, DNAJA3, INHBA, BCL6, IGHV1-58, PAWR, CARD11, IDO1, ANXA1, BMP4, HMGB1, IGHV2-70, IGHM, TNFSF13, FCGR2B, AHR, TNFRSF13B, GRB2, IL7, TAC1, XCL1, VCAM1, SOCS6, PGLYRP2, MARCH7, ICOS, MAD2L2, SPN, SLC39A10, IGHV3-49, MAPK8IP1, SHLD2, PYCARD, CD274, ITPKB, MAD1L1, CD276, CD47, PAK3, IGHV4-61, DPP4, THOC1, PIBF1, IL4, INPP5D, IGHV4-59, KLRK1, MNDA, LEP, MIR30B, PDE5A, SFTPD, MIR21, PLA2G2F, IL6, IGHV3-30, GLI3, GPR183, RIF1, SART1, PPP2R3C, IL6ST, FADD, IGLC1, APLF, MERTK, IGHV3-23, BAD, FANCA, PDPK1, IGHV1-3, FLT3LG, TNFRSF13C, FGF10, IGHV4-4, FANCD2, ADAM8, IFNA2, RIPK2, RHBDD3, IGHV2-26, NLRP3, IRS2, PAXIP1, DUSP3, HSPD1, CBFB, SIRPA, SOX13, HHLA2, RAC1, GNRH1, IGHV3-15, CD28, IGHV3-21, LAMP1, YES1, IL20RB, CORO1A, MAP3K8, IGHV3-53, PDCD1LG2, IL5, GSN, LAT, IL23R, NRARP, LILRB1, MIF, RC3H2, GPAM, IGHG3, IGLC2, CD46, NFATC2, IGHA2, IL2, IL10, IGHV3-11, EBI3, ABL1, ZP4, HES1, NKAP, RC3H1, IL27, IGLC7, CD55, MALT1, IGHV3-66, IGHV3-73, TNIP2, LGALS1, PRKCZ, BCL2, ZFP36L2, CASP3, BTK, ZC3H8, IL21, MEF2C, ATP11C, AP1G1, IL2RA, PLA2G2D, IGHV3-13, LGALS3, IGHV1-18, PTPRC, GATA3, HSPH1, SPTA1, IL1RL2, SIT1, IGLL5, FCRL3, IGHV1OR15-1, CD86, IGHV3-72, HLX, ZEB1, ATAD5, NSD2, MSH6, SASH3, IGHV1-69, SLA2, EPO, CD38, BCL10, TNFRSF4, RAC2, TNFSF18, NCKAP1L, WNT3A, IL12B, IL12A, GRAP2, EFNB3, IGHV3-74, IGHV6-1, TNFRSF18, SOCS1, BST1, IGHV3-64, MSH2, NCK1, TWSG1, RASAL3, IL15, NDFIP1, IL4R, KLRC4-KLRK1, AKT1, CD80, ITCH, IGLC3, IGHA1, MIR17HG, CCDC88B, PDCD1, XBP1, LILRB4, GPNMB, CD300A, IGHG1, SRC, DUSP10, DLG1, SOD1, IGHV4-34, PRDM1, PKN1, CLCF1, TBC1D10C, TNFSF13B, TIGIT, TMEM131L, IGHV3-20, IGHV1-24, CEBPB, LILRB2, SFRP1, IGHV2-5, SIRPG, PRKCQ, LAX1, FYN, BTLA, PELI1, TICAM1, BRAF, IL13, ZFP36L1, PRELID1, TFRC, TIRAP | 85.71429 |
| 34 | GO:0002449 | lymphocyte mediated immunity | CTSH, IGLL1, IGHV3-33, CD40LG, IGHG4, TUBB4B, IGLV2-23, IGLC6, FOXP3, CR1, NCR1, DENND1B, IGHV4-28, IGHV4-39, C8G, IGHV5-51, IGHV3-7, DUSP22, IGLV3-25, EXO1, MLH1, IGHV1-45, IGHE, IFNB1, CD1D, TRBC2, TNFSF4, TSTA3, IGHV3-48, IGLV2-8, SCART1, IGHG2, IGHD, IGLV2-11, IGHV3-43, BCL6, IL13RA2, IGHV1-58, TRAF2, APCS, SH2D1A, C1QBP, IGLV3-1, HMGB1, IGHV2-70, IGHM, FCGR2B, TRPM4, CRK, TNFSF13, C6, C4BPB, PPP3CB, XCL1, MAD2L2, IGLV1-51, IGHV3-49, CRP, SHLD2, FCER1G, IGHV4-61, THOC1, IL4, INPP5D, IGHV4-59, KLRK1, LEP, RAB27A, C4BPA, IL6, IGHV3-30, C1QC, RIF1, KLRF2, IGLC1, FADD, APLF, CLEC12B, IGHV1-3, LIG4, IGHV4-4, GZMM, IGLV7-43, IFNA2, IGLV3-27, C1QB, IGHV2-26, NLRP3, IGLV3-19, PAXIP1, SERPINB4, CR2, MYO1G, HSPD1, IGHV3-15, IGLV1-40, CD28, C9, IGHV3-21, LAMP1, CORO1A, IL20RB, IGHV3-53, WAS, IL23R, LILRB1, IGHG3, CD1A, IGLC2, CD46, IGHA2, IL2, MASP2, IGHV3-11, CD1C, SUSD4, IL10, IGLC7, CD55, MALT1, IGHV3-66, IGHV3-73, PRKCZ, PIK3R6, LYST, BTK, CD1B, IL1R1, IL21, AP1G1, IGHV3-13, GAPT, IGHV1-18, PTPRC, SLAMF6, KIR3DL1, GATA3, IGLV2-14, CD1E, EMP2, IGLL5, KLRD1, IGHV1OR15-1, CD96, IGLV1-47, IGHV3-72, ATAD5, NSD2, IGLV3-21, MSH6, SASH3, IGHV1-69, SLA2, CCR6, RSAD2, NBN, BCL10, SERPINB9, IL12B, IL12A, RNF168, KDELR1, IGHV3-74, IGHV6-1, IGHV3-64, MSH2, KDM5D, SLAMF7, IL18RAP, NDFIP1, IL4R, KLRC4-KLRK1, IGLV1-44, IGLC3, IGHA1, FZD5, IL18R1, C1QA, IGHG1, HPRT1, IGLV6-57, DLG1, CD226, C5, IL31RA, KLRC2, IGHV4-34, C7, CLCF1, CLEC2A, IGHV3-20, IGHV1-24, IGHV2-5, RFTN1, CLU, IGHV3-23, TFRC | 85.71429 |
| 34 | GO:0002433 | immune response-regulating cell surface receptor signaling pathway involved in phagocytosis | IGHV3-33, IGHG4, IGLC6, IGLV2-23, DOCK1, IGHV4-39, VAV2, IGHV3-7, PLCG2, IGLV3-25, WASL, IGHV3-48, IGLV2-8, IGHG2, IGLV2-11, ACTB, CD247, PLA2G6, PRKCE, IGLV3-1, IGHV2-70, FCGR2B, MAPK1, CRK, WASF2, GRB2, ARPC5, IGLV1-51, ARPC4, CD47, RAPGEF1, RAP1A, IGHV4-59, CYFIP1, IGHV3-30, HSP90AA1, MYO1C, ARPC1B, IGLC1, IGLV7-43, IGLV3-27, IGLV3-19, MYO1G, RAC1, IGLV1-40, FCGR1A, YES1, IGHV3-53, WAS, FGR, HCK, ACTR2, IGHG3, FCGR2A, PTPRJ, ABL1, IGHV3-11, IGLC7, ELMO1, PTK2, IGHV3-13, PTPRC, WIPF1, IGLV2-14, IGHV1OR15-1, IGLV1-47, IGLV3-21, BRK1, ABI1, IGHV1-69, NCKAP1L, PIK3CB, MAPK3, MYO10, NCK1, ARPC1A, IGLV1-44, IGHG1, IGLV6-57, SRC, IGHV4-34, WIPF3, FCGR3A, IGHV2-5, NCKAP1, FYN, LIMK1, IGHV3-23 | 85.71429 |
| 34 | GO:0002696 | positive regulation of leukocyte activation | CD83, TESPA1, IGLL1, SHH, IGHV3-33, EGR3, CD40LG, SIRPB1, GAS6, IGHG4, IGLC6, FOXP3, CSK, TNFSF11, IGHV4-28, IGHV4-39, TMIGD2, IGHV5-51, IGHV3-7, PAK2, EFNB2, AP3D1, MLH1, IGHV1-45, IGHE, CD1D, TRBC2, TNFSF4, PCID2, IGHV3-48, CTLA4, IGHG2, IGHD, CAV1, DOCK8, IGHV3-43, TNFRSF14, CLECL1, DNAJA3, BCL6, IGHV1-58, CARD11, ANXA1, HMGB1, IGHV2-70, IGHM, TNFSF13, GRB2, IL7, TAC1, XCL1, VCAM1, PTAFR, ICOS, MAD2L2, SLC39A10, IGHV3-49, SHLD2, PYCARD, CD274, ITPKB, FCER1G, CD276, CD47, PAK3, IGHV4-61, DPP4, IL4, INPP5D, IGHV4-59, KLRK1, MIR30B, LEP, MIR21, SART1, IL6, IGHV3-30, GLI3, GPR183, RIF1, PPP2R3C, IL6ST, FADD, IGLC1, IGHV3-23, BAD, ITGB2, CRLF2, PDPK1, IGHV1-3, FLT3LG, TNFRSF13C, LBP, FGF10, IGHV4-4, ADAM8, RIPK2, IGHV2-26, NLRP3, IRS2, PAXIP1, IL1RL1, HSPD1, CBFB, SIRPA, HHLA2, RAC1, STXBP1, IGHV3-15, CD28, IGHV3-21, LAMP1, YES1, CORO1A, MAP3K8, IGHV3-53, PDCD1LG2, IL5, FGR, IL23R, ADORA2B, LILRB1, MIF, GPAM, IGHG3, IGLC2, CD46, NFATC2, IGHA2, IL2, IL10, IGHV3-11, EBI3, SOCS5, ZP4, HES1, NKAP, IGLC7, CD55, STAP1, MALT1, IGHV3-66, IGHV3-73, TNIP2, LGALS1, PRKCZ, BCL2, BTK, IL21, MEF2C, ATP11C, AP1G1, IL2RA, IGHV3-13, IGHV1-18, PTPRC, GATA3, HSPH1, SPTA1, IL1RL2, IGLL5, FCRL3, IGHV1OR15-1, CD86, IGHV3-72, HLX, ATAD5, NSD2, MSH6, NPPA, SASH3, IGHV1-69, EPO, CD38, BCL10, TNFRSF4, NCKAP1L, WNT3A, IL12B, IL12A, GRAP2, EFNB3, IGHV3-74, IGHV6-1, SOCS1, BST1, IGHV3-64, MSH2, NCK1, RASAL3, ITGAM, IL15, IL4R, KLRC4-KLRK1, AKT1, CD80, IGLC3, IGHA1, CCDC88B, PDCD1, XBP1, LILRB4, IGHG1, SRC, DUSP10, CD226, IGHV4-34, IL33, CLCF1, STX4, TNFSF13B, IGHV3-20, IGHV1-24, LILRB2, F2RL1, IGHV2-5, SIRPG, PRKCQ, NR4A3, FYN, BTLA, PELI1, TICAM1, IL13, TFRC, TIRAP | 85.71429 |
| 34 | GO:0006956 | complement activation | IGLL1, IGHV3-33, IGHG4, IGLC6, IGLV2-23, CR1, IGHV4-28, IGHV4-39, C8G, IGHV5-51, IGHV3-7, KRT1, IGLV3-25, CFHR4, IGHV1-45, IGHE, TRBC2, CFHR5, IGHV3-48, IGLV2-8, IGHG2, IGHD, IGLV2-11, IGHV3-43, IGHV1-58, APCS, C1QBP, IGLV3-1, CFHR2, IGHV2-70, IGHM, C6, C4BPB, IGLV1-51, CPB2, IGHV3-49, SCARA3, CRP, IGHV4-61, IGHV4-59, CFHR1, C4BPA, FCN1, C1QC, IGHV3-30, IGLC1, IGHV1-3, IGHV4-4, IGLV7-43, IGLV3-27, C1QB, IGHV2-26, IGLV3-19, CR2, IGHV3-15, IGLV1-40, C9, CFH, IGHV3-21, RGCC, IGHV3-53, CFD, CPN2, FCN2, IGHG3, IGLC2, CD46, IGHA2, MASP2, IGHV3-11, SUSD4, IGLC7, CD55, IGHV3-66, IGHV3-73, IGHV3-13, IGHV1-18, COL20A1, MASP1, IGLV2-14, IGLL5, IGHV1OR15-1, IGLV1-47, FCN3, IGHV3-72, A2M, IGLV3-21, IGHV1-69, IGHV3-74, IGHV6-1, IGHV3-64, COLEC10, VSIG4, IGLV1-44, IGLC3, IGHA1, C1QA, IGHG1, IGLV6-57, C5, IGHV4-34, C7, IGHV3-20, IGHV1-24, IGHV2-5, CD19, CLU, IGHV3-23 | 85.71429 |
| 34 | GO:0051251 | positive regulation of lymphocyte activation | CD83, TESPA1, IGLL1, SHH, IGHV3-33, EGR3, CD40LG, SIRPB1, GAS6, IGHG4, IGLC6, FOXP3, CSK, TNFSF11, IGHV4-28, IGHV4-39, TMIGD2, IGHV5-51, IGHV3-7, PAK2, EFNB2, AP3D1, MLH1, IGHV1-45, IGHE, CD1D, TRBC2, TNFSF4, PCID2, IGHV3-48, CTLA4, IGHG2, IGHD, CAV1, DOCK8, IGHV3-43, TNFRSF14, CLECL1, DNAJA3, BCL6, IGHV1-58, CARD11, ANXA1, HMGB1, IGHV2-70, IGHM, TNFSF13, GRB2, IL7, TAC1, XCL1, VCAM1, ICOS, MAD2L2, SLC39A10, IGHV3-49, SHLD2, PYCARD, CD274, ITPKB, CD276, CD47, PAK3, IGHV4-61, DPP4, IL4, INPP5D, IGHV4-59, KLRK1, MIR30B, LEP, MIR21, SART1, IL6, IGHV3-30, GLI3, GPR183, RIF1, PPP2R3C, IL6ST, FADD, IGLC1, IGHV3-23, BAD, PDPK1, IGHV1-3, FLT3LG, TNFRSF13C, FGF10, IGHV4-4, ADAM8, RIPK2, IGHV2-26, NLRP3, IRS2, PAXIP1, HSPD1, CBFB, SIRPA, HHLA2, RAC1, IGHV3-15, CD28, IGHV3-21, LAMP1, YES1, CORO1A, MAP3K8, IGHV3-53, PDCD1LG2, IL5, IL23R, LILRB1, MIF, GPAM, IGHG3, IGLC2, NFATC2, CD46, IGHA2, IL2, EBI3, IGHV3-11, SOCS5, ZP4, HES1, NKAP, IGLC7, CD55, MALT1, IGHV3-66, IGHV3-73, TNIP2, LGALS1, PRKCZ, BCL2, BTK, IL21, MEF2C, ATP11C, AP1G1, IL2RA, IGHV3-13, IGHV1-18, PTPRC, GATA3, HSPH1, SPTA1, IL1RL2, IGLL5, FCRL3, IGHV1OR15-1, CD86, IGHV3-72, HLX, ATAD5, NSD2, MSH6, SASH3, IGHV1-69, EPO, CD38, BCL10, TNFRSF4, NCKAP1L, WNT3A, IL12B, IL12A, GRAP2, EFNB3, IGHV3-74, IGHV6-1, SOCS1, BST1, IGHV3-64, MSH2, NCK1, RASAL3, IL15, IL4R, KLRC4-KLRK1, AKT1, CD80, IGLC3, IGHA1, CCDC88B, PDCD1, XBP1, LILRB4, IGHG1, SRC, DUSP10, IGHV4-34, CLCF1, TNFSF13B, IGHV3-20, IGHV1-24, LILRB2, IGHV2-5, SIRPG, PRKCQ, FYN, BTLA, PELI1, TICAM1, IL13, TFRC, TIRAP | 85.71429 |
| 34 | GO:0002768 | immune response-regulating cell surface receptor signaling pathway | TESPA1, IGLL1, IGHV3-33, CARD9, FPR1, RAB29, IGHG4, IGLC6, IGLV2-23, PVRIG, PAG1, CR1, CSK, KLHL6, FOXP3, DOCK1, IGHV4-28, IGHV4-39, DENND1B, DUSP22, IGHV5-51, IGHV3-7, VAV2, PLCG2, BLK, IGLV3-25, PAK2, IKBKG, IGHV1-45, IGHE, TRBC2, WASL, IGHV3-48, CTLA4, HRAS, IGLV2-8, PTPN2, CLEC10A, IGHG2, IGHD, NFAM1, IGLV2-11, LILRA2, ACTB, LCP2, RBCK1, MUC7, IGHV3-43, RELA, CD247, IGHV1-58, MUC17, FPR2, CARD11, PLA2G6, PRKCE, IGLV3-1, PAWR, IGHV2-70, IGHM, FCGR2B, MAPK1, CRK, WASF2, GCSAM, GRB2, ELF2, ARPC5, PPP3CB, PHPT1, SLC39A10, LAT2, IGLV1-51, IGHV3-49, PDE4D, ARPC4, MAPK10, CD276, CD47, FCER1G, PRKACB, PAK3, RAPGEF1, IGHV4-61, RAP1A, INPP5D, TXK, IGHV4-59, CREBBP, MNDA, LIME1, CMTM3, KLRK1, TRAT1, BTNL9, PIK3CD, LILRA4, CYFIP1, IKBKB, FCN1, FPR3, IGHV3-30, HSP90AA1, MYO1C, ARPC1B, IGLC1, PAX5, CLEC12B, PPP3R1, PDPK1, IGHV1-3, IGHV4-4, IGLV7-43, EP300, IGLV3-27, RIPK2, IGHV2-26, ERMAP, IGLV3-19, SH2B2, DUSP3, CR2, MYO1G, CBFB, HHLA2, RAC1, IGHV3-15, IGLV1-40, MUCL1, MUC4, PLEKHA1, CD28, IGHV3-21, FCGR1A, MUC3A, MAP3K1, EZR, YES1, IGHV3-53, WAS, CACNA1F, FGR, BTNL3, PLSCR1, LAT, HCK, GCSAML, RC3H2, LILRB1, ACTR2, IGHG3, FCGR2A, IGLC2, PTPRJ, NFATC2, IGHA2, GPR32, ABL1, IGHV3-11, FYB1, PLCL2, RC3H1, IGLC7, STAP1, MALT1, IGHV3-66, IGHV3-73, PRKACG, MUC12, BCL2, STOML2, BTK, ELMO1, MEF2C, RAF1, IGHV3-13, LGALS3, PDE4B, IGHV1-18, PTPRC, WIPF1, CD200R1, BTNL8, GATA3, IGLV2-14, GPR32P1, IGLL5, FCRL3, IGHV1OR15-1, IGLV1-47, TAB1, IGHV3-72, IGLV3-21, GPS2, BRK1, ABI1, IGHV1-69, SLA2, BTNL10, CD38, BCL10, FCER1A, NCKAP1L, PIK3CB, MAPK3, MYO10, GRAP2, IGHV3-74, IGHV6-1, IGHV3-64, NCK1, ARPC1A, THEMIS2, KLRC4-KLRK1, IGLV1-44, IGLC3, IGHA1, LILRB4, NFATC1, CD300A, IGHG1, IGLV6-57, KIR2DL1, CD226, SRC, PIGR, IGHV4-34, STK11, WIPF3, BAX, MUC20, IGHV3-20, IGHV1-24, MUC1, TEC, LILRB2, FCGR3A, IGHV2-5, ELF1, NCKAP1, CD19, PRKCQ, FYN, LIMK1, RFTN1, BTLA, BRAF, MAPK9, NR4A3, IGHV3-23, EIF2B5 | 85.71429 |
| 34 | GO:0010324 | membrane invagination | SYT11, IGLL1, IGHV3-33, IGHG4, IGLC6, MSR1, SH3BP1, DOCK1, IGHV4-28, IGHV4-39, IGHV5-51, IGHV3-7, IGHV1-45, IGHE, TRBC2, IGHV3-48, IGHG2, IGHD, IGHV3-43, IGHV1-58, IGHV2-70, IGHM, FCGR2B, PPARG, ALKBH4, IGHV3-49, FCER1G, IGHV4-61, IGHV4-59, CD36, IGHV3-30, IGLC1, ITGB2, IGHV1-3, IGHV4-4, IGHV2-26, ARHGAP12, IGHV3-15, IGHV3-21, FCGR1A, RHOBTB2, IGHV3-53, GSN, IGHG3, MEGF10, IGLC2, IGHA2, SPIRE2, IGHV3-11, IGLC7, STAP1, IGHV3-66, IGHV3-73, ELMO1, IGHV3-13, IGHV1-18, IGLL5, IGHV1OR15-1, IGHV3-72, ARF1, IGHV1-69, NCKAP1L, ADGRB1, ARHGAP25, MYH9, IGHV3-74, IGHV6-1, IGHV3-64, ITGAM, ABCA7, MFGE8, SNX9, IGLC3, IGHA1, CD300A, IGHG1, SPIRE1, IGHV4-34, SNX33, GULP1, IGHV3-20, IGHV1-24, XKR8, F2RL1, SMURF1, IGHV2-5, IGHV3-23 | 85.71429 |
| 34 | GO:0002673 | regulation of acute inflammatory response | IGHV3-33, PARK7, IGHG4, IGLC6, IGLV2-23, CR1, TNFSF11, IGHV4-39, C8G, IGHV3-7, CFHR4, IGLV3-25, MIR92A2, GSTP1, CFHR5, IGHV3-48, IGLV2-8, IGHG2, IGLV2-11, PIK3CG, APCS, C1QBP, IGLV3-1, CFHR2, OSMR, IGHV2-70, FCGR2B, C6, TAC1, C4BPB, PPARG, IGLV1-51, CPB2, FCER1G, IGHV4-59, CFHR1, C4BPA, KLKB1, TNFRSF11A, IGHV3-30, IL6, C1QC, IL6ST, IGLC1, F12, ADAM8, IGLV7-43, C1QB, IGLV3-27, RHBDD3, ADORA1, NLRP3, IGLV3-19, CR2, IGLV1-40, C9, CFH, IL20RB, IGHV3-53, SELENOS, CPN2, MIR92A1, IGHG3, CD46, SUSD4, IGHV3-11, IGLC7, CD55, OSM, CREB3L3, DNASE1, BTK, ADCYAP1, IGHV3-13, IGLV2-14, IGHV1OR15-1, IGLV1-47, DNASE1L3, A2M, IGLV3-21, IGHV1-69, EDNRB, ASH1L, PTGS2, PTGER3, IGLV1-44, C1QA, IGHG1, IGLV6-57, C5, IGHV4-34, C7, IGHV2-5, CD19, CLU, IGHV3-23 | 85.71429 |
| 34 | GO:0002920 | regulation of humoral immune response | IGHV3-53, IGLV3-21, IGHV3-33, CXCL13, IGHV1-69, IGHG4, IGLC6, IGLV2-23, CPN2, CR1, ACOD1, IGHV4-59, IGHV4-39, CFHR1, C8G, IGHG3, IGHV3-7, CD46, C4BPA, CFHR4, IGLV3-25, SUSD4, IGHV3-11, ZP4, IGHV3-30, C1QC, PPP2R3C, CFHR5, IGLC7, CD55, IGHV3-48, IGLC1, IGLV2-8, IGLV1-44, IGHG2, IGLV2-11, C1QA, IGHG1, IGLV6-57, IGLV7-43, C5, IGHV3-13, IGLV3-27, IGHV4-34, PTPRC, IGLV3-1, CFHR2, IGLV3-19, IGHV2-70, C1QB, FCGR2B, C7, C1QBP, IGLV2-14, CR2, C6, KLK5, C4BPB, IGHV2-5, IGHV1OR15-1, KLK7, IGLV1-47, IGLV1-40, CD19, C9, CFH, CLU, IGLV1-51, IGHV3-23, CPB2, A2M | 85.71429 |
| 34 | GO:2000257 | regulation of protein activation cascade | IGHV3-53, IGLV3-21, SERPINC1, IGHV3-33, IGHV1-69, IGHG4, IGLC6, IGLV2-23, CPN2, CR1, IGHV4-59, IGHV4-39, IGHG3, CFHR1, C8G, IGHV3-7, CD46, C4BPA, CFHR4, IGLV3-25, SUSD4, IGHV3-11, C1QC, IGHV3-30, CFHR5, IGLC7, CD55, IGHV3-48, IGLC1, IGLV2-8, IGLV1-44, IGHG2, IGLV2-11, C1QA, IGHG1, IGLV6-57, IGLV7-43, C5, IGHV3-13, IGLV3-27, IGHV4-34, IGLV3-1, C1QB, CFHR2, IGLV3-19, IGHV2-70, C7, C1QBP, IGLV2-14, CR2, C6, C4BPB, IGHV2-5, IGHV1OR15-1, IGLV1-47, IGLV1-40, CD19, C9, CFH, CLU, IGLV1-51, IGHV3-23, CPB2, A2M | 85.71429 |
| 34 | GO:0006898 | receptor-mediated endocytosis | SYT11, AMN, AP1S1, CLTCL1, IGHV3-33, CACNG3, CLTC, IGLC6, IGLV2-23, ACHE, AP2A1, MSR1, IGHV4-39, OPHN1, GPR107, IGHV3-7, PLCG2, IGLV3-25, EFNB2, CANX, CACNG5, WASL, EPS15, IGHV3-48, CALCRL, IGLV2-8, SCART1, IGLV2-11, GSG1L, SCYL2, CAV1, LRP1B, CAV3, ILDR1, CD63, VLDLR, SFRP4, RAMP1, LRP2, IGLV3-1, CLEC9A, IGHV2-70, FCGR2B, NEDD4, CACNG8, GRB2, IGF2R, MIR199A2, HTR1B, SCRIB, CLTB, IGLV1-51, SCARA3, FCER1G, SYNJ1, DAB2, CNTN2, IGHV4-59, CD36, SFTPD, SGIP1, IGHV3-30, HSP90AA1, SCARF1, IGLC1, MIR185, JCHAIN, GRK4, ITGB2, DBNL, ASGR2, FMR1, COLEC12, ANXA2P2, NECAB2, IGLV7-43, SCGB3A2, IGLV3-27, PLA2R1, IGLV3-19, DNM1P34, LRRTM2, CD5L, RAC1, SUSD2, IGLV1-40, HHIPL1, FCGR1A, EZR, IGHV3-53, CLTA, SMAP1, LILRB1, MKLN1, CACNG2, SH3GL2, MEGF10, TFR2, SCARA5, RABEPK, APOL1, IGHA2, TBC1D5, IGHV3-11, HIP1, PICK1, CD207, CALR, IGLC7, MICALL1, CD14, DNM1, HTR2B, DLL1, SELE, ITGA4, AAK1, CACNG4, PCSK9, IGHV3-13, MASP1, IGLV2-14, LOXL2, HSPH1, TINAGL1, CLN3, RAB31, CAV2, APOBR, IGHV1OR15-1, LDLR, IGLV1-47, PDLIM7, DMBT1, DNM3, PRG4, IGLV3-21, ASGR1, IGHV1-69, TNK2, ENPP2, GAK, LRPAP1, PIK3CB, ATAD1, CALY, PIP5K1C, CUBN, SERPINE1, SNCA, CACNG7, MRC1, IGLV1-44, ACKR3, SNX9, IGHA1, IGLV6-57, RAMP3, IGHV4-34, RAB21, MAGI2, PRSS12, DRD3, LRP6, IGHV2-5, CLU, DLG4, ITGB1, IGHV3-23, AP2M1, TFRC | 85.71429 |
| 34 | GO:0050871 | positive regulation of B cell activation | IGLL1, IGHV3-33, IGHG4, IGLC6, IGHV4-28, IGHV4-39, IGHV5-51, IGHV3-7, MLH1, IGHV1-45, IGHE, TNFSF4, TRBC2, PCID2, IGHV3-48, IGHG2, IGHD, IGHV3-43, BCL6, IGHV1-58, CARD11, IGHV2-70, IGHM, TNFSF13, IL7, MAD2L2, SLC39A10, IGHV3-49, SHLD2, IGHV4-61, IL4, INPP5D, IGHV4-59, IL6, IGHV3-30, GPR183, RIF1, PPP2R3C, IGLC1, IGHV3-23, BAD, IGHV1-3, TNFRSF13C, IGHV4-4, IGHV2-26, IRS2, PAXIP1, IGHV3-15, CD28, IGHV3-21, IGHV3-53, IL5, MIF, IGHG3, IGLC2, NFATC2, IGHA2, IL2, IGHV3-11, IGLC7, IGHV3-66, IGHV3-73, TNIP2, BCL2, BTK, IL21, MEF2C, ATP11C, IGHV3-13, IGHV1-18, PTPRC, IGLL5, FCRL3, IGHV1OR15-1, IGHV3-72, ATAD5, NSD2, MSH6, SASH3, IGHV1-69, CD38, TNFRSF4, NCKAP1L, WNT3A, IGHV3-74, IGHV6-1, BST1, IGHV3-64, MSH2, IGLC3, IGHA1, XBP1, IGHG1, IGHV4-34, CLCF1, TNFSF13B, IGHV3-20, IGHV1-24, IGHV2-5, PELI1, TICAM1, IL13, TFRC, TIRAP | 85.71429 |
| 34 | GO:0002431 | Fc receptor mediated stimulatory signaling pathway | IGHV3-33, IGHG4, IGLC6, IGLV2-23, CSK, DOCK1, IGHV4-39, VAV2, IGHV3-7, PLCG2, IGLV3-25, WASL, IGHV3-48, IGLV2-8, IGHG2, IGLV2-11, ACTB, CD247, PLA2G6, PRKCE, IGLV3-1, IGHV2-70, FCGR2B, MAPK1, CRK, WASF2, GRB2, ARPC5, IGLV1-51, ARPC4, CD47, FCER1G, RAPGEF1, RAP1A, IGHV4-59, CYFIP1, IGHV3-30, HSP90AA1, MYO1C, ARPC1B, IGLC1, IGLV7-43, IGLV3-27, IGLV3-19, MYO1G, RAC1, IGLV1-40, FCGR1A, YES1, IGHV3-53, WAS, FGR, PLSCR1, HCK, ACTR2, IGHG3, FCGR2A, PTPRJ, ABL1, IGHV3-11, IGLC7, ELMO1, PTK2, IGHV3-13, PTPRC, WIPF1, IGLV2-14, IGHV1OR15-1, IGLV1-47, IGLV3-21, BRK1, ABI1, IGHV1-69, NCKAP1L, PIK3CB, MAPK3, MYO10, NCK1, ARPC1A, IGLV1-44, IGHG1, IGLV6-57, SRC, CD226, IGHV4-34, WIPF3, FCGR3A, IGHV2-5, NCKAP1, NR4A3, FYN, LIMK1, IGHV3-23 | 85.71429 |
| 34 | GO:0006911 | phagocytosis, engulfment | IGHV3-53, FCER1G, IGLL1, GSN, IGHV3-33, IGHV1-69, IGHV4-61, IGHG4, IGLC6, NCKAP1L, IGHV4-59, SH3BP1, DOCK1, IGHV4-28, IGHV4-39, MSR1, IGHG3, IGHV5-51, IGHV3-7, MEGF10, CD36, ARHGAP25, IGLC2, MYH9, IGHA2, IGHV3-74, IGHV6-1, IGHV3-11, IGHV3-30, IGHE, IGHV1-45, IGHV3-64, TRBC2, ITGAM, IGLC7, IGLC1, IGHV3-48, STAP1, ABCA7, IGHV3-66, IGHV3-73, MFGE8, ADGRB1, IGHG2, IGHD, ITGB2, IGLC3, IGHA1, IGHV1-3, IGHV3-43, CD300A, IGHG1, IGHV4-4, ELMO1, IGHV1-58, IGHV3-13, IGHV4-34, IGHV1-18, IGHV2-26, ARHGAP12, GULP1, IGHV2-70, IGHM, FCGR2B, IGHV3-20, IGHV1-24, XKR8, F2RL1, IGLL5, IGHV2-5, PPARG, IGHV1OR15-1, IGHV3-15, IGHV3-21, IGHV3-72, FCGR1A, IGHV3-23, RHOBTB2, IGHV3-49 | 85.71429 |
| 34 | GO:0002460 | adaptive immune response based on somatic recombination of immune receptors built from immunoglobulin superfamily domains | CTSH, IGLL1, IGHV3-33, CXCL13, CD40LG, IGHG4, IGLC6, IGLV2-23, FOXP3, CR1, KLHL6, IRF4, DENND1B, IGHV4-28, IGHV4-39, C8G, IGHV5-51, IGHV3-7, DUSP22, IGLV3-25, EXO1, MLH1, IGHV1-45, IGHE, IFNB1, CD1D, TRBC2, TNFSF4, TSTA3, IGHV3-48, HRAS, IGLV2-8, SCART1, IGHG2, IGHD, IGLV2-11, IGHV3-43, BCL6, IL13RA2, IGHV1-58, TRAF2, APCS, ANXA1, IGLV3-1, HMGB1, C1QBP, IGHV2-70, IGHM, FCGR2B, TRPM4, TNFSF13, C6, C4BPB, PPP3CB, XCL1, MAD2L2, SPN, IGLV1-51, IGHV3-49, CRP, SHLD2, CD274, FCER1G, IGHV4-61, THOC1, IL4, INPP5D, IGHV4-59, RAB27A, C4BPA, MIR21, LY9, IL6, IGHV3-30, C1QC, RIF1, FADD, IGLC1, APLF, IGHV1-3, TNFRSF13C, LIG4, IGHV4-4, GZMM, IGLV7-43, IFNA2, IGLV3-27, RIPK2, IGHV2-26, NLRP3, IGLV3-19, C1QB, PAXIP1, IL1RL1, MTOR, CR2, MYO1G, HSPD1, IGHV3-15, IGLV1-40, SEMA4A, CD28, C9, IGHV3-21, IL20RB, IGHV3-53, WAS, IL23R, RC3H2, LILRB1, IGHG3, CD1A, IGLC2, CD46, SOCS5, IGHA2, IL2, MASP2, IGHV3-11, CD1C, SUSD4, IL10, EBI3, RC3H1, IL27, IGLC7, CD55, MALT1, IGHV3-66, IGHV3-73, PRKCZ, BTK, CD1B, IL1R1, MEF2C, IGHV3-13, GAPT, IGHV1-18, PTPRC, SLAMF6, GATA3, IGLV2-14, CD1E, EMP2, IGLL5, IGHV1OR15-1, IGLV1-47, IGHV3-72, HLX, ATAD5, NSD2, IGLV3-21, MSH6, SASH3, IGHV1-69, SLA2, CCR6, RSAD2, NBN, BCL10, IL12B, IL12A, RNF168, KDELR1, IGHV3-74, IGHV6-1, IGHV3-64, MSH2, KDM5D, IL18RAP, NDFIP1, IL4R, CD80, IGLV1-44, IGLC3, IGHA1, FZD5, IL18R1, C1QA, IGHG1, HPRT1, IGLV6-57, DLG1, CD226, C5, IL31RA, IGHV4-34, IL33, PKN1, C7, CLCF1, TNFSF13B, IGHV3-20, IGHV1-24, IGHV2-5, PRKCQ, RFTN1, CLU, IGHV3-23, TFRC | 85.71429 |
| 34 | GO:0006959 | humoral immune response | CD83, IGLL1, IGHV3-33, CXCL13, ROMO1, IGHG4, IGLC6, IGLV2-23, CR1, IGHV4-28, IGHV4-39, IFNA5, C8G, IGHV5-51, IGHV3-7, DEFB127, DEFA3, KRT1, IGLV3-25, EXO1, SPON2, CHGA, CFHR4, IGHV1-45, IGHE, IFNB1, TRBC2, DEFB1, CFHR5, DEFA4, PRTN3, IGHV3-48, DEFB126, IGLV2-8, IGHG2, IGHD, IGLV2-11, MUC7, AZU1, IGHV3-43, IGHV1-58, DCD, APCS, SH2D1A, PLA2G6, C1QBP, IGLV3-1, IFNA14, KLK3, CFHR2, IGHV2-70, IGHM, FCGR2B, ITLN1, DEFB4A, C6, BPI, IL7, C4BPB, PGLYRP2, BPIFA1, IGLV1-51, HTN1, ST6GAL1, CPB2, IGHV3-49, SCARA3, CRP, IFNA6, MNX1, IFNA4, IFNA10, DEFA5, IGHV4-61, IFNA13, BPIFA2, IGHV4-59, CFHR1, C4BPA, SFTPD, FCN1, IFNE, HTN3, DEFA6, IGHV3-30, GPR183, IL6, C1QC, PPP2R3C, IGLC1, PAX5, YTHDF2, JCHAIN, HRG, IFNA16, IGHV1-3, IGHV4-4, IGLV7-43, IFNA2, IGLV3-27, C1QB, IGHV2-26, RPL39, IGLV3-19, FAM3A, KLK5, DEFA1, CR2, BPIFB2, DEFB118, IGHV3-15, IGLV1-40, CD28, C9, IFNA1, IGHV3-21, CFH, ACOD1, RGCC, IGHV3-53, CFD, CPN2, FCN2, PRSS3, IGHG3, FGA, LCN2, IGLC2, CD46, IGHA2, MASP2, TFE3, IGHV3-11, SUSD4, EBI3, ZP4, IGLC7, CD55, IGHV3-66, IGHV3-73, NOTCH1, DEFB103B, BCL2, DEFA1B, IFNA17, MEF2C, IGHV3-13, LEAP2, IGHV1-18, PTPRC, COL20A1, IFNA7, GATA3, MASP1, IGLV2-14, DEFB103A, IGLL5, TRAF3IP2, IGHV1OR15-1, KLK7, IGLV1-47, FCN3, DMBT1, IGHV3-72, IFNA21, A2M, IGLV3-21, IGHV1-69, CCR6, RNASE7, RARRES2, IGHV3-74, IGHV6-1, BST1, IGHV3-64, FGB, IFNK, COLEC10, VSIG4, IGLV1-44, RNASE6, IFNA8, IGLC3, IGHA1, PLA2G2A, PDCD1, C1QA, IGHG1, IGLV6-57, ELANE, C5, IGHV4-34, C7, RNASE3, IGHV3-20, IGHV1-24, IGHV2-5, BPIFB1, CD19, CLU, IFNW1, IGHV3-23 | 85.71429 |
| 34 | GO:0070613 | regulation of protein processing | IGHV3-33, IGHG4, IGLC6, IGLV2-23, CR1, IGHV4-39, C8G, MELTF, IGHV3-7, IGLV3-25, CFHR4, PRSS37, CFHR5, IGHV3-48, IGLV2-8, IGHG2, IGLV2-11, USP17L2, NKD2, NOL3, C1QBP, IGLV3-1, CFHR2, IGHV2-70, C6, C4BPB, ACP4, IGLV1-51, CPB2, PRKACB, CNTN2, IGHV4-59, CFHR1, C4BPA, KLKB1, C1QC, IGHV3-30, IGLC1, SERPINF2, ANXA2P2, F12, ADAM8, IGLV7-43, IGLV3-27, C1QB, IGLV3-19, CR2, XIAP, IGLV1-40, C9, PLGRKT, CFH, TMEM59, IGHV3-53, GSN, CPN2, IGHG3, CD46, IGHV3-11, SUSD4, ENO1, IGLC7, CD55, TIMM17A, CARD8, IGHV3-13, IGLV2-14, CTSZ, IGHV1OR15-1, SERPINE2, IGLV1-47, ANGPTL8, A2M, IGLV3-21, IGHV1-69, MYH9, SERPINE1, NLRP7, BIRC7, IGLV1-44, BCL2L12, C1QA, IGHG1, IGLV6-57, SRC, C5, IGHV4-34, C7, IL1R2, IGHV2-5, CD19, CLU, TNP2, IGHV3-23, CCBE1, GLG1 | 85.71429 |
| 34 | GO:0042742 | defense response to bacterium | SYT11, IGLL1, IGHV3-33, CXCL13, CARD9, ROMO1, IGHG4, IGLC6, IGHV4-28, IGHV4-39, IRF8, DEFA3, IGHV5-51, IGHV3-7, DEFB127, UNC13B, DEFB115, SPON2, CHGA, DEFB135, IGHV1-45, IGHE, TRBC2, DEFB1, DEFA4, IGHV3-48, DEFB126, IGHG2, IGHD, GSDMD, DEFB121, SIGLEC11, IGHV3-43, AZU1, TNFRSF14, HMGB2, IGHV1-58, DCD, DEFB116, PLA2G6, KLK3, TLR2, IGHV2-70, IGHM, DEFB136, DEFB4A, BPI, ISG15, TIRAP, PGLYRP2, DEFB132, C10orf99, BPIFA1, DEFB125, SPN, DEFB106B, HTN1, LYZL2, DEFB131A, IGHV3-49, CRP, PYCARD, FCER1G, DEFA5, IGHV4-61, BPIFA2, IGHV4-59, KLRK1, CD36, EPX, SFTPD, IFNE, DEFB104A, DEFB124, HTN3, DEFA6, IGHV3-30, IL6, NLRP1, RAB14, MR1, MAVS, IGLC1, JCHAIN, DEFB104B, DEFB108A, DEFB119, IGHV1-3, FOXP1, LBP, IGHV4-4, MPO, NOD1, RIPK2, DEFB128, IGHV2-26, NLRP3, RPL39, DEFB106A, DEFA1, KLK5, DEFB105A, DEFB134, DEFB118, IGHV3-15, LYZL1, IGHV3-21, ANXA3, IGHV3-53, FGR, IL23R, FCN2, RNASE8, IGHG3, FGA, IGLC2, IGHA2, SPAG11A, IL10, IGHV3-11, DEFB129, NR1H4, IGLC7, LYPD8, IGHV3-66, IGHV3-73, TLR3, SELP, DEFB103B, DEFA1B, CYBA, LYST, DEFB130A, DEFB109B, IGHV3-13, LEAP2, IGHV1-18, DEFB123, PRB3, F2, DEFB103A, HP, OPTN, IGLL5, IGHV1OR15-1, DEFB130B, IL22RA1, KLK7, DMBT1, IGHV3-72, LPO, SIGLEC16, PLAC8, IGHV1-69, RNASE7, ADGRB1, IL12B, IL12A, RARRES2, DEFB105B, STATH, IGHV3-74, IGHV6-1, IGHV3-64, FGB, SERPINE1, VGF, KLRC4-KLRK1, RNASE6, TLR5, IGLC3, IGHA1, PLA2G2A, ACP5, SPAG11B, IGHG1, ELANE, IGHV4-34, TMF1, RNASE3, IGHV3-20, IGHV1-24, CEBPB, F2RL1, TBK1, IGHV2-5, LACRT, IGHV3-23, EPHA2, RAB1A | 85.71429 |
| 34 | GO:0038095 | Fc-epsilon receptor signaling pathway | IGHV3-53, IGLV3-21, FCER1G, IGHV3-33, IGHV1-69, IGLC6, IGLV2-23, LAT, BCL10, IGHV4-59, FCER1A, PIK3CB, IGHV4-39, VAV2, IGHV3-7, PLCG2, LILRA4, MAPK3, IKBKB, IGLV3-25, GRAP2, NFATC2, IKBKG, PAK2, IGHV3-11, IGHV3-30, IGHE, IGLC7, IGLC1, IGHV3-48, MALT1, IGLV2-8, IGLV1-44, LCP2, IGLV2-11, PPP3R1, PDPK1, NFATC1, BTK, RELA, IGLV6-57, IGLV7-43, IGHV3-13, IGLV3-27, IGHV4-34, IGLV3-1, CARD11, IGLV3-19, IGHV2-70, MAPK1, TEC, GRB2, IGLV2-14, PPP3CB, IGHV2-5, RAC1, IGHV1OR15-1, NR4A3, IGLV1-47, IGLV1-40, PRKCQ, TAB1, MAPK9, LAT2, IGLV1-51, IGHV3-23, MAP3K1, MAPK10 | 85.71429 |
| 34 | GO:0008037 | cell recognition | TCP1, ROBO4, IGLL1, IGHV3-33, IGHG4, IGLC6, PEAR1, IGHV4-28, IGHV4-39, IGHV5-51, IGHV3-7, EPHB3, OVGP1, EPHB2, CCT2, IGHV1-45, IGHE, PRSS37, TRBC2, IGHV3-48, IGHG2, IGHD, DOCK8, IGHV3-43, CNTNAP3, ZAN, IGHV1-58, IGHV2-70, IGHM, VSTM2L, PCDH12, TNN, NDN, PCSK4, IGHV3-49, IGHV4-61, CDK5R1, CNTN2, IGHV4-59, CD36, IGSF9, CSGALNACT1, CCT3, FCN1, CATSPER3, IGHV3-30, IGLC1, ADAM21, ATP8B3, IGHV1-3, FETUB, BSG, IGHV4-4, COLEC12, CLEC7A, IGHV2-26, MSN, ROBO3, IGHV3-15, CNTN4, IGHV3-21, VDAC2, IGHV3-53, SPA17, B4GALT1, ACR, DOCK2, FCN2, IGHG3, MEGF10, IGLC2, IGHA2, SEMA3A, IGHV3-11, ZP4, DSCAM, ALDOA, IGLC7, CLGN, IGHV3-66, IGHV3-73, ADAM2, SEMA5A, CASP3, SPAM1, ZPBP, IGHV3-13, LGALS3, IGHV1-18, EPHA4, GAP43, IGLL5, IGHV1OR15-1, FCN3, IGHV3-72, CCT5, CNTNAP2, NEXN, PCDHA7, AMIGO1, IGHV1-69, PAEP, KCNU1, ADGRB1, CATSPER1, EFNB3, IGHV3-74, IGHV6-1, IGHV3-64, PCDHB6, NRP1, CCT4, MFGE8, IGLC3, IGHA1, NRCAM, IGHG1, DLG1, CD226, ST6GALNAC6, IGHV4-34, LAMA5, EMB, IGHV3-20, IGHV1-24, CNTN6, IGHV2-5, SPESP1, NPTN, IGHV3-23, IZUMO1, UBAP2L | 85.71429 |

---


---


---

# Cluster 57

| Cluster | Term | Name |
| --- | --- | --- |
| 57 | HP:0001252 | Muscular hypotonia |
| 57 | HP:0000153 | Abnormality of the mouth |
| 57 | HP:0000194 | Open mouth |
| 57 | HP:0000341 | Narrow forehead |
| 57 | HP:0001837 | Broad toe |

| Cluster | Term | Name | Genes | Percentage\_of\_nodes\_with\_funsys |
| --- | --- | --- | --- | --- |
| 57 | GO:0051604 | protein maturation | SEC11C, DHPS, CTSH, LGMN, SHH, IGHV3-33, NAA15, IGHG4, IGLC6, IGLV2-23, STUB1, KLK13, CR1, IGHV4-39, HSCB, C8G, F9, IGHV3-7, MELTF, ASPH, FKBP1A, IGLV3-25, CFHR4, IMMP1L, ASPRV1, YAE1, PRSS37, NNAT, CFHR5, IGHV3-48, IGLV2-8, IGHG2, LMF2, KLK2, IGLV2-11, YME1L1, USP17L2, MME, HM13, GCSH, IGLV3-1, AGA, KLK3, CFHR2, IGHV2-70, C6, C4BPB, PISD, PCSK4, CPXM1, ACP4, IGLV1-51, NAA40, CPB2, PYCARD, CLN5, IMMP2L, CGB3, SOX4, CTSE, ERO1A, CPXM2, NAA60, IGHV4-59, CREBBP, CNTN2, CFHR1, C4BPA, DDI2, ADAMTS2, KLKB1, C1QC, IGHV3-30, GLI3, KLK1, ERO1B, FADD, IGLC1, XPNPEP3, CPE, MAGEF1, BAD, F7, PMPCA, SERPINF2, ANXA2P2, TSPAN14, ADAM8, IGLV7-43, F12, EP300, IGLV3-27, RIPK2, C1QB, IGLV3-19, LTO1, SCG5, CR2, HSPD1, PLAU, C9orf3, XIAP, PCSK6, IGLV1-40, REN, AIP, C9, PLGRKT, SPCS3, CFH, TMEM59, IGHV3-53, GLRX3, GSN, MMP16, CPN2, PSEN2, PRSS3, SEC11B, IGHG3, TFR2, FGA, NAA50, CASP8, ASRGL1, CD46, IGHV3-11, SUSD4, ENO1, NAA11, PGK1, CALR, IGLC7, CD55, NAA10, PCSK1N, TIMM17A, CASP3, PTCH1, STOML2, CAPN2, PCSK5, NCSTN, CARD8, ATP7B, PCSK9, PARP1, IGHV3-13, FGG, FXN, TSPAN33, IGLV2-14, CTSZ, CLN3, CUZD1, PMPCB, IGHV1OR15-1, IFI16, SERPINE2, IGLV1-47, CASP2, LHB, AEBP1, ANGPTL8, A2M, IGLV3-21, WFS1, IGHV1-69, KLK6, TBC1D10A, ADAMTS13, MYH9, CPZ, FGB, SERPINE1, NLRP7, GRIN2A, AFG3L2, ECE2, CPA3, BIRC7, BACE2, IGLV1-44, BOLA2B, LMF1, BCL2L12, C1QA, IGHG1, DISP1, IGLV6-57, SRC, C5, NAA16, BOLA2, IGHV4-34, F11, C7, IL1R2, PRSS12, IGHV2-5, CCS, CD19, CLU, TNP2, IGHV3-23, CCBE1, CHCHD4 | 80 |

---


---


---

# Cluster 6

| Cluster | Term | Name |
| --- | --- | --- |
| 6 | HP:0000431 | Wide nasal bridge |
| 6 | HP:0000248 | Brachycephaly |
| 6 | HP:0000307 | Pointed chin |
| 6 | HP:0009890 | High anterior hairline |

| Cluster | Term | Name | Genes | Percentage\_of\_nodes\_with\_funsys |
| --- | --- | --- | --- | --- |
| 6 | GO:2000181 | negative regulation of blood vessel morphogenesis | MECP2, PDCD10, MIR16-2, MIR23B, TEK, MIR497, AGT, DAB2IP, MIR34A, MIR20B, MIR92A2, MIR424, HHEX, MIR15B, CNMD, MIR222, MIR7-2, MIR410, PML, KLK3, ANGPT4, ANGPT2, STARD13, FOXO4, ATF2, MIR214, THBS2, EPN2, MIR30B, TGFB2, MIR221, E2F2, MIR21, MIR503, TNMD, MIR185, HOXA5, HRG, STAT1, MIR19B1, FOXC1, MIR137, SEMA4A, SARS, GTF2I, MIR377, MIR106B, RGCC, MIR212, MIR342, KRIT1, MMRN2, TBXA2R, MIR92A1, MIR329-1, PGK1, MIR18A, MIR200B, MIR487B, FASLG, NOTCH1, MIR17, MIR19A, MINAR1, MIR2355, WNT4, MIR7-3, MIR329-2, MIR188, MIR505, SPRY2, COL4A2, MIR24-1, MIR495, MIR20A, AMOT, MIR22, CREB3L1, SERPINF1, ADGRB2, MIR16-1, MIR361, MIR494, MIR29C, SERPINE1, PTN, NPPB, HDAC5, MIR19B2, HSPG2, MIR939, MIR15A, EPHA2 | 75 |
| 6 | GO:0016525 | negative regulation of angiogenesis | MECP2, PDCD10, MIR16-2, MIR23B, TEK, MIR497, AGT, DAB2IP, MIR34A, MIR20B, MIR92A2, MIR424, HHEX, MIR15B, CNMD, MIR222, MIR7-2, MIR410, PML, KLK3, ANGPT4, ANGPT2, STARD13, FOXO4, ATF2, MIR214, THBS2, EPN2, MIR30B, TGFB2, MIR221, E2F2, MIR21, MIR503, TNMD, MIR185, HOXA5, HRG, STAT1, MIR19B1, FOXC1, MIR137, SEMA4A, SARS, GTF2I, MIR377, MIR106B, RGCC, MIR212, MIR342, KRIT1, MMRN2, TBXA2R, MIR92A1, MIR329-1, PGK1, MIR18A, MIR200B, MIR487B, FASLG, NOTCH1, MIR17, MIR19A, MINAR1, MIR2355, MIR7-3, MIR329-2, MIR188, MIR505, SPRY2, COL4A2, MIR24-1, MIR495, MIR20A, AMOT, MIR22, CREB3L1, SERPINF1, ADGRB2, MIR16-1, MIR361, MIR494, MIR29C, SERPINE1, PTN, NPPB, HDAC5, MIR19B2, HSPG2, MIR939, MIR15A, EPHA2 | 75 |

---


---


---

# Cluster 20

| Cluster | Term | Name |
| --- | --- | --- |
| 20 | HP:0000077 | Abnormality of the kidney |
| 20 | HP:0000130 | Abnormality of the uterus |
| 20 | HP:0002023 | Anal atresia |
| 20 | HP:0000107 | Renal cyst |

| Cluster | Term | Name | Genes | Percentage\_of\_nodes\_with\_funsys |
| --- | --- | --- | --- | --- |
| 20 | GO:0034754 | cellular hormone metabolic process | UGT1A8, SHH, DHRS3, AKR1C3, CYP11A1, SRD5A1, TSPOAP1, UGT1A7, CYP1A1, BCO1, UGT1A1, DHRS9, SCPEP1, HSD17B2, CRABP2, HSD17B3, UGT1A3, WNT4, RDH5, SULT1A1, COMT, STARD3NL, UGT1A9, AKR1C2, DHRS11, AKR1C4, ECE1, AKR1C1, RBP1, AKR1D1 | 75 |
| 20 | GO:0006694 | steroid biosynthetic process | IDI1, AKR1B1, PRKAG2, MIR182, AKR1C3, CYB5R3, CYP11A1, SREBF2, LEP, TSPO, MIR96, IDI2, MIR185, ACACA, MALRD1, AKR1B15, MIR183, INSIG1, DHRS11, AKR1C4, MIR33A, AKR1D1 | 75 |

---


---


---

# Cluster 38

| Cluster | Term | Name |
| --- | --- | --- |
| 38 | HP:0000047 | Hypospadias |
| 38 | HP:0000110 | Renal dysplasia |
| 38 | HP:0002023 | Anal atresia |
| 38 | HP:0009929 | Abnormality of the columella |

| Cluster | Term | Name | Genes | Percentage\_of\_nodes\_with\_funsys |
| --- | --- | --- | --- | --- |
| 38 | GO:0031670 | cellular response to nutrient | TNKS, HMOX1, PDXP, BRIP1, XBP1, CASTOR1, CASR, PTN, TRIM24, P2RY11, KANK2 | 75 |

---


---


---

# Cluster 39

| Cluster | Term | Name |
| --- | --- | --- |
| 39 | HP:0000286 | Epicanthus |
| 39 | HP:0001540 | Diastasis recti |
| 39 | HP:0000189 | Narrow palate |
| 39 | HP:0005326 | Hypoplastic philtrum |

| Cluster | Term | Name | Genes | Percentage\_of\_nodes\_with\_funsys |
| --- | --- | --- | --- | --- |
| 39 | GO:0046717 | acid secretion | PLA2G4A, TNFSF11, TRPC4, SLC1A3, PLA2G2F, SNX10, PTGER3, SLC1A1, PLA2G5, SLC9A4, PLA2G2A, PLA2G2C, PLA2G2E, AGXT, GIPC1, PLA2G2D, ANXA1, PLA2G10, SLC51A, APBA1, DRD3, SLC1A6, ABCB4, MIR33A | 75 |

---


---


---

# Cluster 40

| Cluster | Term | Name |
| --- | --- | --- |
| 40 | HP:0000414 | Bulbous nose |
| 40 | HP:0000411 | Protruding ear |
| 40 | HP:0001047 | Atopic dermatitis |
| 40 | HP:0009623 | Proximal placement of thumb |

| Cluster | Term | Name | Genes | Percentage\_of\_nodes\_with\_funsys |
| --- | --- | --- | --- | --- |
| 40 | GO:0006473 | protein acetylation | NAT8B, POLE4, NAA15, BRD1, MECP2, NAA60, KANSL1, CREBBP, CDY1, NAA50, CTCF, NAA10, HCFC1, ING3, ATG5, CDY1B, JADE2, MAPT, NAT8, CDY2B, CDY2A, BEND3 | 75 |
| 40 | GO:0043543 | protein acylation | NAT8B, POLE4, NAA15, BRD1, MECP2, NAA60, KANSL1, CREBBP, CDY1, NAA50, CTCF, NAA10, HCFC1, ZDHHC14, ING3, ZDHHC23, ATG5, CDY1B, JADE2, MAPT, NAT8, CDY2B, CDY2A, BEND3 | 75 |
| 40 | GO:0006475 | internal protein amino acid acetylation | NAT8B, POLE4, BRD1, MECP2, NAA60, KANSL1, CREBBP, CDY1, NAA50, CTCF, NAA10, HCFC1, ING3, ATG5, CDY1B, JADE2, MAPT, NAT8, CDY2B, CDY2A, BEND3 | 75 |

---


---


---

# Cluster 41

| Cluster | Term | Name |
| --- | --- | --- |
| 41 | HP:0000581 | Blepharophimosis |
| 41 | HP:0001845 | Overlapping toe |
| 41 | HP:0000506 | Telecanthus |
| 41 | HP:0002208 | Coarse hair |

| Cluster | Term | Name | Genes | Percentage\_of\_nodes\_with\_funsys |
| --- | --- | --- | --- | --- |
| 41 | GO:0101023 | vascular endothelial cell proliferation | APLN, MIR499A, MIR129-1, GHSR, SIRT6, MIR30B, GHRL, MIR34A, MIR20B, MIR329-1, MIR503, MIR21, MIR424, MIR98, MIR15B, MIR487B, PDPK1, FGF2, ITGA4, MIR329-2, HMGB1, MIR126, FLT1, MIR29A, MIR24-1, AKT3, MIR495 | 75 |
| 41 | GO:0060674 | placenta blood vessel development | FBXW8, HEY1, HES1, WNT2, MIR16-2, MIR16-1, AKT1, PKD1, JUNB | 75 |
| 41 | GO:0007009 | plasma membrane organization | CAV1, ANO7, PLSCR5, BIN1, CAV2, PLSCR2, PLSCR1, MICALL1, CAV3, WASL, SNX33, SERP1, CLU, CXCR4, PACSIN2, AKT1, A4GALT, PLSCR4, BAIAP2L2, CRB1 | 75 |
| 41 | GO:1905562 | regulation of vascular endothelial cell proliferation | APLN, MIR499A, MIR129-1, GHSR, SIRT6, MIR30B, GHRL, MIR34A, MIR20B, MIR329-1, MIR503, MIR21, MIR424, MIR98, MIR15B, MIR487B, PDPK1, FGF2, ITGA4, MIR329-2, HMGB1, MIR126, FLT1, MIR29A, MIR24-1, AKT3, MIR495 | 75 |

---


---


---

# Cluster 59

| Cluster | Term | Name |
| --- | --- | --- |
| 59 | HP:0000358 | Posteriorly rotated ears |
| 59 | HP:0001249 | Intellectual disability |
| 59 | HP:0001518 | Small for gestational age |
| 59 | HP:0010490 | Abnormality of the palmar creases |

| Cluster | Term | Name | Genes | Percentage\_of\_nodes\_with\_funsys |
| --- | --- | --- | --- | --- |
| 59 | GO:0008202 | steroid metabolic process | IDI1, AKR1B1, LGMN, UGT1A8, SHH, PRKAG2, MIR182, AKR1C3, HDLBP, CYB5R3, SULT1A2, CYP11A1, TSPOAP1, CFTR, SREBF2, TSPO, MIR96, CYP1A1, PON1, SULT1A4, WWOX, CUBN, IDI2, MBTPS1, PROX1, CYB5R1, CYP2D6, MIR30C2, UGT1A1, ABCB11, DHRS9, MIR185, ACACA, ACADVL, HSD17B2, SULT4A1, MALRD1, HSD11B1, AKR1B15, CYP46A1, TTC39B, CYP3A7, HSD17B3, SOAT1, VLDLR, CYP3A5, LRP2, SP1, SULT1A1, COMT, STARD3NL, MIR183, APOBR, AKR1C2, INSIG1, SDR42E1, DHRS11, AKR1C4, MIR33A, AKR1C1, CYP1A2, NR5A2, AKR1C8P, AKR1D1, CYP3A4, BAAT | 75 |

---


---


---

# Cluster 79

| Cluster | Term | Name |
| --- | --- | --- |
| 79 | HP:0000568 | Microphthalmia |
| 79 | HP:0003272 | Abnormality of the hip bone |
| 79 | HP:0010722 | Asymmetry of the ears |
| 79 | HP:0010609 | Skin tags |

| Cluster | Term | Name | Genes | Percentage\_of\_nodes\_with\_funsys |
| --- | --- | --- | --- | --- |
| 79 | GO:0010874 | regulation of cholesterol efflux | GPS2, MIR27B, TTC39B, MIR302A, PTCH1, MIR19B1, SREBF2, MIR130B, MIR301B, MIR33A, MIR758 | 75 |
| 79 | GO:0032375 | negative regulation of cholesterol transport | MIR302A, ABCG8, ABCG5, SREBF2, MIR130B, MIR301B, MIR33A, MIR148A | 75 |
| 79 | GO:0032372 | negative regulation of sterol transport | MIR302A, ABCG8, ABCG5, SREBF2, MIR130B, MIR301B, MIR33A, MIR148A | 75 |

---


---


---

# Cluster 81

| Cluster | Term | Name |
| --- | --- | --- |
| 81 | HP:0001172 | Abnormal thumb morphology |
| 81 | HP:0001385 | Hip dysplasia |
| 81 | HP:0002827 | Hip dislocation |
| 81 | HP:0003019 | Abnormality of the wrist |

| Cluster | Term | Name | Genes | Percentage\_of\_nodes\_with\_funsys |
| --- | --- | --- | --- | --- |
| 81 | GO:0016180 | snRNA processing | CT45A8, CT45A10, SAGE1, EXOSC8, INTS9, INTS6L, CT45A3, INTS7, EXOSC4, CT45A1, INTS2, INTS10, INTS1, INTS8, CT45A9, EXOSC9, CT45A5, INTS6 | 75 |
| 81 | GO:0043628 | ncRNA 3’-end processing | CT45A3, INTS7, CT45A1, INTS2, SAGE1, CT45A10, INTS1, SSB, CT45A9, INTS8, CT45A5, TRNT1, CT45A8, ERI1, RPS21, TENT4A, EXOSC4, EXOSC8, INTS6L, DKC1, HSD17B10, PARN, EXOSC9, INTS6 | 75 |
| 81 | GO:0034472 | snRNA 3’-end processing | CT45A8, CT45A10, SAGE1, EXOSC8, INTS6L, CT45A3, INTS7, EXOSC4, CT45A1, INTS2, INTS1, INTS8, CT45A9, EXOSC9, CT45A5, INTS6 | 75 |

---


---


---

# Cluster 3

| Cluster | Term | Name |
| --- | --- | --- |
| 3 | HP:0002086 | Abnormality of the respiratory system |
| 3 | HP:0003011 | Abnormality of the musculature |
| 3 | HP:0003549 | Abnormality of connective tissue |

| Cluster | Term | Name | HPOs\_in\_clusters |
| --- | --- | --- | --- |
| 3 | OMIM:236800 | HYDROXYKYNURENINURIA | HP:0003011, HP:0002086 |

---


---


---

# Cluster 5

| Cluster | Term | Name |
| --- | --- | --- |
| 5 | HP:0000248 | Brachycephaly |
| 5 | HP:0000307 | Pointed chin |
| 5 | HP:0009890 | High anterior hairline |

| Cluster | Term | Name | HPOs\_in\_clusters |
| --- | --- | --- | --- |
| 5 | OMIM:117550 | SOTOS SYNDROME 1; SOTOS1 | HP:0009890, HP:0000307 |
| 5 | OMIM:616078 | MENTAL RETARDATION, AUTOSOMAL DOMINANT 29; MRD29 | HP:0000307, HP:0000248 |
| 5 | OMIM:616364 | WHITE-SUTTON SYNDROME; WHSUS | HP:0000307, HP:0000248 |
| 5 | OMIM:616831 | LUSCAN-LUMISH SYNDROME; LLS | HP:0009890, HP:0000307 |

---


---


---

# Cluster 13

| Cluster | Term | Name |
| --- | --- | --- |
| 13 | HP:0000286 | Epicanthus |
| 13 | HP:0000954 | Single transverse palmar crease |
| 13 | HP:0004209 | Clinodactyly of the 5th finger |
| 13 | HP:0000470 | Short neck |

| Cluster | Term | Name | HPOs\_in\_clusters |
| --- | --- | --- | --- |
| 13 | OMIM:113620 | BRANCHIOOCULOFACIAL SYNDROME; BOFS | HP:0000954, HP:0000470, HP:0004209 |
| 13 | OMIM:114300 | ARTHROGRYPOSIS, DISTAL, TYPE 3; DA3 | HP:0000954, HP:0000470, HP:0000286 |
| 13 | OMIM:122470 | CORNELIA DE LANGE SYNDROME 1; CDLS1 | HP:0000954, HP:0000470, HP:0004209 |
| 13 | OMIM:180849 | RUBINSTEIN-TAYBI SYNDROME 1; RSTS1 | HP:0000954, HP:0000286, HP:0004209 |
| 13 | OMIM:201000 | CARPENTER SYNDROME 1; CRPT1 | HP:0000470, HP:0000286, HP:0004209 |
| 13 | OMIM:261540 | PETERS-PLUS SYNDROME; PTRPLS | HP:0000954, HP:0000470, HP:0004209 |
| 13 | OMIM:300209 | SIMPSON-GOLABI-BEHMEL SYNDROME, TYPE 2; SGBS2 | HP:0000954, HP:0000470, HP:0000286 |
| 13 | OMIM:305450 | OPITZ-KAVEGGIA SYNDROME; OKS | HP:0000954, HP:0000470, HP:0000286 |
| 13 | OMIM:616145 | CATEL-MANZKE SYNDROME; CATMANS | HP:0000954, HP:0000470, HP:0004209 |

---


---


---

# Cluster 14

| Cluster | Term | Name |
| --- | --- | --- |
| 14 | HP:0000286 | Epicanthus |
| 14 | HP:0000343 | Long philtrum |
| 14 | HP:0000369 | Low-set ears |
| 14 | HP:0000954 | Single transverse palmar crease |
| 14 | HP:0004209 | Clinodactyly of the 5th finger |

| Cluster | Term | Name | HPOs\_in\_clusters |
| --- | --- | --- | --- |
| 14 | OMIM:117650 | CEREBROCOSTOMANDIBULAR SYNDROME; CCMS | HP:0000369, HP:0000286, HP:0000343, HP:0004209 |
| 14 | OMIM:122470 | CORNELIA DE LANGE SYNDROME 1; CDLS1 | HP:0000954, HP:0000369, HP:0000343, HP:0004209 |
| 14 | OMIM:180849 | RUBINSTEIN-TAYBI SYNDROME 1; RSTS1 | HP:0000954, HP:0000369, HP:0000286, HP:0004209 |
| 14 | OMIM:261540 | PETERS-PLUS SYNDROME; PTRPLS | HP:0000954, HP:0000369, HP:0000343, HP:0004209 |
| 14 | OMIM:605130 | WIEDEMANN-STEINER SYNDROME; WDSTS | HP:0000369, HP:0000286, HP:0000343, HP:0004209 |

---


---


---

# Cluster 17

| Cluster | Term | Name |
| --- | --- | --- |
| 17 | HP:0000218 | High palate |
| 17 | HP:0000219 | Thin upper lip vermilion |
| 17 | HP:0000343 | Long philtrum |
| 17 | HP:0000463 | Anteverted nares |
| 17 | HP:0002007 | Frontal bossing |

| Cluster | Term | Name | HPOs\_in\_clusters |
| --- | --- | --- | --- |
| 17 | OMIM:122470 | CORNELIA DE LANGE SYNDROME 1; CDLS1 | HP:0000463, HP:0000218, HP:0000343, HP:0000219 |
| 17 | OMIM:166250 | OSTEOGLOPHONIC DYSPLASIA; OGD | HP:0002007, HP:0000463, HP:0000218, HP:0000343 |
| 17 | OMIM:180700 | ROBINOW SYNDROME, AUTOSOMAL DOMINANT 1; DRS1 | HP:0002007, HP:0000463, HP:0000343, HP:0000219 |
| 17 | OMIM:211380 | ELSAHY-WATERS SYNDROME; ESWS | HP:0000463, HP:0000218, HP:0000343, HP:0000219 |
| 17 | OMIM:219200 | CUTIS LAXA, AUTOSOMAL RECESSIVE, TYPE IIA; ARCL2A | HP:0002007, HP:0000463, HP:0000218, HP:0000343 |
| 17 | OMIM:268310 | ROBINOW SYNDROME, AUTOSOMAL RECESSIVE 1; RRS1 | HP:0002007, HP:0000463, HP:0000343, HP:0000219 |
| 17 | OMIM:269921 | SIALURIA | HP:0002007, HP:0000218, HP:0000343, HP:0000219 |
| 17 | OMIM:300000 | OPITZ GBBB SYNDROME, TYPE I; GBBB1 | HP:0002007, HP:0000463, HP:0000218, HP:0000219 |
| 17 | OMIM:607812 | CRANIOLENTICULOSUTURAL DYSPLASIA; CLSD | HP:0002007, HP:0000463, HP:0000343, HP:0000219 |
| 17 | OMIM:614080 | MULTIPLE CONGENITAL ANOMALIES-HYPOTONIA-SEIZURES SYNDROME 1; MCAHS1 | HP:0002007, HP:0000463, HP:0000218, HP:0000343 |
| 17 | OMIM:616331 | ROBINOW SYNDROME, AUTOSOMAL DOMINANT 2; DRS2 | HP:0002007, HP:0000463, HP:0000343, HP:0000219 |
| 17 | OMIM:617877 | SHORT STATURE, FACIAL DYSMORPHISM, AND SKELETAL ANOMALIES WITH OR WITHOUT CARDIAC ANOMALIES; SSFSC | HP:0000463, HP:0000218, HP:0000343, HP:0000219 |

---


---


---

# Cluster 26

| Cluster | Term | Name |
| --- | --- | --- |
| 26 | HP:0001631 | Atrial septal defect |
| 26 | HP:0000589 | Coloboma |
| 26 | HP:0008070 | Sparse hair |
| 26 | HP:0010438 | Abnormal ventricular septum morphology |

| Cluster | Term | Name | HPOs\_in\_clusters |
| --- | --- | --- | --- |
| 26 | OMIM:268300 | ROBERTS SYNDROME; RBS | HP:0000589, HP:0001631, HP:0008070 |
| 26 | OMIM:309500 | RENPENNING SYNDROME 1; RENS1 | HP:0000589, HP:0001631, HP:0008070 |

---


---


---

# Cluster 30

| Cluster | Term | Name |
| --- | --- | --- |
| 30 | HP:0000431 | Wide nasal bridge |
| 30 | HP:0000322 | Short philtrum |
| 30 | HP:0000377 | Abnormality of the pinna |
| 30 | HP:0002714 | Downturned corners of mouth |

| Cluster | Term | Name | HPOs\_in\_clusters |
| --- | --- | --- | --- |
| 30 | OMIM:194190 | WOLF-HIRSCHHORN SYNDROME; WHS | HP:0000322, HP:0000377, HP:0002714, HP:0000431 |
| 30 | OMIM:617333 | INTELLECTUAL DEVELOPMENTAL DISORDER WITH DYSMORPHIC FACIES AND PTOSIS; IDDDFP | HP:0000322, HP:0000377, HP:0002714, HP:0000431 |

---


---


---

# Cluster 37

| Cluster | Term | Name |
| --- | --- | --- |
| 37 | HP:0000252 | Microcephaly |
| 37 | HP:0000377 | Abnormality of the pinna |
| 37 | HP:0008872 | Feeding difficulties in infancy |
| 37 | HP:0002164 | Nail dysplasia |

| Cluster | Term | Name | HPOs\_in\_clusters |
| --- | --- | --- | --- |
| 37 | OMIM:180849 | RUBINSTEIN-TAYBI SYNDROME 1; RSTS1 | HP:0000377, HP:0008872, HP:0000252 |
| 37 | OMIM:305600 | FOCAL DERMAL HYPOPLASIA; FDH | HP:0000377, HP:0000252, HP:0002164 |

---


---


---

# Cluster 43

| Cluster | Term | Name |
| --- | --- | --- |
| 43 | HP:0002360 | Sleep disturbance |
| 43 | HP:0000718 | Aggressive behavior |
| 43 | HP:0000729 | Autistic behavior |
| 43 | HP:0010864 | Intellectual disability, severe |

| Cluster | Term | Name | HPOs\_in\_clusters |
| --- | --- | --- | --- |
| 43 | OMIM:610253 | KLEEFSTRA SYNDROME 1; KLEFS1 | HP:0000718, HP:0002360, HP:0010864 |

---


---


---

# Cluster 49

| Cluster | Term | Name |
| --- | --- | --- |
| 49 | HP:0000219 | Thin upper lip vermilion |
| 49 | HP:0000343 | Long philtrum |
| 49 | HP:0000369 | Low-set ears |
| 49 | HP:0000581 | Blepharophimosis |
| 49 | HP:0000954 | Single transverse palmar crease |
| 49 | HP:0004209 | Clinodactyly of the 5th finger |
| 49 | HP:0001773 | Short foot |

| Cluster | Term | Name | HPOs\_in\_clusters |
| --- | --- | --- | --- |
| 49 | OMIM:261540 | PETERS-PLUS SYNDROME; PTRPLS | HP:0000219, HP:0004209, HP:0000954, HP:0000369, HP:0000343, HP:0001773 |

---


---


---

# Cluster 50

| Cluster | Term | Name |
| --- | --- | --- |
| 50 | HP:0000219 | Thin upper lip vermilion |
| 50 | HP:0000463 | Anteverted nares |
| 50 | HP:0001156 | Brachydactyly |
| 50 | HP:0000343 | Long philtrum |
| 50 | HP:0001773 | Short foot |
| 50 | HP:0004209 | Clinodactyly of the 5th finger |

| Cluster | Term | Name | HPOs\_in\_clusters |
| --- | --- | --- | --- |
| 50 | OMIM:261540 | PETERS-PLUS SYNDROME; PTRPLS | HP:0000219, HP:0004209, HP:0001156, HP:0000343, HP:0001773 |

---


---


---

# Cluster 53

| Cluster | Term | Name |
| --- | --- | --- |
| 53 | HP:0001518 | Small for gestational age |
| 53 | HP:0003508 | Proportionate short stature |
| 53 | HP:0000369 | Low-set ears |
| 53 | HP:0004209 | Clinodactyly of the 5th finger |

| Cluster | Term | Name | HPOs\_in\_clusters |
| --- | --- | --- | --- |
| 53 | OMIM:210600 | SECKEL SYNDROME 1; SCKL1 | HP:0003508, HP:0000369, HP:0004209 |
| 53 | OMIM:224690 | MEIER-GORLIN SYNDROME 1; MGORS1 | HP:0001518, HP:0000369, HP:0004209 |

---


---


---

# Cluster 58

| Cluster | Term | Name |
| --- | --- | --- |
| 58 | HP:0000219 | Thin upper lip vermilion |
| 58 | HP:0000463 | Anteverted nares |
| 58 | HP:0000218 | High palate |
| 58 | HP:0000286 | Epicanthus |
| 58 | HP:0000343 | Long philtrum |
| 58 | HP:0000954 | Single transverse palmar crease |

| Cluster | Term | Name | HPOs\_in\_clusters |
| --- | --- | --- | --- |
| 58 | OMIM:122470 | CORNELIA DE LANGE SYNDROME 1; CDLS1 | HP:0000219, HP:0000463, HP:0000218, HP:0000954, HP:0000343 |
| 58 | OMIM:300209 | SIMPSON-GOLABI-BEHMEL SYNDROME, TYPE 2; SGBS2 | HP:0000219, HP:0000463, HP:0000218, HP:0000286, HP:0000954 |
| 58 | OMIM:613406 | WITTEVEEN-KOLK SYNDROME; WITKOS | HP:0000463, HP:0000218, HP:0000286, HP:0000954, HP:0000343 |

---


---


---

# Cluster 62

| Cluster | Term | Name |
| --- | --- | --- |
| 62 | HP:0000218 | High palate |
| 62 | HP:0000286 | Epicanthus |
| 62 | HP:0000358 | Posteriorly rotated ears |
| 62 | HP:0000463 | Anteverted nares |
| 62 | HP:0004209 | Clinodactyly of the 5th finger |
| 62 | HP:0000470 | Short neck |

| Cluster | Term | Name | HPOs\_in\_clusters |
| --- | --- | --- | --- |
| 62 | OMIM:115150 | CARDIOFACIOCUTANEOUS SYNDROME 1; CFC1 | HP:0000358, HP:0000218, HP:0000463, HP:0000286, HP:0004209 |
| 62 | OMIM:218040 | COSTELLO SYNDROME; CSTLO | HP:0000358, HP:0000218, HP:0000470, HP:0000286, HP:0000463 |
| 62 | OMIM:300209 | SIMPSON-GOLABI-BEHMEL SYNDROME, TYPE 2; SGBS2 | HP:0000358, HP:0000218, HP:0000470, HP:0000286, HP:0000463 |
| 62 | OMIM:309580 | MENTAL RETARDATION-HYPOTONIC FACIES SYNDROME, X-LINKED, 1; MRXHF1 | HP:0000358, HP:0000218, HP:0000470, HP:0000286, HP:0000463 |

---


---


---

# Cluster 66

| Cluster | Term | Name |
| --- | --- | --- |
| 66 | HP:0000347 | Micrognathia |
| 66 | HP:0000369 | Low-set ears |
| 66 | HP:0000463 | Anteverted nares |
| 66 | HP:0000692 | Misalignment of teeth |

| Cluster | Term | Name | HPOs\_in\_clusters |
| --- | --- | --- | --- |
| 66 | OMIM:102500 | HAJDU-CHENEY SYNDROME; HJCYS | HP:0000463, HP:0000347, HP:0000369 |
| 66 | OMIM:115150 | CARDIOFACIOCUTANEOUS SYNDROME 1; CFC1 | HP:0000463, HP:0000347, HP:0000369 |
| 66 | OMIM:122470 | CORNELIA DE LANGE SYNDROME 1; CDLS1 | HP:0000463, HP:0000347, HP:0000369 |
| 66 | OMIM:154780 | MARSHALL SYNDROME; MRSHS | HP:0000463, HP:0000347, HP:0000369 |
| 66 | OMIM:164280 | FEINGOLD SYNDROME 1; FGLDS1 | HP:0000463, HP:0000347, HP:0000369 |
| 66 | OMIM:211750 | C SYNDROME | HP:0000463, HP:0000347, HP:0000369 |
| 66 | OMIM:215140 | GREENBERG DYSPLASIA; GRBGD | HP:0000692, HP:0000347, HP:0000369 |
| 66 | OMIM:216340 | YUNIS-VARON SYNDROME; YVS | HP:0000463, HP:0000347, HP:0000369 |
| 66 | OMIM:218040 | COSTELLO SYNDROME; CSTLO | HP:0000463, HP:0000347, HP:0000369 |
| 66 | OMIM:242860 | IMMUNODEFICIENCY-CENTROMERIC INSTABILITY-FACIAL ANOMALIES SYNDROME 1; ICF1 | HP:0000463, HP:0000347, HP:0000369 |
| 66 | OMIM:248700 | MARDEN-WALKER SYNDROME; MWKS | HP:0000463, HP:0000347, HP:0000369 |
| 66 | OMIM:257300 | MOSAIC VARIEGATED ANEUPLOIDY SYNDROME 1; MVA1 | HP:0000463, HP:0000347, HP:0000369 |
| 66 | OMIM:270400 | SMITH-LEMLI-OPITZ SYNDROME; SLOS | HP:0000463, HP:0000347, HP:0000369 |
| 66 | OMIM:272430 | CRISPONI/COLD-INDUCED SWEATING SYNDROME 1; CISS1 | HP:0000463, HP:0000347, HP:0000369 |
| 66 | OMIM:309580 | MENTAL RETARDATION-HYPOTONIC FACIES SYNDROME, X-LINKED, 1; MRXHF1 | HP:0000463, HP:0000347, HP:0000369 |
| 66 | OMIM:311900 | TARP SYNDROME; TARPS | HP:0000463, HP:0000347, HP:0000369 |
| 66 | OMIM:314580 | WIEACKER-WOLFF SYNDROME; WRWF | HP:0000463, HP:0000347, HP:0000369 |
| 66 | OMIM:608013 | GAUCHER DISEASE, PERINATAL LETHAL | HP:0000463, HP:0000347, HP:0000369 |
| 66 | OMIM:610536 | MANDIBULOFACIAL DYSOSTOSIS, GUION-ALMEIDA TYPE; MFDGA | HP:0000463, HP:0000347, HP:0000369 |
| 66 | OMIM:614080 | MULTIPLE CONGENITAL ANOMALIES-HYPOTONIA-SEIZURES SYNDROME 1; MCAHS1 | HP:0000463, HP:0000347, HP:0000369 |
| 66 | OMIM:616503 | LETHAL CONGENITAL CONTRACTURE SYNDROME 9; LCCS9 | HP:0000463, HP:0000347, HP:0000369 |
| 66 | OMIM:616897 | OSTEOCHONDRODYSPLASIA, COMPLEX LETHAL, SYMOENS-BARNES-GISTELINCK TYPE; OCLSBG | HP:0000463, HP:0000347, HP:0000369 |
| 66 | OMIM:616975 | NEURODEVELOPMENTAL DISORDER WITH OR WITHOUT ANOMALIES OF THE BRAIN, EYE, OR HEART; NEDBEH | HP:0000463, HP:0000347, HP:0000369 |
| 66 | OMIM:617062 | OKUR-CHUNG NEURODEVELOPMENTAL SYNDROME; OCNDS | HP:0000463, HP:0000347, HP:0000369 |

---


---


---

# Cluster 68

| Cluster | Term | Name |
| --- | --- | --- |
| 68 | HP:0000463 | Anteverted nares |
| 68 | HP:0000486 | Strabismus |
| 68 | HP:0000750 | Delayed speech and language development |
| 68 | HP:0001252 | Muscular hypotonia |
| 68 | HP:0002007 | Frontal bossing |
| 68 | HP:0003196 | Short nose |

| Cluster | Term | Name | HPOs\_in\_clusters |
| --- | --- | --- | --- |
| 68 | OMIM:219200 | CUTIS LAXA, AUTOSOMAL RECESSIVE, TYPE IIA; ARCL2A | HP:0003196, HP:0002007, HP:0000463, HP:0001252, HP:0000486 |
| 68 | OMIM:613406 | WITTEVEEN-KOLK SYNDROME; WITKOS | HP:0000750, HP:0003196, HP:0000463, HP:0001252, HP:0000486 |

---


---


---

# Cluster 76

| Cluster | Term | Name |
| --- | --- | --- |
| 76 | HP:0000343 | Long philtrum |
| 76 | HP:0003196 | Short nose |
| 76 | HP:0000347 | Micrognathia |
| 76 | HP:0005280 | Depressed nasal bridge |

| Cluster | Term | Name | HPOs\_in\_clusters |
| --- | --- | --- | --- |
| 76 | OMIM:154780 | MARSHALL SYNDROME; MRSHS | HP:0000347, HP:0005280, HP:0000343, HP:0003196 |
| 76 | OMIM:257300 | MOSAIC VARIEGATED ANEUPLOIDY SYNDROME 1; MVA1 | HP:0000347, HP:0005280, HP:0000343, HP:0003196 |
| 76 | OMIM:258315 | OMODYSPLASIA 1; OMOD1 | HP:0000347, HP:0005280, HP:0000343, HP:0003196 |
| 76 | OMIM:612289 | FONTAINE PROGEROID SYNDROME; FPS | HP:0000347, HP:0005280, HP:0000343, HP:0003196 |
| 76 | OMIM:614080 | MULTIPLE CONGENITAL ANOMALIES-HYPOTONIA-SEIZURES SYNDROME 1; MCAHS1 | HP:0000347, HP:0005280, HP:0000343, HP:0003196 |
| 76 | OMIM:616894 | ROBINOW SYNDROME, AUTOSOMAL DOMINANT 3; DRS3 | HP:0000347, HP:0005280, HP:0000343, HP:0003196 |
| 76 | OMIM:108720 | ATELOSTEOGENESIS, TYPE I; AO1 | HP:0000347, HP:0005280, HP:0003196 |
| 76 | OMIM:115150 | CARDIOFACIOCUTANEOUS SYNDROME 1; CFC1 | HP:0000347, HP:0005280, HP:0003196 |
| 76 | OMIM:122470 | CORNELIA DE LANGE SYNDROME 1; CDLS1 | HP:0000347, HP:0005280, HP:0000343 |
| 76 | OMIM:145420 | HYPERTELORISM, TEEBI TYPE; TBHS | HP:0005280, HP:0000343, HP:0003196 |
| 76 | OMIM:166250 | OSTEOGLOPHONIC DYSPLASIA; OGD | HP:0005280, HP:0000343, HP:0003196 |
| 76 | OMIM:194050 | WILLIAMS-BEUREN SYNDROME; WBS | HP:0005280, HP:0000343, HP:0003196 |
| 76 | OMIM:228520 | FIBROCHONDROGENESIS 1; FBCG1 | HP:0005280, HP:0000343, HP:0003196 |
| 76 | OMIM:241410 | HYPOPARATHYROIDISM-RETARDATION-DYSMORPHISM SYNDROME; HRDS | HP:0000347, HP:0005280, HP:0000343 |
| 76 | OMIM:242860 | IMMUNODEFICIENCY-CENTROMERIC INSTABILITY-FACIAL ANOMALIES SYNDROME 1; ICF1 | HP:0000347, HP:0005280, HP:0003196 |
| 76 | OMIM:258480 | OPSISMODYSPLASIA; OPSMD | HP:0005280, HP:0000343, HP:0003196 |
| 76 | OMIM:259775 | RAINE SYNDROME; RNS | HP:0000347, HP:0005280, HP:0003196 |
| 76 | OMIM:261515 | D-BIFUNCTIONAL PROTEIN DEFICIENCY | HP:0000347, HP:0005280, HP:0000343 |
| 76 | OMIM:270400 | SMITH-LEMLI-OPITZ SYNDROME; SLOS | HP:0000347, HP:0005280, HP:0000343 |
| 76 | OMIM:271665 | SPONDYLOMETAEPIPHYSEAL DYSPLASIA, SHORT LIMB-HAND TYPE | HP:0000347, HP:0005280, HP:0003196 |
| 76 | OMIM:272430 | CRISPONI/COLD-INDUCED SWEATING SYNDROME 1; CISS1 | HP:0000347, HP:0005280, HP:0000343 |
| 76 | OMIM:300749 | MENTAL RETARDATION AND MICROCEPHALY WITH PONTINE AND CEREBELLAR HYPOPLASIA; MICPCH | HP:0000347, HP:0000343, HP:0003196 |
| 76 | OMIM:300895 | OHDO SYNDROME, X-LINKED; OHDOX | HP:0000347, HP:0005280, HP:0000343 |
| 76 | OMIM:301022 | NEURODEVELOPMENTAL DISORDER, X-LINKED, WITH CRANIOFACIAL ABNORMALITIES; NEDXCF | HP:0000347, HP:0005280, HP:0000343 |
| 76 | OMIM:605309 | MACROCEPHALY/AUTISM SYNDROME | HP:0005280, HP:0000343, HP:0003196 |
| 76 | OMIM:608013 | GAUCHER DISEASE, PERINATAL LETHAL | HP:0000347, HP:0005280, HP:0003196 |
| 76 | OMIM:608022 | DIAPHANOSPONDYLODYSOSTOSIS | HP:0000347, HP:0005280, HP:0003196 |
| 76 | OMIM:608776 | CONGENITAL DISORDER OF GLYCOSYLATION, TYPE Il; CDG1L | HP:0005280, HP:0000343, HP:0003196 |
| 76 | OMIM:613406 | WITTEVEEN-KOLK SYNDROME; WITKOS | HP:0005280, HP:0000343, HP:0003196 |
| 76 | OMIM:614105 | METHYLMALONATE SEMIALDEHYDE DEHYDROGENASE DEFICIENCY; MMSDHD | HP:0005280, HP:0000343, HP:0003196 |
| 76 | OMIM:615398 | MULTIPLE CONGENITAL ANOMALIES-HYPOTONIA-SEIZURES SYNDROME 3; MCAHS3 | HP:0005280, HP:0000343, HP:0003196 |
| 76 | OMIM:616430 | COMBINED OXIDATIVE PHOSPHORYLATION DEFICIENCY 25; COXPD25 | HP:0005280, HP:0000343, HP:0003196 |
| 76 | OMIM:616638 | SMITH-KINGSMORE SYNDROME; SKS | HP:0005280, HP:0000343, HP:0003196 |
| 76 | OMIM:617527 | NEURODEVELOPMENTAL DISORDER WITH PROGRESSIVE MICROCEPHALY, SPASTICITY, AND BRAIN ANOMALIES; NDMSBA | HP:0000347, HP:0000343, HP:0003196 |
| 76 | OMIM:617752 | MENTAL RETARDATION, AUTOSOMAL DOMINANT 49; MRD49 | HP:0005280, HP:0000343, HP:0003196 |
| 76 | OMIM:617802 | NEURODEVELOPMENTAL DISORDER WITH MICROCEPHALY, SEIZURES, AND CORTICAL ATROPHY; NDMSCA | HP:0000347, HP:0005280, HP:0003196 |
| 76 | OMIM:617991 | DEVELOPMENTAL DELAY, INTELLECTUAL DISABILITY, OBESITY, AND DYSMORPHISM; DIDOD | HP:0000347, HP:0000343, HP:0003196 |

---


---


---

# Cluster 78

| Cluster | Term | Name |
| --- | --- | --- |
| 78 | HP:0000343 | Long philtrum |
| 78 | HP:0000058 | Abnormality of the labia |
| 78 | HP:0000233 | Thin vermilion border |
| 78 | HP:0000319 | Smooth philtrum |

| Cluster | Term | Name | HPOs\_in\_clusters |
| --- | --- | --- | --- |
| 78 | OMIM:300895 | OHDO SYNDROME, X-LINKED; OHDOX | HP:0000319, HP:0000233, HP:0000343 |
| 78 | OMIM:613406 | WITTEVEEN-KOLK SYNDROME; WITKOS | HP:0000319, HP:0000233, HP:0000343 |

---


---


---

# Cluster 83

| Cluster | Term | Name |
| --- | --- | --- |
| 83 | HP:0001622 | Premature birth |
| 83 | HP:0001838 | Rocker bottom foot |
| 83 | HP:0002020 | Gastroesophageal reflux |
| 83 | HP:0001508 | Failure to thrive |

| Cluster | Term | Name | HPOs\_in\_clusters |
| --- | --- | --- | --- |
| 83 | OMIM:115150 | CARDIOFACIOCUTANEOUS SYNDROME 1; CFC1 | HP:0001622, HP:0002020, HP:0001508 |
| 83 | OMIM:270400 | SMITH-LEMLI-OPITZ SYNDROME; SLOS | HP:0001622, HP:0002020, HP:0001508 |

---


---


---

# Cluster 4

| Cluster | Term | Name |
| --- | --- | --- |
| 4 | HP:0000598 | Abnormality of the ear |
| 4 | HP:0002118 | Abnormality of the cerebral ventricles |
| 4 | HP:0003808 | Abnormal muscle tone |

---


---


---

# Cluster 8

| Cluster | Term | Name |
| --- | --- | --- |
| 8 | HP:0000769 | Abnormality of the breast |
| 8 | HP:0000924 | Abnormality of the skeletal system |
| 8 | HP:0003549 | Abnormality of connective tissue |

---


---


---

# Cluster 11

| Cluster | Term | Name |
| --- | --- | --- |
| 11 | HP:0000478 | Abnormality of the eye |
| 11 | HP:0000598 | Abnormality of the ear |
| 11 | HP:0003011 | Abnormality of the musculature |
| 11 | HP:0003549 | Abnormality of connective tissue |

---


---


---

# Cluster 24

| Cluster | Term | Name |
| --- | --- | --- |
| 24 | HP:0001197 | Abnormality of prenatal development or birth |
| 24 | HP:0001626 | Abnormality of the cardiovascular system |
| 24 | HP:0000119 | Abnormality of the genitourinary system |
| 24 | HP:0001384 | Abnormality of the hip joint |
| 24 | HP:0012758 | Neurodevelopmental delay |

---


---


---

# Cluster 27

| Cluster | Term | Name |
| --- | --- | --- |
| 27 | HP:0002650 | Scoliosis |
| 27 | HP:0000411 | Protruding ear |
| 27 | HP:0000821 | Hypothyroidism |
| 27 | HP:0007477 | Abnormal dermatoglyphics |

---


---


---

# Cluster 33

| Cluster | Term | Name |
| --- | --- | --- |
| 33 | HP:0000422 | Abnormality of the nasal bridge |
| 33 | HP:0000436 | Abnormality of the nasal tip |
| 33 | HP:0000775 | Abnormality of the diaphragm |
| 33 | HP:0000204 | Cleft upper lip |
| 33 | HP:0002167 | Neurological speech impairment |

---


---


---

# Cluster 42

| Cluster | Term | Name |
| --- | --- | --- |
| 42 | HP:0001182 | Tapered finger |
| 42 | HP:0002069 | Generalized tonic-clonic seizures |
| 42 | HP:0000448 | Prominent nose |
| 42 | HP:0009894 | Thickened ears |

---


---


---

# Cluster 44

| Cluster | Term | Name |
| --- | --- | --- |
| 44 | HP:0002817 | Abnormality of the upper limb |
| 44 | HP:0009803 | Short phalanx of finger |
| 44 | HP:0000429 | Abnormality of the nasal alae |
| 44 | HP:0001500 | Broad finger |

---


---


---

# Cluster 48

| Cluster | Term | Name |
| --- | --- | --- |
| 48 | HP:0000286 | Epicanthus |
| 48 | HP:0000316 | Hypertelorism |
| 48 | HP:0000343 | Long philtrum |
| 48 | HP:0000463 | Anteverted nares |
| 48 | HP:0000218 | High palate |
| 48 | HP:0000369 | Low-set ears |
| 48 | HP:0000954 | Single transverse palmar crease |
| 48 | HP:0004209 | Clinodactyly of the 5th finger |

---


---


---

# Cluster 51

| Cluster | Term | Name |
| --- | --- | --- |
| 51 | HP:0001252 | Muscular hypotonia |
| 51 | HP:0000426 | Prominent nasal bridge |
| 51 | HP:0000470 | Short neck |
| 51 | HP:0001845 | Overlapping toe |
| 51 | HP:0005280 | Depressed nasal bridge |

---


---


---

# Cluster 56

| Cluster | Term | Name |
| --- | --- | --- |
| 56 | HP:0000356 | Abnormality of the outer ear |
| 56 | HP:0000426 | Prominent nasal bridge |
| 56 | HP:0001845 | Overlapping toe |
| 56 | HP:0005280 | Depressed nasal bridge |
| 56 | HP:0001182 | Tapered finger |

---


---


---

# Cluster 60

| Cluster | Term | Name |
| --- | --- | --- |
| 60 | HP:0000527 | Long eyelashes |
| 60 | HP:0000582 | Upslanted palpebral fissure |
| 60 | HP:0000506 | Telecanthus |
| 60 | HP:0001956 | Truncal obesity |

---


---


---

# Cluster 61

| Cluster | Term | Name |
| --- | --- | --- |
| 61 | HP:0000316 | Hypertelorism |
| 61 | HP:0000272 | Malar flattening |
| 61 | HP:0002353 | EEG abnormality |
| 61 | HP:0002719 | Recurrent infections |
| 61 | HP:0004411 | Deviated nasal septum |
| 61 | HP:0010554 | Cutaneous finger syndactyly |

---


---


---

# Cluster 64

| Cluster | Term | Name |
| --- | --- | --- |
| 64 | HP:0000238 | Hydrocephalus |
| 64 | HP:0000252 | Microcephaly |
| 64 | HP:0000581 | Blepharophimosis |
| 64 | HP:0001371 | Flexion contracture |
| 64 | HP:0000290 | Abnormality of the forehead |
| 64 | HP:0100807 | Long fingers |

---


---


---

# Cluster 67

| Cluster | Term | Name |
| --- | --- | --- |
| 67 | HP:0000219 | Thin upper lip vermilion |
| 67 | HP:0000286 | Epicanthus |
| 67 | HP:0000343 | Long philtrum |
| 67 | HP:0000431 | Wide nasal bridge |
| 67 | HP:0000218 | High palate |
| 67 | HP:0000369 | Low-set ears |
| 67 | HP:0000470 | Short neck |
| 67 | HP:0000954 | Single transverse palmar crease |
| 67 | HP:0004209 | Clinodactyly of the 5th finger |

---


---


---

# Cluster 69

| Cluster | Term | Name |
| --- | --- | --- |
| 69 | HP:0000028 | Cryptorchidism |
| 69 | HP:0000153 | Abnormality of the mouth |
| 69 | HP:0000243 | Trigonocephaly |
| 69 | HP:0010720 | Abnormal hair pattern |
| 69 | HP:0011800 | Midface retrusion |

---


---


---

# Cluster 71

| Cluster | Term | Name |
| --- | --- | --- |
| 71 | HP:0000358 | Posteriorly rotated ears |
| 71 | HP:0000268 | Dolichocephaly |
| 71 | HP:0000664 | Synophrys |
| 71 | HP:0006482 | Abnormality of dental morphology |
| 71 | HP:0010722 | Asymmetry of the ears |

---


---


---

# Cluster 72

| Cluster | Term | Name |
| --- | --- | --- |
| 72 | HP:0000316 | Hypertelorism |
| 72 | HP:0000194 | Open mouth |
| 72 | HP:0000215 | Thick upper lip vermilion |
| 72 | HP:0000232 | Everted lower lip vermilion |
| 72 | HP:0000248 | Brachycephaly |
| 72 | HP:0000664 | Synophrys |

---


---


---

# Cluster 75

| Cluster | Term | Name |
| --- | --- | --- |
| 75 | HP:0000218 | High palate |
| 75 | HP:0000219 | Thin upper lip vermilion |
| 75 | HP:0000286 | Epicanthus |
| 75 | HP:0000369 | Low-set ears |
| 75 | HP:0000463 | Anteverted nares |
| 75 | HP:0000581 | Blepharophimosis |
| 75 | HP:0000954 | Single transverse palmar crease |
| 75 | HP:0004209 | Clinodactyly of the 5th finger |
| 75 | HP:0000470 | Short neck |

---


---


---

# Cluster 77

| Cluster | Term | Name |
| --- | --- | --- |
| 77 | HP:0000219 | Thin upper lip vermilion |
| 77 | HP:0000343 | Long philtrum |
| 77 | HP:0000347 | Micrognathia |
| 77 | HP:0000431 | Wide nasal bridge |
| 77 | HP:0001252 | Muscular hypotonia |
| 77 | HP:0002007 | Frontal bossing |
| 77 | HP:0001182 | Tapered finger |

---


---


---

# Cluster 80

| Cluster | Term | Name |
| --- | --- | --- |
| 80 | HP:0000954 | Single transverse palmar crease |
| 80 | HP:0001257 | Spasticity |
| 80 | HP:0001510 | Growth delay |
| 80 | HP:0010722 | Asymmetry of the ears |

---


---


---

# Cluster 84

| Cluster | Term | Name |
| --- | --- | --- |
| 84 | HP:0000023 | Inguinal hernia |
| 84 | HP:0000054 | Micropenis |
| 84 | HP:0000238 | Hydrocephalus |
| 84 | HP:0000347 | Micrognathia |
| 84 | HP:0000929 | Abnormality of the skull |
| 84 | HP:0001166 | Arachnodactyly |
| 84 | HP:0001363 | Craniosynostosis |
| 84 | HP:0010823 | Ridged cranial sutures |

---


---


---

# Cluster 85

| Cluster | Term | Name |
| --- | --- | --- |
| 85 | HP:0000431 | Wide nasal bridge |
| 85 | HP:0000194 | Open mouth |
| 85 | HP:0000215 | Thick upper lip vermilion |
| 85 | HP:0000232 | Everted lower lip vermilion |
| 85 | HP:0000341 | Narrow forehead |
| 85 | HP:0000684 | Delayed eruption of teeth |
| 85 | HP:0001837 | Broad toe |
| 85 | HP:0001845 | Overlapping toe |
| 85 | HP:0002208 | Coarse hair |
| 85 | HP:0009623 | Proximal placement of thumb |
| 85 | HP:0010800 | Absent cupid’s bow |

---


---


---

# Cluster 86

| Cluster | Term | Name |
| --- | --- | --- |
| 86 | HP:0000545 | Myopia |
| 86 | HP:0000708 | Behavioral abnormality |
| 86 | HP:0001169 | Broad palm |
| 86 | HP:0001773 | Short foot |
| 86 | HP:0002123 | Generalized myoclonic seizures |
| 86 | HP:0009381 | Short finger |
| 86 | HP:0010465 | Precocious puberty in females |
