# Supplementary material for: Phenotype-genotype comorbidity analysis of patients with rare disorders provides insight into their pathological and molecular bases
Source: PLoS Genet. 2020 Oct 1;16(10):e1009054. doi: 10.1371/journal.pgen.1009054 (PMC7553355; doi:10.1371/journal.pgen.1009054)
Supplement: S4 Report — Cluster Details for Reactome coherent clusters. General details for each of the clusters, as well as details of OMIM diseases and Reactome pathways. Although not shown here due to patient-confidentiality, this report can also include tables of patients assigned to each cluster, including details of their phenotypes and genes that overlap with the phenotypes in the clusters, allowing the interested user to generate such information for their own patient cohort. (HTML) [file pgen.1009054.s004.html]

cluster\_details\_reactome\_template.utf8.md


# Report 5: Cluster Details for Reactome coherent clusters

## reactome clusters

---


---


---

# Cluster 1

| Cluster | Term | Name |
| --- | --- | --- |
| 1 | HP:0010485 | Hyperextensibility at elbow |
| 1 | HP:0010500 | Hyperextensibility of the knee |
| 1 | HP:0002751 | Kyphoscoliosis |

| Cluster | Term | Name | Genes | Percentage\_of\_nodes\_with\_funsys |
| --- | --- | --- | --- | --- |
| 1 | R-HSA-888590 | GABA synthesis, release, reuptake and degradation | STX1A, DNAJC5, SLC6A11, ABAT, SLC6A1, CPLX1 | 100 |
| 1 | R-HSA-8956321 | Nucleotide salvage | HPRT1, UCKL1 | 100 |
| 1 | R-HSA-375276 | Peptide ligand-binding receptors | OPRL1, NPBWR2 | 100 |
| 1 | R-HSA-73614 | Pyrimidine salvage | UCKL1 | 100 |
| 1 | R-HSA-373076 | Class A1 (Rhodopsin-like receptors) | OPRL1, NPBWR2 | 100 |
| 1 | R-HSA-418594 | G alpha (i) signalling events | TAS2R16, AKR1C3, OPRL1, GNG13, TAS2R30, OPRD1, AKR1C4, RDH5, GNB1, RBP1, SDC2, LRAT, NPBWR2, PDE1B, TAS2R7, GPC6, TAS2R5, CNR2, TAS2R41, PLA2G4A, TAS2R9, TAS2R19, TAS2R43, GNGT1, TAS2R10, TAS2R14, GRM3, CORT, SDC3, NPY, NPBWR1, GPC5, GUCY2D, MCHR2, KNG1, LRP12, TAS2R40, TAS2R20, PRKCG, PDE1C, PPP3R1, RDH10, OPRK1, ADCY1, MAPK1, GPIHBP1, NPY2R, TAS2R46, TAS2R38, AGT, PRKAR2B, GNG11, GRK7, RBP2, RGS8, TAS2R13, RGS16, TAS1R3, TAS2R39, PRKCQ, TAS2R4, TAS2R3, ACKR3, GPSM1, RGS11, APOA2, ANXA1, CDK5, AGRN, GPR37, GPC1, GNG4, SAG, TAS2R60, GRM8, PENK, GRK1, NAPEPLD, TAS2R8, RXFP4, CALM2, HTR5A, DHRS3, TAS2R31, HEBP1, AKR1C1, TAS1R2, RGS19, RGSL1, DRD3, RGS20, RGS22, OPN1SW, TAS2R50 | 100 |

| Cluster | Term | Name | HPOs\_in\_clusters |
| --- | --- | --- | --- |
| 1 | OMIM:130000 | EHLERS-DANLOS SYNDROME, CLASSIC TYPE, 1; EDSCL1 | HP:0010500, HP:0010485 |

---


---


---

# Cluster 3

| Cluster | Term | Name |
| --- | --- | --- |
| 3 | HP:0001792 | Small nail |
| 3 | HP:0006610 | Wide intermamillary distance |
| 3 | HP:0000430 | Underdeveloped nasal alae |

| Cluster | Term | Name | Genes | Percentage\_of\_nodes\_with\_funsys |
| --- | --- | --- | --- | --- |
| 3 | R-HSA-6805567 | Keratinization | LELP1, KRTAP10-3, KRT80, SPRR2D, KRTAP19-1, KRTAP20-2, KRT81, KRT86, KRTAP22-1, KRTAP10-1, KRT74, KRTAP19-4, KRT85, KRTAP21-2, KRTAP19-6, SPRR2E, PKP4, KRT77, KRTAP8-1, KRT3, SPRR1B, SPRR2G, KRT1, SPRR2A, KRT6A, KRT7, KRTAP10-12, KRT79, KRT18, KRTAP19-5, KRTAP19-7, KRTAP10-7, KRT71, KRTAP11-1, KRT6C, SPRR2F, KRTAP20-1, KRTAP19-3, CSTA, KRTAP10-10, KRTAP10-8, KRTAP6-2, TCHH, SPRR2B, LOR, KRTAP12-3, KRTAP21-1, KAZN, PPL, KRTAP6-1, KRTAP12-2, KRTAP10-2, KRTAP10-6, KRT84, KRT76, KRT75, PKP1, KRT5, KRT73, CASP14, KRT83, KRT82, KRT2, KRTAP12-4, KRTAP21-3, KRT6B, KRTAP10-11, KRTAP12-1, KRT78, KRT8, KRT72, KRTAP10-9, PRSS8, KRTAP19-2, KRTAP19-8 | 100 |
| 3 | R-HSA-6809371 | Formation of the cornified envelope | LELP1, KRT80, SPRR2D, KRT81, KRT86, KLK13, KRT74, KRT85, CELA2A, SPRR2E, KLK8, KRT77, KRT3, SPRR1B, SPRR2G, KRT1, SPRR2A, KRT6A, KLK14, KRT7, KRT79, KRT18, KRT71, KLK12, KRT6C, SPRR2F, CSTA, PPL, TCHH, SPRR2B, LOR, KAZN, KRT84, KLK5, KRT76, KRT75, PKP1, KRT5, KRT73, KRT82, KRT2, KRT6B, KRT78, KRT8, KRT72, CASP14, KRT83 | 100 |

| Cluster | Term | Name | HPOs\_in\_clusters |
| --- | --- | --- | --- |
| 3 | OMIM:129400 | RAPP-HODGKIN SYNDROME; RHS | HP:0000430, HP:0001792 |
| 3 | OMIM:219000 | FRASER SYNDROME 1; FRASRS1 | HP:0000430, HP:0006610 |
| 3 | OMIM:300209 | SIMPSON-GOLABI-BEHMEL SYNDROME, TYPE 2; SGBS2 | HP:0001792, HP:0006610 |
| 3 | OMIM:616580 | AU-KLINE SYNDROME; AUKS | HP:0000430, HP:0006610 |

---


---


---

# Cluster 9

| Cluster | Term | Name |
| --- | --- | --- |
| 9 | HP:0002007 | Frontal bossing |
| 9 | HP:0000280 | Coarse facial features |
| 9 | HP:0002230 | Generalized hirsutism |
| 9 | HP:0002353 | EEG abnormality |

| Cluster | Term | Name | Genes | Percentage\_of\_nodes\_with\_funsys |
| --- | --- | --- | --- | --- |
| 9 | R-HSA-442755 | Activation of NMDA receptors and postsynaptic events | DLG4, GRIN1, CAMK2B, BRAF, CALM2, ACTN2, ADCY1, DLG1, RPS6KA2, PDPK1, LRRC7 | 100 |
| 9 | R-HSA-438064 | Post NMDA receptor activation events | DLG4, GRIN1, CAMK2B, BRAF, CALM2, ACTN2, GRIN2B, CAMK2D, ADCY1, DLG1, RPS6KA2, PDPK1, LRRC7 | 100 |

| Cluster | Term | Name | HPOs\_in\_clusters |
| --- | --- | --- | --- |
| 9 | OMIM:269921 | SIALURIA | HP:0002007, HP:0000280, HP:0002230 |
| 9 | OMIM:617281 | EPILEPTIC ENCEPHALOPATHY, EARLY INFANTILE, 49; EIEE49 | HP:0002007, HP:0000280, HP:0002353 |

---


---


---

# Cluster 12

| Cluster | Term | Name |
| --- | --- | --- |
| 12 | HP:0001629 | Ventricular septal defect |
| 12 | HP:0001643 | Patent ductus arteriosus |
| 12 | HP:0001631 | Atrial septal defect |

| Cluster | Term | Name | Genes | Percentage\_of\_nodes\_with\_funsys |
| --- | --- | --- | --- | --- |
| 12 | R-HSA-6803157 | Antimicrobial peptides | PRTN3, BPIFB6, S100A8, DEFB128, CLU, DEFB105A, PDZD11, PGLYRP4, PLA2G2A, DEFB107B, DEFB131A, DEFA6, DEFA1B, DEFB4B, DEFB106B, DEFB104A, DEFB105B, DEFB107A, S100A7, CCR6, BPIFB1, RNASE3, DEFB103B, DEFB103A, DEFB124, RNASE8, BPIFB4, DEFB127, BPI, HTN3, DEFA5, DEFB125, DEFB115, DEFB104B, DEFB136, DEFB118, DEFB116, HTN1, DEFB4A, DEFB1, DEFB130A, DEFB121, DEFA1, RNASE7, RNASE6, LEAP2, S100A7A, DEFA4, BPIFB2, ITLN1, PGLYRP3, DEFB126, DEFA3, BPIFA1, ATP7A, CHGA, DEFB130B, DEFB123, BPIFA2, DEFB132, PRSS3, DEFB119, ELANE, S100A9, DCD, TLR2, DEFB134, DEFB106A, DEFB135, DEFB129 | 100 |
| 12 | R-HSA-1462054 | Alpha-defensins | DEFA3, DEFA1, DEFA6, DEFA1B, DEFA4, DEFA5 | 100 |
| 12 | R-HSA-1461973 | Defensins | DEFB128, DEFB105A, DEFB107B, DEFB131A, DEFA6, DEFA1B, DEFB4B, DEFB106B, DEFB104A, DEFB105B, DEFB107A, CCR6, DEFB103B, DEFB103A, DEFB124, DEFB127, DEFA5, DEFB125, DEFB115, DEFB104B, DEFB136, DEFB118, DEFB116, DEFB4A, DEFB1, DEFB130A, DEFB121, DEFA1, DEFA4, DEFB126, DEFA3, DEFB130B, DEFB123, DEFB132, PRSS3, DEFB119, TLR2, DEFB134, DEFB106A, DEFB135, DEFB129 | 100 |

| Cluster | Term | Name | HPOs\_in\_clusters |
| --- | --- | --- | --- |
| 12 | OMIM:117550 | SOTOS SYNDROME 1; SOTOS1 | HP:0001629, HP:0001643, HP:0001631 |
| 12 | OMIM:117650 | CEREBROCOSTOMANDIBULAR SYNDROME; CCMS | HP:0001629, HP:0001643, HP:0001631 |
| 12 | OMIM:121050 | CONTRACTURAL ARACHNODACTYLY, CONGENITAL; CCA | HP:0001629, HP:0001643, HP:0001631 |
| 12 | OMIM:135900 | COFFIN-SIRIS SYNDROME 1; CSS1 | HP:0001629, HP:0001643, HP:0001631 |
| 12 | OMIM:145410 | OPITZ GBBB SYNDROME, TYPE II; GBBB2 | HP:0001629, HP:0001643, HP:0001631 |
| 12 | OMIM:163950 | NOONAN SYNDROME 1; NS1 | HP:0001629, HP:0001643, HP:0001631 |
| 12 | OMIM:180849 | RUBINSTEIN-TAYBI SYNDROME 1; RSTS1 | HP:0001629, HP:0001643, HP:0001631 |
| 12 | OMIM:201000 | CARPENTER SYNDROME 1; CRPT1 | HP:0001629, HP:0001643, HP:0001631 |
| 12 | OMIM:214800 | CHARGE SYNDROME | HP:0001629, HP:0001643, HP:0001631 |
| 12 | OMIM:235730 | MOWAT-WILSON SYNDROME; MOWS | HP:0001629, HP:0001643, HP:0001631 |
| 12 | OMIM:257920 | 3MC SYNDROME 1; 3MC1 | HP:0001629, HP:0001643, HP:0001631 |
| 12 | OMIM:261540 | PETERS-PLUS SYNDROME; PTRPLS | HP:0001629, HP:0001643, HP:0001631 |
| 12 | OMIM:268300 | ROBERTS SYNDROME; RBS | HP:0001629, HP:0001643, HP:0001631 |
| 12 | OMIM:270400 | SMITH-LEMLI-OPITZ SYNDROME; SLOS | HP:0001629, HP:0001643, HP:0001631 |
| 12 | OMIM:300166 | MICROPHTHALMIA, SYNDROMIC 2; MCOPS2 | HP:0001629, HP:0001643, HP:0001631 |
| 12 | OMIM:300373 | OSTEOPATHIA STRIATA WITH CRANIAL SCLEROSIS; OSCS | HP:0001629, HP:0001643, HP:0001631 |
| 12 | OMIM:300963 | RITSCHER-SCHINZEL SYNDROME 2; RTSC2 | HP:0001629, HP:0001643, HP:0001631 |
| 12 | OMIM:600001 | HEART DEFECTS, CONGENITAL, AND OTHER CONGENITAL ANOMALIES; HDCA | HP:0001629, HP:0001643, HP:0001631 |
| 12 | OMIM:601186 | MICROPHTHALMIA, SYNDROMIC 9; MCOPS9 | HP:0001629, HP:0001643, HP:0001631 |
| 12 | OMIM:609942 | NOONAN SYNDROME 3; NS3 | HP:0001629, HP:0001643, HP:0001631 |
| 12 | OMIM:613870 | HIRSCHSPRUNG DISEASE, CARDIAC DEFECTS, AND AUTONOMIC DYSFUNCTION; HCAD | HP:0001629, HP:0001643, HP:0001631 |
| 12 | OMIM:616268 | MENTAL RETARDATION, AUTOSOMAL DOMINANT 32; MRD32 | HP:0001629, HP:0001643, HP:0001631 |
| 12 | OMIM:618142 | MICROCEPHALY, FACIAL DYSMORPHISM, RENAL AGENESIS, AND AMBIGUOUS GENITALIA SYNDROME; MFRG | HP:0001629, HP:0001643, HP:0001631 |
| 12 | OMIM:618280 | CARDIAC-UROGENITAL SYNDROME; CUGS | HP:0001629, HP:0001643, HP:0001631 |
| 12 | OMIM:100300 | ADAMS-OLIVER SYNDROME 1; AOS1 | HP:0001629, HP:0001631 |
| 12 | OMIM:105650 | DIAMOND-BLACKFAN ANEMIA 1; DBA1 | HP:0001629, HP:0001631 |
| 12 | OMIM:106260 | ANKYLOBLEPHARON-ECTODERMAL DEFECTS-CLEFT LIP/PALATE; AEC | HP:0001629, HP:0001643 |
| 12 | OMIM:118450 | ALAGILLE SYNDROME 1; ALGS1 | HP:0001629, HP:0001631 |
| 12 | OMIM:145420 | HYPERTELORISM, TEEBI TYPE; TBHS | HP:0001629, HP:0001631 |
| 12 | OMIM:147920 | KABUKI SYNDROME 1; KABUK1 | HP:0001629, HP:0001631 |
| 12 | OMIM:150250 | LARSEN SYNDROME; LRS | HP:0001629, HP:0001631 |
| 12 | OMIM:153400 | LYMPHEDEMA-DISTICHIASIS SYNDROME; LPHDST | HP:0001629, HP:0001643 |
| 12 | OMIM:188400 | DIGEORGE SYNDROME; DGS | HP:0001629, HP:0001643 |
| 12 | OMIM:206900 | MICROPHTHALMIA, SYNDROMIC 3; MCOPS3 | HP:0001629, HP:0001643 |
| 12 | OMIM:208085 | ARTHROGRYPOSIS, RENAL DYSFUNCTION, AND CHOLESTASIS 1; ARCS1 | HP:0001629, HP:0001631 |
| 12 | OMIM:208530 | RIGHT ATRIAL ISOMERISM; RAI | HP:0001629, HP:0001631 |
| 12 | OMIM:208540 | RENAL-HEPATIC-PANCREATIC DYSPLASIA 1; RHPD1 | HP:0001643, HP:0001631 |
| 12 | OMIM:211750 | C SYNDROME | HP:0001629, HP:0001643 |
| 12 | OMIM:214100 | PEROXISOME BIOGENESIS DISORDER 1A (ZELLWEGER); PBD1A | HP:0001629, HP:0001643 |
| 12 | OMIM:218040 | COSTELLO SYNDROME; CSTLO | HP:0001629, HP:0001631 |
| 12 | OMIM:220210 | RITSCHER-SCHINZEL SYNDROME 1; RTSC1 | HP:0001629, HP:0001631 |
| 12 | OMIM:235510 | HENNEKAM LYMPHANGIECTASIA-LYMPHEDEMA SYNDROME 1; HKLLS1 | HP:0001629, HP:0001631 |
| 12 | OMIM:243800 | JOHANSON-BLIZZARD SYNDROME; JBS | HP:0001629, HP:0001631 |
| 12 | OMIM:249270 | THIAMINE-RESPONSIVE MEGALOBLASTIC ANEMIA SYNDROME; TRMA | HP:0001629, HP:0001631 |
| 12 | OMIM:253300 | SPINAL MUSCULAR ATROPHY, TYPE I; SMA1 | HP:0001629, HP:0001631 |
| 12 | OMIM:256520 | NEU-LAXOVA SYNDROME 1; NLS1 | HP:0001629, HP:0001643 |
| 12 | OMIM:258315 | OMODYSPLASIA 1; OMOD1 | HP:0001629, HP:0001631 |
| 12 | OMIM:270100 | HETEROTAXY, VISCERAL, 5, AUTOSOMAL; HTX5 | HP:0001629, HP:0001631 |
| 12 | OMIM:275210 | RESTRICTIVE DERMOPATHY, LETHAL | HP:0001643, HP:0001631 |
| 12 | OMIM:277600 | WEILL-MARCHESANI SYNDROME 1; WMS1 | HP:0001629, HP:0001643 |
| 12 | OMIM:300855 | OGDEN SYNDROME; OGDNS | HP:0001629, HP:0001631 |
| 12 | OMIM:300967 | MENTAL RETARDATION, X-LINKED, SYNDROMIC 34; MRXS34 | HP:0001629, HP:0001643 |
| 12 | OMIM:300968 | MENTAL RETARDATION, X-LINKED 99, SYNDROMIC, FEMALE-RESTRICTED; MRXS99F | HP:0001643, HP:0001631 |
| 12 | OMIM:306955 | HETEROTAXY, VISCERAL, 1, X-LINKED; HTX1 | HP:0001629, HP:0001631 |
| 12 | OMIM:309500 | RENPENNING SYNDROME 1; RENS1 | HP:0001629, HP:0001631 |
| 12 | OMIM:309520 | LUJAN-FRYNS SYNDROME | HP:0001629, HP:0001631 |
| 12 | OMIM:309801 | LINEAR SKIN DEFECTS WITH MULTIPLE CONGENITAL ANOMALIES 1; LSDMCA1 | HP:0001629, HP:0001631 |
| 12 | OMIM:312870 | SIMPSON-GOLABI-BEHMEL SYNDROME, TYPE 1; SGBS1 | HP:0001629, HP:0001643 |
| 12 | OMIM:600987 | CLEFT PALATE, CARDIAC DEFECTS, AND MENTAL RETARDATION; CPCMR | HP:0001629, HP:0001631 |
| 12 | OMIM:602482 | AXENFELD-RIEGER SYNDROME, TYPE 3; RIEG3 | HP:0001643, HP:0001631 |
| 12 | OMIM:602535 | MARSHALL-SMITH SYNDROME; MRSHSS | HP:0001643, HP:0001631 |
| 12 | OMIM:603387 | MEGALENCEPHALY-POLYMICROGYRIA-POLYDACTYLY-HYDROCEPHALUS SYNDROME 1; MPPH1 | HP:0001629, HP:0001631 |
| 12 | OMIM:604169 | LEFT VENTRICULAR NONCOMPACTION 1; LVNC1 | HP:0001629, HP:0001643 |
| 12 | OMIM:605039 | BOHRING-OPITZ SYNDROME; BOPS | HP:0001629, HP:0001631 |
| 12 | OMIM:606003 | TRANSALDOLASE DEFICIENCY | HP:0001629, HP:0001643 |
| 12 | OMIM:606170 | GENITOPATELLAR SYNDROME; GTPTS | HP:0001629, HP:0001631 |
| 12 | OMIM:607323 | DUANE-RADIAL RAY SYNDROME; DRRS | HP:0001629, HP:0001631 |
| 12 | OMIM:607721 | NOONAN SYNDROME-LIKE DISORDER WITH LOOSE ANAGEN HAIR 1; NSLH1 | HP:0001629, HP:0001631 |
| 12 | OMIM:608328 | WEILL-MARCHESANI SYNDROME 2; WMS2 | HP:0001629, HP:0001643 |
| 12 | OMIM:608572 | BURN-MCKEOWN SYNDROME; BMKS | HP:0001629, HP:0001631 |
| 12 | OMIM:610443 | KOOLEN-DE VRIES SYNDROME; KDVS | HP:0001629, HP:0001631 |
| 12 | OMIM:610978 | CHOREOATHETOSIS AND CONGENITAL HYPOTHYROIDISM WITH OR WITHOUT PULMONARY DYSFUNCTION; CAHTP | HP:0001629, HP:0001631 |
| 12 | OMIM:611134 | MECKEL SYNDROME, TYPE 4; MKS4 | HP:0001629, HP:0001631 |
| 12 | OMIM:612289 | FONTAINE PROGEROID SYNDROME; FPS | HP:0001643, HP:0001631 |
| 12 | OMIM:612541 | NEUTROPENIA, SEVERE CONGENITAL, 4, AUTOSOMAL RECESSIVE; SCN4 | HP:0001643, HP:0001631 |
| 12 | OMIM:612561 | DIAMOND-BLACKFAN ANEMIA 6; DBA6 | HP:0001629, HP:0001643 |
| 12 | OMIM:612562 | DIAMOND-BLACKFAN ANEMIA 7; DBA7 | HP:0001629, HP:0001643 |
| 12 | OMIM:612938 | GROWTH RETARDATION, DEVELOPMENTAL DELAY, AND FACIAL DYSMORPHISM; GDFD | HP:0001629, HP:0001643 |
| 12 | OMIM:613001 | ENCEPHALOCRANIOCUTANEOUS LIPOMATOSIS; ECCL | HP:0001629, HP:0001631 |
| 12 | OMIM:614080 | MULTIPLE CONGENITAL ANOMALIES-HYPOTONIA-SEIZURES SYNDROME 1; MCAHS1 | HP:0001643, HP:0001631 |
| 12 | OMIM:614114 | MOSAIC VARIEGATED ANEUPLOIDY SYNDROME 2; MVA2 | HP:0001629, HP:0001631 |
| 12 | OMIM:614262 | ARTHROGRYPOSIS, PERTHES DISEASE, AND UPWARD GAZE PALSY; APUG | HP:0001629, HP:0001631 |
| 12 | OMIM:614886 | PEROXISOME BIOGENESIS DISORDER 12A (ZELLWEGER); PBD12A | HP:0001643, HP:0001631 |
| 12 | OMIM:615355 | NOONAN SYNDROME 8; NS8 | HP:0001629, HP:0001631 |
| 12 | OMIM:616777 | SECKEL SYNDROME 9; SCKL9 | HP:0001629, HP:0001631 |
| 12 | OMIM:616894 | ROBINOW SYNDROME, AUTOSOMAL DOMINANT 3; DRS3 | HP:0001629, HP:0001643 |
| 12 | OMIM:617021 | HYDROPS, LACTIC ACIDOSIS, AND SIDEROBLASTIC ANEMIA; HLASA | HP:0001629, HP:0001643 |
| 12 | OMIM:617044 | SHORT STATURE, DEVELOPMENTAL DELAY, AND CONGENITAL HEART DEFECTS; SDDHD | HP:0001629, HP:0001643 |
| 12 | OMIM:617063 | MEIER-GORLIN SYNDROME 7; MGORS7 | HP:0001629, HP:0001631 |
| 12 | OMIM:617360 | CONGENITAL HEART DEFECTS, DYSMORPHIC FACIAL FEATURES, AND INTELLECTUAL DEVELOPMENTAL DISORDER; CHDFIDD | HP:0001629, HP:0001631 |
| 12 | OMIM:617478 | STRUCTURAL HEART DEFECTS AND RENAL ANOMALIES SYNDROME; SHDRA | HP:0001629, HP:0001631 |
| 12 | OMIM:617506 | NOONAN SYNDROME-LIKE DISORDER WITH LOOSE ANAGEN HAIR 2; NSLH2 | HP:0001629, HP:0001643 |
| 12 | OMIM:617602 | CONGENITAL HEART DEFECTS AND SKELETAL MALFORMATIONS SYNDROME; CHDSKM | HP:0001629, HP:0001631 |
| 12 | OMIM:617912 | CONGENITAL HEART DEFECTS, MULTIPLE TYPES, 5; CHTD5 | HP:0001629, HP:0001631 |
| 12 | OMIM:618162 | SPONDYLOEPIMETAPHYSEAL DYSPLASIA, KRAKOW TYPE; SEMDK | HP:0001643, HP:0001631 |
| 12 | OMIM:618223 | VERTEBRAL ANOMALIES AND VARIABLE ENDOCRINE AND T-CELL DYSFUNCTION; VETD | HP:0001643, HP:0001631 |
| 12 | OMIM:618316 | INTELLECTUAL DEVELOPMENTAL DISORDER WITH CARDIAC DEFECTS AND DYSMORPHIC FACIES; IDDCDF | HP:0001629, HP:0001643 |

---


---


---

# Cluster 13

| Cluster | Term | Name |
| --- | --- | --- |
| 13 | HP:0000219 | Thin upper lip vermilion |
| 13 | HP:0000463 | Anteverted nares |
| 13 | HP:0011304 | Broad thumb |

| Cluster | Term | Name | Genes | Percentage\_of\_nodes\_with\_funsys |
| --- | --- | --- | --- | --- |
| 13 | R-HSA-5669034 | TNFs bind their physiological receptors | FASLG, TNFRSF13B, EDARADD, TNFSF13, TNFRSF1B, TNFRSF25, CD27, TNFSF13B, TNFSF4, TNFRSF8, TNFRSF4, TNFSF18, TNFSF11, TNFRSF1A, TNFRSF14, TNFRSF9, TNFRSF18 | 100 |
| 13 | R-HSA-5668541 | TNFR2 non-canonical NF-kB pathway | PSMD2, EDARADD, TNFSF18, PSMF1, FBXW11, TRAF2, TNFRSF1A, TNFRSF9, TNFRSF13B, UBB, TNFSF13, TNFRSF25, PSMA7, PSMA2, CD27, TNFRSF8, TNFSF12-TNFSF13, UBC, LTBR, PSMD1, PSMA4, TRAF3, FASLG, TNFRSF1B, UBA52, TNFRSF14, PSMC2, TNFRSF18, TNFSF12, TNFSF4, TNFRSF4, TNFSF11, PSMD11, TNFRSF13C | 100 |

| Cluster | Term | Name | HPOs\_in\_clusters |
| --- | --- | --- | --- |
| 13 | OMIM:180700 | ROBINOW SYNDROME, AUTOSOMAL DOMINANT 1; DRS1 | HP:0000219, HP:0000463, HP:0011304 |
| 13 | OMIM:268310 | ROBINOW SYNDROME, AUTOSOMAL RECESSIVE 1; RRS1 | HP:0000219, HP:0000463, HP:0011304 |
| 13 | OMIM:616331 | ROBINOW SYNDROME, AUTOSOMAL DOMINANT 2; DRS2 | HP:0000219, HP:0000463, HP:0011304 |
| 13 | OMIM:103050 | ADENYLOSUCCINASE DEFICIENCY; ADSLD | HP:0000219, HP:0000463 |
| 13 | OMIM:122470 | CORNELIA DE LANGE SYNDROME 1; CDLS1 | HP:0000219, HP:0000463 |
| 13 | OMIM:152950 | MICROCEPHALY WITH OR WITHOUT CHORIORETINOPATHY, LYMPHEDEMA, OR MENTAL RETARDATION; MCLMR | HP:0000219, HP:0000463 |
| 13 | OMIM:211380 | ELSAHY-WATERS SYNDROME; ESWS | HP:0000219, HP:0000463 |
| 13 | OMIM:243310 | BARAITSER-WINTER SYNDROME 1; BRWS1 | HP:0000219, HP:0000463 |
| 13 | OMIM:272200 | MULTIPLE SULFATASE DEFICIENCY; MSD | HP:0000463, HP:0011304 |
| 13 | OMIM:274000 | THROMBOCYTOPENIA-ABSENT RADIUS SYNDROME; TAR | HP:0000463, HP:0011304 |
| 13 | OMIM:300000 | OPITZ GBBB SYNDROME, TYPE I; GBBB1 | HP:0000219, HP:0000463 |
| 13 | OMIM:300209 | SIMPSON-GOLABI-BEHMEL SYNDROME, TYPE 2; SGBS2 | HP:0000219, HP:0000463 |
| 13 | OMIM:300558 | MENTAL RETARDATION, X-LINKED 30; MRX30 | HP:0000219, HP:0000463 |
| 13 | OMIM:300590 | CORNELIA DE LANGE SYNDROME 2; CDLS2 | HP:0000219, HP:0000463 |
| 13 | OMIM:309520 | LUJAN-FRYNS SYNDROME | HP:0000219, HP:0011304 |
| 13 | OMIM:309580 | MENTAL RETARDATION-HYPOTONIC FACIES SYNDROME, X-LINKED, 1; MRXHF1 | HP:0000219, HP:0000463 |
| 13 | OMIM:312870 | SIMPSON-GOLABI-BEHMEL SYNDROME, TYPE 1; SGBS1 | HP:0000463, HP:0011304 |
| 13 | OMIM:607812 | CRANIOLENTICULOSUTURAL DYSPLASIA; CLSD | HP:0000219, HP:0000463 |
| 13 | OMIM:608688 | AICAR TRANSFORMYLASE/IMP CYCLOHYDROLASE DEFICIENCY | HP:0000219, HP:0000463 |
| 13 | OMIM:611209 | CONGENITAL DISORDER OF GLYCOSYLATION, TYPE IIg; CDG2G | HP:0000219, HP:0000463 |
| 13 | OMIM:613735 | BRAIN MALFORMATIONS WITH OR WITHOUT URINARY TRACT DEFECTS; BRMUTD | HP:0000219, HP:0000463 |
| 13 | OMIM:614976 | CARPENTER SYNDROME 2; CRPT2 | HP:0000463, HP:0011304 |
| 13 | OMIM:615761 | MENTAL RETARDATION, AUTOSOMAL DOMINANT 23; MRD23 | HP:0000219, HP:0000463 |
| 13 | OMIM:616503 | LETHAL CONGENITAL CONTRACTURE SYNDROME 9; LCCS9 | HP:0000219, HP:0000463 |
| 13 | OMIM:616801 | HYPOTONIA, INFANTILE, WITH PSYCHOMOTOR RETARDATION AND CHARACTERISTIC FACIES 2; IHPRF2 | HP:0000219, HP:0000463 |
| 13 | OMIM:617062 | OKUR-CHUNG NEURODEVELOPMENTAL SYNDROME; OCNDS | HP:0000219, HP:0000463 |
| 13 | OMIM:617450 | JANSEN-DE VRIES SYNDROME; JDVS | HP:0000219, HP:0000463 |
| 13 | OMIM:617641 | CONGENITAL ANOMALIES OF KIDNEY AND URINARY TRACT SYNDROME WITH OR WITHOUT HEARING LOSS, ABNORMAL EARS, OR DEVELOPMENTAL DELAY; CAKUTHED | HP:0000219, HP:0000463 |
| 13 | OMIM:617763 | SHORT STATURE, HEARING LOSS, RETINITIS PIGMENTOSA, AND DISTINCTIVE FACIES; SHRF | HP:0000219, HP:0011304 |
| 13 | OMIM:617877 | SHORT STATURE, FACIAL DYSMORPHISM, AND SKELETAL ANOMALIES WITH OR WITHOUT CARDIAC ANOMALIES; SSFSC | HP:0000219, HP:0000463 |
| 13 | OMIM:618316 | INTELLECTUAL DEVELOPMENTAL DISORDER WITH CARDIAC DEFECTS AND DYSMORPHIC FACIES; IDDCDF | HP:0000219, HP:0000463 |
| 13 | OMIM:618362 | COFFIN-SIRIS SYNDROME 8; CSS8 | HP:0000219, HP:0000463 |

---


---


---

# Cluster 16

| Cluster | Term | Name |
| --- | --- | --- |
| 16 | HP:0000343 | Long philtrum |
| 16 | HP:0000486 | Strabismus |
| 16 | HP:0003196 | Short nose |
| 16 | HP:0002007 | Frontal bossing |

| Cluster | Term | Name | Genes | Percentage\_of\_nodes\_with\_funsys |
| --- | --- | --- | --- | --- |
| 16 | R-HSA-420499 | pheromone receptors)3 (Metabotropic glutamateClass C | TAS2R43, TAS2R1, TAS2R10, TAS2R14, GRM3, TAS2R60, GRM8, TAS2R16, GPRC6A, TAS2R8, TAS2R13, TAS1R3, TAS2R31, TAS2R30, TAS2R40, TAS2R39, TAS2R20, CASR, TAS1R2, TAS2R4, TAS2R3, GRM7, TAS2R7, TAS2R5, TAS2R46, TAS2R41, TAS2R9, TAS2R38, TAS2R19, TAS2R50 | 100 |

| Cluster | Term | Name | HPOs\_in\_clusters |
| --- | --- | --- | --- |
| 16 | OMIM:219200 | CUTIS LAXA, AUTOSOMAL RECESSIVE, TYPE IIA; ARCL2A | HP:0002007, HP:0000343, HP:0003196, HP:0000486 |
| 16 | OMIM:616638 | SMITH-KINGSMORE SYNDROME; SKS | HP:0002007, HP:0000343, HP:0003196, HP:0000486 |
| 16 | OMIM:103050 | ADENYLOSUCCINASE DEFICIENCY; ADSLD | HP:0000343, HP:0003196, HP:0000486 |
| 16 | OMIM:145420 | HYPERTELORISM, TEEBI TYPE; TBHS | HP:0002007, HP:0000343, HP:0003196 |
| 16 | OMIM:166250 | OSTEOGLOPHONIC DYSPLASIA; OGD | HP:0002007, HP:0000343, HP:0003196 |
| 16 | OMIM:194050 | WILLIAMS-BEUREN SYNDROME; WBS | HP:0000343, HP:0003196, HP:0000486 |
| 16 | OMIM:228520 | FIBROCHONDROGENESIS 1; FBCG1 | HP:0002007, HP:0000343, HP:0003196 |
| 16 | OMIM:252150 | MOLYBDENUM COFACTOR DEFICIENCY, COMPLEMENTATION GROUP A; MOCODA | HP:0002007, HP:0000343, HP:0003196 |
| 16 | OMIM:252160 | MOLYBDENUM COFACTOR DEFICIENCY, COMPLEMENTATION GROUP B; MOCODB | HP:0002007, HP:0000343, HP:0003196 |
| 16 | OMIM:256600 | NEURODEGENERATION WITH BRAIN IRON ACCUMULATION 2A; NBIA2A | HP:0002007, HP:0003196, HP:0000486 |
| 16 | OMIM:258315 | OMODYSPLASIA 1; OMOD1 | HP:0002007, HP:0000343, HP:0003196 |
| 16 | OMIM:258480 | OPSISMODYSPLASIA; OPSMD | HP:0002007, HP:0000343, HP:0003196 |
| 16 | OMIM:261515 | D-BIFUNCTIONAL PROTEIN DEFICIENCY | HP:0002007, HP:0000343, HP:0000486 |
| 16 | OMIM:268400 | ROTHMUND-THOMSON SYNDROME; RTS | HP:0002007, HP:0003196, HP:0000486 |
| 16 | OMIM:305450 | OPITZ-KAVEGGIA SYNDROME; OKS | HP:0002007, HP:0000343, HP:0000486 |
| 16 | OMIM:605309 | MACROCEPHALY/AUTISM SYNDROME | HP:0002007, HP:0000343, HP:0003196 |
| 16 | OMIM:608776 | CONGENITAL DISORDER OF GLYCOSYLATION, TYPE Il; CDG1L | HP:0002007, HP:0000343, HP:0003196 |
| 16 | OMIM:613406 | WITTEVEEN-KOLK SYNDROME; WITKOS | HP:0000343, HP:0003196, HP:0000486 |
| 16 | OMIM:614080 | MULTIPLE CONGENITAL ANOMALIES-HYPOTONIA-SEIZURES SYNDROME 1; MCAHS1 | HP:0002007, HP:0000343, HP:0003196 |
| 16 | OMIM:614105 | METHYLMALONATE SEMIALDEHYDE DEHYDROGENASE DEFICIENCY; MMSDHD | HP:0002007, HP:0000343, HP:0003196 |
| 16 | OMIM:615398 | MULTIPLE CONGENITAL ANOMALIES-HYPOTONIA-SEIZURES SYNDROME 3; MCAHS3 | HP:0000343, HP:0003196, HP:0000486 |
| 16 | OMIM:615539 | EHLERS-DANLOS SYNDROME, MUSCULOCONTRACTURAL TYPE, 2; EDSMC2 | HP:0002007, HP:0000343, HP:0003196 |
| 16 | OMIM:616331 | ROBINOW SYNDROME, AUTOSOMAL DOMINANT 2; DRS2 | HP:0002007, HP:0000343, HP:0003196 |
| 16 | OMIM:617157 | SHORT STATURE, BRACHYDACTYLY, INTELLECTUAL DEVELOPMENTAL DISABILITY, AND SEIZURES; SBIDDS | HP:0002007, HP:0000343, HP:0000486 |
| 16 | OMIM:617752 | MENTAL RETARDATION, AUTOSOMAL DOMINANT 49; MRD49 | HP:0000343, HP:0003196, HP:0000486 |
| 16 | OMIM:617991 | DEVELOPMENTAL DELAY, INTELLECTUAL DISABILITY, OBESITY, AND DYSMORPHISM; DIDOD | HP:0000343, HP:0003196, HP:0000486 |

---


---


---

# Cluster 23

| Cluster | Term | Name |
| --- | --- | --- |
| 23 | HP:0000219 | Thin upper lip vermilion |
| 23 | HP:0000431 | Wide nasal bridge |
| 23 | HP:0011304 | Broad thumb |
| 23 | HP:0004279 | Short palm |

| Cluster | Term | Name | Genes | Percentage\_of\_nodes\_with\_funsys |
| --- | --- | --- | --- | --- |
| 23 | R-HSA-5669034 | TNFs bind their physiological receptors | FASLG, TNFRSF13B, EDARADD, TNFSF13, TNFRSF1B, TNFRSF25, CD27, TNFSF13B, TNFSF4, TNFRSF8, TNFRSF4, TNFSF18, TNFSF11, TNFRSF1A, TNFRSF14, TNFRSF9, TNFRSF18 | 75 |
| 23 | R-HSA-5668541 | TNFR2 non-canonical NF-kB pathway | PSMD2, EDARADD, TNFSF18, PSMF1, FBXW11, TRAF2, TNFRSF1A, TNFRSF9, TNFRSF13B, UBB, TNFSF13, TNFRSF25, PSMA7, PSMA2, CD27, TNFRSF8, TNFSF12-TNFSF13, UBC, LTBR, PSMD1, PSMA4, TRAF3, FASLG, TNFRSF1B, UBA52, TNFRSF14, PSMC2, TNFRSF18, TNFSF12, TNFSF4, TNFRSF4, TNFSF11, PSMD11, TNFRSF13C | 100 |

| Cluster | Term | Name | HPOs\_in\_clusters |
| --- | --- | --- | --- |
| 23 | OMIM:180700 | ROBINOW SYNDROME, AUTOSOMAL DOMINANT 1; DRS1 | HP:0004279, HP:0000431, HP:0000219, HP:0011304 |
| 23 | OMIM:268310 | ROBINOW SYNDROME, AUTOSOMAL RECESSIVE 1; RRS1 | HP:0004279, HP:0000431, HP:0000219, HP:0011304 |
| 23 | OMIM:312870 | SIMPSON-GOLABI-BEHMEL SYNDROME, TYPE 1; SGBS1 | HP:0004279, HP:0000431, HP:0011304 |

---


---


---

# Cluster 25

| Cluster | Term | Name |
| --- | --- | --- |
| 25 | HP:0000160 | Narrow mouth |
| 25 | HP:0000219 | Thin upper lip vermilion |
| 25 | HP:0000677 | Oligodontia |
| 25 | HP:0001369 | Arthritis |
| 25 | HP:0002208 | Coarse hair |

| Cluster | Term | Name | Genes | Percentage\_of\_nodes\_with\_funsys |
| --- | --- | --- | --- | --- |
| 25 | R-HSA-5668541 | TNFR2 non-canonical NF-kB pathway | PSMD2, EDARADD, TNFSF18, PSMF1, FBXW11, TRAF2, TNFRSF1A, TNFRSF9, TNFRSF13B, UBB, TNFSF13, TNFRSF25, PSMA7, PSMA2, CD27, TNFRSF8, TNFSF12-TNFSF13, UBC, LTBR, PSMD1, PSMA4, TRAF3, FASLG, TNFRSF1B, UBA52, TNFRSF14, PSMC2, TNFRSF18, TNFSF12, TNFSF4, TNFRSF4, TNFSF11, PSMD11, TNFRSF13C | 80 |

| Cluster | Term | Name | HPOs\_in\_clusters |
| --- | --- | --- | --- |
| 25 | OMIM:612289 | FONTAINE PROGEROID SYNDROME; FPS | HP:0000160, HP:0000677, HP:0002208, HP:0000219 |

---


---


---

# Cluster 7

| Cluster | Term | Name |
| --- | --- | --- |
| 7 | HP:0001629 | Ventricular septal defect |
| 7 | HP:0001642 | Pulmonic stenosis |
| 7 | HP:0000175 | Cleft palate |
| 7 | HP:0001172 | Abnormal thumb morphology |

| Cluster | Term | Name | Genes | Percentage\_of\_nodes\_with\_funsys |
| --- | --- | --- | --- | --- |
| 7 | R-HSA-420029 | Tight junction interactions | CLDN23, CLDN15, CLDN3, CLDN4, CLDN18, CLDN12, CLDN5, CLDN9, CLDN6, CLDN1, CLDN16, CLDN7, PARD6G, PARD6B, F11R, PRKCI, CLDN11 | 75 |
| 7 | R-HSA-211981 | Xenobiotics | CYP2D6, CYP3A4, CYP3A43, ARNT2, CYP1A2, CYP1A1, CYP3A7-CYP3A51P, CYP3A7, AHR, CYP2W1, CYP3A5 | 75 |

| Cluster | Term | Name | HPOs\_in\_clusters |
| --- | --- | --- | --- |
| 7 | OMIM:100300 | ADAMS-OLIVER SYNDROME 1; AOS1 | HP:0001629, HP:0000175, HP:0001642 |
| 7 | OMIM:214800 | CHARGE SYNDROME | HP:0001629, HP:0000175, HP:0001642 |
| 7 | OMIM:220210 | RITSCHER-SCHINZEL SYNDROME 1; RTSC1 | HP:0001629, HP:0000175, HP:0001642 |
| 7 | OMIM:261540 | PETERS-PLUS SYNDROME; PTRPLS | HP:0001629, HP:0000175, HP:0001642 |
| 7 | OMIM:312870 | SIMPSON-GOLABI-BEHMEL SYNDROME, TYPE 1; SGBS1 | HP:0001629, HP:0000175, HP:0001642 |

---


---


---

# Cluster 8

| Cluster | Term | Name |
| --- | --- | --- |
| 8 | HP:0002007 | Frontal bossing |
| 8 | HP:0000306 | Abnormality of the chin |
| 8 | HP:0000506 | Telecanthus |
| 8 | HP:0001363 | Craniosynostosis |

| Cluster | Term | Name | Genes | Percentage\_of\_nodes\_with\_funsys |
| --- | --- | --- | --- | --- |
| 8 | R-HSA-9022692 | Regulation of MECP2 expression and activity | HDAC3, AGO4, AGO1, AGO3, GPS2, TBL1X, CAMK2B, TNRC6A, HIPK2, CALM2, SIN3A, NCOR1, CAMK2D, HDAC2 | 75 |

| Cluster | Term | Name | HPOs\_in\_clusters |
| --- | --- | --- | --- |
| 8 | OMIM:613610 | CRANIOECTODERMAL DYSPLASIA 2; CED2 | HP:0001363, HP:0002007, HP:0000506 |

---


---


---

# Cluster 15

| Cluster | Term | Name |
| --- | --- | --- |
| 15 | HP:0000286 | Epicanthus |
| 15 | HP:0000486 | Strabismus |
| 15 | HP:0000343 | Long philtrum |
| 15 | HP:0003196 | Short nose |

| Cluster | Term | Name | Genes | Percentage\_of\_nodes\_with\_funsys |
| --- | --- | --- | --- | --- |
| 15 | R-HSA-500792 | GPCR ligand binding | CRHR2, TAS2R16, PTAFR, ADGRE2, LINC02210-CRHR1, TAS2R30, OPRD1, GNRHR, GNB1, GPR35, NPSR1, ADGRE3, CRHR1, TAS2R7, TAS2R5, CNR2, TAS2R41, TAS2R9, TAS2R19, SMO, HTR6, TAS2R43, ADGRE5, GNGT1, TAS2R10, TAS2R14, GRM3, CORT, NLN, NPY, CHRM2, MCHR2, TAS2R40, TAS2R20, PTGER1, ADM2, KEL, FZD1, NPY2R, TAS2R46, SHH, TAS2R38, F2RL3, TAC1, GNG11, RAMP1, GPRC6A, DRD5, UCN3, TAS2R13, RXFP1, TAS1R3, ACKR1, NPS, TAS2R39, WNT2, TAS2R4, KISS1R, TAS2R3, FZD9, ACKR3, PROK2, RLN2, HCRTR1, GPR37, RAMP3, TAS2R60, GRM8, ADCYAP1R1, TAS2R8, RXFP4, HTR5A, TAS2R31, HEBP1, WNT16, TAS1R2, PRLH, GHRHR, DRD3, PLPPR3, VIPR2, OPN1SW, PROKR1, CALCR, TAS2R50 | 75 |
| 15 | R-HSA-420499 | pheromone receptors)3 (Metabotropic glutamateClass C | TAS2R43, TAS2R1, TAS2R10, TAS2R14, GRM3, TAS2R60, GRM8, TAS2R16, GPRC6A, TAS2R8, TAS2R13, TAS1R3, TAS2R31, TAS2R30, TAS2R40, TAS2R39, TAS2R20, CASR, TAS1R2, TAS2R4, TAS2R3, GRM7, TAS2R7, TAS2R5, TAS2R46, TAS2R41, TAS2R9, TAS2R38, TAS2R19, TAS2R50 | 75 |

| Cluster | Term | Name | HPOs\_in\_clusters |
| --- | --- | --- | --- |
| 15 | OMIM:194050 | WILLIAMS-BEUREN SYNDROME; WBS | HP:0000286, HP:0000343, HP:0003196, HP:0000486 |
| 15 | OMIM:613406 | WITTEVEEN-KOLK SYNDROME; WITKOS | HP:0000286, HP:0000343, HP:0003196, HP:0000486 |
| 15 | OMIM:617752 | MENTAL RETARDATION, AUTOSOMAL DOMINANT 49; MRD49 | HP:0000286, HP:0000343, HP:0003196, HP:0000486 |
| 15 | OMIM:617991 | DEVELOPMENTAL DELAY, INTELLECTUAL DISABILITY, OBESITY, AND DYSMORPHISM; DIDOD | HP:0000286, HP:0000343, HP:0003196, HP:0000486 |
| 15 | OMIM:103050 | ADENYLOSUCCINASE DEFICIENCY; ADSLD | HP:0000343, HP:0003196, HP:0000486 |
| 15 | OMIM:115150 | CARDIOFACIOCUTANEOUS SYNDROME 1; CFC1 | HP:0000286, HP:0003196, HP:0000486 |
| 15 | OMIM:154780 | MARSHALL SYNDROME; MRSHS | HP:0000343, HP:0003196, HP:0000286 |
| 15 | OMIM:193700 | ARTHROGRYPOSIS, DISTAL, TYPE 2A; DA2A | HP:0000343, HP:0003196, HP:0000286 |
| 15 | OMIM:211750 | C SYNDROME | HP:0000286, HP:0003196, HP:0000486 |
| 15 | OMIM:219200 | CUTIS LAXA, AUTOSOMAL RECESSIVE, TYPE IIA; ARCL2A | HP:0000343, HP:0003196, HP:0000486 |
| 15 | OMIM:243310 | BARAITSER-WINTER SYNDROME 1; BRWS1 | HP:0000343, HP:0003196, HP:0000286 |
| 15 | OMIM:244450 | KAUFMAN OCULOCEREBROFACIAL SYNDROME; KOS | HP:0000286, HP:0003196, HP:0000486 |
| 15 | OMIM:248700 | MARDEN-WALKER SYNDROME; MWKS | HP:0000286, HP:0000343, HP:0000486 |
| 15 | OMIM:257300 | MOSAIC VARIEGATED ANEUPLOIDY SYNDROME 1; MVA1 | HP:0000343, HP:0003196, HP:0000286 |
| 15 | OMIM:258315 | OMODYSPLASIA 1; OMOD1 | HP:0000343, HP:0003196, HP:0000286 |
| 15 | OMIM:261515 | D-BIFUNCTIONAL PROTEIN DEFICIENCY | HP:0000286, HP:0000343, HP:0000486 |
| 15 | OMIM:270400 | SMITH-LEMLI-OPITZ SYNDROME; SLOS | HP:0000286, HP:0000343, HP:0000486 |
| 15 | OMIM:277590 | WEAVER SYNDROME; WVS | HP:0000286, HP:0000343, HP:0000486 |
| 15 | OMIM:300749 | MENTAL RETARDATION AND MICROCEPHALY WITH PONTINE AND CEREBELLAR HYPOPLASIA; MICPCH | HP:0000343, HP:0003196, HP:0000286 |
| 15 | OMIM:305450 | OPITZ-KAVEGGIA SYNDROME; OKS | HP:0000286, HP:0000343, HP:0000486 |
| 15 | OMIM:605130 | WIEDEMANN-STEINER SYNDROME; WDSTS | HP:0000286, HP:0000343, HP:0000486 |
| 15 | OMIM:606232 | PHELAN-MCDERMID SYNDROME; PHMDS | HP:0000286, HP:0000343, HP:0000486 |
| 15 | OMIM:614080 | MULTIPLE CONGENITAL ANOMALIES-HYPOTONIA-SEIZURES SYNDROME 1; MCAHS1 | HP:0000343, HP:0003196, HP:0000286 |
| 15 | OMIM:614105 | METHYLMALONATE SEMIALDEHYDE DEHYDROGENASE DEFICIENCY; MMSDHD | HP:0000343, HP:0003196, HP:0000286 |
| 15 | OMIM:615398 | MULTIPLE CONGENITAL ANOMALIES-HYPOTONIA-SEIZURES SYNDROME 3; MCAHS3 | HP:0000343, HP:0003196, HP:0000486 |
| 15 | OMIM:616638 | SMITH-KINGSMORE SYNDROME; SKS | HP:0000343, HP:0003196, HP:0000486 |
| 15 | OMIM:616723 | SPONDYLOEPIMETAPHYSEAL DYSPLASIA, FADEN-ALKURAYA TYPE; SEMDFA | HP:0000286, HP:0003196, HP:0000486 |
| 15 | OMIM:616910 | IMMUNODEFICIENCY-CENTROMERIC INSTABILITY-FACIAL ANOMALIES SYNDROME 3; ICF3 | HP:0000286, HP:0003196, HP:0000486 |
| 15 | OMIM:617157 | SHORT STATURE, BRACHYDACTYLY, INTELLECTUAL DEVELOPMENTAL DISABILITY, AND SEIZURES; SBIDDS | HP:0000286, HP:0000343, HP:0000486 |
| 15 | OMIM:618218 | BAKER-GORDON SYNDROME; BAGOS | HP:0000286, HP:0003196, HP:0000486 |

---


---


---

# Cluster 26

| Cluster | Term | Name |
| --- | --- | --- |
| 26 | HP:0000294 | Low anterior hairline |
| 26 | HP:0000369 | Low-set ears |
| 26 | HP:0000290 | Abnormality of the forehead |
| 26 | HP:0000508 | Ptosis |

| Cluster | Term | Name | Genes | Percentage\_of\_nodes\_with\_funsys |
| --- | --- | --- | --- | --- |
| 26 | R-HSA-8957322 | Metabolism of steroids | AKR1C3, IDI1, SREBF2, AKR1D1, PLPP6, CYP46A1, CYP11B2, ABCB11, SMARCD3, ACACA, AKR1C2, AKR1C4, AKR1C1, STARD6, TSPO, FDFT1, OSBPL3, CUBN, LRP2, INSIG1, CYP11B1, SLC10A2, IDI2, CYP11A1, SREBF1, SEC24C | 75 |

| Cluster | Term | Name | HPOs\_in\_clusters |
| --- | --- | --- | --- |
| 26 | OMIM:101400 | SAETHRE-CHOTZEN SYNDROME; SCS | HP:0000294, HP:0000369, HP:0000508 |
| 26 | OMIM:180849 | RUBINSTEIN-TAYBI SYNDROME 1; RSTS1 | HP:0000294, HP:0000369, HP:0000508 |
| 26 | OMIM:615009 | SCHUURS-HOEIJMAKERS SYNDROME; SHMS | HP:0000294, HP:0000369, HP:0000508 |

---


---


---

# Cluster 29

| Cluster | Term | Name |
| --- | --- | --- |
| 29 | HP:0000431 | Wide nasal bridge |
| 29 | HP:0000189 | Narrow palate |
| 29 | HP:0000194 | Open mouth |
| 29 | HP:0001845 | Overlapping toe |

| Cluster | Term | Name | Genes | Percentage\_of\_nodes\_with\_funsys |
| --- | --- | --- | --- | --- |
| 29 | R-HSA-196849 | Metabolism of water-soluble vitamins and cofactors | AMN, SHMT1, NAPRT, PARP10, NMNAT1, MTHFR, SLC52A3, NMRK1, RFK, NADK, PANK4, PARP6, PPCDC | 75 |

| Cluster | Term | Name | HPOs\_in\_clusters |
| --- | --- | --- | --- |
| 29 | OMIM:605039 | BOHRING-OPITZ SYNDROME; BOPS | HP:0000431, HP:0000189, HP:0001845 |

---


---


---

# Cluster 31

| Cluster | Term | Name |
| --- | --- | --- |
| 31 | HP:0000175 | Cleft palate |
| 31 | HP:0000365 | Hearing impairment |
| 31 | HP:0001642 | Pulmonic stenosis |
| 31 | HP:0001172 | Abnormal thumb morphology |

| Cluster | Term | Name | Genes | Percentage\_of\_nodes\_with\_funsys |
| --- | --- | --- | --- | --- |
| 31 | R-HSA-211945 | Phase I - Functionalization of compounds | CYP4V2, CYP2D6, CYP51A1, ADH4, FMO2, CYP4F12, FMO3, ADH1B, CMBL, CYP2W1, CYP3A5, CYP4F2, RXRA, AHRR, ADH7, CYP26B1, ADH5, CYP1A2, POR, CYP3A7-CYP3A51P, ALDH3A1, CYP26A1, EPHX1, NQO2, AADAC, ADH6, NCOA2, CYP4F22, ADH1A, MAOB, CYP3A43, CYP4F11, MAOA, CYP1A1, ADH1C, CYP3A7, CYP26C1, FMO1, CYP4F8, CYP3A4, AOC1, BPHL, ARNT2, CYP11A1, CYP4F3, CBR3, CYB5R3, TBXAS1 | 75 |
| 31 | R-HSA-211981 | Xenobiotics | CYP2D6, CYP3A4, CYP3A43, ARNT2, CYP1A2, CYP1A1, CYP3A7-CYP3A51P, CYP3A7, AHR, CYP2W1, CYP3A5 | 75 |

| Cluster | Term | Name | HPOs\_in\_clusters |
| --- | --- | --- | --- |
| 31 | OMIM:261540 | PETERS-PLUS SYNDROME; PTRPLS | HP:0000175, HP:0000365, HP:0001642 |
| 31 | OMIM:312870 | SIMPSON-GOLABI-BEHMEL SYNDROME, TYPE 1; SGBS1 | HP:0000175, HP:0000365, HP:0001642 |
| 31 | OMIM:612541 | NEUTROPENIA, SEVERE CONGENITAL, 4, AUTOSOMAL RECESSIVE; SCN4 | HP:0000175, HP:0000365, HP:0001642 |

---


---


---

# Cluster 4

| Cluster | Term | Name |
| --- | --- | --- |
| 4 | HP:0000368 | Low-set, posteriorly rotated ears |
| 4 | HP:0002020 | Gastroesophageal reflux |
| 4 | HP:0006008 | Unilateral brachydactyly |
| 4 | HP:0007369 | Atrophy/Degeneration affecting the cerebrum |

| Cluster | Term | Name | Genes | Percentage\_of\_nodes\_with\_funsys |
| --- | --- | --- | --- | --- |
| 4 | R-HSA-5669034 | TNFs bind their physiological receptors | FASLG, TNFRSF13B, EDARADD, TNFSF13, TNFRSF1B, TNFRSF25, CD27, TNFSF13B, TNFSF4, TNFRSF8, TNFRSF4, TNFSF18, TNFSF11, TNFRSF1A, TNFRSF14, TNFRSF9, TNFRSF18 | 75 |
| 4 | R-HSA-5668541 | TNFR2 non-canonical NF-kB pathway | PSMD2, EDARADD, TNFSF18, PSMF1, FBXW11, TRAF2, TNFRSF1A, TNFRSF9, TNFRSF13B, UBB, TNFSF13, TNFRSF25, PSMA7, PSMA2, CD27, TNFRSF8, TNFSF12-TNFSF13, UBC, LTBR, PSMD1, PSMA4, TRAF3, FASLG, TNFRSF1B, UBA52, TNFRSF14, PSMC2, TNFRSF18, TNFSF12, TNFSF4, TNFRSF4, TNFSF11, PSMD11, TNFRSF13C | 100 |

---


---


---

# Cluster 5

| Cluster | Term | Name |
| --- | --- | --- |
| 5 | HP:0000431 | Wide nasal bridge |
| 5 | HP:0000179 | Thick lower lip vermilion |
| 5 | HP:0000455 | Broad nasal tip |
| 5 | HP:0002714 | Downturned corners of mouth |

| Cluster | Term | Name | Genes | Percentage\_of\_nodes\_with\_funsys |
| --- | --- | --- | --- | --- |
| 5 | R-HSA-8951664 | Neddylation | FBXO15, PSMA8, PSMD2, FBXO4, ANKRD9, DCAF17, UBE2F, FBXL5, SPSB1, PSMF1, FBXW8, SPSB4, FBXW11, FBXO22, ASB18, DCAF16, CUL4A, PSMD14, WSB2, FBXO44, FBXO21, UBB, KBTBD6, KLHL42, SKP1, FBXO6, PSMA2, KLHL41, COMMD1, DCUN1D1, LMO7, COPS3, FBXW2, PSMD5, PSMD1, WDR5, KLHL22, CCNF, FBXO2, PSMB1, SKP2, COPS8, ASB1, FBXL14, SOCS6, KBTBD7, WDTC1, COPS7B, FBXL7, COMMD6, COMMD3, FBXW10, UBXN7, COP1, FBXL3, UCHL3, KLHL3, FBXW5, BTBD6, KLHL20, COPS4 | 75 |

---


---


---

# Cluster 11

| Cluster | Term | Name |
| --- | --- | --- |
| 11 | HP:0001252 | Muscular hypotonia |
| 11 | HP:0000951 | Abnormality of the skin |
| 11 | HP:0001643 | Patent ductus arteriosus |
| 11 | HP:0005326 | Hypoplastic philtrum |

| Cluster | Term | Name | Genes | Percentage\_of\_nodes\_with\_funsys |
| --- | --- | --- | --- | --- |
| 11 | R-HSA-5621480 | Dectin-2 family | CLEC4C, CLEC4E, MUC3A, MUC17, CLEC6A, CLEC4D, CLEC4A, MUC20, MUC4, MUC12, PLCG2 | 75 |
| 11 | R-HSA-5083625 | Defective GALNT3 causes familial hyperphosphatemic tumoral calcinosis (HFTC) | MUC3A, MUC17, GALNT3, MUC20, MUC4, MUC12 | 75 |
| 11 | R-HSA-5083632 | Defective C1GALT1C1 causes Tn polyagglutination syndrome (TNPS) | MUC3A, MUC17, MUC20, MUC4, MUC12 | 75 |
| 11 | R-HSA-5083636 | Defective GALNT12 causes colorectal cancer 1 (CRCS1) | MUC3A, MUC17, MUC20, MUC4, MUC12 | 75 |

---


---


---

# Cluster 27

| Cluster | Term | Name |
| --- | --- | --- |
| 27 | HP:0000316 | Hypertelorism |
| 27 | HP:0001770 | Toe syndactyly |
| 27 | HP:0002230 | Generalized hirsutism |
| 27 | HP:0002353 | EEG abnormality |

| Cluster | Term | Name | Genes | Percentage\_of\_nodes\_with\_funsys |
| --- | --- | --- | --- | --- |
| 27 | R-HSA-112314 | Neurotransmitter receptors and postsynaptic signal transmission | NEFL, AP2M1, CHRNA5, GNGT1, GNG11, ADCY8, CHRNA1, CHRNB3, MYO6, ADCY2, GRIK2, CALM2, CACNG4, RRAS, GABRB3, CHRNA2, CHRNA7, PRKACG, GABRG3, DLG4, GABRA5, GNB4, CHRNA3, RASGRF1, PDPK1, AP2A1, GRIP2, ADCY9, RAF1, GNB1, PRKCA, CAMKK1, CHRNB4, CHRND, CAMK2B, CACNG3, BRAF, ACTN2, PRKCG, CACNG8, GNAI1, GNAT3, HTR3D, HTR3C, ADCY1, KCNJ15, CHRFAM7A, GABRA3, DLG1, KCNJ3, RPS6KA2, LRRC7, GLRA4, GRIN2D, CHRNA6, CHRNG, GRIN2A, HTR3E | 75 |

---


---


---

# Cluster 30

| Cluster | Term | Name |
| --- | --- | --- |
| 30 | HP:0001171 | Split hand |
| 30 | HP:0001518 | Small for gestational age |
| 30 | HP:0001839 | Split foot |
| 30 | HP:0012725 | Cutaneous syndactyly |

| Cluster | Term | Name | Genes | Percentage\_of\_nodes\_with\_funsys |
| --- | --- | --- | --- | --- |
| 30 | R-HSA-211981 | Xenobiotics | CYP2D6, CYP3A4, CYP3A43, ARNT2, CYP1A2, CYP1A1, CYP3A7-CYP3A51P, CYP3A7, AHR, CYP2W1, CYP3A5 | 75 |
| 30 | R-HSA-997269 | Inhibition of adenylate cyclase pathway | GNAI1, GNAT3, ADCY1 | 75 |
| 30 | R-HSA-6802952 | Signaling by BRAF and RAF fusions | ZC3HAV1, ATG7, KDM7A, AGK, MPRIP, FAM131B, FGB, SRC, KIAA1549, KSR2, ACTB, RAF1, FGA, CLCN6, MAPK3, ESRP1, ARRB2, BRAF, HRAS, ITGA2B, SND1, MARK3, CSK, TRIM24, MAPK1, JAK2, TLN1, QKI, LMNA, TENT4A, AKAP9, PEBP1, AGTRAP, FGG | 75 |
| 30 | R-HSA-8851805 | MET activates RAS signaling | MET, HRAS, HGF | 75 |
| 30 | R-HSA-373080 | 2 (Secretin family receptors)Class B | GHRH, GNG4, GNGT1, ADGRE5, CRHR2, GNG11, RAMP1, RAMP3, WNT7A, ADCYAP1R1, GNG13, UCN3, ADGRE2, GNB5, LINC02210-CRHR1, FZD2, WNT16, GNB1, WNT2, WNT7B, ADM2, PTCH1, GCG, CALCRL, FZD9, ADGRE3, GHRHR, CRHR1, GNB2, VIPR2, FZD1, SHH, CALCR, SMO, WNT2B | 75 |
| 30 | R-HSA-170670 | Adenylate cyclase inhibitory pathway | GNAI1, GNAT3, ADCY1 | 75 |
| 30 | R-HSA-211897 | Cytochrome P450 - arranged by substrate type | CYP2D6, CYP51A1, CYP2E1, CYP4F12, CYP46A1, CYP2W1, CYP3A5, NR1H4, CYP4F2, RXRA, CYP1A2, POR, CYP3A7-CYP3A51P, NCOA2, CYP4F22, CYP3A43, CYP4F11, CYP1A1, CYP3A7, CYP4F8, CYP3A4, ARNT2, CYP11A1, CYP4F3, AHR, TBXAS1 | 75 |

---


---


---

# Cluster 6

| Cluster | Term | Name |
| --- | --- | --- |
| 6 | HP:0001263 | Global developmental delay |
| 6 | HP:0001999 | Abnormal facial shape |
| 6 | HP:0001290 | Generalized hypotonia |

| Cluster | Term | Name | HPOs\_in\_clusters |
| --- | --- | --- | --- |
| 6 | OMIM:250620 | 3-HYDROXYISOBUTYRYL-CoA HYDROLASE DEFICIENCY; HIBCHD | HP:0001999, HP:0001263, HP:0001290 |
| 6 | OMIM:601776 | EHLERS-DANLOS SYNDROME, MUSCULOCONTRACTURAL TYPE, 1; EDSMC1 | HP:0001999, HP:0001263, HP:0001290 |
| 6 | OMIM:607143 | CONGENITAL DISORDER OF GLYCOSYLATION, TYPE Ig; CDG1G | HP:0001999, HP:0001263, HP:0001290 |
| 6 | OMIM:614105 | METHYLMALONATE SEMIALDEHYDE DEHYDROGENASE DEFICIENCY; MMSDHD | HP:0001999, HP:0001263, HP:0001290 |
| 6 | OMIM:614501 | PSYCHOMOTOR RETARDATION, EPILEPSY, AND CRANIOFACIAL DYSMORPHISM; PMRED | HP:0001999, HP:0001263, HP:0001290 |
| 6 | OMIM:616977 | MENTAL RETARDATION, AUTOSOMAL DOMINANT 43; MRD43 | HP:0001999, HP:0001263, HP:0001290 |
| 6 | OMIM:617752 | MENTAL RETARDATION, AUTOSOMAL DOMINANT 49; MRD49 | HP:0001999, HP:0001263, HP:0001290 |
| 6 | OMIM:617796 | MENTAL RETARDATION, AUTOSOMAL DOMINANT 52; MRD52 | HP:0001999, HP:0001263, HP:0001290 |
| 6 | OMIM:617804 | NEURODEVELOPMENTAL DISORDER WITH SEVERE MOTOR IMPAIRMENT AND ABSENT LANGUAGE; NEDMIAL | HP:0001999, HP:0001263, HP:0001290 |
| 6 | OMIM:617807 | NEURODEVELOPMENTAL DISORDER WITH ATAXIC GAIT, ABSENT SPEECH, AND DECREASED CORTICAL WHITE MATTER; NDAGSCW | HP:0001999, HP:0001263, HP:0001290 |
| 6 | OMIM:617976 | EPILEPTIC ENCEPHALOPATHY, EARLY INFANTILE, 63; EIEE63 | HP:0001999, HP:0001263, HP:0001290 |
| 6 | OMIM:617988 | JABERI-ELAHI SYNDROME; JABELS | HP:0001999, HP:0001263, HP:0001290 |
| 6 | OMIM:618004 | EPILEPTIC ENCEPHALOPATHY, EARLY INFANTILE, 64; EIEE64 | HP:0001999, HP:0001263, HP:0001290 |
| 6 | OMIM:618005 | CONGENITAL DISORDER OF GLYCOSYLATION WITH DEFECTIVE FUCOSYLATION 1; CDGF1 | HP:0001999, HP:0001263, HP:0001290 |
| 6 | OMIM:618065 | PONTOCEREBELLAR HYPOPLASIA, TYPE 1D; PCH1D | HP:0001999, HP:0001263, HP:0001290 |
| 6 | OMIM:618106 | MENTAL RETARDATION, AUTOSOMAL DOMINANT 58; MRD58 | HP:0001999, HP:0001263, HP:0001290 |
| 6 | OMIM:618161 | JOUBERT SYNDROME 35; JBTS35 | HP:0001999, HP:0001263, HP:0001290 |
| 6 | OMIM:618164 | CARDIAC, FACIAL, AND DIGITAL ANOMALIES WITH DEVELOPMENTAL DELAY; CAFDADD | HP:0001999, HP:0001263, HP:0001290 |
| 6 | OMIM:100300 | ADAMS-OLIVER SYNDROME 1; AOS1 | HP:0001290, HP:0001263 |
| 6 | OMIM:103050 | ADENYLOSUCCINASE DEFICIENCY; ADSLD | HP:0001290, HP:0001263 |
| 6 | OMIM:105830 | ANGELMAN SYNDROME; AS | HP:0001290, HP:0001263 |
| 6 | OMIM:124000 | MITOCHONDRIAL COMPLEX III DEFICIENCY, NUCLEAR TYPE 1; MC3DN1 | HP:0001290, HP:0001263 |
| 6 | OMIM:130070 | EHLERS-DANLOS SYNDROME, SPONDYLODYSPLASTIC TYPE, 1; EDSSPD1 | HP:0001290, HP:0001263 |
| 6 | OMIM:136570 | CHROMOSOME 16p12.1 DELETION SYNDROME, 520-KB | HP:0001999, HP:0001263 |
| 6 | OMIM:145410 | OPITZ GBBB SYNDROME, TYPE II; GBBB2 | HP:0001290, HP:0001263 |
| 6 | OMIM:147920 | KABUKI SYNDROME 1; KABUK1 | HP:0001290, HP:0001263 |
| 6 | OMIM:157170 | HOLOPROSENCEPHALY 2; HPE2 | HP:0001290, HP:0001263 |
| 6 | OMIM:162300 | MULTIPLE ENDOCRINE NEOPLASIA, TYPE IIB; MEN2B | HP:0001290, HP:0001263 |
| 6 | OMIM:170100 | PROLIDASE DEFICIENCY | HP:0001999, HP:0001263 |
| 6 | OMIM:176270 | PRADER-WILLI SYNDROME; PWS | HP:0001290, HP:0001263 |
| 6 | OMIM:182212 | SHPRINTZEN-GOLDBERG CRANIOSYNOSTOSIS SYNDROME; SGS | HP:0001290, HP:0001263 |
| 6 | OMIM:182290 | SMITH-MAGENIS SYNDROME; SMS | HP:0001290, HP:0001263 |
| 6 | OMIM:194190 | WOLF-HIRSCHHORN SYNDROME; WHS | HP:0001290, HP:0001263 |
| 6 | OMIM:201450 | ACYL-CoA DEHYDROGENASE, MEDIUM-CHAIN, DEFICIENCY OF; ACADMD | HP:0001290, HP:0001263 |
| 6 | OMIM:201470 | ACYL-CoA DEHYDROGENASE, SHORT-CHAIN, DEFICIENCY OF; ACADSD | HP:0001290, HP:0001263 |
| 6 | OMIM:203700 | MITOCHONDRIAL DNA DEPLETION SYNDROME 4A (ALPERS TYPE); MTDPS4A | HP:0001290, HP:0001263 |
| 6 | OMIM:204750 | 2-AMINOADIPIC 2-OXOADIPIC ACIDURIA; AMOXAD | HP:0001290, HP:0001263 |
| 6 | OMIM:206900 | MICROPHTHALMIA, SYNDROMIC 3; MCOPS3 | HP:0001290, HP:0001263 |
| 6 | OMIM:208085 | ARTHROGRYPOSIS, RENAL DYSFUNCTION, AND CHOLESTASIS 1; ARCS1 | HP:0001290, HP:0001263 |
| 6 | OMIM:210200 | 3-METHYLCROTONYL-CoA CARBOXYLASE 1 DEFICIENCY; MCC1D | HP:0001290, HP:0001263 |
| 6 | OMIM:210210 | 3-METHYLCROTONYL-CoA CARBOXYLASE 2 DEFICIENCY; MCC2D | HP:0001290, HP:0001263 |
| 6 | OMIM:211750 | C SYNDROME | HP:0001290, HP:0001263 |
| 6 | OMIM:212065 | CONGENITAL DISORDER OF GLYCOSYLATION, TYPE Ia; CDG1A | HP:0001290, HP:0001263 |
| 6 | OMIM:213200 | SPINOCEREBELLAR ATAXIA, AUTOSOMAL RECESSIVE 2; SCAR2 | HP:0001290, HP:0001263 |
| 6 | OMIM:213300 | JOUBERT SYNDROME 1; JBTS1 | HP:0001290, HP:0001263 |
| 6 | OMIM:214450 | GRISCELLI SYNDROME, TYPE 1; GS1 | HP:0001290, HP:0001263 |
| 6 | OMIM:216360 | COACH SYNDROME | HP:0001290, HP:0001263 |
| 6 | OMIM:218000 | AGENESIS OF THE CORPUS CALLOSUM WITH PERIPHERAL NEUROPATHY; ACCPN | HP:0001290, HP:0001263 |
| 6 | OMIM:218340 | TEMTAMY SYNDROME; TEMTYS | HP:0001290, HP:0001263 |
| 6 | OMIM:219150 | CUTIS LAXA, AUTOSOMAL RECESSIVE, TYPE IIIA; ARCL3A | HP:0001290, HP:0001263 |
| 6 | OMIM:220110 | MITOCHONDRIAL COMPLEX IV DEFICIENCY | HP:0001290, HP:0001263 |
| 6 | OMIM:220111 | LEIGH SYNDROME, FRENCH CANADIAN TYPE; LSFC | HP:0001290, HP:0001263 |
| 6 | OMIM:220210 | RITSCHER-SCHINZEL SYNDROME 1; RTSC1 | HP:0001290, HP:0001263 |
| 6 | OMIM:224050 | CEREBELLAR ATAXIA, MENTAL RETARDATION, AND DYSEQUILIBRIUM SYNDROME 1; CAMRQ1 | HP:0001290, HP:0001263 |
| 6 | OMIM:229050 | FOLATE MALABSORPTION, HEREDITARY | HP:0001290, HP:0001263 |
| 6 | OMIM:230350 | GALACTOSE EPIMERASE DEFICIENCY | HP:0001290, HP:0001263 |
| 6 | OMIM:236270 | HOMOCYSTINURIA-MEGALOBLASTIC ANEMIA, cblE COMPLEMENTATION TYPE; HMAE | HP:0001290, HP:0001263 |
| 6 | OMIM:238970 | HYPERORNITHINEMIA-HYPERAMMONEMIA-HOMOCITRULLINURIA SYNDROME | HP:0001290, HP:0001263 |
| 6 | OMIM:242840 | VICI SYNDROME; VICIS | HP:0001290, HP:0001263 |
| 6 | OMIM:243310 | BARAITSER-WINTER SYNDROME 1; BRWS1 | HP:0001290, HP:0001263 |
| 6 | OMIM:246900 | DIHYDROLIPOAMIDE DEHYDROGENASE DEFICIENCY; DLDD | HP:0001290, HP:0001263 |
| 6 | OMIM:248360 | MALONYL-CoA DECARBOXYLASE DEFICIENCY | HP:0001290, HP:0001263 |
| 6 | OMIM:248500 | MANNOSIDOSIS, ALPHA B, LYSOSOMAL; MANSA | HP:0001290, HP:0001263 |
| 6 | OMIM:248800 | MARINESCO-SJOGREN SYNDROME; MSS | HP:0001290, HP:0001263 |
| 6 | OMIM:249900 | METACHROMATIC LEUKODYSTROPHY DUE TO SAPOSIN B DEFICIENCY | HP:0001290, HP:0001263 |
| 6 | OMIM:250940 | HOMOCYSTINURIA-MEGALOBLASTIC ANEMIA, cblG COMPLEMENTATION TYPE; HMAG | HP:0001290, HP:0001263 |
| 6 | OMIM:251000 | METHYLMALONIC ACIDURIA DUE TO METHYLMALONYL-CoA MUTASE DEFICIENCY | HP:0001290, HP:0001263 |
| 6 | OMIM:251100 | METHYLMALONIC ACIDURIA, cblA TYPE | HP:0001290, HP:0001263 |
| 6 | OMIM:251110 | METHYLMALONIC ACIDURIA, cblB TYPE | HP:0001290, HP:0001263 |
| 6 | OMIM:252010 | MITOCHONDRIAL COMPLEX I DEFICIENCY, NUCLEAR TYPE 1; MC1DN1 | HP:0001290, HP:0001263 |
| 6 | OMIM:252650 | MUCOLIPIDOSIS IV; ML4 | HP:0001290, HP:0001263 |
| 6 | OMIM:253260 | BIOTINIDASE DEFICIENCY | HP:0001290, HP:0001263 |
| 6 | OMIM:253270 | HOLOCARBOXYLASE SYNTHETASE DEFICIENCY | HP:0001290, HP:0001263 |
| 6 | OMIM:256000 | LEIGH SYNDROME; LS | HP:0001290, HP:0001263 |
| 6 | OMIM:256600 | NEURODEGENERATION WITH BRAIN IRON ACCUMULATION 2A; NBIA2A | HP:0001290, HP:0001263 |
| 6 | OMIM:256730 | CEROID LIPOFUSCINOSIS, NEURONAL, 1; CLN1 | HP:0001290, HP:0001263 |
| 6 | OMIM:256810 | MITOCHONDRIAL DNA DEPLETION SYNDROME 6 (HEPATOCEREBRAL TYPE); MTDPS6 | HP:0001290, HP:0001263 |
| 6 | OMIM:257200 | NIEMANN-PICK DISEASE, TYPE A | HP:0001290, HP:0001263 |
| 6 | OMIM:257220 | NIEMANN-PICK DISEASE, TYPE C1; NPC1 | HP:0001290, HP:0001263 |
| 6 | OMIM:260565 | PEHO SYNDROME; PEHO | HP:0001290, HP:0001263 |
| 6 | OMIM:261515 | D-BIFUNCTIONAL PROTEIN DEFICIENCY | HP:0001999, HP:0001263 |
| 6 | OMIM:264090 | WIEDEMANN-RAUTENSTRAUCH SYNDROME; WDRTS | HP:0001290, HP:0001263 |
| 6 | OMIM:266100 | EPILEPSY, PYRIDOXINE-DEPENDENT; EPD | HP:0001290, HP:0001263 |
| 6 | OMIM:266150 | PYRUVATE CARBOXYLASE DEFICIENCY | HP:0001290, HP:0001263 |
| 6 | OMIM:266510 | PEROXISOME BIOGENESIS DISORDER 3B; PBD3B | HP:0001290, HP:0001263 |
| 6 | OMIM:267000 | PERLMAN SYNDROME; PRLMNS | HP:0001999, HP:0001263 |
| 6 | OMIM:269920 | INFANTILE SIALIC ACID STORAGE DISEASE; ISSD | HP:0001290, HP:0001263 |
| 6 | OMIM:271665 | SPONDYLOMETAEPIPHYSEAL DYSPLASIA, SHORT LIMB-HAND TYPE | HP:0001290, HP:0001263 |
| 6 | OMIM:271980 | SUCCINIC SEMIALDEHYDE DEHYDROGENASE DEFICIENCY; SSADHD | HP:0001290, HP:0001263 |
| 6 | OMIM:272300 | SULFITE OXIDASE DEFICIENCY, ISOLATED; ISOD | HP:0001290, HP:0001263 |
| 6 | OMIM:272750 | GM2-GANGLIOSIDOSIS, AB VARIANT | HP:0001290, HP:0001263 |
| 6 | OMIM:277170 | OROFACIODIGITAL SYNDROME VI; OFD6 | HP:0001290, HP:0001263 |
| 6 | OMIM:277380 | METHYLMALONIC ACIDURIA AND HOMOCYSTINURIA, cblF TYPE; MAHCF | HP:0001290, HP:0001263 |
| 6 | OMIM:277400 | METHYLMALONIC ACIDURIA AND HOMOCYSTINURIA, cblC TYPE; MAHCC | HP:0001290, HP:0001263 |
| 6 | OMIM:277410 | METHYLMALONIC ACIDURIA AND HOMOCYSTINURIA, cblD TYPE; MAHCD | HP:0001290, HP:0001263 |
| 6 | OMIM:277590 | WEAVER SYNDROME; WVS | HP:0001290, HP:0001263 |
| 6 | OMIM:300114 | RAYNAUD-CLAES SYNDROME; MRXSRC | HP:0001290, HP:0001263 |
| 6 | OMIM:300243 | MENTAL RETARDATION, X-LINKED, SYNDROMIC, CHRISTIANSON TYPE; MRXSCH | HP:0001290, HP:0001263 |
| 6 | OMIM:300352 | CEREBRAL CREATINE DEFICIENCY SYNDROME 1; CCDS1 | HP:0001290, HP:0001263 |
| 6 | OMIM:300438 | HSD10 MITOCHONDRIAL DISEASE; HSD10MD | HP:0001290, HP:0001263 |
| 6 | OMIM:300475 | DEAFNESS, DYSTONIA, AND CEREBRAL HYPOMYELINATION; DDCH | HP:0001999, HP:0001263 |
| 6 | OMIM:300486 | MENTAL RETARDATION, X-LINKED, WITH CEREBELLAR HYPOPLASIA AND DISTINCTIVE FACIAL APPEARANCE | HP:0001290, HP:0001263 |
| 6 | OMIM:300577 | MENTAL RETARDATION, X-LINKED 91; MRX91 | HP:0001999, HP:0001263 |
| 6 | OMIM:300672 | EPILEPTIC ENCEPHALOPATHY, EARLY INFANTILE, 2; EIEE2 | HP:0001290, HP:0001263 |
| 6 | OMIM:300749 | MENTAL RETARDATION AND MICROCEPHALY WITH PONTINE AND CEREBELLAR HYPOPLASIA; MICPCH | HP:0001290, HP:0001263 |
| 6 | OMIM:300816 | COMBINED OXIDATIVE PHOSPHORYLATION DEFICIENCY 6; COXPD6 | HP:0001290, HP:0001263 |
| 6 | OMIM:300831 | CK SYNDROME | HP:0001290, HP:0001263 |
| 6 | OMIM:300849 | MENTAL RETARDATION, X-LINKED 41; MRX41 | HP:0001290, HP:0001263 |
| 6 | OMIM:300850 | MENTAL RETARDATION, X-LINKED 90; MRX90 | HP:0001290, HP:0001263 |
| 6 | OMIM:300884 | EPILEPTIC ENCEPHALOPATHY, EARLY INFANTILE, 36; EIEE36 | HP:0001290, HP:0001263 |
| 6 | OMIM:300887 | LINEAR SKIN DEFECTS WITH MULTIPLE CONGENITAL ANOMALIES 2; LSDMCA2 | HP:0001999, HP:0001263 |
| 6 | OMIM:300896 | CONGENITAL DISORDER OF GLYCOSYLATION, TYPE IIm; CDG2M | HP:0001290, HP:0001263 |
| 6 | OMIM:300919 | MENTAL RETARDATION, X-LINKED 99; MRX99 | HP:0001290, HP:0001263 |
| 6 | OMIM:300934 | CONGENITAL DISORDER OF GLYCOSYLATION, TYPE Iy; CDG1Y | HP:0001290, HP:0001263 |
| 6 | OMIM:300960 | MEND SYNDROME; MEND | HP:0001290, HP:0001263 |
| 6 | OMIM:300963 | RITSCHER-SCHINZEL SYNDROME 2; RTSC2 | HP:0001290, HP:0001263 |
| 6 | OMIM:300978 | TONNE-KALSCHEUER SYNDROME; TOKAS | HP:0001290, HP:0001263 |
| 6 | OMIM:300986 | MENTAL RETARDATION, X-LINKED, SYNDROMIC, BAIN TYPE; MRXSB | HP:0001290, HP:0001263 |
| 6 | OMIM:300998 | MENTAL RETARDATION, X-LINKED, SYNDROMIC, 35; MRXS35 | HP:0001290, HP:0001263 |
| 6 | OMIM:301006 | GALLOWAY-MOWAT SYNDROME 2, X-LINKED; GAMOS2 | HP:0001290, HP:0001263 |
| 6 | OMIM:301022 | NEURODEVELOPMENTAL DISORDER, X-LINKED, WITH CRANIOFACIAL ABNORMALITIES; NEDXCF | HP:0001290, HP:0001263 |
| 6 | OMIM:301024 | INTELLECTUAL DEVELOPMENTAL DISORDER, X-LINKED 108; MRX108 | HP:0001290, HP:0001263 |
| 6 | OMIM:304110 | CRANIOFRONTONASAL SYNDROME; CFNS | HP:0001290, HP:0001263 |
| 6 | OMIM:304340 | PETTIGREW SYNDROME; PGS | HP:0001290, HP:0001263 |
| 6 | OMIM:311900 | TARP SYNDROME; TARPS | HP:0001290, HP:0001263 |
| 6 | OMIM:312080 | PELIZAEUS-MERZBACHER DISEASE; PMD | HP:0001290, HP:0001263 |
| 6 | OMIM:312170 | PYRUVATE DEHYDROGENASE E1-ALPHA DEFICIENCY; PDHAD | HP:0001290, HP:0001263 |
| 6 | OMIM:314580 | WIEACKER-WOLFF SYNDROME; WRWF | HP:0001290, HP:0001263 |
| 6 | OMIM:600721 | D-2-HYDROXYGLUTARIC ACIDURIA 1; D2HGA1 | HP:0001290, HP:0001263 |
| 6 | OMIM:601238 | CEREBELLAR ATAXIA, CAYMAN TYPE; ATCAY | HP:0001290, HP:0001263 |
| 6 | OMIM:601539 | PEROXISOME BIOGENESIS DISORDER 1B; PBD1B | HP:0001290, HP:0001263 |
| 6 | OMIM:602473 | ENCEPHALOPATHY, ETHYLMALONIC; EE | HP:0001290, HP:0001263 |
| 6 | OMIM:602501 | MEGALENCEPHALY-CAPILLARY MALFORMATION-POLYMICROGYRIA SYNDROME; MCAP | HP:0001290, HP:0001263 |
| 6 | OMIM:603553 | HEMOPHAGOCYTIC LYMPHOHISTIOCYTOSIS, FAMILIAL, 2; FHL2 | HP:0001290, HP:0001263 |
| 6 | OMIM:603585 | CONGENITAL DISORDER OF GLYCOSYLATION, TYPE IIf; CDG2F | HP:0001290, HP:0001263 |
| 6 | OMIM:604168 | CONGENITAL CATARACTS, FACIAL DYSMORPHISM, AND NEUROPATHY; CCFDN | HP:0001999, HP:0001263 |
| 6 | OMIM:604273 | MITOCHONDRIAL COMPLEX V (ATP SYNTHASE) DEFICIENCY, NUCLEAR TYPE 1; MC5DN1 | HP:0001290, HP:0001263 |
| 6 | OMIM:604369 | SALLA DISEASE; SD | HP:0001290, HP:0001263 |
| 6 | OMIM:604377 | CARDIOENCEPHALOMYOPATHY, FATAL INFANTILE, DUE TO CYTOCHROME c OXIDASE DEFICIENCY 1; CEMCOX1 | HP:0001290, HP:0001263 |
| 6 | OMIM:605039 | BOHRING-OPITZ SYNDROME; BOPS | HP:0001290, HP:0001263 |
| 6 | OMIM:605130 | WIEDEMANN-STEINER SYNDROME; WDSTS | HP:0001290, HP:0001263 |
| 6 | OMIM:606056 | CONGENITAL DISORDER OF GLYCOSYLATION, TYPE IIb; CDG2B | HP:0001290, HP:0001263 |
| 6 | OMIM:606170 | GENITOPATELLAR SYNDROME; GTPTS | HP:0001290, HP:0001263 |
| 6 | OMIM:606812 | FUMARASE DEFICIENCY; FMRD | HP:0001290, HP:0001263 |
| 6 | OMIM:607091 | CONGENITAL DISORDER OF GLYCOSYLATION, TYPE IId; CDG2D | HP:0001290, HP:0001263 |
| 6 | OMIM:607596 | PONTOCEREBELLAR HYPOPLASIA, TYPE 1A; PCH1A | HP:0001290, HP:0001263 |
| 6 | OMIM:607625 | NIEMANN-PICK DISEASE, TYPE C2; NPC2 | HP:0001290, HP:0001263 |
| 6 | OMIM:608022 | DIAPHANOSPONDYLODYSOSTOSIS | HP:0001290, HP:0001263 |
| 6 | OMIM:608091 | JOUBERT SYNDROME 2; JBTS2 | HP:0001290, HP:0001263 |
| 6 | OMIM:608093 | CONGENITAL DISORDER OF GLYCOSYLATION, TYPE Ij; CDG1J | HP:0001290, HP:0001263 |
| 6 | OMIM:608097 | PERIVENTRICULAR HETEROTOPIA WITH MICROCEPHALY, AUTOSOMAL RECESSIVE; ARPHM | HP:0001290, HP:0001263 |
| 6 | OMIM:608540 | CONGENITAL DISORDER OF GLYCOSYLATION, TYPE Ik; CDG1K | HP:0001290, HP:0001263 |
| 6 | OMIM:608776 | CONGENITAL DISORDER OF GLYCOSYLATION, TYPE Il; CDG1L | HP:0001290, HP:0001263 |
| 6 | OMIM:608779 | CONGENITAL DISORDER OF GLYCOSYLATION, TYPE IIe; CDG2E | HP:0001999, HP:0001290 |
| 6 | OMIM:608782 | PYRUVATE DEHYDROGENASE PHOSPHATASE DEFICIENCY; PDHPD | HP:0001290, HP:0001263 |
| 6 | OMIM:608840 | MUSCULAR DYSTROPHY-DYSTROGLYCANOPATHY (CONGENITAL WITH MENTAL RETARDATION), TYPE B, 6; MDDGB6 | HP:0001290, HP:0001263 |
| 6 | OMIM:609015 | MITOCHONDRIAL TRIFUNCTIONAL PROTEIN DEFICIENCY; MTPD | HP:0001290, HP:0001263 |
| 6 | OMIM:609056 | SALT AND PEPPER DEVELOPMENTAL REGRESSION SYNDROME; SPDRS | HP:0001290, HP:0001263 |
| 6 | OMIM:609180 | CONGENITAL DISORDER OF GLYCOSYLATION, TYPE If; CDG1F | HP:0001290, HP:0001263 |
| 6 | OMIM:609241 | SCHINDLER DISEASE, TYPE I | HP:0001290, HP:0001263 |
| 6 | OMIM:609313 | MENTAL RETARDATION, ENTEROPATHY, DEAFNESS, PERIPHERAL NEUROPATHY, ICHTHYOSIS, AND KERATODERMA; MEDNIK | HP:0001290, HP:0001263 |
| 6 | OMIM:609460 | GOLDBERG-SHPRINTZEN SYNDROME; GOSHS | HP:0001290, HP:0001263 |
| 6 | OMIM:609637 | HOLOPROSENCEPHALY 5; HPE5 | HP:0001999, HP:0001263 |
| 6 | OMIM:609924 | AMINOACYLASE 1 DEFICIENCY; ACY1D | HP:0001290, HP:0001263 |
| 6 | OMIM:610006 | 2-METHYLBUTYRYL-CoA DEHYDROGENASE DEFICIENCY | HP:0001290, HP:0001263 |
| 6 | OMIM:610042 | PITT-HOPKINS-LIKE SYNDROME 1; PTHSL1 | HP:0001290, HP:0001263 |
| 6 | OMIM:610125 | MICROPHTHALMIA, SYNDROMIC 5; MCOPS5 | HP:0001290, HP:0001263 |
| 6 | OMIM:610377 | MEVALONIC ACIDURIA; MEVA | HP:0001290, HP:0001263 |
| 6 | OMIM:610443 | KOOLEN-DE VRIES SYNDROME; KDVS | HP:0001290, HP:0001263 |
| 6 | OMIM:610505 | COMBINED OXIDATIVE PHOSPHORYLATION DEFICIENCY 3; COXPD3 | HP:0001290, HP:0001263 |
| 6 | OMIM:610688 | JOUBERT SYNDROME 6; JBTS6 | HP:0001290, HP:0001263 |
| 6 | OMIM:610883 | POTOCKI-LUPSKI SYNDROME; PTLS | HP:0001290, HP:0001263 |
| 6 | OMIM:610978 | CHOREOATHETOSIS AND CONGENITAL HYPOTHYROIDISM WITH OR WITHOUT PULMONARY DYSFUNCTION; CAHTP | HP:0001290, HP:0001263 |
| 6 | OMIM:611087 | POLYHYDRAMNIOS, MEGALENCEPHALY, AND SYMPTOMATIC EPILEPSY; PMSE | HP:0001290, HP:0001263 |
| 6 | OMIM:611209 | CONGENITAL DISORDER OF GLYCOSYLATION, TYPE IIg; CDG2G | HP:0001999, HP:0001263 |
| 6 | OMIM:611523 | PONTOCEREBELLAR HYPOPLASIA, TYPE 6; PCH6 | HP:0001290, HP:0001263 |
| 6 | OMIM:611553 | NOONAN SYNDROME 5; NS5 | HP:0001999, HP:0001263 |
| 6 | OMIM:611560 | JOUBERT SYNDROME 7; JBTS7 | HP:0001290, HP:0001263 |
| 6 | OMIM:611816 | TEMPLE-BARAITSER SYNDROME; TMBTS | HP:0001290, HP:0001263 |
| 6 | OMIM:612015 | CONGENITAL DISORDER OF GLYCOSYLATION, TYPE In; CDG1N | HP:0001290, HP:0001263 |
| 6 | OMIM:612073 | MITOCHONDRIAL DNA DEPLETION SYNDROME 5 (ENCEPHALOMYOPATHIC WITH OR WITHOUT METHYLMALONIC ACIDURIA); MTDPS5 | HP:0001290, HP:0001263 |
| 6 | OMIM:612233 | LEUKODYSTROPHY, HYPOMYELINATING, 4; HLD4 | HP:0001290, HP:0001263 |
| 6 | OMIM:612291 | JOUBERT SYNDROME 8; JBTS8 | HP:0001290, HP:0001263 |
| 6 | OMIM:612301 | OSTEOPETROSIS, AUTOSOMAL RECESSIVE 7; OPTB7 | HP:0001290, HP:0001263 |
| 6 | OMIM:612337 | MENTAL RETARDATION, AUTOSOMAL DOMINANT 22; MRD22 | HP:0001290, HP:0001263 |
| 6 | OMIM:612621 | MENTAL RETARDATION, AUTOSOMAL DOMINANT 5; MRD5 | HP:0001290, HP:0001263 |
| 6 | OMIM:612780 | SEIZURES, SENSORINEURAL DEAFNESS, ATAXIA, MENTAL RETARDATION, AND ELECTROLYTE IMBALANCE; SESAMES | HP:0001290, HP:0001263 |
| 6 | OMIM:613385 | AUTOIMMUNE DISEASE, MULTISYSTEM, WITH FACIAL DYSMORPHISM; ADMFD | HP:0001999, HP:0001263 |
| 6 | OMIM:613398 | WARSAW BREAKAGE SYNDROME; WABS | HP:0001290, HP:0001263 |
| 6 | OMIM:613404 | ARTHROGRYPOSIS, RENAL DYSFUNCTION, AND CHOLESTASIS 2; ARCS2 | HP:0001290, HP:0001263 |
| 6 | OMIM:613406 | WITTEVEEN-KOLK SYNDROME; WITKOS | HP:0001290, HP:0001263 |
| 6 | OMIM:613559 | COMBINED OXIDATIVE PHOSPHORYLATION DEFICIENCY 7; COXPD7 | HP:0001290, HP:0001263 |
| 6 | OMIM:613563 | NOONAN SYNDROME-LIKE DISORDER WITH OR WITHOUT JUVENILE MYELOMONOCYTIC LEUKEMIA; NSLL | HP:0001290, HP:0001263 |
| 6 | OMIM:613680 | BEAULIEU-BOYCOTT-INNES SYNDROME; BBIS | HP:0001999, HP:0001263 |
| 6 | OMIM:613684 | RUBINSTEIN-TAYBI SYNDROME 2; RSTS2 | HP:0001290, HP:0001263 |
| 6 | OMIM:613720 | EPILEPTIC ENCEPHALOPATHY, EARLY INFANTILE, 7; EIEE7 | HP:0001290, HP:0001263 |
| 6 | OMIM:613735 | BRAIN MALFORMATIONS WITH OR WITHOUT URINARY TRACT DEFECTS; BRMUTD | HP:0001290, HP:0001263 |
| 6 | OMIM:613752 | HYPERMETHIONINEMIA WITH S-ADENOSYLHOMOCYSTEINE HYDROLASE DEFICIENCY | HP:0001999, HP:0001263 |
| 6 | OMIM:613839 | MEGALOBLASTIC ANEMIA DUE TO DIHYDROFOLATE REDUCTASE DEFICIENCY | HP:0001290, HP:0001263 |
| 6 | OMIM:613970 | MENTAL RETARDATION, AUTOSOMAL DOMINANT 6, WITH OR WITHOUT SEIZURES; MRD6 | HP:0001290, HP:0001263 |
| 6 | OMIM:614052 | MITOCHONDRIAL COMPLEX V (ATP SYNTHASE) DEFICIENCY, NUCLEAR TYPE 2; MC5DN2 | HP:0001290, HP:0001263 |
| 6 | OMIM:614055 | ACETYL-CoA ACETYLTRANSFERASE-2 DEFICIENCY; ACAT2D | HP:0001290, HP:0001263 |
| 6 | OMIM:614063 | N-ACETYLASPARTATE DEFICIENCY; NACED | HP:0001290, HP:0001263 |
| 6 | OMIM:614080 | MULTIPLE CONGENITAL ANOMALIES-HYPOTONIA-SEIZURES SYNDROME 1; MCAHS1 | HP:0001290, HP:0001263 |
| 6 | OMIM:614207 | HYPERPHOSPHATASIA WITH MENTAL RETARDATION SYNDROME 3; HPMRS3 | HP:0001290, HP:0001263 |
| 6 | OMIM:614219 | ADAMS-OLIVER SYNDROME 2; AOS2 | HP:0001290, HP:0001263 |
| 6 | OMIM:614299 | MULTIPLE MITOCHONDRIAL DYSFUNCTIONS SYNDROME 2 WITH HYPERGLYCINEMIA; MMDS2 | HP:0001290, HP:0001263 |
| 6 | OMIM:614300 | HYPERMETHIONINEMIA DUE TO ADENOSINE KINASE DEFICIENCY | HP:0001290, HP:0001263 |
| 6 | OMIM:614340 | MENTAL RETARDATION, AUTOSOMAL RECESSIVE 27; MRT27 | HP:0001290, HP:0001263 |
| 6 | OMIM:614388 | ENCEPHALOPATHY DUE TO DEFECTIVE MITOCHONDRIAL AND PEROXISOMAL FISSION 1; EMPF1 | HP:0001290, HP:0001263 |
| 6 | OMIM:614424 | JOUBERT SYNDROME 14; JBTS14 | HP:0001290, HP:0001263 |
| 6 | OMIM:614464 | JOUBERT SYNDROME 15; JBTS15 | HP:0001290, HP:0001263 |
| 6 | OMIM:614482 | CONGENITAL CATARACTS, HEARING LOSS, AND NEURODEGENERATION; CCHLND | HP:0001290, HP:0001263 |
| 6 | OMIM:614507 | CONGENITAL DISORDER OF GLYCOSYLATION, TYPE Ir; CDG1R | HP:0001290, HP:0001263 |
| 6 | OMIM:614558 | EPILEPTIC ENCEPHALOPATHY, EARLY INFANTILE, 13; EIEE13 | HP:0001290, HP:0001263 |
| 6 | OMIM:614576 | CONGENITAL DISORDER OF GLYCOSYLATION, TYPE IIl; CDG2L | HP:0001290, HP:0001263 |
| 6 | OMIM:614607 | COFFIN-SIRIS SYNDROME 2; CSS2 | HP:0001290, HP:0001263 |
| 6 | OMIM:614608 | COFFIN-SIRIS SYNDROME 3; CSS3 | HP:0001290, HP:0001263 |
| 6 | OMIM:614609 | COFFIN-SIRIS SYNDROME 4; CSS4 | HP:0001290, HP:0001263 |
| 6 | OMIM:614654 | COENZYME Q10 DEFICIENCY, PRIMARY, 5; COQ10D5 | HP:0001290, HP:0001263 |
| 6 | OMIM:614678 | PONTOCEREBELLAR HYPOPLASIA, TYPE 1B; PCH1B | HP:0001290, HP:0001263 |
| 6 | OMIM:614702 | COMBINED OXIDATIVE PHOSPHORYLATION DEFICIENCY 10; COXPD10 | HP:0001290, HP:0001263 |
| 6 | OMIM:614727 | CONGENITAL DISORDER OF GLYCOSYLATION, TYPE IIk; CDG2K | HP:0001290, HP:0001263 |
| 6 | OMIM:614739 | 3-METHYLGLUTACONIC ACIDURIA WITH DEAFNESS, ENCEPHALOPATHY, AND LEIGH-LIKE SYNDROME; MEGDEL | HP:0001290, HP:0001263 |
| 6 | OMIM:614741 | MITOCHONDRIAL PYRUVATE CARRIER DEFICIENCY; MPYCD | HP:0001290, HP:0001263 |
| 6 | OMIM:614830 | MUSCULAR DYSTROPHY-DYSTROGLYCANOPATHY (CONGENITAL WITH BRAIN AND EYE ANOMALIES), TYPE A, 8; MDDGA8 | HP:0001290, HP:0001263 |
| 6 | OMIM:614831 | SPINOCEREBELLAR ATAXIA, AUTOSOMAL RECESSIVE 13; SCAR13 | HP:0001290, HP:0001263 |
| 6 | OMIM:614863 | PEROXISOME BIOGENESIS DISORDER 4B; PBD4B | HP:0001290, HP:0001263 |
| 6 | OMIM:614866 | PEROXISOME BIOGENESIS DISORDER 5A (ZELLWEGER); PBD5A | HP:0001290, HP:0001263 |
| 6 | OMIM:614867 | PEROXISOME BIOGENESIS DISORDER 5B; PBD5B | HP:0001290, HP:0001263 |
| 6 | OMIM:614886 | PEROXISOME BIOGENESIS DISORDER 12A (ZELLWEGER); PBD12A | HP:0001290, HP:0001263 |
| 6 | OMIM:614922 | COMBINED OXIDATIVE PHOSPHORYLATION DEFICIENCY 11; COXPD11 | HP:0001290, HP:0001263 |
| 6 | OMIM:614946 | COMBINED OXIDATIVE PHOSPHORYLATION DEFICIENCY 14; COXPD14 | HP:0001290, HP:0001263 |
| 6 | OMIM:614961 | PONTOCEREBELLAR HYPOPLASIA, TYPE 8; PCH8 | HP:0001290, HP:0001263 |
| 6 | OMIM:614969 | PONTOCEREBELLAR HYPOPLASIA, TYPE 7; PCH7 | HP:0001290, HP:0001263 |
| 6 | OMIM:615006 | EPILEPTIC ENCEPHALOPATHY, EARLY INFANTILE, 15; EIEE15 | HP:0001290, HP:0001263 |
| 6 | OMIM:615009 | SCHUURS-HOEIJMAKERS SYNDROME; SHMS | HP:0001290, HP:0001263 |
| 6 | OMIM:615031 | SPASTIC PARAPLEGIA 49, AUTOSOMAL RECESSIVE; SPG49 | HP:0001290, HP:0001263 |
| 6 | OMIM:615042 | CONGENITAL DISORDER OF GLYCOSYLATION, TYPE Iu; CDG1U | HP:0001290, HP:0001263 |
| 6 | OMIM:615075 | NEURODEVELOPMENTAL DISORDER WITH SPASTIC DIPLEGIA AND VISUAL DEFECTS; NEDSDV | HP:0001290, HP:0001263 |
| 6 | OMIM:615159 | MITOCHONDRIAL COMPLEX III DEFICIENCY, NUCLEAR TYPE 4; MC3DN4 | HP:0001290, HP:0001263 |
| 6 | OMIM:615338 | EPILEPTIC ENCEPHALOPATHY, EARLY INFANTILE, 16; EIEE16 | HP:0001290, HP:0001263 |
| 6 | OMIM:615351 | MUSCULAR DYSTROPHY-DYSTROGLYCANOPATHY (CONGENITAL WITH MENTAL RETARDATION), TYPE B, 14; MDDGB14 | HP:0001290, HP:0001263 |
| 6 | OMIM:615356 | MUSCULAR DYSTROPHY, LIMB-GIRDLE, AUTOSOMAL RECESSIVE 18; LGMDR18 | HP:0001290, HP:0001263 |
| 6 | OMIM:615398 | MULTIPLE CONGENITAL ANOMALIES-HYPOTONIA-SEIZURES SYNDROME 3; MCAHS3 | HP:0001290, HP:0001263 |
| 6 | OMIM:615438 | INFANTILE LIVER FAILURE SYNDROME 1; ILFS1 | HP:0001290, HP:0001263 |
| 6 | OMIM:615440 | COMBINED OXIDATIVE PHOSPHORYLATION DEFICIENCY 17; COXPD17 | HP:0001290, HP:0001263 |
| 6 | OMIM:615471 | MITOCHONDRIAL DNA DEPLETION SYNDROME 13 (ENCEPHALOMYOPATHIC TYPE); MTDPS13 | HP:0001290, HP:0001263 |
| 6 | OMIM:615476 | EPILEPTIC ENCEPHALOPATHY, EARLY INFANTILE, 18; EIEE18 | HP:0001290, HP:0001263 |
| 6 | OMIM:615501 | MOLYBDENUM COFACTOR DEFICIENCY, COMPLEMENTATION GROUP C; MOCODC | HP:0001290, HP:0001263 |
| 6 | OMIM:615502 | MENTAL RETARDATION, AUTOSOMAL DOMINANT 21; MRD21 | HP:0001290, HP:0001263 |
| 6 | OMIM:615512 | TRIOSEPHOSPHATE ISOMERASE DEFICIENCY; TPID | HP:0001290, HP:0001263 |
| 6 | OMIM:615553 | ARTHROGRYPOSIS, MENTAL RETARDATION, AND SEIZURES; AMRS | HP:0001290, HP:0001263 |
| 6 | OMIM:615596 | CONGENITAL DISORDER OF GLYCOSYLATION, TYPE Iw; CDG1W | HP:0001290, HP:0001263 |
| 6 | OMIM:615597 | CONGENITAL DISORDER OF GLYCOSYLATION, TYPE Ix; CDG1X | HP:0001290, HP:0001263 |
| 6 | OMIM:615636 | JOUBERT SYNDROME 21; JBTS21 | HP:0001290, HP:0001263 |
| 6 | OMIM:615637 | MENTAL RETARDATION, AUTOSOMAL RECESSIVE 41; MRT41 | HP:0001290, HP:0001263 |
| 6 | OMIM:615665 | JOUBERT SYNDROME 22; JBTS22 | HP:0001999, HP:0001263 |
| 6 | OMIM:615716 | HYPERPHOSPHATASIA WITH MENTAL RETARDATION SYNDROME 4; HPMRS4 | HP:0001290, HP:0001263 |
| 6 | OMIM:615760 | MICROCEPHALY, PROGRESSIVE, WITH SEIZURES AND CEREBRAL AND CEREBELLAR ATROPHY; MSCCA | HP:0001290, HP:0001263 |
| 6 | OMIM:615763 | CORTICAL DYSPLASIA, COMPLEX, WITH OTHER BRAIN MALFORMATIONS 5; CDCBM5 | HP:0001290, HP:0001263 |
| 6 | OMIM:615816 | IMMUNODEFICIENCY 23; IMD23 | HP:0001290, HP:0001263 |
| 6 | OMIM:615829 | XIA-GIBBS SYNDROME | HP:0001290, HP:0001263 |
| 6 | OMIM:615833 | EPILEPTIC ENCEPHALOPATHY, EARLY INFANTILE, 21; EIEE21 | HP:0001290, HP:0001263 |
| 6 | OMIM:615834 | MENTAL RETARDATION, AUTOSOMAL DOMINANT 26; MRD26 | HP:0001999, HP:0001263 |
| 6 | OMIM:615838 | MITOCHONDRIAL COMPLEX III DEFICIENCY, NUCLEAR TYPE 8; MC3DN8 | HP:0001290, HP:0001263 |
| 6 | OMIM:615846 | AICARDI-GOUTIERES SYNDROME 7; AGS7 | HP:0001290, HP:0001263 |
| 6 | OMIM:615873 | HELSMOORTEL-VAN DER AA SYNDROME; HVDAS | HP:0001290, HP:0001263 |
| 6 | OMIM:615905 | EPILEPTIC ENCEPHALOPATHY, EARLY INFANTILE, 25, WITH AMELOGENESIS IMPERFECTA; EIEE25 | HP:0001290, HP:0001263 |
| 6 | OMIM:615918 | COMBINED OXIDATIVE PHOSPHORYLATION DEFICIENCY 21; COXPD21 | HP:0001290, HP:0001263 |
| 6 | OMIM:616056 | EPILEPTIC ENCEPHALOPATHY, EARLY INFANTILE, 26; EIEE26 | HP:0001290, HP:0001263 |
| 6 | OMIM:616083 | MENTAL RETARDATION, AUTOSOMAL DOMINANT 30; MRD30 | HP:0001999, HP:0001263 |
| 6 | OMIM:616084 | SIDEROBLASTIC ANEMIA WITH B-CELL IMMUNODEFICIENCY, PERIODIC FEVERS, AND DEVELOPMENTAL DELAY; SIFD | HP:0001290, HP:0001263 |
| 6 | OMIM:616111 | MITOCHONDRIAL COMPLEX III DEFICIENCY, NUCLEAR TYPE 9; MC3DN9 | HP:0001290, HP:0001263 |
| 6 | OMIM:616116 | MENTAL RETARDATION, AUTOSOMAL RECESSIVE 46; MRT46 | HP:0001290, HP:0001263 |
| 6 | OMIM:616127 | SPINOCEREBELLAR ATAXIA, AUTOSOMAL RECESSIVE 17; SCAR17 | HP:0001290, HP:0001263 |
| 6 | OMIM:616139 | EPILEPTIC ENCEPHALOPATHY, EARLY INFANTILE, 27; EIEE27 | HP:0001290, HP:0001263 |
| 6 | OMIM:616154 | PEROXISOMAL FATTY ACYL-CoA REDUCTASE 1 DISORDER; PFCRD | HP:0001290, HP:0001263 |
| 6 | OMIM:616198 | COMBINED OXIDATIVE PHOSPHORYLATION DEFICIENCY 23; COXPD23 | HP:0001290, HP:0001263 |
| 6 | OMIM:616204 | SPINOCEREBELLAR ATAXIA, AUTOSOMAL RECESSIVE 18; SCAR18 | HP:0001290, HP:0001263 |
| 6 | OMIM:616263 | NEUROLOGIC, ENDOCRINE, AND PANCREATIC DISEASE, MULTISYSTEM, INFANTILE-ONSET; IMNEPD | HP:0001290, HP:0001263 |
| 6 | OMIM:616266 | CONGENITAL CONTRACTURES OF THE LIMBS AND FACE, HYPOTONIA, AND DEVELOPMENTAL DELAY; CLIFAHDD | HP:0001290, HP:0001263 |
| 6 | OMIM:616277 | MITOCHONDRIAL SHORT-CHAIN ENOYL-CoA HYDRATASE 1 DEFICIENCY; ECHS1D | HP:0001290, HP:0001263 |
| 6 | OMIM:616281 | MENTAL RETARDATION, AUTOSOMAL RECESSIVE 49; MRT49 | HP:0001290, HP:0001263 |
| 6 | OMIM:616339 | EPILEPTIC ENCEPHALOPATHY, EARLY INFANTILE, 29; EIEE29 | HP:0001290, HP:0001263 |
| 6 | OMIM:616354 | SPINOCEREBELLAR ATAXIA, AUTOSOMAL RECESSIVE 20; SCAR20 | HP:0001290, HP:0001263 |
| 6 | OMIM:616355 | MENTAL RETARDATION, AUTOSOMAL DOMINANT 35; MRD35 | HP:0001290, HP:0001263 |
| 6 | OMIM:616362 | MENTAL RETARDATION, AUTOSOMAL DOMINANT 36; MRD36 | HP:0001290, HP:0001263 |
| 6 | OMIM:616364 | WHITE-SUTTON SYNDROME; WHSUS | HP:0001290, HP:0001263 |
| 6 | OMIM:616430 | COMBINED OXIDATIVE PHOSPHORYLATION DEFICIENCY 25; COXPD25 | HP:0001290, HP:0001263 |
| 6 | OMIM:616449 | BASEL-VANAGAITE-SMIRIN-YOSEF SYNDROME; BVSYS | HP:0001290, HP:0001263 |
| 6 | OMIM:616457 | EPILEPTIC ENCEPHALOPATHY, EARLY INFANTILE, 50; EIEE50 | HP:0001290, HP:0001263 |
| 6 | OMIM:616459 | AL-RAQAD SYNDROME; ARS | HP:0001290, HP:0001263 |
| 6 | OMIM:616486 | MICROCEPHALY 15, PRIMARY, AUTOSOMAL RECESSIVE; MCPH15 | HP:0001290, HP:0001263 |
| 6 | OMIM:616505 | NEUROPATHY, HEREDITARY MOTOR AND SENSORY, TYPE VIB, WITH OPTIC ATROPHY; HMSN6B | HP:0001290, HP:0001263 |
| 6 | OMIM:616538 | MUSCULAR DYSTROPHY-DYSTROGLYCANOPATHY (CONGENITAL WITH BRAIN AND EYE ANOMALIES), TYPE A, 9; MDDGA9 | HP:0001290, HP:0001263 |
| 6 | OMIM:616539 | COMBINED OXIDATIVE PHOSPHORYLATION DEFICIENCY 26; COXPD26 | HP:0001290, HP:0001263 |
| 6 | OMIM:616579 | MENTAL RETARDATION, AUTOSOMAL DOMINANT 40; MRD40 | HP:0001290, HP:0001263 |
| 6 | OMIM:616580 | AU-KLINE SYNDROME; AUKS | HP:0001290, HP:0001263 |
| 6 | OMIM:616586 | SPASTIC PARAPLEGIA 9B, AUTOSOMAL RECESSIVE; SPG9B | HP:0001999, HP:0001263 |
| 6 | OMIM:616602 | CRANIOSYNOSTOSIS 6; CRS6 | HP:0001290, HP:0001263 |
| 6 | OMIM:616603 | CUTIS LAXA, AUTOSOMAL DOMINANT 3; ADCL3 | HP:0001290, HP:0001263 |
| 6 | OMIM:616654 | JOUBERT SYNDROME 24; JBTS24 | HP:0001290, HP:0001263 |
| 6 | OMIM:616657 | SPASTIC TETRAPLEGIA, THIN CORPUS CALLOSUM, AND PROGRESSIVE MICROCEPHALY; SPATCCM | HP:0001290, HP:0001263 |
| 6 | OMIM:616682 | SEIZURES, SCOLIOSIS, AND MACROCEPHALY/MICROCEPHALY SYNDROME; SSMS | HP:0001290, HP:0001263 |
| 6 | OMIM:616721 | CONGENITAL DISORDER OF GLYCOSYLATION, TYPE IIn; CDG2N | HP:0001290, HP:0001263 |
| 6 | OMIM:616733 | COENZYME Q10 DEFICIENCY, PRIMARY, 8; COQ10D8 | HP:0001290, HP:0001263 |
| 6 | OMIM:616737 | TAKENOUCHI-KOSAKI SYNDROME; TKS | HP:0001999, HP:0001290 |
| 6 | OMIM:616756 | SPASTIC PARAPLEGIA AND PSYCHOMOTOR RETARDATION WITH OR WITHOUT SEIZURES; SPPRS | HP:0001290, HP:0001263 |
| 6 | OMIM:616789 | MENTAL RETARDATION AND DISTINCTIVE FACIAL FEATURES WITH OR WITHOUT CARDIAC DEFECTS; MRFACD | HP:0001290, HP:0001263 |
| 6 | OMIM:616803 | LAMB-SHAFFER SYNDROME; LAMSHF | HP:0001290, HP:0001263 |
| 6 | OMIM:616811 | COMBINED OXIDATIVE PHOSPHORYLATION DEFICIENCY 29; COXPD29 | HP:0001290, HP:0001263 |
| 6 | OMIM:616831 | LUSCAN-LUMISH SYNDROME; LLS | HP:0001290, HP:0001263 |
| 6 | OMIM:616881 | LEUKODYSTROPHY, HYPOMYELINATING, 13; HLD13 | HP:0001290, HP:0001263 |
| 6 | OMIM:616917 | MENTAL RETARDATION, AUTOSOMAL RECESSIVE 53; MRT53 | HP:0001290, HP:0001263 |
| 6 | OMIM:616920 | HEART AND BRAIN MALFORMATION SYNDROME; HBMS | HP:0001290, HP:0001263 |
| 6 | OMIM:616954 | YOU-HOOVER-FONG SYNDROME; YHFS | HP:0001290, HP:0001263 |
| 6 | OMIM:616973 | MENTAL RETARDATION, AUTOSOMAL DOMINANT 42; MRD42 | HP:0001290, HP:0001263 |
| 6 | OMIM:616975 | NEURODEVELOPMENTAL DISORDER WITH OR WITHOUT ANOMALIES OF THE BRAIN, EYE, OR HEART; NEDBEH | HP:0001290, HP:0001263 |
| 6 | OMIM:616981 | EPILEPTIC ENCEPHALOPATHY, EARLY INFANTILE, 37; EIEE37 | HP:0001290, HP:0001263 |
| 6 | OMIM:617011 | MACROCEPHALY, DYSMORPHIC FACIES, AND PSYCHOMOTOR RETARDATION; MDFPMR | HP:0001290, HP:0001263 |
| 6 | OMIM:617020 | EPILEPTIC ENCEPHALOPATHY, EARLY INFANTILE, 38; EIEE38 | HP:0001290, HP:0001263 |
| 6 | OMIM:617051 | MENTAL RETARDATION, AUTOSOMAL RECESSIVE 55; MRT55 | HP:0001290, HP:0001263 |
| 6 | OMIM:617062 | OKUR-CHUNG NEURODEVELOPMENTAL SYNDROME; OCNDS | HP:0001290, HP:0001263 |
| 6 | OMIM:617082 | CONGENITAL DISORDER OF GLYCOSYLATION, TYPE Iaa; CDG1AA | HP:0001290, HP:0001263 |
| 6 | OMIM:617093 | GROWTH RETARDATION, IMPAIRED INTELLECTUAL DEVELOPMENT, HYPOTONIA, AND HEPATOPATHY; GRIDHH | HP:0001290, HP:0001263 |
| 6 | OMIM:617105 | EPILEPTIC ENCEPHALOPATHY, EARLY INFANTILE, 41; EIEE41 | HP:0001290, HP:0001263 |
| 6 | OMIM:617106 | EPILEPTIC ENCEPHALOPATHY, EARLY INFANTILE, 42; EIEE42 | HP:0001290, HP:0001263 |
| 6 | OMIM:617113 | EPILEPTIC ENCEPHALOPATHY, EARLY INFANTILE, 43; EIEE43 | HP:0001290, HP:0001263 |
| 6 | OMIM:617120 | JOUBERT SYNDROME 27; JBTS27 | HP:0001290, HP:0001263 |
| 6 | OMIM:617121 | JOUBERT SYNDROME 28; JBTS28 | HP:0001290, HP:0001263 |
| 6 | OMIM:617140 | ZTTK SYNDROME; ZTTKS | HP:0001290, HP:0001263 |
| 6 | OMIM:617153 | EPILEPTIC ENCEPHALOPATHY, EARLY INFANTILE, 45; EIEE45 | HP:0001290, HP:0001263 |
| 6 | OMIM:617157 | SHORT STATURE, BRACHYDACTYLY, INTELLECTUAL DEVELOPMENTAL DISABILITY, AND SEIZURES; SBIDDS | HP:0001290, HP:0001263 |
| 6 | OMIM:617171 | DYSKINESIA, SEIZURES, AND INTELLECTUAL DEVELOPMENTAL DISORDER; DYSEIDD | HP:0001290, HP:0001263 |
| 6 | OMIM:617173 | INTELLECTUAL DEVELOPMENTAL DISORDER WITH CARDIAC ARRHYTHMIA; IDDCA | HP:0001290, HP:0001263 |
| 6 | OMIM:617183 | HAREL-YOON SYNDROME; HAYOS | HP:0001290, HP:0001263 |
| 6 | OMIM:617188 | MENTAL RETARDATION, AUTOSOMAL RECESSIVE 57; MRT57 | HP:0001290, HP:0001263 |
| 6 | OMIM:617190 | SHASHI-PENA SYNDROME; SHAPNS | HP:0001290, HP:0001263 |
| 6 | OMIM:617228 | COMBINED OXIDATIVE PHOSPHORYLATION DEFICIENCY 31; COXPD31 | HP:0001290, HP:0001263 |
| 6 | OMIM:617237 | IMMUNODEFICIENCY 49; IMD49 | HP:0001290, HP:0001263 |
| 6 | OMIM:617260 | GLOBAL DEVELOPMENTAL DELAY, ABSENT OR HYPOPLASTIC CORPUS CALLOSUM, AND DYSMORPHIC FACIES; GDACCF | HP:0001290, HP:0001263 |
| 6 | OMIM:617276 | EPILEPTIC ENCEPHALOPATHY, EARLY INFANTILE, 48; EIEE48 | HP:0001290, HP:0001263 |
| 6 | OMIM:617330 | HYPOTONIA, ATAXIA, AND DELAYED DEVELOPMENT SYNDROME; HADDS | HP:0001290, HP:0001263 |
| 6 | OMIM:617333 | INTELLECTUAL DEVELOPMENTAL DISORDER WITH DYSMORPHIC FACIES AND PTOSIS; IDDDFP | HP:0001290, HP:0001263 |
| 6 | OMIM:617350 | EPILEPTIC ENCEPHALOPATHY, EARLY INFANTILE, 52; EIEE52 | HP:0001290, HP:0001263 |
| 6 | OMIM:617360 | CONGENITAL HEART DEFECTS, DYSMORPHIC FACIAL FEATURES, AND INTELLECTUAL DEVELOPMENTAL DISORDER; CHDFIDD | HP:0001290, HP:0001263 |
| 6 | OMIM:617364 | CONGENITAL HEART DEFECTS AND ECTODERMAL DYSPLASIA; CHDED | HP:0001290, HP:0001263 |
| 6 | OMIM:617370 | PEROXISOME BIOGENESIS DISORDER 10B; PBD10B | HP:0001290, HP:0001263 |
| 6 | OMIM:617391 | EPILEPTIC ENCEPHALOPATHY, EARLY INFANTILE, 54; EIEE54 | HP:0001290, HP:0001263 |
| 6 | OMIM:617393 | NEURODEVELOPMENTAL DISORDER WITH EPILEPSY, CATARACTS, FEEDING DIFFICULTIES, AND DELAYED BRAIN MYELINATION; NECFM | HP:0001290, HP:0001263 |
| 6 | OMIM:617404 | MUSCULAR DYSTROPHY, CONGENITAL, WITH CATARACTS AND INTELLECTUAL DISABILITY; MDCCAID | HP:0001290, HP:0001263 |
| 6 | OMIM:617450 | JANSEN-DE VRIES SYNDROME; JDVS | HP:0001290, HP:0001263 |
| 6 | OMIM:617493 | NEURODEVELOPMENTAL DISORDER WITH INVOLUNTARY MOVEMENTS; NEDIM | HP:0001290, HP:0001263 |
| 6 | OMIM:617506 | NOONAN SYNDROME-LIKE DISORDER WITH LOOSE ANAGEN HAIR 2; NSLH2 | HP:0001290, HP:0001263 |
| 6 | OMIM:617516 | STANKIEWICZ-ISIDOR SYNDROME; STISS | HP:0001290, HP:0001263 |
| 6 | OMIM:617532 | INTELLECTUAL DEVELOPMENTAL DISORDER WITH NEUROPSYCHIATRIC FEATURES; IDDNPF | HP:0001290, HP:0001263 |
| 6 | OMIM:617542 | GAZE PALSY, FAMILIAL HORIZONTAL, WITH PROGRESSIVE SCOLIOSIS 2, WITH IMPAIRED INTELLECTUAL DEVELOPMENT; HGPPS2 | HP:0001290, HP:0001263 |
| 6 | OMIM:617561 | COHEN-GIBSON SYNDROME; COGIS | HP:0001290, HP:0001263 |
| 6 | OMIM:617563 | OROFACIODIGITAL SYNDROME XVI; OFD16 | HP:0001290, HP:0001263 |
| 6 | OMIM:617575 | NEPHROTIC SYNDROME, TYPE 14; NPHS14 | HP:0001290, HP:0001263 |
| 6 | OMIM:617600 | MENTAL RETARDATION, AUTOSOMAL DOMINANT 45; MRD45 | HP:0001290, HP:0001263 |
| 6 | OMIM:617601 | MENTAL RETARDATION, AUTOSOMAL DOMINANT 46; MRD46 | HP:0001290, HP:0001263 |
| 6 | OMIM:617616 | SKRABAN-DEARDORFF SYNDROME; SKDEAS | HP:0001290, HP:0001263 |
| 6 | OMIM:617622 | JOUBERT SYNDROME 30; JBTS30 | HP:0001290, HP:0001263 |
| 6 | OMIM:617635 | MENTAL RETARDATION, AUTOSOMAL DOMINANT 47; MRD47 | HP:0001290, HP:0001263 |
| 6 | OMIM:617643 | CEREBELLAR ATROPHY, DEVELOPMENTAL DELAY, AND SEIZURES; CADEDS | HP:0001290, HP:0001263 |
| 6 | OMIM:617664 | COMBINED OXIDATIVE PHOSPHORYLATION DEFICIENCY 32; COXPD32 | HP:0001290, HP:0001263 |
| 6 | OMIM:617682 | PILAROWSKI-BJORNSSON SYNDROME; PILBOS | HP:0001290, HP:0001263 |
| 6 | OMIM:617694 | AL KAISSI SYNDROME; ALKAS | HP:0001290, HP:0001263 |
| 6 | OMIM:617695 | PONTOCEREBELLAR HYPOPLASIA, TYPE 11; PCH11 | HP:0001290, HP:0001263 |
| 6 | OMIM:617698 | 3-METHYLGLUTACONIC ACIDURIA, TYPE IX; MGCA9 | HP:0001290, HP:0001263 |
| 6 | OMIM:617711 | EPILEPTIC ENCEPHALOPATHY, INFANTILE OR EARLY CHILDHOOD, 1; IECEE1 | HP:0001290, HP:0001263 |
| 6 | OMIM:617729 | GALLOWAY-MOWAT SYNDROME 3; GAMOS3 | HP:0001290, HP:0001263 |
| 6 | OMIM:617730 | GALLOWAY-MOWAT SYNDROME 4; GAMOS4 | HP:0001290, HP:0001263 |
| 6 | OMIM:617731 | GALLOWAY-MOWAT SYNDROME 5; GAMOS5 | HP:0001999, HP:0001263 |
| 6 | OMIM:617751 | MENTAL RETARDATION, AUTOSOMAL DOMINANT 48; MRD48 | HP:0001999, HP:0001290 |
| 6 | OMIM:617755 | NEURODEVELOPMENTAL DISORDER WITH DYSMORPHIC FACIES AND DISTAL LIMB ANOMALIES; NEDDFL | HP:0001290, HP:0001263 |
| 6 | OMIM:617757 | JOUBERT SYNDROME 32; JBTS32 | HP:0001290, HP:0001263 |
| 6 | OMIM:617761 | JOUBERT SYNDROME 31; JBTS31 | HP:0001290, HP:0001263 |
| 6 | OMIM:617771 | EPILEPTIC ENCEPHALOPATHY, EARLY INFANTILE, 57; EIEE57 | HP:0001290, HP:0001263 |
| 6 | OMIM:617798 | MENTAL RETARDATION, AUTOSOMAL DOMINANT 53; MRD53 | HP:0001290, HP:0001263 |
| 6 | OMIM:617802 | NEURODEVELOPMENTAL DISORDER WITH MICROCEPHALY, SEIZURES, AND CORTICAL ATROPHY; NDMSCA | HP:0001290, HP:0001263 |
| 6 | OMIM:617808 | COFFIN-SIRIS SYNDROME 6; CSS6 | HP:0001999, HP:0001290 |
| 6 | OMIM:617810 | GLYCOSYLPHOSPHATIDYLINOSITOL BIOSYNTHESIS DEFECT 15; GPIBD15 | HP:0001290, HP:0001263 |
| 6 | OMIM:617820 | NEURODEVELOPMENTAL DISORDER WITH OR WITHOUT HYPERKINETIC MOVEMENTS AND SEIZURES, AUTOSOMAL RECESSIVE; NDHMSR | HP:0001290, HP:0001263 |
| 6 | OMIM:617822 | ALKURAYA-KUCINSKAS SYNDROME; ALKKUCS | HP:0001290, HP:0001263 |
| 6 | OMIM:617830 | EPILEPTIC ENCEPHALOPATHY, EARLY INFANTILE, 58; EIEE58 | HP:0001290, HP:0001263 |
| 6 | OMIM:617854 | MENTAL RETARDATION, AUTOSOMAL DOMINANT 56; MRD56 | HP:0001290, HP:0001263 |
| 6 | OMIM:617862 | NEURODEVELOPMENTAL DISORDER WITH MICROCEPHALY, EPILEPSY, AND BRAIN ATROPHY; NEDMEBA | HP:0001290, HP:0001263 |
| 6 | OMIM:617865 | NEURODEVELOPMENTAL DISORDER WITH MOVEMENT ABNORMALITIES, ABNORMAL GAIT, AND AUTISTIC FEATURES; NEDMAGA | HP:0001290, HP:0001263 |
| 6 | OMIM:617873 | COMBINED OXIDATIVE PHOSPHORYLATION DEFICIENCY 35; COXPD35 | HP:0001290, HP:0001263 |
| 6 | OMIM:617931 | SPINOCEREBELLAR ATAXIA 47; SCA47 | HP:0001999, HP:0001290 |
| 6 | OMIM:617938 | EPILEPTIC ENCEPHALOPATHY, EARLY INFANTILE, 62; EIEE62 | HP:0001290, HP:0001263 |
| 6 | OMIM:617950 | COMBINED OXIDATIVE PHOSPHORYLATION DEFICIENCY 36; COXPD36 | HP:0001290, HP:0001263 |
| 6 | OMIM:617954 | MULTIPLE MITOCHONDRIAL DYSFUNCTIONS SYNDROME 6; MMDS6 | HP:0001290, HP:0001263 |
| 6 | OMIM:617982 | VERVERI-BRADY SYNDROME; VERBRAS | HP:0001999, HP:0001290 |
| 6 | OMIM:617991 | DEVELOPMENTAL DELAY, INTELLECTUAL DISABILITY, OBESITY, AND DYSMORPHISM; DIDOD | HP:0001290, HP:0001263 |
| 6 | OMIM:618012 | EPILEPTIC ENCEPHALOPATHY, INFANTILE OR EARLY CHILDHOOD, 3; IECEE3 | HP:0001290, HP:0001263 |
| 6 | OMIM:618027 | COFFIN-SIRIS SYNDROME 7; CSS7 | HP:0001290, HP:0001263 |
| 6 | OMIM:618050 | MENTAL RETARDATION, AUTOSOMAL DOMINANT 57; MRD57 | HP:0001290, HP:0001263 |
| 6 | OMIM:618060 | INTELLECTUAL DEVELOPMENTAL DISORDER WITH OR WITHOUT EPILEPSY OR CEREBELLAR ATAXIA; IDDECA | HP:0001290, HP:0001263 |
| 6 | OMIM:618067 | EPILEPTIC ENCEPHALOPATHY, EARLY INFANTILE, 66; EIEE66 | HP:0001290, HP:0001263 |
| 6 | OMIM:618088 | NEURODEVELOPMENTAL DISORDER WITH REGRESSION, ABNORMAL MOVEMENTS, LOSS OF SPEECH, AND SEIZURES; NEDAMSS | HP:0001290, HP:0001263 |
| 6 | OMIM:618089 | INTELLECTUAL DEVELOPMENTAL DISORDER WITH DYSMORPHIC FACIES AND BEHAVIORAL ABNORMALITIES; IDDFBA | HP:0001290, HP:0001263 |
| 6 | OMIM:618090 | NEURODEVELOPMENTAL DISORDER WITH EPILEPSY AND HYPOPLASIA OF THE CORPUS CALLOSUM; NEDEHCC | HP:0001290, HP:0001263 |
| 6 | OMIM:618092 | INTELLECTUAL DEVELOPMENTAL DISORDER WITH SPEECH DELAY, DYSMORPHIC FACIES, AND T-CELL ABNORMALITIES; IDDSFTA | HP:0001290, HP:0001263 |
| 6 | OMIM:618158 | INTELLECTUAL DEVELOPMENTAL DISORDER WITH MACROCEPHALY, SEIZURES, AND SPEECH DELAY; IDDMSSD | HP:0001290, HP:0001263 |
| 6 | OMIM:618174 | CORTICAL DYSPLASIA, COMPLEX, WITH OTHER BRAIN MALFORMATIONS 9; CDCBM9 | HP:0001290, HP:0001263 |
| 6 | OMIM:618205 | SNIJDERS BLOK-CAMPEAU SYNDROME; SNIBCPS | HP:0001290, HP:0001263 |
| 6 | OMIM:618213 | INFLAMMATORY BOWEL DISEASE, IMMUNODEFICIENCY, AND ENCEPHALOPATHY; IBDIMDE | HP:0001290, HP:0001263 |
| 6 | OMIM:618225 | MITOCHONDRIAL COMPLEX I DEFICIENCY, NUCLEAR TYPE 4; MC1DN4 | HP:0001290, HP:0001263 |
| 6 | OMIM:618226 | MITOCHONDRIAL COMPLEX I DEFICIENCY, NUCLEAR TYPE 5; MC1DN5 | HP:0001290, HP:0001263 |
| 6 | OMIM:618233 | MITOCHONDRIAL COMPLEX I DEFICIENCY, NUCLEAR TYPE 10; MC1DN10 | HP:0001290, HP:0001263 |
| 6 | OMIM:618238 | MITOCHONDRIAL COMPLEX I DEFICIENCY, NUCLEAR TYPE 16; MC1DN16 | HP:0001290, HP:0001263 |
| 6 | OMIM:618239 | MITOCHONDRIAL COMPLEX I DEFICIENCY, NUCLEAR TYPE 17; MC1DN17 | HP:0001290, HP:0001263 |
| 6 | OMIM:618241 | MITOCHONDRIAL COMPLEX I DEFICIENCY, NUCLEAR TYPE 19; MC1DN19 | HP:0001290, HP:0001263 |
| 6 | OMIM:618243 | MITOCHONDRIAL COMPLEX I DEFICIENCY, NUCLEAR TYPE 22; MC1DN22 | HP:0001290, HP:0001263 |
| 6 | OMIM:618246 | MITOCHONDRIAL COMPLEX I DEFICIENCY, NUCLEAR TYPE 25; MC1DN25 | HP:0001290, HP:0001263 |
| 6 | OMIM:618248 | MITOCHONDRIAL COMPLEX I DEFICIENCY, NUCLEAR TYPE 27; MC1DN27 | HP:0001290, HP:0001263 |
| 6 | OMIM:618249 | MITOCHONDRIAL COMPLEX I DEFICIENCY, NUCLEAR TYPE 28; MC1DN28 | HP:0001290, HP:0001263 |
| 6 | OMIM:618251 | MITOCHONDRIAL COMPLEX I DEFICIENCY, NUCLEAR TYPE 31; MC1DN31 | HP:0001290, HP:0001263 |
| 6 | OMIM:618253 | MITOCHONDRIAL COMPLEX I DEFICIENCY, NUCLEAR TYPE 33; MC1DN33 | HP:0001290, HP:0001263 |
| 6 | OMIM:618273 | MEGA-CORPUS-CALLOSUM SYNDROME WITH CEREBELLAR HYPOPLASIA AND CORTICAL MALFORMATIONS; MCCCHCM | HP:0001290, HP:0001263 |
| 6 | OMIM:618276 | NEURODEGENERATION, CHILDHOOD-ONSET, WITH CEREBELLAR ATROPHY; CONDCA | HP:0001290, HP:0001263 |
| 6 | OMIM:618292 | NEURODEVELOPMENTAL DISORDER WITH IMPAIRED INTELLECTUAL DEVELOPMENT, HYPOTONIA, AND ATAXIA; NEDIDHA | HP:0001290, HP:0001263 |
| 6 | OMIM:618298 | EPILEPTIC ENCEPHALOPATHY, EARLY INFANTILE, 70; EIEE70 | HP:0001290, HP:0001263 |
| 6 | OMIM:618324 | CONGENITAL DISORDER OF GLYCOSYLATION WITH DEFECTIVE FUCOSYLATION 2; CDGF2 | HP:0001290, HP:0001263 |
| 6 | OMIM:618325 | LISSENCEPHALY 9 WITH COMPLEX BRAINSTEM MALFORMATION; LIS9 | HP:0001290, HP:0001263 |
| 6 | OMIM:618329 | COMBINED OXIDATIVE PHOSPHORYLATION DEFICIENCY 37; COXPD37 | HP:0001290, HP:0001263 |
| 6 | OMIM:618349 | GALLOWAY-MOWAT SYNDROME 8; GAMOS8 | HP:0001290, HP:0001263 |
| 6 | OMIM:618354 | NEURODEVELOPMENTAL DISORDER AND LANGUAGE DELAY WITH OR WITHOUT STRUCTURAL BRAIN ABNORMALITIES; NEDLBA | HP:0001290, HP:0001263 |
| 6 | OMIM:618356 | NEURODEVELOPMENTAL DISORDER WITH CENTRAL AND PERIPHERAL MOTOR DYSFUNCTION; NEDCPMD | HP:0001290, HP:0001263 |
| 6 | OMIM:618360 | BRAIN SMALL VESSEL DISEASE 3; BSVD3 | HP:0001290, HP:0001263 |
| 6 | OMIM:618378 | COMBINED OXIDATIVE PHOSPHORYLATION DEFICIENCY 38; COXPD38 | HP:0001999, HP:0001263 |

---


---


---

# Cluster 10

| Cluster | Term | Name |
| --- | --- | --- |
| 10 | HP:0001252 | Muscular hypotonia |
| 10 | HP:0001257 | Spasticity |
| 10 | HP:0004404 | Abnormal nipple morphology |

| Cluster | Term | Name | HPOs\_in\_clusters |
| --- | --- | --- | --- |
| 10 | OMIM:124000 | MITOCHONDRIAL COMPLEX III DEFICIENCY, NUCLEAR TYPE 1; MC3DN1 | HP:0001252, HP:0001257 |
| 10 | OMIM:164400 | SPINOCEREBELLAR ATAXIA 1; SCA1 | HP:0001252, HP:0001257 |
| 10 | OMIM:183090 | SPINOCEREBELLAR ATAXIA 2; SCA2 | HP:0001252, HP:0001257 |
| 10 | OMIM:208400 | ASPARTYLGLUCOSAMINURIA; AGU | HP:0001252, HP:0001257 |
| 10 | OMIM:216360 | COACH SYNDROME | HP:0001252, HP:0001257 |
| 10 | OMIM:246450 | 3-HYDROXY-3-METHYLGLUTARYL-CoA LYASE DEFICIENCY; HMGCLD | HP:0001252, HP:0001257 |
| 10 | OMIM:248500 | MANNOSIDOSIS, ALPHA B, LYSOSOMAL; MANSA | HP:0001252, HP:0001257 |
| 10 | OMIM:252010 | MITOCHONDRIAL COMPLEX I DEFICIENCY, NUCLEAR TYPE 1; MC1DN1 | HP:0001252, HP:0001257 |
| 10 | OMIM:256000 | LEIGH SYNDROME; LS | HP:0001252, HP:0001257 |
| 10 | OMIM:256730 | CEROID LIPOFUSCINOSIS, NEURONAL, 1; CLN1 | HP:0001252, HP:0001257 |
| 10 | OMIM:257200 | NIEMANN-PICK DISEASE, TYPE A | HP:0001252, HP:0001257 |
| 10 | OMIM:257220 | NIEMANN-PICK DISEASE, TYPE C1; NPC1 | HP:0001252, HP:0001257 |
| 10 | OMIM:277590 | WEAVER SYNDROME; WVS | HP:0001252, HP:0001257 |
| 10 | OMIM:300215 | LISSENCEPHALY, X-LINKED, 2; LISX2 | HP:0001252, HP:0001257 |
| 10 | OMIM:300322 | LESCH-NYHAN SYNDROME; LNS | HP:0001252, HP:0001257 |
| 10 | OMIM:300486 | MENTAL RETARDATION, X-LINKED, WITH CEREBELLAR HYPOPLASIA AND DISTINCTIVE FACIAL APPEARANCE | HP:0001252, HP:0001257 |
| 10 | OMIM:603896 | LEUKOENCEPHALOPATHY WITH VANISHING WHITE MATTER; VWM | HP:0001252, HP:0001257 |
| 10 | OMIM:604369 | SALLA DISEASE; SD | HP:0001252, HP:0001257 |
| 10 | OMIM:607625 | NIEMANN-PICK DISEASE, TYPE C2; NPC2 | HP:0001252, HP:0001257 |
| 10 | OMIM:609241 | SCHINDLER DISEASE, TYPE I | HP:0001252, HP:0001257 |
| 10 | OMIM:612073 | MITOCHONDRIAL DNA DEPLETION SYNDROME 5 (ENCEPHALOMYOPATHIC WITH OR WITHOUT METHYLMALONIC ACIDURIA); MTDPS5 | HP:0001252, HP:0001257 |

---


---


---

# Cluster 20

| Cluster | Term | Name |
| --- | --- | --- |
| 20 | HP:0000252 | Microcephaly |
| 20 | HP:0000527 | Long eyelashes |
| 20 | HP:0001238 | Slender finger |

| Cluster | Term | Name | HPOs\_in\_clusters |
| --- | --- | --- | --- |
| 20 | OMIM:122470 | CORNELIA DE LANGE SYNDROME 1; CDLS1 | HP:0000527, HP:0000252 |
| 20 | OMIM:135900 | COFFIN-SIRIS SYNDROME 1; CSS1 | HP:0000527, HP:0000252 |
| 20 | OMIM:180849 | RUBINSTEIN-TAYBI SYNDROME 1; RSTS1 | HP:0000527, HP:0000252 |
| 20 | OMIM:212066 | CONGENITAL DISORDER OF GLYCOSYLATION, TYPE IIa; CDG2A | HP:0000527, HP:0000252 |
| 20 | OMIM:224690 | MEIER-GORLIN SYNDROME 1; MGORS1 | HP:0000527, HP:0000252 |
| 20 | OMIM:300243 | MENTAL RETARDATION, X-LINKED, SYNDROMIC, CHRISTIANSON TYPE; MRXSCH | HP:0001238, HP:0000252 |
| 20 | OMIM:300590 | CORNELIA DE LANGE SYNDROME 2; CDLS2 | HP:0000527, HP:0000252 |
| 20 | OMIM:300855 | OGDEN SYNDROME; OGDNS | HP:0000527, HP:0000252 |
| 20 | OMIM:309580 | MENTAL RETARDATION-HYPOTONIC FACIES SYNDROME, X-LINKED, 1; MRXHF1 | HP:0001238, HP:0000252 |
| 20 | OMIM:610536 | MANDIBULOFACIAL DYSOSTOSIS, GUION-ALMEIDA TYPE; MFDGA | HP:0001238, HP:0000252 |
| 20 | OMIM:613684 | RUBINSTEIN-TAYBI SYNDROME 2; RSTS2 | HP:0000527, HP:0000252 |
| 20 | OMIM:615866 | COFFIN-SIRIS SYNDROME 9; CSS9 | HP:0000527, HP:0000252 |
| 20 | OMIM:616938 | COFFIN-SIRIS SYNDROME 5; CSS5 | HP:0000527, HP:0000252 |
| 20 | OMIM:617126 | ALAZAMI-YUAN SYNDROME; ALYUS | HP:0000527, HP:0000252 |
| 20 | OMIM:617281 | EPILEPTIC ENCEPHALOPATHY, EARLY INFANTILE, 49; EIEE49 | HP:0000527, HP:0000252 |
| 20 | OMIM:617412 | BRACHYCEPHALY, TRICHOMEGALY, AND DEVELOPMENTAL DELAY; BTDD | HP:0000527, HP:0000252 |
| 20 | OMIM:617523 | NEURODEVELOPMENTAL DISORDER WITH MIDBRAIN AND HINDBRAIN MALFORMATIONS; NEDMHM | HP:0000527, HP:0000252 |
| 20 | OMIM:617755 | NEURODEVELOPMENTAL DISORDER WITH DYSMORPHIC FACIES AND DISTAL LIMB ANOMALIES; NEDDFL | HP:0001238, HP:0000252 |
| 20 | OMIM:617883 | FANCONI ANEMIA, COMPLEMENTATION GROUP S; FANCS | HP:0000527, HP:0000252 |
| 20 | OMIM:618089 | INTELLECTUAL DEVELOPMENTAL DISORDER WITH DYSMORPHIC FACIES AND BEHAVIORAL ABNORMALITIES; IDDFBA | HP:0000527, HP:0000252 |

---


---


---

# Cluster 21

| Cluster | Term | Name |
| --- | --- | --- |
| 21 | HP:0000316 | Hypertelorism |
| 21 | HP:0000582 | Upslanted palpebral fissure |
| 21 | HP:0002007 | Frontal bossing |
| 21 | HP:0000581 | Blepharophimosis |

| Cluster | Term | Name | HPOs\_in\_clusters |
| --- | --- | --- | --- |
| 21 | OMIM:613610 | CRANIOECTODERMAL DYSPLASIA 2; CED2 | HP:0002007, HP:0000582, HP:0000316, HP:0000581 |
| 21 | OMIM:145420 | HYPERTELORISM, TEEBI TYPE; TBHS | HP:0002007, HP:0000582, HP:0000316 |
| 21 | OMIM:261515 | D-BIFUNCTIONAL PROTEIN DEFICIENCY | HP:0002007, HP:0000582, HP:0000316 |
| 21 | OMIM:261540 | PETERS-PLUS SYNDROME; PTRPLS | HP:0002007, HP:0000582, HP:0000316 |
| 21 | OMIM:264090 | WIEDEMANN-RAUTENSTRAUCH SYNDROME; WDRTS | HP:0002007, HP:0000582, HP:0000316 |
| 21 | OMIM:616789 | MENTAL RETARDATION AND DISTINCTIVE FACIAL FEATURES WITH OR WITHOUT CARDIAC DEFECTS; MRFACD | HP:0002007, HP:0000582, HP:0000316 |
| 21 | OMIM:617011 | MACROCEPHALY, DYSMORPHIC FACIES, AND PSYCHOMOTOR RETARDATION; MDFPMR | HP:0002007, HP:0000582, HP:0000316 |
| 21 | OMIM:617883 | FANCONI ANEMIA, COMPLEMENTATION GROUP S; FANCS | HP:0000316, HP:0000582, HP:0000581 |

---


---


---

# Cluster 22

| Cluster | Term | Name |
| --- | --- | --- |
| 22 | HP:0000316 | Hypertelorism |
| 22 | HP:0000582 | Upslanted palpebral fissure |
| 22 | HP:0002007 | Frontal bossing |
| 22 | HP:0005280 | Depressed nasal bridge |

| Cluster | Term | Name | HPOs\_in\_clusters |
| --- | --- | --- | --- |
| 22 | OMIM:145420 | HYPERTELORISM, TEEBI TYPE; TBHS | HP:0002007, HP:0000582, HP:0005280, HP:0000316 |
| 22 | OMIM:261515 | D-BIFUNCTIONAL PROTEIN DEFICIENCY | HP:0002007, HP:0000582, HP:0005280, HP:0000316 |
| 22 | OMIM:613610 | CRANIOECTODERMAL DYSPLASIA 2; CED2 | HP:0002007, HP:0000582, HP:0005280, HP:0000316 |
| 22 | OMIM:616789 | MENTAL RETARDATION AND DISTINCTIVE FACIAL FEATURES WITH OR WITHOUT CARDIAC DEFECTS; MRFACD | HP:0002007, HP:0000582, HP:0005280, HP:0000316 |
| 22 | OMIM:113620 | BRANCHIOOCULOFACIAL SYNDROME; BOFS | HP:0000582, HP:0005280, HP:0000316 |
| 22 | OMIM:118450 | ALAGILLE SYNDROME 1; ALGS1 | HP:0000582, HP:0005280, HP:0000316 |
| 22 | OMIM:119600 | CLEIDOCRANIAL DYSPLASIA; CCD | HP:0002007, HP:0005280, HP:0000316 |
| 22 | OMIM:145410 | OPITZ GBBB SYNDROME, TYPE II; GBBB2 | HP:0002007, HP:0005280, HP:0000316 |
| 22 | OMIM:150250 | LARSEN SYNDROME; LRS | HP:0002007, HP:0005280, HP:0000316 |
| 22 | OMIM:166250 | OSTEOGLOPHONIC DYSPLASIA; OGD | HP:0002007, HP:0005280, HP:0000316 |
| 22 | OMIM:201750 | ANTLEY-BIXLER SYNDROME WITH GENITAL ANOMALIES AND DISORDERED STEROIDOGENESIS; ABS1 | HP:0002007, HP:0005280, HP:0000316 |
| 22 | OMIM:215100 | RHIZOMELIC CHONDRODYSPLASIA PUNCTATA, TYPE 1; RCDP1 | HP:0002007, HP:0000582, HP:0005280 |
| 22 | OMIM:226980 | EPIPHYSEAL DYSPLASIA, MULTIPLE, WITH EARLY-ONSET DIABETES MELLITUS | HP:0000582, HP:0005280, HP:0000316 |
| 22 | OMIM:230740 | GAPO SYNDROME; GAPOS | HP:0002007, HP:0005280, HP:0000316 |
| 22 | OMIM:245600 | MULTIPLE JOINT DISLOCATIONS, SHORT STATURE, AND CRANIOFACIAL DYSMORPHISM WITH OR WITHOUT CONGENITAL HEART DEFECTS; JDSCD | HP:0002007, HP:0005280, HP:0000316 |
| 22 | OMIM:253250 | MULIBREY NANISM | HP:0002007, HP:0005280, HP:0000316 |
| 22 | OMIM:257300 | MOSAIC VARIEGATED ANEUPLOIDY SYNDROME 1; MVA1 | HP:0000582, HP:0005280, HP:0000316 |
| 22 | OMIM:258480 | OPSISMODYSPLASIA; OPSMD | HP:0002007, HP:0005280, HP:0000316 |
| 22 | OMIM:261540 | PETERS-PLUS SYNDROME; PTRPLS | HP:0002007, HP:0000582, HP:0000316 |
| 22 | OMIM:264090 | WIEDEMANN-RAUTENSTRAUCH SYNDROME; WDRTS | HP:0002007, HP:0000582, HP:0000316 |
| 22 | OMIM:264470 | PEROXISOMAL ACYL-CoA OXIDASE DEFICIENCY | HP:0002007, HP:0005280, HP:0000316 |
| 22 | OMIM:269500 | SCLEROSTEOSIS 1; SOST1 | HP:0002007, HP:0005280, HP:0000316 |
| 22 | OMIM:271665 | SPONDYLOMETAEPIPHYSEAL DYSPLASIA, SHORT LIMB-HAND TYPE | HP:0002007, HP:0005280, HP:0000316 |
| 22 | OMIM:280000 | COLOBOMA, CONGENITAL HEART DISEASE, ICHTHYOSIFORM DERMATOSIS, MENTAL RETARDATION, AND EAR ANOMALIES SYNDROME; CHIME | HP:0002007, HP:0005280, HP:0000316 |
| 22 | OMIM:300868 | MULTIPLE CONGENITAL ANOMALIES-HYPOTONIA-SEIZURES SYNDROME 2; MCAHS2 | HP:0000582, HP:0005280, HP:0000316 |
| 22 | OMIM:304120 | OTOPALATODIGITAL SYNDROME, TYPE II; OPD2 | HP:0002007, HP:0005280, HP:0000316 |
| 22 | OMIM:309580 | MENTAL RETARDATION-HYPOTONIC FACIES SYNDROME, X-LINKED, 1; MRXHF1 | HP:0000582, HP:0005280, HP:0000316 |
| 22 | OMIM:606812 | FUMARASE DEFICIENCY; FMRD | HP:0002007, HP:0005280, HP:0000316 |
| 22 | OMIM:608091 | JOUBERT SYNDROME 2; JBTS2 | HP:0002007, HP:0005280, HP:0000316 |
| 22 | OMIM:608776 | CONGENITAL DISORDER OF GLYCOSYLATION, TYPE Il; CDG1L | HP:0002007, HP:0005280, HP:0000316 |
| 22 | OMIM:613406 | WITTEVEEN-KOLK SYNDROME; WITKOS | HP:0000582, HP:0005280, HP:0000316 |
| 22 | OMIM:613443 | MENTAL RETARDATION, AUTOSOMAL DOMINANT 20; MRD20 | HP:0000582, HP:0005280, HP:0000316 |
| 22 | OMIM:613451 | FRONTONASAL DYSPLASIA 2; FND2 | HP:0000582, HP:0005280, HP:0000316 |
| 22 | OMIM:613563 | NOONAN SYNDROME-LIKE DISORDER WITH OR WITHOUT JUVENILE MYELOMONOCYTIC LEUKEMIA; NSLL | HP:0002007, HP:0005280, HP:0000316 |
| 22 | OMIM:614080 | MULTIPLE CONGENITAL ANOMALIES-HYPOTONIA-SEIZURES SYNDROME 1; MCAHS1 | HP:0002007, HP:0005280, HP:0000316 |
| 22 | OMIM:614105 | METHYLMALONATE SEMIALDEHYDE DEHYDROGENASE DEFICIENCY; MMSDHD | HP:0002007, HP:0005280, HP:0000316 |
| 22 | OMIM:614862 | PEROXISOME BIOGENESIS DISORDER 4A (ZELLWEGER); PBD4A | HP:0000582, HP:0005280, HP:0000316 |
| 22 | OMIM:614976 | CARPENTER SYNDROME 2; CRPT2 | HP:0000582, HP:0005280, HP:0000316 |
| 22 | OMIM:615829 | XIA-GIBBS SYNDROME | HP:0000582, HP:0005280, HP:0000316 |
| 22 | OMIM:616638 | SMITH-KINGSMORE SYNDROME; SKS | HP:0002007, HP:0005280, HP:0000316 |
| 22 | OMIM:617011 | MACROCEPHALY, DYSMORPHIC FACIES, AND PSYCHOMOTOR RETARDATION; MDFPMR | HP:0002007, HP:0000582, HP:0000316 |
| 22 | OMIM:617752 | MENTAL RETARDATION, AUTOSOMAL DOMINANT 49; MRD49 | HP:0000582, HP:0005280, HP:0000316 |
| 22 | OMIM:617757 | JOUBERT SYNDROME 32; JBTS32 | HP:0002007, HP:0005280, HP:0000316 |
| 22 | OMIM:617822 | ALKURAYA-KUCINSKAS SYNDROME; ALKKUCS | HP:0000582, HP:0005280, HP:0000316 |
| 22 | OMIM:617925 | SHORT-RIB THORACIC DYSPLASIA 20 WITH POLYDACTYLY; SRTD20 | HP:0002007, HP:0005280, HP:0000316 |
| 22 | OMIM:618142 | MICROCEPHALY, FACIAL DYSMORPHISM, RENAL AGENESIS, AND AMBIGUOUS GENITALIA SYNDROME; MFRG | HP:0000582, HP:0005280, HP:0000316 |
| 22 | OMIM:618272 | GLOBAL DEVELOPMENTAL DELAY, LUNG CYSTS, OVERGROWTH, AND WILMS TUMOR; GLOW | HP:0002007, HP:0005280, HP:0000316 |

---


---


---

# Cluster 24

| Cluster | Term | Name |
| --- | --- | --- |
| 24 | HP:0000490 | Deeply set eye |
| 24 | HP:0002007 | Frontal bossing |
| 24 | HP:0000341 | Narrow forehead |
| 24 | HP:0002714 | Downturned corners of mouth |

| Cluster | Term | Name | HPOs\_in\_clusters |
| --- | --- | --- | --- |
| 24 | OMIM:264090 | WIEDEMANN-RAUTENSTRAUCH SYNDROME; WDRTS | HP:0002007, HP:0000490, HP:0002714 |
| 24 | OMIM:269880 | SHORT SYNDROME | HP:0002007, HP:0000490, HP:0002714 |

---


---


---

# Cluster 2

| Cluster | Term | Name |
| --- | --- | --- |
| 2 | HP:0000478 | Abnormality of the eye |
| 2 | HP:0000769 | Abnormality of the breast |
| 2 | HP:0001507 | Growth abnormality |

---


---


---

# Cluster 14

| Cluster | Term | Name |
| --- | --- | --- |
| 14 | HP:0001263 | Global developmental delay |
| 14 | HP:0007018 | Attention deficit hyperactivity disorder |
| 14 | HP:0007360 | Aplasia/Hypoplasia of the cerebellum |
| 14 | HP:0008935 | Generalized neonatal hypotonia |
| 14 | HP:0009062 | Infantile axial hypotonia |
| 14 | HP:0011994 | Abnormal atrial septum morphology |

---


---


---

# Cluster 17

| Cluster | Term | Name |
| --- | --- | --- |
| 17 | HP:0000218 | High palate |
| 17 | HP:0000581 | Blepharophimosis |
| 17 | HP:0000290 | Abnormality of the forehead |
| 17 | HP:0000537 | Epicanthus inversus |
| 17 | HP:0001369 | Arthritis |
| 17 | HP:0002208 | Coarse hair |
| 17 | HP:0010301 | Spinal dysraphism |

---


---


---

# Cluster 18

| Cluster | Term | Name |
| --- | --- | --- |
| 18 | HP:0000358 | Posteriorly rotated ears |
| 18 | HP:0001249 | Intellectual disability |
| 18 | HP:0009885 | obsolete Prenatal short stature |
| 18 | HP:0001392 | Abnormality of the liver |
| 18 | HP:0002164 | Nail dysplasia |
| 18 | HP:0010722 | Asymmetry of the ears |

---


---


---

# Cluster 19

| Cluster | Term | Name |
| --- | --- | --- |
| 19 | HP:0000243 | Trigonocephaly |
| 19 | HP:0000431 | Wide nasal bridge |
| 19 | HP:0000752 | Hyperactivity |
| 19 | HP:0000684 | Delayed eruption of teeth |
| 19 | HP:0000689 | Dental malocclusion |

---


---


---

# Cluster 28

| Cluster | Term | Name |
| --- | --- | --- |
| 28 | HP:0000286 | Epicanthus |
| 28 | HP:0001540 | Diastasis recti |
| 28 | HP:0000951 | Abnormality of the skin |
| 28 | HP:0001643 | Patent ductus arteriosus |
| 28 | HP:0005326 | Hypoplastic philtrum |
