# Supplementary material for: Phenotype-genotype comorbidity analysis of patients with rare disorders provides insight into their pathological and molecular bases
Source: PLoS Genet. 2020 Oct 1;16(10):e1009054. doi: 10.1371/journal.pgen.1009054 (PMC7553355; doi:10.1371/journal.pgen.1009054)
Supplement: S5 Report — General details for each of the clusters, as well as details of OMIM diseases and KEGG pathways. Although not shown here due to patient-confidentiality, this report can also include tables of patients assigned to each cluster, including details of their phenotypes and genes that overlap with the phenotypes in the clusters, allowing the interested user to generate such information for their own patient cohort. (HTML) [file pgen.1009054.s005.html]

cluster\_details\_kegg\_template.utf8.md


# Report 4: Cluster Details for KEGG coherent clusters

## kegg clusters

---


---


---

# Cluster 2

| Cluster | Term | Name |
| --- | --- | --- |
| 2 | HP:0000377 | Abnormality of the pinna |
| 2 | HP:0001792 | Small nail |
| 2 | HP:0000430 | Underdeveloped nasal alae |

| Cluster | Term | Name | Genes | Percentage\_of\_nodes\_with\_funsys |
| --- | --- | --- | --- | --- |
| 2 | hsa04512 | ECM-receptor interaction | COL1A2, HSPG2, LAMC1, LAMB1, COL3A1, ITGA7, ITGA8, COMP, ITGA5, ITGA6, TNR, ITGB7, ITGB1, RELN, LAMB3, LAMC2, ITGAV, GP1BB, LAMB4, COL5A2, TNN | 100 |

| Cluster | Term | Name | HPOs\_in\_clusters |
| --- | --- | --- | --- |
| 2 | OMIM:129400 | RAPP-HODGKIN SYNDROME; RHS | HP:0001792, HP:0000430 |
| 2 | OMIM:209885 | BARBER-SAY SYNDROME; BBRSAY | HP:0000377, HP:0000430 |
| 2 | OMIM:219000 | FRASER SYNDROME 1; FRASRS1 | HP:0000377, HP:0000430 |
| 2 | OMIM:614607 | COFFIN-SIRIS SYNDROME 2; CSS2 | HP:0001792, HP:0000377 |

---


---


---

# Cluster 4

| Cluster | Term | Name |
| --- | --- | --- |
| 4 | HP:0000369 | Low-set ears |
| 4 | HP:0001518 | Small for gestational age |
| 4 | HP:0001631 | Atrial septal defect |

| Cluster | Term | Name | Genes | Percentage\_of\_nodes\_with\_funsys |
| --- | --- | --- | --- | --- |
| 4 | hsa00980 | Metabolism of xenobiotics by cytochrome P450 | UGT1A1, GSTM3, UGT1A3, GSTT1, ADH7, AKR1C1, UGT2A3, GSTM1, AKR1C4, UGT2B28, UGT2A2, UGT1A8, AKR1C2, ADH1A, UGT2B10, CYP3A4, CYP1A2, AKR1C3, EPHX1, GSTK1, GSTT2, GSTM4, CYP3A5, UGT1A7, ALDH3A1, GSTM2, UGT1A5, UGT2B7, CYP3A7, ADH4, UGT2B11, CYP3A43, UGT1A10, ADH1C, ADH5, UGT1A4, UGT2B15, UGT2B4, CYP1A1, UGT2A1, CYP2E1, ALDH1A3, UGT1A9, ADH1B, GSTM5, MGST2, UGT1A6, DHDH, ADH6 | 100 |

| Cluster | Term | Name | HPOs\_in\_clusters |
| --- | --- | --- | --- |
| 4 | OMIM:115150 | CARDIOFACIOCUTANEOUS SYNDROME 1; CFC1 | HP:0000369, HP:0001631 |
| 4 | OMIM:117650 | CEREBROCOSTOMANDIBULAR SYNDROME; CCMS | HP:0000369, HP:0001631 |
| 4 | OMIM:135900 | COFFIN-SIRIS SYNDROME 1; CSS1 | HP:0000369, HP:0001631 |
| 4 | OMIM:180849 | RUBINSTEIN-TAYBI SYNDROME 1; RSTS1 | HP:0000369, HP:0001631 |
| 4 | OMIM:201000 | CARPENTER SYNDROME 1; CRPT1 | HP:0000369, HP:0001631 |
| 4 | OMIM:208085 | ARTHROGRYPOSIS, RENAL DYSFUNCTION, AND CHOLESTASIS 1; ARCS1 | HP:0000369, HP:0001631 |
| 4 | OMIM:210710 | MICROCEPHALIC OSTEODYSPLASTIC PRIMORDIAL DWARFISM, TYPE I; MOPD1 | HP:0000369, HP:0001631 |
| 4 | OMIM:213980 | CRANIOFACIAL DYSMORPHISM, SKELETAL ANOMALIES, AND MENTAL RETARDATION SYNDROME; CFSMR | HP:0000369, HP:0001631 |
| 4 | OMIM:218040 | COSTELLO SYNDROME; CSTLO | HP:0000369, HP:0001631 |
| 4 | OMIM:220210 | RITSCHER-SCHINZEL SYNDROME 1; RTSC1 | HP:0000369, HP:0001631 |
| 4 | OMIM:222470 | TRICHOHEPATOENTERIC SYNDROME 1; THES1 | HP:0000369, HP:0001518 |
| 4 | OMIM:224690 | MEIER-GORLIN SYNDROME 1; MGORS1 | HP:0000369, HP:0001518 |
| 4 | OMIM:235510 | HENNEKAM LYMPHANGIECTASIA-LYMPHEDEMA SYNDROME 1; HKLLS1 | HP:0000369, HP:0001631 |
| 4 | OMIM:243800 | JOHANSON-BLIZZARD SYNDROME; JBS | HP:0001518, HP:0001631 |
| 4 | OMIM:251300 | GALLOWAY-MOWAT SYNDROME 1; GAMOS1 | HP:0000369, HP:0001518 |
| 4 | OMIM:257300 | MOSAIC VARIEGATED ANEUPLOIDY SYNDROME 1; MVA1 | HP:0000369, HP:0001518 |
| 4 | OMIM:261540 | PETERS-PLUS SYNDROME; PTRPLS | HP:0000369, HP:0001631 |
| 4 | OMIM:268300 | ROBERTS SYNDROME; RBS | HP:0000369, HP:0001631 |
| 4 | OMIM:269150 | SCHINZEL-GIEDION MIDFACE RETRACTION SYNDROME | HP:0000369, HP:0001631 |
| 4 | OMIM:270400 | SMITH-LEMLI-OPITZ SYNDROME; SLOS | HP:0000369, HP:0001631 |
| 4 | OMIM:275210 | RESTRICTIVE DERMOPATHY, LETHAL | HP:0000369, HP:0001631 |
| 4 | OMIM:300373 | OSTEOPATHIA STRIATA WITH CRANIAL SCLEROSIS; OSCS | HP:0000369, HP:0001631 |
| 4 | OMIM:300855 | OGDEN SYNDROME; OGDNS | HP:0000369, HP:0001631 |
| 4 | OMIM:300968 | MENTAL RETARDATION, X-LINKED 99, SYNDROMIC, FEMALE-RESTRICTED; MRXS99F | HP:0000369, HP:0001631 |
| 4 | OMIM:307030 | GLYCEROL KINASE DEFICIENCY; GKD | HP:0000369, HP:0001518 |
| 4 | OMIM:309520 | LUJAN-FRYNS SYNDROME | HP:0000369, HP:0001631 |
| 4 | OMIM:602535 | MARSHALL-SMITH SYNDROME; MRSHSS | HP:0000369, HP:0001631 |
| 4 | OMIM:605039 | BOHRING-OPITZ SYNDROME; BOPS | HP:0000369, HP:0001631 |
| 4 | OMIM:605822 | SPONDYLOOCULAR SYNDROME; SOS | HP:0000369, HP:0001631 |
| 4 | OMIM:606003 | TRANSALDOLASE DEFICIENCY | HP:0000369, HP:0001518 |
| 4 | OMIM:607721 | NOONAN SYNDROME-LIKE DISORDER WITH LOOSE ANAGEN HAIR 1; NSLH1 | HP:0000369, HP:0001631 |
| 4 | OMIM:608688 | AICAR TRANSFORMYLASE/IMP CYCLOHYDROLASE DEFICIENCY | HP:0000369, HP:0001631 |
| 4 | OMIM:609942 | NOONAN SYNDROME 3; NS3 | HP:0000369, HP:0001631 |
| 4 | OMIM:610498 | COMBINED OXIDATIVE PHOSPHORYLATION DEFICIENCY 2; COXPD2 | HP:0000369, HP:0001518 |
| 4 | OMIM:610536 | MANDIBULOFACIAL DYSOSTOSIS, GUION-ALMEIDA TYPE; MFDGA | HP:0000369, HP:0001631 |
| 4 | OMIM:613320 | SPONDYLOMETAPHYSEAL DYSPLASIA, MEGARBANE-DAGHER-MELKI TYPE; SMDMDM | HP:0000369, HP:0001518 |
| 4 | OMIM:614052 | MITOCHONDRIAL COMPLEX V (ATP SYNTHASE) DEFICIENCY, NUCLEAR TYPE 2; MC5DN2 | HP:0000369, HP:0001518 |
| 4 | OMIM:614080 | MULTIPLE CONGENITAL ANOMALIES-HYPOTONIA-SEIZURES SYNDROME 1; MCAHS1 | HP:0000369, HP:0001631 |
| 4 | OMIM:614114 | MOSAIC VARIEGATED ANEUPLOIDY SYNDROME 2; MVA2 | HP:0001518, HP:0001631 |
| 4 | OMIM:614261 | MICROCEPHALY-CAPILLARY MALFORMATION SYNDROME; MICCAP | HP:0000369, HP:0001518 |
| 4 | OMIM:615355 | NOONAN SYNDROME 8; NS8 | HP:0000369, HP:0001631 |
| 4 | OMIM:615834 | MENTAL RETARDATION, AUTOSOMAL DOMINANT 26; MRD26 | HP:0000369, HP:0001518 |
| 4 | OMIM:616268 | MENTAL RETARDATION, AUTOSOMAL DOMINANT 32; MRD32 | HP:0000369, HP:0001631 |
| 4 | OMIM:616546 | SHORT-RIB THORACIC DYSPLASIA 14 WITH POLYDACTYLY; SRTD14 | HP:0000369, HP:0001631 |
| 4 | OMIM:616777 | SECKEL SYNDROME 9; SCKL9 | HP:0001518, HP:0001631 |
| 4 | OMIM:616897 | OSTEOCHONDRODYSPLASIA, COMPLEX LETHAL, SYMOENS-BARNES-GISTELINCK TYPE; OCLSBG | HP:0000369, HP:0001518 |
| 4 | OMIM:617190 | SHASHI-PENA SYNDROME; SHAPNS | HP:0000369, HP:0001631 |
| 4 | OMIM:617360 | CONGENITAL HEART DEFECTS, DYSMORPHIC FACIAL FEATURES, AND INTELLECTUAL DEVELOPMENTAL DISORDER; CHDFIDD | HP:0000369, HP:0001631 |
| 4 | OMIM:617402 | CUTIS LAXA, AUTOSOMAL RECESSIVE, TYPE IIC; ARCL2C | HP:0000369, HP:0001631 |
| 4 | OMIM:617403 | CUTIS LAXA, AUTOSOMAL RECESSIVE, TYPE IID; ARCL2D | HP:0000369, HP:0001631 |
| 4 | OMIM:618142 | MICROCEPHALY, FACIAL DYSMORPHISM, RENAL AGENESIS, AND AMBIGUOUS GENITALIA SYNDROME; MFRG | HP:0000369, HP:0001631 |
| 4 | OMIM:618223 | VERTEBRAL ANOMALIES AND VARIABLE ENDOCRINE AND T-CELL DYSFUNCTION; VETD | HP:0000369, HP:0001631 |

---


---


---

# Cluster 5

| Cluster | Term | Name |
| --- | --- | --- |
| 5 | HP:0001518 | Small for gestational age |
| 5 | HP:0001839 | Split foot |
| 5 | HP:0001171 | Split hand |

| Cluster | Term | Name | Genes | Percentage\_of\_nodes\_with\_funsys |
| --- | --- | --- | --- | --- |
| 5 | hsa00591 | Linoleic acid metabolism | CYP3A4, CYP1A2, PLA2G3, CYP2E1, ALOX15, PLA2G2A, AKR1B10, PLA2G10, CYP3A7, PLA2G2F, PLA2G5, PLA2G4A, PLA2G2D, PLA2G2E, PLA2G2C, PLA2G6, CYP3A43, CYP3A5 | 100 |

| Cluster | Term | Name | HPOs\_in\_clusters |
| --- | --- | --- | --- |
| 5 | OMIM:103285 | ADULT SYNDROME | HP:0001171, HP:0001839 |
| 5 | OMIM:220600 | SPLIT-HAND/FOOT MALFORMATION 1 WITH SENSORINEURAL HEARING LOSS, AUTOSOMAL RECESSIVE; SHFM1D | HP:0001171, HP:0001839 |
| 5 | OMIM:225300 | SPLIT-HAND/FOOT MALFORMATION 6; SHFM6 | HP:0001171, HP:0001839 |
| 5 | OMIM:305600 | FOCAL DERMAL HYPOPLASIA; FDH | HP:0001171, HP:0001839 |
| 5 | OMIM:603543 | LIMB-MAMMARY SYNDROME; LMS | HP:0001171, HP:0001839 |
| 5 | OMIM:604292 | ECTRODACTYLY, ECTODERMAL DYSPLASIA, AND CLEFT LIP/PALATE SYNDROME 3; EEC3 | HP:0001171, HP:0001839 |
| 5 | OMIM:605289 | SPLIT-HAND/FOOT MALFORMATION 4; SHFM4 | HP:0001171, HP:0001839 |

---


---


---

# Cluster 12

| Cluster | Term | Name |
| --- | --- | --- |
| 12 | HP:0001642 | Pulmonic stenosis |
| 12 | HP:0001643 | Patent ductus arteriosus |
| 12 | HP:0001363 | Craniosynostosis |
| 12 | HP:0002087 | Abnormality of the upper respiratory tract |

| Cluster | Term | Name | Genes | Percentage\_of\_nodes\_with\_funsys |
| --- | --- | --- | --- | --- |
| 12 | hsa04810 | Regulation of actin cytoskeleton | PPP1CA, FGF4, MYLPF, FGFR3, CFL1, CRK, RAC2, MYL10, GNA12, ACTN3, FGF19, ACTN2, LIMK1, ARPC1B, ITGB8, PIK3CG, PDGFA, RAC1, ITGAL, MYL5, ITGAM, PDGFB, ACTB, ARPC1A, ITGAX, FGF3, FGF22, CRKL, SSH3, CHRM3 | 75 |
| 12 | hsa04666 | Fc gamma R-mediated phagocytosis | CRK, LIMK1, PIK3CG, LAT, RAC1, ARPC1A, ARPC1B, PLPP2, CRKL, WASF2, MAPK3, PLCG2, FCGR2A, FCGR3A, FCGR2C, FCGR2B, MARCKS, NCF1 | 100 |

| Cluster | Term | Name | HPOs\_in\_clusters |
| --- | --- | --- | --- |
| 12 | OMIM:261540 | PETERS-PLUS SYNDROME; PTRPLS | HP:0001642, HP:0001363, HP:0001643 |
| 12 | OMIM:617506 | NOONAN SYNDROME-LIKE DISORDER WITH LOOSE ANAGEN HAIR 2; NSLH2 | HP:0001642, HP:0001363, HP:0001643 |

---


---


---

# Cluster 20

| Cluster | Term | Name |
| --- | --- | --- |
| 20 | HP:0000486 | Strabismus |
| 20 | HP:0000343 | Long philtrum |
| 20 | HP:0000574 | Thick eyebrow |
| 20 | HP:0002007 | Frontal bossing |

| Cluster | Term | Name | Genes | Percentage\_of\_nodes\_with\_funsys |
| --- | --- | --- | --- | --- |
| 20 | hsa04742 | Taste transduction | GNAT3, GNAS, TAS2R42, TAS1R2, TAS2R9, PRKACB, TAS2R60, TAS2R38, TAS2R39, TAS2R40, TAS2R41, TAS1R3, TAS2R4, TAS1R1, PRKX, TAS2R16, TAS2R1, TAS2R14, TAS2R19, TAS2R43, TAS2R10, TAS2R8, TAS2R3, TAS2R5, TAS2R20, GNG3, TAS2R46, PRKACG, GNG13, GNB1, TAS2R50, TAS2R13, CACNA1B, CACNA1A, PDE1A, TAS2R7, TAS2R31 | 100 |

| Cluster | Term | Name | HPOs\_in\_clusters |
| --- | --- | --- | --- |
| 20 | OMIM:219200 | CUTIS LAXA, AUTOSOMAL RECESSIVE, TYPE IIA; ARCL2A | HP:0002007, HP:0000486, HP:0000343 |
| 20 | OMIM:261515 | D-BIFUNCTIONAL PROTEIN DEFICIENCY | HP:0002007, HP:0000486, HP:0000343 |
| 20 | OMIM:273750 | THREE M SYNDROME 1; 3M1 | HP:0002007, HP:0000574, HP:0000343 |
| 20 | OMIM:305450 | OPITZ-KAVEGGIA SYNDROME; OKS | HP:0002007, HP:0000486, HP:0000343 |
| 20 | OMIM:605130 | WIEDEMANN-STEINER SYNDROME; WDSTS | HP:0000574, HP:0000486, HP:0000343 |
| 20 | OMIM:606232 | PHELAN-MCDERMID SYNDROME; PHMDS | HP:0000574, HP:0000486, HP:0000343 |
| 20 | OMIM:616638 | SMITH-KINGSMORE SYNDROME; SKS | HP:0002007, HP:0000486, HP:0000343 |
| 20 | OMIM:617157 | SHORT STATURE, BRACHYDACTYLY, INTELLECTUAL DEVELOPMENTAL DISABILITY, AND SEIZURES; SBIDDS | HP:0002007, HP:0000486, HP:0000343 |
| 20 | OMIM:617991 | DEVELOPMENTAL DELAY, INTELLECTUAL DISABILITY, OBESITY, AND DYSMORPHISM; DIDOD | HP:0000574, HP:0000486, HP:0000343 |

---


---


---

# Cluster 25

| Cluster | Term | Name |
| --- | --- | --- |
| 25 | HP:0000286 | Epicanthus |
| 25 | HP:0000582 | Upslanted palpebral fissure |
| 25 | HP:0001182 | Tapered finger |

| Cluster | Term | Name | Genes | Percentage\_of\_nodes\_with\_funsys |
| --- | --- | --- | --- | --- |
| 25 | hsa04664 | Fc epsilon RI signaling pathway | IL5, PRKCA, IL3, IL13, AKT1, RAC2, PLCG2, VAV2, PDK1, INPP5D, MAPK3, AKT3, PLA2G2A, CSF2, PLA2G5, PLA2G4A, MAPK11, MAPK10, LAT, MAPK1, FYN, PLA2G10, PIK3CG, RAC1, BTK, PRKCE, PIK3R5, RAF1, MAP2K2, PLA2G2F, IL4, MAPK12, PIK3CD, PLA2G2D, PIK3CB, PLA2G2E, PLA2G2C | 100 |

| Cluster | Term | Name | HPOs\_in\_clusters |
| --- | --- | --- | --- |
| 25 | OMIM:309580 | MENTAL RETARDATION-HYPOTONIC FACIES SYNDROME, X-LINKED, 1; MRXHF1 | HP:0000582, HP:0000286, HP:0001182 |
| 25 | OMIM:617159 | SIFRIM-HITZ-WEISS SYNDROME; SIHIWES | HP:0000582, HP:0000286, HP:0001182 |
| 25 | OMIM:617991 | DEVELOPMENTAL DELAY, INTELLECTUAL DISABILITY, OBESITY, AND DYSMORPHISM; DIDOD | HP:0000582, HP:0000286, HP:0001182 |
| 25 | OMIM:162100 | AMYOTROPHY, HEREDITARY NEURALGIC; HNA | HP:0000582, HP:0000286 |
| 25 | OMIM:164280 | FEINGOLD SYNDROME 1; FGLDS1 | HP:0000582, HP:0000286 |
| 25 | OMIM:190685 | DOWN SYNDROME | HP:0000582, HP:0000286 |
| 25 | OMIM:211750 | C SYNDROME | HP:0000582, HP:0000286 |
| 25 | OMIM:213980 | CRANIOFACIAL DYSMORPHISM, SKELETAL ANOMALIES, AND MENTAL RETARDATION SYNDROME; CFSMR | HP:0000582, HP:0000286 |
| 25 | OMIM:214100 | PEROXISOME BIOGENESIS DISORDER 1A (ZELLWEGER); PBD1A | HP:0000582, HP:0000286 |
| 25 | OMIM:214110 | PEROXISOME BIOGENESIS DISORDER 2A (ZELLWEGER); PBD2A | HP:0000582, HP:0000286 |
| 25 | OMIM:216340 | YUNIS-VARON SYNDROME; YVS | HP:0000582, HP:0001182 |
| 25 | OMIM:239300 | HYPERPHOSPHATASIA WITH MENTAL RETARDATION SYNDROME 1; HPMRS1 | HP:0000582, HP:0001182 |
| 25 | OMIM:254940 | CAREY-FINEMAN-ZITER SYNDROME; CFZS | HP:0000286, HP:0001182 |
| 25 | OMIM:257300 | MOSAIC VARIEGATED ANEUPLOIDY SYNDROME 1; MVA1 | HP:0000582, HP:0000286 |
| 25 | OMIM:260565 | PEHO SYNDROME; PEHO | HP:0000286, HP:0001182 |
| 25 | OMIM:261515 | D-BIFUNCTIONAL PROTEIN DEFICIENCY | HP:0000582, HP:0000286 |
| 25 | OMIM:300209 | SIMPSON-GOLABI-BEHMEL SYNDROME, TYPE 2; SGBS2 | HP:0000286, HP:0001182 |
| 25 | OMIM:300831 | CK SYNDROME | HP:0000582, HP:0000286 |
| 25 | OMIM:300998 | MENTAL RETARDATION, X-LINKED, SYNDROMIC, 35; MRXS35 | HP:0000286, HP:0001182 |
| 25 | OMIM:301040 | ALPHA-THALASSEMIA/MENTAL RETARDATION SYNDROME, X-LINKED; ATRX | HP:0000286, HP:0001182 |
| 25 | OMIM:309500 | RENPENNING SYNDROME 1; RENS1 | HP:0000582, HP:0000286 |
| 25 | OMIM:605039 | BOHRING-OPITZ SYNDROME; BOPS | HP:0000582, HP:0001182 |
| 25 | OMIM:610443 | KOOLEN-DE VRIES SYNDROME; KDVS | HP:0000582, HP:0000286 |
| 25 | OMIM:610536 | MANDIBULOFACIAL DYSOSTOSIS, GUION-ALMEIDA TYPE; MFDGA | HP:0000582, HP:0000286 |
| 25 | OMIM:610954 | PITT-HOPKINS SYNDROME; PTHS | HP:0000582, HP:0001182 |
| 25 | OMIM:613406 | WITTEVEEN-KOLK SYNDROME; WITKOS | HP:0000582, HP:0000286 |
| 25 | OMIM:613610 | CRANIOECTODERMAL DYSPLASIA 2; CED2 | HP:0000582, HP:0000286 |
| 25 | OMIM:614976 | CARPENTER SYNDROME 2; CRPT2 | HP:0000582, HP:0000286 |
| 25 | OMIM:615286 | MENTAL RETARDATION, AUTOSOMAL RECESSIVE 36; MRT36 | HP:0000582, HP:0000286 |
| 25 | OMIM:615824 | MITOCHONDRIAL COMPLEX III DEFICIENCY, NUCLEAR TYPE 7; MC3DN7 | HP:0000582, HP:0000286 |
| 25 | OMIM:616351 | MENTAL RETARDATION, AUTOSOMAL DOMINANT 34; MRD34 | HP:0000582, HP:0000286 |
| 25 | OMIM:616579 | MENTAL RETARDATION, AUTOSOMAL DOMINANT 40; MRD40 | HP:0000582, HP:0000286 |
| 25 | OMIM:616734 | SKIN CREASES, CONGENITAL SYMMETRIC CIRCUMFERENTIAL, 2; CSCSC2 | HP:0000582, HP:0000286 |
| 25 | OMIM:616737 | TAKENOUCHI-KOSAKI SYNDROME; TKS | HP:0000582, HP:0001182 |
| 25 | OMIM:616788 | OROFACIAL CLEFT 15; OFC15 | HP:0000582, HP:0000286 |
| 25 | OMIM:616801 | HYPOTONIA, INFANTILE, WITH PSYCHOMOTOR RETARDATION AND CHARACTERISTIC FACIES 2; IHPRF2 | HP:0000286, HP:0001182 |
| 25 | OMIM:617061 | MENTAL RETARDATION, AUTOSOMAL DOMINANT 44; MRD44 | HP:0000582, HP:0001182 |
| 25 | OMIM:617260 | GLOBAL DEVELOPMENTAL DELAY, ABSENT OR HYPOPLASTIC CORPUS CALLOSUM, AND DYSMORPHIC FACIES; GDACCF | HP:0000582, HP:0000286 |
| 25 | OMIM:617360 | CONGENITAL HEART DEFECTS, DYSMORPHIC FACIAL FEATURES, AND INTELLECTUAL DEVELOPMENTAL DISORDER; CHDFIDD | HP:0000582, HP:0000286 |
| 25 | OMIM:617752 | MENTAL RETARDATION, AUTOSOMAL DOMINANT 49; MRD49 | HP:0000582, HP:0000286 |
| 25 | OMIM:617755 | NEURODEVELOPMENTAL DISORDER WITH DYSMORPHIC FACIES AND DISTAL LIMB ANOMALIES; NEDDFL | HP:0000582, HP:0000286 |
| 25 | OMIM:617807 | NEURODEVELOPMENTAL DISORDER WITH ATAXIC GAIT, ABSENT SPEECH, AND DECREASED CORTICAL WHITE MATTER; NDAGSCW | HP:0000582, HP:0001182 |
| 25 | OMIM:617883 | FANCONI ANEMIA, COMPLEMENTATION GROUP S; FANCS | HP:0000582, HP:0000286 |
| 25 | OMIM:618050 | MENTAL RETARDATION, AUTOSOMAL DOMINANT 57; MRD57 | HP:0000582, HP:0000286 |
| 25 | OMIM:618292 | NEURODEVELOPMENTAL DISORDER WITH IMPAIRED INTELLECTUAL DEVELOPMENT, HYPOTONIA, AND ATAXIA; NEDIDHA | HP:0000286, HP:0001182 |

---


---


---

# Cluster 29

| Cluster | Term | Name |
| --- | --- | --- |
| 29 | HP:0000431 | Wide nasal bridge |
| 29 | HP:0000455 | Broad nasal tip |
| 29 | HP:0000506 | Telecanthus |
| 29 | HP:0002714 | Downturned corners of mouth |

| Cluster | Term | Name | Genes | Percentage\_of\_nodes\_with\_funsys |
| --- | --- | --- | --- | --- |
| 29 | hsa04930 | Type II diabetes mellitus | SLC2A2, PRKCZ, CACNA1E, HK3, IRS2, MTOR, MAPK3, PIK3CA, MAPK10, GCK, MAPK1, MAFA, PRKCE, SOCS1, PIK3R5, HK2, ADIPOQ, SLC2A4, PIK3CD, CACNA1B, PIK3CB, CACNA1A | 75 |
| 29 | hsa04973 | Carbohydrate digestion and absorption | AMY2B, SLC2A2, PIK3R5, AMY2A, AKT3, AMY1B, SLC2A5, TAS1R3, ATP1B3, TAS1R2, G6PC2, ATP1B4, AMY1A, PIK3CA, AMY1C, PIK3CD, AKT1, PIK3CB, SI, ATP1B2 | 100 |

| Cluster | Term | Name | HPOs\_in\_clusters |
| --- | --- | --- | --- |
| 29 | OMIM:269880 | SHORT SYNDROME | HP:0000431, HP:0002714, HP:0000506 |
| 29 | OMIM:603671 | ACROMELIC FRONTONASAL DYSOSTOSIS; AFND | HP:0000431, HP:0000455, HP:0000506 |

---


---


---

# Cluster 6

| Cluster | Term | Name |
| --- | --- | --- |
| 6 | HP:0000286 | Epicanthus |
| 6 | HP:0000189 | Narrow palate |
| 6 | HP:0000272 | Malar flattening |
| 6 | HP:0001852 | Sandal gap |

| Cluster | Term | Name | Genes | Percentage\_of\_nodes\_with\_funsys |
| --- | --- | --- | --- | --- |
| 6 | hsa04730 | Long-term depression | BRAF, GNAS, PRKCA, GRIA2, GNA12, IGF1R, ITPR2, LYN, MAPK3, PLA2G2A, PPP1R17, PLA2G5, PLA2G4A, GNAI3, GNAQ, PRKCG, GUCY1A1, MAPK1, PLA2G10, PPP2CA, CRHR1, GRID2, PLCB3, RAF1, GUCY1B1, PLA2G2F, PLA2G2D, PLA2G2E, PRKG2, PLA2G2C, PLA2G6 | 75 |

| Cluster | Term | Name | HPOs\_in\_clusters |
| --- | --- | --- | --- |
| 6 | OMIM:235510 | HENNEKAM LYMPHANGIECTASIA-LYMPHEDEMA SYNDROME 1; HKLLS1 | HP:0000286, HP:0000272, HP:0000189 |

---


---


---

# Cluster 28

| Cluster | Term | Name |
| --- | --- | --- |
| 28 | HP:0000343 | Long philtrum |
| 28 | HP:0003196 | Short nose |
| 28 | HP:0000574 | Thick eyebrow |
| 28 | HP:0002597 | Abnormality of the vasculature |

| Cluster | Term | Name | Genes | Percentage\_of\_nodes\_with\_funsys |
| --- | --- | --- | --- | --- |
| 28 | hsa04742 | Taste transduction | GNAT3, GNAS, TAS2R42, TAS1R2, TAS2R9, PRKACB, TAS2R60, TAS2R38, TAS2R39, TAS2R40, TAS2R41, TAS1R3, TAS2R4, TAS1R1, PRKX, TAS2R16, TAS2R1, TAS2R14, TAS2R19, TAS2R43, TAS2R10, TAS2R8, TAS2R3, TAS2R5, TAS2R20, GNG3, TAS2R46, PRKACG, GNG13, GNB1, TAS2R50, TAS2R13, CACNA1B, CACNA1A, PDE1A, TAS2R7, TAS2R31 | 75 |

| Cluster | Term | Name | HPOs\_in\_clusters |
| --- | --- | --- | --- |
| 28 | OMIM:617991 | DEVELOPMENTAL DELAY, INTELLECTUAL DISABILITY, OBESITY, AND DYSMORPHISM; DIDOD | HP:0003196, HP:0000574, HP:0000343 |

---


---


---

# Cluster 33

| Cluster | Term | Name |
| --- | --- | --- |
| 33 | HP:0000494 | Downslanted palpebral fissures |
| 33 | HP:0004209 | Clinodactyly of the 5th finger |
| 33 | HP:0000347 | Micrognathia |
| 33 | HP:0000581 | Blepharophimosis |

| Cluster | Term | Name | Genes | Percentage\_of\_nodes\_with\_funsys |
| --- | --- | --- | --- | --- |
| 33 | hsa04630 | Jak-STAT signaling pathway | IL5, IL15RA, IFNA2, IFNA8, IFNB1, TPO, IL12A, IFNA4, IL5RA, IL3, IL13, AKT1, IL7, IFNA6, IFNA16, IFNA7, STAM, CSF2RA, IFNW1, IL3RA, SPRY3, EPO, AKT3, IL4R, SPRY1, CSF2, IFNA13, IL21, BCL2L1, OSMR, IL21R, IL6, IFNA21, CREBBP, IFNLR1, JAK2, CTF1, IFNK, IL2, SPRY4, IFNE, IFNA10, IL15, IL9, SOCS1, IFNA1, IL19, IFNA17, IFNA5, GHR, IL10, IL20, IL24, IL20RB, CRLF2, IFNA14, IL2RA, IL4, IL22RA1, PIK3CD, PIK3CB, LIFR, LEP | 75 |
| 33 | hsa00310 | Lysine degradation | ALDH3A2, TMLHE, SUV39H2, COLGALT2, ALDH1B1, OGDH, NSD1, EHMT1, PLOD3, SETD7, SETDB2, ALDH7A1, NSD2 | 75 |

| Cluster | Term | Name | HPOs\_in\_clusters |
| --- | --- | --- | --- |
| 33 | OMIM:210600 | SECKEL SYNDROME 1; SCKL1 | HP:0000347, HP:0000581, HP:0000494, HP:0004209 |
| 33 | OMIM:115150 | CARDIOFACIOCUTANEOUS SYNDROME 1; CFC1 | HP:0000347, HP:0004209, HP:0000494 |
| 33 | OMIM:180849 | RUBINSTEIN-TAYBI SYNDROME 1; RSTS1 | HP:0000347, HP:0004209, HP:0000494 |
| 33 | OMIM:208050 | ARTERIAL TORTUOSITY SYNDROME; ATORS | HP:0000347, HP:0000581, HP:0000494 |
| 33 | OMIM:224690 | MEIER-GORLIN SYNDROME 1; MGORS1 | HP:0000347, HP:0000581, HP:0004209 |
| 33 | OMIM:255995 | MYOPATHY, CONGENITAL, BAILEY-BLOCH; MYPBB | HP:0000347, HP:0000581, HP:0000494 |
| 33 | OMIM:260660 | COUSIN SYNDROME | HP:0000347, HP:0000581, HP:0004209 |
| 33 | OMIM:300990 | MIDFACE HYPOPLASIA, HEARING IMPAIRMENT, ELLIPTOCYTOSIS, AND NEPHROCALCINOSIS; MFHIEN | HP:0000347, HP:0004209, HP:0000494 |
| 33 | OMIM:605130 | WIEDEMANN-STEINER SYNDROME; WDSTS | HP:0000581, HP:0000494, HP:0004209 |
| 33 | OMIM:614222 | WARBURG MICRO SYNDROME 3; WARBM3 | HP:0000347, HP:0000581, HP:0004209 |
| 33 | OMIM:616975 | NEURODEVELOPMENTAL DISORDER WITH OR WITHOUT ANOMALIES OF THE BRAIN, EYE, OR HEART; NEDBEH | HP:0000347, HP:0000581, HP:0000494 |

---


---


---

# Cluster 1

| Cluster | Term | Name |
| --- | --- | --- |
| 1 | HP:0000772 | Abnormality of the ribs |
| 1 | HP:0002360 | Sleep disturbance |
| 1 | HP:0010628 | Facial palsy |

| Cluster | Term | Name | Genes | Percentage\_of\_nodes\_with\_funsys |
| --- | --- | --- | --- | --- |
| 1 | hsa00920 | Sulfur metabolism | SULT1A3, SULT1A1, SULT2B1, SULT1A4, CHST12, SULT1E1, SULT1A2 | 100 |

---


---


---

# Cluster 3

| Cluster | Term | Name |
| --- | --- | --- |
| 3 | HP:0004689 | Short fourth metatarsal |
| 3 | HP:0010012 | Abnormality of the 4th metacarpal |
| 3 | HP:0010013 | Abnormality of the 5th metacarpal |
| 3 | HP:0010042 | Aplasia/Hypoplasia of the 4th metacarpal |
| 3 | HP:0010045 | Aplasia/Hypoplasia of the 5th metacarpal |
| 3 | HP:0004686 | Short third metatarsal |

| Cluster | Term | Name | Genes | Percentage\_of\_nodes\_with\_funsys |
| --- | --- | --- | --- | --- |
| 3 | hsa05020 | Prion diseases | C6, C1QB, MAPK1, MAPK3, FYN, PRKACG, HSPA5, C9, C1QC, C8B, C7, NOTCH1, C8G, LAMC1, C1QA, C8A | 100 |

---


---


---

# Cluster 7

| Cluster | Term | Name |
| --- | --- | --- |
| 7 | HP:0002817 | Abnormality of the upper limb |
| 7 | HP:0001257 | Spasticity |
| 7 | HP:0002973 | Abnormality of the forearm |
| 7 | HP:0009811 | Abnormality of the elbow |

| Cluster | Term | Name | Genes | Percentage\_of\_nodes\_with\_funsys |
| --- | --- | --- | --- | --- |
| 7 | hsa04730 | Long-term depression | BRAF, GNAS, PRKCA, GRIA2, GNA12, IGF1R, ITPR2, LYN, MAPK3, PLA2G2A, PPP1R17, PLA2G5, PLA2G4A, GNAI3, GNAQ, PRKCG, GUCY1A1, MAPK1, PLA2G10, PPP2CA, CRHR1, GRID2, PLCB3, RAF1, GUCY1B1, PLA2G2F, PLA2G2D, PLA2G2E, PRKG2, PLA2G2C, PLA2G6 | 100 |

---


---


---

# Cluster 9

| Cluster | Term | Name |
| --- | --- | --- |
| 9 | HP:0000422 | Abnormality of the nasal bridge |
| 9 | HP:0000002 | Abnormality of body height |
| 9 | HP:0000204 | Cleft upper lip |
| 9 | HP:0002167 | Neurological speech impairment |

| Cluster | Term | Name | Genes | Percentage\_of\_nodes\_with\_funsys |
| --- | --- | --- | --- | --- |
| 9 | hsa04930 | Type II diabetes mellitus | SLC2A2, PRKCZ, CACNA1E, HK3, IRS2, MTOR, MAPK3, PIK3CA, MAPK10, GCK, MAPK1, MAFA, PRKCE, SOCS1, PIK3R5, HK2, ADIPOQ, SLC2A4, PIK3CD, CACNA1B, PIK3CB, CACNA1A | 100 |

---


---


---

# Cluster 10

| Cluster | Term | Name |
| --- | --- | --- |
| 10 | HP:0000581 | Blepharophimosis |
| 10 | HP:0000290 | Abnormality of the forehead |
| 10 | HP:0001369 | Arthritis |
| 10 | HP:0100807 | Long fingers |

| Cluster | Term | Name | Genes | Percentage\_of\_nodes\_with\_funsys |
| --- | --- | --- | --- | --- |
| 10 | hsa04150 | mTOR signaling pathway | TSC2, VEGFC, AKT1, MTOR, MAPK3, AKT3, RPS6KA2, MLST8, EIF4E2, RPS6KB2, CAB39L, RHEB, MAPK1, PDPK1, PRKAA1, PIK3R5, RPS6, RICTOR, ULK3, PIK3CD, PIK3CB, TSC1, RPS6KB1 | 100 |
| 10 | hsa05223 | Non-small cell lung cancer | CCND1, MAPK3, AKT3, PRKCA, CASP9, RB1, AKT1, E2F3, PIK3CD, E2F2, PDPK1, PIK3CB, RXRA, CDK6 | 75 |

---


---


---

# Cluster 11

| Cluster | Term | Name |
| --- | --- | --- |
| 11 | HP:0001321 | Cerebellar hypoplasia |
| 11 | HP:0005484 | Postnatal microcephaly |
| 11 | HP:0010864 | Intellectual disability, severe |
| 11 | HP:0011451 | Congenital microcephaly |
| 11 | HP:0100704 | Cerebral visual impairment |

| Cluster | Term | Name | Genes | Percentage\_of\_nodes\_with\_funsys |
| --- | --- | --- | --- | --- |
| 11 | hsa00982 | Drug metabolism - cytochrome P450 | UGT1A1, FMO4, GSTM3, UGT1A3, FMO3, ADH7, UGT2A3, GSTM1, UGT2B28, UGT2A2, UGT1A8, ADH1A, MGST3, UGT2B10, CYP1A2, CYP3A4, AOX1, GSTK1, GSTM4, CYP3A5, UGT1A7, ALDH3A1, GSTM2, UGT1A5, UGT2B7, CYP3A7, ADH4, UGT2B11, CYP3A43, UGT1A10, ADH1C, ADH5, UGT1A4, UGT2B15, UGT2B4, MGST2, FMO5, UGT2A1, FMO2, CYP2E1, MAOB, CYP2D6, MAOA, ALDH1A3, UGT1A9, ADH1B, GSTM5, FMO1, UGT1A6, ADH6 | 80 |
| 11 | hsa00380 | Tryptophan metabolism | AOC1, OGDH, KYNU, GCDH, ALDH7A1, DDC, CYP1A2, ACMSD, IDO2, ALDH1B1, IDO1, AOX1, ECHS1, ALDH3A2, KMO, EHHADH, CYP1A1, ASMT, MAOB, INMT, WARS, MAOA, HAAO, TPH2 | 100 |
| 11 | hsa00260 | Glycine, serine and threonine metabolism | MAOA, MAOB | 100 |
| 11 | hsa00340 | Histidine metabolism | ALDH3A1, ALDH3A2, MAOB, HNMT, MAOA, DDC, ALDH7A1 | 100 |
| 11 | hsa00330 | Arginine and proline metabolism | AMD1, AOC1, CPS1, ASS1, GLUL, LAP3, NOS1, GLS, ALDH7A1, ALDH1B1, PYCR3, CKB, OAT, ALDH3A2, ALDH4A1, SAT2, PRODH, SRM, MAOB, NOS3, PYCR2, MAOA, P4HA2, AGMAT | 100 |
| 11 | hsa00350 | Tyrosine metabolism | ADH1A, ADH1C, ALDH3A1, COMT, MAOB, MAOA, ADH4, FAH, BUD23, ADH7, ADH1B, ADH5, ADH6 | 100 |
| 11 | hsa00360 | Phenylalanine metabolism | ALDH3A1, MAOB, MAOA, PRDX6, ALDH1A3 | 100 |

---


---


---

# Cluster 14

| Cluster | Term | Name |
| --- | --- | --- |
| 14 | HP:0000377 | Abnormality of the pinna |
| 14 | HP:0009803 | Short phalanx of finger |
| 14 | HP:0001831 | Short toe |
| 14 | HP:0001792 | Small nail |

| Cluster | Term | Name | Genes | Percentage\_of\_nodes\_with\_funsys |
| --- | --- | --- | --- | --- |
| 14 | hsa04512 | ECM-receptor interaction | COL1A2, HSPG2, LAMC1, LAMB1, COL3A1, ITGA7, ITGA8, COMP, ITGA5, ITGA6, TNR, ITGB7, ITGB1, RELN, LAMB3, LAMC2, ITGAV, GP1BB, LAMB4, COL5A2, TNN | 100 |

---


---


---

# Cluster 21

| Cluster | Term | Name |
| --- | --- | --- |
| 21 | HP:0000219 | Thin upper lip vermilion |
| 21 | HP:0000400 | Macrotia |
| 21 | HP:0000411 | Protruding ear |
| 21 | HP:0005326 | Hypoplastic philtrum |
| 21 | HP:0010282 | Thin lower lip vermilion |

| Cluster | Term | Name | Genes | Percentage\_of\_nodes\_with\_funsys |
| --- | --- | --- | --- | --- |
| 21 | hsa04920 | Adipocytokine signaling pathway | G6PC2, CPT1B, IKBKG, AKT1, PRKAG2, PRKAB2, IRS2, MTOR, AKT3, TRAF2, MAPK10, TNFRSF1B, CPT1C, SLC2A1, JAK2, RXRA, PCK1, PRKAA1, ACSL6, ADIPOQ, ADIPOR2, SLC2A4, LEP | 100 |

---


---


---

# Cluster 24

| Cluster | Term | Name |
| --- | --- | --- |
| 24 | HP:0000252 | Microcephaly |
| 24 | HP:0001249 | Intellectual disability |
| 24 | HP:0000175 | Cleft palate |
| 24 | HP:0001238 | Slender finger |
| 24 | HP:0100490 | Camptodactyly of finger |

| Cluster | Term | Name | Genes | Percentage\_of\_nodes\_with\_funsys |
| --- | --- | --- | --- | --- |
| 24 | hsa00330 | Arginine and proline metabolism | AMD1, AOC1, CPS1, ASS1, GLUL, LAP3, NOS1, GLS, ALDH7A1, ALDH1B1, PYCR3, CKB, OAT, ALDH3A2, ALDH4A1, SAT2, PRODH, SRM, MAOB, NOS3, PYCR2, MAOA, P4HA2, AGMAT | 100 |

---


---


---

# Cluster 26

| Cluster | Term | Name |
| --- | --- | --- |
| 26 | HP:0000414 | Bulbous nose |
| 26 | HP:0000494 | Downslanted palpebral fissures |
| 26 | HP:0000218 | High palate |
| 26 | HP:0000377 | Abnormality of the pinna |
| 26 | HP:0000664 | Synophrys |
| 26 | HP:0010055 | Broad hallux |

| Cluster | Term | Name | Genes | Percentage\_of\_nodes\_with\_funsys |
| --- | --- | --- | --- | --- |
| 26 | hsa00310 | Lysine degradation | ALDH3A2, TMLHE, SUV39H2, COLGALT2, ALDH1B1, OGDH, NSD1, EHMT1, PLOD3, SETD7, SETDB2, ALDH7A1, NSD2 | 83.33333 |

---


---


---

# Cluster 8

| Cluster | Term | Name |
| --- | --- | --- |
| 8 | HP:0000582 | Upslanted palpebral fissure |
| 8 | HP:0000179 | Thick lower lip vermilion |
| 8 | HP:0000322 | Short philtrum |
| 8 | HP:0000455 | Broad nasal tip |

| Cluster | Term | Name | Genes | Percentage\_of\_nodes\_with\_funsys |
| --- | --- | --- | --- | --- |
| 8 | hsa04930 | Type II diabetes mellitus | SLC2A2, PRKCZ, CACNA1E, HK3, IRS2, MTOR, MAPK3, PIK3CA, MAPK10, GCK, MAPK1, MAFA, PRKCE, SOCS1, PIK3R5, HK2, ADIPOQ, SLC2A4, PIK3CD, CACNA1B, PIK3CB, CACNA1A | 75 |
| 8 | hsa05221 | Acute myeloid leukemia | MAPK1, RUNX1T1, PIK3CD, MTOR, PIK3R5, AKT3, RPS6KB1, MAPK3, RAF1 | 75 |

---


---


---

# Cluster 30

| Cluster | Term | Name |
| --- | --- | --- |
| 30 | HP:0000954 | Single transverse palmar crease |
| 30 | HP:0000960 | Sacral dimple |
| 30 | HP:0001172 | Abnormal thumb morphology |
| 30 | HP:0010781 | Skin dimples |

| Cluster | Term | Name | Genes | Percentage\_of\_nodes\_with\_funsys |
| --- | --- | --- | --- | --- |
| 30 | hsa04150 | mTOR signaling pathway | TSC2, VEGFC, AKT1, MTOR, MAPK3, AKT3, RPS6KA2, MLST8, EIF4E2, RPS6KB2, CAB39L, RHEB, MAPK1, PDPK1, PRKAA1, PIK3R5, RPS6, RICTOR, ULK3, PIK3CD, PIK3CB, TSC1, RPS6KB1 | 75 |
| 30 | hsa04530 | Tight junction | PRKCZ, MYL7, CLDN11, EPB41L1, AKT1, CLDN15, MYL10, LLGL1, PRKCI, ACTN2, MYH7B, YBX3, PPP2R1A, SRC, CLDN10, PRKCG, MYL9, AFDN, CLDN7, GNAI1, MYH14, MYL5, MAP3K20, PARD6B, RRAS, MPDZ | 75 |

---


---


---

# Cluster 19

| Cluster | Term | Name |
| --- | --- | --- |
| 19 | HP:0000478 | Abnormality of the eye |
| 19 | HP:0000598 | Abnormality of the ear |
| 19 | HP:0000661 | Palpebral fissure narrowing on adduction |
| 19 | HP:0002118 | Abnormality of the cerebral ventricles |
| 19 | HP:0002167 | Neurological speech impairment |
| 19 | HP:0002438 | Cerebellar malformation |
| 19 | HP:0003549 | Abnormality of connective tissue |

| Cluster | Term | Name | Genes | Percentage\_of\_nodes\_with\_funsys |
| --- | --- | --- | --- | --- |
| 19 | hsa05146 | Amoebiasis | PRKCA, IL12A, GNA14, COL4A2, SERPINB2, IL1R1, SERPINB9, SERPINB3, ACTN2, LAMA1, C9, SERPINB10, PIK3CA, GNAQ, IL6, LAMC1, SERPINB4, SERPINB13, COL4A1, VCL, IL1R2, SERPINB1, PLCB3, SERPINB6, GNAL, PRKACG, LAMB3, LAMC2, PIK3CD, ADCY1 | 71.42857 |

---


---


---

# Cluster 17

| Cluster | Term | Name |
| --- | --- | --- |
| 17 | HP:0000028 | Cryptorchidism |
| 17 | HP:0001371 | Flexion contracture |
| 17 | HP:0004322 | Short stature |
| 17 | HP:0005562 | Multiple renal cysts |

| Cluster | Term | Name | HPOs\_in\_clusters |
| --- | --- | --- | --- |
| 17 | OMIM:180849 | RUBINSTEIN-TAYBI SYNDROME 1; RSTS1 | HP:0004322, HP:0001371, HP:0000028 |
| 17 | OMIM:255995 | MYOPATHY, CONGENITAL, BAILEY-BLOCH; MYPBB | HP:0004322, HP:0001371, HP:0000028 |
| 17 | OMIM:602471 | SHORT STATURE, AUDITORY CANAL ATRESIA, MANDIBULAR HYPOPLASIA, AND SKELETAL ABNORMALITIES; SAMS | HP:0004322, HP:0001371, HP:0000028 |
| 17 | OMIM:615547 | SCHAAF-YANG SYNDROME; SHFYNG | HP:0004322, HP:0001371, HP:0000028 |

---


---


---

# Cluster 18

| Cluster | Term | Name |
| --- | --- | --- |
| 18 | HP:0000347 | Micrognathia |
| 18 | HP:0000494 | Downslanted palpebral fissures |
| 18 | HP:0000581 | Blepharophimosis |
| 18 | HP:0004209 | Clinodactyly of the 5th finger |
| 18 | HP:0001182 | Tapered finger |

| Cluster | Term | Name | HPOs\_in\_clusters |
| --- | --- | --- | --- |
| 18 | OMIM:210600 | SECKEL SYNDROME 1; SCKL1 | HP:0000347, HP:0000581, HP:0000494, HP:0004209 |

---


---


---

# Cluster 22

| Cluster | Term | Name |
| --- | --- | --- |
| 22 | HP:0000486 | Strabismus |
| 22 | HP:0000582 | Upslanted palpebral fissure |
| 22 | HP:0000347 | Micrognathia |
| 22 | HP:0000581 | Blepharophimosis |
| 22 | HP:0002007 | Frontal bossing |

| Cluster | Term | Name | HPOs\_in\_clusters |
| --- | --- | --- | --- |
| 22 | OMIM:224690 | MEIER-GORLIN SYNDROME 1; MGORS1 | HP:0000347, HP:0002007, HP:0000581, HP:0000486 |
| 22 | OMIM:261515 | D-BIFUNCTIONAL PROTEIN DEFICIENCY | HP:0000582, HP:0000347, HP:0002007, HP:0000486 |
| 22 | OMIM:613610 | CRANIOECTODERMAL DYSPLASIA 2; CED2 | HP:0000582, HP:0002007, HP:0000581, HP:0000347 |
| 22 | OMIM:616975 | NEURODEVELOPMENTAL DISORDER WITH OR WITHOUT ANOMALIES OF THE BRAIN, EYE, OR HEART; NEDBEH | HP:0000347, HP:0002007, HP:0000581, HP:0000486 |

---


---


---

# Cluster 31

| Cluster | Term | Name |
| --- | --- | --- |
| 31 | HP:0000463 | Anteverted nares |
| 31 | HP:0001182 | Tapered finger |
| 31 | HP:0004691 | 2-3 toe syndactyly |
| 31 | HP:0000324 | Facial asymmetry |

| Cluster | Term | Name | HPOs\_in\_clusters |
| --- | --- | --- | --- |
| 31 | OMIM:218000 | AGENESIS OF THE CORPUS CALLOSUM WITH PERIPHERAL NEUROPATHY; ACCPN | HP:0001182, HP:0004691, HP:0000324 |

---


---


---

# Cluster 13

| Cluster | Term | Name |
| --- | --- | --- |
| 13 | HP:0001770 | Toe syndactyly |
| 13 | HP:0002857 | Genu valgum |
| 13 | HP:0010554 | Cutaneous finger syndactyly |
| 13 | HP:0002118 | Abnormality of the cerebral ventricles |

---


---


---

# Cluster 15

| Cluster | Term | Name |
| --- | --- | --- |
| 15 | HP:0000286 | Epicanthus |
| 15 | HP:0001540 | Diastasis recti |
| 15 | HP:0003508 | Proportionate short stature |
| 15 | HP:0011304 | Broad thumb |
| 15 | HP:0000177 | Abnormality of upper lip |
| 15 | HP:0000243 | Trigonocephaly |
| 15 | HP:0000356 | Abnormality of the outer ear |
| 15 | HP:0000368 | Low-set, posteriorly rotated ears |
| 15 | HP:0000470 | Short neck |
| 15 | HP:0002230 | Generalized hirsutism |
| 15 | HP:0005326 | Hypoplastic philtrum |
| 15 | HP:0005469 | Flat occiput |
| 15 | HP:0006610 | Wide intermamillary distance |

---


---


---

# Cluster 16

| Cluster | Term | Name |
| --- | --- | --- |
| 16 | HP:0000286 | Epicanthus |
| 16 | HP:0000414 | Bulbous nose |
| 16 | HP:0000377 | Abnormality of the pinna |
| 16 | HP:0000494 | Downslanted palpebral fissures |
| 16 | HP:0000664 | Synophrys |

---


---


---

# Cluster 23

| Cluster | Term | Name |
| --- | --- | --- |
| 23 | HP:0000527 | Long eyelashes |
| 23 | HP:0000582 | Upslanted palpebral fissure |
| 23 | HP:0000154 | Wide mouth |
| 23 | HP:0000445 | Wide nose |
| 23 | HP:0001363 | Craniosynostosis |
| 23 | HP:0001533 | Slender build |
| 23 | HP:0001773 | Short foot |
| 23 | HP:0001956 | Truncal obesity |

---


---


---

# Cluster 27

| Cluster | Term | Name |
| --- | --- | --- |
| 27 | HP:0000232 | Everted lower lip vermilion |
| 27 | HP:0000341 | Narrow forehead |
| 27 | HP:0000545 | Myopia |
| 27 | HP:0001182 | Tapered finger |
| 27 | HP:0001513 | Obesity |
| 27 | HP:0001845 | Overlapping toe |
| 27 | HP:0002069 | Generalized tonic-clonic seizures |
| 27 | HP:0000448 | Prominent nose |
| 27 | HP:0001763 | Pes planus |
| 27 | HP:0009894 | Thickened ears |
| 27 | HP:0010282 | Thin lower lip vermilion |

---


---


---

# Cluster 32

| Cluster | Term | Name |
| --- | --- | --- |
| 32 | HP:0000316 | Hypertelorism |
| 32 | HP:0000463 | Anteverted nares |
| 32 | HP:0000582 | Upslanted palpebral fissure |
| 32 | HP:0000324 | Facial asymmetry |
| 32 | HP:0000951 | Abnormality of the skin |
| 32 | HP:0001852 | Sandal gap |
