# Supplementary material for: Phenotype-genotype comorbidity analysis of patients with rare disorders provides insight into their pathological and molecular bases
Source: PLoS Genet. 2020 Oct 1;16(10):e1009054. doi: 10.1371/journal.pgen.1009054 (PMC7553355; doi:10.1371/journal.pgen.1009054)
Supplement: S6 Report — Also contains details, for each patient, to which clusters their phenotypes map, and the genes that overlap functional systems enriched in these clusters. Due to patient confidentiality, we have not shown exact details of phenotypes and genes for each DECIPHER patient analysed here, however the interested reader who has signed the license agreement with DECIPHER could do so easily. We have included output generated for two patients from the DECIPHER database. (HTML) [file pgen.1009054.s006.html]

patient\_details\_template\_short.utf8


# Report 6: Patient Details

## Summary tables

|  |  |
| --- | --- |
| Type | more\_spec |
| Number of patients to clusters | 3021 |
| Number of patients overlap with functional coherent clusters | 1144 |

### Patients that overlap with clusters build with GO valid pairs

|  |  |
| --- | --- |
| Type | more\_spec |
| Number of patients to clusters | 2939 |
| Number of patients overlap with go functional coherent and OMIM clusters | 1008 |
| Number of patients overlap with go functional coherent clusters | 1024 |
| Number of patients orvelap with OMIM clusters | 2820 |

### Patients that overlap with clusters build with KEGG valid pairs

|  |  |
| --- | --- |
| Type | more\_spec |
| Number of patients to clusters | 2339 |
| Number of patients overlap with kegg functional coherent and OMIM clusters | 285 |
| Number of patients overlap with kegg functional coherent clusters | 367 |
| Number of patients orvelap with OMIM clusters | 1216 |

### Patients that overlap with clusters build with Reactome valid pairs

|  |  |
| --- | --- |
| Type | more\_spec |
| Number of patients to clusters | 2609 |
| Number of patients overlap with reactome functional coherent and OMIM clusters | 290 |
| Number of patients overlap with reactome functional coherent clusters | 298 |
| Number of patients orvelap with OMIM clusters | 1912 |

## Number of cluster vs number of HPO per patient

**Figure A**: ***Number of cluster related to the number of HPO per patient*** (*x-axis Number of HPO per patient. y-axis Number of cluster containing some HPO patient profile*). As we can see, the profile of the patients point to different clusters.

## Example of Patients Data

---


---


---

# Patient 254518

| Patient | Metric | Number\_HPO |
| --- | --- | --- |
| 254518 | Total number of HPOs | 25 |
| 254518 | Total HPOs overlapping clusters | 10 |
| 254518 | Number of genes in patient’s CNV | 30 |
| 254518 | Number of possible patogenic genes | 8 |

| Patient | Gene list from patient CNV |
| --- | --- |
| 254518 | HMGN1P25, MIR151B, MIR6764, RN7SKP92, RN7SL523P, RNU6-91P, RNU1-47P, RN7SL714P, CYP46A1, SLC25A29, DEGS2, EML1, C14orf177, SLC25A47, CCDC85C, RPL3P4, RPS2P3, VDAC3P1, MIR342, MIR345, EVL, BEGAIN, BCL11B, WARS, YY1, WDR25, SETD3, HHIPL1, CCNK, NDUFB3P4 |

| Patient | HPO | Name |
| --- | --- | --- |
| 254518 | HP:0000356 | Abnormality of the outer ear |
| 254518 | HP:0000581 | Blepharophimosis |
| 254518 | HP:0008872 | Feeding difficulties in infancy |
| 254518 | HP:0000316 | Hypertelorism |
| 254518 | HP:0001249 | Intellectual disability |
| 254518 | HP:0000369 | Low-set ears |
| 254518 | HP:0000347 | Micrognathia |
| 254518 | HP:0001252 | Muscular hypotonia |
| 254518 | HP:0003508 | Proportionate short stature |
| 254518 | HP:0000954 | Single transverse palmar crease |

| Patient | Cluster | Shared\_hpos | Systems | Genes |
| --- | --- | --- | --- | --- |
| 254518 | 32 | HP:0000347, HP:0000316, HP:0000581, HP:0000356 | GO:0006898 | HHIPL1 |
| 254518 | 46 | HP:0008872, HP:0000347, HP:0000316, HP:0000369 | GO:0015697 | SLC25A47, SLC25A29 |
| 254518 | 19 | HP:0001252, HP:0000316, HP:0000347 | GO:0006898 | HHIPL1 |
| 254518 | 15 | HP:0000581, HP:0000369 | GO:0001937 | MIR342 |
| 254518 | 16 | HP:0000316, HP:0000581 | GO:0006898 | HHIPL1 |
| 254518 | 18 | HP:0003508, HP:0001249 | GO:0021872 | BCL11B |
| 254518 | 88 | HP:0000954, HP:0000369 | GO:0010633 | EVL |
| 254518 | 88 | HP:0000954, HP:0000369 | GO:0002040 | MIR342 |
| 254518 | 31 | HP:0003508 | GO:0003002 | YY1 |
| 254518 | 73 | HP:0003508 | GO:1903671 | MIR342 |
| 254518 | 26 | HP:0000369 | R-HSA-8957322 | CYP46A1 |

---


---


---

# Patient 256892

| Patient | Metric | Number\_HPO |
| --- | --- | --- |
| 256892 | Total number of HPOs | 17 |
| 256892 | Total HPOs overlapping clusters | 9 |
| 256892 | Number of genes in patient’s CNV | 123 |
| 256892 | Number of possible patogenic genes | 21 |

| Patient | Gene list from patient CNV |
| --- | --- |
| 256892 | JAZF1-AS1, EEF1A1P27, HOXA-AS3, RPS2P30, RPL35P4, RPS26P30, RPL7AP41, MIR1183, SNRPCP19, HOTTIP, FCF1P1, PCMTD1P3, MIR3146, LOC100506098, LOC100506178, HOTAIRM1, HOXA10-HOXA9, RNA5SP227, RNA5SP228, MACC1-AS1, HOXA10-AS, EVX1-AS, LOC101927668, LOC101927769, LOC101927811, TPT1P7, LINC01162, GPNMB, TSEN15P3, LOC105375304, IGF2BP3, RNU7-143P, RN7SKP266, RN7SL542P, RNU6-979P, RNU6-1103P, SUMO2P14, NPM1P13, NUPL2, HIBADH, CBX3, MALSU1, CLK2P1, C7orf31, GSDME, EVX1, TWISTNB, SP8, EEF1A1P6, HOXA11-AS, JAZF1, FERD3L, RPL23P8, TMEM196, STEAP1B, RPS2P32, OSBPL3, RNU1-15P, C7orf71, HOXA-AS2, SNX10, TRA2A, HNRNPA2B1, HOXA1, HOXA2, HOXA3, HOXA4, HOXA5, HOXA6, HOXA7, HOXA9, HOXA10, HOXA11, HOXA13, ABCB5, ASS1P11, FAM221A, MACC1, IL6, ITGB8, RPS26P32, LOC389473, RPL12P10, LOC401312, EIF4HP1, LOC402641, TPM3P4, PPIAP80, MIR148A, LOC441204, NHP2P2, PSMC1P2, LOC442292, LOC442517, RPL23P7, RPL7AP38, HMGB3P20, MIR196B, NPY, MPP6, LOC541472, CYCS, TOMM7, CDCA7L, KLHL7, STK31, NPVF, SP4, SNORD93, RPL21P75, TWIST1, UBA52P1, TSL, FAM126A, DNAH11, TAX1BP1, SKAP2, CCDC126, CREB5, NFE2L3, HDAC9, RAPGEF5, KIAA0087 |

| Patient | HPO | Name |
| --- | --- | --- |
| 256892 | HP:0001156 | Brachydactyly |
| 256892 | HP:0001363 | Craniosynostosis |
| 256892 | HP:0000494 | Downslanted palpebral fissures |
| 256892 | HP:0000286 | Epicanthus |
| 256892 | HP:0000369 | Low-set ears |
| 256892 | HP:0008551 | Microtia |
| 256892 | HP:0003508 | Proportionate short stature |
| 256892 | HP:0000508 | Ptosis |
| 256892 | HP:0001773 | Short foot |

| Patient | Cluster | Shared\_hpos | Systems | Genes |
| --- | --- | --- | --- | --- |
| 256892 | 31 | HP:0003508, HP:0000508, HP:0008551 | GO:0048706 | HOXA9, HOXA6, HOXA3, HOXA1, HOXA11, HOXA4, HOXA5, HOXA7, HOXA2 |
| 256892 | 31 | HP:0003508, HP:0000508, HP:0008551 | GO:0003002 | HOXA4, EVX1, HOXA3, HOXA9, HOXA6, HOXA10, HOXA11, HOXA5, HOXA7, HOXA2 |
| 256892 | 31 | HP:0003508, HP:0000508, HP:0008551 | GO:0048705 | HOXA6, HOXA3, HOXA1, HOXA11, HOXA4, HOXA5, HOXA7, HOXA2 |
| 256892 | 73 | HP:0001156, HP:0003508, HP:0001773 | GO:0090370 | MIR148A |
| 256892 | 52 | HP:0001156, HP:0001773 | GO:1901343 | HOXA5 |
| 256892 | 54 | HP:0000494, HP:0000286 | GO:0002526 | IL6 |
| 256892 | 26 | HP:0000369, HP:0000508 | R-HSA-8957322 | OSBPL3 |
| 256892 | 55 | HP:0000286 | GO:0002526 | IL6 |
| 256892 | 74 | HP:0001773 | GO:0090370 | MIR148A |
| 256892 | 88 | HP:0000369 | GO:0002040 | HDAC9 |
| 256892 | 15 | HP:0000286 | R-HSA-500792 | NPY |
| 256892 | 12 | HP:0001363 | hsa04810 | ITGB8 |
| 256892 | 33 | HP:0000494 | hsa04630 | IL6 |
