# Supplementary material for: Phenotype-genotype comorbidity analysis of patients with rare disorders provides insight into their pathological and molecular bases
Source: PLoS Genet. 2020 Oct 1;16(10):e1009054. doi: 10.1371/journal.pgen.1009054 (PMC7553355; doi:10.1371/journal.pgen.1009054)
Supplement: S7 Report — (HTML) [file pgen.1009054.s007.html]

article\_figures.utf8.md


# Report 7: Article Figures

### Properties of the phenotype-phenotype pairs lists and random models

**Figure 3:** Properties of the Phenotype-Phenotype pairs. {A} Distribution of Hypergeometric Index values for all Phenotype-Phenotype pairs. The vertical line represents a cut-off of 2 used to separate more and less-specific pairs.{B} Prevalence distribution for all phenotypes in the DECIPHER dataset, defined percentage of patients in which a phenotype occurs. {C} Distribution of average prevalence for pairs of phenotypes in the more and less-specific pairs lists.

**Figure 4:** Comparison between the comorbid pairs and external data sources. {A} Overlap of the pairs lists with at least three known diseases from OMIM (left) and Orphanet (right). {B} Distributions of P-values obtained for the co-mention analysis, for the more-specific pairs list (left) compared to the less-specific pairs list. In both cases, the distributions are compared to those of the links random datasets. Spec: specific, uncon: unconnected, l rdm and n rdm: links-based random model and nodes-based random model. Figures generated by the PhenCo workflow.

## Emergent and consistent functional systems

**Figure 5:** Consistent and emergent functional systems from pairs from the different datasets. {A} Total numbers of pairs that show consistent or emergent functional systems for each pairs list. {B} Total numbers of emergent and consistent functional systems among the pairs lists. {C} Numbers of pairs with emergent and/or consistent functional systems. Figures generated by the PhenCo workflow.

## PhenCo finds functionally consistent clusters

**Figure 6:** Properties of the clusters found by PhenCo for GO analysis. {A} Total clusters. {B} Number of clusters where all phenotypes are found within the same OMIM disease, allowing for one missing phenotype. {C} Number of clusters for which all phenotypes show enrichment for the same GO term. {D} Number of clusters where all phenotypes are found within the same OMIM disease, allowing for one missing phenotype, and all phenotypes show enrichment for the same GO term. Figures generated by the PhenCo workflow.
