# Supplementary material for: Phenotype-genotype comorbidity analysis of patients with rare disorders provides insight into their pathological and molecular bases
Source: PLoS Genet. 2020 Oct 1;16(10):e1009054. doi: 10.1371/journal.pgen.1009054 (PMC7553355; doi:10.1371/journal.pgen.1009054)
Supplement: S1 Table — Simulations were repeated 100 times and the average was obtained. S.d: standard deviation. (PDF) [file pgen.1009054.s008.pdf]

Supplementary Table 1

	Real Data	Simulated data (mean ± s.d.)
**Phenotype-FunSys associations (GO)**	5592	942 ± 405
**Distinct phenotypes (GO)**	297	84 ± 17
**Pairs that show emergent/consistent GO terms**	705	132 ± 35
**Phenotype-FunSys associations (KEGG)**	712	166 ± 61
**Distinct phenotypes (KEGG)**	206	77 ± 16
**Pairs that show emergent/consistent KEGG terms**	273	61 ± 18
**Phenotype-FunSys associations (Reactome)**	1989	580 ± 264
**Distinct phenotypes (Reactome)**	289	80 ± 14
**Pairs that show emergent/consistent Reactome terms**	367	72 ± 19

Table 1: Comparison of real data with simulations produced by randomizing SOR loci gene content. Simulations were repeated 100 times and the average was obtained. S.d: standard deviation.
