# Supplementary material for: Phenotype-genotype comorbidity analysis of patients with rare disorders provides insight into their pathological and molecular bases
Source: PLoS Genet. 2020 Oct 1;16(10):e1009054. doi: 10.1371/journal.pgen.1009054 (PMC7553355; doi:10.1371/journal.pgen.1009054)
Supplement: S2 Table — Columns show total numbers of patients with at least 2 and 3 phenotypes in the same PhenCo cluster, for the real clusters and for clusters formed by randomizing Cluster-HPO membership, keeping the same number of HPOs per cluster and the same distribution of HPO terms. (PDF) [file pgen.1009054.s009.pdf]

Supplementary Table 2

	GO		KEGG		REACTOME	
	**Patients with ≥ 2 phenotypes:**	**Patients with ≥ 3 phenotypes:**	**Patients with ≥ 2 phenotypes:**	**Patients with ≥ 3 phenotypes:**	**Patients with ≥ 2 phenotypes:**	**Patients with ≥ 3 phenotypes:**
**PhenCo Clusters**	895.00	261.00	586.00	110.00	458.00	71.00
**Randomized Clusters**	477.15 ±105.4	32.59 ±14.9	320.08 ±89.8	33.23 ±15.2	261.15 ±89.3	15.24 ±89.3

Table 2: Overlap between DECIPHER patient phenotypes and PhenCo clusters. Columns show total numbers of patients with at least 2 and 3 phenotypes in the same PhenCo cluster, for the real clusters and for clusters formed by randomizing Cluster-HPO membership, keeping the same number of HPOs per cluster and the same distribution of HPO terms.
