# Supplementary material for: Phenotype-genotype comorbidity analysis of patients with rare disorders provides insight into their pathological and molecular bases
Source: PLoS Genet. 2020 Oct 1;16(10):e1009054. doi: 10.1371/journal.pgen.1009054 (PMC7553355; doi:10.1371/journal.pgen.1009054)
Supplement: S3 Table — The patient column refers to the number of patients for which the given method predicted at least one gene within the patient CNV. The gene column refers to the total numbers of genes predicted that overlap with the patient CNV. Phenolyzer and Orphamizer were used with thresholds of 0.8 and 0.9, as indicated. Phenomizer was run with adjusted p-value thresholds of 0.01 and 0.05. (PDF) [file pgen.1009054.s010.pdf]

Supplementary Table 3

	Patients tested	Prediction within CNV (patient)	Prediction within CNV (genes)	Total predictions made
**Phenomizer 0.05**	50	13	15	9630
**Phenomizer 0.01**	50	6	6	4766
**Phenolyzer 0.9**	3374	61	62	5986
**Phenolyzer 0.8**	3374	83	87	10329
**Orphamizer 0.9**	50	0	0	4
**Orphamizer 0.8**	50	0	0	6

Table 3: Predictions made by the different knowledge-based methods. The patient column refers to the number of patients for which the given method predicted at least one gene within the patient CNV. The gene column refers to the total numbers of genes predicted that overlap with the patient CNV. Phenolyzer and Orphamizer were used with thresholds of 0.8 and 0.9, as indicated. Phenomizer was run with adjusted p-value thresholds of 0.01 and 0.05.
