# Supplementary material for: Phenotype-genotype comorbidity analysis of patients with rare disorders provides insight into their pathological and molecular bases
Source: PLoS Genet. 2020 Oct 1;16(10):e1009054. doi: 10.1371/journal.pgen.1009054 (PMC7553355; doi:10.1371/journal.pgen.1009054)
Supplement: S4 Table — Values represent mean ± one standard deviation. Clust Coeff: Clustering Coefficient, Avg min path: Average Minimum Path, Spec: specific, l rdm and n rdm: link randomized and node randomized models respectively. (PDF) [file pgen.1009054.s011.pdf]

Supplementary Table 4

Type	Clust Coeff	Diameter	Avg min path	Total nodes
**more spec**	0.28	11	4.02	1685
**more spec n rdm**	0.28 ± 0.001	11 ± 0.001	4.02 ± 0.001	1685 ± 0.001
**more spec l rdm**	0.01 ± 0.001	6 ± 0.001	3.31 ± 0.001	1685 ± 0.001
**less spec**	0.05 ± 0.001	5 ± 0.001	2.92 ± 0.01	1383.92 ± 0.11
**less spec n rdm**	0.05 ± 0.001	5 ± 0.001	2.92 ± 0.01	1383.92 ± 0.11
**less spec l rdm**	0.03 ± 0.001	4.76 ± 0.43	2.96 ± 0.02	1383.92 ± 0.11
**Unconnected**	0.01 ± 0.001	6.16 ± 0.37	3.61 ± 0.001	1763.82 ± 0.44

Table 4: Properties of the networks formed by the different pairs lists. Values represent mean ± one standard deviation. *Clust Coeff*: Clustering Coefficient, *Avg min path*: Average Minimum Path, *Spec*: specific, *l rdm and n rdm*: link randomized and node randomized models respectively.
